# Supplementary material for: TROP2 Represents a Negative Prognostic Factor in Colorectal Adenocarcinoma and Its Expression Is Associated with Features of Epithelial–Mesenchymal Transition and Invasiveness
Source: Cancers (Basel). 2022 Aug 26;14(17):4137. doi: 10.3390/cancers14174137 (PMC9454662; doi:10.3390/cancers14174137)
Supplement: Supplementary file 1 [file cancers-14-04137-s001.zip › cancers-1854246-supplementary.pdf]

Supplementary Information

Supplementary Figure and Table Legends

Supplementary Figure S1

Tissue microarray (TMA) analysis of TROP2 expression in patients with CRC. (A) Left, an example of a TMA with 20 2-mm-diameter cylindrical samples from 10 cases; each case is represented by two samples taken from random locations on the paraffin block. Right, representative images of TROP2 immunostaining; score 0: TROP2 negative; score 4: weak TROP2 positivity, score 9: strong "patchy" positivity; score 12: strong uniform positivity in tumor tissue. Scale bar: 50 µm. (B) Categorization of TROP2 staining by total immunostaining score (TIS) and binarization into low/medium and high expression groups.

Supplementary Figure S2

Kaplan-Meier survival data for groups with low (score 0-4), intermediate (score 6-8), and high (score 9-12) TROP2 expression, censored at 5 years of follow-up, document a significant survival difference between patients with low/intermediate and high TROP2 expression ($p = 0.00073$).

Supplementary Figure S3

Bar graphs of univariate logistic regression show a positive significant correlation of high TROP2 expression (score 9-12) with high grading ($p<0.001$), mucinous and signet ring cell morphology ($p=0.025$), lymph node metastasis ($p=0.012$), advanced stage according to Union for International Cancer Control staging (UICC stage) III+IV ($p=0.047$), localization of primary CRC in the right colon ($p=0.026$), positive PD-L1≥1% ($p=0.018$), CK7 staining ≥10% ($p=0.027$). High TROP2 expression was inversely correlated with SATB2 staining >40% ($p<0.001$).

Supplementary Figure S4

(A) Principal component analysis (PCA) based on the 500 most variably expressed genes between TROP2^{high} and TROP2^{low} human colon tumor cells. Red dots represent TROP2^{high} cells, blue dots represent TROP2^{low} cells. (B) The graph shows the normalized number of *TROP2* mRNA reads detected in TROP2^{high} and TROP2^{low} human CRC cells. The boxes correspond to the first and third quartiles; the median of the relative expression values is shown as a blue line. The range of values is indicated by "whiskers" above and below each box; S12, S4, S5, and S7 refer to individual patients; colored lines connect TROP2^{high} and TROP2^{low} samples from the same patient.

Supplementary Figure S5

Trop2 protein and mRNA are increased in small intestine and colon tumors of *Apc*$^{Min/+}$ mice. (A) Immunohistochemical detection of Trop2 protein in adenomas that developed in the small intestine and

colon; the framed areas are shown at higher magnification in the images below. Membranous and cytoplasmic Trop2 localization is clearly visible throughout tumor tissue. Scale bar: 100 µm (top images); 20 µm (bottom images). (B) Quantitative RT-PCR analysis of *Trop2* expression along the rostro-caudal axis of the intestine. Total RNA was isolated from adenomas that had developed in different regions of the small or large intestine as indicated; healthy tissue was isolated from the same intestinal regions of Apc WT littermates. Two samples, i.e. biological replicates, were obtained for each type of tissue specimen. Trop2 expression was assessed in technical triplicates for each sample and normalized to the *Gapdh* expression levels. The graph shows the expression of *Trop* relative to its average expression in all samples from healthy intestine. The individual dots then represent the average value in the triplicates analyzed. Black bars represent the mean of two biological replicates.

Supplementary Figure S6

TROP2-deficient SW480 cells show increased migration. (A) Western blot analysis of whole cell lysates prepared from sorted single cell clones of DLD1 (left) and SW480 (right) human CRC cells edited with the TROP2-specific CRISPR/Cas9 system; edited clones are labeled "KO". Clones labeled "WT" were treated in the same way as KO clones except that they were transfected with a construct that did not produce TROP2-specific sgRNA, but the vector used was "empty." Results for three KO and three WT clones for each cell line are shown along with immunoblotting of the lysate of parental cells. CTBP, loading control. (B) Left, cell viability assay showed no difference in viability/proliferation between DLD1 KO/WT (n=6/5) and SW480 KO/WT (n=5/7) clones. Viability is indicated by the relative fluorescence intensity of the metabolic product of alamarBlue; the intensity at day 0 was set to 1. The experiment was performed in technical triplicates for each cell clone; mean values of relative fluorescence intensity are plotted; error bars indicate standard deviations (SD). Right, tumor formation after xenotransplantation of SW480 KO (n=3) and SW480 WT (n=3) cell clones. Immunodeficient mice of the NSG strain were injected subcutaneously with 10^6 cells into the lumbar region. The mice were sacrificed 28 days later, and the growing tumors were resected and weighed. The experiment was performed in triplicate for each cell clone. The graph shows the weight of each tumor; the mean is indicated by the black line; no significance level (ns) was reached for the difference in weight between tumors derived from WT and KO cells. (C) Wound healing assay showed increased migration of SW480 KO cells compared to WT clones. The graph shows the percentage of "healed" area 24 hours after wound formation. The experiment was performed with five KO and five WT cell clones for each cell line; six positions were photographed for each clone and time point. The median is indicated by the black line. The significance level ($p < 0.05$) was not reached for the percentage of healed area for DLD1 cells; ****, $p < 0.0001$.

Supplementary Figure S7

A wound healing assay in SW480 iTROP2 cells with doxycycline (DOX)-inducible re-expression of TROP2. (A) Cells from SW480 KO clones #3 and #4 (see Supplementary Figure S6) were transduced with a lentiviral construct that allowed DOX-inducible re-expression of TROP2. Restoration of TROP2 protein production after DOX treatment in the obtained "iTROP2" cells was verified by Western; arrow indicates the putative TROP2 C-terminal fragment; Vinculin, loading control. (B) Left, an example of the wound healing assay; shown are representative images of scratched areas immediately after application of the "wound" and 24 and 48 hours later. The top images show the results obtained with the parental SW480 cells and TROP2 KO clone #4. The lower images show the assay with the iTROP2

#4/5 subclone cultured with or without DOX. Right, a graph showing the percentage of "healed" area 24 and 48 hours after wound formation. The experiment was performed with seven iTROP2 clones derived from KO clone #3; six positions were photographed for each clone and time point. Each dot represents one measurement, bars indicate the average, error bars represent SDs ; *, $p < 0.05$; **, $p < 0.01$.

Supplementary Table S1

Patients and histopathological characteristics of clinical samples. Hyp, hyperplastic polyps; LGD, adenomas with low-grade dysplasia; HGD, adenomas with high-grade dysplasia; CRC, invasive carcinoma.

Supplementary Table S2

Oligonucleotides and primers used in the study

Supplementary Table S3

Patient characteristics and correlation of TROP2 expression scores with clinicopathological parameters in CRC patients. Gene abbreviations: CK7, cytokeratin 7; CK20, cytokeratin 20; PD-L1, programmed death ligand; SATB2, special AT-rich sequence binding protein 2. Other abbreviations: CI, confidence interval; NOS, not otherwise specified; SD, standard deviation; UICC, Union for International Cancer Control.

Supplementary Table S4

Differentially expressed genes in $Trop2^{high}$ compared to $Trop2^{low}$ tumor cells. Genes with significantly altered expression in hyperplastic epithelium 7 days and in adenomas 6 weeks after Apc inactivation are listed on the appropriate sheet according to whether they were upregulated or downregulated. Genes with an adjusted p-value < 0.05 in both experiments were considered significant; pseudogenes were removed from the lists.

Supplementary Table S5

Differentially expressed genes (DEGs) in two populations of RFP-positive, i.e. proliferating cells sorted for Trop2 expression, and in a population of RFP-negative, i.e. differentiated cells sorted from hyperplastic epithelium 7 days after Apc inactivation. Genes with an adjusted p-value < 0.05 and $|\log_2 FC| \geq 1$ were considered significant. DEGs between $Trop2^+RFP^+$ and $Trop2^-RFP^+$ cells and between the two RFP^+ cell-derived gene expression profiles and the RFP^- cell dataset are shown on the corresponding sheet.

Supplementary Table S6

Differentially expressed genes (DEGs) in two populations of tdTomato-positive tumor cells sorted for Trop2 expression and in a population of tdTomato-negative (differentiated) cells obtained from

microroadenomas 6 weeks after Apc inactivation. DEGs between Trop2^{+} tdTomato^{+} and Trop2^{-} tdTomato^{+} cells and between the two tdTomato-positive cell groups and tdTomato-negative cells are shown on the corresponding sheets. Genes with an adjusted p-value < 0.05 and $|\log_2 FC| \geq 1$ were considered significant. Wnt-responsive genes are highlighted in red.

Supplementary Table S7

Overlap of differentially expressed genes in Trop2^{+}RFP/tdTomato^{+} vs Trop2^{-}RFP/tdTomato^{+} neoplastic cells obtained in hyperplastic epithelium and adenomas 7 days and 6 weeks after Apc inactivation, respectively. Genes with adjusted p-value < 0.05 and $|\log_2 FC| \geq 1$ were used for the comparison.

Supplementary Figure S1

Tissue microarray (TMA) analysis of TROP2 expression in patients with CRC

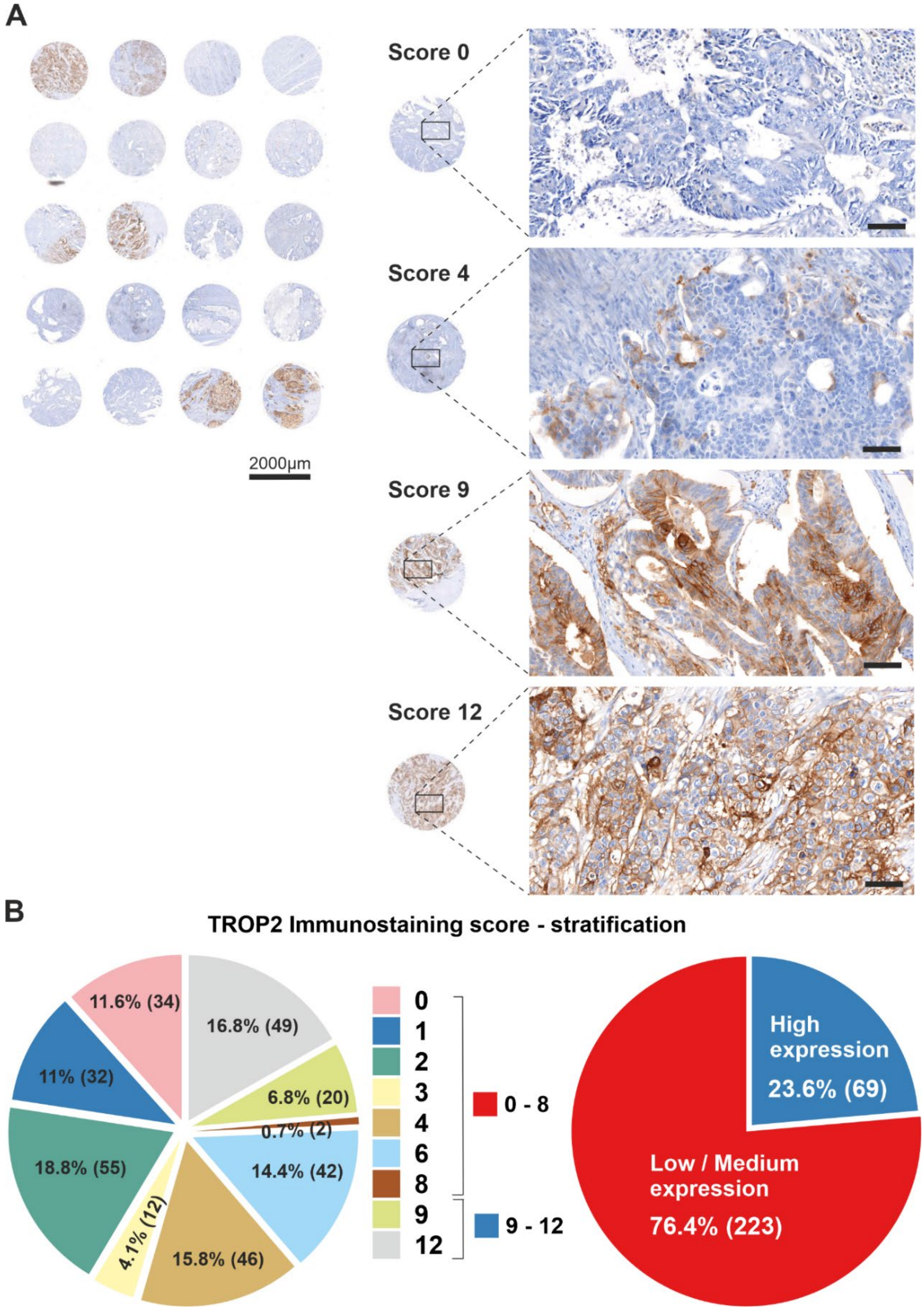

Supplementary Figure S2

Kaplan–Meier survival data for groups with low (score 0–4), intermediate (score 6–8), and high (score 9–12) TROP2 expression

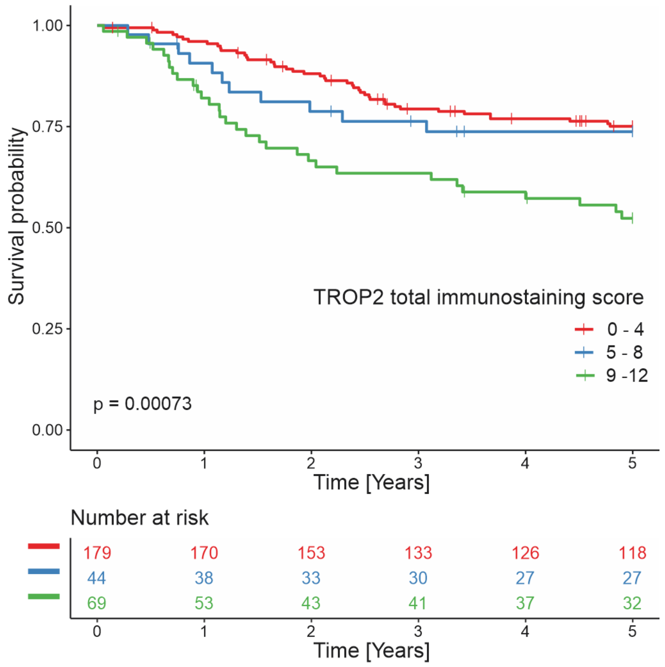

Supplementary Figure S3

Bar graphs of univariate logistic regression show a positive significant correlation of high TROP2 expression with high grading and lymph node metastasis

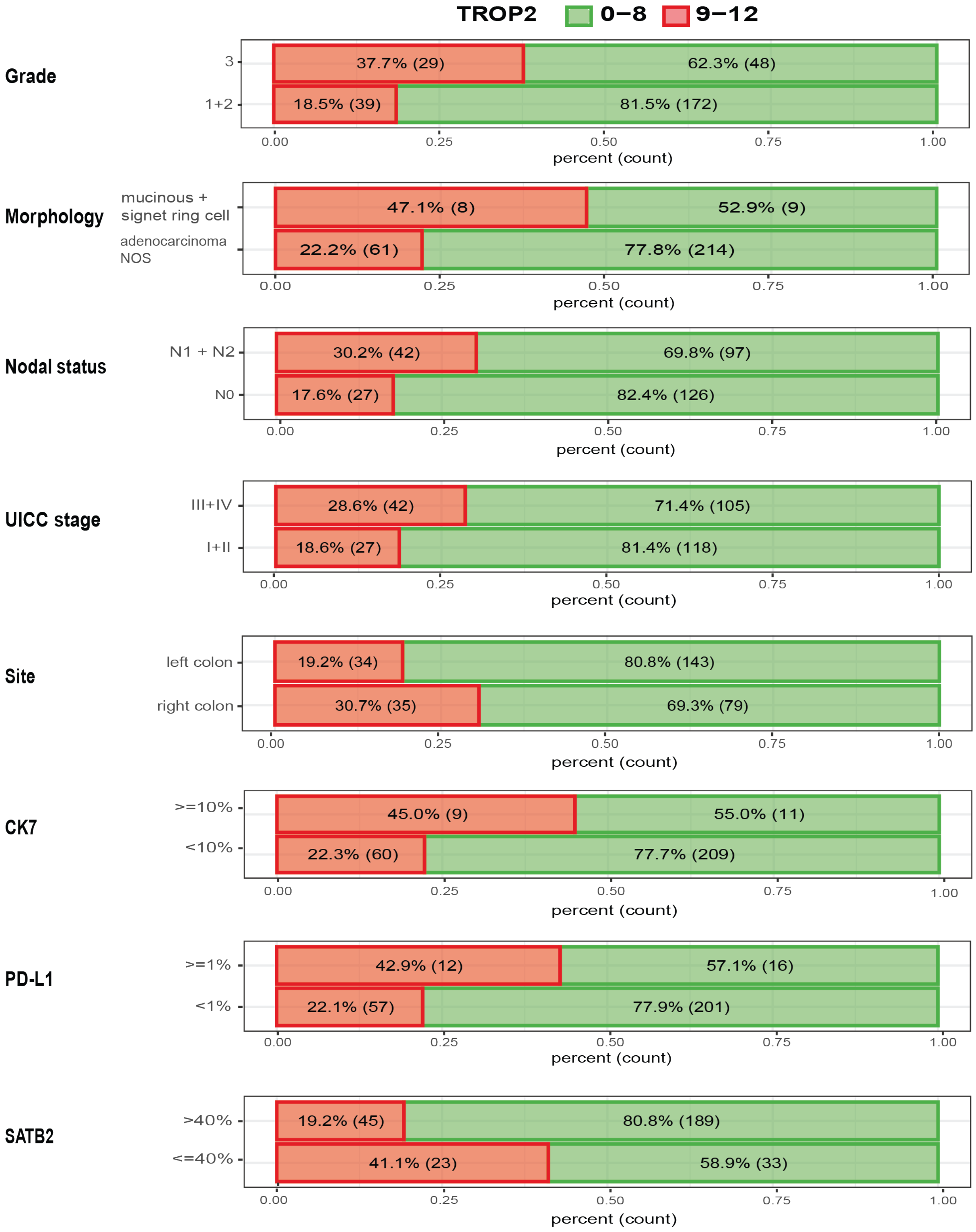

Supplementary Figure S4

Principal component analysis (PCA) based on the 500 most variably expressed genes between TROP2^{high} and TROP2^{low} human colon tumor cells

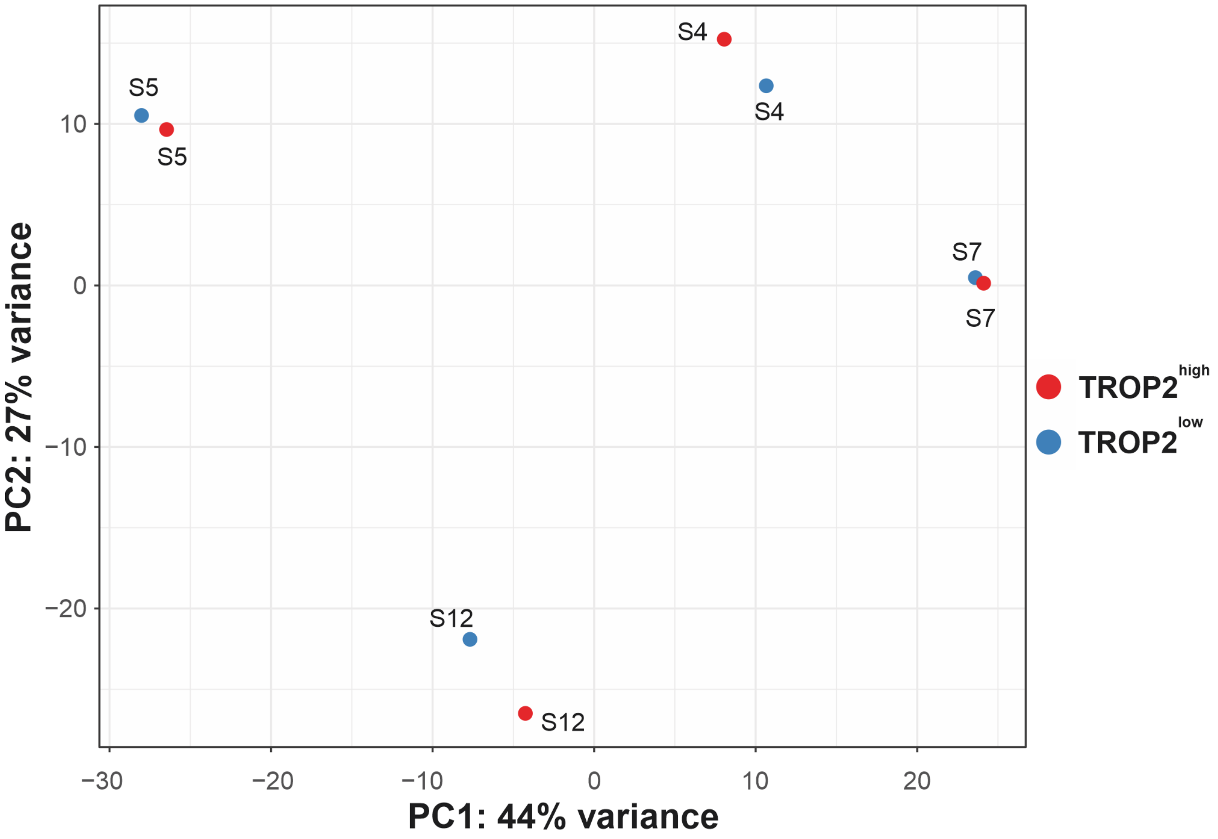

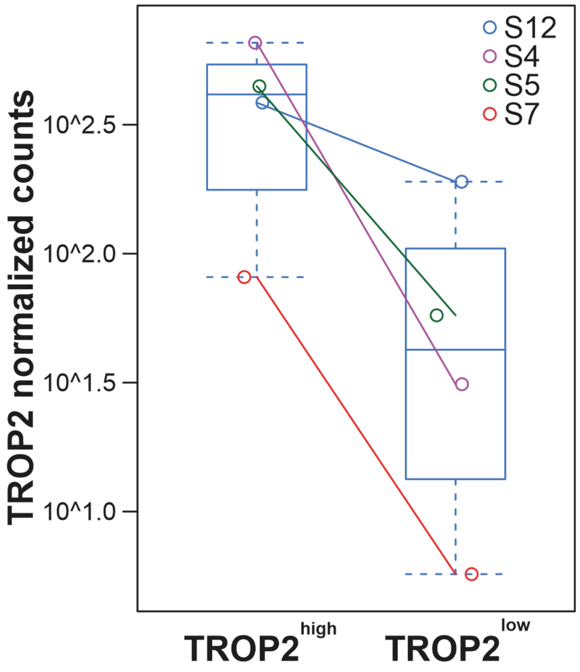

Supplementary Figure S5

Trop2 protein and mRNA are increased in small intestine and colon tumors of *Apc*$^{Min/+}$ mice

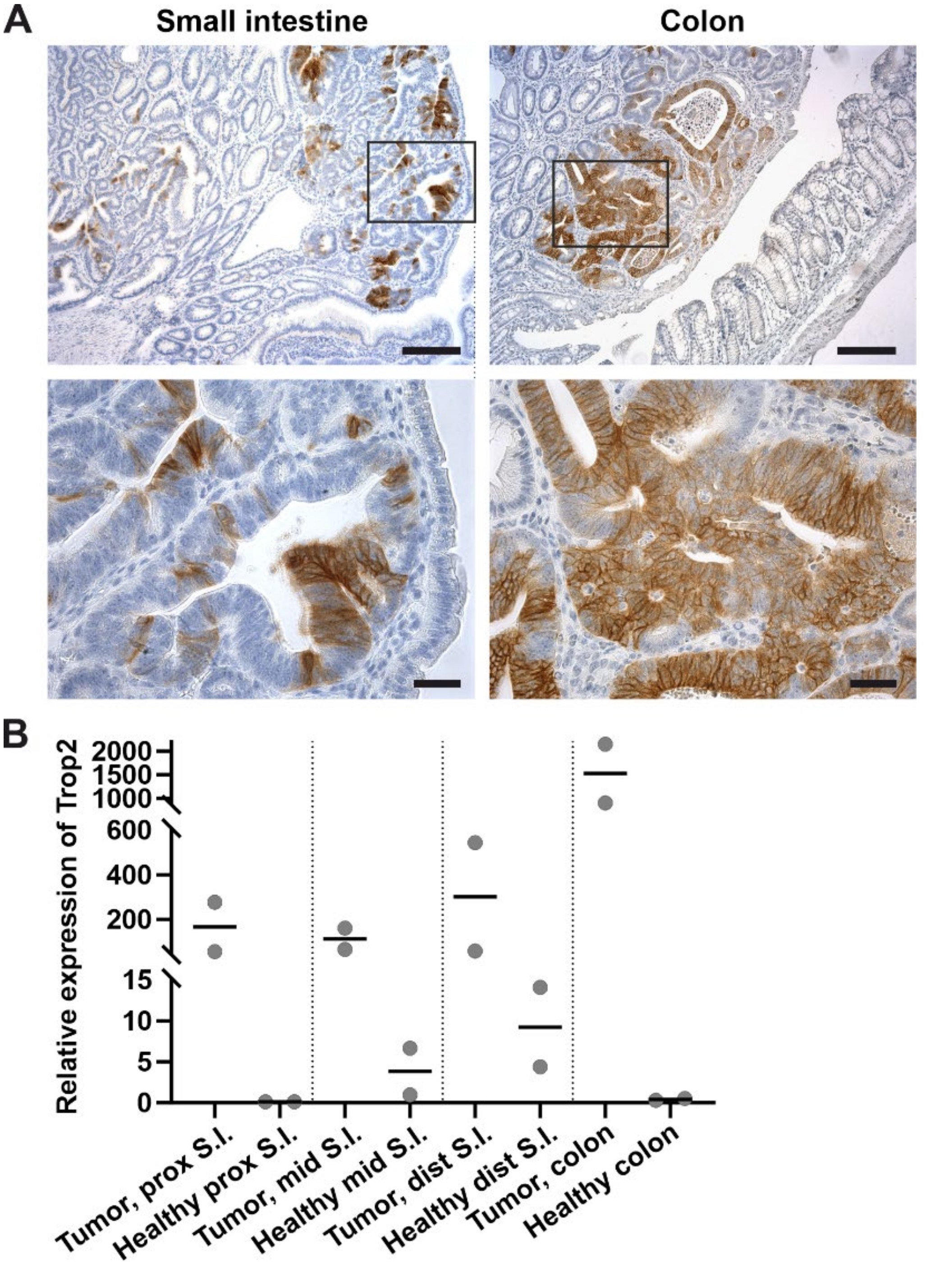

Supplementary Figure S6

TROP2-deficient SW480 cells show increased migration

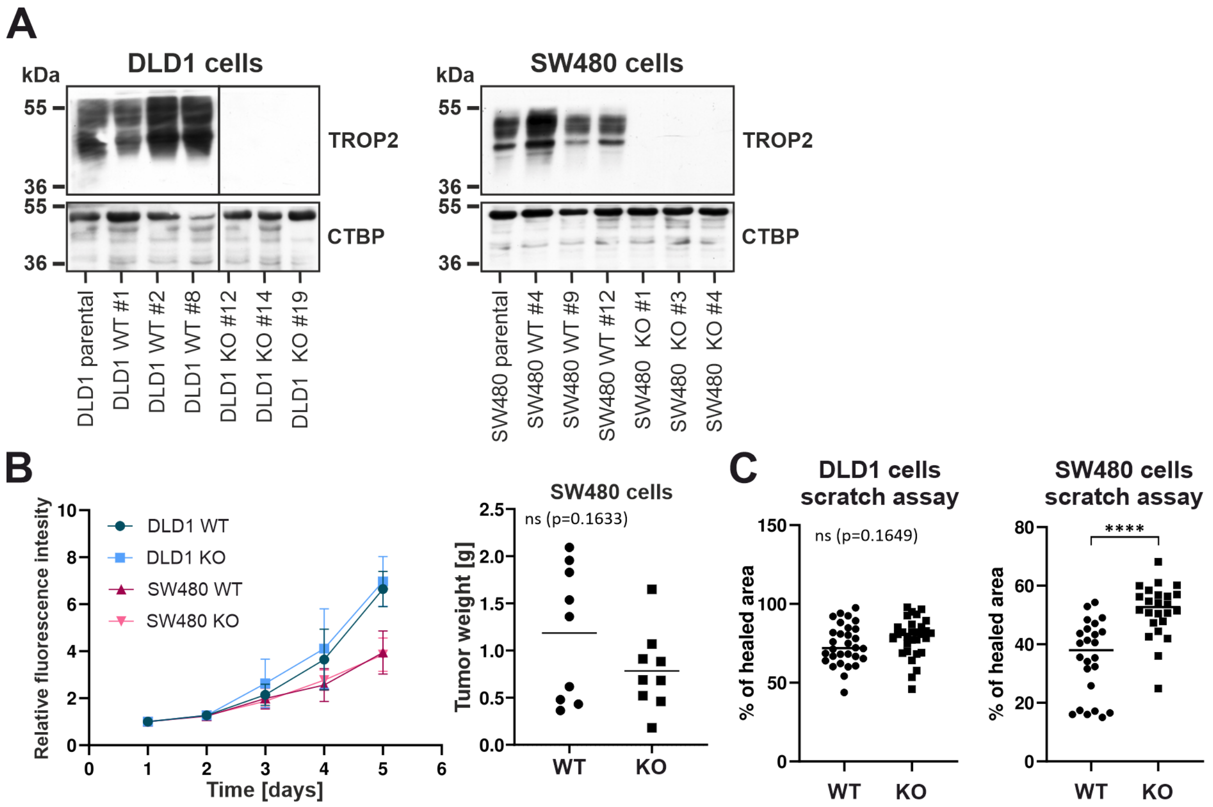

Supplementary Figure S7

A wound healing assay in SW480 iTROP2 cells with doxycycline-inducible re-expression of TROP2

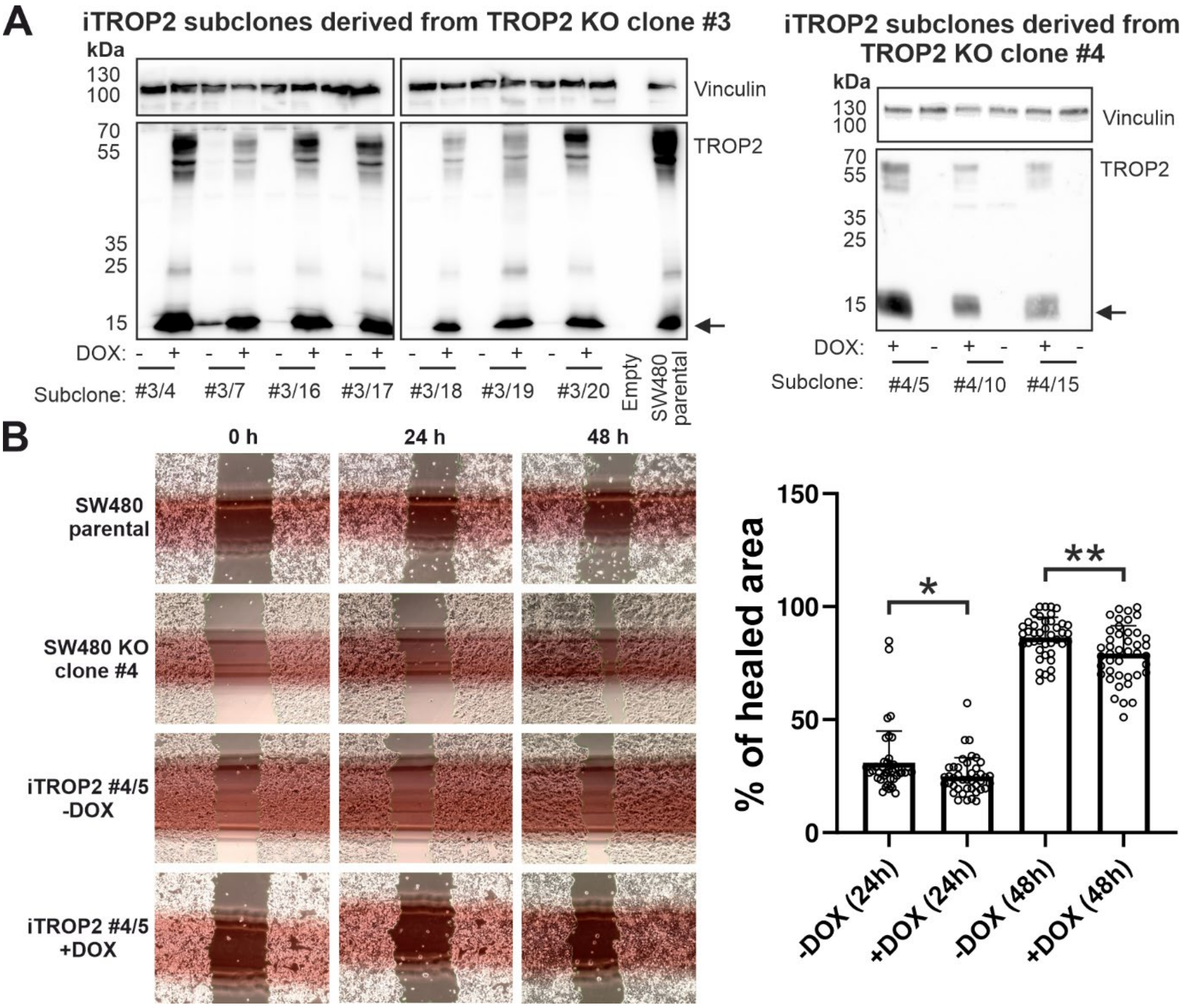

Supplementary Table S1

Patients and histopathological characteristics of clinical samples

Histology	Age (median, min.-max.)	Gender (M/F)
HYP	69 (59-77)	6/3
LGD	67 (53-89)	12/15
HGD	64 (36-85)	15/9
CRC	82 (63-90)	6/6

Supplementary Table S2

Oligonucleotides and primers used in the study

Primers for qRT-PCR on mouse tissue

Gene symbol	Organism	Forward primer sequence (5′ to 3′ orientation)	Reverse primer sequence (5′ to 3′ orientation)
β-actin	Mouse	GATCTGGCACCACACCTTCT	GGGGTGTTGAAGGTCTCAAA
GAPDH	Mouse	AACTTTGGCATTGTGGAAGG	ATCCACAGTCTTCTGGGTGG
Ly6a/Sca1	Mouse	TCTTGTGGCCCTACTGTGTG	GGCAGATGGGTAAGCAAAGA
Olfm4	Mouse	TGGGCAGAAGGTGGGACTGTGT	TGTCAGCGGGAAAGGCGGTA
Trop2	Mouse	GAACCCACCACATCCTCATT	ATGGTGGGCTCCTCATAGTG
Ubb	Mouse	ATGTGAAGGCCAAGATCCAG	TAATAGCCACCCCTCAGACG

Primers for qRT-PCR on human organoids

Gene symbol	Organism	Forward primer sequence (5′ to 3′ orientation)	Reverse primer sequence (5′ to 3′ orientation)
ALPI	Human	GCGGCTTCTACCTCTTTGTG	AAGCCACACCCTCATGATGA
ANKRD1	Human	AGTAGAGGAACTGGTCACTGG	TGGGCTAGAAGTGTCTTCAGAT
ANXA1	Human	GGTGACCGATCTGAGGACTT	ACCTCTGCGAAGTTGTGGAT
ASCL2	Human	GCGAGCTACTCGACTTCTCC	CTCGGCTTCCGGGGCTGAGG
AXIN2	Human	TGAGGTCCACGGAAACTGTTGACAGT	CCCTCCCGCGAATTGAGTGTGA
β-ACTIN	Human	GGCATCCTCACCCTGAAGTA	AGGTGTGGTGCCAGATTTTC
CTGF	Human	CCTGCAGGCTAGAGAAGCAG	TGGAGATTTTGGGAGTACGG
CTSE	Human	GGAATTCTGGGCCTGGGATA	TCTGACTGCCAAGCCTTGAT
CYR61	Human	CCTTGTGGACAGCCAGTGTA	ACTTGGGCCGGTATTTCTTC
E-CADHERIN	Human	CAGTCAAAAGGCCTCTACGG	GGCGTAGACCAAGAAATGGA
E2F1	Human	TCACCACCACCATCATCTCC	TGGGGAAAGGCTGATGAACT
LGR5	Human	CTCTTCCTCAAACCGTCTGC	GATCGGAGGCTAAGCAACTG
SCA1/LY6a	Human	TTTCTTCGCTTGCTCCTCAC	CCACTGTTTCATCCCTTGGC
SIM	Human	ATGTGAAGGTTGCCCAAAAC	AAAATTGGCCATGTTTTCCA
TEAD2	Human	GCCTCCGAGAGCTATATGATCG	TCACTCCGTAGAAGCCACCA
TROP2	Human	ACAACGATGGCCTCTACGAC	TCCAGGTCTGAGTGGTTGAA
VIMENTIN	Human	TGCCCTTAAAGGAACCAATG	TCCAGCAGCTTCCTGTAGGT

Oligos for single-guide RNA sequences

**Oligo name**	**5´- 3´ primer sequence**
FhTROP2 lentiCRISPR_a	CACCGTGGGGCGGAGGAACGCGGAC
RhTROP2 lentiCRISPR_a	AAACGTCCGCGTTCCTCCGCCCCAC
FhTROP2 lentiCRISPR_b	CACCGACACGTGCAGTTGTCCTGCG
RhTROP2 lentiCRISPR_b	AAACCGCAGGACAACTGCACGTGTC
FhTROP2 pARv-RFP_a	GTGGGGCGGAGGAACGCGGACCGGAT
RhTROP2 pARv-RFP_a	CCGGTCCGCGTTCCTCCGCCCCAC
FhTROP2 pARv-RFP_b	GACACGTGCAGTTGTCCTGCGCGGAT
RhTROP2 pARv-RFP_b	CCGCGCAGGACAACTGCACGTGTC

TROP2 genotyping PCR primers

**Oligo name**	**5´- 3´ primer sequence**
FhTROP2 genotyping	CCTGCAGACCATCCCAGAC
RhTROP2 genotyping	TGCACACGGTCATCTTGTTG

Sequencing primers

**Vector**	**5´- 3´ primer sequence**
pARv-RFP	CACCGCTAATTCAAAGCAACCG
lentiCRISPRv2	CTACTATTCTTTCCCCTGCACTGTAC
pGEM T-easy	GATTTAGGTGACACTATAG

Quantitative RT-PCR primers and UPL probes for analysis of human tissue samples

Gene	5´- 3´ primer sequence	UPL- probe No.
FhTROP2	cgcaaaggagacgtttatcc	8
RhTROP2	ctggtgtgtgcgcaaaag	
FhUBB	aggatcctggtatccgctaac	39
RhUBB	tcacattttcgatggtgtcact	
FhTBP	gaacatcatggatcagaacaaca	87
RhTBP	atagggattccgggagtcat	

Supplementary Table S3

Patient characteristics and correlation of TROP2 expression scores with clinicopathological parameters in CRC patients

		TROP2 expression (%)		Univariate analysis by logistic regression	
**Variable**	**Number (%)**	**Low/Medium score (0 - 8)**	**High score (9 - 12)**	**Odds ratio (95% CI)**	**p-value**
**Age at diagnosis (n = 292; 100%)**					
mean ± SD	68.5 ± 11				
< 75 years	205 (70.2%)	156 (76.0%)	49 (24%)		
≥ 75 years	87 (29,8%)	67 (77%)	20 (23%)	0.97 (0.51 - 1.69)	0.85
**Gender (n = 292; 100%)**					
female	129 (44.2%)	93 (72.1%)	36 (27.9%)		
male	163 (55.8%)	130 (79.8%)	33 (20.2%)	0.66 (0.38 - 1.13)	0.127
**Site (n = 291; 99.7%)**					
left colon	178 (61%)	143 (80.8%)	34 (19.2%)		
right colon	113 (38.7%)	79 (69.3%)	35 (30.7%)	1.85 (1.08 - 3.23)	0.026
**Morphology (n = 292; 100%)**					
adenocarcinoma, NOS	275 (94.2%)	214 (77.8%)	61 (22.2%)		
mucinous + signet ring cell	17 (5.8%)	9 (52.9%)	8 (47.1%)	3.12 (1.13 - 8.50)	0.025
**Grade (n = 288; 98.7%)**					
1+2	211 (72.3%)	172 (81.5%)	39 (18.5%)		
3	77 (26.4%)	48 (62.3%)	29 (37.7%)	2.67 (1.50 - 4.75)	<0.001
**Lymph node status (n = 292; 100%)**					
N0	153 (52.4%)	126 (82.4%)	27 (17.6%)		
N1+N2	139 (47.6%)	97 (69.8%)	42 (30.2%)	2.02 (1.17 - 3.54)	0.012
**UICC stage (n = 292; 100%)**					
I+II	145 (49.7%)	118 (81.4%)	27 (18.6%)		
III+IV	147 (50.3%)	105 (71.4%)	42 (28.6%)	1.75 (1.01 - 3. 06)	0.047
**Therapy (n = 291; 99.7%)**					
none	132 (45.2%)	104 (78.8%)	28 (21.2%)		
neoadjuvant	40 (13,7%)	33 (82.5%)	7 (17.5%)		
adjuvant and/or paliative	119 (40.8%)	85 (71.4%)	34 (28.6%)	1.29 (0.75 - 2.25)	0.362
**PD-L1 (n = 284; 98%)**					
< 1%	258 (88.4%)	201 (77.9%)	57 (22.1%)		
≥ 1%	28 (9.6%)	16 (57.1%)	12 (42.9%)	2.65 (1.16 - 5.89)	0.018
**CK7 (n = 289; 98.9%)**					
< 10%	269 (92.1%)	209 (77.7‘%)	60 (22.3%)		
≥ 10%	20 (6.8%)	11 (55%)	9 (45%)	2.85 (1.10 - 7.21)	0.027
**CK20 (n = 289; 98,9%)**					
< 25%	67 (22.9%)	45 (67.2%)	22 (32.8%)		
≥ 25%	222 (76%)	175 (78.8%)	47 (21.2%)	0.55 (0.30 - [illegible]	0.052

				1.01)	

Supplementary Table S4
Differentially expressed genes in Trop2high compared to Trop2low tumor cells

Differentially expressed genes (DEGs) in $Trop2^{high}$ compared to $Trop2^{low}$ tumor cells.
Genes with significantly altered expression in hyperplastic epithelium 7 days and in adenomas 6 weeks after Apc inactivation are listed on the appropriate sheet according to whether they were upregulated (72 genes) or downregulated (31 genes). Genes with an adjusted p-value < 0.05 in both experiments were considered significant; pseudogenes were removed from the lists.

Upregulated genes

Ensembl gene ID	Symbol		log2FC	p-value	FDR	Mean expression
ENSG00000198211	AC092143.1	novel protein (MC1R-TUBB3 readthrough)	3.69E+00	7.15E-06	2.35E-03	1.21
ENSG00000184292	TACSTD2	tumor associated calcium signal transducer 2	2.89E+00	7.75E-09	5.68E-06	2.36
ENSG00000131016	Up	A-kinase anchoring protein 12	2.59E+00	1.94E-09	1.65E-06	2.31
ENSG00000277991	FP236241.1	uncharacterized LOC102723360	2.12E+00	2.81E-05	7.07E-03	1.64
ENSG00000284419	MIR663A	microRNA 663a	1.55E+00	2.28E-18	3.68E-14	3.58
ENSG00000196188	CTSE	cathepsin E	1.52E+00	2.30E-07	1.16E-04	2.52
ENSG00000249624	AP000295.1	novel protein	1.41E+00	5.75E-05	1.27E-02	1.76
ENSG00000212443	SNORA53	small nucleolar RNA, H/ACA box 53	1.33E+00	5.50E-10	5.21E-07	2.6
ENSG00000199270	RNA5S12	RNA, 5S ribosomal 12	1.28E+00	4.03E-12	6.88E-09	3.19
ENSG00000199396	RNA5S5	RNA, 5S ribosomal 5	1.28E+00	4.03E-12	6.88E-09	3.19
ENSG00000201925	RNA5S15	RNA, 5S ribosomal 15	1.28E+00	4.03E-12	6.88E-09	3.19
ENSG00000137673	MMP7	matrix metallopeptidase 7	1.27E+00	2.70E-06	1.06E-03	2.72
ENSG00000206941	SNORD15A	small nucleolar RNA, C/D box 15A	1.24E+00	6.29E-13	3.38E-09	3.38
ENSG00000264462	MIR3648-2	microRNA 3648-2	1.22E+00	1.58E-12	6.03E-09	4.36
ENSG00000198695	MT-ND6	mitochondrially encoded NADH:ubiquinone oxidoreductase core subunit 6	1.07E+00	5.01E-07	2.35E-04	3.32
ENSG00000201998	SNORA23	small nucleolar RNA, H/ACA box 23	1.06E+00	7.82E-08	4.50E-05	2.69
ENSG00000227195	MIR663AHG	MIR663A host gene	1.06E+00	1.19E-05	3.62E-03	4.89
ENSG00000201321	RNA5S9	RNA, 5S ribosomal 9	1.05E+00	5.26E-06	1.97E-03	2.54
ENSG00000201403	SNORD14B	small nucleolar RNA, C/D box 14B	1.00E+00	3.13E-05	7.63E-03	2.45
ENSG00000122861	PLAU	plasminogen activator, urokinase	9.71E-01	6.09E-05	1.31E-02	1.95
ENSG00000023445	BIRC3	baculoviral IAP repeat containing 3	9.56E-01	2.22E-06	9.16E-04	2.71
ENSG00000196924	FLNA	filamin A	9.50E-01	3.26E-08	1.94E-05	3.62
ENSG00000202252	SNORD14C	small nucleolar RNA, C/D box 14C	9.45E-01	2.05E-05	5.69E-03	2.73
ENSG00000011422	PLAUR	plasminogen activator, urokinase receptor	9.41E-01	5.53E-06	1.98E-03	2.78
ENSG00000198888	MT-ND1	mitochondrially encoded NADH:ubiquinone oxidoreductase core subunit 1	9.33E-01	1.87E-12	6.03E-09	5.21
ENSG00000212907	MT-ND4L	mitochondrially encoded NADH:ubiquinone oxidoreductase core subunit 4L	9.23E-01	2.36E-11	3.16E-08	4.3
ENSG00000140279	DUOX2	dual oxidase 2	8.90E-01	1.21E-08	7.83E-06	3.66
ENSG00000277947	SNORD3D	small nucleolar RNA, C/D box 3D	8.76E-01	1.38E-07	7.43E-05	4.39
ENSG00000198727	MT-CYB	mitochondrially encoded cytochrome b	8.75E-01	4.47E-10	4.80E-07	5.15
ENSG00000228253	MT-ATP8	mitochondrially encoded ATP synthase membrane subunit 8	8.67E-01	1.56E-09	1.39E-06	4.54
ENSG00000135046	ANXA1	annexin A1	8.67E-01	5.92E-05	1.29E-02	2.7
ENSG00000129451	KLK10	kallikrein related peptidase 10	8.52E-01	9.48E-05	1.86E-02	1.87
ENSG00000053747	LAMA3	laminin subunit alpha 3	8.15E-01	2.84E-06	1.09E-03	3.32
ENSG00000169908	TM4SF1	transmembrane 4 L six family member 1	8.03E-01	1.32E-06	5.77E-04	2.92
ENSG00000134531	EMP1	epithelial membrane protein 1	7.90E-01	9.91E-05	1.92E-02	2.95
ENSG00000135074	ADAM19	ADAM metallopeptidase domain 19	7.80E-01	2.38E-05	6.29E-03	2.99
ENSG00000222041	CYTOR	cytoskeleton regulator RNA	7.75E-01	1.67E-04	2.89E-02	2.15
ENSG00000074276	CDHR2	cadherin related family member 2	7.36E-01	8.66E-06	2.73E-03	2.92
ENSG00000058085	LAMC2	laminin subunit gamma 2	7.28E-01	5.63E-06	1.98E-03	3.57
ENSG00000102287	GABRE	gamma-aminobutyric acid type A receptor subunit epsilon	7.03E-01	2.80E-05	7.07E-03	3.01
ENSG00000207304	SNORA8	small nucleolar RNA, H/ACA box 8	7.02E-01	8.51E-05	1.71E-02	2.61
ENSG00000263740	RN7SL4P	RNA, 7SL, cytoplasmic 4, pseudogene	6.94E-01	3.26E-05	7.78E-03	3.56
ENSG00000198840	MT-ND3	mitochondrially encoded NADH:ubiquinone oxidoreductase core subunit 3	6.85E-01	5.11E-07	2.35E-04	4.26
ENSG00000200959	SNORA74A	small nucleolar RNA, H/ACA box 74A	6.83E-01	2.84E-04	4.19E-02	2.35
ENSG00000245532	NEAT1	nuclear paraspeckle assembly transcript 1	6.70E-01	8.80E-09	6.03E-06	4.31
ENSG00000252010	SCARNA5	small Cajal body-specific RNA 5	6.53E-01	1.44E-04	2.58E-02	2.93
ENSG00000163739	CXCL1	C-X-C motif chemokine ligand 1	6.52E-01	1.27E-05	3.78E-03	3.01
ENSG00000207241	SNORD45A	small nucleolar RNA, C/D box 45A	6.38E-01	3.44E-04	4.84E-02	2.51
ENSG00000172927	MYEOV	myeloma overexpressed	6.31E-01	2.09E-04	3.43E-02	2.55
ENSG00000254341	SNORD87	small nucleolar RNA, C/D box 87	6.26E-01	1.99E-04	3.31E-02	2.66
ENSG00000267322	SNHG22	small nucleolar RNA host gene 22	6.19E-01	1.20E-04	2.24E-02	2.8
ENSG00000142627	EPHA2	EPH receptor A2	5.86E-01	5.65E-06	1.98E-03	3
ENSG00000204616	TRIM31	tripartite motif containing 31	5.68E-01	1.68E-05	4.83E-03	3.15
ENSG00000238917	SNORD10	small nucleolar RNA, C/D box 10	5.63E-01	8.45E-05	1.71E-02	3.96
ENSG00000280138	AC027290.2	novel transcript	5.40E-01	7.89E-05	1.63E-02	2.96
ENSG00000206588	RNU1-28P	RNA, U1 small nuclear 28, pseudogene	5.20E-01	3.00E-05	7.43E-03	3.87
ENSG00000173559	NABP1	nucleic acid binding protein 1	5.04E-01	2.79E-04	4.16E-02	2.69
ENSG00000130707	ASS1	argininosuccinate synthase 1	4.73E-01	3.61E-04	4.93E-02	3.38
ENSG00000178209	PLEC	plectin	4.53E-01	6.96E-07	3.11E-04	4.3
ENSG00000130164	LDLR	low density lipoprotein receptor	4.44E-01	2.56E-05	6.66E-03	3.34
ENSG00000210082	MT-RNR2	mitochondrially encoded 16S rRNA	4.23E-01	1.51E-04	2.68E-02	5.62
ENSG00000090382	LYZ	lysozyme	4.19E-01	1.21E-04	2.24E-02	3.55
ENSG00000124942	AHNAK	AHNAK nucleoprotein	4.08E-01	9.60E-06	2.97E-03	4.26
ENSG00000123358	NR4A1	nuclear receptor subfamily 4 group A member 1	4.07E-01	2.70E-04	4.06E-02	3.28
ENSG00000182718	ANXA2	annexin A2	3.95E-01	2.31E-04	3.65E-02	3.84
ENSG00000267519	AC020916.1	novel transcript	3.85E-01	8.89E-05	1.77E-02	3.34
ENSG00000173898	SPTBN2	spectrin beta, non-erythrocytic 2	3.80E-01	1.60E-04	2.80E-02	3.2
ENSG00000112715	VEGFA	vascular endothelial growth factor A	3.77E-01	3.81E-05	8.77E-03	3.68
ENSG00000092820	EZR	ezrin	3.41E-01	4.20E-05	9.39E-03	3.81
ENSG00000062716	VMP1	vacuole membrane protein 1	3.05E-01	3.30E-04	4.70E-02	3.68
ENSG00000151914	DST	dystonin	3.03E-01	2.59E-04	3.98E-02	3.83
ENSG00000197956	S100A6	S100 calcium binding protein A6	2.95E-01	3.53E-04	4.85E-02	4.07

Downregulated genes

Ensembl gene ID	Symbol		log2FC	p-value	adjusted p-value	Mean expression
ENSG00000276345	AC004556.3	39S ribosomal protein L23, mitochondrial	-2.87E-01	3.49E-04	4.84E-02	2.29
ENSG00000169100	SLC25A6	solute carrier family 25 member 6	-2.88E-01	1.02E-04	1.95E-02	3.71
ENSG00000165238	WNK2	WNK lysine deficient protein kinase 2	-2.97E-01	2.56E-04	3.98E-02	3.64
ENSG00000135916	ITM2C	integral membrane protein 2C	-3.23E-01	1.29E-04	2.36E-02	3.45
ENSG00000198643	FAM3D	FAM3 metabolism regulating signaling molecule D	-4.39E-01	2.62E-04	3.98E-02	3.17
ENSG00000267022	AC067968.1	novel protein	-4.72E-01	3.25E-04	4.67E-02	1.78
ENSG00000135503	ACVR1B	activin A receptor type 1B	-4.84E-01	1.88E-04	3.18E-02	2.99
ENSG00000237356	AL365295.1	novel transcript	-4.88E-01	2.94E-04	4.30E-02	1.33
ENSG00000204219	TCEA3	transcription elongation factor A3	-5.12E-01	2.33E-05	6.29E-03	3.06
ENSG00000188803	SHISA6	shisa family member 6	-5.27E-01	3.05E-04	4.43E-02	2.11
ENSG00000101333	PLCB4	phospholipase C beta 4	-5.32E-01	6.29E-05	1.32E-02	3.58
ENSG00000174992	ZG16	zymogen granule protein 16	-5.74E-01	2.19E-04	3.49E-02	2.24
ENSG00000256028	AC026362.1	novel transcript	-5.89E-01	2.14E-04	3.46E-02	1.47
ENSG00000148057	IDNK	IDNK gluconokinase	-6.18E-01	2.15E-04	3.46E-02	1.51
ENSG00000116299	ELAPOR1	endosome-lysosome associated apoptosis and autophagy regulator 1	-7.16E-01	3.28E-05	7.78E-03	3.22
ENSG00000162896	PIGR	polymeric immunoglobulin receptor	-7.17E-01	2.21E-11	3.16E-08	4.85
ENSG00000074370	ATP2A3	ATPase sarcoplasmic/endoplasmic reticulum Ca2+ transporting 3	-8.34E-01	1.43E-08	8.88E-06	3.18
ENSG00000117983	MUC5B	mucin 5B, oligomeric mucus/gel-forming	-8.54E-01	8.28E-08	4.60E-05	4.47
ENSG00000206127	GOLGA8O	golgin A8 family member O	-9.63E-01	1.11E-04	2.10E-02	1.41
ENSG00000167080	B4GALNT2	beta-1,4-N-acetyl-galactosaminyltransferase 2	-9.65E-01	6.18E-05	1.31E-02	2.51
ENSG00000198788	MUC2	mucin 2, oligomeric mucus/gel-forming	-9.73E-01	8.99E-09	6.03E-06	5.3
ENSG00000128917	DLL4	delta like canonical Notch ligand 4	-9.81E-01	7.62E-06	2.45E-03	2.53
ENSG00000174136	RGMB	repulsive guidance molecule BMP co-receptor b	-9.86E-01	5.94E-06	2.04E-03	2.9
ENSG00000073849	ST6GAL1	ST6 beta-galactoside alpha-2,6-sialyltransferase 1	-1.21E+00	4.09E-10	4.70E-07	3.03
ENSG00000112562	SMOC2	SPARC related modular calcium binding 2	-1.64E+00	5.24E-10	5.21E-07	2.65
ENSG00000267881	AC243967.1	novel protein, readthrough between CEACAM5-CEACAM6	-1.88E+00	3.50E-05	8.16E-03	1.66
ENSG00000261771	DNAAF4-CCPG1	DNAAF4-CCPG1 readthrough (NMD candidate)	-2.26E+00	2.37E-05	6.29E-03	1.27
ENSG00000277150	F8A3	coagulation factor VIII associated 3	-2.46E+00	1.77E-05	5.00E-03	1.57
ENSG00000118785	SPP1	secreted phosphoprotein 1	-5.06E+00	4.30E-09	3.30E-06	1.73
ENSG00000250151	ARPC4-TTLL3	ARPC4-TTLL3 readthrough	-5.53E+00	1.47E-07	7.61E-05	1.67
ENSG00000100604	CHGA	chromogranin A	-6.32E+00	3.12E-07	1.52E-04	2.13

Supplementary Table S5

Differentially expressed genes in two populations of RFP-positive, i.e., proliferating cells sorted for Trop2 expression, and in a population of RFP-negative, i.e., differentiated cells sorted from hyperplastic epithelium 7 days after Apc inactivation

Differentially expressed genes (DEGs) in two populations of RFP-positive, i.e. proliferating cells sorted for Trop2 expression, and in a population of RFP-negative, i.e. differentiated cells sorted RFP dataset from hyperplastic epithelium 7 days after Apc inactivation. Genes with an adjusted p-value < 0.05 and a log change $|\log_2 FC| \geq 1$ were considered significant. DEGs between $Trop2^{+}RFP^{+}$ and $Trop2^{-}RFP^{+}$ cells and between the two RFP^{+} cell-derived gene expression profiles the RFP^{-} cells dataset and the are shown on the corresponding sheet.

RFP+Trop2+ vs. RFP+Trop2- cells

Ensemble gene code	Symbol	Gene name	logFC
ENSMUSG00000029304	Spp1	secreted phosphoprotein 1	7.42
ENSMUSG00000027485	Bpifb1	BPI fold containing family B, member 1	6.53
ENSMUSG00000051397	Tacstd2	tumor-associated calcium signal transducer 2	6.26
ENSMUSG00000041523	Upk2	uroplakin 2	5.85
ENSMUSG00000032473	Cldn18	claudin 18	5.76
ENSMUSG00000074625	Arhgap40	Rho GTPase activating protein 40	5.61
ENSMUSG00000049001	Ndnf	neuron-derived neurotrophic factor	5.46
ENSMUSG00000059900	Tmem40	transmembrane protein 40	5.40
ENSMUSG00000039005	Tlr4	toll-like receptor 4	5.32
ENSMUSG00000021950	Anxa8	annexin A8	4.98
ENSMUSG00000059668	Krt4	keratin 4	4.76
ENSMUSG00000024659	Anxa1	annexin A1	4.59
ENSMUSG00000022037	Clu	clusterin	4.57
ENSMUSG00000033377	Palmd	palmdelphin	4.44
ENSMUSG00000091243	Vgll3	vestigial like family member 3	4.43
ENSMUSG00000044912	Syt16	synaptotagmin XVI	4.37
ENSMUSG00000031841	Cdh13	cadherin 13	4.27
ENSMUSG00000092586	Ly6g6c	lymphocyte antigen 6 complex, locus G6C	3.91
ENSMUSG00000051076	Vtcn1	V-set domain containing T cell activation inhibitor 1	3.81
ENSMUSG00000034463	Scara3	scavenger receptor class A, member 3	3.71
ENSMUSG00000029648	Flt1	FMS-like tyrosine kinase 1	3.69
ENSMUSG00000021822	Plau	plasminogen activator, urokinase	3.66
ENSMUSG00000021765	Fst	follistatin	3.64
ENSMUSG00000048376	F2r	coagulation factor II (thrombin) receptor	3.59
ENSMUSG00000048915	Efna5	ephrin A5	3.58
ENSMUSG00000037188	Grhl3	grainyhead like transcription factor 3	3.54
ENSMUSG00000040998	Npnt	nephronectin	3.50
ENSMUSG00000046213	Cym	chymosin	3.49
ENSMUSG00000026065	Slc9a4	solute carrier family 9 (sodium/hydrogen exchanger), member 4	3.37
ENSMUSG00000032327	Stra6	stimulated by retinoic acid gene 6	3.35
ENSMUSG00000027376	Prom2	prominin 2	3.33
ENSMUSG00000015134	Aldh1a3	aldehyde dehydrogenase family 1, subfamily A3	3.30
ENSMUSG00000034573	Ptpn13	protein tyrosine phosphatase, non-receptor type 13	3.29
ENSMUSG00000037946	Fgd3	FYVE, RhoGEF and PH domain containing 3	3.28
ENSMUSG00000043088	Il17re	interleukin 17 receptor E	3.23
ENSMUSG00000061517	Sox21	SRY (sex determining region Y)-box 21	3.20
ENSMUSG00000044162	Tnip3	TNFAIP3 interacting protein 3	3.19
ENSMUSG00000037833	Sh2d4b	SH2 domain containing 4B	3.16
ENSMUSG00000056632	Dsg3	desmoglein 3	3.00
ENSMUSG00000004552	Ctse	cathepsin E	2.95
ENSMUSG00000039153	Runx2	runt related transcription factor 2	2.93
ENSMUSG00000055407	Map6	microtubule-associated protein 6	2.93
ENSMUSG00000028236	Sdr16c5	short chain dehydrogenase/reductase family 16C, member 5	2.88
ENSMUSG00000032068	Plet1	placenta expressed transcript 1	2.88
ENSMUSG00000029154	Cwh43	cell wall biogenesis 43 C-terminal homolog	2.80
ENSMUSG00000056888	Glipr1	GLI pathogenesis-related 1 (glioma)	2.78
ENSMUSG00000013338	Fer1l4	fer-1-like 4 (C. elegans)	2.77
ENSMUSG00000063011	Msln	mesothelin	2.77
ENSMUSG00000031430	Vsig1	V-set and immunoglobulin domain containing 1	2.76
ENSMUSG00000055333	Fat2	FAT atypical cadherin 2	2.75
ENSMUSG00000045763	Basp1	brain abundant, membrane attached signal protein 1	2.74
ENSMUSG00000026620	Mark1	MAP/microtubule affinity regulating kinase 1	2.73
ENSMUSG00000021792	Fam213a	family with sequence similarity 213, member A	2.72
ENSMUSG00000001288	Rarg	retinoic acid receptor, gamma	2.72
ENSMUSG00000030510	Cers3	ceramide synthase 3	2.71
ENSMUSG00000031075	Ano1	anoctamin 1, calcium activated chloride channel	2.70
ENSMUSG00000050640	Tmem150c	transmembrane protein 150C	2.70
ENSMUSG00000050359	Sprr1a	small proline-rich protein 1A	2.70
ENSMUSG00000040663	Clcf1	cardiotrophin-like cytokine factor 1	2.68
ENSMUSG00000029484	Anxa3	annexin A3	2.67
ENSMUSG00000040990	Sh3kbp1	SH3-domain kinase binding protein 1	2.67

ENSMUSG00000029869	Ephb6	Eph receptor B6	2.67
ENSMUSG00000048612	Myof	myoferlin	2.66
ENSMUSG00000072812	Ahnak2	AHNAK nucleoprotein 2	2.63
ENSMUSG00000021838	Samd4	sterile alpha motif domain containing 4	2.60
ENSMUSG00000046402	Rbp1	retinol binding protein 1, cellular	2.60
ENSMUSG00000009687	Fxyd5	FXYD domain-containing ion transport regulator 5	2.59
ENSMUSG00000033227	Wnt6	wingless-type MMTV integration site family, member 6	2.59
ENSMUSG00000040488	Ltbp4	latent transforming growth factor beta binding protein 4	2.58
ENSMUSG00000022754	Tmem45a	transmembrane protein 45a	2.56
ENSMUSG00000032348	Gsta4	glutathione S-transferase, alpha 4	2.55
ENSMUSG00000046449	Nexmif	neurite extension and migration factor	2.50
ENSMUSG00000046623	Gjb4	gap junction protein, beta 4	2.49
ENSMUSG00000063727	Tnfrsf11b	tumor necrosis factor receptor superfamily, member 11b (osteoprotegerin)	2.48
ENSMUSG00000025938	Slco5a1	solute carrier organic anion transporter family, member 5A1	2.46
ENSMUSG00000026193	Fn1	fibronectin 1	2.46
ENSMUSG00000025473	Adam8	a disintegrin and metallopeptidase domain 8	2.45
ENSMUSG00000026981	Il1rn	interleukin 1 receptor antagonist	2.44
ENSMUSG00000022203	Efs	embryonal Fyn-associated substrate	2.44
ENSMUSG00000062591	Tubb4a	tubulin, beta 4A class IVA	2.43
ENSMUSG00000028716	Pdzk1ip1	PDZK1 interacting protein 1	2.43
ENSMUSG00000031098	Syt8	synaptotagmin VIII	2.42
ENSMUSG00000108218	Olfr1372-ps1	olfactory receptor 1372, pseudogene 1	2.38
ENSMUSG00000026548	Slamf9	SLAM family member 9	2.36
ENSMUSG00000020651	Slc26a4	solute carrier family 26, member 4	2.35
ENSMUSG00000001435	Col18a1	collagen, type XVIII, alpha 1	2.34
ENSMUSG00000031635	Anxa10	annexin A10	2.32
ENSMUSG00000037060	Cavin3	caveolae associated 3	2.29
ENSMUSG00000008398	Elk3	ELK3, member of ETS oncogene family	2.27
ENSMUSG00000026405	C4bp	complement component 4 binding protein	2.24
ENSMUSG00000024087	Cyp1b1	cytochrome P450, family 1, subfamily b, polypeptide 1	2.21
ENSMUSG00000038587	Akap12	A kinase (PRKA) anchor protein (gravin) 12	2.21
ENSMUSG00000051343	Rab11fip5	RAB11 family interacting protein 5 (class I)	2.20
ENSMUSG00000018340	Anxa6	annexin A6	2.19
ENSMUSG00000034634	Ly6d	lymphocyte antigen 6 complex, locus D	2.18
ENSMUSG00000026509	Capn2	calpain 2	2.17
ENSMUSG00000037716	Ccdc33	coiled-coil domain containing 33	2.17
ENSMUSG00000029371	Cxcl5	chemokine (C-X-C motif) ligand 5	2.17
ENSMUSG00000039384	Dusp10	dual specificity phosphatase 10	2.15
ENSMUSG00000080316	Spaca6	sperm acrosome associated 6	2.15
ENSMUSG00000021367	Edn1	endothelin 1	2.13
ENSMUSG00000047501	Cldn4	claudin 4	2.13
ENSMUSG00000027712	Anxa5	annexin A5	2.12
ENSMUSG00000026435	Slc45a3	solute carrier family 45, member 3	2.12
ENSMUSG00000001025	S100a6	S100 calcium binding protein A6 (calcyclin)	2.11
ENSMUSG00000053007	Creb5	cAMP responsive element binding protein 5	2.11
ENSMUSG00000079330	Lemd1	LEM domain containing 1	2.11
ENSMUSG00000040152	Thbs1	thrombospondin 1	2.10
ENSMUSG00000004951	Hspb1	heat shock protein 1	2.09
ENSMUSG00000027624	Epb41l1	erythrocyte membrane protein band 4.1 like 1	2.08
ENSMUSG00000019997	Ctgf	connective tissue growth factor	2.07
ENSMUSG00000037185	Krt80	keratin 80	2.07
ENSMUSG00000053137	Mapk11	mitogen-activated protein kinase 11	2.06
ENSMUSG00000085412	Halr1	Hoxa adjacent long noncoding RNA 1	2.06
ENSMUSG00000007039	Ddah2	dimethylarginine dimethylaminohydrolase 2	2.05
ENSMUSG00000024421	Lama3	laminin, alpha 3	2.05
ENSMUSG00000028031	Dkk2	dickkopf WNT signaling pathway inhibitor 2	2.02
ENSMUSG00000031586	Rbpms	RNA binding protein gene with multiple splicing	2.02
ENSMUSG00000038843	Gcnt1	glucosaminyl (N-acetyl) transferase 1, core 2	2.02
ENSMUSG00000028128	F3	coagulation factor III	2.01
ENSMUSG00000043430	Psapl1	prosaposin-like 1	2.01
ENSMUSG00000022055	Nefl	neurofilament, light polypeptide	2.01
ENSMUSG00000042734	Ttc9	tetratricopeptide repeat domain 9	2.00
ENSMUSG00000067889	Sptbn2	spectrin beta, non-erythrocytic 2	1.98
ENSMUSG00000045027	Prss22	protease, serine 22	1.98

ENSMUSG00000038463	Olfml2b	olfactomedin-like 2B	1.98
ENSMUSG00000022505	Emp2	epithelial membrane protein 2	1.97
ENSMUSG00000032013	Trim29	tripartite motif-containing 29	1.96
ENSMUSG00000000983	Wfdc18	WAP four-disulfide core domain 18	1.95
ENSMUSG00000034220	Gpc1	glypican 1	1.95
ENSMUSG00000023073	Slc10a2	solute carrier family 10, member 2	1.95
ENSMUSG00000031538	Plat	plasminogen activator, tissue	1.94
ENSMUSG00000031871	Cdh5	cadherin 5	1.94
ENSMUSG00000018800	Abca5	ATP-binding cassette, sub-family A (ABC1), member 5	1.92
ENSMUSG00000024727	Trpm6	transient receptor potential cation channel, subfamily M, member 6	1.91
ENSMUSG00000040430	Pitpnc1	phosphatidylinositol transfer protein, cytoplasmic 1	1.91
ENSMUSG00000040836	Gpr161	G protein-coupled receptor 161	1.91
ENSMUSG00000042306	S100a14	S100 calcium binding protein A14	1.89
ENSMUSG00000038775	Vill	villin-like	1.89
ENSMUSG00000006411	Nectin4	nectin cell adhesion molecule 4	1.87
ENSMUSG00000028270	Gbp2	guanylate binding protein 2	1.86
ENSMUSG00000043461	Sptssb	serine palmitoyltransferase, small subunit B	1.85
ENSMUSG00000026167	Wnt10a	wingless-type MMTV integration site family, member 10A	1.85
ENSMUSG00000024501	Dpysl3	dihydropyrimidinase-like 3	1.84
ENSMUSG00000025921	Rdh10	retinol dehydrogenase 10 (all-trans)	1.83
ENSMUSG00000041886	Macc1	metastasis associated in colon cancer 1	1.83
ENSMUSG00000072235	Tuba1a	tubulin, alpha 1A	1.83
ENSMUSG00000039232	Stx11	syntaxin 11	1.83
ENSMUSG00000004044	Cavin1	caveolae associated 1	1.82
ENSMUSG00000028464	Tpm2	tropomyosin 2, beta	1.82
ENSMUSG00000026678	Rgs5	regulator of G-protein signaling 5	1.81
ENSMUSG00000047344	Lancl3	LanC lantibiotic synthetase component C-like 3 (bacterial)	1.80
ENSMUSG00000025321	Itgb8	integrin beta 8	1.80
ENSMUSG00000030259	Rassf8	Ras association (RalGDS/AF-6) domain family (N-terminal) member 8	1.80
ENSMUSG00000025330	Padi4	peptidyl arginine deiminase, type IV	1.79
ENSMUSG00000072620	Slfn2	schlafen 2	1.77
ENSMUSG00000020121	Srgap1	SLIT-ROBO Rho GTPase activating protein 1	1.76
ENSMUSG00000027858	Tspan2	tetraspanin 2	1.76
ENSMUSG00000044646	Zbtb7c	zinc finger and BTB domain containing 7C	1.75
ENSMUSG00000039934	Gsap	gamma-secretase activating protein	1.73
ENSMUSG00000009097	Tbx1	T-box 1	1.73
ENSMUSG00000021493	Pdlim7	PDZ and LIM domain 7	1.72
ENSMUSG00000024401	Tnf	tumor necrosis factor	1.72
ENSMUSG00000029761	Cald1	caldesmon 1	1.72
ENSMUSG00000027210	Meis2	Meis homeobox 2	1.71
ENSMUSG00000022178	Ajuba	ajuba LIM protein	1.70
ENSMUSG00000039457	Ppl	periplakin	1.70
ENSMUSG00000017723	Wfdc2	WAP four-disulfide core domain 2	1.70
ENSMUSG00000037613	Tnfrsf23	tumor necrosis factor receptor superfamily, member 23	1.69
ENSMUSG00000032186	Tmod2	tropomodulin 2	1.68
ENSMUSG00000031372	Trex2	three prime repair exonuclease 2	1.68
ENSMUSG00000021701	Plk2	polo like kinase 2	1.67
ENSMUSG00000025432	Avil	advillin	1.66
ENSMUSG00000046223	Plaur	plasminogen activator, urokinase receptor	1.66
ENSMUSG00000006403	Adamts4	a disintegrin-like and metallopeptidase (reprolysin type) with thrombospondin type 1	1.66
ENSMUSG00000115009	G930009F23Rik	RIKEN cDNA G930009F23 gene	1.66
ENSMUSG00000031328	Flna	filamin, alpha	1.65
ENSMUSG00000037995	Igsf9	immunoglobulin superfamily, member 9	1.65
ENSMUSG00000052105	Mtcl1	microtubule crosslinking factor 1	1.65
ENSMUSG00000006777	Krt23	keratin 23	1.63
ENSMUSG00000023008	Fmnl3	formin-like 3	1.63
ENSMUSG00000029762	Akr1b8	aldo-keto reductase family 1, member B8	1.63
ENSMUSG00000062661	Ncs1	neuronal calcium sensor 1	1.63
ENSMUSG00000051043	Gprc5c	G protein-coupled receptor, family C, group 5, member C	1.63
ENSMUSG00000090877	Hspa1b	heat shock protein 1B	1.63
ENSMUSG00000026604	Ptpn14	protein tyrosine phosphatase, non-receptor type 14	1.63
ENSMUSG00000018920	Cxcl16	chemokine (C-X-C motif) ligand 16	1.62
ENSMUSG00000020668	Kif3c	kinesin family member 3C	1.62
ENSMUSG00000027737	Slc7a11	solute carrier family 7 (cationic amino acid transporter, y+ system), member 11	1.61

ENSMUSG00000116919	AC165151.1	conserved helix-loop-helix ubiquitous kinase (Chuk) pseudogene	1.60
ENSMUSG00000002980	Bcam	basal cell adhesion molecule	1.59
ENSMUSG00000023885	Thbs2	thrombospondin 2	1.59
ENSMUSG00000048960	Prex2	phosphatidylinositol-3,4,5-trisphosphate-dependent Rac exchange factor 2	1.58
ENSMUSG00000073599	Ecscr	endothelial cell surface expressed chemotaxis and apoptosis regulator	1.57
ENSMUSG00000020427	Igfbp3	insulin-like growth factor binding protein 3	1.57
ENSMUSG00000079018	Ly6c1	lymphocyte antigen 6 complex, locus C1	1.57
ENSMUSG00000032839	Trpc1	transient receptor potential cation channel, subfamily C, member 1	1.56
ENSMUSG00000038415	Foxq1	forkhead box Q1	1.54
ENSMUSG00000026479	Lamc2	laminin, gamma 2	1.54
ENSMUSG00000046589	Lrrc8e	leucine rich repeat containing 8 family, member E	1.54
ENSMUSG00000026639	Lamb3	laminin, beta 3	1.53
ENSMUSG00000027978	Prss12	protease, serine 12 neurotrypsin (motopsin)	1.53
ENSMUSG00000025931	Paqr8	progestin and adipoQ receptor family member VIII	1.53
ENSMUSG00000103738	Gm37652	predicted gene, 37652	1.53
ENSMUSG00000017897	Eya2	EYA transcriptional coactivator and phosphatase 2	1.52
ENSMUSG00000048078	Tenm4	teneurin transmembrane protein 4	1.51
ENSMUSG00000067006	Serpinb5	serine (or cysteine) peptidase inhibitor, clade B, member 5	1.50
ENSMUSG00000032496	Ltf	lactotransferrin	1.50
ENSMUSG00000074364	Ehd2	EH-domain containing 2	1.48
ENSMUSG00000075602	Ly6a	lymphocyte antigen 6 complex, locus A	1.47
ENSMUSG00000035678	Tnfsf9	tumor necrosis factor (ligand) superfamily, member 9	1.47
ENSMUSG00000021559	Dapk1	death associated protein kinase 1	1.46
ENSMUSG00000022371	Col14a1	collagen, type XIV, alpha 1	1.46
ENSMUSG00000035105	Egln3	egl-9 family hypoxia-inducible factor 3	1.45
ENSMUSG00000040543	Pitpnm3	PITPNM family member 3	1.45
ENSMUSG00000027907	S100a11	S100 calcium binding protein A11	1.44
ENSMUSG00000090394	4930523C07Rik	RIKEN cDNA 4930523C07 gene	1.43
ENSMUSG00000041608	Entpd3	ectonucleoside triphosphate diphosphohydrolase 3	1.43
ENSMUSG00000004891	Nes	nestin	1.43
ENSMUSG00000003541	Ier3	immediate early response 3	1.41
ENSMUSG00000026655	Fam107b	family with sequence similarity 107, member B	1.40
ENSMUSG00000061751	Kalrn	kalirin, RhoGEF kinase	1.40
ENSMUSG00000041570	Camsap2	calmodulin regulated spectrin-associated protein family, member 2	1.40
ENSMUSG00000053617	Sh3pxd2a	SH3 and PX domains 2A	1.39
ENSMUSG00000031790	Mmp15	matrix metallopeptidase 15	1.38
ENSMUSG00000040624	Plekhg1	pleckstrin homology domain containing, family G (with RhoGef domain) member 1	1.38
ENSMUSG00000030759	Far1	fatty acyl CoA reductase 1	1.38
ENSMUSG00000020773	Trim47	tripartite motif-containing 47	1.38
ENSMUSG00000032875	Arhgef17	Rho guanine nucleotide exchange factor (GEF) 17	1.36
ENSMUSG00000062488	Ifit3b	interferon-induced protein with tetratricopeptide repeats 3B	1.36
ENSMUSG00000032717	Mdfi	MyoD family inhibitor	1.35
ENSMUSG00000028073	Pear1	platelet endothelial aggregation receptor 1	1.35
ENSMUSG00000025372	Baiap2	brain-specific angiogenesis inhibitor 1-associated protein 2	1.34
ENSMUSG00000021477	Ctsl	cathepsin L	1.34
ENSMUSG00000034595	Ppp1r18	protein phosphatase 1, regulatory subunit 18	1.34
ENSMUSG00000023039	Krt7	keratin 7	1.33
ENSMUSG00000039831	Arhgap29	Rho GTPase activating protein 29	1.33
ENSMUSG00000034825	Nrip3	nuclear receptor interacting protein 3	1.33
ENSMUSG00000035697	Arhgap45	Rho GTPase activating protein 45	1.33
ENSMUSG00000029417	Cxcl9	chemokine (C-X-C motif) ligand 9	1.33
ENSMUSG00000020230	Prmt2	protein arginine N-methyltransferase 2	1.32
ENSMUSG00000036832	Lpar3	lysophosphatidic acid receptor 3	1.32
ENSMUSG00000026335	Pam	peptidylglycine alpha-amidating monooxygenase	1.31
ENSMUSG00000089809	Rasgef1b	RasGEF domain family, member 1B	1.31
ENSMUSG00000021281	Tnfaip2	tumor necrosis factor, alpha-induced protein 2	1.31
ENSMUSG00000051951	Xkr4	X-linked Kx blood group related 4	1.30
ENSMUSG00000017765	Slc12a4	solute carrier family 12, member 4	1.29
ENSMUSG00000031925	Maml2	mastermind like transcriptional coactivator 2	1.29
ENSMUSG00000079339	Ifit1bl1	interferon induced protein with tetratricpeptide repeats 1B like 1	1.29
ENSMUSG00000102460	Gm38197	predicted gene, 38197	1.29
ENSMUSG00000082361	Btc	betacellulin, epidermal growth factor family member	1.28
ENSMUSG00000024810	Il33	interleukin 33	1.28
ENSMUSG00000039364	Sectm1b	secreted and transmembrane 1B	1.27

ENSMUSG00000048058	Ldlrad3	low density lipoprotein receptor class A domain containing 3	1.27
ENSMUSG00000035818	Plekhs1	pleckstrin homology domain containing, family S member 1	1.27
ENSMUSG00000000489	Pdgfb	platelet derived growth factor, B polypeptide	1.27
ENSMUSG00000020181	Nav3	neuron navigator 3	1.27
ENSMUSG00000013076	Amotl1	angiomotin-like 1	1.26
ENSMUSG00000025287	Acot9	acyl-CoA thioesterase 9	1.26
ENSMUSG00000040711	Sh3pxd2b	SH3 and PX domains 2B	1.26
ENSMUSG00000028378	Ptgr1	prostaglandin reductase 1	1.25
ENSMUSG00000074796	Slc4a11	solute carrier family 4, sodium bicarbonate transporter-like, member 11	1.25
ENSMUSG00000041390	Mdfic	MyoD family inhibitor domain containing	1.25
ENSMUSG00000032724	Abtb2	ankyrin repeat and BTB (POZ) domain containing 2	1.24
ENSMUSG00000046694	Tent5b	terminal nucleotidyltransferase 5B	1.24
ENSMUSG00000110218	Gm20219	predicted gene, 20219	1.23
ENSMUSG00000022176	Rem2	rad and gem related GTP binding protein 2	1.23
ENSMUSG00000030352	Tspan9	tetraspanin 9	1.23
ENSMUSG00000021108	Prkch	protein kinase C, eta	1.23
ENSMUSG00000025278	Flnb	filamin, beta	1.23
ENSMUSG00000000706	Btn1a1	butyrophilin, subfamily 1, member A1	1.23
ENSMUSG00000025161	Slc16a3	solute carrier family 16 (monocarboxylic acid transporters), member 3	1.22
ENSMUSG00000022636	Alcam	activated leukocyte cell adhesion molecule	1.21
ENSMUSG00000024912	Fosl1	fos-like antigen 1	1.21
ENSMUSG00000009628	Tex15	testis expressed gene 15	1.21
ENSMUSG00000025383	Il23a	interleukin 23, alpha subunit p19	1.21
ENSMUSG00000024302	Dtna	dystrobrevin alpha	1.21
ENSMUSG00000027381	Bcl2l11	BCL2-like 11 (apoptosis facilitator)	1.20
ENSMUSG00000027257	Pacsin3	protein kinase C and casein kinase substrate in neurons 3	1.20
ENSMUSG00000019647	Sema6a	sema domain, transmembrane domain (TM), and cytoplasmic domain, (semaphorin)	1.20
ENSMUSG00000028435	Aqp3	aquaporin 3	1.20
ENSMUSG00000046731	Kctd11	potassium channel tetramerisation domain containing 11	1.19
ENSMUSG00000022665	Ccdc80	coiled-coil domain containing 80	1.19
ENSMUSG00000075033	Nxpe3	neurexophilin and PC-esterase domain family, member 3	1.18
ENSMUSG00000047735	Samd9l	sterile alpha motif domain containing 9-like	1.18
ENSMUSG00000060923	Acyp2	acylphosphatase 2, muscle type	1.18
ENSMUSG00000021360	Gcnt2	glucosaminyl (N-acetyl) transferase 2, I-branching enzyme	1.17
ENSMUSG00000041189	Chrnb1	cholinergic receptor, nicotinic, beta polypeptide 1 (muscle)	1.17
ENSMUSG00000019853	Hebp2	heme binding protein 2	1.16
ENSMUSG00000022865	Cxadr	coxsackie virus and adenovirus receptor	1.16
ENSMUSG00000038387	Rras	related RAS viral (r-ras) oncogene	1.16
ENSMUSG00000042510	AA986860	expressed sequence AA986860	1.16
ENSMUSG00000032643	Fhl3	four and a half LIM domains 3	1.16
ENSMUSG00000069255	Dusp22	dual specificity phosphatase 22	1.16
ENSMUSG00000037860	Aim2	absent in melanoma 2	1.14
ENSMUSG00000029377	Ereg	epiregulin	1.14
ENSMUSG00000018907	Alox12e	arachidonate lipoxygenase, epidermal	1.14
ENSMUSG00000029822	Osbpl3	oxysterol binding protein-like 3	1.13
ENSMUSG00000034459	Ifit1	interferon-induced protein with tetratricopeptide repeats 1	1.13
ENSMUSG00000042834	Nrep	neuronal regeneration related protein	1.13
ENSMUSG00000049807	Arhgap23	Rho GTPase activating protein 23	1.12
ENSMUSG00000037606	Osbpl5	oxysterol binding protein-like 5	1.12
ENSMUSG00000024940	Ltbp3	latent transforming growth factor beta binding protein 3	1.12
ENSMUSG00000069601	Ank3	ankyrin 3, epithelial	1.11
ENSMUSG00000031844	Hsd17b2	hydroxysteroid (17-beta) dehydrogenase 2	1.11
ENSMUSG00000037419	Endod1	endonuclease domain containing 1	1.10
ENSMUSG00000001473	Tubb6	tubulin, beta 6 class V	1.10
ENSMUSG00000055976	Cldn23	claudin 23	1.10
ENSMUSG00000034295	Fhod3	formin homology 2 domain containing 3	1.10
ENSMUSG00000020023	Tmcc3	transmembrane and coiled coil domains 3	1.09
ENSMUSG00000006435	Neurl1a	neuralized E3 ubiquitin protein ligase 1A	1.09
ENSMUSG00000029832	Nfe2l3	nuclear factor, erythroid derived 2, like 3	1.08
ENSMUSG00000027456	Sdcbp2	syndecan binding protein (syntenin) 2	1.08
ENSMUSG00000020674	Pxdn	peroxidasin	1.08
ENSMUSG00000027860	Vangl1	VANGL planar cell polarity 1	1.07
ENSMUSG00000035547	Capn5	calpain 5	1.07
ENSMUSG00000029378	Areg	amphiregulin	1.07

ENSMUSG00000038295	Atg9b	autophagy related 9B	1.06
ENSMUSG00000028631	Kcnq4	potassium voltage-gated channel, subfamily Q, member 4	1.06
ENSMUSG00000114996	Gm48958	predicted gene, 48958	1.06
ENSMUSG00000026797	Stxbp1	syntaxin binding protein 1	1.05
ENSMUSG00000031441	Atp11a	ATPase, class VI, type 11A	1.05
ENSMUSG00000053702	Nebl	nebulette	1.05
ENSMUSG00000068566	Myadm	myeloid-associated differentiation marker	1.04
ENSMUSG00000036528	Ppfibp2	PTPRF interacting protein, binding protein 2 (liprin beta 2)	1.04
ENSMUSG00000024777	Ppp2r5b	protein phosphatase 2, regulatory subunit B', beta	1.04
ENSMUSG00000038400	Pmepa1	prostate transmembrane protein, androgen induced 1	1.04
ENSMUSG00000049858	Suox	sulfite oxidase	1.04
ENSMUSG00000031367	Ap1s2	adaptor-related protein complex 1, sigma 2 subunit	1.04
ENSMUSG00000104415	Gm37069	predicted gene, 37069	1.04
ENSMUSG00000030342	Cd9	CD9 antigen	1.03
ENSMUSG00000048911	Rnf24	ring finger protein 24	1.03
ENSMUSG00000026961	Lrrc26	leucine rich repeat containing 26	1.03
ENSMUSG00000074006	Omp	olfactory marker protein	1.03
ENSMUSG00000027602	Map1lc3a	microtubule-associated protein 1 light chain 3 alpha	1.02
ENSMUSG00000040447	Spns2	spinster homolog 2	1.02
ENSMUSG00000069114	Zbtb10	zinc finger and BTB domain containing 10	1.02
ENSMUSG00000026478	Lamc1	laminin, gamma 1	1.01
ENSMUSG00000026796	Fam129b	family with sequence similarity 129, member B	1.01
ENSMUSG00000049866	Arl4c	ADP-ribosylation factor-like 4C	1.01
ENSMUSG00000057193	Slc44a2	solute carrier family 44, member 2	1.01
ENSMUSG00000038235	F11r	F11 receptor	1.01
ENSMUSG00000112198	Gm4065	predicted gene 4065	1.01
ENSMUSG00000026971	Itgb6	integrin beta 6	1.00
ENSMUSG00000079547	H2-DMb1	histocompatibility 2, class II, locus Mb1	-1.00
ENSMUSG00000057604	Lmcd1	LIM and cysteine-rich domains 1	-1.00
ENSMUSG00000102380	Gm38140	predicted gene, 38140	-1.00
ENSMUSG00000027452	Acss1	acyl-CoA synthetase short-chain family member 1	-1.01
ENSMUSG00000028415	Spink4	serine peptidase inhibitor, Kazal type 4	-1.01
ENSMUSG00000038255	Neurod2	neurogenic differentiation 2	-1.01
ENSMUSG00000025738	Fbxl16	F-box and leucine-rich repeat protein 16	-1.02
ENSMUSG00000031453	Rasa3	RAS p21 protein activator 3	-1.03
ENSMUSG00000074653	Lrrc31	leucine rich repeat containing 31	-1.03
ENSMUSG00000095328	Defa-ps6	defensin, alpha, pseudogene 6	-1.03
ENSMUSG00000055197	Fev	FEV (ETS oncogene family)	-1.03
ENSMUSG00000060586	H2-Eb1	histocompatibility 2, class II antigen E beta	-1.04
ENSMUSG00000031891	Hsd11b2	hydroxysteroid 11-beta dehydrogenase 2	-1.04
ENSMUSG00000051079	Rgs13	regulator of G-protein signaling 13	-1.04
ENSMUSG00000036006	Ripor2	RHO family interacting cell polarization regulator 2	-1.05
ENSMUSG00000024975	Pdcd4	programmed cell death 4	-1.06
ENSMUSG00000046613	Vwa5b2	von Willebrand factor A domain containing 5B2	-1.06
ENSMUSG00000003545	Fosb	FBJ osteosarcoma oncogene B	-1.07
ENSMUSG00000063895	Nupl1	nucleoporin like 1	-1.07
ENSMUSG00000063142	Kcnma1	potassium large conductance calcium-activated channel, subfamily M, alpha membe	-1.08
ENSMUSG00000021830	Txndc16	thioredoxin domain containing 16	-1.09
ENSMUSG00000108961	Gm32540	predicted gene, 32540	-1.09
ENSMUSG00000030934	Oat	ornithine aminotransferase	-1.10
ENSMUSG00000022816	Fstl1	follistatin-like 1	-1.11
ENSMUSG00000097924	A730020E08Rik	RIKEN cDNA A730020E08 gene	-1.11
ENSMUSG00000043487	Acot6	acyl-CoA thioesterase 6	-1.12
ENSMUSG00000026610	Esrrg	estrogen-related receptor gamma	-1.13
ENSMUSG00000078586	Gm11735	predicted gene 11735	-1.13
ENSMUSG00000033705	Stard9	START domain containing 9	-1.14
ENSMUSG00000009378	Slc16a12	solute carrier family 16 (monocarboxylic acid transporters), member 12	-1.15
ENSMUSG00000033715	Akr1c14	aldo-keto reductase family 1, member C14	-1.15
ENSMUSG00000038745	Nlrp6	NLR family, pyrin domain containing 6	-1.16
ENSMUSG00000031451	Gas6	growth arrest specific 6	-1.16
ENSMUSG00000052026	Slc6a7	solute carrier family 6 (neurotransmitter transporter, L-proline), member 7	-1.16
ENSMUSG00000058740	Kcnt1	potassium channel, subfamily T, member 1	-1.16
ENSMUSG00000097494	4933406C10Rik	RIKEN cDNA 4933406C10 gene	-1.17
ENSMUSG00000028838	Extl1	exostoses (multiple)-like 1	-1.18

ENSMUSG00000024225	Clps	colipase, pancreatic	-1.18
ENSMUSG00000074715	Ccl28	chemokine (C-C motif) ligand 28	-1.19
ENSMUSG00000060208	Defa17	defensin, alpha, 17	-1.19
ENSMUSG00000026398	Nr5a2	nuclear receptor subfamily 5, group A, member 2	-1.21
ENSMUSG00000002346	Slc25a42	solute carrier family 25, member 42	-1.21
ENSMUSG00000040412	5330417C22Rik	RIKEN cDNA 5330417C22 gene	-1.25
ENSMUSG00000056553	Ptprn2	protein tyrosine phosphatase, receptor type, N polypeptide 2	-1.25
ENSMUSG00000109936	Gm45889	predicted gene 45889	-1.25
ENSMUSG00000033740	St18	suppression of tumorigenicity 18	-1.25
ENSMUSG00000110170	St6galnac2	ST6 (alpha-N-acetyl-neuraminyl-2,3-beta-galactosyl-1,3)-N-acetylgalactosaminide alp	-1.25
ENSMUSG00000000416	Cttnbp2	cortactin binding protein 2	-1.26
ENSMUSG00000026442	Nfasc	neurofascin	-1.26
ENSMUSG00000005089	Slc1a2	solute carrier family 1 (glial high affinity glutamate transporter), member 2	-1.26
ENSMUSG00000110622	Iqcn	IQ motif containing N	-1.26
ENSMUSG00000079180	Mptx2	mucosal pentraxin 2	-1.27
ENSMUSG00000043648	Pld6	phospholipase D family, member 6	-1.27
ENSMUSG00000045709	Smkr-ps	smal lysine rich protein 1, pseudogene	-1.28
ENSMUSG00000031173	Otc	ornithine transcarbamylase	-1.29
ENSMUSG00000106944	Gm43843	predicted gene 43843	-1.29
ENSMUSG00000040706	Agmat	agmatine ureohydrolase (agmatinase)	-1.30
ENSMUSG00000028327	Stra6l	STRA6-like	-1.30
ENSMUSG00000048027	Rgmb	repulsive guidance molecule family member B	-1.31
ENSMUSG00000022018	Rgcc	regulator of cell cycle	-1.32
ENSMUSG00000025790	Slco3a1	solute carrier organic anion transporter family, member 3a1	-1.32
ENSMUSG00000033066	Gas7	growth arrest specific 7	-1.32
ENSMUSG00000023886	Smoc2	SPARC related modular calcium binding 2	-1.33
ENSMUSG00000025196	Cpn1	carboxypeptidase N, polypeptide 1	-1.33
ENSMUSG00000022650	Retnlb	resistin like beta	-1.33
ENSMUSG00000058618	AY761184	cDNA sequence AY761184	-1.33
ENSMUSG00000009394	Syn2	synapsin II	-1.33
ENSMUSG00000025082	Vwa2	von Willebrand factor A domain containing 2	-1.34
ENSMUSG00000033721	Vav3	vav 3 oncogene	-1.34
ENSMUSG00000004655	Aqp1	aquaporin 1	-1.36
ENSMUSG00000025867	Cplx2	complexin 2	-1.36
ENSMUSG00000052271	Bhlha15	basic helix-loop-helix family, member a15	-1.38
ENSMUSG00000079559	Colca2	COLCA2 homolog	-1.38
ENSMUSG00000005268	Prlr	prolactin receptor	-1.39
ENSMUSG00000074437	Defa29	defensin, alpha, 29	-1.39
ENSMUSG00000090105	Gm15890	predicted gene 15890	-1.39
ENSMUSG00000070337	Gpr179	G protein-coupled receptor 179	-1.40
ENSMUSG00000032452	Clstn2	calsyntenin 2	-1.40
ENSMUSG00000038058	Nod1	nucleotide-binding oligomerization domain containing 1	-1.43
ENSMUSG00000022504	Ciita	class II transactivator	-1.43
ENSMUSG00000032561	Acpp	acid phosphatase, prostate	-1.44
ENSMUSG00000041193	Pla2g5	phospholipase A2, group V	-1.45
ENSMUSG00000010021	Kif19a	kinesin family member 19A	-1.45
ENSMUSG00000084939	Gm830	predicted gene 830	-1.46
ENSMUSG00000044694	2010007H06Rik	RIKEN cDNA 2010007H06 gene	-1.47
ENSMUSG00000005360	Slc1a3	solute carrier family 1 (glial high affinity glutamate transporter), member 3	-1.48
ENSMUSG00000019900	Rfx6	regulatory factor X, 6	-1.51
ENSMUSG00000020140	Lgr5	leucine rich repeat containing G protein coupled receptor 5	-1.52
ENSMUSG00000033717	Adra2a	adrenergic receptor, alpha 2a	-1.53
ENSMUSG00000026786	Apbb1ip	amyloid beta (A4) precursor protein-binding, family B, member 1 interacting protein	-1.56
ENSMUSG00000112816	Gm48798	predicted gene, 48798	-1.56
ENSMUSG00000025991	Cps1	carbamoyl-phosphate synthetase 1	-1.60
ENSMUSG00000028602	Tnfrsf8	tumor necrosis factor receptor superfamily, member 8	-1.60
ENSMUSG00000040528	Milr1	mast cell immunoglobulin like receptor 1	-1.60
ENSMUSG00000035561	Aldh1b1	aldehyde dehydrogenase 1 family, member B1	-1.61
ENSMUSG00000026489	Coq8a	coenzyme Q8A	-1.61
ENSMUSG00000073424	Cyp4f15	cytochrome P450, family 4, subfamily f, polypeptide 15	-1.61
ENSMUSG00000086513	9130208D14Rik	RIKEN cDNA 9130208D14 gene	-1.65
ENSMUSG00000027359	Slc27a2	solute carrier family 27 (fatty acid transporter), member 2	-1.66
ENSMUSG00000031428	Zcchc18	zinc finger, CCHC domain containing 18	-1.67
ENSMUSG00000021719	Rgs7bp	regulator of G-protein signalling 7 binding protein	-1.68

ENSMUSG00000095649	Gm8979	predicted gene 8979	-1.75
ENSMUSG00000029445	Hpd	4-hydroxyphenylpyruvic acid dioxygenase	-1.76
ENSMUSG00000030207	Fam234b	family with sequence similarity 234, member B	-1.77
ENSMUSG00000018341	Il12rb2	interleukin 12 receptor, beta 2	-1.80
ENSMUSG00000000031	H19	H19, imprinted maternally expressed transcript	-1.81
ENSMUSG00000037953	A4gnt	alpha-1,4-N-acetylglucosaminyltransferase	-1.81
ENSMUSG00000109685	Gm45912	predicted gene 45912	-1.85
ENSMUSG00000023236	Scg5	secretogranin V	-1.86
ENSMUSG00000117254	AC171111.1	novel transcript	-1.87
ENSMUSG00000066687	Zbtb16	zinc finger and BTB domain containing 16	-1.89
ENSMUSG00000036585	Fgf1	fibroblast growth factor 1	-1.95
ENSMUSG00000036526	Card11	caspase recruitment domain family, member 11	-1.99
ENSMUSG00000024411	Aqp4	aquaporin 4	-2.00
ENSMUSG00000023828	Slc22a3	solute carrier family 22 (organic cation transporter), member 3	-2.08
ENSMUSG00000041577	Prelp	proline arginine-rich end leucine-rich repeat	-2.09
ENSMUSG00000034472	Rasd2	RASD family, member 2	-2.09
ENSMUSG00000000402	Egfl6	EGF-like-domain, multiple 6	-2.31
ENSMUSG00000110266	Gm32742	predicted gene, 32742	-2.31
ENSMUSG00000036422	Pcdh8	protocadherin 8	-2.60
ENSMUSG00000022026	Olfm4	olfactomedin 4	-2.76
ENSMUSG00000038765	Lmx1b	LIM homeobox transcription factor 1 beta	-2.92

RFP+Trop2+ vs. differentiated cells

Ensemble gene code	Symbol	Gene name	logFC
ENSMUSG00000029304	Spp1	secreted phosphoprotein 1	5.80
ENSMUSG00000051397	Tacstd2	tumor-associated calcium signal transducer 2	5.16
ENSMUSG00000041523	Upk2	uroplakin 2	4.78
ENSMUSG00000022754	Tmem45a	transmembrane protein 45a	4.67
ENSMUSG00000059668	Krt4	keratin 4	4.65
ENSMUSG00000037060	Cavin3	caveolae associated 3	4.58
ENSMUSG00000024087	Cyp1b1	cytochrome P450, family 1, subfamily b, polypeptide 1	4.51
ENSMUSG00000021950	Anxa8	annexin A8	4.23
ENSMUSG00000024659	Anxa1	annexin A1	4.20
ENSMUSG00000029648	Flt1	FMS-like tyrosine kinase 1	4.20
ENSMUSG00000031430	Vsig1	V-set and immunoglobulin domain containing 1	4.17
ENSMUSG00000033377	Palmd	palmdelphin	3.89
ENSMUSG00000022037	Clu	clusterin	3.82
ENSMUSG00000079330	Lemd1	LEM domain containing 1	3.76
ENSMUSG00000049128	Ivl	involucrin	3.75
ENSMUSG00000091243	Vgll3	vestigial like family member 3	3.68
ENSMUSG00000037124	Trim58	tripartite motif-containing 58	3.63
ENSMUSG00000061517	Sox21	SRY (sex determining region Y)-box 21	3.58
ENSMUSG00000032327	Stra6	stimulated by retinoic acid gene 6	3.57
ENSMUSG00000058354	Krt6a	keratin 6A	3.40
ENSMUSG00000037188	Grhl3	grainyhead like transcription factor 3	3.39
ENSMUSG00000009097	Tbx1	T-box 1	3.34
ENSMUSG00000039153	Runx2	runt related transcription factor 2	3.28
ENSMUSG00000056632	Dsg3	desmoglein 3	3.27
ENSMUSG00000027485	Bpifb1	BPI fold containing family B, member 1	3.23
ENSMUSG00000020773	Trim47	tripartite motif-containing 47	3.21
ENSMUSG00000030510	Cers3	ceramide synthase 3	3.19
ENSMUSG00000097519	4930558J18Rik	RIKEN cDNA 4930558J18 gene	3.17
ENSMUSG00000022203	Efs	embryonal Fyn-associated substrate	3.09
ENSMUSG00000051076	Vtcn1	V-set domain containing T cell activation inhibitor 1	2.93
ENSMUSG00000034463	Scara3	scavenger receptor class A, member 3	2.91
ENSMUSG00000063011	Msln	mesothelin	2.91
ENSMUSG00000048915	Efna5	ephrin A5	2.91
ENSMUSG00000041886	Macc1	metastasis associated in colon cancer 1	2.89
ENSMUSG00000025473	Adam8	a disintegrin and metallopeptidase domain 8	2.86
ENSMUSG00000022371	Col14a1	collagen, type XIV, alpha 1	2.85
ENSMUSG00000063060	Sox7	SRY (sex determining region Y)-box 7	2.85
ENSMUSG00000046623	Gjb4	gap junction protein, beta 4	2.80
ENSMUSG00000035818	Plekhs1	pleckstrin homology domain containing, family S member 1	2.79
ENSMUSG00000021838	Samd4	sterile alpha motif domain containing 4	2.78
ENSMUSG00000082706	Gm11663	predicted gene 11663	2.77
ENSMUSG00000074625	Arhgap40	Rho GTPase activating protein 40	2.76
ENSMUSG00000042734	Ttc9	tetratricopeptide repeat domain 9	2.75
ENSMUSG00000028236	Sdr16c5	short chain dehydrogenase/reductase family 16C, member 5	2.75
ENSMUSG00000031097	Tnni2	troponin I, skeletal, fast 2	2.75
ENSMUSG00000092586	Ly6g6c	lymphocyte antigen 6 complex, locus G6C	2.73
ENSMUSG00000113168	Gm7614	predicted gene 7614	2.70
ENSMUSG00000013338	Fer1l4	fer-1-like 4 (C. elegans)	2.67
ENSMUSG00000040488	Ltbp4	latent transforming growth factor beta binding protein 4	2.66
ENSMUSG00000040543	Pitpnm3	PITPNM family member 3	2.63
ENSMUSG00000046213	Cym	chymosin	2.60
ENSMUSG00000079018	Ly6c1	lymphocyte antigen 6 complex, locus C1	2.52
ENSMUSG00000085412	Halr1	Hoxa adjacent long noncoding RNA 1	2.51
ENSMUSG00000031871	Cdh5	cadherin 5	2.46
ENSMUSG00000031841	Cdh13	cadherin 13	2.42
ENSMUSG00000028464	Tpm2	tropomyosin 2, beta	2.41
ENSMUSG00000107350	Gm19610	predicted gene, 19610	2.41
ENSMUSG00000006411	Nectin4	nectin cell adhesion molecule 4	2.40
ENSMUSG00000037833	Sh2d4b	SH2 domain containing 4B	2.40
ENSMUSG00000021367	Edn1	endothelin 1	2.39
ENSMUSG00000015134	Aldh1a3	aldehyde dehydrogenase family 1, subfamily A3	2.39
ENSMUSG00000034573	Ptpn13	protein tyrosine phosphatase, non-receptor type 13	2.38
ENSMUSG00000114996	Gm48958	predicted gene, 48958	2.38

ENSMUSG00000041482	Piezo2	piezo-type mechanosensitive ion channel component 2	2.38
ENSMUSG00000029371	Cxcl5	chemokine (C-X-C motif) ligand 5	2.35
ENSMUSG00000026981	Il1rn	interleukin 1 receptor antagonist	2.33
ENSMUSG00000029484	Anxa3	annexin A3	2.29
ENSMUSG00000026065	Slc9a4	solute carrier family 9 (sodium/hydrogen exchanger), member 4	2.29
ENSMUSG00000025902	Sox17	SRY (sex determining region Y)-box 17	2.27
ENSMUSG00000030259	Rassf8	Ras association (RalGDS/AF-6) domain family (N-terminal) member 8	2.27
ENSMUSG00000055333	Fat2	FAT atypical cadherin 2	2.25
ENSMUSG00000046402	Rbp1	retinol binding protein 1, cellular	2.23
ENSMUSG00000026327	Serpinb11	serine (or cysteine) peptidase inhibitor, clade B (ovalbumin), member 11	2.23
ENSMUSG00000001288	Rarg	retinoic acid receptor, gamma	2.22
ENSMUSG00000037628	Cdkn3	cyclin-dependent kinase inhibitor 3	2.22
ENSMUSG00000000706	Btn1a1	butyrophilin, subfamily 1, member A1	2.22
ENSMUSG00000022178	Ajuba	ajuba LIM protein	2.20
ENSMUSG00000028068	Iqgap3	IQ motif containing GTPase activating protein 3	2.18
ENSMUSG00000082095	Gm11991	predicted gene 11991	2.18
ENSMUSG00000031098	Syt8	synaptotagmin VIII	2.17
ENSMUSG00000025321	Itgb8	integrin beta 8	2.17
ENSMUSG00000032254	Kif23	kinesin family member 23	2.16
ENSMUSG00000022505	Emp2	epithelial membrane protein 2	2.16
ENSMUSG00000013766	Ly6g6e	lymphocyte antigen 6 complex, locus G6E	2.16
ENSMUSG00000031538	Plat	plasminogen activator, tissue	2.15
ENSMUSG00000020427	Igfbp3	insulin-like growth factor binding protein 3	2.14
ENSMUSG00000094248	Hist1h2ao	histone cluster 1, H2ao	2.10
ENSMUSG00000031328	Flna	filamin, alpha	2.08
ENSMUSG00000027375	Mal	myelin and lymphocyte protein, T cell differentiation protein	2.08
ENSMUSG00000053007	Creb5	cAMP responsive element binding protein 5	2.08
ENSMUSG00000038415	Foxq1	forkhead box Q1	2.06
ENSMUSG00000040998	Npnt	nephronectin	2.06
ENSMUSG00000021176	Efcab11	EF-hand calcium binding domain 11	2.05
ENSMUSG00000051378	Kif18b	kinesin family member 18B	2.04
ENSMUSG00000075602	Ly6a	lymphocyte antigen 6 complex, locus A	2.04
ENSMUSG00000006398	Cdc20	cell division cycle 20	2.02
ENSMUSG00000056888	Glipr1	GLI pathogenesis-related 1 (glioma)	2.02
ENSMUSG00000021822	Plau	plasminogen activator, urokinase	2.02
ENSMUSG00000027861	Casq2	calsequestrin 2	2.02
ENSMUSG00000027469	Tpx2	TPX2, microtubule-associated	2.01
ENSMUSG00000063727	Tnfrsf11b	tumor necrosis factor receptor superfamily, member 11b (osteoprotegerin)	2.01
ENSMUSG00000044201	Cdc25c	cell division cycle 25C	2.01
ENSMUSG00000029380	Cxcl1	chemokine (C-X-C motif) ligand 1	2.01
ENSMUSG00000035365	Parpbp	PARP1 binding protein	2.00
ENSMUSG00000037946	Fgd3	FYVE, RhoGEF and PH domain containing 3	2.00
ENSMUSG00000027699	Ect2	ect2 oncogene	1.99
ENSMUSG00000020330	Hmmr	hyaluronan mediated motility receptor (RHAMM)	1.99
ENSMUSG00000079553	Kifc1	kinesin family member C1	1.99
ENSMUSG00000040711	Sh3pxd2b	SH3 and PX domains 2B	1.99
ENSMUSG00000101355	Hist1h3h	histone cluster 1, H3h	1.99
ENSMUSG00000032783	Troap	trophinin associated protein	1.98
ENSMUSG00000020808	Pimreg	PICALM interacting mitotic regulator	1.98
ENSMUSG00000017716	Birc5	baculoviral IAP repeat-containing 5	1.97
ENSMUSG00000041498	Kif14	kinesin family member 14	1.96
ENSMUSG00000003541	Ier3	immediate early response 3	1.95
ENSMUSG00000028678	Kif2c	kinesin family member 2C	1.95
ENSMUSG00000106296	4632404M16Rik	RIKEN cDNA 4632404M16 gene	1.95
ENSMUSG00000027715	Ccna2	cyclin A2	1.94
ENSMUSG00000059900	Tmem40	transmembrane protein 40	1.94
ENSMUSG00000024989	Cep55	centrosomal protein 55	1.93
ENSMUSG00000003779	Kif20a	kinesin family member 20A	1.93
ENSMUSG00000027379	Bub1	BUB1, mitotic checkpoint serine/threonine kinase	1.93
ENSMUSG00000024056	Ndc80	NDC80 kinetochore complex component	1.92
ENSMUSG00000027860	Vangl1	VANGL planar cell polarity 1	1.91
ENSMUSG00000032875	Arhgef17	Rho guanine nucleotide exchange factor (GEF) 17	1.91
ENSMUSG00000028175	Depdc1a	DEP domain containing 1a	1.91
ENSMUSG00000041431	Ccnb1	cyclin B1	1.90
ENSMUSG00000037544	Dlgap5	DLG associated protein 5	1.89
ENSMUSG00000036777	Anln	anillin, actin binding protein	1.89
ENSMUSG00000023348	Trip6	thyroid hormone receptor interactor 6	1.89

ENSMUSG00000072082	Ccnf	cyclin F	1.89
ENSMUSG00000004044	Cavin1	caveolae associated 1	1.89
ENSMUSG00000026622	Nek2	NIMA (never in mitosis gene a)-related expressed kinase 2	1.88
ENSMUSG00000033952	Aspm	abnormal spindle microtubule assembly	1.88
ENSMUSG00000023505	Cdca3	cell division cycle associated 3	1.87
ENSMUSG00000069310	Hist1h3c	histone cluster 1, H3c	1.86
ENSMUSG00000090394	4930523C07Rik	RIKEN cDNA 4930523C07 gene	1.86
ENSMUSG00000034311	Kif4	kinesin family member 4	1.85
ENSMUSG00000035683	Melk	maternal embryonic leucine zipper kinase	1.84
ENSMUSG00000041984	Rptn	repetin	1.84
ENSMUSG00000114456	Hist1h2bh	histone cluster 1, H2bh	1.83
ENSMUSG00000094338	Hist1h2bl	histone cluster 1, H2bl	1.83
ENSMUSG00000001435	Col18a1	collagen, type XVIII, alpha 1	1.82
ENSMUSG00000019832	Rab32	RAB32, member RAS oncogene family	1.82
ENSMUSG00000069267	Hist1h3b	histone cluster 1, H3b	1.81
ENSMUSG00000022322	Shcbp1	Shc SH2-domain binding protein 1	1.81
ENSMUSG00000001403	Ube2c	ubiquitin-conjugating enzyme E2C	1.81
ENSMUSG00000032218	Ccnb2	cyclin B2	1.80
ENSMUSG00000019942	Cdk1	cyclin-dependent kinase 1	1.80
ENSMUSG00000081740	Gm14279	predicted gene 14279	1.80
ENSMUSG00000023015	Racgap1	Rac GTPase-activating protein 1	1.79
ENSMUSG00000099583	Hist1h3d	histone cluster 1, H3d	1.79
ENSMUSG00000042306	S100a14	S100 calcium binding protein A14	1.79
ENSMUSG00000027737	Slc7a11	solute carrier family 7 (cationic amino acid transporter, y+ system), member 11	1.79
ENSMUSG00000024727	Trpm6	transient receptor potential cation channel, subfamily M, member 6	1.79
ENSMUSG00000026683	Nuf2	NUF2, NDC80 kinetochore complex component	1.78
ENSMUSG00000022096	Hr	hairless	1.77
ENSMUSG00000028873	Cdca8	cell division cycle associated 8	1.77
ENSMUSG00000021965	Ska3	spindle and kinetochore associated complex subunit 3	1.77
ENSMUSG00000021493	Pdlim7	PDZ and LIM domain 7	1.77
ENSMUSG00000020493	Prr11	proline rich 11	1.77
ENSMUSG00000048376	F2r	coagulation factor II (thrombin) receptor	1.77
ENSMUSG00000038943	Prc1	protein regulator of cytokinesis 1	1.76
ENSMUSG00000029177	Cenpa	centromere protein A	1.76
ENSMUSG00000031262	Cenpi	centromere protein I	1.76
ENSMUSG00000099517	Hist1h3g	histone cluster 1, H3g	1.75
ENSMUSG00000026605	Cenpf	centromere protein F	1.74
ENSMUSG00000098985	Gm27219	predicted gene 27219	1.74
ENSMUSG00000037313	Tacc3	transforming, acidic coiled-coil containing protein 3	1.73
ENSMUSG00000029910	Mad2l1	MAD2 mitotic arrest deficient-like 1	1.73
ENSMUSG00000022021	Diaph3	diaphanous related formin 3	1.73
ENSMUSG00000035783	Acta2	actin, alpha 2, smooth muscle, aorta	1.73
ENSMUSG00000045328	Cenpe	centromere protein E	1.72
ENSMUSG00000069910	Spdl1	spindle apparatus coiled-coil protein 1	1.72
ENSMUSG00000036111	Lmo1	LIM domain only 1	1.72
ENSMUSG00000048327	Ckap2l	cytoskeleton associated protein 2-like	1.71
ENSMUSG00000022034	Esco2	establishment of sister chromatid cohesion N-acetyltransferase 2	1.71
ENSMUSG00000027115	Kif18a	kinesin family member 18A	1.71
ENSMUSG00000041064	Pif1	PIF1 5'-to-3' DNA helicase	1.71
ENSMUSG00000067818	Myl9	myosin, light polypeptide 9, regulatory	1.71
ENSMUSG00000027496	Aurka	aurora kinase A	1.70
ENSMUSG00000114279	Hist1h2bm	histone cluster 1, H2bm	1.70
ENSMUSG00000114374	Gm7911	predicted gene 7911	1.70
ENSMUSG00000075031	Hist1h2bb	histone cluster 1, H2bb	1.69
ENSMUSG00000031257	Nox1	NADPH oxidase 1	1.69
ENSMUSG00000074364	Ehd2	EH-domain containing 2	1.69
ENSMUSG00000027331	Knstrn	kinetochore-localized astrin/SPAG5 binding	1.68
ENSMUSG00000023940	Sgo1	shugoshin 1	1.68
ENSMUSG00000062661	Ncs1	neuronal calcium sensor 1	1.68
ENSMUSG00000110644	Gm7390	predicted gene 7390	1.68
ENSMUSG00000017861	Mybl2	myeloblastosis oncogene-like 2	1.67
ENSMUSG00000024912	Fosl1	fos-like antigen 1	1.67
ENSMUSG00000004552	Ctse	cathepsin E	1.67
ENSMUSG00000097651	4930461G14Rik	RIKEN cDNA 4930461G14 gene	1.67
ENSMUSG00000026435	Slc45a3	solute carrier family 45, member 3	1.67
ENSMUSG00000028073	Pear1	platelet endothelial aggregation receptor 1	1.66
ENSMUSG00000033031	Cip2a	cell proliferation regulating inhibitor of protein phosphatase 2A	1.65

ENSMUSG00000030677	Kif22	kinesin family member 22	1.64
ENSMUSG00000014444	Piezo1	piezo-type mechanosensitive ion channel component 1	1.64
ENSMUSG00000069184	Zfp72	zinc finger protein 72	1.64
ENSMUSG00000029377	Ereg	epiregulin	1.64
ENSMUSG00000090877	Hspa1b	heat shock protein 1B	1.63
ENSMUSG00000027306	Nusap1	nucleolar and spindle associated protein 1	1.62
ENSMUSG00000037725	Ckap2	cytoskeleton associated protein 2	1.62
ENSMUSG00000021701	Plk2	polo like kinase 2	1.62
ENSMUSG00000024795	Kif20b	kinesin family member 20B	1.62
ENSMUSG00000021569	Trip13	thyroid hormone receptor interactor 13	1.62
ENSMUSG00000031075	Ano1	anoctamin 1, calcium activated chloride channel	1.62
ENSMUSG00000103445	Gm36948	predicted gene, 36948	1.62
ENSMUSG00000026970	Rbms1	RNA binding motif, single stranded interacting protein 1	1.61
ENSMUSG00000069300	Hist1h2bj	histone cluster 1, H2bj	1.61
ENSMUSG00000062727	Hist1h2bk	histone cluster 1, H2bk	1.61
ENSMUSG00000051439	Cd14	CD14 antigen	1.61
ENSMUSG00000067006	Serpinb5	serine (or cysteine) peptidase inhibitor, clade B, member 5	1.61
ENSMUSG00000037716	Ccdc33	coiled-coil domain containing 33	1.61
ENSMUSG00000032446	Eomes	eomesodermin	1.61
ENSMUSG00000020914	Top2a	topoisomerase (DNA) II alpha	1.60
ENSMUSG00000015880	Ncapg	non-SMC condensin I complex, subunit G	1.60
ENSMUSG00000062937	Mtap	methylthioadenosine phosphorylase	1.60
ENSMUSG00000012443	Kif11	kinesin family member 11	1.60
ENSMUSG00000032717	Mdfi	MyoD family inhibitor	1.60
ENSMUSG00000058773	Hist1h1b	histone cluster 1, H1b	1.59
ENSMUSG00000052565	Hist1h1d	histone cluster 1, H1d	1.59
ENSMUSG00000058290	Espl1	extra spindle pole bodies 1, separase	1.59
ENSMUSG00000020897	Aurkb	aurora kinase B	1.59
ENSMUSG00000040084	Bub1b	BUB1B, mitotic checkpoint serine/threonine kinase	1.59
ENSMUSG00000038295	Atg9b	autophagy related 9B	1.59
ENSMUSG00000040836	Gpr161	G protein-coupled receptor 161	1.59
ENSMUSG00000049866	Arl4c	ADP-ribosylation factor-like 4C	1.58
ENSMUSG00000025372	Baiap2	brain-specific angiogenesis inhibitor 1-associated protein 2	1.58
ENSMUSG00000022883	Robo1	roundabout guidance receptor 1	1.58
ENSMUSG00000037071	Scd1	stearoyl-Coenzyme A desaturase 1	1.58
ENSMUSG00000038252	Ncapd2	non-SMC condensin I complex, subunit D2	1.57
ENSMUSG00000001517	Foxm1	forkhead box M1	1.57
ENSMUSG00000059791	Nrm	nurim (nuclear envelope membrane protein)	1.57
ENSMUSG00000089762	Ier5l	immediate early response 5-like	1.57
ENSMUSG00000073599	Ecscr	endothelial cell surface expressed chemotaxis and apoptosis regulator	1.56
ENSMUSG00000078521	Aunip	aurora kinase A and ninein interacting protein	1.56
ENSMUSG00000082485	Gm15693	predicted gene 15693	1.56
ENSMUSG00000021604	Irx4	Iroquois homeobox 4	1.56
ENSMUSG00000026039	Sgo2a	shugoshin 2A	1.55
ENSMUSG00000069265	Hist1h3a	histone cluster 1, H3a	1.55
ENSMUSG00000004891	Nes	nestin	1.55
ENSMUSG00000023931	Efhb	EF hand domain family, member B	1.55
ENSMUSG00000104238	Gm37587	predicted gene, 37587	1.55
ENSMUSG00000022033	Pbk	PDZ binding kinase	1.54
ENSMUSG00000095217	Hist1h2bn	histone cluster 1, H2bn	1.54
ENSMUSG00000025921	Rdh10	retinol dehydrogenase 10 (all-trans)	1.54
ENSMUSG00000034773	BC030867	cDNA sequence BC030867	1.54
ENSMUSG00000032348	Gsta4	glutathione S-transferase, alpha 4	1.54
ENSMUSG00000043430	Psapl1	prosaposin-like 1	1.54
ENSMUSG00000027323	Rad51	RAD51 recombinase	1.53
ENSMUSG00000069274	Hist1h4f	histone cluster 1, H4f	1.53
ENSMUSG00000032221	Mns1	meiosis-specific nuclear structural protein 1	1.53
ENSMUSG00000097755	2010110K18Rik	RIKEN cDNA 2010110K18 gene	1.53
ENSMUSG00000025279	Dnase1l3	deoxyribonuclease 1-like 3	1.53
ENSMUSG00000058385	Hist1h2bg	histone cluster 1, H2bg	1.52
ENSMUSG00000075033	Nxpe3	neurexophilin and PC-esterase domain family, member 3	1.52
ENSMUSG00000022218	Tgm1	transglutaminase 1, K polypeptide	1.52
ENSMUSG00000061991	Hist1h2af	histone cluster 1, H2af	1.52
ENSMUSG00000025154	Arhgap19	Rho GTPase activating protein 19	1.51
ENSMUSG00000028312	Smc2	structural maintenance of chromosomes 2	1.51
ENSMUSG00000057193	Slc44a2	solute carrier family 44, member 2	1.51
ENSMUSG00000051220	Ercc6l	excision repair cross-complementing rodent repair deficiency complementation grc	1.51

ENSMUSG00000045273	Cenph	centromere protein H	1.51
ENSMUSG00000020492	Ska2	spindle and kinetochore associated complex subunit 2	1.51
ENSMUSG00000006777	Krt23	keratin 23	1.50
ENSMUSG00000027326	Knl1	kinetochore scaffold 1	1.50
ENSMUSG00000068101	Cenpm	centromere protein M	1.50
ENSMUSG00000072980	Oip5	Opa interacting protein 5	1.50
ENSMUSG00000000489	Pdgfb	platelet derived growth factor, B polypeptide	1.50
ENSMUSG00000034906	Ncaph	non-SMC condensin I complex, subunit H	1.49
ENSMUSG00000082361	Btc	betacellulin, epidermal growth factor family member	1.49
ENSMUSG00000022385	Gtse1	G two S phase expressed protein 1	1.49
ENSMUSG00000026278	Bok	BCL2-related ovarian killer	1.49
ENSMUSG00000024590	Lmnb1	lamin B1	1.48
ENSMUSG00000064288	Hist1h4k	histone cluster 1, H4k	1.48
ENSMUSG00000043439	Epop	elongin BC and polycomb repressive complex 2 associated protein	1.48
ENSMUSG00000001473	Tubb6	tubulin, beta 6 class V	1.48
ENSMUSG00000030641	Ddias	DNA damage-induced apoptosis suppressor	1.48
ENSMUSG00000061615	Hist1h2ab	histone cluster 1, H2ab	1.48
ENSMUSG00000040663	Clcf1	cardiotrophin-like cytokine factor 1	1.48
ENSMUSG00000002297	Dbf4	DBF4 zinc finger	1.47
ENSMUSG00000041859	Mcm3	minichromosome maintenance complex component 3	1.47
ENSMUSG00000038379	Ttk	Ttk protein kinase	1.47
ENSMUSG00000090534	Gm4675	predicted gene 4675	1.47
ENSMUSG00000025758	Plk4	polo like kinase 4	1.46
ENSMUSG00000049539	Hist1h1a	histone cluster 1, H1a	1.46
ENSMUSG00000016382	Pls3	plastin 3 (T-isoform)	1.46
ENSMUSG00000047534	Mis18bp1	MIS18 binding protein 1	1.46
ENSMUSG00000055945	Prr18	proline rich 18	1.46
ENSMUSG00000011267	Zfp296	zinc finger protein 296	1.46
ENSMUSG00000116919	AC165151.1	conserved helix-loop-helix ubiquitous kinase (Chuk) pseudogene	1.46
ENSMUSG00000005233	Spc25	SPC25, NDC80 kinetochore complex component, homolog (S. cerevisiae)	1.45
ENSMUSG00000074825	Itpripl1	inositol 1,4,5-triphosphate receptor interacting protein-like 1	1.45
ENSMUSG00000023008	Fmnl3	formin-like 3	1.45
ENSMUSG00000116102	Gm20082	predicted gene, 20082	1.45
ENSMUSG00000036768	Kif15	kinesin family member 15	1.44
ENSMUSG00000023004	Tuba1b	tubulin, alpha 1B	1.44
ENSMUSG00000029762	Akr1b8	aldo-keto reductase family 1, member B8	1.44
ENSMUSG00000042367	Gjb3	gap junction protein, beta 3	1.44
ENSMUSG00000052852	Reep1	receptor accessory protein 1	1.44
ENSMUSG00000071516	Hist1h2ai	histone cluster 1, H2ai	1.44
ENSMUSG00000062310	Glrp1	glutamine repeat protein 1	1.44
ENSMUSG00000081943	Gm9078	predicted gene 9078	1.44
ENSMUSG00000069266	Hist1h4b	histone cluster 1, H4b	1.43
ENSMUSG00000034295	Fhod3	formin homology 2 domain containing 3	1.43
ENSMUSG00000024660	Incenp	inner centromere protein	1.42
ENSMUSG00000100210	Hist1h3f	histone cluster 1, H3f	1.42
ENSMUSG00000036223	Ska1	spindle and kinetochore associated complex subunit 1	1.42
ENSMUSG00000019966	Kitl	kit ligand	1.41
ENSMUSG00000022881	Rfc4	replication factor C (activator 1) 4	1.41
ENSMUSG00000048922	Cdca2	cell division cycle associated 2	1.41
ENSMUSG00000053398	Phgdh	3-phosphoglycerate dehydrogenase	1.41
ENSMUSG00000031756	Cenpn	centromere protein N	1.41
ENSMUSG00000056665	Them6	thioesterase superfamily member 6	1.41
ENSMUSG00000025938	Slco5a1	solute carrier organic anion transporter family, member 5A1	1.41
ENSMUSG00000001228	Uhrf1	ubiquitin-like, containing PHD and RING finger domains, 1	1.40
ENSMUSG00000020185	E2f7	E2F transcription factor 7	1.40
ENSMUSG00000024791	Cdca5	cell division cycle associated 5	1.40
ENSMUSG00000026955	Sapcd2	suppressor APC domain containing 2	1.40
ENSMUSG00000101972	Hist1h3i	histone cluster 1, H3i	1.40
ENSMUSG00000068855	Hist2h2ac	histone cluster 2, H2ac	1.40
ENSMUSG00000004665	Cnn2	calponin 2	1.40
ENSMUSG00000105987	AI506816	expressed sequence AI506816	1.39
ENSMUSG00000002055	Spag5	sperm associated antigen 5	1.39
ENSMUSG00000027803	Wwtr1	WW domain containing transcription regulator 1	1.39
ENSMUSG00000026193	Fn1	fibronectin 1	1.39
ENSMUSG00000027811	4930579G24Rik	RIKEN cDNA 4930579G24 gene	1.39
ENSMUSG00000030342	Cd9	CD9 antigen	1.38
ENSMUSG00000026779	Mastl	microtubule associated serine/threonine kinase-like	1.38

ENSMUSG00000019773	Fbxo5	F-box protein 5	1.38
ENSMUSG00000076437	Selenoh	selenoprotein H	1.37
ENSMUSG00000046591	Ticrr	TOPBP1-interacting checkpoint and replication regulator	1.37
ENSMUSG00000078952	Lncenc1	long non-coding RNA, embryonic stem cells expressed 1	1.37
ENSMUSG00000109404	Gm19963	predicted gene, 19963	1.37
ENSMUSG00000030978	Rrm1	ribonucleotide reductase M1	1.36
ENSMUSG00000029869	Ephb6	Eph receptor B6	1.36
ENSMUSG00000022582	Ly6g	lymphocyte antigen 6 complex, locus G	1.36
ENSMUSG00000005410	Mcm5	minichromosome maintenance complex component 5	1.35
ENSMUSG00000053604	Rpia	ribose 5-phosphate isomerase A	1.35
ENSMUSG00000042489	Clspn	claspin	1.35
ENSMUSG00000029521	Chek2	checkpoint kinase 2	1.35
ENSMUSG00000030513	Pcsk6	proprotein convertase subtilisin/kexin type 6	1.35
ENSMUSG00000051343	Rab11fip5	RAB11 family interacting protein 5 (class I)	1.35
ENSMUSG00000063021	Hist1h2ak	histone cluster 1, H2ak	1.35
ENSMUSG00000069272	Hist1h2ae	histone cluster 1, H2ae	1.35
ENSMUSG00000021765	Fst	follistatin	1.35
ENSMUSG00000028718	Stil	Scl/Tal1 interrupting locus	1.34
ENSMUSG00000032092	Mpzl2	myelin protein zero-like 2	1.34
ENSMUSG00000028378	Ptgr1	prostaglandin reductase 1	1.34
ENSMUSG00000025934	Gsta3	glutathione S-transferase, alpha 3	1.34
ENSMUSG00000073184	Gm10479	predicted gene 10479	1.34
ENSMUSG00000001525	Tubb5	tubulin, beta 5 class I	1.33
ENSMUSG00000028614	Ndc1	NDC1 transmembrane nucleoporin	1.33
ENSMUSG00000048779	P2ry6	pyrimidinergic receptor P2Y, G-protein coupled, 6	1.33
ENSMUSG00000034883	Lrr1	leucine rich repeat protein 1	1.33
ENSMUSG00000049001	Ndnf	neuron-derived neurotrophic factor	1.33
ENSMUSG00000034349	Smc4	structural maintenance of chromosomes 4	1.32
ENSMUSG00000049932	H2afx	H2A histone family, member X	1.32
ENSMUSG00000027381	Bcl2l11	BCL2-like 11 (apoptosis facilitator)	1.32
ENSMUSG00000022325	Pop1	processing of precursor 1, ribonuclease P/MRP family, (S. cerevisiae)	1.32
ENSMUSG00000030254	Rad18	RAD18 E3 ubiquitin protein ligase	1.32
ENSMUSG00000098132	Rassf10	Ras association (RalGDS/AF-6) domain family (N-terminal) member 10	1.32
ENSMUSG00000091021	Gm17300	predicted gene, 17300	1.32
ENSMUSG00000037185	Krt80	keratin 80	1.32
ENSMUSG00000000320	Alox12	arachidonate 12-lipoxygenase	1.32
ENSMUSG00000031004	Mki67	antigen identified by monoclonal antibody Ki 67	1.31
ENSMUSG00000062248	Cks2	CDC28 protein kinase regulatory subunit 2	1.31
ENSMUSG00000001056	Nhp2	NHP2 ribonucleoprotein	1.31
ENSMUSG00000033307	Mif	macrophage migration inhibitory factor (glycosylation-inhibiting factor)	1.31
ENSMUSG00000035351	Nup37	nucleoporin 37	1.31
ENSMUSG00000040658	Dnph1	2'-deoxynucleoside 5'-phosphate N-hydrolase 1	1.31
ENSMUSG00000031861	Lpar2	lysophosphatidic acid receptor 2	1.31
ENSMUSG00000098090	2700099C18Rik	RIKEN cDNA 2700099C18 gene	1.31
ENSMUSG00000037148	Arhgap10	Rho GTPase activating protein 10	1.31
ENSMUSG00000010760	Phlda2	pleckstrin homology like domain, family A, member 2	1.31
ENSMUSG00000028128	F3	coagulation factor III	1.31
ENSMUSG00000108218	Olfr1372-ps1	olfactory receptor 1372, pseudogene 1	1.31
ENSMUSG00000022346	Myc	myelocytomatosis oncogene	1.30
ENSMUSG00000028599	Tnfrsf1b	tumor necrosis factor receptor superfamily, member 1b	1.30
ENSMUSG00000060093	Hist1h4a	histone cluster 1, H4a	1.30
ENSMUSG00000062510	Nsl1	NSL1, MIS12 kinetochore complex component	1.30
ENSMUSG00000078773	Rad54b	RAD54 homolog B (S. cerevisiae)	1.30
ENSMUSG00000031383	Dusp9	dual specificity phosphatase 9	1.30
ENSMUSG00000027210	Meis2	Meis homeobox 2	1.30
ENSMUSG00000083618	Gm11771	predicted gene 11771	1.30
ENSMUSG00000028780	Sema3c	sema domain, immunoglobulin domain (Ig), short basic domain, secreted, (semaph	1.29
ENSMUSG00000005732	Ranbp1	RAN binding protein 1	1.29
ENSMUSG00000035455	Fignl1	fidgetin-like 1	1.29
ENSMUSG00000029414	Kntc1	kinetochore associated 1	1.29
ENSMUSG00000078532	Nkain1	Na+/K+ transporting ATPase interacting 1	1.29
ENSMUSG00000056904	Gm5620	predicted gene 5620	1.29
ENSMUSG00000062007	Hsh2d	hematopoietic SH2 domain containing	1.29
ENSMUSG00000032108	D730048I06Rik	RIKEN cDNA D730048I06 gene	1.29
ENSMUSG00000000028	Cdc45	cell division cycle 45	1.28
ENSMUSG00000031697	Orc6	origin recognition complex, subunit 6	1.28
ENSMUSG00000027752	Exosc8	exosome component 8	1.28

ENSMUSG00000024613	Tcof1	treacle ribosome biogenesis factor 1	1.28
ENSMUSG00000051235	Gen1	GEN1, Holliday junction 5' flap endonuclease	1.28
ENSMUSG00000030929	Eri2	exoribonuclease 2	1.28
ENSMUSG00000032586	Traip	TRAF-interacting protein	1.28
ENSMUSG00000019997	Ctgf	connective tissue growth factor	1.28
ENSMUSG00000079357	Gm11100	predicted gene 11100	1.28
ENSMUSG00000022673	Mcm4	minichromosome maintenance complex component 4	1.27
ENSMUSG00000005470	Asf1b	anti-silencing function 1B histone chaperone	1.27
ENSMUSG00000039994	Timeless	timeless circadian clock 1	1.27
ENSMUSG00000028031	Dkk2	dickkopf WNT signaling pathway inhibitor 2	1.27
ENSMUSG00000035104	Eva1a	eva-1 homolog A (C. elegans)	1.27
ENSMUSG00000098113	Gm2445	predicted gene 2445	1.27
ENSMUSG00000041219	Arhgap11a	Rho GTPase activating protein 11A	1.26
ENSMUSG00000028896	Rcc1	regulator of chromosome condensation 1	1.26
ENSMUSG00000021115	Vrk1	vaccinia related kinase 1	1.26
ENSMUSG00000040034	Nup43	nucleoporin 43	1.26
ENSMUSG00000022978	Mis18a	MIS18 kinetochore protein A	1.26
ENSMUSG00000027353	Mcm8	minichromosome maintenance 8 homologous recombination repair factor	1.26
ENSMUSG00000027907	S100a11	S100 calcium binding protein A11	1.26
ENSMUSG00000038508	Gdf15	growth differentiation factor 15	1.26
ENSMUSG00000040204	Pclaf	PCNA clamp associated factor	1.25
ENSMUSG00000028044	Cks1b	CDC28 protein kinase 1b	1.25
ENSMUSG00000028587	Orc1	origin recognition complex, subunit 1	1.25
ENSMUSG00000068270	Shroom4	shroom family member 4	1.25
ENSMUSG00000013415	Igf2bp1	insulin-like growth factor 2 mRNA binding protein 1	1.25
ENSMUSG00000032013	Trim29	tripartite motif-containing 29	1.25
ENSMUSG00000054717	Hmgb2	high mobility group box 2	1.24
ENSMUSG00000047443	Erfe	erythroferrone	1.24
ENSMUSG00000069268	Hist1h2bf	histone cluster 1, H2bf	1.24
ENSMUSG00000030352	Tspan9	tetraspanin 9	1.24
ENSMUSG00000074796	Slc4a11	solute carrier family 4, sodium bicarbonate transporter-like, member 11	1.24
ENSMUSG00000108528	Gm8319	predicted gene 8319	1.24
ENSMUSG00000006218	Fam131c	family with sequence similarity 131, member C	1.24
ENSMUSG00000085627	Gm11222	predicted gene 11222	1.24
ENSMUSG00000030162	Olr1	oxidized low density lipoprotein (lectin-like) receptor 1	1.24
ENSMUSG00000040681	Hmgn1	high mobility group nucleosomal binding domain 1	1.23
ENSMUSG00000007050	Lsm2	LSM2 homolog, U6 small nuclear RNA and mRNA degradation associated	1.23
ENSMUSG00000032232	Cgnl1	cingulin-like 1	1.23
ENSMUSG00000054342	Kcnn4	potassium intermediate/small conductance calcium-activated channel, subfamily N	1.23
ENSMUSG00000039509	Nup133	nucleoporin 133	1.23
ENSMUSG00000020534	Shmt1	serine hydroxymethyltransferase 1 (soluble)	1.23
ENSMUSG00000037206	Islr	immunoglobulin superfamily containing leucine-rich repeat	1.23
ENSMUSG00000047832	Cdca4	cell division cycle associated 4	1.22
ENSMUSG00000025403	Shmt2	serine hydroxymethyltransferase 2 (mitochondrial)	1.22
ENSMUSG00000069273	Hist1h3e	histone cluster 1, H3e	1.22
ENSMUSG00000022246	Rai14	retinoic acid induced 14	1.22
ENSMUSG00000028702	Rad54l	RAD54 like (S. cerevisiae)	1.22
ENSMUSG00000037466	Tedc1	tubulin epsilon and delta complex 1	1.22
ENSMUSG00000032400	Zwilch	zwilch kinetochore protein	1.22
ENSMUSG00000026196	Bard1	BRCA1 associated RING domain 1	1.22
ENSMUSG00000027635	Dsn1	DSN1 homolog, MIS12 kinetochore complex component	1.22
ENSMUSG00000031629	Cenpu	centromere protein U	1.22
ENSMUSG00000031351	Zfp185	zinc finger protein 185	1.22
ENSMUSG00000030935	Acsm3	acyl-CoA synthetase medium-chain family member 3	1.22
ENSMUSG00000062588	Gm6104	predicted gene 6104	1.22
ENSMUSG00000021509	Slc25a48	solute carrier family 25, member 48	1.22
ENSMUSG00000111729	Gm48038	predicted gene, 48038	1.22
ENSMUSG00000029430	Ran	RAN, member RAS oncogene family	1.21
ENSMUSG00000026434	Nucks1	nuclear casein kinase and cyclin-dependent kinase substrate 1	1.21
ENSMUSG00000005087	Cd44	CD44 antigen	1.21
ENSMUSG00000020592	Sdc1	syndecan 1	1.21
ENSMUSG00000031146	Plp2	proteolipid protein 2	1.21
ENSMUSG00000073705	Cenps	centromere protein S	1.21
ENSMUSG00000096726	Gm5558	predicted gene 5558	1.21
ENSMUSG00000024891	Slc29a2	solute carrier family 29 (nucleoside transporters), member 2	1.21
ENSMUSG00000024401	Tnf	tumor necrosis factor	1.21
ENSMUSG00000018566	Slc2a4	solute carrier family 2 (facilitated glucose transporter), member 4	1.21

ENSMUSG00000037824	Tspan14	tetraspanin 14	1.20
ENSMUSG00000030796	Tead2	TEA domain family member 2	1.20
ENSMUSG00000030528	Blm	Bloom syndrome, RecQ like helicase	1.20
ENSMUSG00000037860	Aim2	absent in melanoma 2	1.20
ENSMUSG00000034205	Loxl2	lysyl oxidase-like 2	1.20
ENSMUSG00000053297	AI854703	expressed sequence AI854703	1.20
ENSMUSG00000005481	Ddx39	DEAD (Asp-Glu-Ala-Asp) box polypeptide 39	1.19
ENSMUSG00000029730	Mcm7	minichromosome maintenance complex component 7	1.19
ENSMUSG00000043336	Filip1l	filamin A interacting protein 1-like	1.19
ENSMUSG00000041506	Rrp9	RRP9, small subunit (SSU) processome component, homolog (yeast)	1.19
ENSMUSG00000025747	Tyms	thymidylate synthase	1.19
ENSMUSG00000026999	Nup35	nucleoporin 35	1.19
ENSMUSG00000016526	Dyrk3	dual-specificity tyrosine-(Y)-phosphorylation regulated kinase 3	1.19
ENSMUSG00000028693	Nasp	nuclear autoantigenic sperm protein (histone-binding)	1.18
ENSMUSG00000032300	1700017B05Rik	RIKEN cDNA 1700017B05 gene	1.18
ENSMUSG00000063018	2010204K13Rik	RIKEN cDNA 2010204K13 gene	1.18
ENSMUSG00000028199	Cryz	crystallin, zeta	1.18
ENSMUSG00000069307	Hist1h2bq	histone cluster 1, H2bq	1.18
ENSMUSG00000084353	Gm15452	predicted gene 15452	1.18
ENSMUSG00000032940	Rbm11	RNA binding motif protein 11	1.18
ENSMUSG00000006585	Cdt1	chromatin licensing and DNA replication factor 1	1.17
ENSMUSG00000002870	Mcm2	minichromosome maintenance complex component 2	1.17
ENSMUSG00000091625	Lsm5	LSM5 homolog, U6 small nuclear RNA and mRNA degradation associated	1.17
ENSMUSG00000022945	Chaf1b	chromatin assembly factor 1, subunit B (p60)	1.17
ENSMUSG00000026646	Suv39h2	suppressor of variegation 3-9 2	1.17
ENSMUSG00000022422	Dscc1	DNA replication and sister chromatid cohesion 1	1.17
ENSMUSG00000080186	Gm14448	predicted gene 14448	1.17
ENSMUSG00000046589	Lrrc8e	leucine rich repeat containing 8 family, member E	1.17
ENSMUSG00000082163	Gm14276	predicted gene 14276	1.17
ENSMUSG00000087201	Gm15261	predicted gene 15261	1.17
ENSMUSG00000019961	Tmpo	thymopoietin	1.16
ENSMUSG00000027405	Nop56	NOP56 ribonucleoprotein	1.16
ENSMUSG00000056394	Lig1	ligase I, DNA, ATP-dependent	1.16
ENSMUSG00000024732	Ccdc86	coiled-coil domain containing 86	1.16
ENSMUSG00000026669	Mcm10	minichromosome maintenance 10 replication initiation factor	1.16
ENSMUSG00000024844	Banf1	barrier to autointegration factor 1	1.16
ENSMUSG00000053801	Grwd1	glutamate-rich WD repeat containing 1	1.16
ENSMUSG00000055760	Gemin6	gem nuclear organelle associated protein 6	1.16
ENSMUSG00000020937	Plcd3	phospholipase C, delta 3	1.16
ENSMUSG00000041801	Phlda3	pleckstrin homology like domain, family A, member 3	1.16
ENSMUSG00000063605	Ccdc102a	coiled-coil domain containing 102A	1.16
ENSMUSG00000102840	Gm38037	predicted gene, 38037	1.16
ENSMUSG00000047246	Hist1h2be	histone cluster 1, H2be	1.15
ENSMUSG00000032640	Chsy1	chondroitin sulfate synthase 1	1.15
ENSMUSG00000027203	Dut	deoxyuridine triphosphatase	1.15
ENSMUSG00000074476	Spc24	SPC24, NDC80 kinetochore complex component, homolog (S. cerevisiae)	1.15
ENSMUSG00000023919	Cenpq	centromere protein Q	1.15
ENSMUSG00000027257	Pacsin3	protein kinase C and casein kinase substrate in neurons 3	1.15
ENSMUSG00000032215	Rsl24d1	ribosomal L24 domain containing 1	1.15
ENSMUSG00000017491	Rarb	retinoic acid receptor, beta	1.15
ENSMUSG00000001025	S100a6	S100 calcium binding protein A6 (calcyclin)	1.15
ENSMUSG00000106358	Gm7047	predicted gene 7047	1.15
ENSMUSG00000082878	Gm12583	predicted gene 12583	1.15
ENSMUSG00000017499	Cdc6	cell division cycle 6	1.14
ENSMUSG00000091405	Hist2h4	histone cluster 2, H4	1.14
ENSMUSG00000029516	Cit	citron	1.14
ENSMUSG00000032815	Fanca	Fanconi anemia, complementation group A	1.14
ENSMUSG00000044037	Als2cl	ALS2 C-terminal like	1.14
ENSMUSG00000040187	Arntl2	aryl hydrocarbon receptor nuclear translocator-like 2	1.14
ENSMUSG00000063628	Gm7665	predicted pseudogene 7665	1.14
ENSMUSG00000042272	Sestd1	SEC14 and spectrin domains 1	1.13
ENSMUSG00000044254	Pcsk9	proprotein convertase subtilisin/kexin type 9	1.13
ENSMUSG00000027878	Notch2	notch 2	1.13
ENSMUSG00000021958	Pinx1	PIN2/TERF1 interacting, telomerase inhibitor 1	1.13
ENSMUSG00000037419	Endod1	endonuclease domain containing 1	1.13
ENSMUSG00000069793	Slfn9	schlafen 9	1.13
ENSMUSG00000039748	Exo1	exonuclease 1	1.13

ENSMUSG00000000555	Itga5	integrin alpha 5 (fibronectin receptor alpha)	1.13
ENSMUSG00000030346	Rad51ap1	RAD51 associated protein 1	1.13
ENSMUSG00000051517	Arhgef39	Rho guanine nucleotide exchange factor (GEF) 39	1.13
ENSMUSG00000031379	Pir	pirin	1.13
ENSMUSG00000075266	Cenpw	centromere protein W	1.13
ENSMUSG00000049916	2610318N02Rik	RIKEN cDNA 2610318N02 gene	1.13
ENSMUSG00000006403	Adamts4	a disintegrin-like and metallopeptidase (reprolysin type) with thrombospondin type	1.13
ENSMUSG00000099902	Gm12115	predicted gene 12115	1.13
ENSMUSG00000109186	Gm34821	predicted gene, 34821	1.13
ENSMUSG00000034317	Trim59	tripartite motif-containing 59	1.12
ENSMUSG00000032184	Lysmd2	LysM, putative peptidoglycan-binding, domain containing 2	1.12
ENSMUSG00000047044	D030056L22Rik	RIKEN cDNA D030056L22 gene	1.12
ENSMUSG00000034206	Polq	polymerase (DNA directed), theta	1.12
ENSMUSG00000043924	Ncmap	noncompact myelin associated protein	1.12
ENSMUSG00000042215	Bag2	BCL2-associated athanogene 2	1.12
ENSMUSG00000110218	Gm20219	predicted gene, 20219	1.12
ENSMUSG00000039187	Fanci	Fanconi anemia, complementation group I	1.12
ENSMUSG00000040990	Sh3kbp1	SH3-domain kinase binding protein 1	1.12
ENSMUSG00000033508	Asprv1	aspartic peptidase, retroviral-like 1	1.12
ENSMUSG00000052833	Sae1	SUMO1 activating enzyme subunit 1	1.11
ENSMUSG00000022678	Nde1	nudE neurodevelopment protein 1	1.11
ENSMUSG00000057113	Npm1	nucleophosmin 1	1.11
ENSMUSG00000015749	Anp32e	acidic (leucine-rich) nuclear phosphoprotein 32 family, member E	1.11
ENSMUSG00000002718	Cse1l	chromosome segregation 1-like (S. cerevisiae)	1.11
ENSMUSG00000026234	Ncl	nucleolin	1.11
ENSMUSG00000026355	Mcm6	minichromosome maintenance complex component 6	1.11
ENSMUSG00000061458	Nol10	nucleolar protein 10	1.11
ENSMUSG00000022636	Alcam	activated leukocyte cell adhesion molecule	1.11
ENSMUSG00000038047	Haus6	HAUS augmin-like complex, subunit 6	1.11
ENSMUSG00000064326	Siva1	SIVA1, apoptosis-inducing factor	1.11
ENSMUSG00000027478	Dnmt3b	DNA methyltransferase 3B	1.11
ENSMUSG00000036992	Nxt1	NTF2-related export protein 1	1.11
ENSMUSG00000044948	Cfap43	cilia and flagella associated protein 43	1.11
ENSMUSG00000060639	Hist1h4i	histone cluster 1, H4i	1.11
ENSMUSG00000006356	Crip2	cysteine rich protein 2	1.11
ENSMUSG00000055653	Gpc3	glypican 3	1.11
ENSMUSG00000114515	Aldoa	aldolase A, fructose-bisphosphate	1.11
ENSMUSG00000066551	Hmgb1	high mobility group box 1	1.10
ENSMUSG00000042029	Ncapg2	non-SMC condensin II complex, subunit G2	1.10
ENSMUSG00000021193	Pitrm1	pitrilysin metallepetidase 1	1.10
ENSMUSG00000109511	Nup62	nucleoporin 62	1.10
ENSMUSG00000032939	Nup93	nucleoporin 93	1.10
ENSMUSG00000070348	Ccnd1	cyclin D1	1.10
ENSMUSG00000026020	Nop58	NOP58 ribonucleoprotein	1.10
ENSMUSG00000024925	Rnaseh2c	ribonuclease H2, subunit C	1.10
ENSMUSG00000021131	Erh	ERH mRNA splicing and mitosis factor	1.10
ENSMUSG00000028010	Gar1	GAR1 ribonucleoprotein	1.10
ENSMUSG00000032344	Mb21d1	Mab-21 domain containing 1	1.10
ENSMUSG00000022464	Slc38a4	solute carrier family 38, member 4	1.10
ENSMUSG00000024479	Mal2	mal, T cell differentiation protein 2	1.10
ENSMUSG00000014075	Tctex1d2	Tctex1 domain containing 2	1.10
ENSMUSG00000078249	Hmga1b	high mobility group AT-hook 1B	1.10
ENSMUSG00000115009	G930009F23Rik	RIKEN cDNA G930009F23 gene	1.10
ENSMUSG00000025134	Alyref	Aly/REF export factor	1.09
ENSMUSG00000058799	Nap1l1	nucleosome assembly protein 1-like 1	1.09
ENSMUSG00000034192	Lsm3	LSM3 homolog, U6 small nuclear RNA and mRNA degradation associated	1.09
ENSMUSG00000096010	Hist4h4	histone cluster 4, H4	1.09
ENSMUSG00000001707	Eef1e1	eukaryotic translation elongation factor 1 epsilon 1	1.09
ENSMUSG00000024800	Rpp30	ribonuclease P/MRP 30 subunit	1.09
ENSMUSG00000032643	Fhl3	four and a half LIM domains 3	1.09
ENSMUSG00000024301	Kifc5b	kinesin family member C5B	1.09
ENSMUSG00000021391	Cenpp	centromere protein P	1.09
ENSMUSG00000021418	Rpp40	ribonuclease P 40 subunit	1.09
ENSMUSG00000027454	Gins1	GINS complex subunit 1 (Psf1 homolog)	1.09
ENSMUSG00000031112	Stk26	serine/threonine kinase 26	1.09
ENSMUSG00000022177	Haus4	HAUS augmin-like complex, subunit 4	1.08
ENSMUSG00000027018	Hat1	histone aminotransferase 1	1.08

ENSMUSG00000030079	Ruvbl1	RuvB-like protein 1	1.08
ENSMUSG00000031403	Dkc1	dyskeratosis congenita 1, dyskerin	1.08
ENSMUSG00000037020	Wdr62	WD repeat domain 62	1.08
ENSMUSG00000012483	Rpa3	replication protein A3	1.08
ENSMUSG00000067367	Lyar	Ly1 antibody reactive clone	1.08
ENSMUSG00000039231	Suv39h1	suppressor of variegation 3-9 1	1.08
ENSMUSG00000039457	Ppl	periplakin	1.08
ENSMUSG00000110631	Gm42047	predicted gene, 42047	1.08
ENSMUSG00000048612	Myof	myoferlin	1.08
ENSMUSG00000050520	Cldn8	claudin 8	1.08
ENSMUSG00000031400	G6pdx	glucose-6-phosphate dehydrogenase X-linked	1.07
ENSMUSG00000033032	Afap1l1	actin filament associated protein 1-like 1	1.07
ENSMUSG00000002835	Chaf1a	chromatin assembly factor 1, subunit A (p150)	1.07
ENSMUSG00000049521	Cdc42ep1	CDC42 effector protein (Rho GTPase binding) 1	1.07
ENSMUSG00000028633	Ctps	cytidine 5'-triphosphate synthase	1.07
ENSMUSG00000037275	Gemin5	gem nuclear organelle associated protein 5	1.07
ENSMUSG00000007080	Pole	polymerase (DNA directed), epsilon	1.07
ENSMUSG00000025289	Prdx4	peroxiredoxin 4	1.07
ENSMUSG00000056305	Usp39	ubiquitin specific peptidase 39	1.07
ENSMUSG00000024691	Fam111a	family with sequence similarity 111, member A	1.07
ENSMUSG00000039055	Eme1	essential meiotic structure-specific endonuclease 1	1.07
ENSMUSG00000039200	Atf7ip2	activating transcription factor 7 interacting protein 2	1.07
ENSMUSG00000023885	Thbs2	thrombospondin 2	1.07
ENSMUSG00000027342	Pcna	proliferating cell nuclear antigen	1.06
ENSMUSG00000026547	Tagln2	transgelin 2	1.06
ENSMUSG00000030189	Ybx3	Y box protein 3	1.06
ENSMUSG00000109324	Prmt1	protein arginine N-methyltransferase 1	1.06
ENSMUSG00000032834	Pwp2	PWP2 periodic tryptophan protein homolog (yeast)	1.06
ENSMUSG00000095567	Noc2l	NOC2 like nucleolar associated transcriptional repressor	1.06
ENSMUSG00000058392	Rrp1b	ribosomal RNA processing 1 homolog B (S. cerevisiae)	1.06
ENSMUSG00000046179	E2f8	E2F transcription factor 8	1.06
ENSMUSG00000028741	Mrto4	mRNA turnover 4, ribosome maturation factor	1.06
ENSMUSG00000025001	Hells	helicase, lymphoid specific	1.06
ENSMUSG00000020974	Pole2	polymerase (DNA directed), epsilon 2 (p59 subunit)	1.06
ENSMUSG00000031563	Wwc2	WW, C2 and coiled-coil domain containing 2	1.06
ENSMUSG00000027624	Epb41l1	erythrocyte membrane protein band 4.1 like 1	1.06
ENSMUSG00000021697	Depdc1b	DEP domain containing 1B	1.06
ENSMUSG00000117256	AC154779.4	peptidylprolyl isomerase A (Ppia) pseudogene	1.06
ENSMUSG00000097797	Gm26901	predicted gene, 26901	1.06
ENSMUSG00000041390	Mdfic	MyoD family inhibitor domain containing	1.06
ENSMUSG00000041688	Amot	angiomotin	1.05
ENSMUSG00000026238	Ptma	prothymosin alpha	1.05
ENSMUSG00000116564	Riok2	RIO kinase 2	1.05
ENSMUSG00000026192	Atic	5-aminoimidazole-4-carboxamide ribonucleotide formyltransferase/IMP cyclohydrc	1.05
ENSMUSG00000002477	Snrpd1	small nuclear ribonucleoprotein D1	1.05
ENSMUSG00000028884	Rpa2	replication protein A2	1.05
ENSMUSG00000024640	Psat1	phosphoserine aminotransferase 1	1.05
ENSMUSG00000026796	Fam129b	family with sequence similarity 129, member B	1.05
ENSMUSG00000030512	Snrpa1	small nuclear ribonucleoprotein polypeptide A'	1.05
ENSMUSG00000021645	Smn1	survival motor neuron 1	1.05
ENSMUSG00000040463	Mybbp1a	MYB binding protein (P160) 1a	1.05
ENSMUSG00000026235	Epha4	Eph receptor A4	1.05
ENSMUSG00000034595	Ppp1r18	protein phosphatase 1, regulatory subunit 18	1.05
ENSMUSG00000010751	Tnfrsf22	tumor necrosis factor receptor superfamily, member 22	1.05
ENSMUSG00000096403	Gm9825	predicted gene 9825	1.05
ENSMUSG00000007033	Hspa1l	heat shock protein 1-like	1.05
ENSMUSG00000003868	Ruvbl2	RuvB-like protein 2	1.04
ENSMUSG00000001674	Ddx18	DEAD (Asp-Glu-Ala-Asp) box polypeptide 18	1.04
ENSMUSG00000060860	Ube2s	ubiquitin-conjugating enzyme E2S	1.04
ENSMUSG00000022391	Rangap1	RAN GTPase activating protein 1	1.04
ENSMUSG00000020929	Eftud2	elongation factor Tu GTP binding domain containing 2	1.04
ENSMUSG00000015176	Nolc1	nucleolar and coiled-body phosphoprotein 1	1.04
ENSMUSG00000022360	Atad2	ATPase family, AAA domain containing 2	1.04
ENSMUSG00000060981	Hist1h4h	histone cluster 1, H4h	1.04
ENSMUSG00000014907	Naf1	nuclear assembly factor 1 ribonucleoprotein	1.04
ENSMUSG00000035960	Apex1	apurinic/apyrimidinic endonuclease 1	1.04
ENSMUSG00000021714	Cenpk	centromere protein K	1.04

ENSMUSG00000067455	Hist1h4j	histone cluster 1, H4j	1.04
ENSMUSG00000095677	Dynlt1f	dynein light chain Tctex-type 1F	1.04
ENSMUSG00000039232	Stx11	syntaxin 11	1.04
ENSMUSG00000019992	Mtfr2	mitochondrial fission regulator 2	1.04
ENSMUSG00000032724	Abtb2	ankyrin repeat and BTB (POZ) domain containing 2	1.04
ENSMUSG00000001131	Timp1	tissue inhibitor of metalloproteinase 1	1.04
ENSMUSG00000116640	AC166114.1	twist basic helix-loop-helix transcription factor 1 neighbor (Twistnb) pseudogene	1.04
ENSMUSG00000025364	Pa2g4	proliferation-associated 2G4	1.03
ENSMUSG00000029014	Dnajc2	DnaJ heat shock protein family (Hsp40) member C2	1.03
ENSMUSG00000033166	Dis3	DIS3 homolog, exosome endoribonuclease and 3'-5' exoribonuclease	1.03
ENSMUSG00000025980	Hspd1	heat shock protein 1 (chaperonin)	1.03
ENSMUSG00000029247	Paics	phosphoribosylaminoimidazole carboxylase, phosphoribosylaminoribosylaminoimic	1.03
ENSMUSG00000020706	Ftsj3	FtsJ RNA methyltransferase homolog 3 (E. coli)	1.03
ENSMUSG00000027330	Cdc25b	cell division cycle 25B	1.03
ENSMUSG00000031434	Morc4	microrchidia 4	1.03
ENSMUSG00000023988	Bysl	bystin-like	1.03
ENSMUSG00000035165	Kcne3	potassium voltage-gated channel, Isk-related subfamily, gene 3	1.03
ENSMUSG00000015217	Hmgb3	high mobility group box 3	1.03
ENSMUSG00000033294	Noc4l	NOC4 like	1.03
ENSMUSG00000025395	Prim1	DNA primase, p49 subunit	1.03
ENSMUSG00000079478	Sssca1	Sjogren syndrome/scleroderma autoantigen 1	1.03
ENSMUSG00000038323	1700066M21Rik	RIKEN cDNA 1700066M21 gene	1.03
ENSMUSG00000028933	Xrcc2	X-ray repair complementing defective repair in Chinese hamster cells 2	1.03
ENSMUSG00000033752	Mnd1	meiotic nuclear divisions 1	1.03
ENSMUSG00000020656	Grhl1	grainyhead like transcription factor 1	1.03
ENSMUSG00000046295	Ankle1	ankyrin repeat and LEM domain containing 1	1.03
ENSMUSG00000079685	Ulbp1	UL16 binding protein 1	1.03
ENSMUSG00000035678	Tnfsf9	tumor necrosis factor (ligand) superfamily, member 9	1.03
ENSMUSG00000058656	Samd12	sterile alpha motif domain containing 12	1.03
ENSMUSG00000080928	Hmgb1-ps6	high mobility group box 1, pseudogene 6	1.03
ENSMUSG00000030098	Grip2	glutamate receptor interacting protein 2	1.03
ENSMUSG00000071724	Smpd5	sphingomyelin phosphodiesterase 5	1.03
ENSMUSG00000054766	Set	SET nuclear oncogene	1.02
ENSMUSG00000061482	Hist1h4d	histone cluster 1, H4d	1.02
ENSMUSG00000054321	Taf4b	TATA-box binding protein associated factor 4b	1.02
ENSMUSG00000028333	Anp32b	acidic (leucine-rich) nuclear phosphoprotein 32 family, member B	1.02
ENSMUSG00000039298	Cdk5rap2	CDK5 regulatory subunit associated protein 2	1.02
ENSMUSG00000068856	Sf3b4	splicing factor 3b, subunit 4	1.02
ENSMUSG00000060288	Ppih	peptidyl prolyl isomerase H	1.02
ENSMUSG00000034612	Chst11	carbohydrate sulfotransferase 11	1.02
ENSMUSG00000050244	Heatr1	HEAT repeat containing 1	1.02
ENSMUSG00000001761	Smo	smoothened, frizzled class receptor	1.02
ENSMUSG00000022792	Yars2	tyrosyl-tRNA synthetase 2 (mitochondrial)	1.02
ENSMUSG00000019853	Hebp2	heme binding protein 2	1.02
ENSMUSG00000050107	Haspin	histone H3 associated protein kinase	1.02
ENSMUSG00000034023	Fancd2	Fanconi anemia, complementation group D2	1.02
ENSMUSG00000097415	AU020206	expressed sequence AU020206	1.02
ENSMUSG00000026404	Ddx59	DEAD (Asp-Glu-Ala-Asp) box polypeptide 59	1.02
ENSMUSG00000039103	Nexn	nexilin	1.02
ENSMUSG00000017765	Slc12a4	solute carrier family 12, member 4	1.02
ENSMUSG00000020834	Dhrs13	dehydrogenase/reductase (SDR family) member 13	1.02
ENSMUSG00000020358	Hnrnpab	heterogeneous nuclear ribonucleoprotein A/B	1.01
ENSMUSG00000022962	Gart	phosphoribosylglycinamide formyltransferase	1.01
ENSMUSG00000028184	Adgrl2	adhesion G protein-coupled receptor L2	1.01
ENSMUSG00000017607	Tns4	tensin 4	1.01
ENSMUSG00000046711	Hmga1	high mobility group AT-hook 1	1.01
ENSMUSG00000001053	N4bp3	NEDD4 binding protein 3	1.01
ENSMUSG00000019214	Chtf18	CTF18, chromosome transmission fidelity factor 18	1.01
ENSMUSG00000027978	Prss12	protease, serine 12 neurotrypsin (motopsin)	1.01
ENSMUSG00000029761	Cald1	caldesmon 1	1.01
ENSMUSG00000044966	Fbxo48	F-box protein 48	1.01
ENSMUSG00000032473	Cldn18	claudin 18	1.01
ENSMUSG00000078157	4931440F15Rik	RIKEN cDNA 4931440F15 gene	1.01
ENSMUSG00000044912	Syt16	synaptotagmin XVI	1.01
ENSMUSG00000025613	Cct8	chaperonin containing Tcp1, subunit 8 (theta)	1.00
ENSMUSG00000014226	Cacybp	calcyclin binding protein	1.00
ENSMUSG00000025534	Gusb	glucuronidase, beta	1.00

ENSMUSG00000022247	Brix1	BRX1, biogenesis of ribosomes	1.00
ENSMUSG00000005846	Rsl1d1	ribosomal L1 domain containing 1	1.00
ENSMUSG00000006715	Gmnn	geminin	1.00
ENSMUSG00000023110	Prmt5	protein arginine N-methyltransferase 5	1.00
ENSMUSG00000031790	Mmp15	matrix metallopeptidase 15	1.00
ENSMUSG00000062127	Cttnbp2nl	CTTNBP2 N-terminal like	1.00
ENSMUSG00000105140	Gm43127	predicted gene 43127	1.00
ENSMUSG00000103576	Gm38188	predicted gene, 38188	1.00
ENSMUSG00000042251	Pm20d1	peptidase M20 domain containing 1	-1.00
ENSMUSG00000073402	Gm8909	predicted gene 8909	-1.00
ENSMUSG00000110773	AC061963.1	novel transcript, antisense to Kmt2a	-1.00
ENSMUSG00000104852	Gm43201	predicted gene 43201	-1.00
ENSMUSG00000032417	Rwdd2a	RWD domain containing 2A	-1.00
ENSMUSG00000004902	Slc25a18	solute carrier family 25 (mitochondrial carrier), member 18	-1.00
ENSMUSG00000107428	Gm44154	predicted gene, 44154	-1.00
ENSMUSG00000032527	Pccb	propionyl Coenzyme A carboxylase, beta polypeptide	-1.01
ENSMUSG00000068587	Mgam	maltase-glucoamylase	-1.01
ENSMUSG00000005686	Ampd3	adenosine monophosphate deaminase 3	-1.01
ENSMUSG00000025467	Prap1	proline-rich acidic protein 1	-1.01
ENSMUSG00000024055	Cyp4f13	cytochrome P450, family 4, subfamily f, polypeptide 13	-1.01
ENSMUSG00000019278	Dpep1	dipeptidase 1 (renal)	-1.01
ENSMUSG00000023959	Clic5	chloride intracellular channel 5	-1.01
ENSMUSG00000039199	Zdhhc1	zinc finger, DHHC domain containing 1	-1.01
ENSMUSG00000054999	Naaladl1	N-acetylated alpha-linked acidic dipeptidase-like 1	-1.01
ENSMUSG00000021684	Pde8b	phosphodiesterase 8B	-1.01
ENSMUSG00000002944	Cd36	CD36 molecule	-1.01
ENSMUSG00000112481	Gm29684	predicted gene, 29684	-1.01
ENSMUSG00000082148	Gm12266	predicted gene 12266	-1.01
ENSMUSG00000026395	Ptprc	protein tyrosine phosphatase, receptor type, C	-1.01
ENSMUSG00000095440	Fignl2	fidgetin-like 2	-1.01
ENSMUSG00000060402	Chst8	carbohydrate (N-acetylgalactosamine 4-0) sulfotransferase 8	-1.01
ENSMUSG00000045689	Pcdhb4	protocadherin beta 4	-1.01
ENSMUSG00000114401	Gm38604	predicted gene, 38604	-1.01
ENSMUSG00000040712	Camta2	calmodulin binding transcription activator 2	-1.02
ENSMUSG00000030102	Itpr1	inositol 1,4,5-trisphosphate receptor 1	-1.02
ENSMUSG00000068742	Cry2	cryptochrome 2 (photolyase-like)	-1.02
ENSMUSG00000066800	Rnasel	ribonuclease L (2', 5'-oligoisoadenylate synthetase-dependent)	-1.02
ENSMUSG00000036833	Pnpla7	patatin-like phospholipase domain containing 7	-1.02
ENSMUSG00000015202	Cnksr3	Cnksr family member 3	-1.02
ENSMUSG00000028211	Trp53inp1	transformation related protein 53 inducible nuclear protein 1	-1.02
ENSMUSG00000000823	Zfp512b	zinc finger protein 512B	-1.02
ENSMUSG00000019989	Enpp3	ectonucleotide pyrophosphatase/phosphodiesterase 3	-1.02
ENSMUSG00000017390	Aldoc	aldolase C, fructose-bisphosphate	-1.02
ENSMUSG00000017204	Gsdma	gasdermin A	-1.02
ENSMUSG00000029188	Slc34a2	solute carrier family 34 (sodium phosphate), member 2	-1.02
ENSMUSG00000022408	Fam83f	family with sequence similarity 83, member F	-1.02
ENSMUSG00000027296	Itpka	inositol 1,4,5-trisphosphate 3-kinase A	-1.02
ENSMUSG00000025396	Hsd17b6	hydroxysteroid (17-beta) dehydrogenase 6	-1.02
ENSMUSG00000026692	Fmo4	flavin containing monooxygenase 4	-1.02
ENSMUSG00000086946	Gm15527	predicted gene 15527	-1.02
ENSMUSG00000037003	Tns2	tensin 2	-1.02
ENSMUSG00000046794	Ppp1r3b	protein phosphatase 1, regulatory subunit 3B	-1.02
ENSMUSG00000064455	n-R5s56	nuclear encoded rRNA 5S 56	-1.02
ENSMUSG00000097258	Gm26767	predicted gene, 26767	-1.02
ENSMUSG00000097006	9530082P21Rik	RIKEN cDNA 9530082P21 gene	-1.02
ENSMUSG00000115918	A930001M01Rik	RIKEN cDNA A930001M01 gene	-1.02
ENSMUSG00000015966	Il17rb	interleukin 17 receptor B	-1.02
ENSMUSG00000086766	Gm15969	predicted gene 15969	-1.02
ENSMUSG00000021566	Slc6a19os	solute carrier family 6 (neurotransmitter transporter), member 19, opposite strand	-1.02
ENSMUSG00000039963	Ccdc40	coiled-coil domain containing 40	-1.02
ENSMUSG00000038011	Dnah10	dynein, axonemal, heavy chain 10	-1.02
ENSMUSG00000034786	Gpsm3	G-protein signalling modulator 3 (AGS3-like, C. elegans)	-1.02
ENSMUSG00000108818	Gm44869	predicted gene 44869	-1.02
ENSMUSG00000030329	Pianp	PILR alpha associated neural protein	-1.02
ENSMUSG00000079497	Gm13420	predicted gene 13420	-1.02
ENSMUSG00000018796	Acsl1	acyl-CoA synthetase long-chain family member 1	-1.03
ENSMUSG00000030465	Psd3	pleckstrin and Sec7 domain containing 3	-1.03

ENSMUSG00000020894	Vamp2	vesicle-associated membrane protein 2	-1.03
ENSMUSG00000039686	Zer1	zyg-11 related, cell cycle regulator	-1.03
ENSMUSG00000031808	Slc27a1	solute carrier family 27 (fatty acid transporter), member 1	-1.03
ENSMUSG00000029161	Cgref1	cell growth regulator with EF hand domain 1	-1.03
ENSMUSG00000003363	Pld3	phospholipase D family, member 3	-1.03
ENSMUSG00000034947	Tmem106a	transmembrane protein 106A	-1.03
ENSMUSG00000112944	Gm48885	predicted gene, 48885	-1.03
ENSMUSG00000026826	Nr4a2	nuclear receptor subfamily 4, group A, member 2	-1.03
ENSMUSG00000044080	S100a1	S100 calcium binding protein A1	-1.03
ENSMUSG00000108954	Gm44901	predicted gene 44901	-1.03
ENSMUSG00000054598	9130230L23Rik	RIKEN cDNA 9130230L23 gene	-1.03
ENSMUSG00000074195	Clca4b	chloride channel accessory 4B	-1.03
ENSMUSG00000029082	Bst1	bone marrow stromal cell antigen 1	-1.03
ENSMUSG00000028785	Hpca	hippocalcin	-1.03
ENSMUSG00000058163	Gm5431	predicted gene 5431	-1.03
ENSMUSG00000097321	1700028E10Rik	RIKEN cDNA 1700028E10 gene	-1.03
ENSMUSG00000067049	Unc93a	unc-93 homolog A	-1.03
ENSMUSG00000103037	Pcdhgb1	protocadherin gamma subfamily B, 1	-1.03
ENSMUSG00000019055	Plod1	procollagen-lysine, 2-oxoglutarate 5-dioxygenase 1	-1.03
ENSMUSG00000104576	F830115B05Rik	RIKEN cDNA F830115B05 gene	-1.03
ENSMUSG00000107227	Gm42559	predicted gene 42559	-1.03
ENSMUSG00000072974	Gm4787	predicted gene 4787	-1.03
ENSMUSG00000114891	Gm47272	predicted gene, 47272	-1.03
ENSMUSG00000111447	Gm48249	predicted gene, 48249	-1.03
ENSMUSG00000002033	Cd3g	CD3 antigen, gamma polypeptide	-1.03
ENSMUSG00000087055	Gm11948	predicted gene 11948	-1.03
ENSMUSG00000054277	Arfgap3	ADP-ribosylation factor GTPase activating protein 3	-1.04
ENSMUSG00000036687	Tmem184a	transmembrane protein 184a	-1.04
ENSMUSG00000061455	Stx17	syntaxin 17	-1.04
ENSMUSG00000029727	Cyp3a13	cytochrome P450, family 3, subfamily a, polypeptide 13	-1.04
ENSMUSG00000032010	Usp2	ubiquitin specific peptidase 2	-1.04
ENSMUSG00000037826	Ppm1k	protein phosphatase 1K (PP2C domain containing)	-1.04
ENSMUSG00000029335	Bmp3	bone morphogenetic protein 3	-1.04
ENSMUSG00000042041	2010003K11Rik	RIKEN cDNA 2010003K11 gene	-1.04
ENSMUSG00000068349	Gml	glycosylphosphatidylinositol anchored molecule like	-1.04
ENSMUSG00000019762	Iyd	iodotyrosine deiodinase	-1.04
ENSMUSG00000047394	Odf3b	outer dense fiber of sperm tails 3B	-1.04
ENSMUSG00000104535	Gm42686	predicted gene 42686	-1.04
ENSMUSG00000113853	Gm47583	predicted gene, 47583	-1.04
ENSMUSG00000057054	Inca1	inhibitor of CDK, cyclin A1 interacting protein 1	-1.04
ENSMUSG00000083149	Gm11380	predicted gene 11380	-1.04
ENSMUSG00000052364	B630019K06Rik	RIKEN cDNA B630019K06 gene	-1.04
ENSMUSG00000115186	Gm49417	predicted gene, 49417	-1.04
ENSMUSG00000097566	A930024N18Rik	RIKEN cDNA A930024N18 gene	-1.04
ENSMUSG00000042106	Inka1	inka box actin regulator 1	-1.04
ENSMUSG00000034563	Ccpg1	cell cycle progression 1	-1.04
ENSMUSG00000099310	Gm19427	predicted gene, 19427	-1.04
ENSMUSG00000049538	Adamts16	a disintegrin-like and metallopeptidase (reprolysin type) with thrombospondin type	-1.04
ENSMUSG00000038648	Creb3l2	cAMP responsive element binding protein 3-like 2	-1.05
ENSMUSG00000022575	Gsdmd	gasdermin D	-1.05
ENSMUSG00000044786	Zfp36	zinc finger protein 36	-1.05
ENSMUSG00000002227	Mov10	Moloney leukemia virus 10	-1.05
ENSMUSG00000025557	Slc15a1	solute carrier family 15 (oligopeptide transporter), member 1	-1.05
ENSMUSG00000028517	Plpp3	phospholipid phosphatase 3	-1.05
ENSMUSG00000060487	Samd5	sterile alpha motif domain containing 5	-1.05
ENSMUSG00000042439	Zfp532	zinc finger protein 532	-1.05
ENSMUSG00000055114	Anxa13	annexin A13	-1.05
ENSMUSG00000047617	Paxx	non-homologous end joining factor	-1.05
ENSMUSG00000078650	G6pc	glucose-6-phosphatase, catalytic	-1.05
ENSMUSG00000020121	Srgap1	SLIT-ROBO Rho GTPase activating protein 1	-1.05
ENSMUSG00000043432	Leng9	leukocyte receptor cluster (LRC) member 9	-1.05
ENSMUSG00000030930	Chst15	carbohydrate (N-acetylgalactosamine 4-sulfate 6-O) sulfotransferase 15	-1.05
ENSMUSG00000108181	C030015A19Rik	RIKEN cDNA C030015A19 gene	-1.05
ENSMUSG00000050640	Tmem150c	transmembrane protein 150C	-1.05
ENSMUSG00000107549	Gm43961	predicted gene, 43961	-1.05
ENSMUSG00000089636	1700058P15Rik	RIKEN cDNA 1700058P15 gene	-1.05
ENSMUSG00000040856	Dlk1	delta like non-canonical Notch ligand 1	-1.05

ENSMUSG00000102148	Gm38059	predicted gene, 38059	-1.05
ENSMUSG00000103701	Gm10728	predicted gene 10728	-1.05
ENSMUSG00000105083	Gm42699	predicted gene 42699	-1.05
ENSMUSG00000032065	Tex12	testis expressed 12	-1.05
ENSMUSG00000095041	AC149090.1	NULL	-1.06
ENSMUSG00000060227	Casc4	cancer susceptibility candidate 4	-1.06
ENSMUSG00000043668	Tox3	TOX high mobility group box family member 3	-1.06
ENSMUSG00000079507	H2-Q1	histocompatibility 2, Q region locus 1	-1.06
ENSMUSG00000028307	Aldob	aldolase B, fructose-bisphosphate	-1.06
ENSMUSG00000047193	Dync2h1	dynein cytoplasmic 2 heavy chain 1	-1.06
ENSMUSG00000030492	Slc7a9	solute carrier family 7 (cationic amino acid transporter, y+ system), member 9	-1.06
ENSMUSG00000096929	A330023F24Rik	RIKEN cDNA A330023F24 gene	-1.06
ENSMUSG00000091387	Gcnt4	glucosaminyl (N-acetyl) transferase 4, core 2 (beta-1,6-N-acetylglucosaminyltransfe	-1.06
ENSMUSG00000013643	Lypd8	LY6/PLAUR domain containing 8	-1.06
ENSMUSG00000054723	Vmac	vimentin-type intermediate filament associated coiled-coil protein	-1.06
ENSMUSG00000020641	Rsad2	radical S-adenosyl methionine domain containing 2	-1.06
ENSMUSG00000110277	Gm45871	predicted gene 45871	-1.06
ENSMUSG00000023017	Asic1	acid-sensing (proton-gated) ion channel 1	-1.06
ENSMUSG00000039339	Mfsd4b2	major facilitator superfamily domain containing 4B2	-1.06
ENSMUSG00000050944	Efcab5	EF-hand calcium binding domain 5	-1.06
ENSMUSG00000026888	Grb14	growth factor receptor bound protein 14	-1.06
ENSMUSG00000030107	Usp18	ubiquitin specific peptidase 18	-1.06
ENSMUSG00000022840	Adcy5	adenylate cyclase 5	-1.06
ENSMUSG00000075604	Cyp11b1	cytochrome P450, family 11, subfamily b, polypeptide 1	-1.06
ENSMUSG00000043811	Rtn4r	reticulon 4 receptor	-1.06
ENSMUSG00000093668	Pou5f2	POU domain class 5, transcription factor 2	-1.06
ENSMUSG00000000686	Abhd15	abhydrolase domain containing 15	-1.06
ENSMUSG00000103632	Gm38329	predicted gene, 38329	-1.06
ENSMUSG00000020609	Apob	apolipoprotein B	-1.07
ENSMUSG00000033618	Map3k13	mitogen-activated protein kinase kinase kinase 13	-1.07
ENSMUSG00000051359	Ncald	neurocalcin delta	-1.07
ENSMUSG00000014361	Mertk	c-mer proto-oncogene tyrosine kinase	-1.07
ENSMUSG00000020142	Slc1a4	solute carrier family 1 (glutamate/neutral amino acid transporter), member 4	-1.07
ENSMUSG00000032080	Apoa4	apolipoprotein A-IV	-1.07
ENSMUSG00000052595	A1cf	APOBEC1 complementation factor	-1.07
ENSMUSG00000102752	Gm7694	predicted gene 7694	-1.07
ENSMUSG00000055897	Ppp4r1l-ps	protein phosphatase 4, regulatory subunit 1-like, pseudogene	-1.07
ENSMUSG00000017697	Ada	adenosine deaminase	-1.07
ENSMUSG00000032593	Amigo3	adhesion molecule with Ig like domain 3	-1.07
ENSMUSG00000031886	Ces2e	carboxylesterase 2E	-1.07
ENSMUSG00000096243	Gm24265	predicted gene, 24265	-1.07
ENSMUSG00000029001	Fbxo44	F-box protein 44	-1.07
ENSMUSG00000079038	D130040H23Rik	RIKEN cDNA D130040H23 gene	-1.07
ENSMUSG00000045775	Slc16a5	solute carrier family 16 (monocarboxylic acid transporters), member 5	-1.07
ENSMUSG00000031173	Otc	ornithine transcarbamylase	-1.07
ENSMUSG00000036216	Leap2	liver-expressed antimicrobial peptide 2	-1.07
ENSMUSG00000109852	Gm45360	predicted gene 45360	-1.07
ENSMUSG00000027438	Napb	N-ethylmaleimide sensitive fusion protein attachment protein beta	-1.07
ENSMUSG00000031445	Proz	protein Z, vitamin K-dependent plasma glycoprotein	-1.07
ENSMUSG00000103183	Gm37090	predicted gene, 37090	-1.07
ENSMUSG00000112990	Gm47372	predicted gene, 47372	-1.07
ENSMUSG00000114443	Gm19241	predicted gene, 19241	-1.07
ENSMUSG00000041323	Ak7	adenylate kinase 7	-1.07
ENSMUSG00000005493	Msh4	mutS homolog 4	-1.07
ENSMUSG00000095280	Gm21738	predicted gene, 21738	-1.07
ENSMUSG00000086133	Gm16331	predicted gene 16331	-1.07
ENSMUSG00000054901	Arhgef33	Rho guanine nucleotide exchange factor (GEF) 33	-1.07
ENSMUSG00000030275	Etnk1	ethanolamine kinase 1	-1.08
ENSMUSG00000067212	H2-T23	histocompatibility 2, T region locus 23	-1.08
ENSMUSG00000028088	Fmo5	flavin containing monooxygenase 5	-1.08
ENSMUSG00000030155	Clec2e	C-type lectin domain family 2, member e	-1.08
ENSMUSG00000054422	Fabp1	fatty acid binding protein 1, liver	-1.08
ENSMUSG00000039853	Trim14	tripartite motif-containing 14	-1.08
ENSMUSG00000028024	Enpep	glutamyl aminopeptidase	-1.08
ENSMUSG00000029269	Sult1b1	sulfotransferase family 1B, member 1	-1.08
ENSMUSG00000022938	Fam3b	family with sequence similarity 3, member B	-1.08
ENSMUSG00000035429	Ptprh	protein tyrosine phosphatase, receptor type, H	-1.08

ENSMUSG00000039911	Spsb1	splA/ryanodine receptor domain and SOCS box containing 1	-1.08
ENSMUSG00000005237	Dnah2	dynein, axonemal, heavy chain 2	-1.08
ENSMUSG00000075010	AW112010	expressed sequence AW112010	-1.08
ENSMUSG00000105358	Gm43099	predicted gene 43099	-1.08
ENSMUSG00000050357	Carmil2	capping protein regulator and myosin 1 linker 2	-1.08
ENSMUSG00000023132	Gzma	granzyme A	-1.08
ENSMUSG00000110928	Gm48114	predicted gene, 48114	-1.08
ENSMUSG00000027489	Necab3	N-terminal EF-hand calcium binding protein 3	-1.08
ENSMUSG00000029054	Gabrd	gamma-aminobutyric acid (GABA) A receptor, subunit delta	-1.08
ENSMUSG00000103222	Gm37729	predicted gene, 37729	-1.08
ENSMUSG00000022629	Kif21a	kinesin family member 21A	-1.09
ENSMUSG00000018126	Baiap2l2	BAI1-associated protein 2-like 2	-1.09
ENSMUSG00000035112	Wnk4	WNK lysine deficient protein kinase 4	-1.09
ENSMUSG00000005958	Ephb3	Eph receptor B3	-1.09
ENSMUSG00000006395	Hyi	hydroxypyruvate isomerase (putative)	-1.09
ENSMUSG00000021676	Iqgap2	IQ motif containing GTPase activating protein 2	-1.09
ENSMUSG00000001225	Slc26a3	solute carrier family 26, member 3	-1.09
ENSMUSG00000043831	Lysmd4	LysM, putative peptidoglycan-binding, domain containing 4	-1.09
ENSMUSG00000038178	Slc43a2	solute carrier family 43, member 2	-1.09
ENSMUSG00000037736	Limch1	LIM and calponin homology domains 1	-1.09
ENSMUSG00000026170	Cyp27a1	cytochrome P450, family 27, subfamily a, polypeptide 1	-1.09
ENSMUSG00000042286	Stab1	stabilin 1	-1.09
ENSMUSG00000025743	Sdc3	syndecan 3	-1.09
ENSMUSG00000040740	Slc25a34	solute carrier family 25, member 34	-1.09
ENSMUSG00000049536	Tceal1	transcription elongation factor A (SII)-like 1	-1.09
ENSMUSG00000026586	Prrx1	paired related homeobox 1	-1.09
ENSMUSG00000038527	C1rl	complement component 1, r subcomponent-like	-1.09
ENSMUSG00000042417	Ccno	cyclin O	-1.09
ENSMUSG00000059852	Kcng2	potassium voltage-gated channel, subfamily G, member 2	-1.09
ENSMUSG00000001542	Ell2	elongation factor RNA polymerase II 2	-1.10
ENSMUSG00000027799	Nbea	neurobeachin	-1.10
ENSMUSG00000029470	P2rx4	purinergic receptor P2X, ligand-gated ion channel 4	-1.10
ENSMUSG00000071356	Reg3b	regenerating islet-derived 3 beta	-1.10
ENSMUSG00000030650	Tmc5	transmembrane channel-like gene family 5	-1.10
ENSMUSG00000074715	Ccl28	chemokine (C-C motif) ligand 28	-1.10
ENSMUSG00000049858	Suox	sulfite oxidase	-1.10
ENSMUSG00000024140	Epas1	endothelial PAS domain protein 1	-1.10
ENSMUSG00000022383	Ppara	peroxisome proliferator activated receptor alpha	-1.10
ENSMUSG00000058063	Trim31	tripartite motif-containing 31	-1.10
ENSMUSG00000068341	Reg3d	regenerating islet-derived 3 delta	-1.10
ENSMUSG00000032489	Kif9	kinesin family member 9	-1.10
ENSMUSG00000024253	Dync2li1	dynein cytoplasmic 2 light intermediate chain 1	-1.10
ENSMUSG00000100094	1810008I18Rik	RIKEN cDNA 1810008I18 gene	-1.10
ENSMUSG00000059013	Sh2d3c	SH2 domain containing 3C	-1.10
ENSMUSG00000047242	Taf9b	TATA-box binding protein associated factor 9B	-1.10
ENSMUSG00000087047	1700110K17Rik	RIKEN cDNA 1700110K17 gene	-1.10
ENSMUSG00000087691	Gm15674	predicted gene 15674	-1.10
ENSMUSG00000029352	Crybb3	crystallin, beta B3	-1.10
ENSMUSG00000020836	Coro6	coronin 6	-1.10
ENSMUSG00000033487	Fndc3a	fibronectin type III domain containing 3A	-1.11
ENSMUSG00000028555	Ttc39a	tetratricopeptide repeat domain 39A	-1.11
ENSMUSG00000020246	Hcfc2	host cell factor C2	-1.11
ENSMUSG00000022040	Ephx2	epoxide hydrolase 2, cytoplasmic	-1.11
ENSMUSG00000039899	Fgl2	fibrinogen-like protein 2	-1.11
ENSMUSG00000024190	Dusp1	dual specificity phosphatase 1	-1.11
ENSMUSG00000062638	Btnl1	butyrophilin-like 1	-1.11
ENSMUSG00000010601	Apol7a	apolipoprotein L 7a	-1.11
ENSMUSG00000036989	Trim3	tripartite motif-containing 3	-1.11
ENSMUSG00000022180	Slc7a8	solute carrier family 7 (cationic amino acid transporter, y+ system), member 8	-1.11
ENSMUSG00000021751	Acox2	acyl-Coenzyme A oxidase 2, branched chain	-1.11
ENSMUSG00000099746	Ppnr	per-pentamer repeat gene	-1.11
ENSMUSG00000037049	Smpd1	sphingomyelin phosphodiesterase 1, acid lysosomal	-1.11
ENSMUSG00000024503	Spink1	serine peptidase inhibitor, Kazal type 1	-1.11
ENSMUSG00000038496	Slc19a3	solute carrier family 19, member 3	-1.11
ENSMUSG00000023987	Pgc	progastricsin (pepsinogen C)	-1.11
ENSMUSG00000029156	Sgcb	sarcoglycan, beta (dystrophin-associated glycoprotein)	-1.11
ENSMUSG00000039239	Tgfb2	transforming growth factor, beta 2	-1.11

ENSMUSG00000110276	Gm45330	predicted gene 45330	-1.11
ENSMUSG00000107456	Gm10400	predicted gene 10400	-1.11
ENSMUSG00000090062	Galnt6os	polypeptide N-acetylgalactosaminyltransferase 6, opposite strand	-1.11
ENSMUSG00000117182	CT025731.2	ribosomal protein S6 (Rps6) pseudogene	-1.11
ENSMUSG00000035969	Rusc2	RUN and SH3 domain containing 2	-1.11
ENSMUSG00000023036	Pcdhgc4	protocadherin gamma subfamily C, 4	-1.11
ENSMUSG00000093916	Gm379	predicted gene 379	-1.11
ENSMUSG00000097578	Gm26798	predicted gene, 26798	-1.11
ENSMUSG00000046056	Sbsn	suprabasin	-1.11
ENSMUSG00000033191	Tie1	tyrosine kinase with immunoglobulin-like and EGF-like domains 1	-1.11
ENSMUSG00000100572	2410021H03Rik	RIKEN cDNA 2410021H03 gene	-1.11
ENSMUSG00000086991	Gm15334	predicted gene 15334	-1.11
ENSMUSG00000113250	Gm48585	predicted gene, 48585	-1.11
ENSMUSG00000113864	Gm48381	predicted gene, 48381	-1.11
ENSMUSG00000036151	Tm6sf2	transmembrane 6 superfamily member 2	-1.12
ENSMUSG00000038375	Trp53inp2	transformation related protein 53 inducible nuclear protein 2	-1.12
ENSMUSG00000045038	Prkce	protein kinase C, epsilon	-1.12
ENSMUSG00000021565	Slc6a19	solute carrier family 6 (neurotransmitter transporter), member 19	-1.12
ENSMUSG00000027219	Slc28a2	solute carrier family 28 (sodium-coupled nucleoside transporter), member 2	-1.12
ENSMUSG00000074604	Mgst2	microsomal glutathione S-transferase 2	-1.12
ENSMUSG00000021062	Rab15	RAB15, member RAS oncogene family	-1.12
ENSMUSG00000005107	Slc2a9	solute carrier family 2 (facilitated glucose transporter), member 9	-1.12
ENSMUSG00000097222	1010001N08Rik	RIKEN cDNA 1010001N08 gene	-1.12
ENSMUSG00000111375	Btbd8	BTB (POZ) domain containing 8	-1.12
ENSMUSG00000028519	Dab1	disabled 1	-1.12
ENSMUSG00000069805	Fbp1	fructose bisphosphatase 1	-1.12
ENSMUSG00000017453	Pipox	pipecolic acid oxidase	-1.12
ENSMUSG00000049134	Nrap	nebulin-related anchoring protein	-1.12
ENSMUSG00000103159	F830112A20Rik	RIKEN cDNA F830112A20 gene	-1.12
ENSMUSG00000059540	Tcea2	transcription elongation factor A (SII), 2	-1.12
ENSMUSG00000027843	Ptpn22	protein tyrosine phosphatase, non-receptor type 22 (lymphoid)	-1.12
ENSMUSG00000040147	Maob	monoamine oxidase B	-1.12
ENSMUSG00000020573	Pik3cg	phosphatidylinositol-4,5-bisphosphate 3-kinase catalytic subunit gamma	-1.12
ENSMUSG00000041117	Ccdc8	coiled-coil domain containing 8	-1.12
ENSMUSG00000024901	Peli3	pellino 3	-1.12
ENSMUSG00000104943	Gm42868	predicted gene 42868	-1.12
ENSMUSG00000107878	Gm44106	predicted gene, 44106	-1.12
ENSMUSG00000038418	Egr1	early growth response 1	-1.13
ENSMUSG00000035506	Slc12a8	solute carrier family 12 (potassium/chloride transporters), member 8	-1.13
ENSMUSG00000065954	Tacc1	transforming, acidic coiled-coil containing protein 1	-1.13
ENSMUSG00000025408	Ddit3	DNA-damage inducible transcript 3	-1.13
ENSMUSG00000006342	Susd2	sushi domain containing 2	-1.13
ENSMUSG00000035919	Bbs9	Bardet-Biedl syndrome 9 (human)	-1.13
ENSMUSG00000074622	Mafb	v-maf musculoaponeurotic fibrosarcoma oncogene family, protein B (avian)	-1.13
ENSMUSG00000077167	Gm24119	predicted gene, 24119	-1.13
ENSMUSG00000110803	Gm20275	predicted gene, 20275	-1.13
ENSMUSG00000069713	4933406P04Rik	RIKEN cDNA 4933406P04 gene	-1.13
ENSMUSG00000030306	Tmtc1	transmembrane and tetratricopeptide repeat containing 1	-1.13
ENSMUSG00000052125	F730043M19Rik	RIKEN cDNA F730043M19 gene	-1.13
ENSMUSG00000104211	Gm37985	predicted gene, 37985	-1.13
ENSMUSG00000022564	Grina	glutamate receptor, ionotropic, N-methyl D-aspartate-associated protein 1 (glutam	-1.14
ENSMUSG00000028207	Asph	aspartate-beta-hydroxylase	-1.14
ENSMUSG00000037280	Galnt6	polypeptide N-acetylgalactosaminyltransferase 6	-1.14
ENSMUSG00000022750	Klhl22	kelch-like 22	-1.14
ENSMUSG00000036745	Ttll7	tubulin tyrosine ligase-like family, member 7	-1.14
ENSMUSG00000029811	Aoc1	amine oxidase, copper-containing 1	-1.14
ENSMUSG00000022574	Naprt	nicotinate phosphoribosyltransferase	-1.14
ENSMUSG00000067813	Xkr9	X-linked Kx blood group related 9	-1.14
ENSMUSG00000040389	Wdr47	WD repeat domain 47	-1.14
ENSMUSG00000001156	Mxd1	MAX dimerization protein 1	-1.14
ENSMUSG00000035121	Neil2	nei like 2 (E. coli)	-1.14
ENSMUSG00000110170	St6galnac2	ST6 (alpha-N-acetyl-neuraminyl-2,3-beta-galactosyl-1,3)-N-acetylgalactosaminide a	-1.14
ENSMUSG00000059824	Dbp	D site albumin promoter binding protein	-1.14
ENSMUSG00000112276	5033421B08Rik	RIKEN cDNA 5033421B08 gene	-1.14
ENSMUSG00000098439	Hm629797	cDNA sequence HM629797	-1.14
ENSMUSG00000081245	Gm6587	predicted gene 6587	-1.14
ENSMUSG00000041180	Hectd2	HECT domain E3 ubiquitin protein ligase 2	-1.14

ENSMUSG00000042066	Tmcc2	transmembrane and coiled-coil domains 2	-1.14
ENSMUSG00000034377	Tulp4	tubby like protein 4	-1.15
ENSMUSG00000026589	Sec16b	SEC16 homolog B (S. cerevisiae)	-1.15
ENSMUSG00000019970	Sgk1	serum/glucocorticoid regulated kinase 1	-1.15
ENSMUSG00000031378	Abcd1	ATP-binding cassette, sub-family D (ALD), member 1	-1.15
ENSMUSG00000041695	Kcnj2	potassium inwardly-rectifying channel, subfamily J, member 2	-1.15
ENSMUSG00000021620	Acot12	acyl-CoA thioesterase 12	-1.15
ENSMUSG00000108460	Gm39041	predicted gene, 39041	-1.15
ENSMUSG00000074196	Clca4c-ps	chloride channel accessory 4C, pseudogene	-1.15
ENSMUSG00000024885	Aldh3b1	aldehyde dehydrogenase 3 family, member B1	-1.15
ENSMUSG00000025064	Col17a1	collagen, type XVII, alpha 1	-1.15
ENSMUSG00000040441	Slc26a10	solute carrier family 26, member 10	-1.15
ENSMUSG00000062064	Slc2a7	solute carrier family 2 (facilitated glucose transporter), member 7	-1.15
ENSMUSG00000033847	Pla2g4c	phospholipase A2, group IVC (cytosolic, calcium-independent)	-1.15
ENSMUSG00000112592	Gm19972	predicted gene, 19972	-1.15
ENSMUSG00000045062	Pcdhb7	protocadherin beta 7	-1.15
ENSMUSG00000038860	Garnl3	GTPase activating RANGAP domain-like 3	-1.15
ENSMUSG00000024451	Arap3	ArfGAP with RhoGAP domain, ankyrin repeat and PH domain 3	-1.15
ENSMUSG00000102967	Gm37946	predicted gene, 37946	-1.15
ENSMUSG00000022783	Spag6l	sperm associated antigen 6-like	-1.15
ENSMUSG00000108693	Gm45153	predicted gene 45153	-1.15
ENSMUSG00000070436	Serpinh1	serine (or cysteine) peptidase inhibitor, clade H, member 1	-1.15
ENSMUSG00000030838	Ush1c	USH1 protein network component harmonin	-1.16
ENSMUSG00000020600	Slc7a15	solute carrier family 7 (cationic amino acid transporter, y+ system), member 15	-1.16
ENSMUSG00000020681	Ace	angiotensin I converting enzyme (peptidyl-dipeptidase A) 1	-1.16
ENSMUSG00000020261	Slc36a1	solute carrier family 36 (proton/amino acid symporter), member 1	-1.16
ENSMUSG00000023960	Enpp5	ectonucleotide pyrophosphatase/phosphodiesterase 5	-1.16
ENSMUSG00000032454	Rbp2	retinol binding protein 2, cellular	-1.16
ENSMUSG00000033917	Gde1	glycerophosphodiester phosphodiesterase 1	-1.16
ENSMUSG00000032105	Pdzd3	PDZ domain containing 3	-1.16
ENSMUSG00000039878	Slc39a5	solute carrier family 39 (metal ion transporter), member 5	-1.16
ENSMUSG00000107109	Gm42571	predicted gene 42571	-1.16
ENSMUSG00000109585	Gm45358	predicted gene 45358	-1.16
ENSMUSG00000034450	Gulo	gulonolactone (L-) oxidase	-1.16
ENSMUSG00000029168	Dpysl5	dihydropyrimidinase-like 5	-1.16
ENSMUSG00000000253	Gmpr	guanosine monophosphate reductase	-1.16
ENSMUSG00000112955	Gm48889	predicted gene, 48889	-1.16
ENSMUSG00000085774	Gm13055	predicted gene 13055	-1.16
ENSMUSG00000113102	A930040O22Rik	RIKEN cDNA A930040O22 gene	-1.16
ENSMUSG00000029162	Khk	ketohexokinase	-1.17
ENSMUSG00000035847	Ids	iduronate 2-sulfatase	-1.17
ENSMUSG00000024131	Slc3a1	solute carrier family 3, member 1	-1.17
ENSMUSG00000043822	Adamtsl5	ADAMTS-like 5	-1.17
ENSMUSG00000079445	B3gnt7	UDP-GlcNAc:betaGal beta-1,3-N-acetylglucosaminyltransferase 7	-1.17
ENSMUSG00000055148	Klf2	Kruppel-like factor 2 (lung)	-1.17
ENSMUSG00000097277	2900076A07Rik	RIKEN cDNA 2900076A07 gene	-1.17
ENSMUSG00000102423	Gm37465	predicted gene, 37465	-1.17
ENSMUSG00000104693	Gm42941	predicted gene 42941	-1.17
ENSMUSG00000042770	Hebp1	heme binding protein 1	-1.17
ENSMUSG00000043760	Pkhd1	polycystic kidney and hepatic disease 1	-1.17
ENSMUSG00000051503	Gm6583	predicted gene 6583	-1.17
ENSMUSG00000084798	4930533B01Rik	RIKEN cDNA 4930533B01 gene	-1.17
ENSMUSG00000054822	1700041G16Rik	RIKEN cDNA 1700041G16 gene	-1.17
ENSMUSG00000113679	Gm10432	predicted gene 10432	-1.17
ENSMUSG00000079993	Gm11799	predicted gene 11799	-1.17
ENSMUSG00000039545	Flicr	Foxp3 regulating long intergenic noncoding RNA	-1.17
ENSMUSG00000100009	Gm7967	predicted gene 7967	-1.17
ENSMUSG00000048970	C1galt1c1	C1GALT1-specific chaperone 1	-1.18
ENSMUSG00000032120	C2cd2l	C2 calcium-dependent domain containing 2-like	-1.18
ENSMUSG00000025497	Cdhr5	cadherin-related family member 5	-1.18
ENSMUSG00000023886	Smoc2	SPARC related modular calcium binding 2	-1.18
ENSMUSG00000002228	Ppm1j	protein phosphatase 1J	-1.18
ENSMUSG00000037139	Myom3	myomesin family, member 3	-1.18
ENSMUSG00000042684	Npl	N-acetylneuraminate pyruvate lyase	-1.18
ENSMUSG00000022861	Dgkg	diacylglycerol kinase, gamma	-1.18
ENSMUSG00000106596	Gm42822	predicted gene 42822	-1.18
ENSMUSG00000044906	4930503L19Rik	RIKEN cDNA 4930503L19 gene	-1.18

ENSMUSG00000026969	Fam166a	family with sequence similarity 166, member A	-1.18
ENSMUSG00000032482	Cspg5	chondroitin sulfate proteoglycan 5	-1.18
ENSMUSG00000029830	Svopl	SV2 related protein homolog (rat)-like	-1.18
ENSMUSG00000036960	Clca2	chloride channel accessory 2	-1.18
ENSMUSG00000092268	Gm20540	predicted gene 20540	-1.18
ENSMUSG00000018672	Copz2	coatomer protein complex, subunit zeta 2	-1.18
ENSMUSG00000062542	Syt9	synaptotagmin IX	-1.18
ENSMUSG00000021070	Bdkrb2	bradykinin receptor, beta 2	-1.18
ENSMUSG00000090061	Nwd2	NACHT and WD repeat domain containing 2	-1.18
ENSMUSG00000020484	Xbp1	X-box binding protein 1	-1.19
ENSMUSG00000015094	Npdc1	neural proliferation, differentiation and control 1	-1.19
ENSMUSG00000019960	Dusp6	dual specificity phosphatase 6	-1.19
ENSMUSG00000091705	H2-Q2	histocompatibility 2, Q region locus 2	-1.19
ENSMUSG00000024313	Mep1b	meprin 1 beta	-1.19
ENSMUSG00000029802	Abcg2	ATP binding cassette subfamily G member 2 (Junior blood group)	-1.19
ENSMUSG00000032098	Treh	trehalase (brush-border membrane glycoprotein)	-1.19
ENSMUSG00000038751	Ptk6	PTK6 protein tyrosine kinase 6	-1.19
ENSMUSG00000035713	Usp35	ubiquitin specific peptidase 35	-1.19
ENSMUSG00000029630	Cyp3a25	cytochrome P450, family 3, subfamily a, polypeptide 25	-1.19
ENSMUSG00000027983	Cyp2u1	cytochrome P450, family 2, subfamily u, polypeptide 1	-1.19
ENSMUSG00000039084	Chad	chondroadherin	-1.19
ENSMUSG00000038074	Fkbp14	FK506 binding protein 14	-1.19
ENSMUSG00000025092	Hspa12a	heat shock protein 12A	-1.19
ENSMUSG00000105701	Gm42587	predicted gene 42587	-1.19
ENSMUSG00000029369	Afm	afamin	-1.19
ENSMUSG00000026211	Obsl1	obscurin-like 1	-1.19
ENSMUSG00000103785	Gm35025	predicted gene, 35025	-1.19
ENSMUSG00000085900	A930041C12Rik	RIKEN cDNA A930041C12 gene	-1.19
ENSMUSG00000003469	Phyhip	phytanoyl-CoA hydroxylase interacting protein	-1.19
ENSMUSG00000022708	Zbtb20	zinc finger and BTB domain containing 20	-1.20
ENSMUSG00000022844	Pdia5	protein disulfide isomerase associated 5	-1.20
ENSMUSG00000027792	Bche	butyrylcholinesterase	-1.20
ENSMUSG00000021238	Aldh6a1	aldehyde dehydrogenase family 6, subfamily A1	-1.20
ENSMUSG00000032842	Abcc10	ATP-binding cassette, sub-family C (CFTR/MRP), member 10	-1.20
ENSMUSG00000039620	Trmt9b	tRNA methyltransferase 9B	-1.20
ENSMUSG00000107171	Gm42572	predicted gene 42572	-1.20
ENSMUSG00000056133	Unc93a2	unc-93 homolog A2	-1.20
ENSMUSG00000057265	Bbof1	basal body orientation factor 1	-1.20
ENSMUSG00000102440	Pcdhga9	protocadherin gamma subfamily A, 9	-1.20
ENSMUSG00000085615	A330035P11Rik	RIKEN cDNA A330035P11 gene	-1.20
ENSMUSG00000040473	Cfap69	cilia and flagella associated protein 69	-1.20
ENSMUSG00000028681	Ptch2	patched 2	-1.20
ENSMUSG00000038700	Hoxb5	homeobox B5	-1.20
ENSMUSG00000014529	Tmbim7	transmembrane BAX inhibitor motif containing 7	-1.20
ENSMUSG00000060988	Galnt13	polypeptide N-acetylgalactosaminyltransferase 13	-1.20
ENSMUSG00000108489	Gm45025	predicted gene 45025	-1.20
ENSMUSG00000040963	Asgr2	asialoglycoprotein receptor 2	-1.20
ENSMUSG00000019564	Arid3a	AT rich interactive domain 3A (BRIGHT-like)	-1.21
ENSMUSG00000032741	Tpcn1	two pore channel 1	-1.21
ENSMUSG00000034918	Cdhr2	cadherin-related family member 2	-1.21
ENSMUSG00000052302	Tbc1d30	TBC1 domain family, member 30	-1.21
ENSMUSG00000070461	9230112E08Rik	RIKEN cDNA 9230112E08 gene	-1.21
ENSMUSG00000084911	Gm16185	predicted gene 16185	-1.21
ENSMUSG00000086844	B230206H07Rik	RIKEN cDNA B230206H07 gene	-1.21
ENSMUSG00000032081	Apoc3	apolipoprotein C-III	-1.21
ENSMUSG00000031972	Acta1	actin, alpha 1, skeletal muscle	-1.21
ENSMUSG00000104156	Gm38102	predicted gene, 38102	-1.21
ENSMUSG00000086199	Bcas3os1	breast carcinoma amplified sequence 3, opposite strand 1	-1.21
ENSMUSG00000089028	Gm25010	predicted gene, 25010	-1.21
ENSMUSG00000087143	A830082K12Rik	RIKEN cDNA A830082K12 gene	-1.21
ENSMUSG00000047495	Dlgap2	DLG associated protein 2	-1.21
ENSMUSG00000046447	Camk2n1	calcium/calmodulin-dependent protein kinase II inhibitor 1	-1.22
ENSMUSG00000035722	Abca7	ATP-binding cassette, sub-family A (ABC1), member 7	-1.22
ENSMUSG00000037685	Atp8a1	ATPase, aminophospholipid transporter (APLT), class I, type 8A, member 1	-1.22
ENSMUSG00000052512	Nav2	neuron navigator 2	-1.22
ENSMUSG00000073399	Trim40	tripartite motif-containing 40	-1.22
ENSMUSG00000026222	Sp100	nuclear antigen Sp100	-1.22

ENSMUSG00000029607	Ankrd61	ankyrin repeat domain 61	-1.22
ENSMUSG00000034687	Fras1	Fraser extracellular matrix complex subunit 1	-1.22
ENSMUSG00000019359	Gdpd2	glycerophosphodiester phosphodiesterase domain containing 2	-1.22
ENSMUSG00000030600	Lrfn1	leucine rich repeat and fibronectin type III domain containing 1	-1.22
ENSMUSG00000031766	Slc12a3	solute carrier family 12, member 3	-1.22
ENSMUSG00000007783	Cpt1c	carnitine palmitoyltransferase 1c	-1.22
ENSMUSG00000101462	Gm3052	predicted gene 3052	-1.22
ENSMUSG00000064413	Gm22245	predicted gene, 22245	-1.22
ENSMUSG00000110767	Gm31432	predicted gene, 31432	-1.22
ENSMUSG00000084052	Gm11745	predicted gene 11745	-1.22
ENSMUSG00000105344	Gm43556	predicted gene 43556	-1.22
ENSMUSG00000025469	Msx3	msh homeobox 3	-1.22
ENSMUSG00000025498	Irf7	interferon regulatory factor 7	-1.23
ENSMUSG00000026113	Inpp4a	inositol polyphosphate-4-phosphatase, type I	-1.23
ENSMUSG00000048249	Crebrf	CREB3 regulatory factor	-1.23
ENSMUSG00000044860	Gm1123	predicted gene 1123	-1.23
ENSMUSG00000024254	Abcg8	ATP binding cassette subfamily G member 8	-1.23
ENSMUSG00000029370	Rassf6	Ras association (RalGDS/AF-6) domain family member 6	-1.23
ENSMUSG00000026614	Slc30a10	solute carrier family 30, member 10	-1.23
ENSMUSG00000047641	Krt87	keratin 87	-1.23
ENSMUSG00000085747	Slc13a2os	solute carrier family 13 (sodium-dependent dicarboxylate transporter), member 2,	-1.23
ENSMUSG00000042010	Acacb	acetyl-Coenzyme A carboxylase beta	-1.23
ENSMUSG00000103693	Gm37529	predicted gene, 37529	-1.23
ENSMUSG00000048347	Pcdhb18	protocadherin beta 18	-1.23
ENSMUSG00000021268	Meg3	maternally expressed 3	-1.23
ENSMUSG00000022766	Serpind1	serine (or cysteine) peptidase inhibitor, clade D, member 1	-1.23
ENSMUSG00000111063	Zkscan7	zinc finger with KRAB and SCAN domains 7	-1.23
ENSMUSG00000113780	Gm33195	predicted gene, 33195	-1.23
ENSMUSG00000047033	Pcdhb15	protocadherin beta 15	-1.23
ENSMUSG00000030865	Chp2	calcineurin-like EF hand protein 2	-1.24
ENSMUSG00000050747	Trim15	tripartite motif-containing 15	-1.24
ENSMUSG00000025140	Pycr1	pyrroline-5-carboxylate reductase 1	-1.24
ENSMUSG00000073889	Il11ra1	interleukin 11 receptor, alpha chain 1	-1.24
ENSMUSG00000032289	Thsd4	thrombospondin, type I, domain containing 4	-1.24
ENSMUSG00000032036	Kirrel3	kirre like nephrin family adhesion molecule 3	-1.24
ENSMUSG00000103472	Pcdhga7	protocadherin gamma subfamily A, 7	-1.24
ENSMUSG00000103567	Pcdhga5	protocadherin gamma subfamily A, 5	-1.24
ENSMUSG00000113918	Gm6566	predicted gene 6566	-1.24
ENSMUSG00000086507	Adap2os	ArfGAP with dual PH domains 2, opposite strand	-1.24
ENSMUSG00000084890	A830036E02Rik	RIKEN cDNA A830036E02 gene	-1.24
ENSMUSG00000000392	Fap	fibroblast activation protein	-1.24
ENSMUSG00000086533	Mypopos	Myb-related transcription factor, partner of profilin, opposite strand	-1.24
ENSMUSG00000109780	Gm45447	predicted gene 45447	-1.24
ENSMUSG00000109869	Gm39158	predicted gene, 39158	-1.24
ENSMUSG00000021919	Chat	choline acetyltransferase	-1.24
ENSMUSG00000048562	Sp8	trans-acting transcription factor 8	-1.24
ENSMUSG00000106450	Gm43018	predicted gene 43018	-1.24
ENSMUSG00000108076	9530086O07Rik	RIKEN cDNA 9530086O07 gene	-1.24
ENSMUSG00000038894	Irs2	insulin receptor substrate 2	-1.25
ENSMUSG00000005580	Adcy9	adenylate cyclase 9	-1.25
ENSMUSG00000037822	Smim14	small integral membrane protein 14	-1.25
ENSMUSG00000078439	Smim24	small integral membrane protein 24	-1.25
ENSMUSG00000048473	Sult6b2	sulfotransferase family 6B, member 2	-1.25
ENSMUSG00000022540	Rogdi	rogdi homolog	-1.25
ENSMUSG00000043621	Ubxn10	UBX domain protein 10	-1.25
ENSMUSG00000094830	n-R5s194	nuclear encoded rRNA 5S 194	-1.25
ENSMUSG00000040432	Ltb4r2	leukotriene B4 receptor 2	-1.25
ENSMUSG00000107813	Gm44434	predicted gene, 44434	-1.25
ENSMUSG00000103432	6720464F23Rik	RIKEN cDNA 6720464F23 gene	-1.25
ENSMUSG00000055493	Epm2a	epilepsy, progressive myoclonic epilepsy, type 2 gene alpha	-1.25
ENSMUSG00000073680	Tmem88b	transmembrane protein 88B	-1.25
ENSMUSG00000038143	Stox2	storkhead box 2	-1.25
ENSMUSG00000113041	Gm48302	predicted gene, 48302	-1.25
ENSMUSG00000113852	Gm48062	predicted gene, 48062	-1.25
ENSMUSG00000033768	Nrxn2	neurexin II	-1.25
ENSMUSG00000038576	Susd4	sushi domain containing 4	-1.25
ENSMUSG00000032083	Apoa1	apolipoprotein A-I	-1.26

ENSMUSG00000028015	Ctso	cathepsin O	-1.26
ENSMUSG00000032596	Uba7	ubiquitin-like modifier activating enzyme 7	-1.26
ENSMUSG00000033147	Slc22a15	solute carrier family 22 (organic anion/cation transporter), member 15	-1.26
ENSMUSG00000036473	Tbc1d24	TBC1 domain family, member 24	-1.26
ENSMUSG00000033987	Dnah17	dynein, axonemal, heavy chain 17	-1.26
ENSMUSG00000074653	Lrrc31	leucine rich repeat containing 31	-1.26
ENSMUSG00000051043	Gprc5c	G protein-coupled receptor, family C, group 5, member C	-1.26
ENSMUSG00000033721	Vav3	vav 3 oncogene	-1.26
ENSMUSG00000097443	Gm17529	predicted gene, 17529	-1.26
ENSMUSG00000034579	Pla2g3	phospholipase A2, group III	-1.26
ENSMUSG00000073403	Gm10499	predicted gene 10499	-1.26
ENSMUSG00000054477	Kcnn2	potassium intermediate/small conductance calcium-activated channel, subfamily N	-1.26
ENSMUSG00000052767	Gm12703	predicted gene 12703	-1.26
ENSMUSG00000113370	Gm46348	predicted gene, 46348	-1.26
ENSMUSG00000110366	Gm45885	predicted gene 45885	-1.26
ENSMUSG00000025207	Sema4g	sema domain, immunoglobulin domain (Ig), transmembrane domain (TM) and shor	-1.27
ENSMUSG00000022546	Gpt	glutamic pyruvic transaminase, soluble	-1.27
ENSMUSG00000032038	St3gal4	ST3 beta-galactoside alpha-2,3-sialyltransferase 4	-1.27
ENSMUSG00000032652	Crebl2	cAMP responsive element binding protein-like 2	-1.27
ENSMUSG00000056602	Fry	FRY microtubule binding protein	-1.27
ENSMUSG00000027513	Pck1	phosphoenolpyruvate carboxykinase 1, cytosolic	-1.27
ENSMUSG00000108291	Gm44292	predicted gene, 44292	-1.27
ENSMUSG00000094377	Gm24407	predicted gene, 24407	-1.27
ENSMUSG00000074261	Erich4	glutamate rich 4	-1.27
ENSMUSG00000028427	Aqp7	aquaporin 7	-1.27
ENSMUSG00000048895	Cdk5r1	cyclin-dependent kinase 5, regulatory subunit 1 (p35)	-1.27
ENSMUSG00000032420	Nt5e	5' nucleotidase, ecto	-1.27
ENSMUSG00000098269	Mir8094	microRNA 8094	-1.27
ENSMUSG00000085903	Gm15340	predicted gene 15340	-1.27
ENSMUSG00000042532	Golga7b	golgi autoantigen, golgin subfamily a, 7B	-1.27
ENSMUSG00000102466	Gm38200	predicted gene, 38200	-1.27
ENSMUSG00000103897	Pcdhga8	protocadherin gamma subfamily A, 8	-1.27
ENSMUSG00000108075	Gm45061	predicted gene 45061	-1.27
ENSMUSG00000041481	Serpina3g	serine (or cysteine) peptidase inhibitor, clade A, member 3G	-1.27
ENSMUSG00000086708	Gm15577	predicted gene 15577	-1.27
ENSMUSG00000106516	Gm42441	predicted gene 42441	-1.27
ENSMUSG00000113535	AU015791	expressed sequence AU015791	-1.27
ENSMUSG00000029992	Gfpt1	glutamine fructose-6-phosphate transaminase 1	-1.28
ENSMUSG00000092341	Malat1	metastasis associated lung adenocarcinoma transcript 1 (non-coding RNA)	-1.28
ENSMUSG00000060002	Chpt1	choline phosphotransferase 1	-1.28
ENSMUSG00000004655	Aqp1	aquaporin 1	-1.28
ENSMUSG00000006435	Neurl1a	neuralized E3 ubiquitin protein ligase 1A	-1.28
ENSMUSG00000028017	Egf	epidermal growth factor	-1.28
ENSMUSG00000029163	Emilin1	elastin microfibril interfacer 1	-1.28
ENSMUSG00000079355	Ackr4	atypical chemokine receptor 4	-1.28
ENSMUSG00000085925	Rtl1	retrotransposon Gaglike 1	-1.28
ENSMUSG00000009654	Oit3	oncoprotein induced transcript 3	-1.28
ENSMUSG00000038522	Mfsd4b1	major facilitator superfamily domain containing 4B1	-1.28
ENSMUSG00000020169	Best3	bestrophin 3	-1.28
ENSMUSG00000052469	Tcp10c	t-complex protein 10c	-1.28
ENSMUSG00000038305	Spats2l	spermatogenesis associated, serine-rich 2-like	-1.29
ENSMUSG00000005373	Mlxipl	MLX interacting protein-like	-1.29
ENSMUSG00000013275	Slc41a1	solute carrier family 41, member 1	-1.29
ENSMUSG00000102964	9430034N14Rik	RIKEN cDNA 9430034N14 gene	-1.29
ENSMUSG00000051606	2010001K21Rik	RIKEN cDNA 2010001K21 gene	-1.29
ENSMUSG00000110068	Gm45294	predicted gene 45294	-1.29
ENSMUSG00000040794	C1qtnf4	C1q and tumor necrosis factor related protein 4	-1.29
ENSMUSG00000021725	Parp8	poly (ADP-ribose) polymerase family, member 8	-1.29
ENSMUSG00000087530	Gm15533	predicted gene 15533	-1.29
ENSMUSG00000112234	Gm48427	predicted gene, 48427	-1.29
ENSMUSG00000040265	Dnm3	dynamin 3	-1.29
ENSMUSG00000105470	Gm42589	predicted gene 42589	-1.29
ENSMUSG00000112462	Gm48772	predicted gene, 48772	-1.29
ENSMUSG00000028777	Gnat3	guanine nucleotide binding protein, alpha transducing 3	-1.29
ENSMUSG00000026807	Ak8	adenylate kinase 8	-1.29
ENSMUSG00000021190	Lgmn	legumain	-1.30
ENSMUSG00000023045	Soat2	sterol O-acyltransferase 2	-1.30

ENSMUSG00000040270	Bach2	BTB and CNC homology, basic leucine zipper transcription factor 2	-1.30
ENSMUSG00000066258	Trim12a	tripartite motif-containing 12A	-1.30
ENSMUSG00000040721	Zfhx2	zinc finger homeobox 2	-1.30
ENSMUSG00000059708	Akap17b	A kinase (PRKA) anchor protein 17B	-1.30
ENSMUSG00000038058	Nod1	nucleotide-binding oligomerization domain containing 1	-1.30
ENSMUSG00000037940	Inpp4b	inositol polyphosphate-4-phosphatase, type II	-1.30
ENSMUSG00000110187	Gm45496	predicted gene 45496	-1.30
ENSMUSG00000068600	Gml2	glycosylphosphatidylinositol anchored molecule like 2	-1.30
ENSMUSG00000085884	Gm15342	predicted gene 15342	-1.30
ENSMUSG00000084329	Gm6733	predicted gene 6733	-1.30
ENSMUSG00000116180	Gm49492	predicted gene, 49492	-1.30
ENSMUSG00000036437	Npy1r	neuropeptide Y receptor Y1	-1.30
ENSMUSG00000083603	Gm14810	predicted gene 14810	-1.30
ENSMUSG00000014905	Dnajb9	DnaJ heat shock protein family (Hsp40) member B9	-1.31
ENSMUSG00000030017	Reg3g	regenerating islet-derived 3 gamma	-1.31
ENSMUSG00000031570	Plpp5	phospholipid phosphatase 5	-1.31
ENSMUSG00000034449	Dhrs11	dehydrogenase/reductase (SDR family) member 11	-1.31
ENSMUSG00000004952	Rasa4	RAS p21 protein activator 4	-1.31
ENSMUSG00000053334	Ficd	FIC domain containing	-1.31
ENSMUSG00000095915	n-R5s115	nuclear encoded rRNA 5S 115	-1.31
ENSMUSG00000023092	Fhl1	four and a half LIM domains 1	-1.31
ENSMUSG00000097147	Gm26780	predicted gene, 26780	-1.31
ENSMUSG00000019737	Syne4	spectrin repeat containing, nuclear envelope family member 4	-1.31
ENSMUSG00000047115	Fam221a	family with sequence similarity 221, member A	-1.31
ENSMUSG00000052783	Grk4	G protein-coupled receptor kinase 4	-1.31
ENSMUSG00000029195	Klb	klotho beta	-1.31
ENSMUSG00000062017	Abca14	ATP-binding cassette, sub-family A (ABC1), member 14	-1.31
ENSMUSG00000113811	Gm47882	predicted gene, 47882	-1.31
ENSMUSG00000036995	Asap3	ArfGAP with SH3 domain, ankyrin repeat and PH domain 3	-1.31
ENSMUSG00000108246	Gm43896	predicted gene, 43896	-1.31
ENSMUSG00000096549	Prickle4	prickle planar cell polarity protein 4	-1.31
ENSMUSG00000026070	Il18r1	interleukin 18 receptor 1	-1.31
ENSMUSG00000028185	Dnase2b	deoxyribonuclease II beta	-1.31
ENSMUSG00000036587	Fut7	fucosyltransferase 7	-1.31
ENSMUSG00000102404	5530400K19Rik	RIKEN cDNA 5530400K19 gene	-1.31
ENSMUSG00000026825	Dnm1	dynamin 1	-1.32
ENSMUSG00000019066	Rab3d	RAB3D, member RAS oncogene family	-1.32
ENSMUSG00000027790	Sis	sucrase isomaltase (alpha-glucosidase)	-1.32
ENSMUSG00000015944	Castor2	cytosolic arginine sensor for mTORC1 subunit 2	-1.32
ENSMUSG00000002504	Slc9a3r2	solute carrier family 9 (sodium/hydrogen exchanger), member 3 regulator 2	-1.32
ENSMUSG00000079516	Reg3a	regenerating islet-derived 3 alpha	-1.32
ENSMUSG00000031155	Pim2	proviral integration site 2	-1.32
ENSMUSG00000046387	Pcdhb17	protocadherin beta 17	-1.32
ENSMUSG00000036298	Slc2a13	solute carrier family 2 (facilitated glucose transporter), member 13	-1.32
ENSMUSG00000046191	Pcdhb20	protocadherin beta 20	-1.32
ENSMUSG00000032656	37681	membrane-associated ring finger (C3HC4) 3	-1.32
ENSMUSG00000049246	Hspe1-ps2	heat shock protein 1 (chaperonin 10), pseudogene 2	-1.32
ENSMUSG00000096039	D830030K20Rik	RIKEN cDNA D830030K20 gene	-1.32
ENSMUSG00000031230	Fgf16	fibroblast growth factor 16	-1.32
ENSMUSG00000035561	Aldh1b1	aldehyde dehydrogenase 1 family, member B1	-1.33
ENSMUSG00000023951	Vegfa	vascular endothelial growth factor A	-1.33
ENSMUSG00000053054	Adh6a	alcohol dehydrogenase 6A (class V)	-1.33
ENSMUSG00000042675	Ypel3	yippee like 3	-1.33
ENSMUSG00000046546	Fam43a	family with sequence similarity 43, member A	-1.33
ENSMUSG00000020709	Adap2	ArfGAP with dual PH domains 2	-1.33
ENSMUSG00000022197	Pdzd2	PDZ domain containing 2	-1.33
ENSMUSG00000052135	Foxo6	forkhead box O6	-1.33
ENSMUSG00000000402	Egfl6	EGF-like-domain, multiple 6	-1.33
ENSMUSG00000079499	6530402F18Rik	RIKEN cDNA 6530402F18 gene	-1.33
ENSMUSG00000033029	1700088E04Rik	RIKEN cDNA 1700088E04 gene	-1.33
ENSMUSG00000112338	Gm47693	predicted gene, 47693	-1.33
ENSMUSG00000024965	Fermt3	fermitin family member 3	-1.33
ENSMUSG00000025083	Afap1l2	actin filament associated protein 1-like 2	-1.33
ENSMUSG00000112596	Gm48804	predicted gene, 48804	-1.33
ENSMUSG00000084137	Gm9085	predicted gene 9085	-1.33
ENSMUSG00000035279	Ssc5d	scavenger receptor cysteine rich family, 5 domains	-1.33
ENSMUSG00000078451	Ppil6	peptidylprolyl isomerase (cyclophilin)-like 6	-1.33

ENSMUSG00000073242	Dnmt3aos	DNA methyltransferase 3A, opposite strand	-1.33
ENSMUSG00000052188	Gm14964	predicted gene 14964	-1.33
ENSMUSG00000001334	Fndc5	fibronectin type III domain containing 5	-1.33
ENSMUSG00000041642	Kif21b	kinesin family member 21B	-1.34
ENSMUSG00000062995	Ica1	islet cell autoantigen 1	-1.34
ENSMUSG00000032776	Mctp2	multiple C2 domains, transmembrane 2	-1.34
ENSMUSG00000042428	Mgat3	mannoside acetylglucosaminyltransferase 3	-1.34
ENSMUSG00000020447	Npc1l1	NPC1 like intracellular cholesterol transporter 1	-1.34
ENSMUSG00000026610	Esrrg	estrogen-related receptor gamma	-1.34
ENSMUSG00000035493	Tgfbi	transforming growth factor, beta induced	-1.34
ENSMUSG00000020614	Fam20a	family with sequence similarity 20, member A	-1.34
ENSMUSG00000045094	Arhgef37	Rho guanine nucleotide exchange factor (GEF) 37	-1.34
ENSMUSG00000042312	S100a13	S100 calcium binding protein A13	-1.34
ENSMUSG00000101365	Gm19325	predicted gene, 19325	-1.34
ENSMUSG00000086189	Gm15462	predicted gene 15462	-1.34
ENSMUSG00000047227	Gm527	predicted gene 527	-1.34
ENSMUSG00000107094	Gm43294	predicted gene 43294	-1.34
ENSMUSG00000104020	Gm37215	predicted gene, 37215	-1.34
ENSMUSG00000043719	Col6a6	collagen, type VI, alpha 6	-1.34
ENSMUSG00000113203	Gm47587	predicted gene, 47587	-1.34
ENSMUSG00000032899	Styk1	serine/threonine/tyrosine kinase 1	-1.35
ENSMUSG00000038312	Edem2	ER degradation enhancer, mannosidase alpha-like 2	-1.35
ENSMUSG00000042632	Pla2g6	phospholipase A2, group VI	-1.35
ENSMUSG00000028943	Espn	espin	-1.35
ENSMUSG00000034312	Iqsec1	IQ motif and Sec7 domain 1	-1.35
ENSMUSG00000058921	Slc10a5	solute carrier family 10 (sodium/bile acid cotransporter family), member 5	-1.35
ENSMUSG00000030657	Xylt1	xylosyltransferase 1	-1.35
ENSMUSG00000051427	Ccdc157	coiled-coil domain containing 157	-1.35
ENSMUSG00000056025	Clca3a1	chloride channel accessory 3A1	-1.35
ENSMUSG00000033066	Gas7	growth arrest specific 7	-1.35
ENSMUSG00000113119	Gm48883	predicted gene, 48883	-1.35
ENSMUSG00000049556	Lingo1	leucine rich repeat and Ig domain containing 1	-1.35
ENSMUSG00000063564	Col23a1	collagen, type XXIII, alpha 1	-1.35
ENSMUSG00000020793	Galr2	galanin receptor 2	-1.35
ENSMUSG00000110357	A030001D20Rik	RIKEN cDNA A030001D20 gene	-1.35
ENSMUSG00000038311	2410017I17Rik	RIKEN cDNA 2410017I17 gene	-1.35
ENSMUSG00000028782	Adgrb2	adhesion G protein-coupled receptor B2	-1.35
ENSMUSG00000041559	Fmod	fibromodulin	-1.35
ENSMUSG00000082207	Gm12998	predicted gene 12998	-1.35
ENSMUSG00000040929	Rfx3	regulatory factor X, 3 (influences HLA class II expression)	-1.36
ENSMUSG00000024713	Pcsk5	proprotein convertase subtilisin/kexin type 5	-1.36
ENSMUSG00000053862	Slc51b	solute carrier family 51, beta subunit	-1.36
ENSMUSG00000055730	Ces2a	carboxylesterase 2A	-1.36
ENSMUSG00000044626	Liph	lipase, member H	-1.36
ENSMUSG00000008999	Bmp7	bone morphogenetic protein 7	-1.36
ENSMUSG00000068735	Trp53i11	transformation related protein 53 inducible protein 11	-1.36
ENSMUSG00000102732	Gm37342	predicted gene, 37342	-1.36
ENSMUSG00000023467	Tulp2	tubby-like protein 2	-1.36
ENSMUSG00000116506	5730414N17Rik	RIKEN cDNA 5730414N17 gene	-1.36
ENSMUSG00000093580	Gm20706	predicted gene 20706	-1.36
ENSMUSG00000114302	Gm48683	predicted gene, 48683	-1.36
ENSMUSG00000021250	Fos	FBJ osteosarcoma oncogene	-1.37
ENSMUSG00000052085	Dock8	dedicator of cytokinesis 8	-1.37
ENSMUSG00000037005	Xpnpep2	X-prolyl aminopeptidase (aminopeptidase P) 2, membrane-bound	-1.37
ENSMUSG00000028051	Hcn3	hyperpolarization-activated, cyclic nucleotide-gated K+ 3	-1.37
ENSMUSG00000032839	Trpc1	transient receptor potential cation channel, subfamily C, member 1	-1.37
ENSMUSG00000075270	Pde11a	phosphodiesterase 11A	-1.37
ENSMUSG00000022686	B3gnt5	UDP-GlcNAc:betaGal beta-1,3-N-acetylglucosaminyltransferase 5	-1.37
ENSMUSG00000003282	Plag1	pleiomorphic adenoma gene 1	-1.37
ENSMUSG00000107197	Gm43312	predicted gene 43312	-1.37
ENSMUSG00000075014	Gm10800	predicted gene 10800	-1.37
ENSMUSG00000041729	Coro2b	coronin, actin binding protein, 2B	-1.37
ENSMUSG00000084744	Gm25291	predicted gene, 25291	-1.37
ENSMUSG00000063929	Cyp4a32	cytochrome P450, family 4, subfamily a, polypeptide 32	-1.37
ENSMUSG00000087341	0610040F04Rik	RIKEN cDNA 0610040F04 gene	-1.37
ENSMUSG00000042800	Spata46	spermatogenesis associated 46	-1.37
ENSMUSG00000052889	Prkcb	protein kinase C, beta	-1.37

ENSMUSG00000038540	Tmc3	transmembrane channel-like gene family 3	-1.37
ENSMUSG00000104343	5730408A14Rik	RIKEN cDNA 5730408A14 gene	-1.37
ENSMUSG00000105975	Gm42609	predicted gene 42609	-1.37
ENSMUSG00000032564	Cpne4	copine IV	-1.37
ENSMUSG00000019789	Hey2	hairy/enhancer-of-split related with YRPW motif 2	-1.37
ENSMUSG00000062991	Nrg1	neuregulin 1	-1.37
ENSMUSG00000023259	Slc26a6	solute carrier family 26, member 6	-1.38
ENSMUSG00000020080	Hkdc1	hexokinase domain containing 1	-1.38
ENSMUSG00000024340	Btnl2	butyrophilin-like 2	-1.38
ENSMUSG00000073420	Btnl5-ps	butyrophilin-like 5, pseudogene	-1.38
ENSMUSG00000003032	Klf4	Kruppel-like factor 4 (gut)	-1.38
ENSMUSG00000052353	Cemip	cell migration inducing protein, hyaluronan binding	-1.38
ENSMUSG00000037921	Ddx60	DEAD (Asp-Glu-Ala-Asp) box polypeptide 60	-1.38
ENSMUSG00000096349	Gm22513	predicted gene, 22513	-1.38
ENSMUSG00000000296	Tpd52l1	tumor protein D52-like 1	-1.38
ENSMUSG00000025268	Maged2	melanoma antigen, family D, 2	-1.38
ENSMUSG00000045312	Lhfpl2	lipoma HMGIC fusion partner-like 2	-1.38
ENSMUSG00000068323	Slc4a5	solute carrier family 4, sodium bicarbonate cotransporter, member 5	-1.38
ENSMUSG00000020604	Arsg	arylsulfatase G	-1.38
ENSMUSG00000038541	Srd5a2	steroid 5 alpha-reductase 2	-1.38
ENSMUSG00000018620	Mmp20	matrix metallopeptidase 20 (enamelysin)	-1.38
ENSMUSG00000103793	Pcdhga6	protocadherin gamma subfamily A, 6	-1.38
ENSMUSG00000009092	Derl3	Der1-like domain family, member 3	-1.38
ENSMUSG00000006784	Ttc25	tetratricopeptide repeat domain 25	-1.38
ENSMUSG00000102224	4930447F24Rik	RIKEN cDNA 4930447F24 gene	-1.38
ENSMUSG00000021101	4930408O17Rik	RIKEN cDNA 4930408O17 gene	-1.38
ENSMUSG00000045518	Onecut3	one cut domain, family member 3	-1.38
ENSMUSG00000027634	Ndrg3	N-myc downstream regulated gene 3	-1.39
ENSMUSG00000028836	Slc30a2	solute carrier family 30 (zinc transporter), member 2	-1.39
ENSMUSG00000034981	Parm1	prostate androgen-regulated mucin-like protein 1	-1.39
ENSMUSG00000078612	Fyb2	FYN binding protein 2	-1.39
ENSMUSG00000034614	Pik3ip1	phosphoinositide-3-kinase interacting protein 1	-1.39
ENSMUSG00000061462	Obscn	obscurin, cytoskeletal calmodulin and titin-interacting RhoGEF	-1.39
ENSMUSG00000033389	Arhgap44	Rho GTPase activating protein 44	-1.39
ENSMUSG00000114576	Naip3-ps1	NLR family, apoptosis inhibitory protein 3, pseudogene 1	-1.39
ENSMUSG00000032679	Cd59a	CD59a antigen	-1.39
ENSMUSG00000116262	Gm49544	predicted gene, 49544	-1.39
ENSMUSG00000039543	Cfap70	cilia and flagella associated protein 70	-1.39
ENSMUSG00000086258	Gm15239	predicted gene 15239	-1.39
ENSMUSG00000097911	Gm26691	predicted gene, 26691	-1.39
ENSMUSG00000048540	Nhlh2	nescient helix loop helix 2	-1.39
ENSMUSG00000026815	Gfi1b	growth factor independent 1B	-1.39
ENSMUSG00000025006	Sorbs1	sorbin and SH3 domain containing 1	-1.40
ENSMUSG00000031618	Nr3c2	nuclear receptor subfamily 3, group C, member 2	-1.40
ENSMUSG00000014773	Dll1	delta like canonical Notch ligand 1	-1.40
ENSMUSG00000035390	Brsk1	BR serine/threonine kinase 1	-1.40
ENSMUSG00000042589	Cux2	cut-like homeobox 2	-1.40
ENSMUSG00000060224	Pyroxd2	pyridine nucleotide-disulphide oxidoreductase domain 2	-1.40
ENSMUSG00000063239	Grm4	glutamate receptor, metabotropic 4	-1.40
ENSMUSG00000022340	Sybu	syntabulin (syntaxin-interacting)	-1.40
ENSMUSG00000002633	Shh	sonic hedgehog	-1.40
ENSMUSG00000036611	Eepd1	endonuclease/exonuclease/phosphatase family domain containing 1	-1.41
ENSMUSG00000022048	Dpysl2	dihydropyrimidinase-like 2	-1.41
ENSMUSG00000038473	Nos1ap	nitric oxide synthase 1 (neuronal) adaptor protein	-1.41
ENSMUSG00000030659	Nucb2	nucleobindin 2	-1.41
ENSMUSG00000057880	Abat	4-aminobutyrate aminotransferase	-1.41
ENSMUSG00000041420	Meis3	Meis homeobox 3	-1.41
ENSMUSG00000020882	Cacnb1	calcium channel, voltage-dependent, beta 1 subunit	-1.41
ENSMUSG00000073733	Rsg1	REM2 and RAB-like small GTPase 1	-1.41
ENSMUSG00000005986	Ankrd13d	ankyrin repeat domain 13 family, member D	-1.41
ENSMUSG00000105873	Gm43708	predicted gene 43708	-1.41
ENSMUSG00000020715	Ern1	endoplasmic reticulum (ER) to nucleus signalling 1	-1.42
ENSMUSG00000038967	Pdk2	pyruvate dehydrogenase kinase, isoenzyme 2	-1.42
ENSMUSG00000054708	Ankrd24	ankyrin repeat domain 24	-1.42
ENSMUSG00000047496	Rnf152	ring finger protein 152	-1.42
ENSMUSG00000040181	Fmo1	flavin containing monooxygenase 1	-1.42
ENSMUSG00000026452	Syt2	synaptotagmin II	-1.42

ENSMUSG00000028750	Pla2g2c	phospholipase A2, group IIC	-1.42
ENSMUSG00000086391	1700042O10Rik	RIKEN cDNA 1700042O10 gene	-1.42
ENSMUSG00000106384	Gm43188	predicted gene 43188	-1.42
ENSMUSG00000074439	Defa5	defensin, alpha, 5	-1.42
ENSMUSG00000028167	Bdh2	3-hydroxybutyrate dehydrogenase, type 2	-1.42
ENSMUSG00000036452	Arhgap26	Rho GTPase activating protein 26	-1.43
ENSMUSG00000028150	Rorc	RAR-related orphan receptor gamma	-1.43
ENSMUSG00000110060	Gm9860	predicted gene 9860	-1.43
ENSMUSG00000061728	Btnl7-ps	butyrophilin-like 7, pseudogene	-1.43
ENSMUSG00000073406	H2-Bl	histocompatibility 2, blastocyst	-1.43
ENSMUSG00000103194	Gm37643	predicted gene, 37643	-1.43
ENSMUSG00000104351	Gm37125	predicted gene, 37125	-1.43
ENSMUSG00000113966	Gm47980	predicted gene, 47980	-1.43
ENSMUSG00000090214	Gm15657	predicted gene 15657	-1.44
ENSMUSG00000092618	Btnl6	butyrophilin-like 6	-1.44
ENSMUSG00000021208	Ifi27l2b	interferon, alpha-inducible protein 27 like 2B	-1.44
ENSMUSG00000048027	Rgmb	repulsive guidance molecule family member B	-1.44
ENSMUSG00000026347	Tmem163	transmembrane protein 163	-1.44
ENSMUSG00000038042	Ptpdc1	protein tyrosine phosphatase domain containing 1	-1.44
ENSMUSG00000111271	Gm48127	predicted gene, 48127	-1.44
ENSMUSG00000079157	Fam155a	family with sequence similarity 155, member A	-1.44
ENSMUSG00000022946	Dopey2	dopey family member 2	-1.45
ENSMUSG00000022026	Olfm4	olfactomedin 4	-1.45
ENSMUSG00000026131	Dst	dystonin	-1.45
ENSMUSG00000035041	Creb3l3	cAMP responsive element binding protein 3-like 3	-1.45
ENSMUSG00000040505	Abcg5	ATP binding cassette subfamily G member 5	-1.45
ENSMUSG00000029273	Sult1d1	sulfotransferase family 1D, member 1	-1.45
ENSMUSG00000031613	Hpgd	hydroxyprostaglandin dehydrogenase 15 (NAD)	-1.45
ENSMUSG00000034570	Inpp5j	inositol polyphosphate 5-phosphatase J	-1.45
ENSMUSG00000094651	Gal3st2	galactose-3-O-sulfotransferase 2	-1.45
ENSMUSG00000029650	Slc46a3	solute carrier family 46, member 3	-1.45
ENSMUSG00000051146	Camk2n2	calcium/calmodulin-dependent protein kinase II inhibitor 2	-1.45
ENSMUSG00000045348	Nyap1	neuronal tyrosine-phosphorylated phosphoinositide 3-kinase adaptor 1	-1.45
ENSMUSG00000011256	Adam19	a disintegrin and metallopeptidase domain 19 (meltrin beta)	-1.45
ENSMUSG00000089874	9230117E06Rik	RIKEN cDNA 9230117E06 gene	-1.45
ENSMUSG00000015243	Abca1	ATP-binding cassette, sub-family A (ABC1), member 1	-1.46
ENSMUSG00000066621	Tecpr1	tectonin beta-propeller repeat containing 1	-1.46
ENSMUSG00000037390	Muc3	mucin 3, intestinal	-1.46
ENSMUSG00000043079	Synpo	synaptopodin	-1.46
ENSMUSG00000048572	Tmem252	transmembrane protein 252	-1.46
ENSMUSG00000025082	Vwa2	von Willebrand factor A domain containing 2	-1.46
ENSMUSG00000027805	Pfn2	profilin 2	-1.46
ENSMUSG00000037493	Cib2	calcium and integrin binding family member 2	-1.46
ENSMUSG00000108476	Gm44974	predicted gene 44974	-1.46
ENSMUSG00000074115	Saa1	serum amyloid A 1	-1.46
ENSMUSG00000072915	Gm12258	predicted gene 12258	-1.46
ENSMUSG00000024986	Hhex	hematopoietically expressed homeobox	-1.46
ENSMUSG00000078949	R3hdml	R3H domain containing-like	-1.46
ENSMUSG00000042564	Fam227a	family with sequence similarity 227, member A	-1.46
ENSMUSG00000084950	Gm5577	predicted gene 5577	-1.46
ENSMUSG00000072683	Gm7457	predicted gene 7457	-1.46
ENSMUSG00000092482	Gm20531	predicted gene 20531	-1.46
ENSMUSG00000046242	Nme9	NME/NM23 family member 9	-1.46
ENSMUSG00000028139	Riiad1	regulatory subunit of type II PKA R-subunit (RIIa) domain containing 1	-1.46
ENSMUSG00000063873	Slc24a3	solute carrier family 24 (sodium/potassium/calcium exchanger), member 3	-1.46
ENSMUSG00000022416	Cacna1i	calcium channel, voltage-dependent, alpha 1I subunit	-1.46
ENSMUSG00000032925	Itgbl1	integrin, beta-like 1	-1.46
ENSMUSG00000034731	Dgkh	diacylglycerol kinase, eta	-1.47
ENSMUSG00000010307	Tmem86a	transmembrane protein 86A	-1.47
ENSMUSG00000084803	5830444B04Rik	RIKEN cDNA 5830444B04 gene	-1.47
ENSMUSG00000100183	Gm28512	predicted gene 28512	-1.47
ENSMUSG00000112501	Gm33091	predicted gene, 33091	-1.47
ENSMUSG00000032690	Oas2	2'-5' oligoadenylate synthetase 2	-1.47
ENSMUSG00000073400	Trim10	tripartite motif-containing 10	-1.47
ENSMUSG00000037235	Mxd4	Max dimerization protein 4	-1.48
ENSMUSG00000031770	Herpud1	homocysteine-inducible, endoplasmic reticulum stress-inducible, ubiquitin-like dom	-1.48
ENSMUSG00000036813	Entpd8	ectonucleoside triphosphate diphosphohydrolase 8	-1.48

ENSMUSG00000040327	Cul9	cullin 9	-1.48
ENSMUSG00000075590	Nrbp2	nuclear receptor binding protein 2	-1.48
ENSMUSG00000030717	Nupr1	nuclear protein transcription regulator 1	-1.48
ENSMUSG00000025196	Cpn1	carboxypeptidase N, polypeptide 1	-1.48
ENSMUSG00000037664	Cdkn1c	cyclin-dependent kinase inhibitor 1C (P57)	-1.48
ENSMUSG00000090293	Gm17034	predicted gene 17034	-1.48
ENSMUSG00000015879	Fam184b	family with sequence similarity 184, member B	-1.48
ENSMUSG00000018341	Il12rb2	interleukin 12 receptor, beta 2	-1.48
ENSMUSG00000112887	Gm47961	predicted gene, 47961	-1.48
ENSMUSG00000112556	Gm47339	predicted gene, 47339	-1.48
ENSMUSG00000007021	Syngr3	synaptogyrin 3	-1.48
ENSMUSG00000052520	Cyp2j5	cytochrome P450, family 2, subfamily j, polypeptide 5	-1.48
ENSMUSG00000036782	Klhl13	kelch-like 13	-1.48
ENSMUSG00000071019	Sdr16c6	short chain dehydrogenase/reductase family 16C, member 6	-1.48
ENSMUSG00000037977	6430571L13Rik	RIKEN cDNA 6430571L13 gene	-1.48
ENSMUSG00000039234	Sec24d	Sec24 related gene family, member D (S. cerevisiae)	-1.49
ENSMUSG00000034427	Myo15b	myosin XVB	-1.49
ENSMUSG00000052117	D630039A03Rik	RIKEN cDNA D630039A03 gene	-1.49
ENSMUSG00000036814	Slc6a20a	solute carrier family 6 (neurotransmitter transporter), member 20A	-1.49
ENSMUSG00000005268	Prlr	prolactin receptor	-1.49
ENSMUSG00000085333	1700030A11Rik	RIKEN cDNA 1700030A11 gene	-1.49
ENSMUSG00000036882	Arhgap33	Rho GTPase activating protein 33	-1.49
ENSMUSG00000005267	Zfp287	zinc finger protein 287	-1.49
ENSMUSG00000048481	Mypop	Myb-related transcription factor, partner of profilin	-1.49
ENSMUSG00000074981	Dcdc5	doublecortin domain containing 5	-1.49
ENSMUSG00000029185	Fam114a1	family with sequence similarity 114, member A1	-1.50
ENSMUSG00000009739	Pou6f1	POU domain, class 6, transcription factor 1	-1.50
ENSMUSG00000070315	4930581F22Rik	RIKEN cDNA 4930581F22 gene	-1.50
ENSMUSG00000015468	Notch4	notch 4	-1.50
ENSMUSG00000047656	Trpt1	tRNA phosphotransferase 1	-1.50
ENSMUSG00000055805	Fmnl1	formin-like 1	-1.50
ENSMUSG00000078161	Erich3	glutamate rich 3	-1.50
ENSMUSG00000040016	Ptger3	prostaglandin E receptor 3 (subtype EP3)	-1.50
ENSMUSG00000026940	Ccdc183	coiled-coil domain containing 183	-1.50
ENSMUSG00000106542	Gm43410	predicted gene 43410	-1.50
ENSMUSG00000011751	Sptbn4	spectrin beta, non-erythrocytic 4	-1.51
ENSMUSG00000047910	Pcdhb16	protocadherin beta 16	-1.51
ENSMUSG00000033276	Stk36	serine/threonine kinase 36	-1.51
ENSMUSG00000024065	Ehd3	EH-domain containing 3	-1.51
ENSMUSG00000103144	Pcdhga1	protocadherin gamma subfamily A, 1	-1.51
ENSMUSG00000100071	Gm28707	predicted gene 28707	-1.51
ENSMUSG00000059991	Nptx2	neuronal pentraxin 2	-1.52
ENSMUSG00000027950	Chrnb2	cholinergic receptor, nicotinic, beta polypeptide 2 (neuronal)	-1.52
ENSMUSG00000044681	Cnpy1	canopy FGF signaling regulator 1	-1.52
ENSMUSG00000103332	Pcdhga2	protocadherin gamma subfamily A, 2	-1.52
ENSMUSG00000052584	Serp2	stress-associated endoplasmic reticulum protein family member 2	-1.52
ENSMUSG00000106045	Gm42996	predicted gene 42996	-1.52
ENSMUSG00000041119	Pde9a	phosphodiesterase 9A	-1.53
ENSMUSG00000041351	Rap1gap	Rap1 GTPase-activating protein	-1.53
ENSMUSG00000022537	Tmem44	transmembrane protein 44	-1.53
ENSMUSG00000044067	Gpr22	G protein-coupled receptor 22	-1.53
ENSMUSG00000087651	1500009L16Rik	RIKEN cDNA 1500009L16 gene	-1.53
ENSMUSG00000055862	Izumo4	IZUMO family member 4	-1.53
ENSMUSG00000027801	Tm4sf4	transmembrane 4 superfamily member 4	-1.53
ENSMUSG00000111296	2010001M07Rik	RIKEN cDNA 2010001M07 gene	-1.53
ENSMUSG00000102533	Gm37226	predicted gene, 37226	-1.53
ENSMUSG00000021219	Rgs6	regulator of G-protein signaling 6	-1.53
ENSMUSG00000114626	Gm48266	predicted gene, 48266	-1.53
ENSMUSG00000027227	Sord	sorbitol dehydrogenase	-1.54
ENSMUSG00000026380	Tfcp2l1	transcription factor CP2-like 1	-1.54
ENSMUSG00000106847	Peg13	paternally expressed 13	-1.54
ENSMUSG00000006345	Ggt1	gamma-glutamyltransferase 1	-1.54
ENSMUSG00000049690	Nckap5	NCK-associated protein 5	-1.54
ENSMUSG00000037818	Abhd18	abhydrolase domain containing 18	-1.54
ENSMUSG00000020072	Pbld2	phenazine biosynthesis-like protein domain containing 2	-1.54
ENSMUSG00000031840	Rab3a	RAB3A, member RAS oncogene family	-1.54
ENSMUSG00000033898	Cfhr2	complement factor H-related 2	-1.54

ENSMUSG00000094605	Gm25873	predicted gene, 25873	-1.54
ENSMUSG00000028713	Cyp4b1	cytochrome P450, family 4, subfamily b, polypeptide 1	-1.55
ENSMUSG00000021336	Slc17a4	solute carrier family 17 (sodium phosphate), member 4	-1.55
ENSMUSG00000030364	Clec2h	C-type lectin domain family 2, member h	-1.55
ENSMUSG00000033308	Dpyd	dihydropyrimidine dehydrogenase	-1.55
ENSMUSG00000035274	Tpbg	trophoblast glycoprotein	-1.55
ENSMUSG00000046314	Stxbp6	syntaxin binding protein 6 (amisyn)	-1.55
ENSMUSG00000005873	Reep5	receptor accessory protein 5	-1.55
ENSMUSG00000103585	Pcdhgb4	protocadherin gamma subfamily B, 4	-1.55
ENSMUSG00000015053	Gata2	GATA binding protein 2	-1.55
ENSMUSG00000092035	Peg10	paternally expressed 10	-1.55
ENSMUSG00000020732	Rab37	RAB37, member RAS oncogene family	-1.55
ENSMUSG00000070777	Ceacam20	carcinoembryonic antigen-related cell adhesion molecule 20	-1.56
ENSMUSG00000030223	Ptpro	protein tyrosine phosphatase, receptor type, O	-1.56
ENSMUSG00000024924	Vldlr	very low density lipoprotein receptor	-1.56
ENSMUSG00000078490	Cfap74	cilia and flagella associated protein 74	-1.56
ENSMUSG00000072572	Slc39a2	solute carrier family 39 (zinc transporter), member 2	-1.56
ENSMUSG00000018378	Cuedc1	CUE domain containing 1	-1.57
ENSMUSG00000002565	Scin	scinderin	-1.57
ENSMUSG00000074207	Adh1	alcohol dehydrogenase 1 (class I)	-1.57
ENSMUSG00000102758	Naaladl2	N-acetylated alpha-linked acidic dipeptidase-like 2	-1.57
ENSMUSG00000097494	4933406C10Rik	RIKEN cDNA 4933406C10 gene	-1.57
ENSMUSG00000022683	Pla2g10	phospholipase A2, group X	-1.57
ENSMUSG00000074892	B3galt5	UDP-Gal:betaGlcNAc beta 1,3-galactosyltransferase, polypeptide 5	-1.58
ENSMUSG00000023809	Rps6ka2	ribosomal protein S6 kinase, polypeptide 2	-1.58
ENSMUSG00000020284	1810043G02Rik	RIKEN cDNA 1810043G02 gene	-1.58
ENSMUSG00000052131	Akr1b7	aldo-keto reductase family 1, member B7	-1.58
ENSMUSG00000030834	Abcc6	ATP-binding cassette, sub-family C (CFTR/MRP), member 6	-1.58
ENSMUSG00000105762	Gm43605	predicted gene 43605	-1.58
ENSMUSG00000029428	Stx2	syntaxin 2	-1.58
ENSMUSG00000074461	Gm10699	predicted gene 10699	-1.58
ENSMUSG00000075520	Malrd1	MAM and LDL receptor class A domain containing 1	-1.59
ENSMUSG00000003134	Tbc1d8	TBC1 domain family, member 8	-1.59
ENSMUSG00000028860	Sytl1	synaptotagmin-like 1	-1.59
ENSMUSG00000042523	Dnal1	dynein, axonemal, light chain 1	-1.59
ENSMUSG00000030621	Me3	malic enzyme 3, NADP(+)-dependent, mitochondrial	-1.59
ENSMUSG00000052605	Isoc2b	isochorismatase domain containing 2b	-1.59
ENSMUSG00000021364	Elovl2	elongation of very long chain fatty acids (FEN1/Elo2, SUR4/Elo3, yeast)-like 2	-1.59
ENSMUSG00000115098	Gm49134	predicted gene, 49134	-1.59
ENSMUSG00000000120	Ngfr	nerve growth factor receptor (TNFR superfamily, member 16)	-1.59
ENSMUSG00000105302	Gm19817	predicted gene, 19817	-1.59
ENSMUSG00000099893	Gm29245	predicted gene 29245	-1.59
ENSMUSG00000005800	Mmp8	matrix metallopeptidase 8	-1.59
ENSMUSG00000000202	Btbd17	BTB (POZ) domain containing 17	-1.60
ENSMUSG00000067203	H2-K2	histocompatibility 2, K region locus 2	-1.60
ENSMUSG00000102723	Gm37936	predicted gene, 37936	-1.60
ENSMUSG00000050600	Zfp831	zinc finger protein 831	-1.60
ENSMUSG00000030731	Syt3	synaptotagmin III	-1.60
ENSMUSG00000074912	Gm14207	predicted gene 14207	-1.61
ENSMUSG00000103284	3110080O07Rik	RIKEN cDNA 3110080O07 gene	-1.61
ENSMUSG00000017740	Slc12a5	solute carrier family 12, member 5	-1.61
ENSMUSG00000097768	2310043M15Rik	RIKEN cDNA 2310043M15 gene	-1.61
ENSMUSG00000107182	Gm43268	predicted gene 43268	-1.61
ENSMUSG00000019429	Ffar3	free fatty acid receptor 3	-1.61
ENSMUSG00000110301	Gm35363	predicted gene, 35363	-1.61
ENSMUSG00000001604	Tcea3	transcription elongation factor A (SII), 3	-1.62
ENSMUSG00000018740	Slc25a35	solute carrier family 25, member 35	-1.62
ENSMUSG00000035930	Chst4	carbohydrate (chondroitin 6/keratan) sulfotransferase 4	-1.62
ENSMUSG00000036492	Rnf39	ring finger protein 39	-1.62
ENSMUSG00000048960	Prex2	phosphatidylinositol-3,4,5-trisphosphate-dependent Rac exchange factor 2	-1.62
ENSMUSG00000043313	Pcdhb19	protocadherin beta 19	-1.62
ENSMUSG00000018581	Dnah11	dynein, axonemal, heavy chain 11	-1.62
ENSMUSG00000103577	9330162B11Rik	RIKEN cDNA 9330162B11 gene	-1.62
ENSMUSG00000027514	Zbp1	Z-DNA binding protein 1	-1.63
ENSMUSG00000022219	Cideb	cell death-inducing DNA fragmentation factor, alpha subunit-like effector B	-1.63
ENSMUSG00000095105	Edaradd	EDAR (ectodysplasin-A receptor)-associated death domain	-1.63
ENSMUSG00000025422	Agap2	ArfGAP with GTPase domain, ankyrin repeat and PH domain 2	-1.63

ENSMUSG00000030882	Dnhd1	dynein heavy chain domain 1	-1.63
ENSMUSG00000035849	Krt222	keratin 222	-1.63
ENSMUSG00000041696	Rasl12	RAS-like, family 12	-1.63
ENSMUSG00000068859	Sp9	trans-acting transcription factor 9	-1.63
ENSMUSG00000028845	Tekt2	tektin 2	-1.63
ENSMUSG00000064373	Selenop	selenoprotein P	-1.64
ENSMUSG00000022332	Khdrbs3	KH domain containing, RNA binding, signal transduction associated 3	-1.64
ENSMUSG00000036585	Fgf1	fibroblast growth factor 1	-1.64
ENSMUSG00000029700	Slc13a1	solute carrier family 13 (sodium/sulfate symporters), member 1	-1.64
ENSMUSG00000049971	Glt1d1	glycosyltransferase 1 domain containing 1	-1.64
ENSMUSG00000050295	Foxc1	forkhead box C1	-1.64
ENSMUSG00000001249	Hpn	hepsin	-1.64
ENSMUSG00000061578	Ksr2	kinase suppressor of ras 2	-1.64
ENSMUSG00000068196	Col8a1	collagen, type VIII, alpha 1	-1.64
ENSMUSG00000036040	Adamtsl2	ADAMTS-like 2	-1.64
ENSMUSG00000078942	Naip6	NLR family, apoptosis inhibitory protein 6	-1.65
ENSMUSG00000108427	Gm36696	predicted gene, 36696	-1.65
ENSMUSG00000037942	Crp	C-reactive protein, pentraxin-related	-1.65
ENSMUSG00000024193	Phf1	PHD finger protein 1	-1.66
ENSMUSG00000033705	Stard9	START domain containing 9	-1.66
ENSMUSG00000028795	Ccdc28b	coiled coil domain containing 28B	-1.66
ENSMUSG00000026728	Vim	vimentin	-1.66
ENSMUSG00000006241	Ccdc159	coiled-coil domain containing 159	-1.66
ENSMUSG00000092210	A930009A15Rik	RIKEN cDNA A930009A15 gene	-1.66
ENSMUSG00000037418	Best1	bestrophin 1	-1.66
ENSMUSG00000064140	Trim38	tripartite motif-containing 38	-1.66
ENSMUSG00000033684	Qsox1	quiescin Q6 sulfhydryl oxidase 1	-1.67
ENSMUSG00000039578	Ccser1	coiled-coil serine rich 1	-1.67
ENSMUSG00000048232	Fbxo10	F-box protein 10	-1.67
ENSMUSG00000035357	Pdzrn3	PDZ domain containing RING finger 3	-1.67
ENSMUSG00000007950	Abhd8	abhydrolase domain containing 8	-1.67
ENSMUSG00000018339	Gpx3	glutathione peroxidase 3	-1.67
ENSMUSG00000045912	C2cd4c	C2 calcium-dependent domain containing 4C	-1.67
ENSMUSG00000079114	Gm7849	predicted gene 7849	-1.67
ENSMUSG00000025479	Cyp2e1	cytochrome P450, family 2, subfamily e, polypeptide 1	-1.68
ENSMUSG00000085873	Ttc39aos1	Ttc39a opposite strand RNA 1	-1.68
ENSMUSG00000104966	Gm43273	predicted gene 43273	-1.68
ENSMUSG00000043614	Vps37d	vacuolar protein sorting 37D	-1.68
ENSMUSG00000032238	Rora	RAR-related orphan receptor alpha	-1.68
ENSMUSG00000039236	Isg20	interferon-stimulated protein	-1.68
ENSMUSG00000085003	Pip5k1bos	phosphatidylinositol-4-phosphate 5-kinase, type 1 beta, opposite strand	-1.68
ENSMUSG00000024391	Apom	apolipoprotein M	-1.68
ENSMUSG00000032251	Irak1bp1	interleukin-1 receptor-associated kinase 1 binding protein 1	-1.68
ENSMUSG00000039168	Dap	death-associated protein	-1.69
ENSMUSG00000038167	Plekhg6	pleckstrin homology domain containing, family G (with RhoGef domain) member 6	-1.69
ENSMUSG00000068551	Zfp467	zinc finger protein 467	-1.69
ENSMUSG00000051435	Fhad1	forkhead-associated (FHA) phosphopeptide binding domain 1	-1.69
ENSMUSG00000052921	Arhgef15	Rho guanine nucleotide exchange factor (GEF) 15	-1.69
ENSMUSG00000040061	Plcb2	phospholipase C, beta 2	-1.69
ENSMUSG00000086970	Bcas1os1	breast carcinoma amplified sequence 1, opposite strand 1	-1.69
ENSMUSG00000075015	Gm10801	predicted gene 10801	-1.69
ENSMUSG00000023781	Hes7	hes family bHLH transcription factor 7	-1.69
ENSMUSG00000081059	Gm11945	predicted gene 11945	-1.69
ENSMUSG00000016942	Tmprss6	transmembrane serine protease 6	-1.69
ENSMUSG00000091043	Glyatl3	glycine-N-acyltransferase-like 3	-1.69
ENSMUSG00000036377	C530008M17Rik	RIKEN cDNA C530008M17 gene	-1.70
ENSMUSG00000078486	Perm1	PPARGC1 and ESRR induced regulator, muscle 1	-1.70
ENSMUSG00000039976	Tbc1d16	TBC1 domain family, member 16	-1.70
ENSMUSG00000097924	A730020E08Rik	RIKEN cDNA A730020E08 gene	-1.70
ENSMUSG00000022199	Slc22a17	solute carrier family 22 (organic cation transporter), member 17	-1.70
ENSMUSG00000092323	BB365896	expressed sequence BB365896	-1.70
ENSMUSG00000084433	Gm25945	predicted gene, 25945	-1.70
ENSMUSG00000055003	Lrtm2	leucine-rich repeats and transmembrane domains 2	-1.70
ENSMUSG00000041261	Car8	carbonic anhydrase 8	-1.71
ENSMUSG00000010830	Kdelr3	KDEL (Lys-Asp-Glu-Leu) endoplasmic reticulum protein retention receptor 3	-1.71
ENSMUSG00000027612	Mmp24	matrix metallopeptidase 24	-1.71
ENSMUSG00000022440	C1qtnf6	C1q and tumor necrosis factor related protein 6	-1.71

ENSMUSG00000047473	Zfp30	zinc finger protein 30	-1.71
ENSMUSG00000073418	C4b	complement component 4B (Chido blood group)	-1.71
ENSMUSG00000079471	Mymx	myomixer, myoblast fusion factor	-1.71
ENSMUSG00000032226	Gcnt3	glucosaminyl (N-acetyl) transferase 3, mucin type	-1.72
ENSMUSG00000027457	Snph	syntaphilin	-1.72
ENSMUSG00000038497	Tmco3	transmembrane and coiled-coil domains 3	-1.73
ENSMUSG00000037025	Foxa2	forkhead box A2	-1.73
ENSMUSG00000030861	Acadsb	acyl-Coenzyme A dehydrogenase, short/branched chain	-1.73
ENSMUSG00000054252	Fgfr3	fibroblast growth factor receptor 3	-1.73
ENSMUSG00000003355	Fkbp11	FK506 binding protein 11	-1.73
ENSMUSG00000032661	Oas3	2'-5' oligoadenylate synthetase 3	-1.73
ENSMUSG00000033460	Armcx1	armadillo repeat containing, X-linked 1	-1.73
ENSMUSG00000039114	Nrn1	neuritin 1	-1.73
ENSMUSG00000030630	Fah	fumarylacetoacetate hydrolase	-1.73
ENSMUSG00000055497	Gm9974	predicted gene 9974	-1.73
ENSMUSG00000085589	A430078I02Rik	RIKEN cDNA A430078I02 gene	-1.73
ENSMUSG00000032353	Tmed3	transmembrane p24 trafficking protein 3	-1.74
ENSMUSG00000037709	Fam13a	family with sequence similarity 13, member A	-1.74
ENSMUSG00000033717	Adra2a	adrenergic receptor, alpha 2a	-1.74
ENSMUSG00000030483	Cyp2b10	cytochrome P450, family 2, subfamily b, polypeptide 10	-1.74
ENSMUSG00000117286	Gm1043	predicted 1043	-1.74
ENSMUSG00000047878	A4galt	alpha 1,4-galactosyltransferase	-1.74
ENSMUSG00000079174	Gm3054	predicted gene 3054	-1.75
ENSMUSG00000028838	Extl1	exostoses (multiple)-like 1	-1.75
ENSMUSG00000085852	Gm13807	predicted gene 13807	-1.75
ENSMUSG00000107076	Gm43480	predicted gene 43480	-1.75
ENSMUSG00000003410	Elavl3	ELAV like RNA binding protein 3	-1.75
ENSMUSG00000085941	Gm11201	predicted gene 11201	-1.75
ENSMUSG00000109093	Gm19950	predicted gene, 19950	-1.75
ENSMUSG00000104362	Gm37928	predicted gene, 37928	-1.75
ENSMUSG00000039943	Plcb4	phospholipase C, beta 4	-1.76
ENSMUSG00000003378	Grik5	glutamate receptor, ionotropic, kainate 5 (gamma 2)	-1.76
ENSMUSG00000110397	Gm45540	predicted gene 45540	-1.76
ENSMUSG00000004933	Matk	megakaryocyte-associated tyrosine kinase	-1.76
ENSMUSG00000081270	Gm11653	predicted gene 11653	-1.76
ENSMUSG00000024530	Prelid3a	PRELI domain containing 3A	-1.76
ENSMUSG00000045875	Adra1a	adrenergic receptor, alpha 1a	-1.76
ENSMUSG00000031451	Gas6	growth arrest specific 6	-1.77
ENSMUSG00000049307	Fut4	fucosyltransferase 4	-1.77
ENSMUSG00000024063	Lbh	limb-bud and heart	-1.77
ENSMUSG00000035246	Pcyt1b	phosphate cytidylyltransferase 1, choline, beta isoform	-1.77
ENSMUSG00000024778	Fas	Fas (TNF receptor superfamily member 6)	-1.77
ENSMUSG00000033287	Kctd17	potassium channel tetramerisation domain containing 17	-1.77
ENSMUSG00000094286	Gm12424	predicted gene 12424	-1.77
ENSMUSG00000110666	Gm9172	predicted gene 9172	-1.77
ENSMUSG00000023328	Ache	acetylcholinesterase	-1.78
ENSMUSG00000073067	9130019P16Rik	RIKEN cDNA 9130019P16 gene	-1.78
ENSMUSG00000032740	Ccdc88a	coiled coil domain containing 88A	-1.78
ENSMUSG00000087670	9530036M11Rik	RIKEN cDNA 9530036M11 gene	-1.78
ENSMUSG00000038777	Sema6c	sema domain, transmembrane domain (TM), and cytoplasmic domain, (semaphorir	-1.78
ENSMUSG00000033209	Ttc28	tetratricopeptide repeat domain 28	-1.78
ENSMUSG00000012017	Scarf2	scavenger receptor class F, member 2	-1.78
ENSMUSG00000074052	BC048644	cDNA sequence BC048644	-1.78
ENSMUSG00000028758	Kif17	kinesin family member 17	-1.78
ENSMUSG00000069456	Rdh16	retinol dehydrogenase 16	-1.79
ENSMUSG00000032053	Pou2af1	POU domain, class 2, associating factor 1	-1.79
ENSMUSG00000028186	Uox	urate oxidase	-1.79
ENSMUSG00000021414	Fam217a	family with sequence similarity 217, member A	-1.79
ENSMUSG00000021057	Akap5	A kinase (PRKA) anchor protein 5	-1.80
ENSMUSG00000027075	Slc43a1	solute carrier family 43, member 1	-1.80
ENSMUSG00000112307	Gm48751	predicted gene, 48751	-1.80
ENSMUSG00000056856	Jakmip3	janus kinase and microtubule interacting protein 3	-1.80
ENSMUSG00000056648	Hoxb8	homeobox B8	-1.80
ENSMUSG00000092384	Gm4189	predicted gene 4189	-1.80
ENSMUSG00000028524	Sgip1	SH3-domain GRB2-like (endophilin) interacting protein 1	-1.80
ENSMUSG00000027435	Cd93	CD93 antigen	-1.80
ENSMUSG00000036390	Gadd45a	growth arrest and DNA-damage-inducible 45 alpha	-1.81

ENSMUSG00000011832	Evi5l	ecotropic viral integration site 5 like	-1.81
ENSMUSG00000015981	Stk32c	serine/threonine kinase 32C	-1.81
ENSMUSG00000039485	Tspyl4	TSPY-like 4	-1.81
ENSMUSG00000055370	Gm9968	predicted gene 9968	-1.81
ENSMUSG00000040387	Klhl32	kelch-like 32	-1.81
ENSMUSG00000031133	Arhgef6	Rac/Cdc42 guanine nucleotide exchange factor (GEF) 6	-1.81
ENSMUSG00000111323	Gm48129	predicted gene, 48129	-1.81
ENSMUSG00000099826	Scgb2b10	secretoglobin, family 2B, member 10	-1.81
ENSMUSG00000040987	Mill2	MHC I like leukocyte 2	-1.82
ENSMUSG00000110540	Gm45743	predicted gene 45743	-1.82
ENSMUSG00000004630	Pcp2	Purkinje cell protein 2 (L7)	-1.82
ENSMUSG00000027314	Dll4	delta like canonical Notch ligand 4	-1.83
ENSMUSG00000051335	Gfod1	glucose-fructose oxidoreductase domain containing 1	-1.83
ENSMUSG00000006711	D130043K22Rik	RIKEN cDNA D130043K22 gene	-1.83
ENSMUSG00000020034	Tcp11l2	t-complex 11 (mouse) like 2	-1.83
ENSMUSG00000025221	Kcnip2	Kv channel-interacting protein 2	-1.83
ENSMUSG00000078091	Gm10912	predicted gene 10912	-1.83
ENSMUSG00000091189	Ear-ps3	eosinophil-associated, ribonuclease A family, pseudogene 3	-1.83
ENSMUSG00000024109	Nrxn1	neurexin I	-1.83
ENSMUSG00000071203	Naip5	NLR family, apoptosis inhibitory protein 5	-1.84
ENSMUSG00000028634	Hivep3	human immunodeficiency virus type I enhancer binding protein 3	-1.84
ENSMUSG00000040350	Trim7	tripartite motif-containing 7	-1.84
ENSMUSG00000049804	Armcx4	armadillo repeat containing, X-linked 4	-1.84
ENSMUSG00000025104	Hdgfl3	HDGF like 3	-1.84
ENSMUSG00000087049	Bach2it1	BTB and CNC homology 2, intronic transcript 1	-1.84
ENSMUSG00000063522	2010109I03Rik	RIKEN cDNA 2010109I03 gene	-1.85
ENSMUSG00000025352	Gdf11	growth differentiation factor 11	-1.85
ENSMUSG00000056185	Snx32	sorting nexin 32	-1.85
ENSMUSG00000037902	Sirpa	signal-regulatory protein alpha	-1.85
ENSMUSG00000053675	Tgm5	transglutaminase 5	-1.85
ENSMUSG00000032101	Ddx25	DEAD (Asp-Glu-Ala-Asp) box polypeptide 25	-1.85
ENSMUSG00000029563	Foxp2	forkhead box P2	-1.85
ENSMUSG00000034112	Atp2c2	ATPase, Ca++ transporting, type 2C, member 2	-1.86
ENSMUSG00000036422	Pcdh8	protocadherin 8	-1.86
ENSMUSG00000040896	Kcnd3	potassium voltage-gated channel, Shal-related family, member 3	-1.86
ENSMUSG00000042388	Dlgap3	DLG associated protein 3	-1.86
ENSMUSG00000047904	Sstr2	somatostatin receptor 2	-1.86
ENSMUSG00000044250	Pced1b	PC-esterase domain containing 1B	-1.86
ENSMUSG00000038608	Dock10	dedicator of cytokinesis 10	-1.86
ENSMUSG00000110131	Gm18066	predicted gene, 18066	-1.86
ENSMUSG00000022012	Enox1	ecto-NOX disulfide-thiol exchanger 1	-1.86
ENSMUSG00000082443	Gm12604	predicted gene 12604	-1.86
ENSMUSG00000033715	Akr1c14	aldo-keto reductase family 1, member C14	-1.87
ENSMUSG00000039115	Itga9	integrin alpha 9	-1.87
ENSMUSG00000029769	Ccdc136	coiled-coil domain containing 136	-1.87
ENSMUSG00000041710	Trpc5	transient receptor potential cation channel, subfamily C, member 5	-1.87
ENSMUSG00000087623	Gm12404	predicted gene 12404	-1.87
ENSMUSG00000030207	Fam234b	family with sequence similarity 234, member B	-1.88
ENSMUSG00000084939	Gm830	predicted gene 830	-1.88
ENSMUSG00000029919	Hpgds	hematopoietic prostaglandin D synthase	-1.88
ENSMUSG00000054434	Tmem120b	transmembrane protein 120B	-1.88
ENSMUSG00000072720	Myo18b	myosin XVIIIb	-1.88
ENSMUSG00000014158	Trpv4	transient receptor potential cation channel, subfamily V, member 4	-1.88
ENSMUSG00000031561	Tenm3	teneurin transmembrane protein 3	-1.88
ENSMUSG00000117328	AC169675.1	novel transcript, antisense to Smoc2	-1.88
ENSMUSG00000027188	Pamr1	peptidase domain containing associated with muscle regeneration 1	-1.88
ENSMUSG00000065145	Vaultrc5	vault RNA component 5	-1.89
ENSMUSG00000038893	Fam117a	family with sequence similarity 117, member A	-1.89
ENSMUSG00000023927	Satb1	special AT-rich sequence binding protein 1	-1.89
ENSMUSG00000032978	Guca2b	guanylate cyclase activator 2b (retina)	-1.90
ENSMUSG00000039652	Cpeb3	cytoplasmic polyadenylation element binding protein 3	-1.90
ENSMUSG00000025656	Arhgef9	CDC42 guanine nucleotide exchange factor (GEF) 9	-1.90
ENSMUSG00000026959	Grin1	glutamate receptor, ionotropic, NMDA1 (zeta 1)	-1.90
ENSMUSG00000027217	Tspan18	tetraspanin 18	-1.90
ENSMUSG00000050132	Sarm1	sterile alpha and HEAT/Armadillo motif containing 1	-1.90
ENSMUSG00000089712	Gm15889	predicted gene 15889	-1.91
ENSMUSG00000035067	Xkr6	X-linked Kx blood group related 6	-1.92

ENSMUSG00000073424	Cyp4f15	cytochrome P450, family 4, subfamily f, polypeptide 15	-1.92
ENSMUSG00000041789	2700046A07Rik	RIKEN cDNA 2700046A07 gene	-1.92
ENSMUSG00000048078	Tenm4	teneurin transmembrane protein 4	-1.93
ENSMUSG00000057069	Ero1lb	ERO1-like beta (S. cerevisiae)	-1.93
ENSMUSG00000026360	Rgs2	regulator of G-protein signaling 2	-1.93
ENSMUSG00000103579	Gm37113	predicted gene, 37113	-1.93
ENSMUSG00000027339	Rassf2	Ras association (RalGDS/AF-6) domain family member 2	-1.93
ENSMUSG00000029797	Sspo	SCO-spondin	-1.93
ENSMUSG00000047161	Chst9	carbohydrate (N-acetylgalactosamine 4-0) sulfotransferase 9	-1.93
ENSMUSG00000024039	Cbs	cystathionine beta-synthase	-1.94
ENSMUSG00000062859	Tcp11	t-complex protein 11	-1.94
ENSMUSG00000030228	Pik3c2g	phosphatidylinositol-4-phosphate 3-kinase catalytic subunit type 2 gamma	-1.94
ENSMUSG00000031202	Rab39b	RAB39B, member RAS oncogene family	-1.94
ENSMUSG00000020423	Btg2	B cell translocation gene 2, anti-proliferative	-1.95
ENSMUSG00000041132	N4bp2l1	NEDD4 binding protein 2-like 1	-1.95
ENSMUSG00000018623	Mmp7	matrix metallopeptidase 7	-1.95
ENSMUSG00000054150	Syne3	spectrin repeat containing, nuclear envelope family member 3	-1.95
ENSMUSG00000009628	Tex15	testis expressed gene 15	-1.95
ENSMUSG00000016356	Col20a1	collagen, type XX, alpha 1	-1.95
ENSMUSG00000103220	Gm37728	predicted gene, 37728	-1.95
ENSMUSG00000039419	Cntnap2	contactin associated protein-like 2	-1.95
ENSMUSG00000067642	Adgrf3	adhesion G protein-coupled receptor F3	-1.95
ENSMUSG00000055737	Ghr	growth hormone receptor	-1.96
ENSMUSG00000018427	Ypel2	yippee like 2	-1.96
ENSMUSG00000034591	Slc41a2	solute carrier family 41, member 2	-1.96
ENSMUSG00000024124	Prss30	protease, serine 30	-1.96
ENSMUSG00000006464	Bbs1	Bardet-Biedl syndrome 1 (human)	-1.96
ENSMUSG00000041912	Tdrkh	tudor and KH domain containing protein	-1.96
ENSMUSG00000043020	Wdr63	WD repeat domain 63	-1.96
ENSMUSG00000115360	Gm48932	predicted gene, 48932	-1.96
ENSMUSG00000032609	Klhdc8b	kelch domain containing 8B	-1.97
ENSMUSG00000021675	F2rl2	coagulation factor II (thrombin) receptor-like 2	-1.97
ENSMUSG00000074782	4833422C13Rik	RIKEN cDNA 4833422C13 gene	-1.97
ENSMUSG00000025978	Rftn2	raftlin family member 2	-1.97
ENSMUSG00000036887	C1qa	complement component 1, q subcomponent, alpha polypeptide	-1.97
ENSMUSG00000040867	Begain	brain-enriched guanylate kinase-associated	-1.97
ENSMUSG00000042269	Fam92b	family with sequence similarity 92, member B	-1.97
ENSMUSG00000072115	Ang	angiogenin, ribonuclease, RNase A family, 5	-1.98
ENSMUSG00000060935	Tmem263	transmembrane protein 263	-1.98
ENSMUSG00000006930	Hap1	huntingtin-associated protein 1	-1.98
ENSMUSG00000061808	Ttr	transthyretin	-1.98
ENSMUSG00000111282	Gm47528	predicted gene, 47528	-1.98
ENSMUSG00000117050	AC167363.2	TEC	-1.98
ENSMUSG00000020486	38231	septin 4	-1.98
ENSMUSG00000057400	Ces1c	carboxylesterase 1C	-1.98
ENSMUSG00000066516	Klk1b21	kallikrein 1-related peptidase b21	-1.98
ENSMUSG00000025427	Rnf165	ring finger protein 165	-1.98
ENSMUSG00000052387	Trpm3	transient receptor potential cation channel, subfamily M, member 3	-1.98
ENSMUSG00000051910	Sox6	SRY (sex determining region Y)-box 6	-2.00
ENSMUSG00000049191	Rtl5	retrotransposon Gag like 5	-2.00
ENSMUSG00000031791	Tmem38a	transmembrane protein 38A	-2.00
ENSMUSG00000034949	Zfr2	zinc finger RNA binding protein 2	-2.00
ENSMUSG00000039629	Strip2	striatin interacting protein 2	-2.00
ENSMUSG00000023966	Rsph9	radial spoke head 9 homolog (Chlamydomonas)	-2.00
ENSMUSG00000085664	Atxn7l1os2	ataxin 7-like 1, opposite strand 2	-2.00
ENSMUSG00000036196	Slc26a8	solute carrier family 26, member 8	-2.00
ENSMUSG00000036892	Prodh2	proline dehydrogenase (oxidase) 2	-2.00
ENSMUSG00000061576	Dpp6	dipeptidylpeptidase 6	-2.01
ENSMUSG00000090812	Samd15	sterile alpha motif domain containing 15	-2.01
ENSMUSG00000106749	Gm43281	predicted gene 43281	-2.01
ENSMUSG00000088891	Gm22516	predicted gene, 22516	-2.01
ENSMUSG00000058897	Col25a1	collagen, type XXV, alpha 1	-2.01
ENSMUSG00000105495	Gm42995	predicted gene 42995	-2.01
ENSMUSG00000029166	Mapre3	microtubule-associated protein, RP/EB family, member 3	-2.02
ENSMUSG00000032548	Slco2a1	solute carrier organic anion transporter family, member 2a1	-2.02
ENSMUSG00000040415	Dtx3	deltex 3, E3 ubiquitin ligase	-2.02
ENSMUSG00000035211	Xrra1	X-ray radiation resistance associated 1	-2.02

ENSMUSG00000031303	Map3k15	mitogen-activated protein kinase kinase kinase 15	-2.03
ENSMUSG00000047146	Tet1	tet methylcytosine dioxygenase 1	-2.03
ENSMUSG00000028076	Cd1d1	CD1d1 antigen	-2.03
ENSMUSG00000112601	Gm47590	predicted gene, 47590	-2.03
ENSMUSG00000023906	Cldn6	claudin 6	-2.04
ENSMUSG00000046378	Asphd1	aspartate beta-hydroxylase domain containing 1	-2.04
ENSMUSG00000032356	Rasgrf1	RAS protein-specific guanine nucleotide-releasing factor 1	-2.04
ENSMUSG00000106350	Gm4869	predicted gene 4869	-2.04
ENSMUSG00000045658	Pid1	phosphotyrosine interaction domain containing 1	-2.04
ENSMUSG00000056679	Gpr173	G-protein coupled receptor 173	-2.04
ENSMUSG00000038271	Iffo1	intermediate filament family orphan 1	-2.05
ENSMUSG00000094840	Muc3a	mucin 3A, cell surface associated	-2.06
ENSMUSG00000040852	Plekhh2	pleckstrin homology domain containing, family H (with MyTH4 domain) member 2	-2.06
ENSMUSG00000059898	Dsc3	desmocollin 3	-2.06
ENSMUSG00000104776	Gm43691	predicted gene 43691	-2.06
ENSMUSG00000073102	Drc1	dynein regulatory complex subunit 1	-2.06
ENSMUSG00000027297	Ltk	leukocyte tyrosine kinase	-2.07
ENSMUSG00000053141	Ptprt	protein tyrosine phosphatase, receptor type, T	-2.07
ENSMUSG00000040495	Chrm4	cholinergic receptor, muscarinic 4	-2.07
ENSMUSG00000042109	Csdc2	cold shock domain containing C2, RNA binding	-2.07
ENSMUSG00000020140	Lgr5	leucine rich repeat containing G protein coupled receptor 5	-2.08
ENSMUSG00000027223	Mapk8ip1	mitogen-activated protein kinase 8 interacting protein 1	-2.08
ENSMUSG00000051166	Eml5	echinoderm microtubule associated protein like 5	-2.08
ENSMUSG00000054893	Zfp667	zinc finger protein 667	-2.08
ENSMUSG00000044726	Erich5	glutamate rich 5	-2.08
ENSMUSG00000097328	Tnfsf12	tumor necrosis factor (ligand) superfamily, member 12	-2.08
ENSMUSG00000107451	Gm44421	predicted gene, 44421	-2.08
ENSMUSG00000045498	Pcdhb3	protocadherin beta 3	-2.08
ENSMUSG00000095066	Defa20	defensin, alpha, 20	-2.08
ENSMUSG00000031824	6430548M08Rik	RIKEN cDNA 6430548M08 gene	-2.09
ENSMUSG00000085363	Gm15478	predicted gene 15478	-2.09
ENSMUSG00000026841	Fibcd1	fibrinogen C domain containing 1	-2.09
ENSMUSG00000029359	Tesc	tescalcin	-2.10
ENSMUSG00000038793	Lefty1	left right determination factor 1	-2.10
ENSMUSG00000030406	Gipr	gastric inhibitory polypeptide receptor	-2.10
ENSMUSG00000020953	Coch	cochlin	-2.10
ENSMUSG00000105720	Gm42440	predicted gene 42440	-2.10
ENSMUSG00000050963	Kcns2	K+ voltage-gated channel, subfamily S, 2	-2.10
ENSMUSG00000102112	1810041H14Rik	RIKEN cDNA 1810041H14 gene	-2.11
ENSMUSG00000003411	Rab3b	RAB3B, member RAS oncogene family	-2.11
ENSMUSG00000020930	Ccdc103	coiled-coil domain containing 103	-2.11
ENSMUSG00000038175	Mylip	myosin regulatory light chain interacting protein	-2.12
ENSMUSG00000112035	Gm49335	predicted gene, 49335	-2.12
ENSMUSG00000027931	Npr1	natriuretic peptide receptor 1	-2.13
ENSMUSG00000040367	Lrrd1	leucine rich repeats and death domain containing 1	-2.13
ENSMUSG00000104936	2610011E03Rik	RIKEN cDNA 2610011E03 gene	-2.13
ENSMUSG00000103331	Gm37995	predicted gene, 37995	-2.14
ENSMUSG00000017639	Rab11fip4	RAB11 family interacting protein 4 (class II)	-2.15
ENSMUSG00000034116	Vav1	vav 1 oncogene	-2.15
ENSMUSG00000110887	Cbx3-ps8	chromobox 3, pseudogene 8	-2.15
ENSMUSG00000063160	Numbl	numb-like	-2.15
ENSMUSG00000031919	Tmed6	transmembrane p24 trafficking protein 6	-2.16
ENSMUSG00000049892	Rasd1	RAS, dexamethasone-induced 1	-2.16
ENSMUSG00000111086	Gm48671	predicted gene, 48671	-2.16
ENSMUSG00000031523	Dlc1	deleted in liver cancer 1	-2.16
ENSMUSG00000034115	Scn11a	sodium channel, voltage-gated, type XI, alpha	-2.16
ENSMUSG00000027359	Slc27a2	solute carrier family 27 (fatty acid transporter), member 2	-2.17
ENSMUSG00000110622	Iqcn	IQ motif containing N	-2.17
ENSMUSG00000006958	Chrd	chordin	-2.17
ENSMUSG00000108961	Gm32540	predicted gene, 32540	-2.17
ENSMUSG00000038807	Rap1gap2	RAP1 GTPase activating protein 2	-2.18
ENSMUSG00000062785	Kcnc3	potassium voltage gated channel, Shaw-related subfamily, member 3	-2.18
ENSMUSG00000004187	Kifc2	kinesin family member C2	-2.18
ENSMUSG00000021379	Id4	inhibitor of DNA binding 4	-2.18
ENSMUSG00000105289	Gm43483	predicted gene 43483	-2.18
ENSMUSG00000020182	Ddc	dopa decarboxylase	-2.19
ENSMUSG00000072294	Klf12	Kruppel-like factor 12	-2.19

ENSMUSG00000055214	Pld5	phospholipase D family, member 5	-2.20
ENSMUSG00000029544	Cabp1	calcium binding protein 1	-2.20
ENSMUSG00000036027	1810046K07Rik	RIKEN cDNA 1810046K07 gene	-2.20
ENSMUSG00000078964	Ces1b	carboxylesterase 1B	-2.20
ENSMUSG00000105485	Gm42447	predicted gene 42447	-2.20
ENSMUSG00000047787	Flrt1	fibronectin leucine rich transmembrane protein 1	-2.22
ENSMUSG00000017652	Cd40	CD40 antigen	-2.22
ENSMUSG00000023828	Slc22a3	solute carrier family 22 (organic cation transporter), member 3	-2.22
ENSMUSG00000073293	Nudt10	nudix (nucleoside diphosphate linked moiety X)-type motif 10	-2.22
ENSMUSG00000040310	Alx4	aristaless-like homeobox 4	-2.23
ENSMUSG00000089371	Mir1938	microRNA 1938	-2.24
ENSMUSG00000021453	Gadd45g	growth arrest and DNA-damage-inducible 45 gamma	-2.25
ENSMUSG00000071489	Ptgdr	prostaglandin D receptor	-2.25
ENSMUSG00000094305	Scgb2b20	secretoglobin, family 2B, member 20	-2.25
ENSMUSG00000027938	Creb3l4	cAMP responsive element binding protein 3-like 4	-2.26
ENSMUSG00000034675	Dbn1	drebrin 1	-2.26
ENSMUSG00000020227	Irak3	interleukin-1 receptor-associated kinase 3	-2.26
ENSMUSG00000037738	Nek5	NIMA (never in mitosis gene a)-related expressed kinase 5	-2.26
ENSMUSG00000037217	Syn1	synapsin I	-2.26
ENSMUSG00000097294	Gm26888	predicted gene, 26888	-2.26
ENSMUSG00000046668	Cxxc5	CXXC finger 5	-2.27
ENSMUSG00000050100	Hmx2	H6 homeobox 2	-2.27
ENSMUSG00000032549	Rab6b	RAB6B, member RAS oncogene family	-2.28
ENSMUSG00000052726	Kcnt2	potassium channel, subfamily T, member 2	-2.28
ENSMUSG00000049811	Fam161a	family with sequence similarity 161, member A	-2.28
ENSMUSG00000108348	Gm42372	predicted gene, 42372	-2.28
ENSMUSG00000070385	Ampd1	adenosine monophosphate deaminase 1	-2.28
ENSMUSG00000112805	Gm34574	predicted gene, 34574	-2.28
ENSMUSG00000024232	Bambi	BMP and activin membrane-bound inhibitor	-2.29
ENSMUSG00000041000	Trim62	tripartite motif-containing 62	-2.29
ENSMUSG00000048330	Ric3	RIC3 acetylcholine receptor chaperone	-2.29
ENSMUSG00000047181	Samd14	sterile alpha motif domain containing 14	-2.29
ENSMUSG00000035863	Palm	paralemmin	-2.29
ENSMUSG00000028327	Stra6l	STRA6-like	-2.30
ENSMUSG00000057335	Cep170	centrosomal protein 170	-2.30
ENSMUSG00000000530	Acvrl1	activin A receptor, type II-like 1	-2.30
ENSMUSG00000097194	9330175E14Rik	RIKEN cDNA 9330175E14 gene	-2.30
ENSMUSG00000052861	Dnah6	dynein, axonemal, heavy chain 6	-2.30
ENSMUSG00000025576	Rbfox3	RNA binding protein, fox-1 homolog (C. elegans) 3	-2.31
ENSMUSG00000037492	Zmat4	zinc finger, matrin type 4	-2.31
ENSMUSG00000029490	Mfsd7a	major facilitator superfamily domain containing 7A	-2.32
ENSMUSG00000000305	Cdh4	cadherin 4	-2.32
ENSMUSG00000089678	Agxt2	alanine-glyoxylate aminotransferase 2	-2.32
ENSMUSG00000066687	Zbtb16	zinc finger and BTB domain containing 16	-2.33
ENSMUSG00000052942	Glis3	GLIS family zinc finger 3	-2.33
ENSMUSG00000025787	Tgm4	transglutaminase 4 (prostate)	-2.33
ENSMUSG00000079559	Colca2	COLCA2 homolog	-2.33
ENSMUSG00000034758	Tle6	transducin-like enhancer of split 6	-2.33
ENSMUSG00000020651	Slc26a4	solute carrier family 26, member 4	-2.33
ENSMUSG00000046719	Nxph3	neurexophilin 3	-2.33
ENSMUSG00000060508	Nlrp9b	NLR family, pyrin domain containing 9B	-2.34
ENSMUSG00000025969	Nrp2	neuropilin 2	-2.34
ENSMUSG00000024500	Ppp2r2b	protein phosphatase 2, regulatory subunit B, beta	-2.34
ENSMUSG00000008193	Spib	Spi-B transcription factor (Spi-1/PU.1 related)	-2.34
ENSMUSG00000102620	Gm37675	predicted gene, 37675	-2.34
ENSMUSG00000022438	Parvb	parvin, beta	-2.34
ENSMUSG00000106825	2510016D11Rik	RIKEN cDNA 2510016D11 gene	-2.35
ENSMUSG00000028909	Ptpru	protein tyrosine phosphatase, receptor type, U	-2.35
ENSMUSG00000083718	Ccnb2-ps	cyclin B2, pseudogene	-2.35
ENSMUSG00000008461	Fut1	fucosyltransferase 1	-2.35
ENSMUSG00000044022	Pcdhb21	protocadherin beta 21	-2.35
ENSMUSG00000042249	Grk3	G protein-coupled receptor kinase 3	-2.36
ENSMUSG00000052026	Slc6a7	solute carrier family 6 (neurotransmitter transporter, L-proline), member 7	-2.36
ENSMUSG00000037754	Ppp1r16b	protein phosphatase 1, regulatory subunit 16B	-2.36
ENSMUSG00000002578	Ikzf4	IKAROS family zinc finger 4	-2.36
ENSMUSG00000041577	Prelp	proline arginine-rich end leucine-rich repeat	-2.37
ENSMUSG00000006651	Aplp1	amyloid beta (A4) precursor-like protein 1	-2.38

ENSMUSG00000039126	Prune2	prune homolog 2	-2.38
ENSMUSG00000029408	Abcb9	ATP-binding cassette, sub-family B (MDR/TAP), member 9	-2.38
ENSMUSG00000033427	Upb1	ureidopropionase, beta	-2.38
ENSMUSG00000029275	Gfi1	growth factor independent 1	-2.38
ENSMUSG00000019986	Ahi1	Abelson helper integration site 1	-2.38
ENSMUSG00000022661	Cd200	CD200 antigen	-2.38
ENSMUSG00000037341	Slc9a7	solute carrier family 9 (sodium/hydrogen exchanger), member 7	-2.38
ENSMUSG00000021876	Rnase4	ribonuclease, RNase A family 4	-2.39
ENSMUSG00000021986	Amer2	APC membrane recruitment 2	-2.39
ENSMUSG00000035486	Plk5	polo like kinase 5	-2.39
ENSMUSG00000043789	Vwce	von Willebrand factor C and EGF domains	-2.40
ENSMUSG00000001663	Gstt1	glutathione S-transferase, theta 1	-2.40
ENSMUSG00000043458	Pcdhb12	protocadherin beta 12	-2.40
ENSMUSG00000103466	Gm37247	predicted gene, 37247	-2.40
ENSMUSG00000036295	Lrrn3	leucine rich repeat protein 3, neuronal	-2.41
ENSMUSG00000022208	Jph4	junctophilin 4	-2.41
ENSMUSG00000042096	Dao	D-amino acid oxidase	-2.41
ENSMUSG00000056031	9330154J02Rik	RIKEN cDNA 9330154J02 gene	-2.41
ENSMUSG00000055430	Nap1l5	nucleosome assembly protein 1-like 5	-2.41
ENSMUSG00000069227	Gprin1	G protein-regulated inducer of neurite outgrowth 1	-2.42
ENSMUSG00000020599	Rgs9	regulator of G-protein signaling 9	-2.42
ENSMUSG00000112124	Gm19056	predicted gene, 19056	-2.42
ENSMUSG00000116287	Gm3924	predicted gene 3924	-2.42
ENSMUSG00000003153	Slc2a3	solute carrier family 2 (facilitated glucose transporter), member 3	-2.42
ENSMUSG00000020990	Cdkl1	cyclin-dependent kinase-like 1 (CDC2-related kinase)	-2.42
ENSMUSG00000028972	Car6	carbonic anhydrase 6	-2.42
ENSMUSG00000021913	Ogdhl	oxoglutarate dehydrogenase-like	-2.43
ENSMUSG00000039579	Grin3a	glutamate receptor ionotropic, NMDA3A	-2.43
ENSMUSG00000038555	Reep2	receptor accessory protein 2	-2.43
ENSMUSG00000062296	Trank1	tetratricopeptide repeat and ankyrin repeat containing 1	-2.43
ENSMUSG00000019467	Arhgef25	Rho guanine nucleotide exchange factor (GEF) 25	-2.43
ENSMUSG00000005672	Kit	KIT proto-oncogene receptor tyrosine kinase	-2.44
ENSMUSG00000027230	Creb3l1	cAMP responsive element binding protein 3-like 1	-2.44
ENSMUSG00000041268	Dmxl2	Dmx-like 2	-2.44
ENSMUSG00000044694	2010007H06Rik	RIKEN cDNA 2010007H06 gene	-2.44
ENSMUSG00000028439	Fam219a	family with sequence similarity 219, member A	-2.44
ENSMUSG00000091735	Gpr62	G protein-coupled receptor 62	-2.44
ENSMUSG00000087675	Gm11762	predicted gene 11762	-2.44
ENSMUSG00000102481	Gm37925	predicted gene, 37925	-2.44
ENSMUSG00000033730	Egr3	early growth response 3	-2.45
ENSMUSG00000062044	Lmtk3	lemur tyrosine kinase 3	-2.45
ENSMUSG00000030911	Zp2	zona pellucida glycoprotein 2	-2.45
ENSMUSG00000006575	Rundc3a	RUN domain containing 3A	-2.46
ENSMUSG00000035580	Kcnh8	potassium voltage-gated channel, subfamily H (eag-related), member 8	-2.46
ENSMUSG00000074657	Kif5a	kinesin family member 5A	-2.46
ENSMUSG00000045657	Pcdhb10	protocadherin beta 10	-2.46
ENSMUSG00000117254	AC171111.1	novel transcript	-2.47
ENSMUSG00000034438	Gbp8	guanylate-binding protein 8	-2.47
ENSMUSG00000097587	4930578M01Rik	RIKEN cDNA 4930578M01 gene	-2.47
ENSMUSG00000022580	Rhpn1	rhophilin, Rho GTPase binding protein 1	-2.47
ENSMUSG00000102424	Paupar	Pax6 upstream antisense RNA	-2.47
ENSMUSG00000069378	Prdm6	PR domain containing 6	-2.47
ENSMUSG00000038692	Hoxb4	homeobox B4	-2.48
ENSMUSG00000112433	Gm30122	predicted gene, 30122	-2.48
ENSMUSG00000045467	Ttll13	tubulin tyrosine ligase-like family, member 13	-2.48
ENSMUSG00000042351	Grap2	GRB2-related adaptor protein 2	-2.48
ENSMUSG00000034111	Tmed8	transmembrane p24 trafficking protein 8	-2.49
ENSMUSG00000029102	Hgfac	hepatocyte growth factor activator	-2.49
ENSMUSG00000074882	Cyp2c68	cytochrome P450, family 2, subfamily c, polypeptide 68	-2.49
ENSMUSG00000001248	Gramd1a	GRAM domain containing 1A	-2.49
ENSMUSG00000029298	Gbp9	guanylate-binding protein 9	-2.49
ENSMUSG00000025348	Itga7	integrin alpha 7	-2.49
ENSMUSG00000060843	Ctnna3	catenin (cadherin associated protein), alpha 3	-2.49
ENSMUSG00000050822	Slc29a4	solute carrier family 29 (nucleoside transporters), member 4	-2.50
ENSMUSG00000079442	St6galnac4	ST6 (alpha-N-acetyl-neuraminyl-2,3-beta-galactosyl-1,3)-N-acetylgalactosaminide a	-2.50
ENSMUSG00000070498	Tmem132b	transmembrane protein 132B	-2.50
ENSMUSG00000049551	Fzd9	frizzled class receptor 9	-2.51

ENSMUSG00000038370	Pcp4l1	Purkinje cell protein 4-like 1	-2.51
ENSMUSG00000040183	Ankrd6	ankyrin repeat domain 6	-2.51
ENSMUSG00000063428	Ddo	D-aspartate oxidase	-2.51
ENSMUSG00000023826	Prkn	parkin RBR E3 ubiquitin protein ligase	-2.51
ENSMUSG00000051486	Pcdhb11	protocadherin beta 11	-2.51
ENSMUSG00000032500	Dclk3	doublecortin-like kinase 3	-2.51
ENSMUSG00000020847	Rph3al	rabphilin 3A-like (without C2 domains)	-2.52
ENSMUSG00000053046	Brsk2	BR serine/threonine kinase 2	-2.52
ENSMUSG00000048782	Insc	INSC spindle orientation adaptor protein	-2.52
ENSMUSG00000040428	Plekha4	pleckstrin homology domain containing, family A (phosphoinositide binding specific	-2.52
ENSMUSG00000030317	Timp4	tissue inhibitor of metalloproteinase 4	-2.52
ENSMUSG00000111792	Gm33858	predicted gene, 33858	-2.52
ENSMUSG00000018830	Myh11	myosin, heavy polypeptide 11, smooth muscle	-2.53
ENSMUSG00000103529	A730089K16Rik	RIKEN cDNA A730089K16 gene	-2.53
ENSMUSG00000089829	Gm16565	predicted gene 16565	-2.53
ENSMUSG00000003418	St8sia6	ST8 alpha-N-acetyl-neuraminide alpha-2,8-sialyltransferase 6	-2.53
ENSMUSG00000039330	Tsga10ip	testis specific 10 interacting protein	-2.54
ENSMUSG00000020589	Fam49a	family with sequence similarity 49, member A	-2.55
ENSMUSG00000074899	Sptbn5	spectrin beta, non-erythrocytic 5	-2.55
ENSMUSG00000097391	Mirg	miRNA containing gene	-2.55
ENSMUSG00000047250	Ptgs1	prostaglandin-endoperoxide synthase 1	-2.55
ENSMUSG00000030110	Ret	ret proto-oncogene	-2.56
ENSMUSG00000000632	Sez6	seizure related gene 6	-2.56
ENSMUSG00000048965	Mrgpre	MAS-related GPR, member E	-2.57
ENSMUSG00000050321	Neto1	neuropilin (NRP) and tolloid (TLL)-like 1	-2.57
ENSMUSG00000006457	Actn3	actinin alpha 3	-2.57
ENSMUSG00000074274	D930028M14Rik	RIKEN cDNA D930028M14 gene	-2.57
ENSMUSG00000030866	Ern2	endoplasmic reticulum (ER) to nucleus signalling 2	-2.58
ENSMUSG00000038065	Mturn	maturin, neural progenitor differentiation regulator homolog (Xenopus)	-2.58
ENSMUSG00000057614	Gnai1	guanine nucleotide binding protein (G protein), alpha inhibiting 1	-2.58
ENSMUSG00000073608	Gal3st2c	galactose-3-O-sulfotransferase 2C	-2.58
ENSMUSG00000079502	Cfap77	cilia and flagella associated protein 77	-2.58
ENSMUSG00000053580	Tanc2	tetratricopeptide repeat, ankyrin repeat and coiled-coil containing 2	-2.59
ENSMUSG00000057897	Camk2b	calcium/calmodulin-dependent protein kinase II, beta	-2.59
ENSMUSG00000030772	Dkk3	dickkopf WNT signaling pathway inhibitor 3	-2.59
ENSMUSG00000111293	Gm34006	predicted gene, 34006	-2.59
ENSMUSG00000039954	Stk32a	serine/threonine kinase 32A	-2.59
ENSMUSG00000024215	Spdef	SAM pointed domain containing ets transcription factor	-2.60
ENSMUSG00000004709	Cd244a	CD244 molecule A	-2.60
ENSMUSG00000108688	Gm44985	predicted gene 44985	-2.61
ENSMUSG00000029279	Brdt	bromodomain, testis-specific	-2.61
ENSMUSG00000073295	Nudt11	nudix (nucleoside diphosphate linked moiety X)-type motif 11	-2.61
ENSMUSG00000044288	Cnr1	cannabinoid receptor 1 (brain)	-2.62
ENSMUSG00000006362	Cbfa2t3	core-binding factor, runt domain, alpha subunit 2, translocated to, 3 (human)	-2.63
ENSMUSG00000020581	Agr2	anterior gradient 2	-2.63
ENSMUSG00000073043	Atoh1	atonal bHLH transcription factor 1	-2.63
ENSMUSG00000001281	Itgb7	integrin beta 7	-2.63
ENSMUSG00000042763	Maneal	mannosidase, endo-alpha-like	-2.63
ENSMUSG00000112197	Gm47337	predicted gene, 47337	-2.63
ENSMUSG00000110394	Gm18991	predicted gene, 18991	-2.63
ENSMUSG00000025701	Alox5	arachidonate 5-lipoxygenase	-2.64
ENSMUSG00000092517	Art2a-ps	ADP-ribosyltransferase 2a, pseudogene	-2.64
ENSMUSG00000051652	Lrrc3	leucine rich repeat containing 3	-2.64
ENSMUSG00000005045	Chd5	chromodomain helicase DNA binding protein 5	-2.65
ENSMUSG00000032085	Tagln	transgelin	-2.65
ENSMUSG00000086219	Srrm4os	serine/arginine repetitive matrix 4, opposite strand	-2.65
ENSMUSG00000032816	Igdcc4	immunoglobulin superfamily, DCC subclass, member 4	-2.66
ENSMUSG00000058488	Kl	klotho	-2.66
ENSMUSG00000050395	Tnfsf15	tumor necrosis factor (ligand) superfamily, member 15	-2.67
ENSMUSG00000036862	Dchs1	dachsous cadherin related 1	-2.67
ENSMUSG00000020788	Atp2a3	ATPase, Ca++ transporting, ubiquitous	-2.68
ENSMUSG00000021730	Hcn1	hyperpolarization-activated, cyclic nucleotide-gated K+ 1	-2.68
ENSMUSG00000008153	Clstn3	calsyntenin 3	-2.68
ENSMUSG00000019890	Nts	neurotensin	-2.68
ENSMUSG00000031442	Mcf2l	mcf.2 transforming sequence-like	-2.70
ENSMUSG00000022469	Rapgef3	Rap guanine nucleotide exchange factor (GEF) 3	-2.70
ENSMUSG00000038517	Tbkbp1	TBK1 binding protein 1	-2.70

ENSMUSG00000015222	Map2	microtubule-associated protein 2	-2.71
ENSMUSG00000046999	1110032F04Rik	RIKEN cDNA 1110032F04 gene	-2.71
ENSMUSG00000045215	Asxl3	additional sex combs like 3, transcriptional regulator	-2.71
ENSMUSG00000110266	Gm32742	predicted gene, 32742	-2.72
ENSMUSG00000021013	Ttc8	tetratricopeptide repeat domain 8	-2.72
ENSMUSG00000085189	Gm11963	predicted gene 11963	-2.72
ENSMUSG00000047298	Kcnv2	potassium channel, subfamily V, member 2	-2.72
ENSMUSG00000001670	Tat	tyrosine aminotransferase	-2.73
ENSMUSG00000038349	Plcl1	phospholipase C-like 1	-2.73
ENSMUSG00000051177	Plcb1	phospholipase C, beta 1	-2.74
ENSMUSG00000062861	Zfp28	zinc finger protein 28	-2.74
ENSMUSG00000035551	Igfbpl1	insulin-like growth factor binding protein-like 1	-2.74
ENSMUSG00000053049	Gm15413	predicted gene 15413	-2.74
ENSMUSG00000032087	Dscaml1	DS cell adhesion molecule like 1	-2.74
ENSMUSG00000036533	Cdc42ep3	CDC42 effector protein (Rho GTPase binding) 3	-2.75
ENSMUSG00000019906	Lin7a	lin-7 homolog A (C. elegans)	-2.75
ENSMUSG00000037386	Rims2	regulating synaptic membrane exocytosis 2	-2.76
ENSMUSG00000102380	Gm38140	predicted gene, 38140	-2.77
ENSMUSG00000037159	Wee2	WEE1 homolog 2 (S. pombe)	-2.78
ENSMUSG00000023009	Nckap5l	NCK-associated protein 5-like	-2.79
ENSMUSG00000048070	Pirt	phosphoinositide-interacting regulator of transient receptor potential channels	-2.79
ENSMUSG00000035509	Fbxl21	F-box and leucine-rich repeat protein 21	-2.79
ENSMUSG00000031374	Zfp92	zinc finger protein 92	-2.80
ENSMUSG00000027199	Gatm	glycine amidinotransferase (L-arginine:glycine amidinotransferase)	-2.80
ENSMUSG00000034336	Ina	internexin neuronal intermediate filament protein, alpha	-2.81
ENSMUSG00000003992	Ssbp2	single-stranded DNA binding protein 2	-2.81
ENSMUSG00000061947	Serpina10	serine (or cysteine) peptidase inhibitor, clade A (alpha-1 antiproteinase, antitrypsin	-2.81
ENSMUSG00000037259	Dzank1	double zinc ribbon and ankyrin repeat domains 1	-2.82
ENSMUSG00000049281	Scn3b	sodium channel, voltage-gated, type III, beta	-2.82
ENSMUSG00000026828	Galnt5	polypeptide N-acetylgalactosaminyltransferase 5	-2.83
ENSMUSG00000034958	Atcay	ataxia, cerebellar, Cayman type	-2.83
ENSMUSG00000049044	Rapgef4	Rap guanine nucleotide exchange factor (GEF) 4	-2.84
ENSMUSG00000035200	Chrnb4	cholinergic receptor, nicotinic, beta polypeptide 4	-2.85
ENSMUSG00000037973	Ccdc129	coiled-coil domain containing 129	-2.85
ENSMUSG00000028626	Col9a2	collagen, type IX, alpha 2	-2.87
ENSMUSG00000047238	Mageh1	melanoma antigen, family H, 1	-2.88
ENSMUSG00000035095	Fam167a	family with sequence similarity 167, member A	-2.88
ENSMUSG00000025584	Pde8a	phosphodiesterase 8A	-2.89
ENSMUSG00000034648	Lrrn1	leucine rich repeat protein 1, neuronal	-2.89
ENSMUSG00000059136	Olfr539	olfactory receptor 539	-2.89
ENSMUSG00000005503	Evx1	even-skipped homeobox 1	-2.90
ENSMUSG00000063919	Srrm4	serine/arginine repetitive matrix 4	-2.90
ENSMUSG00000026482	Rgl1	ral guanine nucleotide dissociation stimulator,-like 1	-2.90
ENSMUSG00000112816	Gm48798	predicted gene, 48798	-2.90
ENSMUSG00000111014	Gm47795	predicted gene, 47795	-2.90
ENSMUSG00000045382	Cxcr4	chemokine (C-X-C motif) receptor 4	-2.91
ENSMUSG00000017446	C1qtnf1	C1q and tumor necrosis factor related protein 1	-2.91
ENSMUSG00000031845	Bco1	beta-carotene oxygenase 1	-2.92
ENSMUSG00000037868	Egr2	early growth response 2	-2.92
ENSMUSG00000014602	Kif1a	kinesin family member 1A	-2.92
ENSMUSG00000036915	Kirrel2	kirre like nephrin family adhesion molecule 2	-2.92
ENSMUSG00000044468	Tent5c	terminal nucleotidyltransferase 5C	-2.93
ENSMUSG00000058420	Syt17	synaptotagmin XVII	-2.93
ENSMUSG00000003545	Fosb	FBJ osteosarcoma oncogene B	-2.94
ENSMUSG00000103732	Gm38315	predicted gene, 38315	-2.94
ENSMUSG00000030664	Sox6os	SRY (sex determining region Y)-box 6, opposite strand	-2.94
ENSMUSG00000020019	Ntn4	netrin 4	-2.95
ENSMUSG00000029343	Crybb1	crystallin, beta B1	-2.95
ENSMUSG00000005973	Rcn1	reticulocalbin 1	-2.96
ENSMUSG00000025091	Pnliprp2	pancreatic lipase-related protein 2	-2.96
ENSMUSG00000094818	Defa32	defensin, alpha, 32	-2.96
ENSMUSG00000028600	Podn	podocan	-2.97
ENSMUSG00000086126	Evx1os	even skipped homeotic gene 1, opposite strand	-2.97
ENSMUSG00000032773	Chrm1	cholinergic receptor, muscarinic 1, CNS	-2.98
ENSMUSG00000102581	Gm37443	predicted gene, 37443	-2.99
ENSMUSG00000030401	Rtn2	reticulon 2 (Z-band associated protein)	-3.00
ENSMUSG00000032015	Pou2f3	POU domain, class 2, transcription factor 3	-3.00

ENSMUSG00000051251	Nhlh1	nescient helix loop helix 1	-3.00
ENSMUSG00000064043	Trerf1	transcriptional regulating factor 1	-3.01
ENSMUSG00000046159	Chrm3	cholinergic receptor, muscarinic 3, cardiac	-3.03
ENSMUSG00000075702	Selenom	selenoprotein M	-3.04
ENSMUSG00000003436	Dll3	delta like canonical Notch ligand 3	-3.04
ENSMUSG00000087326	Gm12503	predicted gene 12503	-3.04
ENSMUSG00000049515	Espnl	espin-like	-3.04
ENSMUSG00000019775	Rgs17	regulator of G-protein signaling 17	-3.04
ENSMUSG00000021719	Rgs7bp	regulator of G-protein signalling 7 binding protein	-3.05
ENSMUSG00000064262	Gimap8	GTPase, IMAP family member 8	-3.05
ENSMUSG00000044043	Pcdhb14	protocadherin beta 14	-3.05
ENSMUSG00000034771	Tle2	transducin-like enhancer of split 2	-3.05
ENSMUSG00000035158	Mitf	melanogenesis associated transcription factor	-3.05
ENSMUSG00000052087	Rgs14	regulator of G-protein signaling 14	-3.06
ENSMUSG00000024238	Zeb1	zinc finger E-box binding homeobox 1	-3.06
ENSMUSG00000085329	2810404F17Rik	RIKEN cDNA 2810404F17 gene	-3.07
ENSMUSG00000078670	Fam174b	family with sequence similarity 174, member B	-3.08
ENSMUSG00000038594	Cep85l	centrosomal protein 85-like	-3.08
ENSMUSG00000042846	Lrrtm3	leucine rich repeat transmembrane neuronal 3	-3.08
ENSMUSG00000017211	Gsdma2	gasdermin A2	-3.09
ENSMUSG00000032452	Clstn2	calsyntenin 2	-3.09
ENSMUSG00000028364	Tnc	tenascin C	-3.09
ENSMUSG00000036764	Dnajc12	DnaJ heat shock protein family (Hsp40) member C12	-3.10
ENSMUSG00000036872	Abcc12	ATP-binding cassette, sub-family C (CFTR/MRP), member 12	-3.10
ENSMUSG00000071424	Grid2	glutamate receptor, ionotropic, delta 2	-3.10
ENSMUSG00000035864	Syt1	synaptotagmin I	-3.10
ENSMUSG00000039601	Rcan2	regulator of calcineurin 2	-3.11
ENSMUSG00000038319	Kcnh2	potassium voltage-gated channel, subfamily H (eag-related), member 2	-3.12
ENSMUSG00000096146	Kcnj11	potassium inwardly rectifying channel, subfamily J, member 11	-3.12
ENSMUSG00000033510	Otud7a	OTU domain containing 7A	-3.13
ENSMUSG00000039013	Siglecf	sialic acid binding Ig-like lectin F	-3.14
ENSMUSG00000010064	Slc38a3	solute carrier family 38, member 3	-3.14
ENSMUSG00000032936	Camkv	CaM kinase-like vesicle-associated	-3.15
ENSMUSG00000099148	Gm3331	predicted gene 3331	-3.15
ENSMUSG00000001333	Sync	syncoilin	-3.16
ENSMUSG00000026764	Kif5c	kinesin family member 5C	-3.16
ENSMUSG00000042757	Tmem108	transmembrane protein 108	-3.16
ENSMUSG00000095079	Igha	immunoglobulin heavy constant alpha	-3.16
ENSMUSG00000043391	2510009E07Rik	RIKEN cDNA 2510009E07 gene	-3.17
ENSMUSG00000056812	St8sia3	ST8 alpha-N-acetyl-neuraminide alpha-2,8-sialyltransferase 3	-3.17
ENSMUSG00000106547	B230303O12Rik	RIKEN cDNA B230303O12 gene	-3.17
ENSMUSG00000113502	Gm47411	predicted gene, 47411	-3.17
ENSMUSG00000022441	Efcab6	EF-hand calcium binding domain 6	-3.17
ENSMUSG00000066191	Anks6	ankyrin repeat and sterile alpha motif domain containing 6	-3.18
ENSMUSG00000072623	Zfp9	zinc finger protein 9	-3.18
ENSMUSG00000025876	Unc5a	unc-5 netrin receptor A	-3.19
ENSMUSG00000090105	Gm15890	predicted gene 15890	-3.19
ENSMUSG00000102498	Gm19445	predicted gene, 19445	-3.19
ENSMUSG00000000214	Th	tyrosine hydroxylase	-3.20
ENSMUSG00000023247	Guca2a	guanylate cyclase activator 2a (guanylin)	-3.21
ENSMUSG00000038486	Sv2a	synaptic vesicle glycoprotein 2 a	-3.21
ENSMUSG00000063704	Mapk15	mitogen-activated protein kinase 15	-3.22
ENSMUSG00000035459	Stab2	stabilin 2	-3.22
ENSMUSG00000038599	Capn8	calpain 8	-3.23
ENSMUSG00000060671	Atp8b2	ATPase, class I, type 8B, member 2	-3.25
ENSMUSG00000033220	Rac2	Rac family small GTPase 2	-3.25
ENSMUSG00000018470	Kcnab3	potassium voltage-gated channel, shaker-related subfamily, beta member 3	-3.26
ENSMUSG00000030616	Sytl2	synaptotagmin-like 2	-3.27
ENSMUSG00000079037	Prnp	prion protein	-3.27
ENSMUSG00000044548	Dact1	dishevelled-binding antagonist of beta-catenin 1	-3.27
ENSMUSG00000086141	9030622O22Rik	RIKEN cDNA 9030622O22 gene	-3.27
ENSMUSG00000042515	Mum1l1	melanoma associated antigen (mutated) 1-like 1	-3.27
ENSMUSG00000114985	1810028F09Rik	RIKEN cDNA 1810028F09 gene	-3.28
ENSMUSG00000040146	Rgl3	ral guanine nucleotide dissociation stimulator-like 3	-3.29
ENSMUSG00000086231	Rapgef4os3	Rap guanine nucleotide exchange factor (GEF) 4, opposite strand 3	-3.29
ENSMUSG00000074436	Defa-ps18	defensin, alpha, pseudogene 18	-3.29
ENSMUSG00000029134	Plb1	phospholipase B1	-3.30

ENSMUSG00000041673	Lrrc18	leucine rich repeat containing 18	-3.30
ENSMUSG00000026950	Neb	nebulin	-3.31
ENSMUSG00000013846	St3gal1	ST3 beta-galactoside alpha-2,3-sialyltransferase 1	-3.31
ENSMUSG00000013523	Bcas1	breast carcinoma amplified sequence 1	-3.31
ENSMUSG00000038132	Rbm24	RNA binding motif protein 24	-3.31
ENSMUSG00000048763	Hoxb3	homeobox B3	-3.32
ENSMUSG00000107061	Gm19590	predicted gene, 19590	-3.32
ENSMUSG00000024743	Syt7	synaptotagmin VII	-3.33
ENSMUSG00000015968	Cacna1d	calcium channel, voltage-dependent, L type, alpha 1D subunit	-3.33
ENSMUSG00000113680	Gm47405	predicted gene, 47405	-3.34
ENSMUSG00000038738	Shank1	SH3 and multiple ankyrin repeat domains 1	-3.34
ENSMUSG00000060735	Rxfp3	relaxin family peptide receptor 3	-3.34
ENSMUSG00000061414	Cracr2a	calcium release activated channel regulator 2A	-3.35
ENSMUSG00000024548	Setbp1	SET binding protein 1	-3.35
ENSMUSG00000040528	Milr1	mast cell immunoglobulin like receptor 1	-3.35
ENSMUSG00000037106	Fer1l6	fer-1-like 6 (C. elegans)	-3.36
ENSMUSG00000034818	Celf5	CUGBP, Elav-like family member 5	-3.36
ENSMUSG00000020340	Cyfip2	cytoplasmic FMR1 interacting protein 2	-3.37
ENSMUSG00000086245	Gm16170	predicted gene 16170	-3.37
ENSMUSG00000031510	1700128E19Rik	RIKEN cDNA 1700128E19 gene	-3.37
ENSMUSG00000032502	Stac	src homology three (SH3) and cysteine rich domain	-3.38
ENSMUSG00000018486	Wnt9b	wingless-type MMTV integration site family, member 9B	-3.38
ENSMUSG00000026475	Rgs16	regulator of G-protein signaling 16	-3.38
ENSMUSG00000074344	Tmigd3	transmembrane and immunoglobulin domain containing 3	-3.38
ENSMUSG00000028528	Dnajc6	DnaJ heat shock protein family (Hsp40) member C6	-3.38
ENSMUSG00000032202	Rab27a	RAB27A, member RAS oncogene family	-3.39
ENSMUSG00000050821	Fam131a	family with sequence similarity 131, member A	-3.39
ENSMUSG00000026285	Pdcd1	programmed cell death 1	-3.39
ENSMUSG00000016763	Scube1	signal peptide, CUB domain, EGF-like 1	-3.40
ENSMUSG00000036306	Lzts1	leucine zipper, putative tumor suppressor 1	-3.40
ENSMUSG00000023868	Pde10a	phosphodiesterase 10A	-3.40
ENSMUSG00000057716	Tmem178b	transmembrane protein 178B	-3.40
ENSMUSG00000020218	Wif1	Wnt inhibitory factor 1	-3.40
ENSMUSG00000042078	Svop	SV2 related protein	-3.40
ENSMUSG00000021609	Slc6a3	solute carrier family 6 (neurotransmitter transporter, dopamine), member 3	-3.41
ENSMUSG00000059588	Calcrl	calcitonin receptor-like	-3.41
ENSMUSG00000033900	Map9	microtubule-associated protein 9	-3.43
ENSMUSG00000093805	Gal3st2b	galactose-3-O-sulfotransferase 2B	-3.43
ENSMUSG00000091017	Fam71a	family with sequence similarity 71, member A	-3.43
ENSMUSG00000072966	Gprasp2	G protein-coupled receptor associated sorting protein 2	-3.43
ENSMUSG00000040121	Rep15	RAB15 effector protein	-3.44
ENSMUSG00000037239	Spred3	sprouty-related, EVH1 domain containing 3	-3.44
ENSMUSG00000026288	Inpp5d	inositol polyphosphate-5-phosphatase D	-3.44
ENSMUSG00000007594	Hapln4	hyaluronan and proteoglycan link protein 4	-3.44
ENSMUSG00000084048	Gm12042	predicted gene 12042	-3.44
ENSMUSG00000044748	Defb1	defensin beta 1	-3.45
ENSMUSG00000000197	Nalcn	sodium leak channel, non-selective	-3.45
ENSMUSG00000027661	Slc2a10	solute carrier family 2 (facilitated glucose transporter), member 10	-3.46
ENSMUSG00000032826	Ank2	ankyrin 2, brain	-3.46
ENSMUSG00000000125	Wnt3	wingless-type MMTV integration site family, member 3	-3.47
ENSMUSG00000026463	Atp2b4	ATPase, Ca++ transporting, plasma membrane 4	-3.47
ENSMUSG00000047730	Fcgbp	Fc fragment of IgG binding protein	-3.47
ENSMUSG00000030087	Klf15	Kruppel-like factor 15	-3.47
ENSMUSG00000034460	Six4	sine oculis-related homeobox 4	-3.47
ENSMUSG00000018593	Sparc	secreted acidic cysteine rich glycoprotein	-3.48
ENSMUSG00000085247	4930545L23Rik	RIKEN cDNA 4930545L23 gene	-3.48
ENSMUSG00000021559	Dapk1	death associated protein kinase 1	-3.49
ENSMUSG00000023032	Slc4a8	solute carrier family 4 (anion exchanger), member 8	-3.49
ENSMUSG00000021614	Vcan	versican	-3.50
ENSMUSG00000098557	Kctd12	potassium channel tetramerisation domain containing 12	-3.51
ENSMUSG00000020102	Slc16a7	solute carrier family 16 (monocarboxylic acid transporters), member 7	-3.51
ENSMUSG00000042804	Gpr153	G protein-coupled receptor 153	-3.51
ENSMUSG00000022456	37865	septin 3	-3.52
ENSMUSG00000041681	Iapp	islet amyloid polypeptide	-3.52
ENSMUSG00000010086	Rnf112	ring finger protein 112	-3.52
ENSMUSG00000035896	Rnase1	ribonuclease, RNase A family, 1 (pancreatic)	-3.53
ENSMUSG00000029875	Ccdc184	coiled-coil domain containing 184	-3.53

ENSMUSG00000070576	Mn1	meningioma 1	-3.53
ENSMUSG00000025272	Tro	trophinin	-3.54
ENSMUSG00000030428	Ttyh1	tweety family member 1	-3.55
ENSMUSG00000015484	Fam163a	family with sequence similarity 163, member A	-3.55
ENSMUSG00000013921	Clip3	CAP-GLY domain containing linker protein 3	-3.55
ENSMUSG00000078776	9530053A07Rik	RIKEN cDNA 9530053A07 gene	-3.56
ENSMUSG00000089995	Gm15716	predicted gene 15716	-3.56
ENSMUSG00000032268	Tmprss5	transmembrane protease, serine 5 (spinesin)	-3.56
ENSMUSG00000024330	Col11a2	collagen, type XI, alpha 2	-3.57
ENSMUSG00000026834	Acvr1c	activin A receptor, type IC	-3.57
ENSMUSG00000049047	Armcx3	armadillo repeat containing, X-linked 3	-3.57
ENSMUSG00000030411	Nova2	neuro-oncological ventral antigen 2	-3.57
ENSMUSG00000032035	Ets1	E26 avian leukemia oncogene 1, 5' domain	-3.58
ENSMUSG00000024029	Tff3	trefoil factor 3, intestinal	-3.59
ENSMUSG00000080715	Gm5406	predicted gene 5406	-3.59
ENSMUSG00000036912	Piwil4	piwi-like RNA-mediated gene silencing 4	-3.59
ENSMUSG00000059921	Unc5c	unc-5 netrin receptor C	-3.59
ENSMUSG00000034107	Ano7	anoctamin 7	-3.60
ENSMUSG00000018927	Ccl6	chemokine (C-C motif) ligand 6	-3.60
ENSMUSG00000043518	Rai2	retinoic acid induced 2	-3.60
ENSMUSG00000047642	D930020B18Rik	RIKEN cDNA D930020B18 gene	-3.60
ENSMUSG00000055963	Triqk	triple QxxK/R motif containing	-3.61
ENSMUSG00000001120	Pcbp3	poly(rC) binding protein 3	-3.62
ENSMUSG00000024897	Apba1	amyloid beta (A4) precursor protein binding, family A, member 1	-3.62
ENSMUSG00000031028	Tub	tubby bipartite transcription factor	-3.62
ENSMUSG00000042179	Pnliprp1	pancreatic lipase related protein 1	-3.63
ENSMUSG00000022425	Enpp2	ectonucleotide pyrophosphatase/phosphodiesterase 2	-3.63
ENSMUSG00000020704	Asic2	acid-sensing (proton-gated) ion channel 2	-3.63
ENSMUSG00000001827	Folr1	folate receptor 1 (adult)	-3.63
ENSMUSG00000032744	Heyl	hairy/enhancer-of-split related with YRPW motif-like	-3.63
ENSMUSG00000104283	Gm37459	predicted gene, 37459	-3.63
ENSMUSG00000035964	Tmem59l	transmembrane protein 59-like	-3.64
ENSMUSG00000028445	Enho	energy homeostasis associated	-3.64
ENSMUSG00000053552	Ebf4	early B cell factor 4	-3.64
ENSMUSG00000036330	Slc18a1	solute carrier family 18 (vesicular monoamine), member 1	-3.65
ENSMUSG00000086999	Bcas1os2	breast carcinoma amplified sequence 1, opposite strand 2	-3.65
ENSMUSG00000040136	Abcc8	ATP-binding cassette, sub-family C (CFTR/MRP), member 8	-3.66
ENSMUSG00000032064	Dixdc1	DIX domain containing 1	-3.67
ENSMUSG00000001802	Lrp3	low density lipoprotein receptor-related protein 3	-3.67
ENSMUSG00000031428	Zcchc18	zinc finger, CCHC domain containing 18	-3.67
ENSMUSG00000020120	Plek	pleckstrin	-3.67
ENSMUSG00000024598	Fbn2	fibrillin 2	-3.68
ENSMUSG00000054169	Ceacam10	carcinoembryonic antigen-related cell adhesion molecule 10	-3.69
ENSMUSG00000015396	Cd83	CD83 antigen	-3.69
ENSMUSG00000040563	Plppr2	phospholipid phosphatase related 2	-3.69
ENSMUSG00000003283	Hck	hemopoietic cell kinase	-3.69
ENSMUSG00000097311	Gm26871	predicted gene, 26871	-3.69
ENSMUSG00000020181	Nav3	neuron navigator 3	-3.70
ENSMUSG00000031760	Mt3	metallothionein 3	-3.70
ENSMUSG00000020601	Trib2	tribbles pseudokinase 2	-3.70
ENSMUSG00000034472	Rasd2	RASD family, member 2	-3.71
ENSMUSG00000028749	Pla2g2f	phospholipase A2, group IIF	-3.72
ENSMUSG00000018862	Otop3	otopetrin 3	-3.72
ENSMUSG00000027520	Zdbf2	zinc finger, DBF-type containing 2	-3.72
ENSMUSG00000004961	Syt5	synaptotagmin V	-3.73
ENSMUSG00000056004	9330182L06Rik	RIKEN cDNA 9330182L06 gene	-3.73
ENSMUSG00000044017	Adgrd1	adhesion G protein-coupled receptor D1	-3.73
ENSMUSG00000044312	Neurog3	neurogenin 3	-3.73
ENSMUSG00000035513	Ntng2	netrin G2	-3.73
ENSMUSG00000032377	Plscr4	phospholipid scramblase 4	-3.73
ENSMUSG00000028354	Fmn2	formin 2	-3.73
ENSMUSG00000053182	Gm609	predicted gene 609	-3.74
ENSMUSG00000025075	Habp2	hyaluronic acid binding protein 2	-3.74
ENSMUSG00000027674	Pex5l	peroxisomal biogenesis factor 5-like	-3.74
ENSMUSG00000021587	Pcsk1	proprotein convertase subtilisin/kexin type 1	-3.75
ENSMUSG00000064125	Prr36	proline rich 36	-3.75
ENSMUSG00000026870	Cutal	cutA divalent cation tolerance homolog-like	-3.75

ENSMUSG00000021539	Lect2	leukocyte cell-derived chemotaxin 2	-3.76
ENSMUSG00000054453	Sytl5	synaptotagmin-like 5	-3.76
ENSMUSG00000018169	Mfng	MFNG O-fucosylpeptide 3-beta-N-acetylglucosaminyltransferase	-3.76
ENSMUSG00000047867	Gimap6	GTPase, IMAP family member 6	-3.76
ENSMUSG00000031377	Bmx	BMX non-receptor tyrosine kinase	-3.77
ENSMUSG00000022829	Stxbp5l	syntaxin binding protein 5-like	-3.77
ENSMUSG00000009246	Trpm5	transient receptor potential cation channel, subfamily M, member 5	-3.78
ENSMUSG00000062444	Ap3b2	adaptor-related protein complex 3, beta 2 subunit	-3.78
ENSMUSG00000037035	Inhbb	inhibin beta-B	-3.78
ENSMUSG00000072875	Gpr27	G protein-coupled receptor 27	-3.78
ENSMUSG00000025551	Fgf14	fibroblast growth factor 14	-3.78
ENSMUSG00000020435	Osbp2	oxysterol binding protein 2	-3.79
ENSMUSG00000029223	Uchl1	ubiquitin carboxy-terminal hydrolase L1	-3.79
ENSMUSG00000101126	Gm10538	predicted gene 10538	-3.79
ENSMUSG00000040170	Fmo2	flavin containing monooxygenase 2	-3.80
ENSMUSG00000037579	Kcnh3	potassium voltage-gated channel, subfamily H (eag-related), member 3	-3.81
ENSMUSG00000069171	Nr2f1	nuclear receptor subfamily 2, group F, member 1	-3.81
ENSMUSG00000022995	Enah	ENAH actin regulator	-3.82
ENSMUSG00000037306	Man1c1	mannosidase, alpha, class 1C, member 1	-3.82
ENSMUSG00000019122	Ccl9	chemokine (C-C motif) ligand 9	-3.83
ENSMUSG00000020101	Vsir	V-set immunoregulatory receptor	-3.83
ENSMUSG00000020312	Shc2	SHC (Src homology 2 domain containing) transforming protein 2	-3.84
ENSMUSG00000027947	Il6ra	interleukin 6 receptor, alpha	-3.84
ENSMUSG00000080115	Eef1akmt3	EEF1A lysine methyltransferase 3	-3.84
ENSMUSG00000017692	Rhbdl3	rhomboid like 3	-3.84
ENSMUSG00000048216	Gpr85	G protein-coupled receptor 85	-3.84
ENSMUSG00000102123	Gm4319	predicted gene 4319	-3.84
ENSMUSG00000038916	Soga3	SOGA family member 3	-3.85
ENSMUSG00000034799	Unc13a	unc-13 homolog A	-3.86
ENSMUSG00000046922	Gpr6	G protein-coupled receptor 6	-3.86
ENSMUSG00000081303	Gm16011	predicted gene 16011	-3.87
ENSMUSG00000051832	E230016K23Rik	RIKEN cDNA E230016K23 gene	-3.87
ENSMUSG00000027249	F2	coagulation factor II	-3.87
ENSMUSG00000038665	Dgki	diacylglycerol kinase, iota	-3.87
ENSMUSG00000060063	Alox5ap	arachidonate 5-lipoxygenase activating protein	-3.87
ENSMUSG00000005883	Spo11	SPO11 meiotic protein covalently bound to DSB	-3.87
ENSMUSG00000074442	Defa31	defensin, alpha, 31	-3.87
ENSMUSG00000055733	Nap1l3	nucleosome assembly protein 1-like 3	-3.87
ENSMUSG00000022148	Fyb	FYN binding protein	-3.88
ENSMUSG00000031551	Ido1	indoleamine 2,3-dioxygenase 1	-3.88
ENSMUSG00000037610	Kcnmb2	potassium large conductance calcium-activated channel, subfamily M, beta membe	-3.88
ENSMUSG00000040148	Hmx3	H6 homeobox 3	-3.89
ENSMUSG00000052271	Bhlha15	basic helix-loop-helix family, member a15	-3.90
ENSMUSG00000033615	Cplx1	complexin 1	-3.91
ENSMUSG00000114751	5430425E15Rik	RIKEN cDNA 5430425E15 gene	-3.91
ENSMUSG00000063903	Klk1	kallikrein 1	-3.92
ENSMUSG00000024030	Abcg1	ATP binding cassette subfamily G member 1	-3.92
ENSMUSG00000043388	Tmem130	transmembrane protein 130	-3.92
ENSMUSG00000001985	Grik3	glutamate receptor, ionotropic, kainate 3	-3.92
ENSMUSG00000031981	Capn9	calpain 9	-3.95
ENSMUSG00000042115	Klhdc8a	kelch domain containing 8A	-3.95
ENSMUSG00000044933	Sstr3	somatostatin receptor 3	-3.96
ENSMUSG00000057914	Cacnb2	calcium channel, voltage-dependent, beta 2 subunit	-3.96
ENSMUSG00000074434	Defa28	defensin, alpha, 28	-3.96
ENSMUSG00000027570	Col9a3	collagen, type IX, alpha 3	-3.97
ENSMUSG00000024013	Fgd2	FYVE, RhoGEF and PH domain containing 2	-3.97
ENSMUSG00000028255	Clca1	chloride channel accessory 1	-3.98
ENSMUSG00000052212	Cd177	CD177 antigen	-3.99
ENSMUSG00000057315	Arhgap24	Rho GTPase activating protein 24	-3.99
ENSMUSG00000060615	Ang4	angiogenin, ribonuclease A family, member 4	-3.99
ENSMUSG00000058589	Anks1b	ankyrin repeat and sterile alpha motif domain containing 1B	-3.99
ENSMUSG00000028865	Cd164l2	CD164 sialomucin-like 2	-3.99
ENSMUSG00000022211	Carmil3	capping protein regulator and myosin 1 linker 3	-4.00
ENSMUSG00000097266	Gm26802	predicted gene, 26802	-4.00
ENSMUSG00000028078	Dclk2	doublecortin-like kinase 2	-4.01
ENSMUSG00000029245	Epha5	Eph receptor A5	-4.01
ENSMUSG00000038112	AW551984	expressed sequence AW551984	-4.01

ENSMUSG00000070687	Htr1d	5-hydroxytryptamine (serotonin) receptor 1D	-4.01
ENSMUSG00000041794	Myrip	myosin VIIA and Rab interacting protein	-4.02
ENSMUSG00000024011	Pi16	peptidase inhibitor 16	-4.02
ENSMUSG00000071550	Cfap44	cilia and flagella associated protein 44	-4.03
ENSMUSG00000029368	Alb	albumin	-4.03
ENSMUSG00000019232	Etnppl	ethanolamine phosphate phospholyase	-4.04
ENSMUSG00000042766	Trim46	tripartite motif-containing 46	-4.04
ENSMUSG00000021087	Rtn1	reticulon 1	-4.04
ENSMUSG00000041544	Disp3	dispatched RND transporter family member 3	-4.04
ENSMUSG00000079481	Nhsl2	NHS-like 2	-4.05
ENSMUSG00000069515	Lyz1	lysozyme 1	-4.06
ENSMUSG00000042873	Lhfpl4	lipoma HMGIC fusion partner-like protein 4	-4.08
ENSMUSG00000030074	Gxylt2	glucoside xylosyltransferase 2	-4.08
ENSMUSG00000030302	Atp2b2	ATPase, Ca++ transporting, plasma membrane 2	-4.09
ENSMUSG00000084934	Gm16035	predicted gene 16035	-4.09
ENSMUSG00000040723	Rcsd1	RCSD domain containing 1	-4.10
ENSMUSG00000038580	Sct	secretin	-4.11
ENSMUSG00000031980	Agt	angiotensinogen (serpin peptidase inhibitor, clade A, member 8)	-4.11
ENSMUSG00000044177	Wfikkn2	WAP, follistatin/kazal, immunoglobulin, kunitz and netrin domain containing 2	-4.11
ENSMUSG00000021069	Pygl	liver glycogen phosphorylase	-4.11
ENSMUSG00000040412	5330417C22Rik	RIKEN cDNA 5330417C22 gene	-4.12
ENSMUSG00000019846	Lama4	laminin, alpha 4	-4.12
ENSMUSG00000024084	Qpct	glutaminyl-peptide cyclotransferase (glutaminyl cyclase)	-4.12
ENSMUSG00000097520	4930488L21Rik	RIKEN cDNA 4930488L21 gene	-4.12
ENSMUSG00000016200	Syt14	synaptotagmin XIV	-4.13
ENSMUSG00000034353	Ramp1	receptor (calcitonin) activity modifying protein 1	-4.13
ENSMUSG00000025582	Nptx1	neuronal pentraxin 1	-4.13
ENSMUSG00000022650	Retnlb	resistin like beta	-4.14
ENSMUSG00000033200	Tpsg1	tryptase gamma 1	-4.14
ENSMUSG00000114501	Gm48582	predicted gene, 48582	-4.14
ENSMUSG00000025867	Cplx2	complexin 2	-4.16
ENSMUSG00000031748	Gnao1	guanine nucleotide binding protein, alpha O	-4.16
ENSMUSG00000070337	Gpr179	G protein-coupled receptor 179	-4.16
ENSMUSG00000056296	Synpr	synaptoporin	-4.16
ENSMUSG00000026527	Rgs7	regulator of G protein signaling 7	-4.17
ENSMUSG00000022054	Nefm	neurofilament, medium polypeptide	-4.18
ENSMUSG00000099025	Gm27162	predicted gene 27162	-4.18
ENSMUSG00000020175	Rab36	RAB36, member RAS oncogene family	-4.18
ENSMUSG00000057060	Slc35f3	solute carrier family 35, member F3	-4.18
ENSMUSG00000071113	Mboat4	membrane bound O-acyltransferase domain containing 4	-4.19
ENSMUSG00000049148	Plcxd3	phosphatidylinositol-specific phospholipase C, X domain containing 3	-4.19
ENSMUSG00000091890	A830073O21Rik	RIKEN cDNA A830073O21 gene	-4.19
ENSMUSG00000024299	Adamts10	a disintegrin-like and metallopeptidase (reprolysin type) with thrombospondin type	-4.20
ENSMUSG00000054932	Afp	alpha fetoprotein	-4.20
ENSMUSG00000030518	Fam189a1	family with sequence similarity 189, member A1	-4.20
ENSMUSG00000023216	Epb42	erythrocyte membrane protein band 4.2	-4.21
ENSMUSG00000028415	Spink4	serine peptidase inhibitor, Kazal type 4	-4.21
ENSMUSG00000049122	Frmd3	FERM domain containing 3	-4.21
ENSMUSG00000044156	Hepacam2	HEPACAM family member 2	-4.22
ENSMUSG00000074446	Defa23	defensin, alpha, 23	-4.23
ENSMUSG00000032625	Thsd7a	thrombospondin, type I, domain containing 7A	-4.23
ENSMUSG00000033174	Mgll	monoglyceride lipase	-4.23
ENSMUSG00000029778	Adcyap1r1	adenylate cyclase activating polypeptide 1 receptor 1	-4.23
ENSMUSG00000036699	Zcchc12	zinc finger, CCHC domain containing 12	-4.23
ENSMUSG00000109561	Ankrd31	ankyrin repeat domain 31	-4.24
ENSMUSG00000027273	Snap25	synaptosomal-associated protein 25	-4.26
ENSMUSG00000004151	Etv1	ets variant 1	-4.27
ENSMUSG00000025515	Muc2	mucin 2	-4.28
ENSMUSG00000023473	Celsr3	cadherin, EGF LAG seven-pass G-type receptor 3	-4.29
ENSMUSG00000049350	Zg16	zymogen granule protein 16	-4.29
ENSMUSG00000106386	Gm42991	predicted gene 42991	-4.29
ENSMUSG00000044988	Ucn3	urocortin 3	-4.29
ENSMUSG00000025855	Prkar1b	protein kinase, cAMP dependent regulatory, type I beta	-4.30
ENSMUSG00000068617	Efcab1	EF-hand calcium binding domain 1	-4.30
ENSMUSG00000082211	Defa27	defensin, alpha, 27	-4.30
ENSMUSG00000051111	Sv2c	synaptic vesicle glycoprotein 2c	-4.30
ENSMUSG00000026817	Ak1	adenylate kinase 1	-4.30

ENSMUSG00000069516	Lyz2	lysozyme 2	-4.31
ENSMUSG00000034656	Cacna1a	calcium channel, voltage-dependent, P/Q type, alpha 1A subunit	-4.31
ENSMUSG00000062760	Shisal1	shisa like 1	-4.31
ENSMUSG00000038248	Sobp	sine oculis binding protein	-4.32
ENSMUSG00000038209	Itln1	intelectin 1 (galactofuranose binding)	-4.33
ENSMUSG00000015829	Tnr	tenascin R	-4.33
ENSMUSG00000048022	Tmem229a	transmembrane protein 229A	-4.34
ENSMUSG00000056973	Ces1d	carboxylesterase 1D	-4.34
ENSMUSG00000071226	Cecr2	CECR2, histone acetyl-lysine reader	-4.35
ENSMUSG00000103761	Gm37859	predicted gene, 37859	-4.35
ENSMUSG00000020717	Pecam1	platelet/endothelial cell adhesion molecule 1	-4.36
ENSMUSG00000046204	Pnma2	paraneoplastic antigen MA2	-4.36
ENSMUSG00000066176	Gm12511	predicted gene 12511	-4.36
ENSMUSG00000109305	1810010D01Rik	RIKEN cDNA 1810010D01 gene	-4.38
ENSMUSG00000069814	Ccdc92b	coiled-coil domain containing 92B	-4.39
ENSMUSG00000063142	Kcnma1	potassium large conductance calcium-activated channel, subfamily M, alpha memb	-4.40
ENSMUSG00000027797	Dclk1	doublecortin-like kinase 1	-4.40
ENSMUSG00000058297	Spock2	sparc/osteonectin, cwcv and kazal-like domains proteoglycan 2	-4.41
ENSMUSG00000029193	Cckar	cholecystokinin A receptor	-4.41
ENSMUSG00000020901	Pik3r5	phosphoinositide-3-kinase regulatory subunit 5	-4.42
ENSMUSG00000030263	Lrmp	lymphoid-restricted membrane protein	-4.42
ENSMUSG00000056752	Dnah9	dynein, axonemal, heavy chain 9	-4.42
ENSMUSG00000004707	Ly9	lymphocyte antigen 9	-4.42
ENSMUSG00000111443	Gm46123	predicted gene, 46123	-4.42
ENSMUSG00000086029	Pax6os1	paired box 6 opposite strand 1	-4.42
ENSMUSG00000087211	Lhx1os	LIM homeobox 1, opposite strand	-4.42
ENSMUSG00000051314	Ffar2	free fatty acid receptor 2	-4.43
ENSMUSG00000049154	Fam183b	family with sequence similarity 183, member B	-4.44
ENSMUSG00000015599	Ttbk1	tau tubulin kinase 1	-4.44
ENSMUSG00000033152	Podxl2	podocalyxin-like 2	-4.44
ENSMUSG00000002900	Lamb1	laminin B1	-4.45
ENSMUSG00000046460	Sh2d7	SH2 domain containing 7	-4.45
ENSMUSG00000018822	Sfrp5	secreted frizzled-related sequence protein 5	-4.46
ENSMUSG00000028926	Cdk14	cyclin-dependent kinase 14	-4.47
ENSMUSG00000040978	Gm11992	predicted gene 11992	-4.47
ENSMUSG00000061958	Gm14851	predicted gene 14851	-4.49
ENSMUSG00000025175	Fn3k	fructosamine 3 kinase	-4.49
ENSMUSG00000030020	Prickle2	prickle planar cell polarity protein 2	-4.49
ENSMUSG00000066705	Fxyd6	FXYD domain-containing ion transport regulator 6	-4.50
ENSMUSG00000004113	Cacna1b	calcium channel, voltage-dependent, N type, alpha 1B subunit	-4.51
ENSMUSG00000032297	Celf6	CUGBP, Elav-like family member 6	-4.51
ENSMUSG00000056492	Adgrf5	adhesion G protein-coupled receptor F5	-4.52
ENSMUSG00000024304	Cdh2	cadherin 2	-4.52
ENSMUSG00000106491	Gm42446	predicted gene 42446	-4.53
ENSMUSG00000050994	Adgb	androglobin	-4.53
ENSMUSG00000071178	Serpina1b	serine (or cysteine) preptidase inhibitor, clade A, member 1B	-4.55
ENSMUSG00000052572	Dlg2	discs large MAGUK scaffold protein 2	-4.55
ENSMUSG00000064213	Defa24	defensin, alpha, 24	-4.57
ENSMUSG00000052631	Sh2d6	SH2 domain containing 6	-4.57
ENSMUSG00000037627	Rgs22	regulator of G-protein signalling 22	-4.57
ENSMUSG00000029445	Hpd	4-hydroxyphenylpyruvic acid dioxygenase	-4.58
ENSMUSG00000002265	Peg3	paternally expressed 3	-4.58
ENSMUSG00000009394	Syn2	synapsin II	-4.58
ENSMUSG00000022449	Adamts20	a disintegrin-like and metallopeptidase (reprolysin type) with thrombospondin type	-4.58
ENSMUSG00000040653	Ppp1r14c	protein phosphatase 1, regulatory inhibitor subunit 14C	-4.58
ENSMUSG00000037953	A4gnt	alpha-1,4-N-acetylglucosaminyltransferase	-4.59
ENSMUSG00000074441	Gm15292	predicted gene 15292	-4.59
ENSMUSG00000010066	Cacna2d2	calcium channel, voltage-dependent, alpha 2/delta subunit 2	-4.59
ENSMUSG00000000031	H19	H19, imprinted maternally expressed transcript	-4.60
ENSMUSG00000023033	Scn8a	sodium channel, voltage-gated, type VIII, alpha	-4.60
ENSMUSG00000044807	Zfp354c	zinc finger protein 354C	-4.61
ENSMUSG00000032852	Rspo4	R-spondin 4	-4.62
ENSMUSG00000075517	Cyp2d37-ps	cytochrome P450, family 2, subfamily d, polypeptide 37, pseudogene	-4.62
ENSMUSG00000021071	Trim9	tripartite motif-containing 9	-4.63
ENSMUSG00000074440	Defa3	defensin, alpha, 3	-4.64
ENSMUSG00000000394	Gcg	glucagon	-4.65
ENSMUSG00000060070	Defa26	defensin, alpha, 26	-4.66

ENSMUSG00000074444	Defa30	defensin, alpha, 30	-4.66
ENSMUSG00000026247	Ecel1	endothelin converting enzyme-like 1	-4.66
ENSMUSG00000041078	Grid1	glutamate receptor, ionotropic, delta 1	-4.68
ENSMUSG00000074443	Defa22	defensin, alpha, 22	-4.69
ENSMUSG00000074437	Defa29	defensin, alpha, 29	-4.70
ENSMUSG00000019124	Scrn1	secernin 1	-4.71
ENSMUSG00000079015	Serpina1c	serine (or cysteine) peptidase inhibitor, clade A, member 1C	-4.71
ENSMUSG00000021647	Cartpt	CART prepropeptide	-4.73
ENSMUSG00000046215	Rprml	reprimo-like	-4.73
ENSMUSG00000060208	Defa17	defensin, alpha, 17	-4.74
ENSMUSG00000111977	Gm47163	predicted gene, 47163	-4.74
ENSMUSG00000094662	Defa36	defensin, alpha, 36	-4.74
ENSMUSG00000030683	Sez6l2	seizure related 6 homolog like 2	-4.75
ENSMUSG00000039809	Gabbr2	gamma-aminobutyric acid (GABA) B receptor, 2	-4.75
ENSMUSG00000074447	Defa21	defensin, alpha, 21	-4.75
ENSMUSG00000065956	Defa37	defensin, alpha, 37	-4.75
ENSMUSG00000097927	Gm6999	predicted gene 6999	-4.76
ENSMUSG00000027434	Nkx2-2	NK2 homeobox 2	-4.77
ENSMUSG00000028137	Celf3	CUGBP, Elav-like family member 3	-4.77
ENSMUSG00000040035	Disp2	dispatched RND tramsporter family member 2	-4.78
ENSMUSG00000024225	Clps	colipase, pancreatic	-4.78
ENSMUSG00000061911	Myt1l	myelin transcription factor 1-like	-4.79
ENSMUSG00000061845	Defa35	defensin, alpha, 35	-4.80
ENSMUSG00000064177	Ghrl	ghrelin	-4.82
ENSMUSG00000027254	Map1a	microtubule-associated protein 1 A	-4.82
ENSMUSG00000036913	Trim67	tripartite motif-containing 67	-4.82
ENSMUSG00000056069	Otulinl	OTU deubiquitinase with linear linkage specificity like	-4.83
ENSMUSG00000020787	P2rx1	purinergic receptor P2X, ligand-gated ion channel, 1	-4.83
ENSMUSG00000004347	Pde1c	phosphodiesterase 1C	-4.84
ENSMUSG00000063206	Defa34	defensin, alpha, 34	-4.84
ENSMUSG00000029005	Draxin	dorsal inhibitory axon guidance protein	-4.84
ENSMUSG00000079056	Kcnip3	Kv channel interacting protein 3, calsenilin	-4.85
ENSMUSG00000050824	Sstr5	somatostatin receptor 5	-4.85
ENSMUSG00000031144	Syp	synaptophysin	-4.86
ENSMUSG00000058618	AY761184	cDNA sequence AY761184	-4.86
ENSMUSG00000020701	Tmem132e	transmembrane protein 132E	-4.88
ENSMUSG00000025964	Adam23	a disintegrin and metallopeptidase domain 23	-4.88
ENSMUSG00000025020	Slit1	slit guidance ligand 1	-4.89
ENSMUSG00000025089	Gfra1	glial cell line derived neurotrophic factor family receptor alpha 1	-4.89
ENSMUSG00000037428	Vgf	VGF nerve growth factor inducible	-4.90
ENSMUSG00000021763	BC067074	cDNA sequence BC067074	-4.90
ENSMUSG00000096295	Defa2	defensin, alpha, 2	-4.91
ENSMUSG00000064080	Fbln2	fibulin 2	-4.91
ENSMUSG00000031284	Pak3	p21 (RAC1) activated kinase 3	-4.91
ENSMUSG00000004110	Cacna1e	calcium channel, voltage-dependent, R type, alpha 1E subunit	-4.91
ENSMUSG00000051331	Cacna1c	calcium channel, voltage-dependent, L type, alpha 1C subunit	-4.92
ENSMUSG00000020848	Doc2b	double C2, beta	-4.92
ENSMUSG00000032769	Trpa1	transient receptor potential cation channel, subfamily A, member 1	-4.92
ENSMUSG00000026833	Olfm1	olfactomedin 1	-4.93
ENSMUSG00000043301	Kcnj6	potassium inwardly-rectifying channel, subfamily J, member 6	-4.93
ENSMUSG00000019796	Lrp11	low density lipoprotein receptor-related protein 11	-4.95
ENSMUSG00000038224	Serpinf2	serine (or cysteine) peptidase inhibitor, clade F, member 2	-4.95
ENSMUSG00000051079	Rgs13	regulator of G-protein signaling 13	-4.97
ENSMUSG00000044365	Cxxc4	CXXC finger 4	-4.98
ENSMUSG00000041633	Kctd12b	potassium channel tetramerisation domain containing 12b	-4.99
ENSMUSG00000099907	Gm10421	predicted gene 10421	-5.00
ENSMUSG00000037852	Cpe	carboxypeptidase E	-5.02
ENSMUSG00000083282	Ctsf	cathepsin F	-5.02
ENSMUSG00000033061	Resp18	regulated endocrine-specific protein 18	-5.02
ENSMUSG00000035967	Ints6l	integrator complex subunit 6 like	-5.02
ENSMUSG00000058248	Kcnh1	potassium voltage-gated channel, subfamily H (eag-related), member 1	-5.02
ENSMUSG00000020734	Grin2c	glutamate receptor, ionotropic, NMDA2C (epsilon 3)	-5.02
ENSMUSG00000075012	Fjx1	four jointed box 1	-5.03
ENSMUSG00000020090	Npffr1	neuropeptide FF receptor 1	-5.04
ENSMUSG00000068154	Insm1	insulinoma-associated 1	-5.05
ENSMUSG00000020723	Cacng4	calcium channel, voltage-dependent, gamma subunit 4	-5.06
ENSMUSG00000047507	Baiap3	BAI1-associated protein 3	-5.07

ENSMUSG00000028008	Asic5	acid-sensing (proton-gated) ion channel family member 5	-5.09
ENSMUSG00000020333	Acsl6	acyl-CoA synthetase long-chain family member 6	-5.10
ENSMUSG00000091345	Col6a5	collagen, type VI, alpha 5	-5.10
ENSMUSG00000105271	Gm42875	predicted gene 42875	-5.10
ENSMUSG00000049605	Olfr418	olfactory receptor 418	-5.11
ENSMUSG00000028339	Col15a1	collagen, type XV, alpha 1	-5.13
ENSMUSG00000106320	Gm42992	predicted gene 42992	-5.13
ENSMUSG00000008932	Slc1a7	solute carrier family 1 (glutamate transporter), member 7	-5.13
ENSMUSG00000016346	Kcnq2	potassium voltage-gated channel, subfamily Q, member 2	-5.13
ENSMUSG00000006546	Cryba2	crystallin, beta A2	-5.14
ENSMUSG00000062380	Tubb3	tubulin, beta 3 class III	-5.15
ENSMUSG00000020811	Wscd1	WSC domain containing 1	-5.15
ENSMUSG00000051980	Casr	calcium-sensing receptor	-5.15
ENSMUSG00000020886	Dlg4	discs large MAGUK scaffold protein 4	-5.16
ENSMUSG00000057182	Scn3a	sodium channel, voltage-gated, type III, alpha	-5.17
ENSMUSG00000090291	Lrrc10b	leucine rich repeat containing 10B	-5.17
ENSMUSG00000017311	Pyy	peptide YY	-5.17
ENSMUSG00000015401	Cltrn	collectrin, amino acid transport regulator	-5.18
ENSMUSG00000039037	St6galnac5	ST6 (alpha-N-acetyl-neuraminyl-2,3-beta-galactosyl-1,3)-N-acetylgalactosaminide a	-5.19
ENSMUSG00000036766	Dner	delta/notch-like EGF repeat containing	-5.19
ENSMUSG00000017943	Gdap1l1	ganglioside-induced differentiation-associated protein 1-like 1	-5.19
ENSMUSG00000047798	Cd300lf	CD300 molecule like family member F	-5.19
ENSMUSG00000005640	Insrr	insulin receptor-related receptor	-5.19
ENSMUSG00000105985	Gm42993	predicted gene 42993	-5.20
ENSMUSG00000104886	Gm43000	predicted gene 43000	-5.20
ENSMUSG00000055567	Unc80	unc-80, NALCN activator	-5.21
ENSMUSG00000070577	Gm572	predicted gene 572	-5.21
ENSMUSG00000040276	Pacsin1	protein kinase C and casein kinase substrate in neurons 1	-5.22
ENSMUSG00000020703	5530401A14Rik	RIKEN cDNA 5530401A14 gene	-5.22
ENSMUSG00000027834	Serpini1	serine (or cysteine) peptidase inhibitor, clade I, member 1	-5.23
ENSMUSG00000027876	Reg4	regenerating islet-derived family, member 4	-5.23
ENSMUSG00000095328	Defa-ps6	defensin, alpha, pseudogene 6	-5.23
ENSMUSG00000114624	Gm48267	predicted gene, 48267	-5.23
ENSMUSG00000037624	Kcnk2	potassium channel, subfamily K, member 2	-5.23
ENSMUSG00000079180	Mptx2	mucosal pentraxin 2	-5.25
ENSMUSG00000044359	P2ry4	pyrimidinergic receptor P2Y, G-protein coupled, 4	-5.25
ENSMUSG00000050751	Pgbd5	piggyBac transposable element derived 5	-5.25
ENSMUSG00000048148	Nwd1	NACHT and WD repeat domain containing 1	-5.26
ENSMUSG00000058975	Kcnc1	potassium voltage gated channel, Shaw-related subfamily, member 1	-5.27
ENSMUSG00000040118	Cacna2d1	calcium channel, voltage-dependent, alpha2/delta subunit 1	-5.28
ENSMUSG00000032338	Hcn4	hyperpolarization-activated, cyclic nucleotide-gated K+ 4	-5.28
ENSMUSG00000106048	Gm42444	predicted gene 42444	-5.28
ENSMUSG00000010505	Myt1	myelin transcription factor 1	-5.29
ENSMUSG00000028469	Npr2	natriuretic peptide receptor 2	-5.29
ENSMUSG00000025810	Nrp1	neuropilin 1	-5.30
ENSMUSG00000115190	Gm49542	predicted gene, 49542	-5.30
ENSMUSG00000023224	Serping1	serine (or cysteine) peptidase inhibitor, clade G, member 1	-5.32
ENSMUSG00000052727	Map1b	microtubule-associated protein 1B	-5.32
ENSMUSG00000031740	Mmp2	matrix metallopeptidase 2	-5.32
ENSMUSG00000035431	Sstr1	somatostatin receptor 1	-5.35
ENSMUSG00000114245	Gm38655	predicted gene, 38655	-5.35
ENSMUSG00000043004	Gng2	guanine nucleotide binding protein (G protein), gamma 2	-5.36
ENSMUSG00000040009	Gnaz	guanine nucleotide binding protein, alpha z subunit	-5.36
ENSMUSG00000006586	Runx1t1	runt-related transcription factor 1; translocated to, 1 (cyclin D-related)	-5.36
ENSMUSG00000031837	Necab2	N-terminal EF-hand calcium binding protein 2	-5.36
ENSMUSG00000028360	Slc44a5	solute carrier family 44, member 5	-5.37
ENSMUSG00000038456	Dennd2a	DENN/MADD domain containing 2A	-5.38
ENSMUSG00000024682	Gif	gastric intrinsic factor	-5.39
ENSMUSG00000075256	Cerkl	ceramide kinase-like	-5.40
ENSMUSG00000032394	Igdcc3	immunoglobulin superfamily, DCC subclass, member 3	-5.40
ENSMUSG00000046613	Vwa5b2	von Willebrand factor A domain containing 5B2	-5.41
ENSMUSG00000026872	Zeb2	zinc finger E-box binding homeobox 2	-5.41
ENSMUSG00000021303	Gng4	guanine nucleotide binding protein (G protein), gamma 4	-5.43
ENSMUSG00000031099	Smarca1	SWI/SNF related, matrix associated, actin dependent regulator of chromatin, subfa	-5.43
ENSMUSG00000096847	Tmem151b	transmembrane protein 151B	-5.44
ENSMUSG00000046743	Fat4	FAT atypical cadherin 4	-5.47
ENSMUSG00000048038	Ccdc187	coiled-coil domain containing 187	-5.47

ENSMUSG00000097767	Miat	myocardial infarction associated transcript (non-protein coding)	-5.48
ENSMUSG00000105526	Gm43490	predicted gene 43490	-5.48
ENSMUSG00000054667	Irs4	insulin receptor substrate 4	-5.48
ENSMUSG00000028356	Ambp	alpha 1 microglobulin/bikunin	-5.49
ENSMUSG00000050473	Slc35d3	solute carrier family 35, member D3	-5.49
ENSMUSG00000058740	Kcnt1	potassium channel, subfamily T, member 1	-5.50
ENSMUSG00000051985	Igfn1	immunoglobulin-like and fibronectin type III domain containing 1	-5.50
ENSMUSG00000104301	Wdr49	WD repeat domain 49	-5.51
ENSMUSG00000092083	Kcnb2	potassium voltage gated channel, Shab-related subfamily, member 2	-5.52
ENSMUSG00000029706	Pax4	paired box 4	-5.53
ENSMUSG00000026443	Lrrn2	leucine rich repeat protein 2, neuronal	-5.53
ENSMUSG00000027009	Itga4	integrin alpha 4	-5.54
ENSMUSG00000042750	Bex2	brain expressed X-linked 2	-5.55
ENSMUSG00000100182	1810006J02Rik	RIKEN cDNA 1810006J02 gene	-5.58
ENSMUSG00000026442	Nfasc	neurofascin	-5.59
ENSMUSG00000038530	Rgs4	regulator of G-protein signaling 4	-5.59
ENSMUSG00000040046	Tph1	tryptophan hydroxylase 1	-5.59
ENSMUSG00000034145	Tmem63c	transmembrane protein 63c	-5.61
ENSMUSG00000001901	Kcnh6	potassium voltage-gated channel, subfamily H (eag-related), member 6	-5.62
ENSMUSG00000039323	Igfbp2	insulin-like growth factor binding protein 2	-5.62
ENSMUSG00000043857	Mgat5b	mannoside acetylglucosaminyltransferase 5, isoenzyme B	-5.62
ENSMUSG00000104149	Gm37138	predicted gene, 37138	-5.63
ENSMUSG00000027360	Hdc	histidine decarboxylase	-5.67
ENSMUSG00000026163	Sphkap	SPHK1 interactor, AKAP domain containing	-5.69
ENSMUSG00000090071	Cdk5r2	cyclin-dependent kinase 5, regulatory subunit 2 (p39)	-5.70
ENSMUSG00000035277	Arx	aristaless related homeobox	-5.73
ENSMUSG00000039059	Hrh3	histamine receptor H3	-5.73
ENSMUSG00000041798	Gck	glucokinase	-5.74
ENSMUSG00000026432	Avpr1b	arginine vasopressin receptor 1B	-5.74
ENSMUSG00000024366	Gfra3	glial cell line derived neurotrophic factor family receptor alpha 3	-5.75
ENSMUSG00000033676	Gabrb3	gamma-aminobutyric acid (GABA) A receptor, subunit beta 3	-5.75
ENSMUSG00000086067	Gm16183	predicted gene 16183	-5.76
ENSMUSG00000034701	Neurod1	neurogenic differentiation 1	-5.77
ENSMUSG00000070802	Pnmal2	PNMA-like 2	-5.77
ENSMUSG00000095813	Defa-ps1	defensin, alpha, pseudogene 1	-5.78
ENSMUSG00000021536	Adcy2	adenylate cyclase 2	-5.78
ENSMUSG00000061171	Slc38a11	solute carrier family 38, member 11	-5.79
ENSMUSG00000094626	Tmem121b	transmembrane protein 121B	-5.79
ENSMUSG00000061762	Tac1	tachykinin 1	-5.80
ENSMUSG00000024990	Rbp4	retinol binding protein 4, plasma	-5.81
ENSMUSG00000021194	Chga	chromogranin A	-5.82
ENSMUSG00000032181	Scg3	secretogranin III	-5.82
ENSMUSG00000053166	Cdh22	cadherin 22	-5.82
ENSMUSG00000028546	Elavl4	ELAV like RNA binding protein 4	-5.83
ENSMUSG00000047842	Diras2	DIRAS family, GTP-binding RAS-like 2	-5.83
ENSMUSG00000101814	Gm17807	predicted gene, 17807	-5.83
ENSMUSG00000024112	Cacna1h	calcium channel, voltage-dependent, T type, alpha 1H subunit	-5.87
ENSMUSG00000056553	Ptprn2	protein tyrosine phosphatase, receptor type, N polypeptide 2	-5.88
ENSMUSG00000054640	Slc8a1	solute carrier family 8 (sodium/calcium exchanger), member 1	-5.88
ENSMUSG00000020177	9530003J23Rik	RIKEN cDNA 9530003J23 gene	-5.88
ENSMUSG00000027350	Chgb	chromogranin B	-5.92
ENSMUSG00000010021	Kif19a	kinesin family member 19A	-5.93
ENSMUSG00000033740	St18	suppression of tumorigenicity 18	-5.94
ENSMUSG00000026204	Ptprn	protein tyrosine phosphatase, receptor type, N	-5.96
ENSMUSG00000066456	Hmgn3	high mobility group nucleosomal binding domain 3	-5.96
ENSMUSG00000042631	Xkr7	X-linked Kx blood group related 7	-5.96
ENSMUSG00000039860	Srrm3	serine/arginine repetitive matrix 3	-5.97
ENSMUSG00000043496	Tril	TLR4 interactor with leucine-rich repeats	-5.97
ENSMUSG00000110557	Gm5159	predicted gene 5159	-5.97
ENSMUSG00000025375	Aatk	apoptosis-associated tyrosine kinase	-5.99
ENSMUSG00000021700	Rab3c	RAB3C, member RAS oncogene family	-6.02
ENSMUSG00000029420	Rimbp2	RIMS binding protein 2	-6.03
ENSMUSG00000072849	Serpina1e	serine (or cysteine) peptidase inhibitor, clade A, member 1E	-6.03
ENSMUSG00000033981	Gria2	glutamate receptor, ionotropic, AMPA2 (alpha 2)	-6.04
ENSMUSG00000021904	Sema3g	sema domain, immunoglobulin domain (Ig), short basic domain, secreted, (semaph	-6.07
ENSMUSG00000004366	Sst	somatostatin	-6.08
ENSMUSG00000071265	1700086L19Rik	RIKEN cDNA 1700086L19 gene	-6.10

ENSMUSG00000019900	Rfx6	regulatory factor X, 6	-6.12
ENSMUSG00000027168	Pax6	paired box 6	-6.12
ENSMUSG00000039683	Sdk1	sidekick cell adhesion molecule 1	-6.13
ENSMUSG00000101930	Gm5441	predicted gene 5441	-6.13
ENSMUSG00000053545	6430503K07Rik	RIKEN cDNA 6430503K07 gene	-6.16
ENSMUSG00000079116	Gm15293	predicted gene 15293	-6.18
ENSMUSG00000019876	Pkib	protein kinase inhibitor beta, cAMP dependent, testis specific	-6.18
ENSMUSG00000044453	Ffar1	free fatty acid receptor 1	-6.21
ENSMUSG00000039278	Pcsk1n	proprotein convertase subtilisin/kexin type 1 inhibitor	-6.23
ENSMUSG00000040972	Igsf21	immunoglobulin superfamily, member 21	-6.23
ENSMUSG00000026824	Kcnj3	potassium inwardly-rectifying channel, subfamily J, member 3	-6.24
ENSMUSG00000027220	Syt13	synaptotagmin XIII	-6.25
ENSMUSG00000023236	Scg5	secretogranin V	-6.28
ENSMUSG00000061601	Pclo	piccolo (presynaptic cytomatrix protein)	-6.30
ENSMUSG00000050556	Kcnb1	potassium voltage gated channel, Shab-related subfamily, member 1	-6.30
ENSMUSG00000021198	Unc79	unc-79 homolog	-6.30
ENSMUSG00000048483	Zdhhc22	zinc finger, DHHC-type containing 22	-6.30
ENSMUSG00000038255	Neurod2	neurogenic differentiation 2	-6.37
ENSMUSG00000068263	Efcc1	EF hand and coiled-coil domain containing 1	-6.38
ENSMUSG00000048215	A630023P12Rik	RIKEN cDNA A630023P12 gene	-6.38
ENSMUSG00000026686	Lmx1a	LIM homeobox transcription factor 1 alpha	-6.40
ENSMUSG00000023387	Kcnk16	potassium channel, subfamily K, member 16	-6.43
ENSMUSG00000048218	Amigo2	adhesion molecule with Ig like domain 2	-6.43
ENSMUSG00000054423	Cadps	Ca2+-dependent secretion activator	-6.45
ENSMUSG00000050711	Scg2	secretogranin II	-6.48
ENSMUSG00000037032	Apbb1	amyloid beta (A4) precursor protein-binding, family B, member 1	-6.48
ENSMUSG00000114230	Gm48239	predicted gene, 48239	-6.48
ENSMUSG00000052951	C130021I20Rik	Riken cDNA C130021I20 gene	-6.49
ENSMUSG00000106139	Gm30648	predicted gene, 30648	-6.51
ENSMUSG00000042258	Isl1	ISL1 transcription factor, LIM/homeodomain	-6.57
ENSMUSG00000014351	Gip	gastric inhibitory polypeptide	-6.58
ENSMUSG00000032532	Cck	cholecystokinin	-6.58
ENSMUSG00000029878	Dbpht2	DNA binding protein with his-thr domain	-6.59
ENSMUSG00000071719	Tmem28	transmembrane protein 28	-6.59
ENSMUSG00000025738	Fbxl16	F-box and leucine-rich repeat protein 16	-6.70
ENSMUSG00000102937	Gm38116	predicted gene, 38116	-6.72
ENSMUSG00000021337	Scgn	secretagogin, EF-hand calcium binding protein	-6.74
ENSMUSG00000069072	Slc7a14	solute carrier family 7 (cationic amino acid transporter, y+ system), member 14	-6.80
ENSMUSG00000073530	Pappa2	pappalysin 2	-6.88
ENSMUSG00000029121	Crmp1	collapsin response mediator protein 1	-6.94
ENSMUSG00000024553	Galr1	galanin receptor 1	-6.94
ENSMUSG00000055409	Nell1	NEL-like 1	-7.00
ENSMUSG00000028226	Mmp16	matrix metallopeptidase 16	-7.10
ENSMUSG00000055197	Fev	FEV (ETS oncogene family)	-7.25
ENSMUSG00000009292	Trpm2	transient receptor potential cation channel, subfamily M, member 2	-7.40
ENSMUSG00000022762	Ncam2	neural cell adhesion molecule 2	-7.43
ENSMUSG00000039372	38047	membrane-associated ring finger (C3HC4) 4	-7.51
ENSMUSG00000041141	Pnmal1	PNMA-like 1	-7.61
ENSMUSG00000018698	Lhx1	LIM homeobox protein 1	-7.68
ENSMUSG00000030359	Pzp	PZP, alpha-2-macroglobulin like	-7.76
ENSMUSG00000053852	Adgrg4	adhesion G protein-coupled receptor G4	-7.97
ENSMUSG00000038765	Lmx1b	LIM homeobox transcription factor 1 beta	-8.47
ENSMUSG00000022619	Mapk8ip2	mitogen-activated protein kinase 8 interacting protein 2	-9.32

RFP+Trop2- vs. differentiated cells

Ensemble gene code	Symbol	Gene name	logFC
ENSMUSG00000097519	4930558J18Rik	RIKEN cDNA 4930558J18 gene	2.88
ENSMUSG00000082095	Gm11991	predicted gene 11991	2.55
ENSMUSG00000082706	Gm11663	predicted gene 11663	2.35
ENSMUSG00000037124	Trim58	tripartite motif-containing 58	2.33
ENSMUSG00000033952	Aspm	abnormal spindle microtubule assembly	2.26
ENSMUSG00000032783	Troap	trophinin associated protein	2.26
ENSMUSG00000041498	Kif14	kinesin family member 14	2.25
ENSMUSG00000044201	Cdc25c	cell division cycle 25C	2.21
ENSMUSG00000061991	Hist1h2af	histone cluster 1, H2af	2.20
ENSMUSG00000037628	Cdkn3	cyclin-dependent kinase inhibitor 3	2.19
ENSMUSG00000108528	Gm8319	predicted gene 8319	2.19
ENSMUSG00000023505	Cdca3	cell division cycle associated 3	2.17
ENSMUSG00000028678	Kif2c	kinesin family member 2C	2.17
ENSMUSG00000027715	Ccna2	cyclin A2	2.16
ENSMUSG00000102359	Gm38351	predicted gene, 38351	2.16
ENSMUSG00000028068	Iqgap3	IQ motif containing GTPase activating protein 3	2.15
ENSMUSG00000035365	Parpbp	PARP1 binding protein	2.13
ENSMUSG00000017716	Birc5	baculoviral IAP repeat-containing 5	2.11
ENSMUSG00000026605	Cenpf	centromere protein F	2.11
ENSMUSG00000026622	Nek2	NIMA (never in mitosis gene a)-related expressed kinase 2	2.11
ENSMUSG00000026683	Nuf2	NUF2, NDC80 kinetochore complex component	2.11
ENSMUSG00000041431	Ccnb1	cyclin B1	2.11
ENSMUSG00000006398	Cdc20	cell division cycle 20	2.10
ENSMUSG00000039814	Xkr5	X-linked Kx blood group related 5	2.10
ENSMUSG00000003779	Kif20a	kinesin family member 20A	2.08
ENSMUSG00000051378	Kif18b	kinesin family member 18B	2.08
ENSMUSG00000020330	Hmmr	hyaluronan mediated motility receptor (RHAMM)	2.07
ENSMUSG00000045328	Cenpe	centromere protein E	2.07
ENSMUSG00000041064	Pif1	PIF1 5'-to-3' DNA helicase	2.07
ENSMUSG00000032218	Ccnb2	cyclin B2	2.06
ENSMUSG00000058290	Espl1	extra spindle pole bodies 1, separase	2.06
ENSMUSG00000079553	Kifc1	kinesin family member C1	2.06
ENSMUSG00000027469	Tpx2	TPX2, microtubule-associated	2.05
ENSMUSG00000027379	Bub1	BUB1, mitotic checkpoint serine/threonine kinase	2.04
ENSMUSG00000114456	Hist1h2bh	histone cluster 1, H2bh	2.04
ENSMUSG00000029177	Cenpa	centromere protein A	2.02
ENSMUSG00000001403	Ube2c	ubiquitin-conjugating enzyme E2C	2.01
ENSMUSG00000031262	Cenpi	centromere protein I	2.01
ENSMUSG00000094248	Hist1h2ao	histone cluster 1, H2ao	2.01
ENSMUSG00000113168	Gm7614	predicted gene 7614	2.00
ENSMUSG00000027115	Kif18a	kinesin family member 18A	1.99
ENSMUSG00000098985	Gm27219	predicted gene 27219	1.99
ENSMUSG00000028175	Depdc1a	DEP domain containing 1a	1.98
ENSMUSG00000027699	Ect2	ect2 oncogene	1.97
ENSMUSG00000027331	Knstrn	kinetochore-localized astrin/SPAG5 binding	1.97
ENSMUSG00000037725	Ckap2	cytoskeleton associated protein 2	1.97
ENSMUSG00000024795	Kif20b	kinesin family member 20B	1.97
ENSMUSG00000012443	Kif11	kinesin family member 11	1.95
ENSMUSG00000022322	Shcbp1	Shc SH2-domain binding protein 1	1.94
ENSMUSG00000072082	Ccnf	cyclin F	1.94
ENSMUSG00000027326	Knl1	kinetochore scaffold 1	1.92
ENSMUSG00000019942	Cdk1	cyclin-dependent kinase 1	1.92
ENSMUSG00000023015	Racgap1	Rac GTPase-activating protein 1	1.92
ENSMUSG00000088956	Gm24945	predicted gene, 24945	1.92
ENSMUSG00000027496	Aurka	aurora kinase A	1.91
ENSMUSG00000021176	Efcab11	EF-hand calcium binding domain 11	1.91
ENSMUSG00000034311	Kif4	kinesin family member 4	1.90
ENSMUSG00000027811	4930579G24Rik	RIKEN cDNA 4930579G24 gene	1.90
ENSMUSG00000020914	Top2a	topoisomerase (DNA) II alpha	1.89
ENSMUSG00000069910	Spdl1	spindle apparatus coiled-coil protein 1	1.89
ENSMUSG00000099583	Hist1h3d	histone cluster 1, H3d	1.89
ENSMUSG00000021965	Ska3	spindle and kinetochore associated complex subunit 3	1.88
ENSMUSG00000023940	Sgo1	shugoshin 1	1.88
ENSMUSG00000030677	Kif22	kinesin family member 22	1.87
ENSMUSG00000048327	Ckap2l	cytoskeleton associated protein 2-like	1.87
ENSMUSG00000069300	Hist1h2bj	histone cluster 1, H2bj	1.87
ENSMUSG00000024056	Ndc80	NDC80 kinetochore complex component	1.87
ENSMUSG00000032254	Kif23	kinesin family member 23	1.86
ENSMUSG00000037544	Dlgap5	DLG associated protein 5	1.86
ENSMUSG00000035683	Melk	maternal embryonic leucine zipper kinase	1.86

ENSMUSG00000099517	Hist1h3g	histone cluster 1, H3g	1.86
ENSMUSG00000033031	Cip2a	cell proliferation regulating inhibitor of protein phosphatase 2A	1.85
ENSMUSG00000114279	Hist1h2bm	histone cluster 1, H2bm	1.85
ENSMUSG00000032221	Mns1	meiosis-specific nuclear structural protein 1	1.85
ENSMUSG00000055653	Gpc3	glypican 3	1.85
ENSMUSG00000028873	Cdca8	cell division cycle associated 8	1.84
ENSMUSG00000069267	Hist1h3b	histone cluster 1, H3b	1.84
ENSMUSG00000040084	Bub1b	BUB1B, mitotic checkpoint serine/threonine kinase	1.84
ENSMUSG00000026955	Sapcd2	suppressor APC domain containing 2	1.83
ENSMUSG00000024989	Cep55	centrosomal protein 55	1.82
ENSMUSG00000037313	Tacc3	transforming, acidic coiled-coil containing protein 3	1.82
ENSMUSG00000038252	Ncapd2	non-SMC condensin I complex, subunit D2	1.82
ENSMUSG00000101355	Hist1h3h	histone cluster 1, H3h	1.82
ENSMUSG00000031004	Mki67	antigen identified by monoclonal antibody Ki 67	1.81
ENSMUSG00000022033	Pbk	PDZ binding kinase	1.81
ENSMUSG00000036768	Kif15	kinesin family member 15	1.81
ENSMUSG00000020897	Aurkb	aurora kinase B	1.80
ENSMUSG00000020493	Prr11	proline rich 11	1.79
ENSMUSG00000024087	Cyp1b1	cytochrome P450, family 1, subfamily b, polypeptide 1	1.79
ENSMUSG00000058773	Hist1h1b	histone cluster 1, H1b	1.78
ENSMUSG00000015880	Ncapg	non-SMC condensin I complex, subunit G	1.78
ENSMUSG00000075031	Hist1h2bb	histone cluster 1, H2bb	1.78
ENSMUSG00000069274	Hist1h4f	histone cluster 1, H4f	1.77
ENSMUSG00000069310	Hist1h3c	histone cluster 1, H3c	1.77
ENSMUSG00000062727	Hist1h2bk	histone cluster 1, H2bk	1.76
ENSMUSG00000038379	Ttk	Ttk protein kinase	1.76
ENSMUSG00000046295	Ankle1	ankyrin repeat and LEM domain containing 1	1.76
ENSMUSG00000044966	Fbxo48	F-box protein 48	1.76
ENSMUSG00000082605	Gm16148	predicted gene 16148	1.76
ENSMUSG00000048922	Cdca2	cell division cycle associated 2	1.75
ENSMUSG00000027306	Nusap1	nucleolar and spindle associated protein 1	1.74
ENSMUSG00000034906	Ncaph	non-SMC condensin I complex, subunit H	1.74
ENSMUSG00000095217	Hist1h2bn	histone cluster 1, H2bn	1.74
ENSMUSG00000028312	Smc2	structural maintenance of chromosomes 2	1.74
ENSMUSG00000031756	Cenpn	centromere protein N	1.74
ENSMUSG00000038943	Prc1	protein regulator of cytokinesis 1	1.73
ENSMUSG00000024301	Kifc5b	kinesin family member C5B	1.73
ENSMUSG00000085627	Gm11222	predicted gene 11222	1.73
ENSMUSG00000051517	Arhgef39	Rho guanine nucleotide exchange factor (GEF) 39	1.72
ENSMUSG00000017969	Ptgis	prostaglandin I2 (prostacyclin) synthase	1.71
ENSMUSG00000001517	Foxm1	forkhead box M1	1.70
ENSMUSG00000022385	Gtse1	G two S phase expressed protein 1	1.70
ENSMUSG00000064288	Hist1h4k	histone cluster 1, H4k	1.70
ENSMUSG00000021569	Trip13	thyroid hormone receptor interactor 13	1.70
ENSMUSG00000027323	Rad51	RAD51 recombinase	1.70
ENSMUSG00000051220	Ercc6l	excision repair cross-complementing rodent repair deficiency complementation group 6 like	1.70
ENSMUSG00000034883	Lrr1	leucine rich repeat protein 1	1.70
ENSMUSG00000094338	Hist1h2bl	histone cluster 1, H2bl	1.70
ENSMUSG00000047534	Mis18bp1	MIS18 binding protein 1	1.68
ENSMUSG00000049916	2610318N02Rik	RIKEN cDNA 2610318N02 gene	1.68
ENSMUSG00000022021	Diaph3	diaphanous related formin 3	1.67
ENSMUSG00000036223	Ska1	spindle and kinetochore associated complex subunit 1	1.67
ENSMUSG00000067818	Myl9	myosin, light polypeptide 9, regulatory	1.67
ENSMUSG00000022034	Esco2	establishment of sister chromatid cohesion N-acetyltransferase 2	1.66
ENSMUSG00000002055	Spag5	sperm associated antigen 5	1.66
ENSMUSG00000078773	Rad54b	RAD54 homolog B (S. cerevisiae)	1.66
ENSMUSG00000024660	Incenp	inner centromere protein	1.65
ENSMUSG00000040204	Pclaf	PCNA clamp associated factor	1.65
ENSMUSG00000036777	Anln	anillin, actin binding protein	1.65
ENSMUSG00000058385	Hist1h2bg	histone cluster 1, H2bg	1.64
ENSMUSG00000029910	Mad2l1	MAD2 mitotic arrest deficient-like 1	1.64
ENSMUSG00000005233	Spc25	SPC25, NDC80 kinetochore complex component, homolog (S. cerevisiae)	1.63
ENSMUSG00000026039	Sgo2a	shugoshin 2A	1.63
ENSMUSG00000051235	Gen1	GEN1, Holliday junction 5' flap endonuclease	1.63
ENSMUSG00000025154	Arhgap19	Rho GTPase activating protein 19	1.62
ENSMUSG00000034773	BC030867	cDNA sequence BC030867	1.62
ENSMUSG00000074476	Spc24	SPC24, NDC80 kinetochore complex component, homolog (S. cerevisiae)	1.61
ENSMUSG00000021697	Depdc1b	DEP domain containing 1B	1.61
ENSMUSG00000017861	Mybl2	myeloblastosis oncogene-like 2	1.60
ENSMUSG00000029516	Cit	citron	1.60
ENSMUSG00000023004	Tuba1b	tubulin, alpha 1B	1.60
ENSMUSG00000081670	Gm15697	predicted gene 15697	1.60
ENSMUSG00000046591	Ticrr	TOPBP1-interacting checkpoint and replication regulator	1.59

ENSMUSG00000062248	Cks2	CDC28 protein kinase regulatory subunit 2	1.58
ENSMUSG00000029521	Chek2	checkpoint kinase 2	1.58
ENSMUSG00000031257	Nox1	NADPH oxidase 1	1.58
ENSMUSG00000034349	Smc4	structural maintenance of chromosomes 4	1.57
ENSMUSG00000025902	Sox17	SRY (sex determining region Y)-box 17	1.57
ENSMUSG00000011267	Zfp296	zinc finger protein 296	1.57
ENSMUSG00000020808	Pimreg	PICALM interacting mitotic regulator	1.57
ENSMUSG00000000028	Cdc45	cell division cycle 45	1.56
ENSMUSG00000005470	Asf1b	anti-silencing function 1B histone chaperone	1.56
ENSMUSG00000035455	Fignl1	fidgetin-like 1	1.56
ENSMUSG00000026669	Mcm10	minichromosome maintenance 10 replication initiation factor	1.54
ENSMUSG00000063021	Hist1h2ak	histone cluster 1, H2ak	1.54
ENSMUSG00000078521	Aunip	aurora kinase A and ninein interacting protein	1.54
ENSMUSG00000079357	Gm11100	predicted gene 11100	1.54
ENSMUSG00000054717	Hmgb2	high mobility group box 2	1.53
ENSMUSG00000025758	Plk4	polo like kinase 4	1.53
ENSMUSG00000042489	Clspn	claspin	1.53
ENSMUSG00000060093	Hist1h4a	histone cluster 1, H4a	1.53
ENSMUSG00000068744	Psrc1	proline/serine-rich coiled-coil 1	1.53
ENSMUSG00000052565	Hist1h1d	histone cluster 1, H1d	1.52
ENSMUSG00000002297	Dbf4	DBF4 zinc finger	1.52
ENSMUSG00000024791	Cdca5	cell division cycle associated 5	1.52
ENSMUSG00000045273	Cenph	centromere protein H	1.52
ENSMUSG00000102840	Gm38037	predicted gene, 38037	1.52
ENSMUSG00000024590	Lmnb1	lamin B1	1.51
ENSMUSG00000005410	Mcm5	minichromosome maintenance complex component 5	1.51
ENSMUSG00000020185	E2f7	E2F transcription factor 7	1.51
ENSMUSG00000039994	Timeless	timeless circadian clock 1	1.51
ENSMUSG00000049539	Hist1h1a	histone cluster 1, H1a	1.50
ENSMUSG00000027203	Dut	deoxyuridine triphosphatase	1.50
ENSMUSG00000034023	Fancd2	Fanconi anemia, complementation group D2	1.50
ENSMUSG00000072980	Oip5	Opa interacting protein 5	1.50
ENSMUSG00000028933	Xrcc2	X-ray repair complementing defective repair in Chinese hamster cells 2	1.50
ENSMUSG00000069307	Hist1h2bq	histone cluster 1, H2bq	1.50
ENSMUSG00000049932	H2afx	H2A histone family, member X	1.49
ENSMUSG00000030978	Rrm1	ribonucleotide reductase M1	1.49
ENSMUSG00000046179	E2f8	E2F transcription factor 8	1.49
ENSMUSG00000062937	Mtap	methylthioadenosine phosphorylase	1.49
ENSMUSG00000039509	Nup133	nucleoporin 133	1.49
ENSMUSG00000032400	Zwilch	zwilch kinetochore protein	1.49
ENSMUSG00000061615	Hist1h2ab	histone cluster 1, H2ab	1.49
ENSMUSG00000039187	Fanci	Fanconi anemia, complementation group I	1.49
ENSMUSG00000069266	Hist1h4b	histone cluster 1, H4b	1.48
ENSMUSG00000040658	Dnph1	2'-deoxynucleoside 5'-phosphate N-hydrolase 1	1.48
ENSMUSG00000110644	Gm7390	predicted gene 7390	1.48
ENSMUSG00000062510	Nsl1	NSL1, MIS12 kinetochore complex component	1.47
ENSMUSG00000027635	Dsn1	DSN1 homolog, MIS12 kinetochore complex component	1.47
ENSMUSG00000069268	Hist1h2bf	histone cluster 1, H2bf	1.47
ENSMUSG00000059791	Nrm	nurim (nuclear envelope membrane protein)	1.47
ENSMUSG00000054342	Kcnn4	potassium intermediate/small conductance calcium-activated channel, subfamily N, member 4	1.46
ENSMUSG00000028702	Rad54l	RAD54 like (S. cerevisiae)	1.46
ENSMUSG00000035351	Nup37	nucleoporin 37	1.46
ENSMUSG00000073705	Cenps	centromere protein S	1.46
ENSMUSG00000032586	Traip	TRAF-interacting protein	1.46
ENSMUSG00000110631	Gm42047	predicted gene, 42047	1.46
ENSMUSG00000080186	Gm14448	predicted gene 14448	1.46
ENSMUSG00000001228	Uhrf1	ubiquitin-like, containing PHD and RING finger domains, 1	1.45
ENSMUSG00000025747	Tyms	thymidylate synthase	1.45
ENSMUSG00000039396	Neil3	nei like 3 (E. coli)	1.45
ENSMUSG00000069184	Zfp72	zinc finger protein 72	1.45
ENSMUSG00000028614	Ndc1	NDC1 transmembrane nucleoporin	1.44
ENSMUSG00000030346	Rad51ap1	RAD51 associated protein 1	1.44
ENSMUSG00000069272	Hist1h2ae	histone cluster 1, H2ae	1.44
ENSMUSG00000031112	Stk26	serine/threonine kinase 26	1.44
ENSMUSG00000007050	Lsm2	LSM2 homolog, U6 small nuclear RNA and mRNA degradation associated	1.43
ENSMUSG00000019773	Fbxo5	F-box protein 5	1.43
ENSMUSG00000041859	Mcm3	minichromosome maintenance complex component 3	1.43
ENSMUSG00000024613	Tcof1	treacle ribosome biogenesis factor 1	1.43
ENSMUSG00000069265	Hist1h3a	histone cluster 1, H3a	1.43
ENSMUSG00000024891	Slc29a2	solute carrier family 29 (nucleoside transporters), member 2	1.43
ENSMUSG00000026779	Mastl	microtubule associated serine/threonine kinase-like	1.42
ENSMUSG00000031629	Cenpu	centromere protein U	1.42
ENSMUSG00000028718	Stil	Scl/Tal1 interrupting locus	1.41

ENSMUSG00000021175	Cdca7l	cell division cycle associated 7 like	1.41
ENSMUSG00000032344	Mb21d1	Mab-21 domain containing 1	1.41
ENSMUSG00000037466	Tedc1	tubulin epsilon and delta complex 1	1.41
ENSMUSG00000039055	Eme1	essential meiotic structure-specific endonuclease 1	1.41
ENSMUSG00000075266	Cenpw	centromere protein W	1.41
ENSMUSG00000056394	Lig1	ligase I, DNA, ATP-dependent	1.40
ENSMUSG00000020534	Shmt1	serine hydroxymethyltransferase 1 (soluble)	1.40
ENSMUSG00000068101	Cenpm	centromere protein M	1.40
ENSMUSG00000027353	Mcm8	minichromosome maintenance 8 homologous recombination repair factor	1.40
ENSMUSG00000063018	2010204K13Rik	RIKEN cDNA 2010204K13 gene	1.39
ENSMUSG00000091021	Gm17300	predicted gene, 17300	1.39
ENSMUSG00000039200	Atf7ip2	activating transcription factor 7 interacting protein 2	1.39
ENSMUSG00000106883	Gm34091	predicted gene, 34091	1.39
ENSMUSG00000022177	Haus4	HAUS augmin-like complex, subunit 4	1.38
ENSMUSG00000038644	Pold1	polymerase (DNA directed), delta 1, catalytic subunit	1.38
ENSMUSG00000034206	Polq	polymerase (DNA directed), theta	1.38
ENSMUSG00000082163	Gm14276	predicted gene 14276	1.38
ENSMUSG00000006585	Cdt1	chromatin licensing and DNA replication factor 1	1.37
ENSMUSG00000027018	Hat1	histone aminotransferase 1	1.37
ENSMUSG00000007080	Pole	polymerase (DNA directed), epsilon	1.37
ENSMUSG00000022881	Rfc4	replication factor C (activator 1) 4	1.37
ENSMUSG00000074825	Itpripl1	inositol 1,4,5-triphosphate receptor interacting protein-like 1	1.37
ENSMUSG00000116542	Gm17783	predicted, 17783	1.37
ENSMUSG00000030528	Blm	Bloom syndrome, RecQ like helicase	1.37
ENSMUSG00000098090	2700099C18Rik	RIKEN cDNA 2700099C18 gene	1.37
ENSMUSG00000041219	Arhgap11a	Rho GTPase activating protein 11A	1.36
ENSMUSG00000025403	Shmt2	serine hydroxymethyltransferase 2 (mitochondrial)	1.36
ENSMUSG00000091405	Hist2h4	histone cluster 2, H4	1.36
ENSMUSG00000030641	Ddias	DNA damage-induced apoptosis suppressor	1.36
ENSMUSG00000050107	Haspin	histone H3 associated protein kinase	1.35
ENSMUSG00000026196	Bard1	BRCA1 associated RING domain 1	1.35
ENSMUSG00000029414	Kntc1	kinetochore associated 1	1.35
ENSMUSG00000044005	Gls2	glutaminase 2 (liver, mitochondrial)	1.35
ENSMUSG00000098318	Lockd	lncRNA downstream of Cdkn1b	1.35
ENSMUSG00000022673	Mcm4	minichromosome maintenance complex component 4	1.34
ENSMUSG00000032815	Fanca	Fanconi anemia, complementation group A	1.34
ENSMUSG00000022364	Tbc1d31	TBC1 domain family, member 31	1.34
ENSMUSG00000036875	Dna2	DNA replication helicase/nuclease 2	1.34
ENSMUSG00000087060	Eldr	Egfr long non-coding downstream RNA	1.34
ENSMUSG00000028602	Tnfrsf8	tumor necrosis factor receptor superfamily, member 8	1.34
ENSMUSG00000019961	Tmpo	thymopoietin	1.33
ENSMUSG00000028896	Rcc1	regulator of chromosome condensation 1	1.33
ENSMUSG00000029730	Mcm7	minichromosome maintenance complex component 7	1.33
ENSMUSG00000001056	Nhp2	NHP2 ribonucleoprotein	1.33
ENSMUSG00000040034	Nup43	nucleoporin 43	1.33
ENSMUSG00000022945	Chaf1b	chromatin assembly factor 1, subunit B (p60)	1.33
ENSMUSG00000028066	Pmf1	polyamine-modulated factor 1	1.32
ENSMUSG00000026434	Nucks1	nuclear casein kinase and cyclin-dependent kinase substrate 1	1.32
ENSMUSG00000005732	Ranbp1	RAN binding protein 1	1.32
ENSMUSG00000004880	Lbr	lamin B receptor	1.32
ENSMUSG00000100210	Hist1h3f	histone cluster 1, H3f	1.32
ENSMUSG00000044702	Palb2	partner and localizer of BRCA2	1.32
ENSMUSG00000033752	Mnd1	meiotic nuclear divisions 1	1.32
ENSMUSG00000083921	Gm15750	predicted gene 15750	1.32
ENSMUSG00000097180	2700038G22Rik	RIKEN cDNA 2700038G22 gene	1.32
ENSMUSG00000099474	1700097N02Rik	RIKEN cDNA 1700097N02 gene	1.32
ENSMUSG00000078586	Gm11735	predicted gene 11735	1.32
ENSMUSG00000086721	Gm13750	predicted gene 13750	1.32
ENSMUSG00000028693	Nasp	nuclear autoantigenic sperm protein (histone-binding)	1.31
ENSMUSG00000025980	Hspd1	heat shock protein 1 (chaperonin)	1.31
ENSMUSG00000002870	Mcm2	minichromosome maintenance complex component 2	1.31
ENSMUSG00000067455	Hist1h4j	histone cluster 1, H4j	1.31
ENSMUSG00000021391	Cenpp	centromere protein P	1.31
ENSMUSG00000073184	Gm10479	predicted gene 10479	1.31
ENSMUSG00000028884	Rpa2	replication protein A2	1.30
ENSMUSG00000002835	Chaf1a	chromatin assembly factor 1, subunit A (p150)	1.30
ENSMUSG00000025001	Hells	helicase, lymphoid specific	1.30
ENSMUSG00000037020	Wdr62	WD repeat domain 62	1.30
ENSMUSG00000022325	Pop1	processing of precursor 1, ribonuclease P/MRP family, (S. cerevisiae)	1.30
ENSMUSG00000029283	Cdc7	cell division cycle 7 (S. cerevisiae)	1.30
ENSMUSG00000053398	Phgdh	3-phosphoglycerate dehydrogenase	1.30
ENSMUSG00000030929	Eri2	exoribonuclease 2	1.30
ENSMUSG00000027654	Fam83d	family with sequence similarity 83, member D	1.30

ENSMUSG00000097651	4930461G14Rik	RIKEN cDNA 4930461G14 gene	1.30
ENSMUSG00000031762	Mt2	metallothionein 2	1.30
ENSMUSG00000109245	Gm44860	predicted gene 44860	1.30
ENSMUSG00000112406	4921515L22Rik	RIKEN cDNA 4921515L22 gene	1.30
ENSMUSG00000021115	Vrk1	vaccinia related kinase 1	1.29
ENSMUSG00000026355	Mcm6	minichromosome maintenance complex component 6	1.29
ENSMUSG00000060981	Hist1h4h	histone cluster 1, H4h	1.29
ENSMUSG00000027752	Exosc8	exosome component 8	1.29
ENSMUSG00000025395	Prim1	DNA primase, p49 subunit	1.29
ENSMUSG00000019992	Mtfr2	mitochondrial fission regulator 2	1.29
ENSMUSG00000081740	Gm14279	predicted gene 14279	1.29
ENSMUSG00000047246	Hist1h2be	histone cluster 1, H2be	1.28
ENSMUSG00000101972	Hist1h3i	histone cluster 1, H3i	1.28
ENSMUSG00000001525	Tubb5	tubulin, beta 5 class I	1.27
ENSMUSG00000034317	Trim59	tripartite motif-containing 59	1.27
ENSMUSG00000076437	Selenoh	selenoprotein H	1.27
ENSMUSG00000031403	Dkc1	dyskeratosis congenita 1, dyskerin	1.27
ENSMUSG00000105987	AI506816	expressed sequence AI506816	1.27
ENSMUSG00000022360	Atad2	ATPase family, AAA domain containing 2	1.27
ENSMUSG00000047443	Erfe	erythroferrone	1.27
ENSMUSG00000028044	Cks1b	CDC28 protein kinase 1b	1.27
ENSMUSG00000030254	Rad18	RAD18 E3 ubiquitin protein ligase	1.27
ENSMUSG00000020649	Rrm2	ribonucleotide reductase M2	1.27
ENSMUSG00000009248	Ascl2	achaete-scute family bHLH transcription factor 2	1.27
ENSMUSG00000048779	P2ry6	pyrimidinergic receptor P2Y, G-protein coupled, 6	1.27
ENSMUSG00000012483	Rpa3	replication protein A3	1.26
ENSMUSG00000029366	Dck	deoxycytidine kinase	1.26
ENSMUSG00000030079	Ruvbl1	RuvB-like protein 1	1.25
ENSMUSG00000035165	Kcne3	potassium voltage-gated channel, Isk-related subfamily, gene 3	1.25
ENSMUSG00000024691	Fam111a	family with sequence similarity 111, member A	1.25
ENSMUSG00000022978	Mis18a	MIS18 kinetochore protein A	1.25
ENSMUSG00000038046	Mrm3	mitochondrial rRNA methyltransferase 3	1.25
ENSMUSG00000036526	Card11	caspase recruitment domain family, member 11	1.25
ENSMUSG00000097412	1810014B01Rik	RIKEN cDNA 1810014B01 gene	1.25
ENSMUSG00000078952	Lncenc1	long non-coding RNA, embryonic stem cells expressed 1	1.25
ENSMUSG00000066551	Hmgb1	high mobility group box 1	1.24
ENSMUSG00000042029	Ncapg2	non-SMC condensin II complex, subunit G2	1.24
ENSMUSG00000057113	Npm1	nucleophosmin 1	1.24
ENSMUSG00000021048	Mthfd1	methylenetetrahydrofolate dehydrogenase (NADP+ dependent), methenyltetrahydrofolate cyclohyc	1.24
ENSMUSG00000022792	Yars2	tyrosyl-tRNA synthetase 2 (mitochondrial)	1.24
ENSMUSG00000067367	Lyar	Ly1 antibody reactive clone	1.24
ENSMUSG00000028587	Orc1	origin recognition complex, subunit 1	1.24
ENSMUSG00000109936	Gm45889	predicted gene 45889	1.24
ENSMUSG00000044320	1700001O22Rik	RIKEN cDNA 1700001O22 gene	1.24
ENSMUSG00000060794	Tssk5	testis-specific serine kinase 5	1.24
ENSMUSG00000022962	Gart	phosphoribosylglycinamide formyltransferase	1.23
ENSMUSG00000027405	Nop56	NOP56 ribonucleoprotein	1.23
ENSMUSG00000026234	Ncl	nucleolin	1.23
ENSMUSG00000031697	Orc6	origin recognition complex, subunit 6	1.23
ENSMUSG00000022026	Olfm4	olfactomedin 4	1.23
ENSMUSG00000039748	Exo1	exonuclease 1	1.23
ENSMUSG00000026785	Pkn3	protein kinase N3	1.23
ENSMUSG00000045709	Smkr-ps	smal lysine rich protein 1, pseudogene	1.23
ENSMUSG00000116009	Gm49503	predicted gene, 49503	1.23
ENSMUSG00000025991	Cps1	carbamoyl-phosphate synthetase 1	1.22
ENSMUSG00000096010	Hist4h4	histone cluster 4, H4	1.22
ENSMUSG00000039298	Cdk5rap2	CDK5 regulatory subunit associated protein 2	1.22
ENSMUSG00000032555	Topbp1	topoisomerase (DNA) II binding protein 1	1.22
ENSMUSG00000027424	Mgme1	mitochondrial genome maintenance exonuclease 1	1.22
ENSMUSG00000086513	9130208D14Rik	RIKEN cDNA 9130208D14 gene	1.22
ENSMUSG00000025912	Mybl1	myeloblastosis oncogene-like 1	1.22
ENSMUSG00000022754	Tmem45a	transmembrane protein 45a	1.22
ENSMUSG00000081151	Gm11448	predicted gene 11448	1.22
ENSMUSG00000052833	Sae1	SUMO1 activating enzyme subunit 1	1.21
ENSMUSG00000004099	Dnmt1	DNA methyltransferase (cytosine-5) 1	1.21
ENSMUSG00000050410	Tcf19	transcription factor 19	1.21
ENSMUSG00000020974	Pole2	polymerase (DNA directed), epsilon 2 (p59 subunit)	1.21
ENSMUSG00000014633	Cmc2	COX assembly mitochondrial protein 2	1.21
ENSMUSG00000047757	Fancb	Fanconi anemia, complementation group B	1.21
ENSMUSG00000114515	Aldoa	aldolase A, fructose-bisphosphate	1.21
ENSMUSG00000060777	Gm10087	predicted gene 10087	1.21
ENSMUSG00000029430	Ran	RAN, member RAS oncogene family	1.20
ENSMUSG00000044254	Pcsk9	proprotein convertase subtilisin/kexin type 9	1.20

ENSMUSG00000015749	Anp32e	acidic (leucine-rich) nuclear phosphoprotein 32 family, member E	1.20
ENSMUSG00000003868	Ruvbl2	RuvB-like protein 2	1.20
ENSMUSG00000017493	Igfbp4	insulin-like growth factor binding protein 4	1.20
ENSMUSG00000039356	Exosc2	exosome component 2	1.20
ENSMUSG00000022797	Tfrc	transferrin receptor	1.20
ENSMUSG00000069793	Slfn9	schlafen 9	1.20
ENSMUSG00000041193	Pla2g5	phospholipase A2, group V	1.20
ENSMUSG00000019214	Chtf18	CTF18, chromosome transmission fidelity factor 18	1.20
ENSMUSG00000027454	Gins1	GINS complex subunit 1 (Psf1 homolog)	1.20
ENSMUSG00000073415	Gm10501	predicted gene 10501	1.20
ENSMUSG00000018446	C1qbp	complement component 1, q subcomponent binding protein	1.19
ENSMUSG00000034192	Lsm3	LSM3 homolog, U6 small nuclear RNA and mRNA degradation associated	1.19
ENSMUSG00000006715	Gmnn	geminin	1.19
ENSMUSG00000053801	Grwd1	glutamate-rich WD repeat containing 1	1.19
ENSMUSG00000097415	AU020206	expressed sequence AU020206	1.19
ENSMUSG00000056904	Gm5620	predicted gene 5620	1.19
ENSMUSG00000037148	Arhgap10	Rho GTPase activating protein 10	1.19
ENSMUSG00000035818	Plekhs1	pleckstrin homology domain containing, family S member 1	1.19
ENSMUSG00000068220	Lgals1	lectin, galactose binding, soluble 1	1.19
ENSMUSG00000071516	Hist1h2ai	histone cluster 1, H2ai	1.19
ENSMUSG00000097755	2010110K18Rik	RIKEN cDNA 2010110K18 gene	1.19
ENSMUSG00000042606	Hirip3	HIRA interacting protein 3	1.18
ENSMUSG00000039231	Suv39h1	suppressor of variegation 3-9 1	1.18
ENSMUSG00000038323	1700066M21Rik	RIKEN cDNA 1700066M21 gene	1.18
ENSMUSG00000021418	Rpp40	ribonuclease P 40 subunit	1.18
ENSMUSG00000079139	Gm4204	predicted gene 4204	1.18
ENSMUSG00000022096	Hr	hairless	1.17
ENSMUSG00000002477	Snrpd1	small nuclear ribonucleoprotein D1	1.17
ENSMUSG00000002718	Cse1l	chromosome segregation 1-like (S. cerevisiae)	1.17
ENSMUSG00000032939	Nup93	nucleoporin 93	1.17
ENSMUSG00000068394	Cep152	centrosomal protein 152	1.17
ENSMUSG00000025507	Pidd1	p53 induced death domain protein 1	1.17
ENSMUSG00000027395	Polr1b	polymerase (RNA) I polypeptide B	1.17
ENSMUSG00000014907	Naf1	nuclear assembly factor 1 ribonucleoprotein	1.17
ENSMUSG00000025742	Prps2	phosphoribosyl pyrophosphate synthetase 2	1.17
ENSMUSG00000005360	Slc1a3	solute carrier family 1 (glial high affinity glutamate transporter), member 3	1.17
ENSMUSG00000041840	Haus1	HAUS augmin-like complex, subunit 1	1.17
ENSMUSG00000063445	Nmral1	NmrA-like family domain containing 1	1.17
ENSMUSG00000060950	Trmt61a	tRNA methyltransferase 61A	1.17
ENSMUSG00000003824	Syce2	synaptonemal complex central element protein 2	1.17
ENSMUSG00000078502	Gm13212	predicted gene 13212	1.17
ENSMUSG00000042854	Trp53rkb	transformation related protein 53 regulating kinase B	1.17
ENSMUSG00000062588	Gm6104	predicted gene 6104	1.17
ENSMUSG00000064672	Gm22806	predicted gene, 22806	1.17
ENSMUSG00000069273	Hist1h3e	histone cluster 1, H3e	1.16
ENSMUSG00000041506	Rrp9	RRP9, small subunit (SSU) processome component, homolog (yeast)	1.16
ENSMUSG00000017146	Brca1	breast cancer 1, early onset	1.16
ENSMUSG00000061533	Cep128	centrosomal protein 128	1.16
ENSMUSG00000086421	Gm14091	predicted gene 14091	1.16
ENSMUSG00000057244	Gm6139	predicted gene 6139	1.16
ENSMUSG00000022346	Myc	myelocytomatosis oncogene	1.15
ENSMUSG00000005481	Ddx39	DEAD (Asp-Glu-Ala-Asp) box polypeptide 39	1.15
ENSMUSG00000028333	Anp32b	acidic (leucine-rich) nuclear phosphoprotein 32 family, member B	1.15
ENSMUSG00000024925	Rnaseh2c	ribonuclease H2, subunit C	1.15
ENSMUSG00000026646	Suv39h2	suppressor of variegation 3-9 2	1.15
ENSMUSG00000059939	9430015G10Rik	RIKEN cDNA 9430015G10 gene	1.15
ENSMUSG00000060639	Hist1h4i	histone cluster 1, H4i	1.15
ENSMUSG00000079685	Ulbp1	UL16 binding protein 1	1.15
ENSMUSG00000020773	Trim47	tripartite motif-containing 47	1.15
ENSMUSG00000017607	Tns4	tensin 4	1.14
ENSMUSG00000024844	Banf1	barrier to autointegration factor 1	1.14
ENSMUSG00000032373	Car12	carbonic anhydrase 12	1.14
ENSMUSG00000095567	Noc2l	NOC2 like nucleolar associated transcriptional repressor	1.14
ENSMUSG00000033307	Mif	macrophage migration inhibitory factor (glycosylation-inhibiting factor)	1.14
ENSMUSG00000022913	Psmg1	proteasome (prosome, macropain) assembly chaperone 1	1.14
ENSMUSG00000025732	Mcrip2	MAPK regulated corepressor interacting protein 2	1.14
ENSMUSG00000021714	Cenpk	centromere protein K	1.14
ENSMUSG00000109685	Gm45912	predicted gene 45912	1.14
ENSMUSG00000044827	Tlr1	toll-like receptor 1	1.14
ENSMUSG00000069308	Hist1h2bp	histone cluster 1, H2bp	1.14
ENSMUSG00000028560	Usp1	ubiquitin specific peptidase 1	1.13
ENSMUSG00000060860	Ube2s	ubiquitin-conjugating enzyme E2S	1.13
ENSMUSG00000070348	Ccnd1	cyclin D1	1.13

ENSMUSG00000015176	Nolc1	nucleolar and coiled-body phosphoprotein 1	1.13
ENSMUSG00000030662	Ipo5	importin 5	1.13
ENSMUSG00000037275	Gemin5	gem nuclear organelle associated protein 5	1.13
ENSMUSG00000022070	Bora	bora, aurora kinase A activator	1.13
ENSMUSG00000015217	Hmgb3	high mobility group box 3	1.13
ENSMUSG00000057541	Pus7	pseudouridylate synthase 7	1.13
ENSMUSG00000013629	Cad	carbamoyl-phosphate synthetase 2, aspartate transcarbamylase, and dihydroorotase	1.13
ENSMUSG00000097769	Snhg4	small nucleolar RNA host gene 4	1.13
ENSMUSG00000035960	Apex1	apurinic/apyrimidinic endonuclease 1	1.13
ENSMUSG00000025665	Rps6ka6	ribosomal protein S6 kinase polypeptide 6	1.13
ENSMUSG00000026238	Ptma	prothymosin alpha	1.12
ENSMUSG00000027342	Pcna	proliferating cell nuclear antigen	1.12
ENSMUSG00000054766	Set	SET nuclear oncogene	1.12
ENSMUSG00000061482	Hist1h4d	histone cluster 1, H4d	1.12
ENSMUSG00000036109	Mbnl3	muscleblind like splicing factor 3	1.12
ENSMUSG00000028633	Ctps	cytidine 5'-triphosphate synthase	1.12
ENSMUSG00000023908	Pkmyt1	protein kinase, membrane associated tyrosine/threonine 1	1.12
ENSMUSG00000055760	Gemin6	gem nuclear organelle associated protein 6	1.12
ENSMUSG00000059493	Nhs	NHS actin remodeling regulator	1.12
ENSMUSG00000035439	Haus8	4HAUS augmin-like complex, subunit 8	1.12
ENSMUSG00000052852	Reep1	receptor accessory protein 1	1.12
ENSMUSG00000026192	Atic	5-aminoimidazole-4-carboxamide ribonucleotide formyltransferase/IMP cyclohydrolase	1.11
ENSMUSG00000054321	Taf4b	TATA-box binding protein associated factor 4b	1.11
ENSMUSG00000020706	Ftsj3	FtsJ RNA methyltransferase homolog 3 (E. coli)	1.11
ENSMUSG00000046711	Hmga1	high mobility group AT-hook 1	1.11
ENSMUSG00000026281	Dtymk	deoxythymidylate kinase	1.11
ENSMUSG00000111877	Gm6477	predicted gene 6477	1.11
ENSMUSG00000040463	Mybbp1a	MYB binding protein (P160) 1a	1.11
ENSMUSG00000033706	Smyd5	SET and MYND domain containing 5	1.11
ENSMUSG00000036678	Aaas	achalasia, adrenocortical insufficiency, alacrimia	1.11
ENSMUSG00000023348	Trip6	thyroid hormone receptor interactor 6	1.11
ENSMUSG00000080928	Hmgb1-ps6	high mobility group box 1, pseudogene 6	1.11
ENSMUSG00000103576	Gm38188	predicted gene, 38188	1.11
ENSMUSG00000024640	Psat1	phosphoserine aminotransferase 1	1.10
ENSMUSG00000020739	Nup85	nucleoporin 85	1.10
ENSMUSG00000003438	Timm50	translocase of inner mitochondrial membrane 50	1.10
ENSMUSG00000021377	Dek	DEK oncogene (DNA binding)	1.10
ENSMUSG00000023110	Prmt5	protein arginine N-methyltransferase 5	1.10
ENSMUSG00000050244	Heatr1	HEAT repeat containing 1	1.10
ENSMUSG00000091625	Lsm5	LSM5 homolog, U6 small nuclear RNA and mRNA degradation associated	1.10
ENSMUSG00000037474	Dtl	denticleless E3 ubiquitin protein ligase	1.10
ENSMUSG00000054115	Skp2	S-phase kinase-associated protein 2 (p45)	1.10
ENSMUSG00000026404	Ddx59	DEAD (Asp-Glu-Ala-Asp) box polypeptide 59	1.10
ENSMUSG00000073627	C130036L24Rik	RIKEN cDNA C130036L24 gene	1.10
ENSMUSG00000106358	Gm7047	predicted gene 7047	1.10
ENSMUSG00000097177	9330159M07Rik	RIKEN cDNA 9330159M07 gene	1.10
ENSMUSG00000038685	Rtel1	regulator of telomere elongation helicase 1	1.09
ENSMUSG00000030512	Snrpa1	small nuclear ribonucleoprotein polypeptide A'	1.09
ENSMUSG00000109511	Nup62	nucleoporin 62	1.09
ENSMUSG00000027067	Ssrp1	structure specific recognition protein 1	1.09
ENSMUSG00000004356	Utp20	UTP20 small subunit processome component	1.09
ENSMUSG00000027160	Ccdc34	coiled-coil domain containing 34	1.09
ENSMUSG00000026020	Nop58	NOP58 ribonucleoprotein	1.09
ENSMUSG00000070304	Scn2b	sodium channel, voltage-gated, type II, beta	1.09
ENSMUSG00000019982	Myb	myeloblastosis oncogene	1.09
ENSMUSG00000053553	3110082I17Rik	RIKEN cDNA 3110082I17 gene	1.09
ENSMUSG00000024800	Rpp30	ribonuclease P/MRP 30 subunit	1.09
ENSMUSG00000097493	9930014A18Rik	RIKEN cDNA 9930014A18 gene	1.09
ENSMUSG00000039254	Pomt1	protein-O-mannosyltransferase 1	1.09
ENSMUSG00000022748	Cmss1	cms small ribosomal subunit 1	1.09
ENSMUSG00000078941	Ak6	adenylate kinase 6	1.09
ENSMUSG00000068855	Hist2h2ac	histone cluster 2, H2ac	1.09
ENSMUSG00000115306	Gm49159	predicted gene, 49159	1.09
ENSMUSG00000025364	Pa2g4	proliferation-associated 2G4	1.08
ENSMUSG00000033166	Dis3	DIS3 homolog, exosome endoribonuclease and 3'-5' exoribonuclease	1.08
ENSMUSG00000025134	Alyref	Aly/REF export factor	1.08
ENSMUSG00000014226	Cacybp	calcyclin binding protein	1.08
ENSMUSG00000058799	Nap1l1	nucleosome assembly protein 1-like 1	1.08
ENSMUSG00000040681	Hmgn1	high mobility group nucleosomal binding domain 1	1.08
ENSMUSG00000032459	Mrps22	mitochondrial ribosomal protein S22	1.08
ENSMUSG00000097195	Snhg5	small nucleolar RNA host gene 5	1.08
ENSMUSG00000036285	Noa1	nitric oxide associated 1	1.08
ENSMUSG00000026999	Nup35	nucleoporin 35	1.08

ENSMUSG00000021177	Tdp1	tyrosyl-DNA phosphodiesterase 1	1.08
ENSMUSG00000036639	Nudt1	nudix (nucleoside diphosphate linked moiety X)-type motif 1	1.08
ENSMUSG00000021572	Cep72	centrosomal protein 72	1.08
ENSMUSG00000017832	Hspb9	heat shock protein, alpha-crystallin-related, B9	1.08
ENSMUSG00000034424	Gcsh	glycine cleavage system protein H (aminomethyl carrier)	1.07
ENSMUSG00000021193	Pitrm1	pitrilysin metallepetidase 1	1.07
ENSMUSG00000018921	Pelp1	proline, glutamic acid and leucine rich protein 1	1.07
ENSMUSG00000024833	Pola2	polymerase (DNA directed), alpha 2	1.07
ENSMUSG00000020899	Pfas	phosphoribosylformylglycinamidine synthase (FGAR amidotransferase)	1.07
ENSMUSG00000031453	Rasa3	RAS p21 protein activator 3	1.07
ENSMUSG00000109324	Prmt1	protein arginine N-methyltransferase 1	1.07
ENSMUSG00000023066	Rttn	rotatin	1.07
ENSMUSG00000042215	Bag2	BCL2-associated athanogene 2	1.07
ENSMUSG00000028391	Wdr31	WD repeat domain 31	1.07
ENSMUSG00000062480	Acat3	acetyl-Coenzyme A acetyltransferase 3	1.07
ENSMUSG00000057469	E2f6	E2F transcription factor 6	1.06
ENSMUSG00000025869	Nop16	NOP16 nucleolar protein	1.06
ENSMUSG00000026709	Dars2	aspartyl-tRNA synthetase 2 (mitochondrial)	1.06
ENSMUSG00000025962	Fastkd2	FAST kinase domains 2	1.06
ENSMUSG00000060288	Ppih	peptidyl prolyl isomerase H	1.06
ENSMUSG00000017499	Cdc6	cell division cycle 6	1.06
ENSMUSG00000027076	Timm10	translocase of inner mitochondrial membrane 10	1.06
ENSMUSG00000056209	Npm3	nucleoplasmin 3	1.06
ENSMUSG00000031821	Gins2	GINS complex subunit 2 (Psf2 homolog)	1.06
ENSMUSG00000024525	Impa2	inositol (myo)-1(or 4)-monophosphatase 2	1.06
ENSMUSG00000079478	Sssca1	Sjogren syndrome/scleroderma autoantigen 1	1.06
ENSMUSG00000014075	Tctex1d2	Tctex1 domain containing 2	1.06
ENSMUSG00000026322	Htr4	5 hydroxytryptamine (serotonin) receptor 4	1.06
ENSMUSG00000103445	Gm36948	predicted gene, 36948	1.06
ENSMUSG00000022698	Naa50	N(alpha)-acetyltransferase 50, NatE catalytic subunit	1.05
ENSMUSG00000005899	Smpd4	sphingomyelin phosphodiesterase 4	1.05
ENSMUSG00000047832	Cdca4	cell division cycle associated 4	1.05
ENSMUSG00000044934	Zfp367	zinc finger protein 367	1.05
ENSMUSG00000026019	Wdr12	WD repeat domain 12	1.05
ENSMUSG00000024411	Aqp4	aquaporin 4	1.05
ENSMUSG00000031669	Gins3	GINS complex subunit 3 (Psf3 homolog)	1.05
ENSMUSG00000025086	Trub1	TruB pseudouridine (psi) synthase family member 1	1.05
ENSMUSG00000085156	Snhg15	small nucleolar RNA host gene 15	1.05
ENSMUSG00000084129	Hmgb1-ps1	high mobility group box 1, pseudogene 1	1.05
ENSMUSG00000032446	Eomes	eomesodermin	1.05
ENSMUSG00000025026	Add3	adducin 3 (gamma)	1.04
ENSMUSG00000029036	Atad3a	ATPase family, AAA domain containing 3A	1.04
ENSMUSG00000062867	Impdh2	inosine monophosphate dehydrogenase 2	1.04
ENSMUSG00000024732	Ccdc86	coiled-coil domain containing 86	1.04
ENSMUSG00000031672	Got2	glutamatic-oxaloacetic transaminase 2, mitochondrial	1.04
ENSMUSG00000029246	Ppat	phosphoribosyl pyrophosphate amidotransferase	1.04
ENSMUSG00000024785	Rcl1	RNA terminal phosphate cyclase-like 1	1.04
ENSMUSG00000020471	Pold2	polymerase (DNA directed), delta 2, regulatory subunit	1.04
ENSMUSG00000058392	Rrp1b	ribosomal RNA processing 1 homolog B (S. cerevisiae)	1.04
ENSMUSG00000002068	Ccne1	cyclin E1	1.04
ENSMUSG00000026676	Ccdc3	coiled-coil domain containing 3	1.04
ENSMUSG00000034329	Brip1	BRCA1 interacting protein C-terminal helicase 1	1.04
ENSMUSG00000024078	Ttc27	tetratricopeptide repeat domain 27	1.04
ENSMUSG00000032113	Chek1	checkpoint kinase 1	1.04
ENSMUSG00000056305	Usp39	ubiquitin specific peptidase 39	1.04
ENSMUSG00000028212	Ccne2	cyclin E2	1.04
ENSMUSG00000023919	Cenpq	centromere protein Q	1.04
ENSMUSG00000030513	Pcsk6	proprotein convertase subtilisin/kexin type 6	1.04
ENSMUSG00000056531	Ccdc18	coiled-coil domain containing 18	1.04
ENSMUSG00000095677	Dynlt1f	dynein light chain Tctex-type 1F	1.04
ENSMUSG00000040321	Zfp770	zinc finger protein 770	1.04
ENSMUSG00000090602	Gm5611	predicted gene 5611	1.04
ENSMUSG00000106926	Rpl7-ps7	ribosomal protein L7, pseudogene 7	1.04
ENSMUSG00000043424	Eif3j2	eukaryotic translation initiation factor 3, subunit J2	1.04
ENSMUSG00000020290	Xpo1	exportin 1	1.03
ENSMUSG00000061479	Snrpa	small nuclear ribonucleoprotein polypeptide A	1.03
ENSMUSG00000038759	Nup205	nucleoporin 205	1.03
ENSMUSG00000061718	Ppp1r1b	protein phosphatase 1, regulatory inhibitor subunit 1B	1.03
ENSMUSG00000034203	Chchd4	coiled-coil-helix-coiled-coil-helix domain containing 4	1.03
ENSMUSG00000024542	Cep192	centrosomal protein 192	1.03
ENSMUSG00000038510	Rpf2	ribosome production factor 2 homolog	1.03
ENSMUSG00000020918	Kat2a	K(lysine) acetyltransferase 2A	1.03
ENSMUSG00000038845	Phb	prohibitin	1.03

ENSMUSG00000055612	Cdca7	cell division cycle associated 7	1.03
ENSMUSG00000033294	Noc4l	NOC4 like	1.03
ENSMUSG00000063480	Snu13	SNU13 homolog, small nuclear ribonucleoprotein (U4/U6.U5)	1.03
ENSMUSG00000105703	Gm43305	predicted gene 43305	1.03
ENSMUSG00000056032	BC018473	cDNA sequence BC018473	1.03
ENSMUSG00000105077	Gm4859	predicted gene 4859	1.03
ENSMUSG00000068631	Gm7676	predicted gene 7676	1.03
ENSMUSG00000114374	Gm7911	predicted gene 7911	1.03
ENSMUSG00000042354	Gnl3	guanine nucleotide binding protein-like 3 (nucleolar)	1.02
ENSMUSG00000022678	Nde1	nudE neurodevelopment protein 1	1.02
ENSMUSG00000020741	Cluh	clustered mitochondria (cluA/CLU1) homolog	1.02
ENSMUSG00000063052	Lrrc40	leucine rich repeat containing 40	1.02
ENSMUSG00000018583	G3bp1	GTPase activating protein (SH3 domain) binding protein 1	1.02
ENSMUSG00000020929	Eftud2	elongation factor Tu GTP binding domain containing 2	1.02
ENSMUSG00000014444	Piezo1	piezo-type mechanosensitive ion channel component 1	1.02
ENSMUSG00000032834	Pwp2	PWP2 periodic tryptophan protein homolog (yeast)	1.02
ENSMUSG00000046791	Riox1	ribosomal oxygenase 1	1.02
ENSMUSG00000028741	Mrto4	mRNA turnover 4, ribosome maturation factor	1.02
ENSMUSG00000040749	Siah1b	siah E3 ubiquitin protein ligase 1B	1.02
ENSMUSG00000035606	Ky	kyphoscoliosis peptidase	1.02
ENSMUSG00000081205	Gm5940	predicted gene 5940	1.02
ENSMUSG00000041057	Wdr43	WD repeat domain 43	1.01
ENSMUSG00000073676	Hspe1	heat shock protein 1 (chaperonin 10)	1.01
ENSMUSG00000019297	Nop9	NOP9 nucleolar protein	1.01
ENSMUSG00000022391	Rangap1	RAN GTPase activating protein 1	1.01
ENSMUSG00000001785	Pwp1	PWP1 homolog, endonuclein	1.01
ENSMUSG00000038047	Haus6	HAUS augmin-like complex, subunit 6	1.01
ENSMUSG00000078713	Tomm5	translocase of outer mitochondrial membrane 5	1.01
ENSMUSG00000032092	Mpzl2	myelin protein zero-like 2	1.01
ENSMUSG00000029703	Lrwd1	leucine-rich repeats and WD repeat domain containing 1	1.01
ENSMUSG00000031388	Naa10	N(alpha)-acetyltransferase 10, NatA catalytic subunit	1.01
ENSMUSG00000032397	Tipin	timeless interacting protein	1.01
ENSMUSG00000086290	Snhg12	small nucleolar RNA host gene 12	1.01
ENSMUSG00000073771	Btbd19	BTB (POZ) domain containing 19	1.01
ENSMUSG00000035215	Lsm7	LSM7 homolog, U6 small nuclear RNA and mRNA degradation associated	1.01
ENSMUSG00000062981	Mrpl42	mitochondrial ribosomal protein L42	1.00
ENSMUSG00000001674	Ddx18	DEAD (Asp-Glu-Ala-Asp) box polypeptide 18	1.00
ENSMUSG00000031146	Plp2	proteolipid protein 2	1.00
ENSMUSG00000033222	Ttf2	transcription termination factor, RNA polymerase II	1.00
ENSMUSG00000016984	Etaa1	Ewing tumor-associated antigen 1	1.00
ENSMUSG00000028551	Cdkn2c	cyclin dependent kinase inhibitor 2C	1.00
ENSMUSG00000104350	Gm38244	predicted gene, 38244	-1.00
ENSMUSG00000027313	Chac1	ChaC, cation transport regulator 1	-1.00
ENSMUSG00000092185	Gm20416	predicted gene 20416	-1.00
ENSMUSG00000028059	Arhgef2	rho/rac guanine nucleotide exchange factor (GEF) 2	-1.01
ENSMUSG00000005580	Adcy9	adenylate cyclase 9	-1.01
ENSMUSG00000021097	Clmn	calmin	-1.01
ENSMUSG00000028699	Tspan1	tetraspanin 1	-1.01
ENSMUSG00000002227	Mov10	Moloney leukemia virus 10	-1.01
ENSMUSG00000028755	Cda	cytidine deaminase	-1.01
ENSMUSG00000057092	Fxyd3	FXYD domain-containing ion transport regulator 3	-1.01
ENSMUSG00000096243	Gm24265	predicted gene, 24265	-1.01
ENSMUSG00000025059	Gk	glycerol kinase	-1.01
ENSMUSG00000002083	Bbc3	BCL2 binding component 3	-1.01
ENSMUSG00000050592	Fam78a	family with sequence similarity 78, member A	-1.01
ENSMUSG00000093815	Gm26444	predicted gene, 26444	-1.01
ENSMUSG00000029163	Emilin1	elastin microfibril interfacer 1	-1.01
ENSMUSG00000045382	Cxcr4	chemokine (C-X-C motif) receptor 4	-1.01
ENSMUSG00000083240	Gm13453	predicted gene 13453	-1.01
ENSMUSG00000075420	Smim6	small integral membrane protein 6	-1.02
ENSMUSG00000026628	Atf3	activating transcription factor 3	-1.02
ENSMUSG00000037580	Gch1	GTP cyclohydrolase 1	-1.02
ENSMUSG00000024777	Ppp2r5b	protein phosphatase 2, regulatory subunit B', beta	-1.02
ENSMUSG00000029162	Khk	ketohexokinase	-1.02
ENSMUSG00000024937	Ehbp1l1	EH domain binding protein 1-like 1	-1.02
ENSMUSG00000023045	Soat2	sterol O-acyltransferase 2	-1.02
ENSMUSG00000112944	Gm48885	predicted gene, 48885	-1.02
ENSMUSG00000027456	Sdcbp2	syndecan binding protein (syntenin) 2	-1.02
ENSMUSG00000043621	Ubxn10	UBX domain protein 10	-1.02
ENSMUSG00000024462	Gabbr1	gamma-aminobutyric acid (GABA) B receptor, 1	-1.02
ENSMUSG00000089712	Gm15889	predicted gene 15889	-1.02
ENSMUSG00000035969	Rusc2	RUN and SH3 domain containing 2	-1.02
ENSMUSG00000098224	Gm7985	predicted gene 7985	-1.02

ENSMUSG00000083813	Gm15502	predicted gene 15502	-1.02
ENSMUSG00000052783	Grk4	G protein-coupled receptor kinase 4	-1.02
ENSMUSG00000036036	Zfp57	zinc finger protein 57	-1.02
ENSMUSG00000042417	Ccno	cyclin O	-1.02
ENSMUSG00000044034	Npb	neuropeptide B	-1.02
ENSMUSG00000042632	Pla2g6	phospholipase A2, group VI	-1.03
ENSMUSG00000013160	Atp6v0d1	ATPase, H+ transporting, lysosomal V0 subunit D1	-1.03
ENSMUSG00000037025	Foxa2	forkhead box A2	-1.03
ENSMUSG00000040111	Gramd1b	GRAM domain containing 1B	-1.03
ENSMUSG00000036813	Entpd8	ectonucleoside triphosphate diphosphohydrolase 8	-1.03
ENSMUSG00000022445	Cyp2d26	cytochrome P450, family 2, subfamily d, polypeptide 26	-1.03
ENSMUSG00000053113	Socs3	suppressor of cytokine signaling 3	-1.03
ENSMUSG00000033147	Slc22a15	solute carrier family 22 (organic anion/cation transporter), member 15	-1.03
ENSMUSG00000029674	Limk1	LIM-domain containing, protein kinase	-1.03
ENSMUSG00000031378	Abcd1	ATP-binding cassette, sub-family D (ALD), member 1	-1.03
ENSMUSG00000055675	Kbtbd11	kelch repeat and BTB (POZ) domain containing 11	-1.03
ENSMUSG00000020774	Aspa	aspartoacylase	-1.03
ENSMUSG00000022176	Rem2	rad and gem related GTP binding protein 2	-1.03
ENSMUSG00000096349	Gm22513	predicted gene, 22513	-1.03
ENSMUSG00000093880	Tmem181c-ps	transmembrane protein 181C, pseudogene	-1.03
ENSMUSG00000064215	Ifi27	interferon, alpha-inducible protein 27	-1.03
ENSMUSG00000034687	Fras1	Fraser extracellular matrix complex subunit 1	-1.03
ENSMUSG00000018620	Mmp20	matrix metallopeptidase 20 (enamelysin)	-1.03
ENSMUSG00000034156	Tspoap1	TSPO associated protein 1	-1.03
ENSMUSG00000109679	Gm45342	predicted gene 45342	-1.03
ENSMUSG00000040134	Rdh7	retinol dehydrogenase 7	-1.03
ENSMUSG00000112955	Gm48889	predicted gene, 48889	-1.03
ENSMUSG00000060441	Trim5	tripartite motif-containing 5	-1.03
ENSMUSG00000021363	Mak	male germ cell-associated kinase	-1.03
ENSMUSG00000107182	Gm43268	predicted gene 43268	-1.03
ENSMUSG00000086544	Chn1os3	chimerin 1, opposite strand 3	-1.03
ENSMUSG00000084154	Gm15644	predicted gene 15644	-1.03
ENSMUSG00000030275	Etnk1	ethanolamine kinase 1	-1.04
ENSMUSG00000032899	Styk1	serine/threonine/tyrosine kinase 1	-1.04
ENSMUSG00000038418	Egr1	early growth response 1	-1.04
ENSMUSG00000020777	Acox1	acyl-Coenzyme A oxidase 1, palmitoyl	-1.04
ENSMUSG00000023055	Calcoco1	calcium binding and coiled coil domain 1	-1.04
ENSMUSG00000032098	Treh	trehalase (brush-border membrane glycoprotein)	-1.04
ENSMUSG00000070287	Slc35g2	solute carrier family 35, member G2	-1.04
ENSMUSG00000051427	Ccdc157	coiled-coil domain containing 157	-1.04
ENSMUSG00000032105	Pdzd3	PDZ domain containing 3	-1.04
ENSMUSG00000043789	Vwce	von Willebrand factor C and EGF domains	-1.04
ENSMUSG00000034220	Gpc1	glypican 1	-1.04
ENSMUSG00000031586	Rbpms	RNA binding protein gene with multiple splicing	-1.04
ENSMUSG00000041298	Katnal1	katanin p60 subunit A-like 1	-1.04
ENSMUSG00000108181	C030015A19Rik	RIKEN cDNA C030015A19 gene	-1.04
ENSMUSG00000072915	Gm12258	predicted gene 12258	-1.04
ENSMUSG00000037348	Paqr7	progestin and adipoQ receptor family member VII	-1.04
ENSMUSG00000021057	Akap5	A kinase (PRKA) anchor protein 5	-1.04
ENSMUSG00000055862	Izumo4	IZUMO family member 4	-1.04
ENSMUSG00000024778	Fas	Fas (TNF receptor superfamily member 6)	-1.04
ENSMUSG00000021675	F2rl2	coagulation factor II (thrombin) receptor-like 2	-1.04
ENSMUSG00000000901	Mmp11	matrix metallopeptidase 11	-1.04
ENSMUSG00000029195	Klb	klotho beta	-1.04
ENSMUSG00000096992	Gm26788	predicted gene, 26788	-1.04
ENSMUSG00000114150	Gm46367	predicted gene, 46367	-1.04
ENSMUSG00000116180	Gm49492	predicted gene, 49492	-1.04
ENSMUSG00000040462	Os9	amplified in osteosarcoma	-1.05
ENSMUSG00000031532	Saraf	store-operated calcium entry-associated regulatory factor	-1.05
ENSMUSG00000036246	Gmip	Gem-interacting protein	-1.05
ENSMUSG00000020284	1810043G02Rik	RIKEN cDNA 1810043G02 gene	-1.05
ENSMUSG00000107225	Gm43637	predicted gene 43637	-1.05
ENSMUSG00000037139	Myom3	myomesin family, member 3	-1.05
ENSMUSG00000018931	Natd1	N-acetyltransferase domain containing 1	-1.05
ENSMUSG00000050957	Insl6	insulin-like 6	-1.05
ENSMUSG00000017314	Mpp2	membrane protein, palmitoylated 2 (MAGUK p55 subfamily member 2)	-1.05
ENSMUSG00000097006	9530082P21Rik	RIKEN cDNA 9530082P21 gene	-1.05
ENSMUSG00000107741	Gm2011	predicted gene 2011	-1.05
ENSMUSG00000096924	Gm26824	predicted gene, 26824	-1.05
ENSMUSG00000035279	Ssc5d	scavenger receptor cysteine rich family, 5 domains	-1.05
ENSMUSG00000020120	Plek	pleckstrin	-1.05
ENSMUSG00000044505	Lingo4	leucine rich repeat and Ig domain containing 4	-1.05
ENSMUSG00000054901	Arhgef33	Rho guanine nucleotide exchange factor (GEF) 33	-1.05

ENSMUSG00000038507	Parp12	poly (ADP-ribose) polymerase family, member 12	-1.06
ENSMUSG00000073420	Btnl5-ps	butyrophilin-like 5, pseudogene	-1.06
ENSMUSG00000022565	Plec	plectin	-1.06
ENSMUSG00000034312	Iqsec1	IQ motif and Sec7 domain 1	-1.06
ENSMUSG00000044080	S100a1	S100 calcium binding protein A1	-1.06
ENSMUSG00000041548	Hspb8	heat shock protein 8	-1.06
ENSMUSG00000019577	Pdk4	pyruvate dehydrogenase kinase, isoenzyme 4	-1.06
ENSMUSG00000027524	Edn3	endothelin 3	-1.06
ENSMUSG00000099021	Rn7s1	7S RNA 1	-1.06
ENSMUSG00000113119	Gm48883	predicted gene, 48883	-1.06
ENSMUSG00000035692	Isg15	ISG15 ubiquitin-like modifier	-1.06
ENSMUSG00000115918	A930001M01Rik	RIKEN cDNA A930001M01 gene	-1.06
ENSMUSG00000103037	Pcdhgb1	protocadherin gamma subfamily B, 1	-1.06
ENSMUSG00000053985	Zfp14	zinc finger protein 14	-1.06
ENSMUSG00000026347	Tmem163	transmembrane protein 163	-1.06
ENSMUSG00000104830	5830487J09Rik	RIKEN cDNA 5830487J09 gene	-1.06
ENSMUSG00000026475	Rgs16	regulator of G-protein signaling 16	-1.06
ENSMUSG00000097855	A930007I19Rik	RIKEN cDNA A930007I19 gene	-1.06
ENSMUSG00000113076	Gm47088	predicted gene, 47088	-1.06
ENSMUSG00000096883	Shisa8	shisa family member 8	-1.06
ENSMUSG00000038528	Mfsd4b5	major facilitator superfamily domain containing 4B5	-1.06
ENSMUSG00000022783	Spag6l	sperm associated antigen 6-like	-1.06
ENSMUSG00000033898	Cfhr2	complement factor H-related 2	-1.06
ENSMUSG00000032198	Dock6	dedicator of cytokinesis 6	-1.07
ENSMUSG00000018750	Zbtb4	zinc finger and BTB domain containing 4	-1.07
ENSMUSG00000025192	Entpd7	ectonucleoside triphosphate diphosphohydrolase 7	-1.07
ENSMUSG00000033538	Casp4	caspase 4, apoptosis-related cysteine peptidase	-1.07
ENSMUSG00000033308	Dpyd	dihydropyrimidine dehydrogenase	-1.07
ENSMUSG00000022098	Bmp1	bone morphogenetic protein 1	-1.07
ENSMUSG00000001604	Tcea3	transcription elongation factor A (SII), 3	-1.07
ENSMUSG00000025429	Pstpip2	proline-serine-threonine phosphatase-interacting protein 2	-1.07
ENSMUSG00000053702	Nebl	nebulette	-1.07
ENSMUSG00000020407	Upp1	uridine phosphorylase 1	-1.07
ENSMUSG00000054708	Ankrd24	ankyrin repeat domain 24	-1.07
ENSMUSG00000038298	Pdzk1	PDZ domain containing 1	-1.07
ENSMUSG00000109585	Gm45358	predicted gene 45358	-1.07
ENSMUSG00000085873	Ttc39aos1	Ttc39a opposite strand RNA 1	-1.07
ENSMUSG00000020032	Nuak1	NUAK family, SNF1-like kinase, 1	-1.07
ENSMUSG00000110187	Gm45496	predicted gene 45496	-1.07
ENSMUSG00000002107	Celf2	CUGBP, Elav-like family member 2	-1.07
ENSMUSG00000104576	F830115B05Rik	RIKEN cDNA F830115B05 gene	-1.07
ENSMUSG00000049536	Tceal1	transcription elongation factor A (SII)-like 1	-1.07
ENSMUSG00000038860	Garnl3	GTPase activating RANGAP domain-like 3	-1.07
ENSMUSG00000073608	Gal3st2c	galactose-3-O-sulfotransferase 2C	-1.07
ENSMUSG00000059852	Kcng2	potassium voltage-gated channel, subfamily G, member 2	-1.07
ENSMUSG00000105212	Gm43557	predicted gene 43557	-1.07
ENSMUSG00000085709	Gm14211	predicted gene 14211	-1.07
ENSMUSG00000015291	Gdi1	guanosine diphosphate (GDP) dissociation inhibitor 1	-1.08
ENSMUSG00000023951	Vegfa	vascular endothelial growth factor A	-1.08
ENSMUSG00000020577	Tspan13	tetraspanin 13	-1.08
ENSMUSG00000028555	Ttc39a	tetratricopeptide repeat domain 39A	-1.08
ENSMUSG00000002885	Adgre5	adhesion G protein-coupled receptor E5	-1.08
ENSMUSG00000014361	Mertk	c-mer proto-oncogene tyrosine kinase	-1.08
ENSMUSG00000045038	Prkce	protein kinase C, epsilon	-1.08
ENSMUSG00000025648	Pfkfb4	6-phosphofructo-2-kinase/fructose-2,6-biphosphatase 4	-1.08
ENSMUSG00000030747	Dgat2	diacylglycerol O-acyltransferase 2	-1.08
ENSMUSG00000026773	Pfkfb3	6-phosphofructo-2-kinase/fructose-2,6-biphosphatase 3	-1.08
ENSMUSG00000019558	Slc6a8	solute carrier family 6 (neurotransmitter transporter, creatine), member 8	-1.08
ENSMUSG00000102752	Gm7694	predicted gene 7694	-1.08
ENSMUSG00000028542	Slc6a9	solute carrier family 6 (neurotransmitter transporter, glycine), member 9	-1.08
ENSMUSG00000031919	Tmed6	transmembrane p24 trafficking protein 6	-1.08
ENSMUSG00000073889	Il11ra1	interleukin 11 receptor, alpha chain 1	-1.08
ENSMUSG00000027078	Ube2l6	ubiquitin-conjugating enzyme E2L 6	-1.08
ENSMUSG00000071551	Akr1c19	aldo-keto reductase family 1, member C19	-1.08
ENSMUSG00000086844	B230206H07Rik	RIKEN cDNA B230206H07 gene	-1.08
ENSMUSG00000031844	Hsd17b2	hydroxysteroid (17-beta) dehydrogenase 2	-1.08
ENSMUSG00000032726	Bmp8a	bone morphogenetic protein 8a	-1.08
ENSMUSG00000049612	Omg	oligodendrocyte myelin glycoprotein	-1.08
ENSMUSG00000057137	Tmem140	transmembrane protein 140	-1.08
ENSMUSG00000055214	Pld5	phospholipase D family, member 5	-1.08
ENSMUSG00000086825	Gm15675	predicted gene 15675	-1.08
ENSMUSG00000074628	Tldc2	TBC/LysM associated domain containing 2	-1.08
ENSMUSG00000106547	B230303O12Rik	RIKEN cDNA B230303O12 gene	-1.08

ENSMUSG00000109899	Gm6012	predicted gene 6012	-1.08
ENSMUSG00000081516	Gm12470	predicted gene 12470	-1.08
ENSMUSG00000049515	Espnl	espin-like	-1.08
ENSMUSG00000018830	Myh11	myosin, heavy polypeptide 11, smooth muscle	-1.08
ENSMUSG00000097294	Gm26888	predicted gene, 26888	-1.08
ENSMUSG00000036862	Dchs1	dachsous cadherin related 1	-1.08
ENSMUSG00000001542	Ell2	elongation factor RNA polymerase II 2	-1.09
ENSMUSG00000048970	C1galt1c1	C1GALT1-specific chaperone 1	-1.09
ENSMUSG00000056116	H2-T22	histocompatibility 2, T region locus 22	-1.09
ENSMUSG00000028413	B4galt1	UDP-Gal:betaGlcNAc beta 1,4- galactosyltransferase, polypeptide 1	-1.09
ENSMUSG00000015202	Cnksr3	Cnksr family member 3	-1.09
ENSMUSG00000012123	Crybg2	crystallin beta-gamma domain containing 2	-1.09
ENSMUSG00000041608	Entpd3	ectonucleoside triphosphate diphosphohydrolase 3	-1.09
ENSMUSG00000102440	Pcdhga9	protocadherin gamma subfamily A, 9	-1.09
ENSMUSG00000044681	Cnpy1	canopy FGF signaling regulator 1	-1.09
ENSMUSG00000089960	Ugt1a1	UDP glucuronosyltransferase 1 family, polypeptide A1	-1.09
ENSMUSG00000054404	Slfn5	schlafen 5	-1.09
ENSMUSG00000112459	Gm47689	predicted gene, 47689	-1.09
ENSMUSG00000112303	Gm48759	predicted gene, 48759	-1.09
ENSMUSG00000046242	Nme9	NME/NM23 family member 9	-1.09
ENSMUSG00000051736	Fam229b	family with sequence similarity 229, member B	-1.09
ENSMUSG00000096107	Gm16505	predicted gene 16505	-1.09
ENSMUSG00000022844	Pdia5	protein disulfide isomerase associated 5	-1.10
ENSMUSG00000021360	Gcnt2	glucosaminyl (N-acetyl) transferase 2, I-branching enzyme	-1.10
ENSMUSG00000038781	Stap2	signal transducing adaptor family member 2	-1.10
ENSMUSG00000099746	Ppnr	per-pentamer repeat gene	-1.10
ENSMUSG00000022540	Rogdi	rogdi homolog	-1.10
ENSMUSG00000094830	n-R5s194	nuclear encoded rRNA 5S 194	-1.10
ENSMUSG00000024774	Ankrd22	ankyrin repeat domain 22	-1.10
ENSMUSG00000028631	Kcnq4	potassium voltage-gated channel, subfamily Q, member 4	-1.10
ENSMUSG00000061451	Tmem151a	transmembrane protein 151A	-1.10
ENSMUSG00000030882	Dnhd1	dynein heavy chain domain 1	-1.10
ENSMUSG00000026678	Rgs5	regulator of G-protein signaling 5	-1.10
ENSMUSG00000030589	Rasgrp4	RAS guanyl releasing protein 4	-1.10
ENSMUSG00000060988	Galnt13	polypeptide N-acetylgalactosaminyltransferase 13	-1.10
ENSMUSG00000036760	Kcnk9	potassium channel, subfamily K, member 9	-1.10
ENSMUSG00000104429	2610034O05Rik	RIKEN cDNA 2610034O05 gene	-1.10
ENSMUSG00000043556	Fbxl7	F-box and leucine-rich repeat protein 7	-1.10
ENSMUSG00000078627	40238	membrane-associated ring finger (C3HC4) 10	-1.10
ENSMUSG00000039050	Osbpl2	oxysterol binding protein-like 2	-1.11
ENSMUSG00000037012	Hk1	hexokinase 1	-1.11
ENSMUSG00000024066	Xdh	xanthine dehydrogenase	-1.11
ENSMUSG00000035847	Ids	iduronate 2-sulfatase	-1.11
ENSMUSG00000035699	Slc51a	solute carrier family 51, alpha subunit	-1.11
ENSMUSG00000041845	Rhod	ras homolog family member D	-1.11
ENSMUSG00000095915	n-R5s115	nuclear encoded rRNA 5S 115	-1.11
ENSMUSG00000097615	Gm2061	predicted gene 2061	-1.11
ENSMUSG00000111539	Gm48372	predicted gene, 48372	-1.11
ENSMUSG00000027075	Slc43a1	solute carrier family 43, member 1	-1.11
ENSMUSG00000052605	Isoc2b	isochorismatase domain containing 2b	-1.11
ENSMUSG00000046733	Gprc5a	G protein-coupled receptor, family C, group 5, member A	-1.11
ENSMUSG00000087691	Gm15674	predicted gene 15674	-1.11
ENSMUSG00000116862	AC114990.2	novel transcript	-1.11
ENSMUSG00000049670	Morn4	MORN repeat containing 4	-1.11
ENSMUSG00000020486	38231	septin 4	-1.11
ENSMUSG00000081683	Fzd10	frizzled class receptor 10	-1.11
ENSMUSG00000104346	Pcdhga3	protocadherin gamma subfamily A, 3	-1.11
ENSMUSG00000037784	Dzip1l	DAZ interacting protein 1-like	-1.11
ENSMUSG00000087143	A830082K12Rik	RIKEN cDNA A830082K12 gene	-1.11
ENSMUSG00000047495	Dlgap2	DLG associated protein 2	-1.11
ENSMUSG00000081854	Gm11447	predicted gene 11447	-1.11
ENSMUSG00000106478	Gm36551	predicted gene, 36551	-1.11
ENSMUSG00000025469	Msx3	msh homeobox 3	-1.11
ENSMUSG00000112532	Gm36283	predicted gene, 36283	-1.11
ENSMUSG00000024892	Pcx	pyruvate carboxylase	-1.12
ENSMUSG00000028307	Aldob	aldolase B, fructose-bisphosphate	-1.12
ENSMUSG00000032596	Uba7	ubiquitin-like modifier activating enzyme 7	-1.12
ENSMUSG00000038963	Slco4a1	solute carrier organic anion transporter family, member 4a1	-1.12
ENSMUSG00000022090	Pdlim2	PDZ and LIM domain 2	-1.12
ENSMUSG00000024885	Aldh3b1	aldehyde dehydrogenase 3 family, member B1	-1.12
ENSMUSG00000042684	Npl	N-acetylneuraminate pyruvate lyase	-1.12
ENSMUSG00000051146	Camk2n2	calcium/calmodulin-dependent protein kinase II inhibitor 2	-1.12
ENSMUSG00000042942	Greb1l	growth regulation by estrogen in breast cancer-like	-1.12

ENSMUSG00000028076	Cd1d1	CD1d1 antigen	-1.12
ENSMUSG00000037613	Tnfrsf23	tumor necrosis factor receptor superfamily, member 23	-1.12
ENSMUSG00000034648	Lrrn1	leucine rich repeat protein 1, neuronal	-1.12
ENSMUSG00000043811	Rtn4r	reticulon 4 receptor	-1.12
ENSMUSG00000019699	Akt3	thymoma viral proto-oncogene 3	-1.12
ENSMUSG00000006542	Prkag3	protein kinase, AMP-activated, gamma 3 non-catalytic subunit	-1.12
ENSMUSG00000086507	Adap2os	ArfGAP with dual PH domains 2, opposite strand	-1.12
ENSMUSG00000111951	Gm48755	predicted gene, 48755	-1.12
ENSMUSG00000030402	Ppm1n	protein phosphatase, Mg2+/Mn2+ dependent, 1N (putative)	-1.12
ENSMUSG00000103194	Gm37643	predicted gene, 37643	-1.12
ENSMUSG00000051652	Lrrc3	leucine rich repeat containing 3	-1.12
ENSMUSG00000030651	Art2b	ADP-ribosyltransferase 2b	-1.12
ENSMUSG00000033510	Otud7a	OTU domain containing 7A	-1.12
ENSMUSG00000034377	Tulp4	tubby like protein 4	-1.13
ENSMUSG00000042207	Kdm5b	lysine (K)-specific demethylase 5B	-1.13
ENSMUSG00000027215	Cd82	CD82 antigen	-1.13
ENSMUSG00000028207	Asph	aspartate-beta-hydroxylase	-1.13
ENSMUSG00000025357	Dgka	diacylglycerol kinase, alpha	-1.13
ENSMUSG00000037280	Galnt6	polypeptide N-acetylgalactosaminyltransferase 6	-1.13
ENSMUSG00000023952	Gtpbp2	GTP binding protein 2	-1.13
ENSMUSG00000029470	P2rx4	purinergic receptor P2X, ligand-gated ion channel 4	-1.13
ENSMUSG00000021749	Oit1	oncoprotein induced transcript 1	-1.13
ENSMUSG00000028158	Mttp	microsomal triglyceride transfer protein	-1.13
ENSMUSG00000024292	Cyp4f14	cytochrome P450, family 4, subfamily f, polypeptide 14	-1.13
ENSMUSG00000048911	Rnf24	ring finger protein 24	-1.13
ENSMUSG00000031886	Ces2e	carboxylesterase 2E	-1.13
ENSMUSG00000026354	Lct	lactase	-1.13
ENSMUSG00000004552	Ctse	cathepsin E	-1.13
ENSMUSG00000038751	Ptk6	PTK6 protein tyrosine kinase 6	-1.13
ENSMUSG00000042523	Dnal1	dynein, axonemal, light chain 1	-1.13
ENSMUSG00000038843	Gcnt1	glucosaminyl (N-acetyl) transferase 1, core 2	-1.13
ENSMUSG00000033029	1700088E04Rik	RIKEN cDNA 1700088E04 gene	-1.13
ENSMUSG00000006958	Chrd	chordin	-1.13
ENSMUSG00000103897	Pcdhga8	protocadherin gamma subfamily A, 8	-1.13
ENSMUSG00000086847	Tbx3os2	T-box 3, opposite strand 2	-1.13
ENSMUSG00000102742	Pcdhga11	protocadherin gamma subfamily A, 11	-1.13
ENSMUSG00000020701	Tmem132e	transmembrane protein 132E	-1.13
ENSMUSG00000010342	Tex14	testis expressed gene 14	-1.13
ENSMUSG00000047298	Kcnv2	potassium channel, subfamily V, member 2	-1.13
ENSMUSG00000020432	Tcn2	transcobalamin 2	-1.14
ENSMUSG00000040929	Rfx3	regulatory factor X, 3 (influences HLA class II expression)	-1.14
ENSMUSG00000037995	Igsf9	immunoglobulin superfamily, member 9	-1.14
ENSMUSG00000024854	Pold4	polymerase (DNA-directed), delta 4	-1.14
ENSMUSG00000034449	Dhrs11	dehydrogenase/reductase (SDR family) member 11	-1.14
ENSMUSG00000008398	Elk3	ELK3, member of ETS oncogene family	-1.14
ENSMUSG00000056602	Fry	FRY microtubule binding protein	-1.14
ENSMUSG00000017390	Aldoc	aldolase C, fructose-bisphosphate	-1.14
ENSMUSG00000029919	Hpgds	hematopoietic prostaglandin D synthase	-1.14
ENSMUSG00000071847	Apcdd1	adenomatosis polyposis coli down-regulated 1	-1.14
ENSMUSG00000037868	Egr2	early growth response 2	-1.14
ENSMUSG00000062064	Slc2a7	solute carrier family 2 (facilitated glucose transporter), member 7	-1.14
ENSMUSG00000079339	Ifit1bl1	interferon induced protein with tetratricpeptide repeats 1B like 1	-1.14
ENSMUSG00000047473	Zfp30	zinc finger protein 30	-1.14
ENSMUSG00000104612	Gm42449	predicted gene 42449	-1.14
ENSMUSG00000003992	Ssbp2	single-stranded DNA binding protein 2	-1.14
ENSMUSG00000091898	Tnnc1	troponin C, cardiac/slow skeletal	-1.14
ENSMUSG00000033498	Strc	stereocilin	-1.14
ENSMUSG00000009687	Fxyd5	FXYD domain-containing ion transport regulator 5	-1.14
ENSMUSG00000087938	Gm26049	predicted gene, 26049	-1.14
ENSMUSG00000079497	Gm13420	predicted gene 13420	-1.14
ENSMUSG00000025221	Kcnip2	Kv channel-interacting protein 2	-1.14
ENSMUSG00000021091	Serpina3n	serine (or cysteine) peptidase inhibitor, clade A, member 3N	-1.14
ENSMUSG00000113370	Gm46348	predicted gene, 46348	-1.14
ENSMUSG00000033487	Fndc3a	fibronectin type III domain containing 3A	-1.15
ENSMUSG00000039286	Fndc3b	fibronectin type III domain containing 3B	-1.15
ENSMUSG00000020376	Rnf130	ring finger protein 130	-1.15
ENSMUSG00000001225	Slc26a3	solute carrier family 26, member 3	-1.15
ENSMUSG00000058624	Gda	guanine deaminase	-1.15
ENSMUSG00000031840	Rab3a	RAB3A, member RAS oncogene family	-1.15
ENSMUSG00000048191	Muc6	mucin 6, gastric	-1.15
ENSMUSG00000074115	Saa1	serum amyloid A 1	-1.15
ENSMUSG00000103472	Pcdhga7	protocadherin gamma subfamily A, 7	-1.15
ENSMUSG00000097990	Gm19557	predicted gene, 19557	-1.15

ENSMUSG00000113918	Gm6566	predicted gene 6566	-1.15
ENSMUSG00000002769	Gnmt	glycine N-methyltransferase	-1.15
ENSMUSG00000072572	Slc39a2	solute carrier family 39 (zinc transporter), member 2	-1.15
ENSMUSG00000114302	Gm48683	predicted gene, 48683	-1.15
ENSMUSG00000026247	Ecel1	endothelin converting enzyme-like 1	-1.15
ENSMUSG00000092116	Gm10320	predicted pseudogene 10320	-1.15
ENSMUSG00000016942	Tmprss6	transmembrane serine protease 6	-1.15
ENSMUSG00000073295	Nudt11	nudix (nucleoside diphosphate linked moiety X)-type motif 11	-1.15
ENSMUSG00000022270	Retreg1	reticulophagy regulator 1	-1.16
ENSMUSG00000020246	Hcfc2	host cell factor C2	-1.16
ENSMUSG00000039782	Cpeb2	cytoplasmic polyadenylation element binding protein 2	-1.16
ENSMUSG00000024887	Asah2	N-acylsphingosine amidohydrolase 2	-1.16
ENSMUSG00000039270	Megf9	multiple EGF-like-domains 9	-1.16
ENSMUSG00000025467	Prap1	proline-rich acidic protein 1	-1.16
ENSMUSG00000039062	Anpep	alanyl (membrane) aminopeptidase	-1.16
ENSMUSG00000024731	Ms4a10	membrane-spanning 4-domains, subfamily A, member 10	-1.16
ENSMUSG00000046410	Kcnk6	potassium inwardly-rectifying channel, subfamily K, member 6	-1.16
ENSMUSG00000024140	Epas1	endothelial PAS domain protein 1	-1.16
ENSMUSG00000032515	Csrnp1	cysteine-serine-rich nuclear protein 1	-1.16
ENSMUSG00000016496	Cd274	CD274 antigen	-1.16
ENSMUSG00000027297	Ltk	leukocyte tyrosine kinase	-1.16
ENSMUSG00000028909	Ptpru	protein tyrosine phosphatase, receptor type, U	-1.16
ENSMUSG00000040856	Dlk1	delta like non-canonical Notch ligand 1	-1.16
ENSMUSG00000044726	Erich5	glutamate rich 5	-1.16
ENSMUSG00000068859	Sp9	trans-acting transcription factor 9	-1.16
ENSMUSG00000105986	Gm43065	predicted gene 43065	-1.16
ENSMUSG00000015568	Lpl	lipoprotein lipase	-1.16
ENSMUSG00000038070	Cntln	centlein, centrosomal protein	-1.16
ENSMUSG00000087675	Gm11762	predicted gene 11762	-1.16
ENSMUSG00000078439	Smim24	small integral membrane protein 24	-1.17
ENSMUSG00000043831	Lysmd4	LysM, putative peptidoglycan-binding, domain containing 4	-1.17
ENSMUSG00000015647	Lama5	laminin, alpha 5	-1.17
ENSMUSG00000025140	Pycr1	pyrroline-5-carboxylate reductase 1	-1.17
ENSMUSG00000049971	Glt1d1	glycosyltransferase 1 domain containing 1	-1.17
ENSMUSG00000068349	Gml	glycosylphosphatidylinositol anchored molecule like	-1.17
ENSMUSG00000022683	Pla2g10	phospholipase A2, group X	-1.17
ENSMUSG00000062743	Zfp677	zinc finger protein 677	-1.17
ENSMUSG00000027377	Mall	mal, T cell differentiation protein-like	-1.17
ENSMUSG00000001248	Gramd1a	GRAM domain containing 1A	-1.17
ENSMUSG00000067049	Unc93a	unc-93 homolog A	-1.17
ENSMUSG00000087242	C78197	expressed sequence C78197	-1.17
ENSMUSG00000095440	Fignl2	fidgetin-like 2	-1.17
ENSMUSG00000106417	Gm42628	predicted gene 42628	-1.17
ENSMUSG00000052469	Tcp10c	t-complex protein 10c	-1.17
ENSMUSG00000086708	Gm15577	predicted gene 15577	-1.17
ENSMUSG00000026223	Itm2c	integral membrane protein 2C	-1.18
ENSMUSG00000036151	Tm6sf2	transmembrane 6 superfamily member 2	-1.18
ENSMUSG00000030650	Tmc5	transmembrane channel-like gene family 5	-1.18
ENSMUSG00000062638	Btnl1	butyrophilin-like 1	-1.18
ENSMUSG00000026170	Cyp27a1	cytochrome P450, family 27, subfamily a, polypeptide 1	-1.18
ENSMUSG00000079445	B3gnt7	UDP-GlcNAc:betaGal beta-1,3-N-acetylglucosaminyltransferase 7	-1.18
ENSMUSG00000042743	Sgtb	small glutamine-rich tetratricopeptide repeat (TPR)-containing, beta	-1.18
ENSMUSG00000061878	Sphk1	sphingosine kinase 1	-1.18
ENSMUSG00000039339	Mfsd4b2	major facilitator superfamily domain containing 4B2	-1.18
ENSMUSG00000022857	Tmprss15	transmembrane protease, serine 15	-1.18
ENSMUSG00000056486	Chn1	chimerin 1	-1.18
ENSMUSG00000108991	Gm45807	predicted gene 45807	-1.18
ENSMUSG00000046449	Nexmif	neurite extension and migration factor	-1.18
ENSMUSG00000031133	Arhgef6	Rac/Cdc42 guanine nucleotide exchange factor (GEF) 6	-1.18
ENSMUSG00000085500	Gm16976	predicted gene, 16976	-1.18
ENSMUSG00000112548	Gm47338	predicted gene, 47338	-1.18
ENSMUSG00000097834	Gm26911	predicted gene, 26911	-1.18
ENSMUSG00000038894	Irs2	insulin receptor substrate 2	-1.19
ENSMUSG00000029119	Man2b2	mannosidase 2, alpha B2	-1.19
ENSMUSG00000042428	Mgat3	mannoside acetylglucosaminyltransferase 3	-1.19
ENSMUSG00000024340	Btnl2	butyrophilin-like 2	-1.19
ENSMUSG00000004661	Arid3b	AT rich interactive domain 3B (BRIGHT-like)	-1.19
ENSMUSG00000022043	Trim35	tripartite motif-containing 35	-1.19
ENSMUSG00000042249	Grk3	G protein-coupled receptor kinase 3	-1.19
ENSMUSG00000022900	Ildr1	immunoglobulin-like domain containing receptor 1	-1.19
ENSMUSG00000074063	Osgin1	oxidative stress induced growth inhibitor 1	-1.19
ENSMUSG00000083138	Cyp4a29	cytochrome P450, family 4, subfamily a, polypeptide 29	-1.19
ENSMUSG00000025017	Pik3ap1	phosphoinositide-3-kinase adaptor protein 1	-1.19

ENSMUSG00000026452	Syt2	synaptotagmin II	-1.19
ENSMUSG00000054417	Cyp3a44	cytochrome P450, family 3, subfamily a, polypeptide 44	-1.19
ENSMUSG00000038540	Tmc3	transmembrane channel-like gene family 3	-1.19
ENSMUSG00000036587	Fut7	fucosyltransferase 7	-1.19
ENSMUSG00000024907	Gal	galanin	-1.19
ENSMUSG00000038312	Edem2	ER degradation enhancer, mannosidase alpha-like 2	-1.20
ENSMUSG00000041132	N4bp2l1	NEDD4 binding protein 2-like 1	-1.20
ENSMUSG00000031570	Plpp5	phospholipid phosphatase 5	-1.20
ENSMUSG00000005107	Slc2a9	solute carrier family 2 (facilitated glucose transporter), member 9	-1.20
ENSMUSG00000030223	Ptpro	protein tyrosine phosphatase, receptor type, O	-1.20
ENSMUSG00000017697	Ada	adenosine deaminase	-1.20
ENSMUSG00000028214	Gem	GTP binding protein (gene overexpressed in skeletal muscle)	-1.20
ENSMUSG00000027339	Rassf2	Ras association (RalGDS/AF-6) domain family member 2	-1.20
ENSMUSG00000112700	Gm48757	predicted gene, 48757	-1.20
ENSMUSG00000111528	Gm39460	predicted gene, 39460	-1.20
ENSMUSG00000049001	Ndnf	neuron-derived neurotrophic factor	-1.20
ENSMUSG00000027559	Car3	carbonic anhydrase 3	-1.20
ENSMUSG00000111731	Gm48727	predicted gene, 48727	-1.20
ENSMUSG00000086689	Gm16876	predicted gene, 16876	-1.20
ENSMUSG00000066621	Tecpr1	tectonin beta-propeller repeat containing 1	-1.21
ENSMUSG00000019302	Atp6v0a1	ATPase, H+ transporting, lysosomal V0 subunit A1	-1.21
ENSMUSG00000039007	Cpq	carboxypeptidase Q	-1.21
ENSMUSG00000054422	Fabp1	fatty acid binding protein 1, liver	-1.21
ENSMUSG00000030865	Chp2	calcineurin-like EF hand protein 2	-1.21
ENSMUSG00000047501	Cldn4	claudin 4	-1.21
ENSMUSG00000029269	Sult1b1	sulfotransferase family 1B, member 1	-1.21
ENSMUSG00000040721	Zfhx2	zinc finger homeobox 2	-1.21
ENSMUSG00000050777	Tmem37	transmembrane protein 37	-1.21
ENSMUSG00000051606	2010001K21Rik	RIKEN cDNA 2010001K21 gene	-1.21
ENSMUSG00000074912	Gm14207	predicted gene 14207	-1.21
ENSMUSG00000026879	Gsn	gelsolin	-1.21
ENSMUSG00000046694	Tent5b	terminal nucleotidyltransferase 5B	-1.21
ENSMUSG00000024299	Adamts10	a disintegrin-like and metallopeptidase (reprolysin type) with thrombospondin type 1 motif, 10	-1.21
ENSMUSG00000021306	Gpr137b	G protein-coupled receptor 137B	-1.21
ENSMUSG00000048562	Sp8	trans-acting transcription factor 8	-1.21
ENSMUSG00000037747	Phyhipl	phytanoyl-CoA hydroxylase interacting protein-like	-1.21
ENSMUSG00000006395	Hyi	hydroxypyruvate isomerase (putative)	-1.22
ENSMUSG00000051910	Sox6	SRY (sex determining region Y)-box 6	-1.22
ENSMUSG00000059316	Slc27a4	solute carrier family 27 (fatty acid transporter), member 4	-1.22
ENSMUSG00000023959	Clic5	chloride intracellular channel 5	-1.22
ENSMUSG00000018919	Tm4sf5	transmembrane 4 superfamily member 5	-1.22
ENSMUSG00000096215	Smim22	small integral membrane protein 22	-1.22
ENSMUSG00000047641	Krt87	keratin 87	-1.22
ENSMUSG00000059540	Tcea2	transcription elongation factor A (SII), 2	-1.22
ENSMUSG00000049556	Lingo1	leucine rich repeat and Ig domain containing 1	-1.22
ENSMUSG00000112276	5033421B08Rik	RIKEN cDNA 5033421B08 gene	-1.22
ENSMUSG00000045027	Prss22	protease, serine 22	-1.22
ENSMUSG00000052125	F730043M19Rik	RIKEN cDNA F730043M19 gene	-1.22
ENSMUSG00000106871	Gm3289	predicted gene 3289	-1.22
ENSMUSG00000068699	Flnc	filamin C, gamma	-1.22
ENSMUSG00000052188	Gm14964	predicted gene 14964	-1.22
ENSMUSG00000048249	Crebrf	CREB3 regulatory factor	-1.23
ENSMUSG00000031618	Nr3c2	nuclear receptor subfamily 3, group C, member 2	-1.23
ENSMUSG00000024131	Slc3a1	solute carrier family 3, member 1	-1.23
ENSMUSG00000034858	Fam214a	family with sequence similarity 214, member A	-1.23
ENSMUSG00000023495	Pcbp4	poly(rC) binding protein 4	-1.23
ENSMUSG00000060429	Sntb1	syntrophin, basic 1	-1.23
ENSMUSG00000078612	Fyb2	FYN binding protein 2	-1.23
ENSMUSG00000022219	Cideb	cell death-inducing DNA fragmentation factor, alpha subunit-like effector B	-1.23
ENSMUSG00000041540	Sox5	SRY (sex determining region Y)-box 5	-1.23
ENSMUSG00000001249	Hpn	hepsin	-1.23
ENSMUSG00000027438	Napb	N-ethylmaleimide sensitive fusion protein attachment protein beta	-1.23
ENSMUSG00000115062	4930544F09Rik	RIKEN cDNA 4930544F09 gene	-1.23
ENSMUSG00000074896	Ifit3	interferon-induced protein with tetratricopeptide repeats 3	-1.23
ENSMUSG00000091017	Fam71a	family with sequence similarity 71, member A	-1.23
ENSMUSG00000105985	Gm42993	predicted gene 42993	-1.23
ENSMUSG00000102620	Gm37675	predicted gene, 37675	-1.23
ENSMUSG00000038608	Dock10	dedicator of cytokinesis 10	-1.23
ENSMUSG00000089861	Gm16139	predicted gene 16139	-1.23
ENSMUSG00000071356	Reg3b	regenerating islet-derived 3 beta	-1.24
ENSMUSG00000020829	Slc46a1	solute carrier family 46, member 1	-1.24
ENSMUSG00000034947	Tmem106a	transmembrane protein 106A	-1.24
ENSMUSG00000001095	Slc13a2	solute carrier family 13 (sodium-dependent dicarboxylate transporter), member 2	-1.24

ENSMUSG00000097277	2900076A07Rik	RIKEN cDNA 2900076A07 gene	-1.24
ENSMUSG00000033460	Armcx1	armadillo repeat containing, X-linked 1	-1.24
ENSMUSG00000034450	Gulo	gulonolactone (L-) oxidase	-1.24
ENSMUSG00000103693	Gm37529	predicted gene, 37529	-1.24
ENSMUSG00000097601	Gm26660	predicted gene, 26660	-1.24
ENSMUSG00000038777	Sema6c	sema domain, transmembrane domain (TM), and cytoplasmic domain, (semaphorin) 6C	-1.24
ENSMUSG00000033276	Stk36	serine/threonine kinase 36	-1.24
ENSMUSG00000035551	Igfbpl1	insulin-like growth factor binding protein-like 1	-1.24
ENSMUSG00000033227	Wnt6	wingless-type MMTV integration site family, member 6	-1.24
ENSMUSG00000084137	Gm9085	predicted gene 9085	-1.24
ENSMUSG00000041696	Rasl12	RAS-like, family 12	-1.24
ENSMUSG00000057060	Slc35f3	solute carrier family 35, member F3	-1.24
ENSMUSG00000085085	1700086P04Rik	RIKEN cDNA 1700086P04 gene	-1.24
ENSMUSG00000105302	Gm19817	predicted gene, 19817	-1.24
ENSMUSG00000040296	Ddx58	DEAD (Asp-Glu-Ala-Asp) box polypeptide 58	-1.25
ENSMUSG00000038244	Mical2	microtubule associated monooxygenase, calponin and LIM domain containing 2	-1.25
ENSMUSG00000091705	H2-Q2	histocompatibility 2, Q region locus 2	-1.25
ENSMUSG00000032120	C2cd2l	C2 calcium-dependent domain containing 2-like	-1.25
ENSMUSG00000079507	H2-Q1	histocompatibility 2, Q region locus 1	-1.25
ENSMUSG00000040327	Cul9	cullin 9	-1.25
ENSMUSG00000039943	Plcb4	phospholipase C, beta 4	-1.25
ENSMUSG00000042675	Ypel3	yippee like 3	-1.25
ENSMUSG00000032593	Amigo3	adhesion molecule with Ig like domain 3	-1.25
ENSMUSG00000068923	Syt11	synaptotagmin XI	-1.25
ENSMUSG00000058063	Trim31	tripartite motif-containing 31	-1.25
ENSMUSG00000047617	Paxx	non-homologous end joining factor	-1.25
ENSMUSG00000057706	Mex3b	mex3 RNA binding family member B	-1.25
ENSMUSG00000021061	Sptb	spectrin beta, erythrocytic	-1.25
ENSMUSG00000024899	Papss2	3'-phosphoadenosine 5'-phosphosulfate synthase 2	-1.25
ENSMUSG00000040483	Xaf1	XIAP associated factor 1	-1.25
ENSMUSG00000110276	Gm45330	predicted gene 45330	-1.25
ENSMUSG00000031841	Cdh13	cadherin 13	-1.25
ENSMUSG00000038354	Ankrd35	ankyrin repeat domain 35	-1.25
ENSMUSG00000104020	Gm37215	predicted gene, 37215	-1.25
ENSMUSG00000021414	Fam217a	family with sequence similarity 217, member A	-1.25
ENSMUSG00000031647	Mfap3l	microfibrillar-associated protein 3-like	-1.25
ENSMUSG00000115360	Gm48932	predicted gene, 48932	-1.25
ENSMUSG00000054277	Arfgap3	ADP-ribosylation factor GTPase activating protein 3	-1.26
ENSMUSG00000075520	Malrd1	MAM and LDL receptor class A domain containing 1	-1.26
ENSMUSG00000035506	Slc12a8	solute carrier family 12 (potassium/chloride transporters), member 8	-1.26
ENSMUSG00000062995	Ica1	islet cell autoantigen 1	-1.26
ENSMUSG00000031613	Hpgd	hydroxyprostaglandin dehydrogenase 15 (NAD)	-1.26
ENSMUSG00000042041	2010003K11Rik	RIKEN cDNA 2010003K11 gene	-1.26
ENSMUSG00000085333	1700030A11Rik	RIKEN cDNA 1700030A11 gene	-1.26
ENSMUSG00000040990	Sh3kbp1	SH3-domain kinase binding protein 1	-1.26
ENSMUSG00000031155	Pim2	proviral integration site 2	-1.26
ENSMUSG00000089817	Gm7162	predicted gene 7162	-1.26
ENSMUSG00000103749	Pcdhgb5	protocadherin gamma subfamily B, 5	-1.26
ENSMUSG00000096995	2810029C07Rik	RIKEN cDNA 2810029C07 gene	-1.26
ENSMUSG00000000126	Wnt9a	wingless-type MMTV integration site family, member 9A	-1.26
ENSMUSG00000038623	Tm6sf1	transmembrane 6 superfamily member 1	-1.26
ENSMUSG00000025207	Sema4g	sema domain, immunoglobulin domain (Ig), transmembrane domain (TM) and short cytoplasmic dor	-1.27
ENSMUSG00000032776	Mctp2	multiple C2 domains, transmembrane 2	-1.27
ENSMUSG00000015405	Ace2	angiotensin I converting enzyme (peptidyl-dipeptidase A) 2	-1.27
ENSMUSG00000024254	Abcg8	ATP binding cassette subfamily G member 8	-1.27
ENSMUSG00000039899	Fgl2	fibrinogen-like protein 2	-1.27
ENSMUSG00000071862	Lrrtm2	leucine rich repeat transmembrane neuronal 2	-1.27
ENSMUSG00000037818	Abhd18	abhydrolase domain containing 18	-1.27
ENSMUSG00000054999	Naaladl1	N-acetylated alpha-linked acidic dipeptidase-like 1	-1.27
ENSMUSG00000068341	Reg3d	regenerating islet-derived 3 delta	-1.27
ENSMUSG00000102428	Pcdhga12	protocadherin gamma subfamily A, 12	-1.27
ENSMUSG00000040350	Trim7	tripartite motif-containing 7	-1.27
ENSMUSG00000074782	4833422C13Rik	RIKEN cDNA 4833422C13 gene	-1.27
ENSMUSG00000033177	Tmprss7	transmembrane serine protease 7	-1.27
ENSMUSG00000049811	Fam161a	family with sequence similarity 161, member A	-1.27
ENSMUSG00000024682	Gif	gastric intrinsic factor	-1.27
ENSMUSG00000107619	Gm32479	predicted gene, 32479	-1.27
ENSMUSG00000069378	Prdm6	PR domain containing 6	-1.27
ENSMUSG00000025129	Ppp1r27	protein phosphatase 1, regulatory subunit 27	-1.27
ENSMUSG00000047307	Pcdhb13	protocadherin beta 13	-1.27
ENSMUSG00000014905	Dnajb9	DnaJ heat shock protein family (Hsp40) member B9	-1.28
ENSMUSG00000023067	Cdkn1a	cyclin-dependent kinase inhibitor 1A (P21)	-1.28
ENSMUSG00000059991	Nptx2	neuronal pentraxin 2	-1.28

ENSMUSG00000028015	Ctso	cathepsin O	-1.28
ENSMUSG00000024713	Pcsk5	proprotein convertase subtilisin/kexin type 5	-1.28
ENSMUSG00000020261	Slc36a1	solute carrier family 36 (proton/amino acid symporter), member 1	-1.28
ENSMUSG00000029188	Slc34a2	solute carrier family 34 (sodium phosphate), member 2	-1.28
ENSMUSG00000021125	Arg2	arginase type II	-1.28
ENSMUSG00000018740	Slc25a35	solute carrier family 25, member 35	-1.28
ENSMUSG00000002603	Tgfb1	transforming growth factor, beta 1	-1.28
ENSMUSG00000073403	Gm10499	predicted gene 10499	-1.28
ENSMUSG00000031637	Lrp2bp	Lrp2 binding protein	-1.28
ENSMUSG00000031298	Adgrg2	adhesion G protein-coupled receptor G2	-1.28
ENSMUSG00000035964	Tmem59l	transmembrane protein 59-like	-1.28
ENSMUSG00000028435	Aqp3	aquaporin 3	-1.28
ENSMUSG00000070576	Mn1	meningioma 1	-1.28
ENSMUSG00000086701	Gm13595	predicted gene 13595	-1.28
ENSMUSG00000020609	Apob	apolipoprotein B	-1.29
ENSMUSG00000068742	Cry2	cryptochrome 2 (photolyase-like)	-1.29
ENSMUSG00000049307	Fut4	fucosyltransferase 4	-1.29
ENSMUSG00000021062	Rab15	RAB15, member RAS oncogene family	-1.29
ENSMUSG00000068735	Trp53i11	transformation related protein 53 inducible protein 11	-1.29
ENSMUSG00000039579	Grin3a	glutamate receptor ionotropic, NMDA3A	-1.29
ENSMUSG00000037493	Cib2	calcium and integrin binding family member 2	-1.29
ENSMUSG00000042564	Fam227a	family with sequence similarity 227, member A	-1.29
ENSMUSG00000002997	Prkar2b	protein kinase, cAMP dependent regulatory, type II beta	-1.29
ENSMUSG00000041912	Tdrkh	tudor and KH domain containing protein	-1.29
ENSMUSG00000047669	Msl3l2	MSL3 like 2	-1.29
ENSMUSG00000022041	Chrna2	cholinergic receptor, nicotinic, alpha polypeptide 2 (neuronal)	-1.29
ENSMUSG00000074121	Ntf5	neurotrophin 5	-1.29
ENSMUSG00000020169	Best3	bestrophin 3	-1.29
ENSMUSG00000070436	Serpinh1	serine (or cysteine) peptidase inhibitor, clade H, member 1	-1.29
ENSMUSG00000112805	Gm34574	predicted gene, 34574	-1.29
ENSMUSG00000005125	Ndrg1	N-myc downstream regulated gene 1	-1.30
ENSMUSG00000029370	Rassf6	Ras association (RalGDS/AF-6) domain family member 6	-1.30
ENSMUSG00000047728	BC025446	cDNA sequence BC025446	-1.30
ENSMUSG00000032192	Gnb5	guanine nucleotide binding protein (G protein), beta 5	-1.30
ENSMUSG00000067297	Ifit1bl2	interferon induced protein with tetratricopeptide repeats 1B like 2	-1.30
ENSMUSG00000021411	Pxdc1	PX domain containing 1	-1.30
ENSMUSG00000052364	B630019K06Rik	RIKEN cDNA B630019K06 gene	-1.30
ENSMUSG00000032053	Pou2af1	POU domain, class 2, associating factor 1	-1.30
ENSMUSG00000087623	Gm12404	predicted gene 12404	-1.30
ENSMUSG00000109136	Gm45114	predicted gene 45114	-1.30
ENSMUSG00000085723	Gm15915	predicted gene 15915	-1.30
ENSMUSG00000022040	Ephx2	epoxide hydrolase 2, cytoplasmic	-1.31
ENSMUSG00000052302	Tbc1d30	TBC1 domain family, member 30	-1.31
ENSMUSG00000029561	Oasl2	2'-5' oligoadenylate synthetase-like 2	-1.31
ENSMUSG00000022377	Asap1	ArfGAP with SH3 domain, ankyrin repeat and PH domain1	-1.31
ENSMUSG00000102758	Naaladl2	N-acetylated alpha-linked acidic dipeptidase-like 2	-1.31
ENSMUSG00000070315	4930581F22Rik	RIKEN cDNA 4930581F22 gene	-1.31
ENSMUSG00000074261	Erich4	glutamate rich 4	-1.31
ENSMUSG00000075270	Pde11a	phosphodiesterase 11A	-1.31
ENSMUSG00000021759	Plpp1	phospholipid phosphatase 1	-1.31
ENSMUSG00000027805	Pfn2	profilin 2	-1.31
ENSMUSG00000084803	5830444B04Rik	RIKEN cDNA 5830444B04 gene	-1.31
ENSMUSG00000027225	Duoxa2	dual oxidase maturation factor 2	-1.31
ENSMUSG00000029925	Tbxas1	thromboxane A synthase 1, platelet	-1.31
ENSMUSG00000021268	Meg3	maternally expressed 3	-1.31
ENSMUSG00000054477	Kcnn2	potassium intermediate/small conductance calcium-activated channel, subfamily N, member 2	-1.31
ENSMUSG00000062859	Tcp11	t-complex protein 11	-1.31
ENSMUSG00000087341	0610040F04Rik	RIKEN cDNA 0610040F04 gene	-1.31
ENSMUSG00000106709	Gm30270	predicted gene, 30270	-1.31
ENSMUSG00000019906	Lin7a	lin-7 homolog A (C. elegans)	-1.31
ENSMUSG00000110802	Gm47141	predicted gene, 47141	-1.31
ENSMUSG00000062991	Nrg1	neuregulin 1	-1.31
ENSMUSG00000027820	Mme	membrane metallo endopeptidase	-1.32
ENSMUSG00000038473	Nos1ap	nitric oxide synthase 1 (neuronal) adaptor protein	-1.32
ENSMUSG00000053334	Ficd	FIC domain containing	-1.32
ENSMUSG00000039911	Spsb1	splA/ryanodine receptor domain and SOCS box containing 1	-1.32
ENSMUSG00000028389	Zfp37	zinc finger protein 37	-1.32
ENSMUSG00000030340	Scnn1a	sodium channel, nonvoltage-gated 1 alpha	-1.32
ENSMUSG00000020034	Tcp11l2	t-complex 11 (mouse) like 2	-1.32
ENSMUSG00000029279	Brdt	bromodomain, testis-specific	-1.32
ENSMUSG00000000805	Car4	carbonic anhydrase 4	-1.32
ENSMUSG00000033191	Tie1	tyrosine kinase with immunoglobulin-like and EGF-like domains 1	-1.32
ENSMUSG00000044250	Pced1b	PC-esterase domain containing 1B	-1.32

ENSMUSG00000079157	Fam155a	family with sequence similarity 155, member A	-1.32
ENSMUSG00000046447	Camk2n1	calcium/calmodulin-dependent protein kinase II inhibitor 1	-1.33
ENSMUSG00000041609	Bicdl1	BICD family like cargo adaptor 1	-1.33
ENSMUSG00000090214	Gm15657	predicted gene 15657	-1.33
ENSMUSG00000024730	Ms4a8a	membrane-spanning 4-domains, subfamily A, member 8A	-1.33
ENSMUSG00000028150	Rorc	RAR-related orphan receptor gamma	-1.33
ENSMUSG00000026614	Slc30a10	solute carrier family 30, member 10	-1.33
ENSMUSG00000028860	Sytl1	synaptotagmin-like 1	-1.33
ENSMUSG00000074207	Adh1	alcohol dehydrogenase 1 (class I)	-1.33
ENSMUSG00000085091	Egfros	epidermal growth factor receptor, opposite strand	-1.33
ENSMUSG00000024503	Spink1	serine peptidase inhibitor, Kazal type 1	-1.33
ENSMUSG00000032238	Rora	RAR-related orphan receptor alpha	-1.33
ENSMUSG00000110206	Flt3l	FMS-like tyrosine kinase 3 ligand	-1.33
ENSMUSG00000089028	Gm25010	predicted gene, 25010	-1.33
ENSMUSG00000081968	Rpl23a-ps2	ribosomal protein L23A, pseudogene 2	-1.33
ENSMUSG00000042066	Tmcc2	transmembrane and coiled-coil domains 2	-1.33
ENSMUSG00000098843	Gm27240	predicted gene 27240	-1.33
ENSMUSG00000022512	Cldn1	claudin 1	-1.33
ENSMUSG00000038150	Ormdl3	ORM1-like 3 (S. cerevisiae)	-1.34
ENSMUSG00000036452	Arhgap26	Rho GTPase activating protein 26	-1.34
ENSMUSG00000061825	Ces2c	carboxylesterase 2C	-1.34
ENSMUSG00000032038	St3gal4	ST3 beta-galactoside alpha-2,3-sialyltransferase 4	-1.34
ENSMUSG00000040998	Npnt	nephronectin	-1.34
ENSMUSG00000034919	Ttc22	tetratricopeptide repeat domain 22	-1.34
ENSMUSG00000037126	Psd	pleckstrin and Sec7 domain containing	-1.34
ENSMUSG00000032081	Apoc3	apolipoprotein C-III	-1.34
ENSMUSG00000072620	Slfn2	schlafen 2	-1.34
ENSMUSG00000064262	Gimap8	GTPase, IMAP family member 8	-1.34
ENSMUSG00000092323	BB365896	expressed sequence BB365896	-1.34
ENSMUSG00000067261	Foxd3	forkhead box D3	-1.34
ENSMUSG00000097983	Gm26971	predicted gene, 26971	-1.34
ENSMUSG00000056296	Synpr	synaptoporin	-1.34
ENSMUSG00000022416	Cacna1i	calcium channel, voltage-dependent, alpha 1I subunit	-1.34
ENSMUSG00000019823	Mical1	microtubule associated monooxygenase, calponin and LIM domain containing 1	-1.35
ENSMUSG00000059713	Rcan3	regulator of calcineurin 3	-1.35
ENSMUSG00000020600	Slc7a15	solute carrier family 7 (cationic amino acid transporter, y+ system), member 15	-1.35
ENSMUSG00000024313	Mep1b	meprin 1 beta	-1.35
ENSMUSG00000043029	Trpv3	transient receptor potential cation channel, subfamily V, member 3	-1.35
ENSMUSG00000040147	Maob	monoamine oxidase B	-1.35
ENSMUSG00000024386	Proc	protein C	-1.35
ENSMUSG00000113864	Gm48381	predicted gene, 48381	-1.35
ENSMUSG00000112198	Gm4065	predicted gene 4065	-1.35
ENSMUSG00000072966	Gprasp2	G protein-coupled receptor associated sorting protein 2	-1.35
ENSMUSG00000026938	Fcna	ficolin A	-1.35
ENSMUSG00000035722	Abca7	ATP-binding cassette, sub-family A (ABC1), member 7	-1.36
ENSMUSG00000056035	Cyp3a11	cytochrome P450, family 3, subfamily a, polypeptide 11	-1.36
ENSMUSG00000039770	Ypel5	yippee like 5	-1.36
ENSMUSG00000052105	Mtcl1	microtubule crosslinking factor 1	-1.36
ENSMUSG00000041361	Myzap	myocardial zonula adherens protein	-1.36
ENSMUSG00000019989	Enpp3	ectonucleotide pyrophosphatase/phosphodiesterase 3	-1.36
ENSMUSG00000079440	Alpi	alkaline phosphatase, intestinal	-1.36
ENSMUSG00000028051	Hcn3	hyperpolarization-activated, cyclic nucleotide-gated K+ 3	-1.36
ENSMUSG00000039384	Dusp10	dual specificity phosphatase 10	-1.36
ENSMUSG00000022661	Cd200	CD200 antigen	-1.36
ENSMUSG00000054150	Syne3	spectrin repeat containing, nuclear envelope family member 3	-1.36
ENSMUSG00000089857	Zfp882	zinc finger protein 882	-1.36
ENSMUSG00000019831	Wasf1	WAS protein family, member 1	-1.36
ENSMUSG00000111293	Gm34006	predicted gene, 34006	-1.36
ENSMUSG00000034774	Dsg1c	desmoglein 1 gamma	-1.36
ENSMUSG00000056091	St3gal5	ST3 beta-galactoside alpha-2,3-sialyltransferase 5	-1.36
ENSMUSG00000005640	Insrr	insulin receptor-related receptor	-1.36
ENSMUSG00000037235	Mxd4	Max dimerization protein 4	-1.37
ENSMUSG00000020300	Cpeb4	cytoplasmic polyadenylation element binding protein 4	-1.37
ENSMUSG00000030017	Reg3g	regenerating islet-derived 3 gamma	-1.37
ENSMUSG00000024193	Phf1	PHD finger protein 1	-1.37
ENSMUSG00000028976	Slc2a5	solute carrier family 2 (facilitated glucose transporter), member 5	-1.37
ENSMUSG00000019278	Dpep1	dipeptidase 1 (renal)	-1.37
ENSMUSG00000029082	Bst1	bone marrow stromal cell antigen 1	-1.37
ENSMUSG00000103579	Gm37113	predicted gene, 37113	-1.37
ENSMUSG00000110754	4930405J17Rik	RIKEN cDNA 4930405J17 gene	-1.37
ENSMUSG00000019762	Iyd	iodotyrosine deiodinase	-1.37
ENSMUSG00000059013	Sh2d3c	SH2 domain containing 3C	-1.37
ENSMUSG00000026586	Prrx1	paired related homeobox 1	-1.37

ENSMUSG00000035158	Mitf	melanogenesis associated transcription factor	-1.37
ENSMUSG00000044912	Syt16	synaptotagmin XVI	-1.37
ENSMUSG00000109093	Gm19950	predicted gene, 19950	-1.37
ENSMUSG00000022946	Dopey2	dopey family member 2	-1.38
ENSMUSG00000092618	Btnl6	butyrophilin-like 6	-1.38
ENSMUSG00000040505	Abcg5	ATP binding cassette subfamily G member 5	-1.38
ENSMUSG00000021751	Acox2	acyl-Coenzyme A oxidase 2, branched chain	-1.38
ENSMUSG00000007989	Fzd3	frizzled class receptor 3	-1.38
ENSMUSG00000057596	Trim30d	tripartite motif-containing 30D	-1.38
ENSMUSG00000019647	Sema6a	sema domain, transmembrane domain (TM), and cytoplasmic domain, (semaphorin) 6A	-1.38
ENSMUSG00000027358	Bmp2	bone morphogenetic protein 2	-1.38
ENSMUSG00000037738	Nek5	NIMA (never in mitosis gene a)-related expressed kinase 5	-1.38
ENSMUSG00000020604	Arsg	arylsulfatase G	-1.38
ENSMUSG00000097443	Gm17529	predicted gene, 17529	-1.38
ENSMUSG00000060244	Alyref2	Aly/REF export factor 2	-1.38
ENSMUSG00000000386	Mx1	MX dynamin-like GTPase 1	-1.38
ENSMUSG00000111425	Gm47324	predicted gene, 47324	-1.38
ENSMUSG00000043313	Pcdhb19	protocadherin beta 19	-1.38
ENSMUSG00000095066	Defa20	defensin, alpha, 20	-1.38
ENSMUSG00000026303	Mlph	melanophilin	-1.39
ENSMUSG00000028088	Fmo5	flavin containing monooxygenase 5	-1.39
ENSMUSG00000020447	Npc1l1	NPC1 like intracellular cholesterol transporter 1	-1.39
ENSMUSG00000018427	Ypel2	yippee like 2	-1.39
ENSMUSG00000048612	Myof	myoferlin	-1.39
ENSMUSG00000048232	Fbxo10	F-box protein 10	-1.39
ENSMUSG00000047910	Pcdhb16	protocadherin beta 16	-1.39
ENSMUSG00000025348	Itga7	integrin alpha 7	-1.39
ENSMUSG00000062017	Abca14	ATP-binding cassette, sub-family A (ABC1), member 14	-1.39
ENSMUSG00000086231	Rapgef4os3	Rap guanine nucleotide exchange factor (GEF) 4, opposite strand 3	-1.39
ENSMUSG00000042567	Nek10	NIMA (never in mitosis gene a)- related kinase 10	-1.39
ENSMUSG00000038700	Hoxb5	homeobox B5	-1.39
ENSMUSG00000026509	Capn2	calpain 2	-1.40
ENSMUSG00000024190	Dusp1	dual specificity phosphatase 1	-1.40
ENSMUSG00000021453	Gadd45g	growth arrest and DNA-damage-inducible 45 gamma	-1.40
ENSMUSG00000031303	Map3k15	mitogen-activated protein kinase kinase kinase 15	-1.40
ENSMUSG00000002944	Cd36	CD36 molecule	-1.40
ENSMUSG00000037049	Smpd1	sphingomyelin phosphodiesterase 1, acid lysosomal	-1.40
ENSMUSG00000045838	Ccdc9b	coiled-coil domain containing 9B	-1.40
ENSMUSG00000068452	Duox2	dual oxidase 2	-1.40
ENSMUSG00000034685	Fam171a2	family with sequence similarity 171, member A2	-1.40
ENSMUSG00000000253	Gmpr	guanosine monophosphate reductase	-1.40
ENSMUSG00000034634	Ly6d	lymphocyte antigen 6 complex, locus D	-1.40
ENSMUSG00000015745	Plekho1	pleckstrin homology domain containing, family O member 1	-1.40
ENSMUSG00000024421	Lama3	laminin, alpha 3	-1.40
ENSMUSG00000033847	Pla2g4c	phospholipase A2, group IVC (cytosolic, calcium-independent)	-1.40
ENSMUSG00000074217	2210011C24Rik	RIKEN cDNA 2210011C24 gene	-1.40
ENSMUSG00000010066	Cacna2d2	calcium channel, voltage-dependent, alpha 2/delta subunit 2	-1.40
ENSMUSG00000056162	Cndp1	carnosine dipeptidase 1 (metallopeptidase M20 family)	-1.40
ENSMUSG00000027314	Dll4	delta like canonical Notch ligand 4	-1.41
ENSMUSG00000019970	Sgk1	serum/glucocorticoid regulated kinase 1	-1.41
ENSMUSG00000066258	Trim12a	tripartite motif-containing 12A	-1.41
ENSMUSG00000032289	Thsd4	thrombospondin, type I, domain containing 4	-1.41
ENSMUSG00000022885	St6gal1	beta galactoside alpha 2,6 sialyltransferase 1	-1.41
ENSMUSG00000087331	1810021B22Rik	RIKEN cDNA 1810021B22 gene	-1.41
ENSMUSG00000005986	Ankrd13d	ankyrin repeat domain 13 family, member D	-1.41
ENSMUSG00000074657	Kif5a	kinesin family member 5A	-1.41
ENSMUSG00000030897	Cnga4	cyclic nucleotide gated channel alpha 4	-1.41
ENSMUSG00000020423	Btg2	B cell translocation gene 2, anti-proliferative	-1.42
ENSMUSG00000025498	Irf7	interferon regulatory factor 7	-1.42
ENSMUSG00000020715	Ern1	endoplasmic reticulum (ER) to nucleus signalling 1	-1.42
ENSMUSG00000034918	Cdhr2	cadherin-related family member 2	-1.42
ENSMUSG00000030155	Clec2e	C-type lectin domain family 2, member e	-1.42
ENSMUSG00000022048	Dpysl2	dihydropyrimidinase-like 2	-1.42
ENSMUSG00000036989	Trim3	tripartite motif-containing 3	-1.42
ENSMUSG00000065145	Vaultrc5	vault RNA component 5	-1.42
ENSMUSG00000025035	Arl3	ADP-ribosylation factor-like 3	-1.42
ENSMUSG00000051452	Gm11437	predicted gene 11437	-1.42
ENSMUSG00000048895	Cdk5r1	cyclin-dependent kinase 5, regulatory subunit 1 (p35)	-1.42
ENSMUSG00000027931	Npr1	natriuretic peptide receptor 1	-1.42
ENSMUSG00000100593	1700119H24Rik	RIKEN cDNA 1700119H24 gene	-1.42
ENSMUSG00000096054	Syne1	spectrin repeat containing, nuclear envelope 1	-1.42
ENSMUSG00000097572	Gm26797	predicted gene, 26797	-1.42
ENSMUSG00000103738	Gm37652	predicted gene, 37652	-1.42

ENSMUSG00000100747	1700084E18Rik	RIKEN cDNA 1700084E18 gene	-1.42
ENSMUSG00000081245	Gm6587	predicted gene 6587	-1.42
ENSMUSG00000021850	ccdc198	coiled-coil domain containing 198	-1.42
ENSMUSG00000020836	Coro6	coronin 6	-1.42
ENSMUSG00000032892	Rangrf	RAN guanine nucleotide release factor	-1.42
ENSMUSG00000029992	Gfpt1	glutamine fructose-6-phosphate transaminase 1	-1.43
ENSMUSG00000052085	Dock8	dedicator of cytokinesis 8	-1.43
ENSMUSG00000034981	Parm1	prostate androgen-regulated mucin-like protein 1	-1.43
ENSMUSG00000020709	Adap2	ArfGAP with dual PH domains 2	-1.43
ENSMUSG00000061808	Ttr	transthyretin	-1.43
ENSMUSG00000022686	B3gnt5	UDP-GlcNAc:betaGal beta-1,3-N-acetylglucosaminyltransferase 5	-1.43
ENSMUSG00000079499	6530402F18Rik	RIKEN cDNA 6530402F18 gene	-1.43
ENSMUSG00000042109	Csdc2	cold shock domain containing C2, RNA binding	-1.43
ENSMUSG00000105720	Gm42440	predicted gene 42440	-1.43
ENSMUSG00000078942	Naip6	NLR family, apoptosis inhibitory protein 6	-1.44
ENSMUSG00000020021	Fgd6	FYVE, RhoGEF and PH domain containing 6	-1.44
ENSMUSG00000028024	Enpep	glutamyl aminopeptidase	-1.44
ENSMUSG00000041827	Oasl1	2'-5' oligoadenylate synthetase-like 1	-1.44
ENSMUSG00000085148	Mir22hg	Mir22 host gene (non-protein coding)	-1.44
ENSMUSG00000040389	Wdr47	WD repeat domain 47	-1.44
ENSMUSG00000054793	Cadm4	cell adhesion molecule 4	-1.44
ENSMUSG00000116940	CT033750.3	antizyme inhibitor 2 (Azin2) pseudogene	-1.44
ENSMUSG00000097081	Gm10425	predicted gene 10425	-1.44
ENSMUSG00000011256	Adam19	a disintegrin and metallopeptidase domain 19 (meltrin beta)	-1.44
ENSMUSG00000103726	Gm30074	predicted gene, 30074	-1.44
ENSMUSG00000037822	Smim14	small integral membrane protein 14	-1.45
ENSMUSG00000041119	Pde9a	phosphodiesterase 9A	-1.45
ENSMUSG00000053054	Adh6a	alcohol dehydrogenase 6A (class V)	-1.45
ENSMUSG00000027219	Slc28a2	solute carrier family 28 (sodium-coupled nucleoside transporter), member 2	-1.45
ENSMUSG00000019066	Rab3d	RAB3D, member RAS oncogene family	-1.45
ENSMUSG00000044734	Serpinb1a	serine (or cysteine) peptidase inhibitor, clade B, member 1a	-1.45
ENSMUSG00000055730	Ces2a	carboxylesterase 2A	-1.45
ENSMUSG00000041695	Kcnj2	potassium inwardly-rectifying channel, subfamily J, member 2	-1.45
ENSMUSG00000030643	Rab30	RAB30, member RAS oncogene family	-1.45
ENSMUSG00000027950	Chrnb2	cholinergic receptor, nicotinic, beta polypeptide 2 (neuronal)	-1.45
ENSMUSG00000024846	Cst6	cystatin E/M	-1.45
ENSMUSG00000020900	Myh10	myosin, heavy polypeptide 10, non-muscle	-1.45
ENSMUSG00000024039	Cbs	cystathionine beta-synthase	-1.45
ENSMUSG00000097147	Gm26780	predicted gene, 26780	-1.45
ENSMUSG00000022091	Sorbs3	sorbin and SH3 domain containing 3	-1.45
ENSMUSG00000097391	Mirg	miRNA containing gene	-1.45
ENSMUSG00000014158	Trpv4	transient receptor potential cation channel, subfamily V, member 4	-1.45
ENSMUSG00000040428	Plekha4	pleckstrin homology domain containing, family A (phosphoinositide binding specific) member 4	-1.45
ENSMUSG00000107583	Gm44104	predicted gene, 44104	-1.45
ENSMUSG00000056418	BC043934	cDNA sequence BC043934	-1.45
ENSMUSG00000102882	Gm2065	predicted gene 2065	-1.45
ENSMUSG00000038178	Slc43a2	solute carrier family 43, member 2	-1.46
ENSMUSG00000048905	4930539E08Rik	RIKEN cDNA 4930539E08 gene	-1.46
ENSMUSG00000022197	Pdzd2	PDZ domain containing 2	-1.46
ENSMUSG00000110397	Gm45540	predicted gene 45540	-1.46
ENSMUSG00000061740	Cyp2d22	cytochrome P450, family 2, subfamily d, polypeptide 22	-1.46
ENSMUSG00000105374	Gm42551	predicted gene 42551	-1.46
ENSMUSG00000029838	Ptn	pleiotrophin	-1.46
ENSMUSG00000094840	Muc3a	mucin 3A, cell surface associated	-1.47
ENSMUSG00000036390	Gadd45a	growth arrest and DNA-damage-inducible 45 alpha	-1.47
ENSMUSG00000039217	Il18	interleukin 18	-1.47
ENSMUSG00000032652	Crebl2	cAMP responsive element binding protein-like 2	-1.47
ENSMUSG00000058921	Slc10a5	solute carrier family 10 (sodium/bile acid cotransporter family), member 5	-1.47
ENSMUSG00000048473	Sult6b2	sulfotransferase family 6B, member 2	-1.47
ENSMUSG00000055148	Klf2	Kruppel-like factor 2 (lung)	-1.47
ENSMUSG00000074622	Mafb	v-maf musculoaponeurotic fibrosarcoma oncogene family, protein B (avian)	-1.47
ENSMUSG00000110887	Cbx3-ps8	chromobox 3, pseudogene 8	-1.47
ENSMUSG00000057933	Gsta2	glutathione S-transferase, alpha 2 (Yc2)	-1.47
ENSMUSG00000023393	Slc17a9	solute carrier family 17, member 9	-1.47
ENSMUSG00000032965	Ift57	intraflagellar transport 57	-1.47
ENSMUSG00000051579	Tceal8	transcription elongation factor A (SII)-like 8	-1.47
ENSMUSG00000050132	Sarm1	sterile alpha and HEAT/Armadillo motif containing 1	-1.47
ENSMUSG00000029707	Fscn3	fascin actin-bundling protein 3	-1.47
ENSMUSG00000038648	Creb3l2	cAMP responsive element binding protein 3-like 2	-1.48
ENSMUSG00000041644	Slc5a12	solute carrier family 5 (sodium/glucose cotransporter), member 12	-1.48
ENSMUSG00000062785	Kcnc3	potassium voltage gated channel, Shaw-related subfamily, member 3	-1.48
ENSMUSG00000038893	Fam117a	family with sequence similarity 117, member A	-1.48
ENSMUSG00000103432	6720464F23Rik	RIKEN cDNA 6720464F23 gene	-1.48

ENSMUSG00000022440	C1qtnf6	C1q and tumor necrosis factor related protein 6	-1.48
ENSMUSG00000006675	P4htm	prolyl 4-hydroxylase, transmembrane (endoplasmic reticulum)	-1.48
ENSMUSG00000035513	Ntng2	netrin G2	-1.48
ENSMUSG00000026380	Tfcp2l1	transcription factor CP2-like 1	-1.49
ENSMUSG00000036611	Eepd1	endonuclease/exonuclease/phosphatase family domain containing 1	-1.49
ENSMUSG00000000204	Slfn4	schlafen 4	-1.49
ENSMUSG00000050295	Foxc1	forkhead box C1	-1.49
ENSMUSG00000073406	H2-Bl	histocompatibility 2, blastocyst	-1.49
ENSMUSG00000020178	Adora2a	adenosine A2a receptor	-1.49
ENSMUSG00000101587	Gm29036	predicted gene 29036	-1.49
ENSMUSG00000085003	Pip5k1bos	phosphatidylinositol-4-phosphate 5-kinase, type 1 beta, opposite strand	-1.49
ENSMUSG00000036872	Abcc12	ATP-binding cassette, sub-family C (CFTR/MRP), member 12	-1.49
ENSMUSG00000110497	9230110F11Rik	RIKEN cDNA 9230110F11 gene	-1.49
ENSMUSG00000097194	9330175E14Rik	RIKEN cDNA 9330175E14 gene	-1.49
ENSMUSG00000087625	4930419G24Rik	RIKEN cDNA 4930419G24 gene	-1.49
ENSMUSG00000058488	Kl	klotho	-1.49
ENSMUSG00000021565	Slc6a19	solute carrier family 6 (neurotransmitter transporter), member 19	-1.50
ENSMUSG00000053862	Slc51b	solute carrier family 51, beta subunit	-1.50
ENSMUSG00000024232	Bambi	BMP and activin membrane-bound inhibitor	-1.50
ENSMUSG00000004187	Kifc2	kinesin family member C2	-1.50
ENSMUSG00000058163	Gm5431	predicted gene 5431	-1.50
ENSMUSG00000024940	Ltbp3	latent transforming growth factor beta binding protein 3	-1.50
ENSMUSG00000113680	Gm47405	predicted gene, 47405	-1.50
ENSMUSG00000085852	Gm13807	predicted gene 13807	-1.50
ENSMUSG00000055805	Fmnl1	formin-like 1	-1.50
ENSMUSG00000022340	Sybu	syntabulin (syntaxin-interacting)	-1.50
ENSMUSG00000032085	Tagln	transgelin	-1.50
ENSMUSG00000102147	Gm38055	predicted gene, 38055	-1.50
ENSMUSG00000036040	Adamtsl2	ADAMTS-like 2	-1.50
ENSMUSG00000039168	Dap	death-associated protein	-1.51
ENSMUSG00000003363	Pld3	phospholipase D family, member 3	-1.51
ENSMUSG00000027602	Map1lc3a	microtubule-associated protein 1 light chain 3 alpha	-1.51
ENSMUSG00000032080	Apoa4	apolipoprotein A-IV	-1.51
ENSMUSG00000003032	Klf4	Kruppel-like factor 4 (gut)	-1.51
ENSMUSG00000032454	Rbp2	retinol binding protein 2, cellular	-1.51
ENSMUSG00000028519	Dab1	disabled 1	-1.51
ENSMUSG00000067813	Xkr9	X-linked Kx blood group related 9	-1.51
ENSMUSG00000036002	Fam214b	family with sequence similarity 214, member B	-1.51
ENSMUSG00000094584	Ms4a18	membrane-spanning 4-domains, subfamily A, member 18	-1.51
ENSMUSG00000039934	Gsap	gamma-secretase activating protein	-1.51
ENSMUSG00000028427	Aqp7	aquaporin 7	-1.51
ENSMUSG00000039963	Ccdc40	coiled-coil domain containing 40	-1.51
ENSMUSG00000037605	Adgrl3	adhesion G protein-coupled receptor L3	-1.51
ENSMUSG00000115555	Gm49307	predicted gene, 49307	-1.51
ENSMUSG00000003418	St8sia6	ST8 alpha-N-acetyl-neuraminide alpha-2,8-sialyltransferase 6	-1.51
ENSMUSG00000037762	Slc16a9	solute carrier family 16 (monocarboxylic acid transporters), member 9	-1.51
ENSMUSG00000046876	Atxn1	ataxin 1	-1.52
ENSMUSG00000013643	Lypd8	LY6/PLAUR domain containing 8	-1.52
ENSMUSG00000032226	Gcnt3	glucosaminyl (N-acetyl) transferase 3, mucin type	-1.52
ENSMUSG00000036377	C530008M17Rik	RIKEN cDNA C530008M17 gene	-1.52
ENSMUSG00000022938	Fam3b	family with sequence similarity 3, member B	-1.52
ENSMUSG00000108291	Gm44292	predicted gene, 44292	-1.52
ENSMUSG00000026222	Sp100	nuclear antigen Sp100	-1.52
ENSMUSG00000021281	Tnfaip2	tumor necrosis factor, alpha-induced protein 2	-1.52
ENSMUSG00000019359	Gdpd2	glycerophosphodiester phosphodiesterase domain containing 2	-1.52
ENSMUSG00000039004	Bmp6	bone morphogenetic protein 6	-1.52
ENSMUSG00000074183	Gsta1	glutathione S-transferase, alpha 1 (Ya)	-1.52
ENSMUSG00000045664	Cdc42ep2	CDC42 effector protein (Rho GTPase binding) 2	-1.52
ENSMUSG00000104776	Gm43691	predicted gene 43691	-1.52
ENSMUSG00000113404	Gm48622	predicted gene, 48622	-1.52
ENSMUSG00000102481	Gm37925	predicted gene, 37925	-1.52
ENSMUSG00000040093	Bmf	BCL2 modifying factor	-1.53
ENSMUSG00000020847	Rph3al	rabphilin 3A-like (without C2 domains)	-1.53
ENSMUSG00000047496	Rnf152	ring finger protein 152	-1.53
ENSMUSG00000074195	Clca4b	chloride channel accessory 4B	-1.53
ENSMUSG00000085747	Slc13a2os	solute carrier family 13 (sodium-dependent dicarboxylate transporter), member 2, opposite strand	-1.53
ENSMUSG00000045349	Sh2d5	SH2 domain containing 5	-1.53
ENSMUSG00000106229	Gm19409	predicted gene, 19409	-1.53
ENSMUSG00000070687	Htr1d	5-hydroxytryptamine (serotonin) receptor 1D	-1.53
ENSMUSG00000056596	Trnp1	TMF1-regulated nuclear protein 1	-1.53
ENSMUSG00000029343	Crybb1	crystallin, beta B1	-1.53
ENSMUSG00000043822	Adamtsl5	ADAMTS-like 5	-1.54
ENSMUSG00000068547	Clca4a	chloride channel accessory 4A	-1.54

ENSMUSG00000025528	2010106E10Rik	RIKEN cDNA 2010106E10 gene	-1.54
ENSMUSG00000053158	Fes	feline sarcoma oncogene	-1.54
ENSMUSG00000079038	D130040H23Rik	RIKEN cDNA D130040H23 gene	-1.54
ENSMUSG00000035390	Brsk1	BR serine/threonine kinase 1	-1.54
ENSMUSG00000025854	Fam20c	family with sequence similarity 20, member C	-1.54
ENSMUSG00000042096	Dao	D-amino acid oxidase	-1.54
ENSMUSG00000085830	Grin1os	glutamate receptor, ionotropic, NMDA1 (zeta 1), opposite strand	-1.54
ENSMUSG00000041710	Trpc5	transient receptor potential cation channel, subfamily C, member 5	-1.54
ENSMUSG00000051678	Pcdhb6	protocadherin beta 6	-1.54
ENSMUSG00000022564	Grina	glutamate receptor, ionotropic, N-methyl D-aspartate-associated protein 1 (glutamate binding)	-1.55
ENSMUSG00000092341	Malat1	metastasis associated lung adenocarcinoma transcript 1 (non-coding RNA)	-1.55
ENSMUSG00000037390	Muc3	mucin 3, intestinal	-1.55
ENSMUSG00000018378	Cuedc1	CUE domain containing 1	-1.55
ENSMUSG00000040447	Spns2	spinster homolog 2	-1.55
ENSMUSG00000006464	Bbs1	Bardet-Biedl syndrome 1 (human)	-1.55
ENSMUSG00000040432	Ltb4r2	leukotriene B4 receptor 2	-1.55
ENSMUSG00000056973	Ces1d	carboxylesterase 1D	-1.55
ENSMUSG00000028758	Kif17	kinesin family member 17	-1.55
ENSMUSG00000108603	5330411O13Rik	RIKEN cDNA 5330411O13 gene	-1.55
ENSMUSG00000020080	Hkdc1	hexokinase domain containing 1	-1.56
ENSMUSG00000006930	Hap1	huntingtin-associated protein 1	-1.56
ENSMUSG00000039652	Cpeb3	cytoplasmic polyadenylation element binding protein 3	-1.56
ENSMUSG00000049551	Fzd9	frizzled class receptor 9	-1.56
ENSMUSG00000074196	Clca4c-ps	chloride channel accessory 4C, pseudogene	-1.56
ENSMUSG00000019916	P4ha1	procollagen-proline, 2-oxoglutarate 4-dioxygenase (proline 4-hydroxylase), alpha 1 polypeptide	-1.56
ENSMUSG00000026620	Mark1	MAP/microtubule affinity regulating kinase 1	-1.56
ENSMUSG00000022146	Osmr	oncostatin M receptor	-1.56
ENSMUSG00000030317	Timp4	tissue inhibitor of metalloproteinase 4	-1.56
ENSMUSG00000022575	Gsdmd	gasdermin D	-1.57
ENSMUSG00000039621	Prex1	phosphatidylinositol-3,4,5-trisphosphate-dependent Rac exchange factor 1	-1.57
ENSMUSG00000035493	Tgfbi	transforming growth factor, beta induced	-1.57
ENSMUSG00000073399	Trim40	tripartite motif-containing 40	-1.57
ENSMUSG00000014303	Glis2	GLIS family zinc finger 2	-1.57
ENSMUSG00000040714	Klc3	kinesin light chain 3	-1.57
ENSMUSG00000085363	Gm15478	predicted gene 15478	-1.57
ENSMUSG00000036912	Piwil4	piwi-like RNA-mediated gene silencing 4	-1.57
ENSMUSG00000117182	CT025731.2	ribosomal protein S6 (Rps6) pseudogene	-1.57
ENSMUSG00000105326	6030400A10Rik	RIKEN cDNA 6030400A10 gene	-1.57
ENSMUSG00000116506	5730414N17Rik	RIKEN cDNA 5730414N17 gene	-1.57
ENSMUSG00000086039	Gm12227	predicted gene 12227	-1.57
ENSMUSG00000031523	Dlc1	deleted in liver cancer 1	-1.57
ENSMUSG00000087082	Gm15423	predicted gene 15423	-1.57
ENSMUSG00000023259	Slc26a6	solute carrier family 26, member 6	-1.58
ENSMUSG00000025497	Cdhr5	cadherin-related family member 5	-1.58
ENSMUSG00000037005	Xpnpep2	X-prolyl aminopeptidase (aminopeptidase P) 2, membrane-bound	-1.58
ENSMUSG00000061728	Btnl7-ps	butyrophilin-like 7, pseudogene	-1.58
ENSMUSG00000046794	Ppp1r3b	protein phosphatase 1, regulatory subunit 3B	-1.58
ENSMUSG00000081219	Bambi-ps1	BMP and activin membrane-bound inhibitor, pseudogene (Xenopus laevis)	-1.58
ENSMUSG00000051951	Xkr4	X-linked Kx blood group related 4	-1.58
ENSMUSG00000061576	Dpp6	dipeptidylpeptidase 6	-1.58
ENSMUSG00000027199	Gatm	glycine amidinotransferase (L-arginine:glycine amidinotransferase)	-1.58
ENSMUSG00000104519	Gm37161	predicted gene, 37161	-1.58
ENSMUSG00000003153	Slc2a3	solute carrier family 2 (facilitated glucose transporter), member 3	-1.58
ENSMUSG00000073293	Nudt10	nudix (nucleoside diphosphate linked moiety X)-type motif 10	-1.58
ENSMUSG00000041261	Car8	carbonic anhydrase 8	-1.59
ENSMUSG00000034586	Hid1	HID1 domain containing	-1.59
ENSMUSG00000029727	Cyp3a13	cytochrome P450, family 3, subfamily a, polypeptide 13	-1.59
ENSMUSG00000006345	Ggt1	gamma-glutamyltransferase 1	-1.59
ENSMUSG00000041351	Rap1gap	Rap1 GTPase-activating protein	-1.59
ENSMUSG00000024511	Rab27b	RAB27B, member RAS oncogene family	-1.59
ENSMUSG00000025787	Tgm4	transglutaminase 4 (prostate)	-1.59
ENSMUSG00000016356	Col20a1	collagen, type XX, alpha 1	-1.59
ENSMUSG00000074067	Gm10619	predicted gene 10619	-1.59
ENSMUSG00000024011	Pi16	peptidase inhibitor 16	-1.59
ENSMUSG00000117246	CT025671.2	periphilin 1 (Pphln1) pseudogene	-1.59
ENSMUSG00000006342	Susd2	sushi domain containing 2	-1.60
ENSMUSG00000003378	Grik5	glutamate receptor, ionotropic, kainate 5 (gamma 2)	-1.60
ENSMUSG00000036295	Lrrn3	leucine rich repeat protein 3, neuronal	-1.60
ENSMUSG00000035775	Krt20	keratin 20	-1.60
ENSMUSG00000028737	Aldh4a1	aldehyde dehydrogenase 4 family, member A1	-1.60
ENSMUSG00000017667	Zfp334	zinc finger protein 334	-1.60
ENSMUSG00000061947	Serpina10	serine (or cysteine) peptidase inhibitor, clade A (alpha-1 antiproteinase, antitrypsin), member 10	-1.60
ENSMUSG00000117286	Gm1043	predicted 1043	-1.60

ENSMUSG00000055357	4933400A11Rik	RIKEN cDNA 4933400A11 gene	-1.60
ENSMUSG00000024027	Glp1r	glucagon-like peptide 1 receptor	-1.60
ENSMUSG00000066392	Nrxn3	neurexin III	-1.60
ENSMUSG00000107427	Gm43948	predicted gene, 43948	-1.60
ENSMUSG00000042846	Lrrtm3	leucine rich repeat transmembrane neuronal 3	-1.60
ENSMUSG00000019960	Dusp6	dual specificity phosphatase 6	-1.61
ENSMUSG00000055978	Fut2	fucosyltransferase 2	-1.61
ENSMUSG00000034427	Myo15b	myosin XVB	-1.61
ENSMUSG00000022546	Gpt	glutamic pyruvic transaminase, soluble	-1.61
ENSMUSG00000055114	Anxa13	annexin A13	-1.61
ENSMUSG00000047787	Flrt1	fibronectin leucine rich transmembrane protein 1	-1.61
ENSMUSG00000100094	1810008I18Rik	RIKEN cDNA 1810008I18 gene	-1.61
ENSMUSG00000040473	Cfap69	cilia and flagella associated protein 69	-1.61
ENSMUSG00000062488	Ifit3b	interferon-induced protein with tetratricopeptide repeats 3B	-1.61
ENSMUSG00000024526	Cidea	cell death-inducing DNA fragmentation factor, alpha subunit-like effector A	-1.61
ENSMUSG00000031074	Fgf3	fibroblast growth factor 3	-1.61
ENSMUSG00000045875	Adra1a	adrenergic receptor, alpha 1a	-1.61
ENSMUSG00000020182	Ddc	dopa decarboxylase	-1.62
ENSMUSG00000038903	Ccdc68	coiled-coil domain containing 68	-1.62
ENSMUSG00000114601	Gm48188	predicted gene, 48188	-1.62
ENSMUSG00000052921	Arhgef15	Rho guanine nucleotide exchange factor (GEF) 15	-1.62
ENSMUSG00000025104	Hdgfl3	HDGF like 3	-1.62
ENSMUSG00000026765	Lypd6b	LY6/PLAUR domain containing 6B	-1.62
ENSMUSG00000027107	Chrna1	cholinergic receptor, nicotinic, alpha polypeptide 1 (muscle)	-1.62
ENSMUSG00000104362	Gm37928	predicted gene, 37928	-1.62
ENSMUSG00000022358	Fbxo32	F-box protein 32	-1.63
ENSMUSG00000010307	Tmem86a	transmembrane protein 86A	-1.63
ENSMUSG00000102224	4930447F24Rik	RIKEN cDNA 4930447F24 gene	-1.63
ENSMUSG00000025175	Fn3k	fructosamine 3 kinase	-1.63
ENSMUSG00000090743	Gm17213	predicted gene 17213	-1.63
ENSMUSG00000023328	Ache	acetylcholinesterase	-1.64
ENSMUSG00000061751	Kalrn	kalirin, RhoGEF kinase	-1.64
ENSMUSG00000040624	Plekhg1	pleckstrin homology domain containing, family G (with RhoGef domain) member 1	-1.64
ENSMUSG00000035429	Ptprh	protein tyrosine phosphatase, receptor type, H	-1.64
ENSMUSG00000040613	Apobec1	apolipoprotein B mRNA editing enzyme, catalytic polypeptide 1	-1.64
ENSMUSG00000031635	Anxa10	annexin A10	-1.64
ENSMUSG00000020573	Pik3cg	phosphatidylinositol-4,5-bisphosphate 3-kinase catalytic subunit gamma	-1.64
ENSMUSG00000074461	Gm10699	predicted gene 10699	-1.64
ENSMUSG00000030911	Zp2	zona pellucida glycoprotein 2	-1.64
ENSMUSG00000039234	Sec24d	Sec24 related gene family, member D (S. cerevisiae)	-1.65
ENSMUSG00000002233	Rhoc	ras homolog family member C	-1.65
ENSMUSG00000036553	Sh3tc1	SH3 domain and tetratricopeptide repeats 1	-1.65
ENSMUSG00000102423	Gm37465	predicted gene, 37465	-1.65
ENSMUSG00000055745	Rtl6	retrotransposon Gag like 6	-1.65
ENSMUSG00000111282	Gm47528	predicted gene, 47528	-1.65
ENSMUSG00000040310	Alx4	aristaless-like homeobox 4	-1.65
ENSMUSG00000099826	Scgb2b10	secretoglobin, family 2B, member 10	-1.65
ENSMUSG00000028713	Cyp4b1	cytochrome P450, family 4, subfamily b, polypeptide 1	-1.66
ENSMUSG00000021336	Slc17a4	solute carrier family 17 (sodium phosphate), member 4	-1.66
ENSMUSG00000044626	Liph	lipase, member H	-1.66
ENSMUSG00000003545	Fosb	FBJ osteosarcoma oncogene B	-1.66
ENSMUSG00000027230	Creb3l1	cAMP responsive element binding protein 3-like 1	-1.66
ENSMUSG00000021620	Acot12	acyl-CoA thioesterase 12	-1.66
ENSMUSG00000026405	C4bp	complement component 4 binding protein	-1.66
ENSMUSG00000010830	Kdelr3	KDEL (Lys-Asp-Glu-Leu) endoplasmic reticulum protein retention receptor 3	-1.66
ENSMUSG00000051166	Eml5	echinoderm microtubule associated protein like 5	-1.66
ENSMUSG00000037902	Sirpa	signal-regulatory protein alpha	-1.66
ENSMUSG00000097566	A930024N18Rik	RIKEN cDNA A930024N18 gene	-1.66
ENSMUSG00000035041	Creb3l3	cAMP responsive element binding protein 3-like 3	-1.67
ENSMUSG00000028943	Espn	espin	-1.67
ENSMUSG00000015944	Castor2	cytosolic arginine sensor for mTORC1 subunit 2	-1.67
ENSMUSG00000025268	Maged2	melanoma antigen, family D, 2	-1.67
ENSMUSG00000030930	Chst15	carbohydrate (N-acetylgalactosamine 4-sulfate 6-O) sulfotransferase 15	-1.67
ENSMUSG00000023966	Rsph9	radial spoke head 9 homolog (Chlamydomonas)	-1.67
ENSMUSG00000005045	Chd5	chromodomain helicase DNA binding protein 5	-1.67
ENSMUSG00000085925	Rtl1	retrotransposon Gaglike 1	-1.67
ENSMUSG00000036923	Stox1	storkhead box 1	-1.67
ENSMUSG00000095280	Gm21738	predicted gene, 21738	-1.67
ENSMUSG00000100522	Gm19280	predicted gene, 19280	-1.67
ENSMUSG00000108693	Gm45153	predicted gene 45153	-1.67
ENSMUSG00000010064	Slc38a3	solute carrier family 38, member 3	-1.67
ENSMUSG00000024109	Nrxn1	neurexin I	-1.67
ENSMUSG00000043458	Pcdhb12	protocadherin beta 12	-1.67

ENSMUSG00000009216	Fam163b	family with sequence similarity 163, member B	-1.67
ENSMUSG00000067212	H2-T23	histocompatibility 2, T region locus 23	-1.68
ENSMUSG00000043885	Slc36a4	solute carrier family 36 (proton/amino acid symporter), member 4	-1.68
ENSMUSG00000003863	Ppfia3	protein tyrosine phosphatase, receptor type, f polypeptide (PTPRF), interacting protein (liprin), alpha	-1.68
ENSMUSG00000039021	Ttc16	tetratricopeptide repeat domain 16	-1.68
ENSMUSG00000039485	Tspyl4	TSPY-like 4	-1.68
ENSMUSG00000104140	Gm37140	predicted gene, 37140	-1.68
ENSMUSG00000031202	Rab39b	RAB39B, member RAS oncogene family	-1.68
ENSMUSG00000072115	Ang	angiogenin, ribonuclease, RNase A family, 5	-1.69
ENSMUSG00000020681	Ace	angiotensin I converting enzyme (peptidyl-dipeptidase A) 1	-1.69
ENSMUSG00000021208	Ifi27l2b	interferon, alpha-inducible protein 27 like 2B	-1.69
ENSMUSG00000025557	Slc15a1	solute carrier family 15 (oligopeptide transporter), member 1	-1.69
ENSMUSG00000042439	Zfp532	zinc finger protein 532	-1.69
ENSMUSG00000031824	6430548M08Rik	RIKEN cDNA 6430548M08 gene	-1.69
ENSMUSG00000000627	Sema4f	sema domain, immunoglobulin domain (Ig), TM domain, and short cytoplasmic domain	-1.69
ENSMUSG00000027188	Pamr1	peptidase domain containing associated with muscle regeneration 1	-1.69
ENSMUSG00000020908	Myh3	myosin, heavy polypeptide 3, skeletal muscle, embryonic	-1.69
ENSMUSG00000021876	Rnase4	ribonuclease, RNase A family 4	-1.70
ENSMUSG00000021792	Fam213a	family with sequence similarity 213, member A	-1.70
ENSMUSG00000036814	Slc6a20a	solute carrier family 6 (neurotransmitter transporter), member 20A	-1.70
ENSMUSG00000048070	Pirt	phosphoinositide-interacting regulator of transient receptor potential channels	-1.70
ENSMUSG00000068600	Gml2	glycosylphosphatidylinositol anchored molecule like 2	-1.70
ENSMUSG00000043614	Vps37d	vacuolar protein sorting 37D	-1.70
ENSMUSG00000034758	Tle6	transducin-like enhancer of split 6	-1.70
ENSMUSG00000020151	Ptprr	protein tyrosine phosphatase, receptor type, R	-1.70
ENSMUSG00000022594	Lynx1	Ly6/neurotoxin 1	-1.70
ENSMUSG00000009092	Derl3	Der1-like domain family, member 3	-1.70
ENSMUSG00000006457	Actn3	actinin alpha 3	-1.70
ENSMUSG00000055733	Nap1l3	nucleosome assembly protein 1-like 3	-1.70
ENSMUSG00000027068	Dhrs9	dehydrogenase/reductase (SDR family) member 9	-1.71
ENSMUSG00000019055	Plod1	procollagen-lysine, 2-oxoglutarate 5-dioxygenase 1	-1.71
ENSMUSG00000038042	Ptpdc1	protein tyrosine phosphatase domain containing 1	-1.71
ENSMUSG00000017446	C1qtnf1	C1q and tumor necrosis factor related protein 1	-1.71
ENSMUSG00000115834	Gm10385	predicted gene 10385	-1.71
ENSMUSG00000110767	Gm31432	predicted gene, 31432	-1.71
ENSMUSG00000034677	Gpr142	G protein-coupled receptor 142	-1.71
ENSMUSG00000038497	Tmco3	transmembrane and coiled-coil domains 3	-1.72
ENSMUSG00000033213	AA467197	expressed sequence AA467197	-1.72
ENSMUSG00000100183	Gm28512	predicted gene 28512	-1.72
ENSMUSG00000018486	Wnt9b	wingless-type MMTV integration site family, member 9B	-1.72
ENSMUSG00000005267	Zfp287	zinc finger protein 287	-1.72
ENSMUSG00000000214	Th	tyrosine hydroxylase	-1.72
ENSMUSG00000002228	Ppm1j	protein phosphatase 1J	-1.73
ENSMUSG00000068551	Zfp467	zinc finger protein 467	-1.73
ENSMUSG00000110405	Gm45534	predicted gene 45534	-1.73
ENSMUSG00000069456	Rdh16	retinol dehydrogenase 16	-1.73
ENSMUSG00000107497	Gm44957	predicted gene 44957	-1.73
ENSMUSG00000106825	2510016D11Rik	RIKEN cDNA 2510016D11 gene	-1.74
ENSMUSG00000029154	Cwh43	cell wall biogenesis 43 C-terminal homolog	-1.74
ENSMUSG00000075014	Gm10800	predicted gene 10800	-1.74
ENSMUSG00000040016	Ptger3	prostaglandin E receptor 3 (subtype EP3)	-1.74
ENSMUSG00000111077	Gm48743	predicted gene, 48743	-1.74
ENSMUSG00000074344	Tmigd3	transmembrane and immunoglobulin domain containing 3	-1.74
ENSMUSG00000090706	Gm17233	predicted gene 17233	-1.74
ENSMUSG00000079174	Gm3054	predicted gene 3054	-1.75
ENSMUSG00000015094	Npdc1	neural proliferation, differentiation and control 1	-1.75
ENSMUSG00000032083	Apoa1	apolipoprotein A-I	-1.75
ENSMUSG00000090891	D6Ertd527e	DNA segment, Chr 6, ERATO Doi 527, expressed	-1.75
ENSMUSG00000050447	Lypd6	LY6/PLAUR domain containing 6	-1.75
ENSMUSG00000032353	Tmed3	transmembrane p24 trafficking protein 3	-1.76
ENSMUSG00000019564	Arid3a	AT rich interactive domain 3A (BRIGHT-like)	-1.76
ENSMUSG00000060487	Samd5	sterile alpha motif domain containing 5	-1.76
ENSMUSG00000009739	Pou6f1	POU domain, class 6, transcription factor 1	-1.76
ENSMUSG00000034949	Zfr2	zinc finger RNA binding protein 2	-1.76
ENSMUSG00000000392	Fap	fibroblast activation protein	-1.76
ENSMUSG00000060063	Alox5ap	arachidonate 5-lipoxygenase activating protein	-1.76
ENSMUSG00000001827	Folr1	folate receptor 1 (adult)	-1.76
ENSMUSG00000110301	Gm35363	predicted gene, 35363	-1.76
ENSMUSG00000047161	Chst9	carbohydrate (N-acetylgalactosamine 4-0) sulfotransferase 9	-1.76
ENSMUSG00000038807	Rap1gap2	RAP1 GTPase activating protein 2	-1.77
ENSMUSG00000043079	Synpo	synaptopodin	-1.77
ENSMUSG00000040270	Bach2	BTB and CNC homology, basic leucine zipper transcription factor 2	-1.77
ENSMUSG00000040415	Dtx3	deltex 3, E3 ubiquitin ligase	-1.77

ENSMUSG00000029630	Cyp3a25	cytochrome P450, family 3, subfamily a, polypeptide 25	-1.77
ENSMUSG00000038305	Spats2l	spermatogenesis associated, serine-rich 2-like	-1.78
ENSMUSG00000026639	Lamb3	laminin, beta 3	-1.78
ENSMUSG00000032420	Nt5e	5' nucleotidase, ecto	-1.78
ENSMUSG00000032549	Rab6b	RAB6B, member RAS oncogene family	-1.78
ENSMUSG00000055737	Ghr	growth hormone receptor	-1.79
ENSMUSG00000030364	Clec2h	C-type lectin domain family 2, member h	-1.79
ENSMUSG00000074892	B3galt5	UDP-Gal:betaGlcNAc beta 1,3-galactosyltransferase, polypeptide 5	-1.79
ENSMUSG00000071203	Naip5	NLR family, apoptosis inhibitory protein 5	-1.79
ENSMUSG00000027790	Sis	sucrase isomaltase (alpha-glucosidase)	-1.79
ENSMUSG00000087651	1500009L16Rik	RIKEN cDNA 1500009L16 gene	-1.79
ENSMUSG00000034438	Gbp8	guanylate-binding protein 8	-1.79
ENSMUSG00000030228	Pik3c2g	phosphatidylinositol-4-phosphate 3-kinase catalytic subunit type 2 gamma	-1.79
ENSMUSG00000081270	Gm11653	predicted gene 11653	-1.79
ENSMUSG00000024530	Prelid3a	PRELI domain containing 3A	-1.79
ENSMUSG00000114113	Gm47639	predicted gene, 47639	-1.79
ENSMUSG00000038375	Trp53inp2	transformation related protein 53 inducible nuclear protein 2	-1.80
ENSMUSG00000020646	Mboat2	membrane bound O-acyltransferase domain containing 2	-1.80
ENSMUSG00000033427	Upb1	ureidopropionase, beta	-1.80
ENSMUSG00000020062	Slc5a8	solute carrier family 5 (iodide transporter), member 8	-1.80
ENSMUSG00000017868	Sgk2	serum/glucocorticoid regulated kinase 2	-1.80
ENSMUSG00000060735	Rxfp3	relaxin family peptide receptor 3	-1.80
ENSMUSG00000006576	Slc4a3	solute carrier family 4 (anion exchanger), member 3	-1.80
ENSMUSG00000023826	Prkn	parkin RBR E3 ubiquitin protein ligase	-1.80
ENSMUSG00000104211	Gm37985	predicted gene, 37985	-1.80
ENSMUSG00000102424	Paupar	Pax6 upstream antisense RNA	-1.80
ENSMUSG00000042269	Fam92b	family with sequence similarity 92, member B	-1.80
ENSMUSG00000029185	Fam114a1	family with sequence similarity 114, member A1	-1.81
ENSMUSG00000049690	Nckap5	NCK-associated protein 5	-1.81
ENSMUSG00000018623	Mmp7	matrix metallopeptidase 7	-1.81
ENSMUSG00000029273	Sult1d1	sulfotransferase family 1D, member 1	-1.81
ENSMUSG00000030659	Nucb2	nucleobindin 2	-1.81
ENSMUSG00000029156	Sgcb	sarcoglycan, beta (dystrophin-associated glycoprotein)	-1.81
ENSMUSG00000044072	Eml6	echinoderm microtubule associated protein like 6	-1.81
ENSMUSG00000044748	Defb1	defensin beta 1	-1.81
ENSMUSG00000052387	Trpm3	transient receptor potential cation channel, subfamily M, member 3	-1.81
ENSMUSG00000025006	Sorbs1	sorbin and SH3 domain containing 1	-1.82
ENSMUSG00000017652	Cd40	CD40 antigen	-1.82
ENSMUSG00000047143	Dmrta2	doublesex and mab-3 related transcription factor like family A2	-1.82
ENSMUSG00000087326	Gm12503	predicted gene 12503	-1.82
ENSMUSG00000017692	Rhbdl3	rhomboid like 3	-1.82
ENSMUSG00000020363	Gfpt2	glutamine fructose-6-phosphate transaminase 2	-1.82
ENSMUSG00000046169	Adamts6	a disintegrin-like and metallopeptidase (reprolysin type) with thrombospondin type 1 motif, 6	-1.82
ENSMUSG00000050600	Zfp831	zinc finger protein 831	-1.82
ENSMUSG00000060935	Tmem263	transmembrane protein 263	-1.83
ENSMUSG00000037606	Osbpl5	oxysterol binding protein-like 5	-1.83
ENSMUSG00000050747	Trim15	tripartite motif-containing 15	-1.83
ENSMUSG00000032186	Tmod2	tropomodulin 2	-1.83
ENSMUSG00000017211	Gsdma2	gasdermin A2	-1.83
ENSMUSG00000028716	Pdzk1ip1	PDZK1 interacting protein 1	-1.83
ENSMUSG00000032773	Chrm1	cholinergic receptor, muscarinic 1, CNS	-1.83
ENSMUSG00000046908	Ltb4r1	leukotriene B4 receptor 1	-1.83
ENSMUSG00000009394	Syn2	synapsin II	-1.83
ENSMUSG00000061958	Gm14851	predicted gene 14851	-1.83
ENSMUSG00000102184	Gm37192	predicted gene, 37192	-1.83
ENSMUSG00000034731	Dgkh	diacylglycerol kinase, eta	-1.84
ENSMUSG00000022602	Arc	activity regulated cytoskeletal-associated protein	-1.84
ENSMUSG00000001663	Gstt1	glutathione S-transferase, theta 1	-1.84
ENSMUSG00000028246	Faxc	failed axon connections homolog	-1.84
ENSMUSG00000084934	Gm16035	predicted gene 16035	-1.84
ENSMUSG00000028845	Tekt2	tektin 2	-1.84
ENSMUSG00000063873	Slc24a3	solute carrier family 24 (sodium/potassium/calcium exchanger), member 3	-1.84
ENSMUSG00000103284	3110080O07Rik	RIKEN cDNA 3110080O07 gene	-1.85
ENSMUSG00000108427	Gm36696	predicted gene, 36696	-1.85
ENSMUSG00000045518	Onecut3	one cut domain, family member 3	-1.85
ENSMUSG00000054934	Kcnmb4	potassium large conductance calcium-activated channel, subfamily M, beta member 4	-1.85
ENSMUSG00000060962	Dmkn	dermokine	-1.85
ENSMUSG00000112601	Gm47590	predicted gene, 47590	-1.85
ENSMUSG00000020990	Cdkl1	cyclin-dependent kinase-like 1 (CDC2-related kinase)	-1.85
ENSMUSG00000029275	Gfi1	growth factor independent 1	-1.86
ENSMUSG00000032816	Igdcc4	immunoglobulin superfamily, DCC subclass, member 4	-1.86
ENSMUSG00000015981	Stk32c	serine/threonine kinase 32C	-1.86
ENSMUSG00000050821	Fam131a	family with sequence similarity 131, member A	-1.86

ENSMUSG00000015243	Abca1	ATP-binding cassette, sub-family A (ABC1), member 1	-1.87
ENSMUSG00000033917	Gde1	glycerophosphodiester phosphodiesterase 1	-1.87
ENSMUSG00000049191	Rtl5	retrotransposon Gag like 5	-1.87
ENSMUSG00000021765	Fst	follistatin	-1.87
ENSMUSG00000033209	Ttc28	tetratricopeptide repeat domain 28	-1.87
ENSMUSG00000103701	Gm10728	predicted gene 10728	-1.87
ENSMUSG00000006711	D130043K22Rik	RIKEN cDNA D130043K22 gene	-1.88
ENSMUSG00000029798	Herc6	hect domain and RLD 6	-1.88
ENSMUSG00000008153	Clstn3	calsyntenin 3	-1.88
ENSMUSG00000040387	Klhl32	kelch-like 32	-1.88
ENSMUSG00000020953	Coch	cochlin	-1.88
ENSMUSG00000046159	Chrm3	cholinergic receptor, muscarinic 3, cardiac	-1.89
ENSMUSG00000103585	Pcdhgb4	protocadherin gamma subfamily B, 4	-1.89
ENSMUSG00000030087	Klf15	Kruppel-like factor 15	-1.89
ENSMUSG00000024065	Ehd3	EH-domain containing 3	-1.89
ENSMUSG00000047904	Sstr2	somatostatin receptor 2	-1.89
ENSMUSG00000108348	Gm42372	predicted gene, 42372	-1.89
ENSMUSG00000026070	Il18r1	interleukin 18 receptor 1	-1.89
ENSMUSG00000002633	Shh	sonic hedgehog	-1.89
ENSMUSG00000026335	Pam	peptidylglycine alpha-amidating monooxygenase	-1.90
ENSMUSG00000033684	Qsox1	quiescin Q6 sulfhydryl oxidase 1	-1.90
ENSMUSG00000034459	Ifit1	interferon-induced protein with tetratricopeptide repeats 1	-1.90
ENSMUSG00000031791	Tmem38a	transmembrane protein 38A	-1.90
ENSMUSG00000025701	Alox5	arachidonate 5-lipoxygenase	-1.90
ENSMUSG00000104136	Gm36955	predicted gene, 36955	-1.90
ENSMUSG00000029428	Stx2	syntaxin 2	-1.90
ENSMUSG00000040148	Hmx3	H6 homeobox 3	-1.90
ENSMUSG00000114282	5330431K02Rik	RIKEN cDNA 5330431K02 gene	-1.90
ENSMUSG00000085329	2810404F17Rik	RIKEN cDNA 2810404F17 gene	-1.91
ENSMUSG00000038541	Srd5a2	steroid 5 alpha-reductase 2	-1.91
ENSMUSG00000072623	Zfp9	zinc finger protein 9	-1.91
ENSMUSG00000038271	Iffo1	intermediate filament family orphan 1	-1.91
ENSMUSG00000106320	Gm42992	predicted gene 42992	-1.91
ENSMUSG00000039419	Cntnap2	contactin associated protein-like 2	-1.91
ENSMUSG00000010601	Apol7a	apolipoprotein L 7a	-1.92
ENSMUSG00000005672	Kit	KIT proto-oncogene receptor tyrosine kinase	-1.92
ENSMUSG00000029408	Abcb9	ATP-binding cassette, sub-family B (MDR/TAP), member 9	-1.92
ENSMUSG00000073802	Cdkn2b	cyclin dependent kinase inhibitor 2B	-1.92
ENSMUSG00000004933	Matk	megakaryocyte-associated tyrosine kinase	-1.92
ENSMUSG00000023868	Pde10a	phosphodiesterase 10A	-1.92
ENSMUSG00000020882	Cacnb1	calcium channel, voltage-dependent, beta 1 subunit	-1.92
ENSMUSG00000111014	Gm47795	predicted gene, 47795	-1.92
ENSMUSG00000022580	Rhpn1	rhophilin, Rho GTPase binding protein 1	-1.92
ENSMUSG00000021379	Id4	inhibitor of DNA binding 4	-1.93
ENSMUSG00000051832	E230016K23Rik	RIKEN cDNA E230016K23 gene	-1.93
ENSMUSG00000020435	Osbp2	oxysterol binding protein 2	-1.93
ENSMUSG00000068196	Col8a1	collagen, type VIII, alpha 1	-1.93
ENSMUSG00000114626	Gm48266	predicted gene, 48266	-1.93
ENSMUSG00000067889	Sptbn2	spectrin beta, non-erythrocytic 2	-1.94
ENSMUSG00000035930	Chst4	carbohydrate (chondroitin 6/keratan) sulfotransferase 4	-1.94
ENSMUSG00000034116	Vav1	vav 1 oncogene	-1.94
ENSMUSG00000042312	S100a13	S100 calcium binding protein A13	-1.94
ENSMUSG00000050212	Eva1b	eva-1 homolog B (C. elegans)	-1.94
ENSMUSG00000084950	Gm5577	predicted gene 5577	-1.94
ENSMUSG00000027249	F2	coagulation factor II	-1.94
ENSMUSG00000053168	9030619P08Rik	RIKEN cDNA 9030619P08 gene	-1.94
ENSMUSG00000032101	Ddx25	DEAD (Asp-Glu-Ala-Asp) box polypeptide 25	-1.94
ENSMUSG00000018217	Pmp22	peripheral myelin protein 22	-1.95
ENSMUSG00000041120	Nbl1	neuroblastoma, suppression of tumorigenicity 1	-1.95
ENSMUSG00000027612	Mmp24	matrix metallopeptidase 24	-1.95
ENSMUSG00000080115	Eef1akmt3	EEF1A lysine methyltransferase 3	-1.95
ENSMUSG00000018822	Sfrp5	secreted frizzled-related sequence protein 5	-1.95
ENSMUSG00000059900	Tmem40	transmembrane protein 40	-1.95
ENSMUSG00000029359	Tesc	tescalcin	-1.96
ENSMUSG00000074442	Defa31	defensin, alpha, 31	-1.96
ENSMUSG00000110131	Gm18066	predicted gene, 18066	-1.96
ENSMUSG00000103331	Gm37995	predicted gene, 37995	-1.96
ENSMUSG00000002980	Bcam	basal cell adhesion molecule	-1.97
ENSMUSG00000026961	Lrrc26	leucine rich repeat containing 26	-1.97
ENSMUSG00000078670	Fam174b	family with sequence similarity 174, member B	-1.97
ENSMUSG00000027801	Tm4sf4	transmembrane 4 superfamily member 4	-1.97
ENSMUSG00000073043	Atoh1	atonal bHLH transcription factor 1	-1.98
ENSMUSG00000000202	Btbd17	BTB (POZ) domain containing 17	-1.98

ENSMUSG00000066516	Klk1b21	kallikrein 1-related peptidase b21	-1.98
ENSMUSG00000069114	Zbtb10	zinc finger and BTB domain containing 10	-1.99
ENSMUSG00000031845	Bco1	beta-carotene oxygenase 1	-1.99
ENSMUSG00000035509	Fbxl21	F-box and leucine-rich repeat protein 21	-1.99
ENSMUSG00000078161	Erich3	glutamate rich 3	-1.99
ENSMUSG00000034460	Six4	sine oculis-related homeobox 4	-1.99
ENSMUSG00000007783	Cpt1c	carnitine palmitoyltransferase 1c	-1.99
ENSMUSG00000027175	Tcp11l1	t-complex 11 like 1	-2.00
ENSMUSG00000035067	Xkr6	X-linked Kx blood group related 6	-2.00
ENSMUSG00000038793	Lefty1	left right determination factor 1	-2.00
ENSMUSG00000040181	Fmo1	flavin containing monooxygenase 1	-2.00
ENSMUSG00000094818	Defa32	defensin, alpha, 32	-2.00
ENSMUSG00000017943	Gdap1l1	ganglioside-induced differentiation-associated protein 1-like 1	-2.00
ENSMUSG00000078650	G6pc	glucose-6-phosphatase, catalytic	-2.01
ENSMUSG00000057069	Ero1lb	ERO1-like beta (S. cerevisiae)	-2.01
ENSMUSG00000025743	Sdc3	syndecan 3	-2.01
ENSMUSG00000049804	Armcx4	armadillo repeat containing, X-linked 4	-2.01
ENSMUSG00000026797	Stxbp1	syntaxin binding protein 1	-2.02
ENSMUSG00000053617	Sh3pxd2a	SH3 and PX domains 2A	-2.02
ENSMUSG00000048330	Ric3	RIC3 acetylcholine receptor chaperone	-2.02
ENSMUSG00000038587	Akap12	A kinase (PRKA) anchor protein (gravin) 12	-2.02
ENSMUSG00000063687	Pcdhb5	protocadherin beta 5	-2.02
ENSMUSG00000016179	Camk1g	calcium/calmodulin-dependent protein kinase I gamma	-2.02
ENSMUSG00000027938	Creb3l4	cAMP responsive element binding protein 3-like 4	-2.03
ENSMUSG00000094651	Gal3st2	galactose-3-O-sulfotransferase 2	-2.03
ENSMUSG00000053046	Brsk2	BR serine/threonine kinase 2	-2.03
ENSMUSG00000032661	Oas3	2'-5' oligoadenylate synthetase 3	-2.03
ENSMUSG00000019944	Rhobtb1	Rho-related BTB domain containing 1	-2.03
ENSMUSG00000060508	Nlrp9b	NLR family, pyrin domain containing 9B	-2.03
ENSMUSG00000046191	Pcdhb20	protocadherin beta 20	-2.03
ENSMUSG00000048481	Mypop	Myb-related transcription factor, partner of profilin	-2.03
ENSMUSG00000028865	Cd164l2	CD164 sialomucin-like 2	-2.03
ENSMUSG00000117220	CT025671.1	novel transcript	-2.03
ENSMUSG00000064373	Selenop	selenoprotein P	-2.04
ENSMUSG00000020230	Prmt2	protein arginine N-methyltransferase 2	-2.04
ENSMUSG00000045312	Lhfpl2	lipoma HMGIC fusion partner-like 2	-2.04
ENSMUSG00000042286	Stab1	stabilin 1	-2.04
ENSMUSG00000026817	Ak1	adenylate kinase 1	-2.04
ENSMUSG00000093580	Gm20706	predicted gene 20706	-2.04
ENSMUSG00000037973	Ccdc129	coiled-coil domain containing 129	-2.04
ENSMUSG00000031028	Tub	tubby bipartite transcription factor	-2.04
ENSMUSG00000052584	Serp2	stress-associated endoplasmic reticulum protein family member 2	-2.04
ENSMUSG00000019933	Mrln	myoregulin	-2.04
ENSMUSG00000026834	Acvr1c	activin A receptor, type IC	-2.05
ENSMUSG00000049281	Scn3b	sodium channel, voltage-gated, type III, beta	-2.05
ENSMUSG00000032268	Tmprss5	transmembrane protease, serine 5 (spinesin)	-2.05
ENSMUSG00000021219	Rgs6	regulator of G-protein signaling 6	-2.05
ENSMUSG00000045094	Arhgef37	Rho guanine nucleotide exchange factor (GEF) 37	-2.06
ENSMUSG00000032609	Klhdc8b	kelch domain containing 8B	-2.06
ENSMUSG00000032740	Ccdc88a	coiled coil domain containing 88A	-2.06
ENSMUSG00000073680	Tmem88b	transmembrane protein 88B	-2.06
ENSMUSG00000038738	Shank1	SH3 and multiple ankyrin repeat domains 1	-2.06
ENSMUSG00000033805	Ephx4	epoxide hydrolase 4	-2.06
ENSMUSG00000053675	Tgm5	transglutaminase 5	-2.06
ENSMUSG00000029650	Slc46a3	solute carrier family 46, member 3	-2.07
ENSMUSG00000052131	Akr1b7	aldo-keto reductase family 1, member B7	-2.07
ENSMUSG00000001156	Mxd1	MAX dimerization protein 1	-2.07
ENSMUSG00000038175	Mylip	myosin regulatory light chain interacting protein	-2.07
ENSMUSG00000018927	Ccl6	chemokine (C-C motif) ligand 6	-2.07
ENSMUSG00000037341	Slc9a7	solute carrier family 9 (sodium/hydrogen exchanger), member 7	-2.07
ENSMUSG00000006784	Ttc25	tetratricopeptide repeat domain 25	-2.07
ENSMUSG00000038011	Dnah10	dynein, axonemal, heavy chain 10	-2.08
ENSMUSG00000029797	Sspo	SCO-spondin	-2.08
ENSMUSG00000030866	Ern2	endoplasmic reticulum (ER) to nucleus signalling 2	-2.09
ENSMUSG00000020581	Agr2	anterior gradient 2	-2.09
ENSMUSG00000078486	Perm1	PPARGC1 and ESRR induced regulator, muscle 1	-2.09
ENSMUSG00000063704	Mapk15	mitogen-activated protein kinase 15	-2.09
ENSMUSG00000093916	Gm379	predicted gene 379	-2.09
ENSMUSG00000048148	Nwd1	NACHT and WD repeat domain containing 1	-2.09
ENSMUSG00000004951	Hspb1	heat shock protein 1	-2.09
ENSMUSG00000070577	Gm572	predicted gene 572	-2.09
ENSMUSG00000069171	Nr2f1	nuclear receptor subfamily 2, group F, member 1	-2.09
ENSMUSG00000035580	Kcnh8	potassium voltage-gated channel, subfamily H (eag-related), member 8	-2.10

ENSMUSG00000022650	Retnlb	resistin like beta	-2.10
ENSMUSG00000035208	Slfn8	schlafen 8	-2.10
ENSMUSG00000097520	4930488L21Rik	RIKEN cDNA 4930488L21 gene	-2.10
ENSMUSG00000114751	5430425E15Rik	RIKEN cDNA 5430425E15 gene	-2.10
ENSMUSG00000032978	Guca2b	guanylate cyclase activator 2b (retina)	-2.11
ENSMUSG00000017204	Gsdma	gasdermin A	-2.11
ENSMUSG00000027513	Pck1	phosphoenolpyruvate carboxykinase 1, cytosolic	-2.11
ENSMUSG00000024124	Prss30	protease, serine 30	-2.11
ENSMUSG00000054893	Zfp667	zinc finger protein 667	-2.11
ENSMUSG00000115186	Gm49417	predicted gene, 49417	-2.11
ENSMUSG00000031442	Mcf2l	mcf.2 transforming sequence-like	-2.12
ENSMUSG00000024063	Lbh	limb-bud and heart	-2.12
ENSMUSG00000049892	Rasd1	RAS, dexamethasone-induced 1	-2.12
ENSMUSG00000056031	9330154J02Rik	RIKEN cDNA 9330154J02 gene	-2.12
ENSMUSG00000023927	Satb1	special AT-rich sequence binding protein 1	-2.12
ENSMUSG00000074439	Defa5	defensin, alpha, 5	-2.12
ENSMUSG00000074882	Cyp2c68	cytochrome P450, family 2, subfamily c, polypeptide 68	-2.13
ENSMUSG00000022469	Rapgef3	Rap guanine nucleotide exchange factor (GEF) 3	-2.13
ENSMUSG00000011832	Evi5l	ecotropic viral integration site 5 like	-2.13
ENSMUSG00000028634	Hivep3	human immunodeficiency virus type I enhancer binding protein 3	-2.13
ENSMUSG00000045348	Nyap1	neuronal tyrosine-phosphorylated phosphoinositide 3-kinase adaptor 1	-2.13
ENSMUSG00000019817	Plagl1	pleiomorphic adenoma gene-like 1	-2.13
ENSMUSG00000044162	Tnip3	TNFAIP3 interacting protein 3	-2.13
ENSMUSG00000021087	Rtn1	reticulon 1	-2.13
ENSMUSG00000025324	Atp10a	ATPase, class V, type 10A	-2.13
ENSMUSG00000104886	Gm43000	predicted gene 43000	-2.13
ENSMUSG00000070777	Ceacam20	carcinoembryonic antigen-related cell adhesion molecule 20	-2.14
ENSMUSG00000005973	Rcn1	reticulocalbin 1	-2.14
ENSMUSG00000026888	Grb14	growth factor receptor bound protein 14	-2.14
ENSMUSG00000035200	Chrnb4	cholinergic receptor, nicotinic, beta polypeptide 4	-2.14
ENSMUSG00000020429	Igfbp1	insulin-like growth factor binding protein 1	-2.14
ENSMUSG00000006241	Ccdc159	coiled-coil domain containing 159	-2.14
ENSMUSG00000006362	Cbfa2t3	core-binding factor, runt domain, alpha subunit 2, translocated to, 3 (human)	-2.15
ENSMUSG00000020641	Rsad2	radical S-adenosyl methionine domain containing 2	-2.15
ENSMUSG00000030483	Cyp2b10	cytochrome P450, family 2, subfamily b, polypeptide 10	-2.15
ENSMUSG00000074899	Sptbn5	spectrin beta, non-erythrocytic 5	-2.15
ENSMUSG00000092517	Art2a-ps	ADP-ribosyltransferase 2a, pseudogene	-2.15
ENSMUSG00000042804	Gpr153	G protein-coupled receptor 153	-2.15
ENSMUSG00000021609	Slc6a3	solute carrier family 6 (neurotransmitter transporter, dopamine), member 3	-2.15
ENSMUSG00000024924	Vldlr	very low density lipoprotein receptor	-2.16
ENSMUSG00000080316	Spaca6	sperm acrosome associated 6	-2.16
ENSMUSG00000022548	Apod	apolipoprotein D	-2.16
ENSMUSG00000036437	Npy1r	neuropeptide Y receptor Y1	-2.16
ENSMUSG00000061414	Cracr2a	calcium release activated channel regulator 2A	-2.17
ENSMUSG00000025656	Arhgef9	CDC42 guanine nucleotide exchange factor (GEF) 9	-2.17
ENSMUSG00000022012	Enox1	ecto-NOX disulfide-thiol exchanger 1	-2.17
ENSMUSG00000021913	Ogdhl	oxoglutarate dehydrogenase-like	-2.18
ENSMUSG00000038167	Plekhg6	pleckstrin homology domain containing, family G (with RhoGef domain) member 6	-2.19
ENSMUSG00000075702	Selenom	selenoprotein M	-2.19
ENSMUSG00000033730	Egr3	early growth response 3	-2.19
ENSMUSG00000000530	Acvrl1	activin A receptor, type II-like 1	-2.19
ENSMUSG00000046387	Pcdhb17	protocadherin beta 17	-2.19
ENSMUSG00000108041	Gm5154	predicted gene 5154	-2.19
ENSMUSG00000103466	Gm37247	predicted gene, 37247	-2.19
ENSMUSG00000112307	Gm48751	predicted gene, 48751	-2.20
ENSMUSG00000051435	Fhad1	forkhead-associated (FHA) phosphopeptide binding domain 1	-2.20
ENSMUSG00000042515	Mum1l1	melanoma associated antigen (mutated) 1-like 1	-2.20
ENSMUSG00000026131	Dst	dystonin	-2.21
ENSMUSG00000024215	Spdef	SAM pointed domain containing ets transcription factor	-2.21
ENSMUSG00000037953	A4gnt	alpha-1,4-N-acetylglucosaminyltransferase	-2.21
ENSMUSG00000097266	Gm26802	predicted gene, 26802	-2.21
ENSMUSG00000037921	Ddx60	DEAD (Asp-Glu-Ala-Asp) box polypeptide 60	-2.22
ENSMUSG00000017639	Rab11fip4	RAB11 family interacting protein 4 (class II)	-2.22
ENSMUSG00000025352	Gdf11	growth differentiation factor 11	-2.22
ENSMUSG00000019986	Ahi1	Abelson helper integration site 1	-2.22
ENSMUSG00000035863	Palm	paralemmin	-2.22
ENSMUSG00000011751	Sptbn4	spectrin beta, non-erythrocytic 4	-2.22
ENSMUSG00000037259	Dzank1	double zinc ribbon and ankyrin repeat domains 1	-2.22
ENSMUSG00000020788	Atp2a3	ATPase, Ca++ transporting, ubiquitous	-2.23
ENSMUSG00000026828	Galnt5	polypeptide N-acetylgalactosaminyltransferase 5	-2.23
ENSMUSG00000050395	Tnfsf15	tumor necrosis factor (ligand) superfamily, member 15	-2.23
ENSMUSG00000039236	Isg20	interferon-stimulated protein	-2.23
ENSMUSG00000000197	Nalcn	sodium leak channel, non-selective	-2.23

ENSMUSG00000036745	Ttll7	tubulin tyrosine ligase-like family, member 7	-2.24
ENSMUSG00000025584	Pde8a	phosphodiesterase 8A	-2.24
ENSMUSG00000053980	Gm9930	predicted gene 9930	-2.24
ENSMUSG00000089995	Gm15716	predicted gene 15716	-2.24
ENSMUSG00000028637	Ccdc30	coiled-coil domain containing 30	-2.24
ENSMUSG00000097927	Gm6999	predicted gene 6999	-2.24
ENSMUSG00000029700	Slc13a1	solute carrier family 13 (sodium/sulfate symporters), member 1	-2.25
ENSMUSG00000049044	Rapgef4	Rap guanine nucleotide exchange factor (GEF) 4	-2.25
ENSMUSG00000045763	Basp1	brain abundant, membrane attached signal protein 1	-2.25
ENSMUSG00000051335	Gfod1	glucose-fructose oxidoreductase domain containing 1	-2.26
ENSMUSG00000032548	Slco2a1	solute carrier organic anion transporter family, member 2a1	-2.26
ENSMUSG00000027514	Zbp1	Z-DNA binding protein 1	-2.26
ENSMUSG00000021986	Amer2	APC membrane recruitment 2	-2.26
ENSMUSG00000048965	Mrgpre	MAS-related GPR, member E	-2.26
ENSMUSG00000040061	Plcb2	phospholipase C, beta 2	-2.26
ENSMUSG00000044468	Tent5c	terminal nucleotidyltransferase 5C	-2.27
ENSMUSG00000075010	AW112010	expressed sequence AW112010	-2.27
ENSMUSG00000050321	Neto1	neuropilin (NRP) and tolloid (TLL)-like 1	-2.27
ENSMUSG00000022208	Jph4	junctophilin 4	-2.27
ENSMUSG00000084890	A830036E02Rik	RIKEN cDNA A830036E02 gene	-2.27
ENSMUSG00000109561	Ankrd31	ankyrin repeat domain 31	-2.27
ENSMUSG00000029102	Hgfac	hepatocyte growth factor activator	-2.28
ENSMUSG00000028270	Gbp2	guanylate binding protein 2	-2.28
ENSMUSG00000018169	Mfng	MFNG O-fucosylpeptide 3-beta-N-acetylglucosaminyltransferase	-2.28
ENSMUSG00000037217	Syn1	synapsin I	-2.28
ENSMUSG00000045062	Pcdhb7	protocadherin beta 7	-2.28
ENSMUSG00000041729	Coro2b	coronin, actin binding protein, 2B	-2.28
ENSMUSG00000111323	Gm48129	predicted gene, 48129	-2.28
ENSMUSG00000060843	Ctnna3	catenin (cadherin associated protein), alpha 3	-2.28
ENSMUSG00000002565	Scin	scinderin	-2.29
ENSMUSG00000054434	Tmem120b	transmembrane protein 120B	-2.29
ENSMUSG00000103567	Pcdhga5	protocadherin gamma subfamily A, 5	-2.29
ENSMUSG00000034591	Slc41a2	solute carrier family 41, member 2	-2.30
ENSMUSG00000035274	Tpbg	trophoblast glycoprotein	-2.30
ENSMUSG00000039126	Prune2	prune homolog 2	-2.31
ENSMUSG00000063522	2010109I03Rik	RIKEN cDNA 2010109I03 gene	-2.31
ENSMUSG00000041268	Dmxl2	Dmx-like 2	-2.31
ENSMUSG00000023809	Rps6ka2	ribosomal protein S6 kinase, polypeptide 2	-2.31
ENSMUSG00000038143	Stox2	storkhead box 2	-2.31
ENSMUSG00000074437	Defa29	defensin, alpha, 29	-2.31
ENSMUSG00000030329	Pianp	PILR alpha associated neural protein	-2.31
ENSMUSG00000029348	Asphd2	aspartate beta-hydroxylase domain containing 2	-2.31
ENSMUSG00000040152	Thbs1	thrombospondin 1	-2.32
ENSMUSG00000051177	Plcb1	phospholipase C, beta 1	-2.32
ENSMUSG00000108688	Gm44985	predicted gene 44985	-2.32
ENSMUSG00000003436	Dll3	delta like canonical Notch ligand 3	-2.32
ENSMUSG00000042388	Dlgap3	DLG associated protein 3	-2.32
ENSMUSG00000036915	Kirrel2	kirre like nephrin family adhesion molecule 2	-2.32
ENSMUSG00000025551	Fgf14	fibroblast growth factor 14	-2.32
ENSMUSG00000099148	Gm3331	predicted gene 3331	-2.32
ENSMUSG00000020599	Rgs9	regulator of G-protein signaling 9	-2.33
ENSMUSG00000047867	Gimap6	GTPase, IMAP family member 6	-2.33
ENSMUSG00000078235	Fam43b	family with sequence similarity 43, member B	-2.33
ENSMUSG00000036492	Rnf39	ring finger protein 39	-2.34
ENSMUSG00000058618	AY761184	cDNA sequence AY761184	-2.34
ENSMUSG00000044646	Zbtb7c	zinc finger and BTB domain containing 7C	-2.34
ENSMUSG00000020940	1700023F06Rik	RIKEN cDNA 1700023F06 gene	-2.34
ENSMUSG00000033768	Nrxn2	neurexin II	-2.34
ENSMUSG00000020019	Ntn4	netrin 4	-2.35
ENSMUSG00000033287	Kctd17	potassium channel tetramerisation domain containing 17	-2.35
ENSMUSG00000045912	C2cd4c	C2 calcium-dependent domain containing 4C	-2.35
ENSMUSG00000032017	Grik4	glutamate receptor, ionotropic, kainate 4	-2.35
ENSMUSG00000034111	Tmed8	transmembrane p24 trafficking protein 8	-2.36
ENSMUSG00000013846	St3gal1	ST3 beta-galactoside alpha-2,3-sialyltransferase 1	-2.36
ENSMUSG00000042834	Nrep	neuronal regeneration related protein	-2.36
ENSMUSG00000102732	Gm37342	predicted gene, 37342	-2.36
ENSMUSG00000074625	Arhgap40	Rho GTPase activating protein 40	-2.36
ENSMUSG00000037016	Frem2	Fras1 related extracellular matrix protein 2	-2.36
ENSMUSG00000063239	Grm4	glutamate receptor, metabotropic 4	-2.36
ENSMUSG00000113811	Gm47882	predicted gene, 47882	-2.36
ENSMUSG00000046215	Rprml	reprimo-like	-2.36
ENSMUSG00000049858	Suox	sulfite oxidase	-2.37
ENSMUSG00000030406	Gipr	gastric inhibitory polypeptide receptor	-2.37

ENSMUSG00000102723	Gm37936	predicted gene, 37936	-2.37
ENSMUSG00000046668	Cxxc5	CXXC finger 5	-2.38
ENSMUSG00000052271	Bhlha15	basic helix-loop-helix family, member a15	-2.38
ENSMUSG00000063160	Numbl	numb-like	-2.38
ENSMUSG00000007950	Abhd8	abhydrolase domain containing 8	-2.38
ENSMUSG00000041794	Myrip	myosin VIIA and Rab interacting protein	-2.39
ENSMUSG00000073102	Drc1	dynein regulatory complex subunit 1	-2.39
ENSMUSG00000043020	Wdr63	WD repeat domain 63	-2.39
ENSMUSG00000060402	Chst8	carbohydrate (N-acetylgalactosamine 4-0) sulfotransferase 8	-2.40
ENSMUSG00000053580	Tanc2	tetratricopeptide repeat, ankyrin repeat and coiled-coil containing 2	-2.41
ENSMUSG00000057897	Camk2b	calcium/calmodulin-dependent protein kinase II, beta	-2.41
ENSMUSG00000036306	Lzts1	leucine zipper, putative tumor suppressor 1	-2.41
ENSMUSG00000112197	Gm47337	predicted gene, 47337	-2.41
ENSMUSG00000091890	A830073O21Rik	RIKEN cDNA A830073O21 gene	-2.41
ENSMUSG00000043461	Sptssb	serine palmitoyltransferase, small subunit B	-2.42
ENSMUSG00000030107	Usp18	ubiquitin specific peptidase 18	-2.42
ENSMUSG00000033200	Tpsg1	tryptase gamma 1	-2.42
ENSMUSG00000043760	Pkhd1	polycystic kidney and hepatic disease 1	-2.43
ENSMUSG00000037627	Rgs22	regulator of G-protein signalling 22	-2.43
ENSMUSG00000101126	Gm10538	predicted gene 10538	-2.43
ENSMUSG00000037664	Cdkn1c	cyclin-dependent kinase inhibitor 1C (P57)	-2.44
ENSMUSG00000017897	Eya2	EYA transcriptional coactivator and phosphatase 2	-2.44
ENSMUSG00000114985	1810028F09Rik	RIKEN cDNA 1810028F09 gene	-2.44
ENSMUSG00000024500	Ppp2r2b	protein phosphatase 2, regulatory subunit B, beta	-2.44
ENSMUSG00000042351	Grap2	GRB2-related adaptor protein 2	-2.44
ENSMUSG00000016763	Scube1	signal peptide, CUB domain, EGF-like 1	-2.45
ENSMUSG00000038594	Cep85l	centrosomal protein 85-like	-2.45
ENSMUSG00000070056	Mfhas1	malignant fibrous histiocytoma amplified sequence 1	-2.46
ENSMUSG00000086141	9030622O22Rik	RIKEN cDNA 9030622O22 gene	-2.46
ENSMUSG00000024238	Zeb1	zinc finger E-box binding homeobox 1	-2.46
ENSMUSG00000035864	Syt1	synaptotagmin I	-2.46
ENSMUSG00000039059	Hrh3	histamine receptor H3	-2.47
ENSMUSG00000039976	Tbc1d16	TBC1 domain family, member 16	-2.48
ENSMUSG00000020227	Irak3	interleukin-1 receptor-associated kinase 3	-2.48
ENSMUSG00000023009	Nckap5l	NCK-associated protein 5-like	-2.48
ENSMUSG00000028439	Fam219a	family with sequence similarity 219, member A	-2.48
ENSMUSG00000058740	Kcnt1	potassium channel, subfamily T, member 1	-2.48
ENSMUSG00000103001	A930005N03Rik	RIKEN cDNA A930005N03 gene	-2.48
ENSMUSG00000029166	Mapre3	microtubule-associated protein, RP/EB family, member 3	-2.50
ENSMUSG00000030717	Nupr1	nuclear protein transcription regulator 1	-2.50
ENSMUSG00000070337	Gpr179	G protein-coupled receptor 179	-2.50
ENSMUSG00000038319	Kcnh2	potassium voltage-gated channel, subfamily H (eag-related), member 2	-2.50
ENSMUSG00000004709	Cd244a	CD244 molecule A	-2.50
ENSMUSG00000000125	Wnt3	wingless-type MMTV integration site family, member 3	-2.51
ENSMUSG00000069227	Gprin1	G protein-regulated inducer of neurite outgrowth 1	-2.51
ENSMUSG00000048763	Hoxb3	homeobox B3	-2.51
ENSMUSG00000041889	Shisa4	shisa family member 4	-2.51
ENSMUSG00000026950	Neb	nebulin	-2.52
ENSMUSG00000020102	Slc16a7	solute carrier family 16 (monocarboxylic acid transporters), member 7	-2.52
ENSMUSG00000112433	Gm30122	predicted gene, 30122	-2.52
ENSMUSG00000019122	Ccl9	chemokine (C-C motif) ligand 9	-2.53
ENSMUSG00000080715	Gm5406	predicted gene 5406	-2.53
ENSMUSG00000103761	Gm37859	predicted gene, 37859	-2.53
ENSMUSG00000050824	Sstr5	somatostatin receptor 5	-2.53
ENSMUSG00000057182	Scn3a	sodium channel, voltage-gated, type III, alpha	-2.53
ENSMUSG00000024598	Fbn2	fibrillin 2	-2.53
ENSMUSG00000064413	Gm22245	predicted gene, 22245	-2.53
ENSMUSG00000039115	Itga9	integrin alpha 9	-2.54
ENSMUSG00000021013	Ttc8	tetratricopeptide repeat domain 8	-2.54
ENSMUSG00000038065	Mturn	maturin, neural progenitor differentiation regulator homolog (Xenopus)	-2.55
ENSMUSG00000057614	Gnai1	guanine nucleotide binding protein (G protein), alpha inhibiting 1	-2.55
ENSMUSG00000040183	Ankrd6	ankyrin repeat domain 6	-2.55
ENSMUSG00000029134	Plb1	phospholipase B1	-2.55
ENSMUSG00000106048	Gm42444	predicted gene 42444	-2.56
ENSMUSG00000036699	Zcchc12	zinc finger, CCHC domain containing 12	-2.56
ENSMUSG00000000031	H19	H19, imprinted maternally expressed transcript	-2.57
ENSMUSG00000074444	Defa30	defensin, alpha, 30	-2.57
ENSMUSG00000006575	Rundc3a	RUN domain containing 3A	-2.58
ENSMUSG00000021725	Parp8	poly (ADP-ribose) polymerase family, member 8	-2.58
ENSMUSG00000062296	Trank1	tetratricopeptide repeat and ankyrin repeat containing 1	-2.58
ENSMUSG00000039543	Cfap70	cilia and flagella associated protein 70	-2.58
ENSMUSG00000005873	Reep5	receptor accessory protein 5	-2.59
ENSMUSG00000050822	Slc29a4	solute carrier family 29 (nucleoside transporters), member 4	-2.60

ENSMUSG00000024451	Arap3	ArfGAP with RhoGAP domain, ankyrin repeat and PH domain 3	-2.60
ENSMUSG00000049420	Tmem200a	transmembrane protein 200A	-2.60
ENSMUSG00000039005	Tlr4	toll-like receptor 4	-2.60
ENSMUSG00000029445	Hpd	4-hydroxyphenylpyruvic acid dioxygenase	-2.61
ENSMUSG00000001333	Sync	syncoilin	-2.61
ENSMUSG00000078949	R3hdml	R3H domain containing-like	-2.61
ENSMUSG00000001281	Itgb7	integrin beta 7	-2.61
ENSMUSG00000026167	Wnt10a	wingless-type MMTV integration site family, member 10A	-2.61
ENSMUSG00000036960	Clca2	chloride channel accessory 2	-2.61
ENSMUSG00000015396	Cd83	CD83 antigen	-2.62
ENSMUSG00000032068	Plet1	placenta expressed transcript 1	-2.62
ENSMUSG00000040367	Lrrd1	leucine rich repeats and death domain containing 1	-2.62
ENSMUSG00000037610	Kcnmb2	potassium large conductance calcium-activated channel, subfamily M, beta member 2	-2.62
ENSMUSG00000001670	Tat	tyrosine aminotransferase	-2.63
ENSMUSG00000038370	Pcp4l1	Purkinje cell protein 4-like 1	-2.63
ENSMUSG00000069515	Lyz1	lysozyme 1	-2.63
ENSMUSG00000037754	Ppp1r16b	protein phosphatase 1, regulatory subunit 16B	-2.64
ENSMUSG00000037579	Kcnh3	potassium voltage-gated channel, subfamily H (eag-related), member 3	-2.64
ENSMUSG00000060070	Defa26	defensin, alpha, 26	-2.64
ENSMUSG00000020175	Rab36	RAB36, member RAS oncogene family	-2.64
ENSMUSG00000049047	Armcx3	armadillo repeat containing, X-linked 3	-2.65
ENSMUSG00000083718	Ccnb2-ps	cyclin B2, pseudogene	-2.65
ENSMUSG00000044312	Neurog3	neurogenin 3	-2.65
ENSMUSG00000100396	Gm29367	predicted gene 29367	-2.65
ENSMUSG00000031980	Agt	angiotensinogen (serpin peptidase inhibitor, clade A, member 8)	-2.66
ENSMUSG00000027223	Mapk8ip1	mitogen-activated protein kinase 8 interacting protein 1	-2.67
ENSMUSG00000029490	Mfsd7a	major facilitator superfamily domain containing 7A	-2.67
ENSMUSG00000034059	Ypel4	yippee like 4	-2.67
ENSMUSG00000006651	Aplp1	amyloid beta (A4) precursor-like protein 1	-2.68
ENSMUSG00000072235	Tuba1a	tubulin, alpha 1A	-2.69
ENSMUSG00000040495	Chrm4	cholinergic receptor, muscarinic 4	-2.69
ENSMUSG00000114245	Gm38655	predicted gene, 38655	-2.69
ENSMUSG00000046056	Sbsn	suprabasin	-2.70
ENSMUSG00000064043	Trerf1	transcriptional regulating factor 1	-2.70
ENSMUSG00000005883	Spo11	SPO11 meiotic protein covalently bound to DSB	-2.70
ENSMUSG00000053182	Gm609	predicted gene 609	-2.71
ENSMUSG00000074441	Gm15292	predicted gene 15292	-2.71
ENSMUSG00000027376	Prom2	prominin 2	-2.71
ENSMUSG00000028078	Dclk2	doublecortin-like kinase 2	-2.72
ENSMUSG00000074981	Dcdc5	doublecortin domain containing 5	-2.72
ENSMUSG00000027520	Zdbf2	zinc finger, DBF-type containing 2	-2.73
ENSMUSG00000018340	Anxa6	annexin A6	-2.74
ENSMUSG00000036764	Dnajc12	DnaJ heat shock protein family (Hsp40) member C12	-2.74
ENSMUSG00000065956	Defa37	defensin, alpha, 37	-2.74
ENSMUSG00000025867	Cplx2	complexin 2	-2.75
ENSMUSG00000021730	Hcn1	hyperpolarization-activated, cyclic nucleotide-gated K+ 1	-2.75
ENSMUSG00000051079	Rgs13	regulator of G-protein signaling 13	-2.75
ENSMUSG00000028749	Pla2g2f	phospholipase A2, group IIF	-2.76
ENSMUSG00000025876	Unc5a	unc-5 netrin receptor A	-2.76
ENSMUSG00000066191	Anks6	ankyrin repeat and sterile alpha motif domain containing 6	-2.76
ENSMUSG00000021364	Elovl2	elongation of very long chain fatty acids (FEN1/Elo2, SUR4/Elo3, yeast)-like 2	-2.76
ENSMUSG00000015484	Fam163a	family with sequence similarity 163, member A	-2.76
ENSMUSG00000063903	Klk1	kallikrein 1	-2.77
ENSMUSG00000020668	Kif3c	kinesin family member 3C	-2.77
ENSMUSG00000057315	Arhgap24	Rho GTPase activating protein 24	-2.77
ENSMUSG00000062591	Tubb4a	tubulin, beta 4A class IVA	-2.78
ENSMUSG00000074447	Defa21	defensin, alpha, 21	-2.78
ENSMUSG00000109305	1810010D01Rik	RIKEN cDNA 1810010D01 gene	-2.78
ENSMUSG00000021069	Pygl	liver glycogen phosphorylase	-2.78
ENSMUSG00000025091	Pnliprp2	pancreatic lipase-related protein 2	-2.79
ENSMUSG00000040412	5330417C22Rik	RIKEN cDNA 5330417C22 gene	-2.80
ENSMUSG00000031981	Capn9	calpain 9	-2.81
ENSMUSG00000109780	Gm45447	predicted gene 45447	-2.81
ENSMUSG00000024743	Syt7	synaptotagmin VII	-2.82
ENSMUSG00000069516	Lyz2	lysozyme 2	-2.82
ENSMUSG00000035896	Rnase1	ribonuclease, RNase A family, 1 (pancreatic)	-2.83
ENSMUSG00000038209	Itln1	intelectin 1 (galactofuranose binding)	-2.83
ENSMUSG00000074446	Defa23	defensin, alpha, 23	-2.83
ENSMUSG00000029778	Adcyap1r1	adenylate cyclase activating polypeptide 1 receptor 1	-2.83
ENSMUSG00000038599	Capn8	calpain 8	-2.84
ENSMUSG00000025969	Nrp2	neuropilin 2	-2.84
ENSMUSG00000037386	Rims2	regulating synaptic membrane exocytosis 2	-2.84
ENSMUSG00000028415	Spink4	serine peptidase inhibitor, Kazal type 4	-2.85

ENSMUSG00000040121	Rep15	RAB15 effector protein	-2.86
ENSMUSG00000048022	Tmem229a	transmembrane protein 229A	-2.86
ENSMUSG00000071178	Serpina1b	serine (or cysteine) preptidase inhibitor, clade A, member 1B	-2.86
ENSMUSG00000013921	Clip3	CAP-GLY domain containing linker protein 3	-2.86
ENSMUSG00000075517	Cyp2d37-ps	cytochrome P450, family 2, subfamily d, polypeptide 37, pseudogene	-2.86
ENSMUSG00000028626	Col9a2	collagen, type IX, alpha 2	-2.87
ENSMUSG00000099025	Gm27162	predicted gene 27162	-2.87
ENSMUSG00000036832	Lpar3	lysophosphatidic acid receptor 3	-2.87
ENSMUSG00000026546	Cfap45	cilia and flagella associated protein 45	-2.87
ENSMUSG00000031377	Bmx	BMX non-receptor tyrosine kinase	-2.88
ENSMUSG00000026728	Vim	vimentin	-2.88
ENSMUSG00000072294	Klf12	Kruppel-like factor 12	-2.88
ENSMUSG00000064213	Defa24	defensin, alpha, 24	-2.88
ENSMUSG00000042873	Lhfpl4	lipoma HMGIC fusion partner-like protein 4	-2.88
ENSMUSG00000051242	Pcdhb9	protocadherin beta 9	-2.88
ENSMUSG00000060208	Defa17	defensin, alpha, 17	-2.89
ENSMUSG00000082443	Gm12604	predicted gene 12604	-2.89
ENSMUSG00000044933	Sstr3	somatostatin receptor 3	-2.90
ENSMUSG00000039013	Siglecf	sialic acid binding Ig-like lectin F	-2.90
ENSMUSG00000026959	Grin1	glutamate receptor, ionotropic, NMDA1 (zeta 1)	-2.91
ENSMUSG00000041000	Trim62	tripartite motif-containing 62	-2.92
ENSMUSG00000030110	Ret	ret proto-oncogene	-2.92
ENSMUSG00000034107	Ano7	anoctamin 7	-2.92
ENSMUSG00000015968	Cacna1d	calcium channel, voltage-dependent, L type, alpha 1D subunit	-2.93
ENSMUSG00000018593	Sparc	secreted acidic cysteine rich glycoprotein	-2.94
ENSMUSG00000111086	Gm48671	predicted gene, 48671	-2.94
ENSMUSG00000068617	Efcab1	EF-hand calcium binding domain 1	-2.95
ENSMUSG00000032502	Stac	src homology three (SH3) and cysteine rich domain	-2.96
ENSMUSG00000032015	Pou2f3	POU domain, class 2, transcription factor 3	-2.96
ENSMUSG00000082211	Defa27	defensin, alpha, 27	-2.96
ENSMUSG00000044548	Dact1	dishevelled-binding antagonist of beta-catenin 1	-2.97
ENSMUSG00000033220	Rac2	Rac family small GTPase 2	-2.97
ENSMUSG00000024330	Col11a2	collagen, type XI, alpha 2	-2.98
ENSMUSG00000030772	Dkk3	dickkopf WNT signaling pathway inhibitor 3	-2.98
ENSMUSG00000002900	Lamb1	laminin B1	-2.98
ENSMUSG00000008932	Slc1a7	solute carrier family 1 (glutamate transporter), member 7	-2.98
ENSMUSG00000026870	Cutal	cutA divalent cation tolerance homolog-like	-2.99
ENSMUSG00000026482	Rgl1	ral guanine nucleotide dissociation stimulator,-like 1	-2.99
ENSMUSG00000019775	Rgs17	regulator of G-protein signaling 17	-2.99
ENSMUSG00000098557	Kctd12	potassium channel tetramerisation domain containing 12	-3.00
ENSMUSG00000055963	Triqk	triple QxxK/R motif containing	-3.00
ENSMUSG00000051486	Pcdhb11	protocadherin beta 11	-3.00
ENSMUSG00000051527	Usp29	ubiquitin specific peptidase 29	-3.00
ENSMUSG00000020901	Pik3r5	phosphoinositide-3-kinase regulatory subunit 5	-3.01
ENSMUSG00000093805	Gal3st2b	galactose-3-O-sulfotransferase 2B	-3.01
ENSMUSG00000000394	Gcg	glucagon	-3.01
ENSMUSG00000032202	Rab27a	RAB27A, member RAS oncogene family	-3.02
ENSMUSG00000040723	Rcsd1	RCSD domain containing 1	-3.02
ENSMUSG00000073400	Trim10	tripartite motif-containing 10	-3.02
ENSMUSG00000033174	Mgll	monoglyceride lipase	-3.02
ENSMUSG00000036412	Arsi	arylsulfatase i	-3.02
ENSMUSG00000023032	Slc4a8	solute carrier family 4 (anion exchanger), member 8	-3.03
ENSMUSG00000060615	Ang4	angiogenin, ribonuclease A family, member 4	-3.03
ENSMUSG00000032690	Oas2	2'-5' oligoadenylate synthetase 2	-3.03
ENSMUSG00000086067	Gm16183	predicted gene 16183	-3.03
ENSMUSG00000023247	Guca2a	guanylate cyclase activator 2a (guanylin)	-3.04
ENSMUSG00000022211	Carmil3	capping protein regulator and myosin 1 linker 3	-3.04
ENSMUSG00000036533	Cdc42ep3	CDC42 effector protein (Rho GTPase binding) 3	-3.04
ENSMUSG00000045672	Col27a1	collagen, type XXVII, alpha 1	-3.04
ENSMUSG00000020121	Srgap1	SLIT-ROBO Rho GTPase activating protein 1	-3.05
ENSMUSG00000047181	Samd14	sterile alpha motif domain containing 14	-3.05
ENSMUSG00000072812	Ahnak2	AHNAK nucleoprotein 2	-3.05
ENSMUSG00000074443	Defa22	defensin, alpha, 22	-3.05
ENSMUSG00000020101	Vsir	V-set immunoregulatory receptor	-3.05
ENSMUSG00000040978	Gm11992	predicted gene 11992	-3.06
ENSMUSG00000020717	Pecam1	platelet/endothelial cell adhesion molecule 1	-3.06
ENSMUSG00000025964	Adam23	a disintegrin and metallopeptidase domain 23	-3.06
ENSMUSG00000058589	Anks1b	ankyrin repeat and sterile alpha motif domain containing 1B	-3.06
ENSMUSG00000001120	Pcbp3	poly(rC) binding protein 3	-3.07
ENSMUSG00000090812	Samd15	sterile alpha motif domain containing 15	-3.07
ENSMUSG00000079442	St6galnac4	ST6 (alpha-N-acetyl-neuraminyl-2,3-beta-galactosyl-1,3)-N-acetylgalactosaminide alpha-2,6-sialyltra	-3.08
ENSMUSG00000027661	Slc2a10	solute carrier family 2 (facilitated glucose transporter), member 10	-3.08
ENSMUSG00000047642	D930020B18Rik	RIKEN cDNA D930020B18 gene	-3.08

ENSMUSG00000025075	Habp2	hyaluronic acid binding protein 2	-3.09
ENSMUSG00000006435	Neurl1a	neuralized E3 ubiquitin protein ligase 1A	-3.09
ENSMUSG00000026288	Inpp5d	inositol polyphosphate-5-phosphatase D	-3.09
ENSMUSG00000034818	Celf5	CUGBP, Elav-like family member 5	-3.09
ENSMUSG00000033152	Podxl2	podocalyxin-like 2	-3.09
ENSMUSG00000050359	Sprr1a	small proline-rich protein 1A	-3.09
ENSMUSG00000079015	Serpina1c	serine (or cysteine) peptidase inhibitor, clade A, member 1C	-3.09
ENSMUSG00000029563	Foxp2	forkhead box P2	-3.09
ENSMUSG00000024225	Clps	colipase, pancreatic	-3.11
ENSMUSG00000044022	Pcdhb21	protocadherin beta 21	-3.11
ENSMUSG00000051251	Nhlh1	nescient helix loop helix 1	-3.11
ENSMUSG00000001802	Lrp3	low density lipoprotein receptor-related protein 3	-3.12
ENSMUSG00000018800	Abca5	ATP-binding cassette, sub-family A (ABC1), member 5	-3.12
ENSMUSG00000021071	Trim9	tripartite motif-containing 9	-3.12
ENSMUSG00000063142	Kcnma1	potassium large conductance calcium-activated channel, subfamily M, alpha member 1	-3.13
ENSMUSG00000043391	2510009E07Rik	RIKEN cDNA 2510009E07 gene	-3.13
ENSMUSG00000034353	Ramp1	receptor (calcitonin) activity modifying protein 1	-3.13
ENSMUSG00000018470	Kcnab3	potassium voltage-gated channel, shaker-related subfamily, beta member 3	-3.13
ENSMUSG00000103793	Pcdhga6	protocadherin gamma subfamily A, 6	-3.13
ENSMUSG00000059898	Dsc3	desmocollin 3	-3.14
ENSMUSG00000071550	Cfap44	cilia and flagella associated protein 44	-3.14
ENSMUSG00000046922	Gpr6	G protein-coupled receptor 6	-3.14
ENSMUSG00000022148	Fyb	FYN binding protein	-3.16
ENSMUSG00000052889	Prkcb	protein kinase C, beta	-3.16
ENSMUSG00000051043	Gprc5c	G protein-coupled receptor, family C, group 5, member C	-3.17
ENSMUSG00000061845	Defa35	defensin, alpha, 35	-3.17
ENSMUSG00000036062	Phf24	PHD finger protein 24	-3.17
ENSMUSG00000035357	Pdzrn3	PDZ domain containing RING finger 3	-3.18
ENSMUSG00000028600	Podn	podocan	-3.18
ENSMUSG00000063206	Defa34	defensin, alpha, 34	-3.18
ENSMUSG00000009246	Trpm5	transient receptor potential cation channel, subfamily M, member 5	-3.19
ENSMUSG00000021614	Vcan	versican	-3.19
ENSMUSG00000095328	Defa-ps6	defensin, alpha, pseudogene 6	-3.19
ENSMUSG00000008193	Spib	Spi-B transcription factor (Spi-1/PU.1 related)	-3.19
ENSMUSG00000072875	Gpr27	G protein-coupled receptor 27	-3.19
ENSMUSG00000094305	Scgb2b20	secretoglobin, family 2B, member 20	-3.19
ENSMUSG00000087211	Lhx1os	LIM homeobox 1, opposite strand	-3.19
ENSMUSG00000013523	Bcas1	breast carcinoma amplified sequence 1	-3.20
ENSMUSG00000042115	Klhdc8a	kelch domain containing 8A	-3.20
ENSMUSG00000032625	Thsd7a	thrombospondin, type I, domain containing 7A	-3.20
ENSMUSG00000019232	Etnppl	ethanolamine phosphate phospholyase	-3.21
ENSMUSG00000023216	Epb42	erythrocyte membrane protein band 4.2	-3.22
ENSMUSG00000025855	Prkar1b	protein kinase, cAMP dependent regulatory, type I beta	-3.22
ENSMUSG00000041420	Meis3	Meis homeobox 3	-3.22
ENSMUSG00000069814	Ccdc92b	coiled-coil domain containing 92B	-3.22
ENSMUSG00000079180	Mptx2	mucosal pentraxin 2	-3.22
ENSMUSG00000006546	Cryba2	crystallin, beta A2	-3.22
ENSMUSG00000037712	Fermt2	fermitin family member 2	-3.23
ENSMUSG00000032839	Trpc1	transient receptor potential cation channel, subfamily C, member 1	-3.24
ENSMUSG00000081303	Gm16011	predicted gene 16011	-3.25
ENSMUSG00000036913	Trim67	tripartite motif-containing 67	-3.25
ENSMUSG00000056069	Otulinl	OTU deubiquitinase with linear linkage specificity like	-3.25
ENSMUSG00000074274	D930028M14Rik	RIKEN cDNA D930028M14 gene	-3.25
ENSMUSG00000022441	Efcab6	EF-hand calcium binding domain 6	-3.26
ENSMUSG00000031760	Mt3	metallothionein 3	-3.27
ENSMUSG00000043088	Il17re	interleukin 17 receptor E	-3.27
ENSMUSG00000018339	Gpx3	glutathione peroxidase 3	-3.27
ENSMUSG00000033389	Arhgap44	Rho GTPase activating protein 44	-3.28
ENSMUSG00000045689	Pcdhb4	protocadherin beta 4	-3.28
ENSMUSG00000074440	Defa3	defensin, alpha, 3	-3.29
ENSMUSG00000029769	Ccdc136	coiled-coil domain containing 136	-3.29
ENSMUSG00000030411	Nova2	neuro-oncological ventral antigen 2	-3.29
ENSMUSG00000036480	Prss56	protease, serine 56	-3.29
ENSMUSG00000102625	Gm37672	predicted gene, 37672	-3.29
ENSMUSG00000036330	Slc18a1	solute carrier family 18 (vesicular monoamine), member 1	-3.30
ENSMUSG00000034675	Dbn1	drebrin 1	-3.30
ENSMUSG00000047730	Fcgbp	Fc fragment of IgG binding protein	-3.30
ENSMUSG00000004707	Ly9	lymphocyte antigen 9	-3.30
ENSMUSG00000038555	Reep2	receptor accessory protein 2	-3.30
ENSMUSG00000030074	Gxylt2	glucoside xylosyltransferase 2	-3.30
ENSMUSG00000032473	Cldn18	claudin 18	-3.30
ENSMUSG00000026360	Rgs2	regulator of G-protein signaling 2	-3.31
ENSMUSG00000069255	Dusp22	dual specificity phosphatase 22	-3.31

ENSMUSG00000023033	Scn8a	sodium channel, voltage-gated, type VIII, alpha	-3.32
ENSMUSG00000032936	Camkv	CaM kinase-like vesicle-associated	-3.33
ENSMUSG00000096295	Defa2	defensin, alpha, 2	-3.33
ENSMUSG00000044043	Pcdhb14	protocadherin beta 14	-3.33
ENSMUSG00000054169	Ceacam10	carcinoembryonic antigen-related cell adhesion molecule 10	-3.34
ENSMUSG00000043857	Mgat5b	mannoside acetylglucosaminyltransferase 5, isoenzyme B	-3.34
ENSMUSG00000086970	Bcas1os1	breast carcinoma amplified sequence 1, opposite strand 1	-3.34
ENSMUSG00000021539	Lect2	leukocyte cell-derived chemotaxin 2	-3.35
ENSMUSG00000027570	Col9a3	collagen, type IX, alpha 3	-3.35
ENSMUSG00000018862	Otop3	otopetrin 3	-3.35
ENSMUSG00000110394	Gm18991	predicted gene, 18991	-3.35
ENSMUSG00000024030	Abcg1	ATP binding cassette subfamily G member 1	-3.37
ENSMUSG00000038255	Neurod2	neurogenic differentiation 2	-3.37
ENSMUSG00000101930	Gm5441	predicted gene 5441	-3.37
ENSMUSG00000053141	Ptprt	protein tyrosine phosphatase, receptor type, T	-3.38
ENSMUSG00000025515	Muc2	mucin 2	-3.39
ENSMUSG00000030616	Sytl2	synaptotagmin-like 2	-3.39
ENSMUSG00000052942	Glis3	GLIS family zinc finger 3	-3.39
ENSMUSG00000030401	Rtn2	reticulon 2 (Z-band associated protein)	-3.39
ENSMUSG00000035246	Pcyt1b	phosphate cytidylyltransferase 1, choline, beta isoform	-3.40
ENSMUSG00000005503	Evx1	even-skipped homeobox 1	-3.40
ENSMUSG00000020848	Doc2b	double C2, beta	-3.40
ENSMUSG00000047238	Mageh1	melanoma antigen, family H, 1	-3.40
ENSMUSG00000028356	Ambp	alpha 1 microglobulin/bikunin	-3.40
ENSMUSG00000015053	Gata2	GATA binding protein 2	-3.40
ENSMUSG00000030428	Ttyh1	tweety family member 1	-3.41
ENSMUSG00000052212	Cd177	CD177 antigen	-3.42
ENSMUSG00000054453	Sytl5	synaptotagmin-like 5	-3.42
ENSMUSG00000029298	Gbp9	guanylate-binding protein 9	-3.42
ENSMUSG00000034336	Ina	internexin neuronal intermediate filament protein, alpha	-3.42
ENSMUSG00000024029	Tff3	trefoil factor 3, intestinal	-3.43
ENSMUSG00000044017	Adgrd1	adhesion G protein-coupled receptor D1	-3.43
ENSMUSG00000100182	1810006J02Rik	RIKEN cDNA 1810006J02 gene	-3.43
ENSMUSG00000091735	Gpr62	G protein-coupled receptor 62	-3.44
ENSMUSG00000019846	Lama4	laminin, alpha 4	-3.45
ENSMUSG00000031374	Zfp92	zinc finger protein 92	-3.46
ENSMUSG00000026443	Lrrn2	leucine rich repeat protein 2, neuronal	-3.46
ENSMUSG00000029193	Cckar	cholecystokinin A receptor	-3.46
ENSMUSG00000051314	Ffar2	free fatty acid receptor 2	-3.47
ENSMUSG00000051111	Sv2c	synaptic vesicle glycoprotein 2c	-3.47
ENSMUSG00000104149	Gm37138	predicted gene, 37138	-3.49
ENSMUSG00000035967	Ints6l	integrator complex subunit 6 like	-3.50
ENSMUSG00000040170	Fmo2	flavin containing monooxygenase 2	-3.50
ENSMUSG00000050473	Slc35d3	solute carrier family 35, member D3	-3.50
ENSMUSG00000055407	Map6	microtubule-associated protein 6	-3.51
ENSMUSG00000057335	Cep170	centrosomal protein 170	-3.52
ENSMUSG00000048347	Pcdhb18	protocadherin beta 18	-3.52
ENSMUSG00000041078	Grid1	glutamate receptor, ionotropic, delta 1	-3.52
ENSMUSG00000034656	Cacna1a	calcium channel, voltage-dependent, P/Q type, alpha 1A subunit	-3.53
ENSMUSG00000020601	Trib2	tribbles pseudokinase 2	-3.53
ENSMUSG00000042766	Trim46	tripartite motif-containing 46	-3.53
ENSMUSG00000040276	Pacsin1	protein kinase C and casein kinase substrate in neurons 1	-3.54
ENSMUSG00000044988	Ucn3	urocortin 3	-3.54
ENSMUSG00000097587	4930578M01Rik	RIKEN cDNA 4930578M01 gene	-3.55
ENSMUSG00000042179	Pnliprp1	pancreatic lipase related protein 1	-3.56
ENSMUSG00000048078	Tenm4	teneurin transmembrane protein 4	-3.58
ENSMUSG00000044156	Hepacam2	HEPACAM family member 2	-3.58
ENSMUSG00000053545	6430503K07Rik	RIKEN cDNA 6430503K07 gene	-3.58
ENSMUSG00000038349	Plcl1	phospholipase C-like 1	-3.58
ENSMUSG00000036395	Glb1l2	galactosidase, beta 1-like 2	-3.58
ENSMUSG00000041180	Hectd2	HECT domain E3 ubiquitin protein ligase 2	-3.58
ENSMUSG00000024548	Setbp1	SET binding protein 1	-3.59
ENSMUSG00000046204	Pnma2	paraneoplastic antigen MA2	-3.59
ENSMUSG00000041633	Kctd12b	potassium channel tetramerisation domain containing 12b	-3.59
ENSMUSG00000047250	Ptgs1	prostaglandin-endoperoxide synthase 1	-3.60
ENSMUSG00000052631	Sh2d6	SH2 domain containing 6	-3.61
ENSMUSG00000040009	Gnaz	guanine nucleotide binding protein, alpha z subunit	-3.61
ENSMUSG00000021904	Sema3g	sema domain, immunoglobulin domain (Ig), short basic domain, secreted, (semaphorin) 3G	-3.61
ENSMUSG00000035459	Stab2	stabilin 2	-3.61
ENSMUSG00000020703	5530401A14Rik	RIKEN cDNA 5530401A14 gene	-3.62
ENSMUSG00000085247	4930545L23Rik	RIKEN cDNA 4930545L23 gene	-3.63
ENSMUSG00000052087	Rgs14	regulator of G-protein signaling 14	-3.64
ENSMUSG00000025810	Nrp1	neuropilin 1	-3.64

ENSMUSG00000039037	St6galnac5	ST6 (alpha-N-acetyl-neuraminyl-2,3-beta-galactosyl-1,3)-N-acetylgalactosaminide alpha-2,6-sialyltra	-3.64
ENSMUSG00000003283	Hck	hemopoietic cell kinase	-3.64
ENSMUSG00000066705	Fxyd6	FXYD domain-containing ion transport regulator 6	-3.65
ENSMUSG00000056812	St8sia3	ST8 alpha-N-acetyl-neuraminide alpha-2,8-sialyltransferase 3	-3.66
ENSMUSG00000064125	Prr36	proline rich 36	-3.67
ENSMUSG00000086126	Evx1os	even skipped homeotic gene 1, opposite strand	-3.67
ENSMUSG00000105271	Gm42875	predicted gene 42875	-3.67
ENSMUSG00000021256	Vash1	vasohibin 1	-3.68
ENSMUSG00000021763	BC067074	cDNA sequence BC067074	-3.68
ENSMUSG00000086029	Pax6os1	paired box 6 opposite strand 1	-3.68
ENSMUSG00000032826	Ank2	ankyrin 2, brain	-3.69
ENSMUSG00000037239	Spred3	sprouty-related, EVH1 domain containing 3	-3.70
ENSMUSG00000062861	Zfp28	zinc finger protein 28	-3.70
ENSMUSG00000055430	Nap1l5	nucleosome assembly protein 1-like 5	-3.71
ENSMUSG00000029005	Draxin	dorsal inhibitory axon guidance protein	-3.71
ENSMUSG00000072849	Serpina1e	serine (or cysteine) peptidase inhibitor, clade A, member 1E	-3.71
ENSMUSG00000032852	Rspo4	R-spondin 4	-3.72
ENSMUSG00000030518	Fam189a1	family with sequence similarity 189, member A1	-3.72
ENSMUSG00000020340	Cyfip2	cytoplasmic FMR1 interacting protein 2	-3.73
ENSMUSG00000024013	Fgd2	FYVE, RhoGEF and PH domain containing 2	-3.74
ENSMUSG00000038224	Serpinf2	serine (or cysteine) peptidase inhibitor, clade F, member 2	-3.74
ENSMUSG00000040852	Plekhh2	pleckstrin homology domain containing, family H (with MyTH4 domain) member 2	-3.74
ENSMUSG00000023473	Celsr3	cadherin, EGF LAG seven-pass G-type receptor 3	-3.75
ENSMUSG00000032064	Dixdc1	DIX domain containing 1	-3.75
ENSMUSG00000002578	Ikzf4	IKAROS family zinc finger 4	-3.75
ENSMUSG00000040563	Plppr2	phospholipid phosphatase related 2	-3.75
ENSMUSG00000034799	Unc13a	unc-13 homolog A	-3.76
ENSMUSG00000095813	Defa-ps1	defensin, alpha, pseudogene 1	-3.76
ENSMUSG00000022054	Nefm	neurofilament, medium polypeptide	-3.77
ENSMUSG00000057914	Cacnb2	calcium channel, voltage-dependent, beta 2 subunit	-3.77
ENSMUSG00000038486	Sv2a	synaptic vesicle glycoprotein 2 a	-3.78
ENSMUSG00000078776	9530053A07Rik	RIKEN cDNA 9530053A07 gene	-3.79
ENSMUSG00000004347	Pde1c	phosphodiesterase 1C	-3.79
ENSMUSG00000079481	Nhsl2	NHS-like 2	-3.79
ENSMUSG00000055567	Unc80	unc-80, NALCN activator	-3.79
ENSMUSG00000025738	Fbxl16	F-box and leucine-rich repeat protein 16	-3.79
ENSMUSG00000028360	Slc44a5	solute carrier family 44, member 5	-3.79
ENSMUSG00000015222	Map2	microtubule-associated protein 2	-3.80
ENSMUSG00000030263	Lrmp	lymphoid-restricted membrane protein	-3.80
ENSMUSG00000097767	Miat	myocardial infarction associated transcript (non-protein coding)	-3.80
ENSMUSG00000026072	Il1r1	interleukin 1 receptor, type I	-3.80
ENSMUSG00000027254	Map1a	microtubule-associated protein 1 A	-3.81
ENSMUSG00000049605	Olfr418	olfactory receptor 418	-3.81
ENSMUSG00000066176	Gm12511	predicted gene 12511	-3.81
ENSMUSG00000023913	Pla2g7	phospholipase A2, group VII (platelet-activating factor acetylhydrolase, plasma)	-3.81
ENSMUSG00000102581	Gm37443	predicted gene, 37443	-3.82
ENSMUSG00000034771	Tle2	transducin-like enhancer of split 2	-3.83
ENSMUSG00000015599	Ttbk1	tau tubulin kinase 1	-3.83
ENSMUSG00000030302	Atp2b2	ATPase, Ca++ transporting, plasma membrane 2	-3.83
ENSMUSG00000068154	Insm1	insulinoma-associated 1	-3.85
ENSMUSG00000094662	Defa36	defensin, alpha, 36	-3.85
ENSMUSG00000021587	Pcsk1	proprotein convertase subtilisin/kexin type 1	-3.86
ENSMUSG00000063919	Srrm4	serine/arginine repetitive matrix 4	-3.86
ENSMUSG00000112234	Gm48427	predicted gene, 48427	-3.86
ENSMUSG00000052951	C130021I20Rik	Riken cDNA C130021I20 gene	-3.86
ENSMUSG00000047507	Baiap3	BAI1-associated protein 3	-3.87
ENSMUSG00000027674	Pex5l	peroxisomal biogenesis factor 5-like	-3.87
ENSMUSG00000040146	Rgl3	ral guanine nucleotide dissociation stimulator-like 3	-3.88
ENSMUSG00000020090	Npffr1	neuropeptide FF receptor 1	-3.88
ENSMUSG00000022438	Parvb	parvin, beta	-3.89
ENSMUSG00000111443	Gm46123	predicted gene, 46123	-3.89
ENSMUSG00000034958	Atcay	ataxia, cerebellar, Cayman type	-3.89
ENSMUSG00000032035	Ets1	E26 avian leukemia oncogene 1, 5' domain	-3.90
ENSMUSG00000075256	Cerkl	ceramide kinase-like	-3.90
ENSMUSG00000037106	Fer1l6	fer-1-like 6 (C. elegans)	-3.91
ENSMUSG00000056004	9330182L06Rik	RIKEN cDNA 9330182L06 gene	-3.91
ENSMUSG00000009628	Tex15	testis expressed gene 15	-3.92
ENSMUSG00000021536	Adcy2	adenylate cyclase 2	-3.92
ENSMUSG00000058420	Syt17	synaptotagmin XVII	-3.92
ENSMUSG00000040035	Disp2	dispatched RND tramsporter family member 2	-3.94
ENSMUSG00000038665	Dgki	diacylglycerol kinase, iota	-3.94
ENSMUSG00000050994	Adgb	androglobin	-3.94
ENSMUSG00000027048	Abcb11	ATP-binding cassette, sub-family B (MDR/TAP), member 11	-3.94

ENSMUSG00000112596	Gm48804	predicted gene, 48804	-3.95
ENSMUSG00000028469	Npr2	natriuretic peptide receptor 2	-3.95
ENSMUSG00000070498	Tmem132b	transmembrane protein 132B	-3.95
ENSMUSG00000049122	Frmd3	FERM domain containing 3	-3.96
ENSMUSG00000102937	Gm38116	predicted gene, 38116	-3.96
ENSMUSG00000046460	Sh2d7	SH2 domain containing 7	-3.96
ENSMUSG00000000632	Sez6	seizure related gene 6	-3.97
ENSMUSG00000029706	Pax4	paired box 4	-3.98
ENSMUSG00000040136	Abcc8	ATP-binding cassette, sub-family C (CFTR/MRP), member 8	-3.99
ENSMUSG00000020811	Wscd1	WSC domain containing 1	-4.00
ENSMUSG00000096146	Kcnj11	potassium inwardly rectifying channel, subfamily J, member 11	-4.00
ENSMUSG00000027876	Reg4	regenerating islet-derived family, member 4	-4.01
ENSMUSG00000039809	Gabbr2	gamma-aminobutyric acid (GABA) B receptor, 2	-4.02
ENSMUSG00000028255	Clca1	chloride channel accessory 1	-4.02
ENSMUSG00000048960	Prex2	phosphatidylinositol-3,4,5-trisphosphate-dependent Rac exchange factor 2	-4.02
ENSMUSG00000023906	Cldn6	claudin 6	-4.03
ENSMUSG00000028339	Col15a1	collagen, type XV, alpha 1	-4.03
ENSMUSG00000020333	Acsl6	acyl-CoA synthetase long-chain family member 6	-4.04
ENSMUSG00000019124	Scrn1	secernin 1	-4.04
ENSMUSG00000071226	Cecr2	CECR2, histone acetyl-lysine reader	-4.05
ENSMUSG00000010505	Myt1	myelin transcription factor 1	-4.05
ENSMUSG00000037035	Inhbb	inhibin beta-B	-4.06
ENSMUSG00000038248	Sobp	sine oculis binding protein	-4.06
ENSMUSG00000036766	Dner	delta/notch-like EGF repeat containing	-4.06
ENSMUSG00000043004	Gng2	guanine nucleotide binding protein (G protein), gamma 2	-4.07
ENSMUSG00000071719	Tmem28	transmembrane protein 28	-4.07
ENSMUSG00000074434	Defa28	defensin, alpha, 28	-4.07
ENSMUSG00000038916	Soga3	SOGA family member 3	-4.07
ENSMUSG00000044177	Wfikkn2	WAP, follistatin/kazal, immunoglobulin, kunitz and netrin domain containing 2	-4.08
ENSMUSG00000023236	Scg5	secretogranin V	-4.09
ENSMUSG00000038580	Sct	secretin	-4.09
ENSMUSG00000062760	Shisal1	shisa like 1	-4.09
ENSMUSG00000044288	Cnr1	cannabinoid receptor 1 (brain)	-4.09
ENSMUSG00000003411	Rab3b	RAB3B, member RAS oncogene family	-4.09
ENSMUSG00000039954	Stk32a	serine/threonine kinase 32A	-4.09
ENSMUSG00000047798	Cd300lf	CD300 molecule like family member F	-4.09
ENSMUSG00000096847	Tmem151b	transmembrane protein 151B	-4.10
ENSMUSG00000025582	Nptx1	neuronal pentraxin 1	-4.10
ENSMUSG00000022055	Nefl	neurofilament, light polypeptide	-4.10
ENSMUSG00000031748	Gnao1	guanine nucleotide binding protein, alpha O	-4.11
ENSMUSG00000049350	Zg16	zymogen granule protein 16	-4.11
ENSMUSG00000079116	Gm15293	predicted gene 15293	-4.11
ENSMUSG00000099907	Gm10421	predicted gene 10421	-4.11
ENSMUSG00000024897	Apba1	amyloid beta (A4) precursor protein binding, family A, member 1	-4.12
ENSMUSG00000027834	Serpini1	serine (or cysteine) peptidase inhibitor, clade I, member 1	-4.13
ENSMUSG00000040653	Ppp1r14c	protein phosphatase 1, regulatory inhibitor subunit 14C	-4.13
ENSMUSG00000020732	Rab37	RAB37, member RAS oncogene family	-4.13
ENSMUSG00000025020	Slit1	slit guidance ligand 1	-4.14
ENSMUSG00000034701	Neurod1	neurogenic differentiation 1	-4.14
ENSMUSG00000038692	Hoxb4	homeobox B4	-4.14
ENSMUSG00000027797	Dclk1	doublecortin-like kinase 1	-4.14
ENSMUSG00000106491	Gm42446	predicted gene 42446	-4.14
ENSMUSG00000029368	Alb	albumin	-4.14
ENSMUSG00000026442	Nfasc	neurofascin	-4.15
ENSMUSG00000114624	Gm48267	predicted gene, 48267	-4.15
ENSMUSG00000014602	Kif1a	kinesin family member 1A	-4.16
ENSMUSG00000105526	Gm43490	predicted gene 43490	-4.16
ENSMUSG00000086245	Gm16170	predicted gene 16170	-4.16
ENSMUSG00000036782	Klhl13	kelch-like 13	-4.16
ENSMUSG00000027434	Nkx2-2	NK2 homeobox 2	-4.18
ENSMUSG00000038765	Lmx1b	LIM homeobox transcription factor 1 beta	-4.18
ENSMUSG00000022456	37865	septin 3	-4.18
ENSMUSG00000029245	Epha5	Eph receptor A5	-4.18
ENSMUSG00000032377	Plscr4	phospholipid scramblase 4	-4.18
ENSMUSG00000032394	Igdcc3	immunoglobulin superfamily, DCC subclass, member 3	-4.18
ENSMUSG00000028528	Dnajc6	DnaJ heat shock protein family (Hsp40) member C6	-4.18
ENSMUSG00000010021	Kif19a	kinesin family member 19A	-4.20
ENSMUSG00000058975	Kcnc1	potassium voltage gated channel, Shaw-related subfamily, member 1	-4.20
ENSMUSG00000070802	Pnmal2	PNMA-like 2	-4.21
ENSMUSG00000022425	Enpp2	ectonucleotide pyrophosphatase/phosphodiesterase 2	-4.21
ENSMUSG00000033615	Cplx1	complexin 1	-4.21
ENSMUSG00000086999	Bcas1os2	breast carcinoma amplified sequence 1, opposite strand 2	-4.21
ENSMUSG00000046613	Vwa5b2	von Willebrand factor A domain containing 5B2	-4.22

ENSMUSG00000004961	Syt5	synaptotagmin V	-4.22
ENSMUSG00000040046	Tph1	tryptophan hydroxylase 1	-4.24
ENSMUSG00000026463	Atp2b4	ATPase, Ca++ transporting, plasma membrane 4	-4.25
ENSMUSG00000106139	Gm30648	predicted gene, 30648	-4.25
ENSMUSG00000019796	Lrp11	low density lipoprotein receptor-related protein 11	-4.26
ENSMUSG00000050640	Tmem150c	transmembrane protein 150C	-4.26
ENSMUSG00000062044	Lmtk3	lemur tyrosine kinase 3	-4.26
ENSMUSG00000000120	Ngfr	nerve growth factor receptor (TNFR superfamily, member 16)	-4.26
ENSMUSG00000064177	Ghrl	ghrelin	-4.27
ENSMUSG00000031551	Ido1	indoleamine 2,3-dioxygenase 1	-4.27
ENSMUSG00000071113	Mboat4	membrane bound O-acyltransferase domain containing 4	-4.27
ENSMUSG00000104301	Wdr49	WD repeat domain 49	-4.27
ENSMUSG00000015401	Cltrn	collectrin, amino acid transport regulator	-4.28
ENSMUSG00000043388	Tmem130	transmembrane protein 130	-4.28
ENSMUSG00000097311	Gm26871	predicted gene, 26871	-4.28
ENSMUSG00000026285	Pdcd1	programmed cell death 1	-4.29
ENSMUSG00000030020	Prickle2	prickle planar cell polarity protein 2	-4.29
ENSMUSG00000058297	Spock2	sparc/osteonectin, cwcv and kazal-like domains proteoglycan 2	-4.30
ENSMUSG00000061603	Akap6	A kinase (PRKA) anchor protein 6	-4.30
ENSMUSG00000026872	Zeb2	zinc finger E-box binding homeobox 2	-4.32
ENSMUSG00000079037	Prnp	prion protein	-4.33
ENSMUSG00000030683	Sez6l2	seizure related 6 homolog like 2	-4.33
ENSMUSG00000051331	Cacna1c	calcium channel, voltage-dependent, L type, alpha 1C subunit	-4.33
ENSMUSG00000028926	Cdk14	cyclin-dependent kinase 14	-4.34
ENSMUSG00000043496	Tril	TLR4 interactor with leucine-rich repeats	-4.34
ENSMUSG00000112556	Gm47339	predicted gene, 47339	-4.34
ENSMUSG00000032087	Dscaml1	DS cell adhesion molecule like 1	-4.34
ENSMUSG00000001901	Kcnh6	potassium voltage-gated channel, subfamily H (eag-related), member 6	-4.35
ENSMUSG00000020177	9530003J23Rik	RIKEN cDNA 9530003J23 gene	-4.35
ENSMUSG00000110557	Gm5159	predicted gene 5159	-4.35
ENSMUSG00000024990	Rbp4	retinol binding protein 4, plasma	-4.36
ENSMUSG00000024366	Gfra3	glial cell line derived neurotrophic factor family receptor alpha 3	-4.37
ENSMUSG00000028137	Celf3	CUGBP, Elav-like family member 3	-4.37
ENSMUSG00000021647	Cartpt	CART prepropeptide	-4.37
ENSMUSG00000038463	Olfml2b	olfactomedin-like 2B	-4.38
ENSMUSG00000060671	Atp8b2	ATPase, class I, type 8B, member 2	-4.39
ENSMUSG00000056752	Dnah9	dynein, axonemal, heavy chain 9	-4.39
ENSMUSG00000032356	Rasgrf1	RAS protein-specific guanine nucleotide-releasing factor 1	-4.39
ENSMUSG00000071489	Ptgdr	prostaglandin D receptor	-4.40
ENSMUSG00000092083	Kcnb2	potassium voltage gated channel, Shab-related subfamily, member 2	-4.41
ENSMUSG00000030359	Pzp	PZP, alpha-2-macroglobulin like	-4.41
ENSMUSG00000037032	Apbb1	amyloid beta (A4) precursor protein-binding, family B, member 1	-4.42
ENSMUSG00000073530	Pappa2	pappalysin 2	-4.42
ENSMUSG00000104283	Gm37459	predicted gene, 37459	-4.42
ENSMUSG00000018698	Lhx1	LIM homeobox protein 1	-4.43
ENSMUSG00000019900	Rfx6	regulatory factor X, 6	-4.44
ENSMUSG00000033740	St18	suppression of tumorigenicity 18	-4.44
ENSMUSG00000044365	Cxxc4	CXXC finger 4	-4.44
ENSMUSG00000016200	Syt14	synaptotagmin XIV	-4.44
ENSMUSG00000034145	Tmem63c	transmembrane protein 63c	-4.44
ENSMUSG00000033900	Map9	microtubule-associated protein 9	-4.45
ENSMUSG00000066456	Hmgn3	high mobility group nucleosomal binding domain 3	-4.46
ENSMUSG00000026833	Olfm1	olfactomedin 1	-4.46
ENSMUSG00000032769	Trpa1	transient receptor potential cation channel, subfamily A, member 1	-4.47
ENSMUSG00000103529	A730089K16Rik	RIKEN cDNA A730089K16 gene	-4.48
ENSMUSG00000045215	Asxl3	additional sex combs like 3, transcriptional regulator	-4.50
ENSMUSG00000027009	Itga4	integrin alpha 4	-4.51
ENSMUSG00000090291	Lrrc10b	leucine rich repeat containing 10B	-4.51
ENSMUSG00000010086	Rnf112	ring finger protein 112	-4.51
ENSMUSG00000022995	Enah	ENAH actin regulator	-4.52
ENSMUSG00000031561	Tenm3	teneurin transmembrane protein 3	-4.52
ENSMUSG00000046743	Fat4	FAT atypical cadherin 4	-4.53
ENSMUSG00000047878	A4galt	alpha 1,4-galactosyltransferase	-4.53
ENSMUSG00000042750	Bex2	brain expressed X-linked 2	-4.54
ENSMUSG00000062444	Ap3b2	adaptor-related protein complex 3, beta 2 subunit	-4.54
ENSMUSG00000027360	Hdc	histidine decarboxylase	-4.54
ENSMUSG00000079056	Kcnip3	Kv channel interacting protein 3, calsenilin	-4.54
ENSMUSG00000114501	Gm48582	predicted gene, 48582	-4.54
ENSMUSG00000056553	Ptprn2	protein tyrosine phosphatase, receptor type, N polypeptide 2	-4.55
ENSMUSG00000037306	Man1c1	mannosidase, alpha, class 1C, member 1	-4.55
ENSMUSG00000006586	Runx1t1	runt-related transcription factor 1; translocated to, 1 (cyclin D-related)	-4.56
ENSMUSG00000033676	Gabrb3	gamma-aminobutyric acid (GABA) A receptor, subunit beta 3	-4.56
ENSMUSG00000054667	Irs4	insulin receptor substrate 4	-4.56

ENSMUSG00000042078	Svop	SV2 related protein	-4.57
ENSMUSG00000101814	Gm17807	predicted gene, 17807	-4.59
ENSMUSG00000039683	Sdk1	sidekick cell adhesion molecule 1	-4.60
ENSMUSG00000051980	Casr	calcium-sensing receptor	-4.60
ENSMUSG00000028546	Elavl4	ELAV like RNA binding protein 4	-4.61
ENSMUSG00000055197	Fev	FEV (ETS oncogene family)	-4.61
ENSMUSG00000009292	Trpm2	transient receptor potential cation channel, subfamily M, member 2	-4.61
ENSMUSG00000037428	Vgf	VGF nerve growth factor inducible	-4.62
ENSMUSG00000031144	Syp	synaptophysin	-4.63
ENSMUSG00000029420	Rimbp2	RIMS binding protein 2	-4.64
ENSMUSG00000020218	Wif1	Wnt inhibitory factor 1	-4.66
ENSMUSG00000032338	Hcn4	hyperpolarization-activated, cyclic nucleotide-gated K+ 4	-4.67
ENSMUSG00000022762	Ncam2	neural cell adhesion molecule 2	-4.67
ENSMUSG00000032297	Celf6	CUGBP, Elav-like family member 6	-4.67
ENSMUSG00000027858	Tspan2	tetraspanin 2	-4.67
ENSMUSG00000114230	Gm48239	predicted gene, 48239	-4.68
ENSMUSG00000048218	Amigo2	adhesion molecule with Ig like domain 2	-4.69
ENSMUSG00000095079	Igha	immunoglobulin heavy constant alpha	-4.69
ENSMUSG00000020312	Shc2	SHC (Src homology 2 domain containing) transforming protein 2	-4.71
ENSMUSG00000001985	Grik3	glutamate receptor, ionotropic, kainate 3	-4.71
ENSMUSG00000044359	P2ry4	pyrimidinergic receptor P2Y, G-protein coupled, 4	-4.72
ENSMUSG00000038112	AW551984	expressed sequence AW551984	-4.72
ENSMUSG00000029765	Plxna4	plexin A4	-4.72
ENSMUSG00000035095	Fam167a	family with sequence similarity 167, member A	-4.74
ENSMUSG00000031837	Necab2	N-terminal EF-hand calcium binding protein 2	-4.76
ENSMUSG00000090071	Cdk5r2	cyclin-dependent kinase 5, regulatory subunit 2 (p39)	-4.77
ENSMUSG00000026527	Rgs7	regulator of G protein signaling 7	-4.77
ENSMUSG00000046999	1110032F04Rik	RIKEN cDNA 1110032F04 gene	-4.77
ENSMUSG00000026204	Ptprn	protein tyrosine phosphatase, receptor type, N	-4.78
ENSMUSG00000053049	Gm15413	predicted gene 15413	-4.78
ENSMUSG00000038530	Rgs4	regulator of G-protein signaling 4	-4.79
ENSMUSG00000061762	Tac1	tachykinin 1	-4.79
ENSMUSG00000058248	Kcnh1	potassium voltage-gated channel, subfamily H (eag-related), member 1	-4.79
ENSMUSG00000029875	Ccdc184	coiled-coil domain containing 184	-4.79
ENSMUSG00000023224	Serping1	serine (or cysteine) peptidase inhibitor, clade G, member 1	-4.80
ENSMUSG00000020886	Dlg4	discs large MAGUK scaffold protein 4	-4.81
ENSMUSG00000024084	Qpct	glutaminyl-peptide cyclotransferase (glutaminyl cyclase)	-4.81
ENSMUSG00000040972	Igsf21	immunoglobulin superfamily, member 21	-4.81
ENSMUSG00000062380	Tubb3	tubulin, beta 3 class III	-4.82
ENSMUSG00000083282	Ctsf	cathepsin F	-4.82
ENSMUSG00000049154	Fam183b	family with sequence similarity 183, member B	-4.83
ENSMUSG00000021700	Rab3c	RAB3C, member RAS oncogene family	-4.84
ENSMUSG00000022829	Stxbp5l	syntaxin binding protein 5-like	-4.84
ENSMUSG00000069072	Slc7a14	solute carrier family 7 (cationic amino acid transporter, y+ system), member 14	-4.86
ENSMUSG00000004113	Cacna1b	calcium channel, voltage-dependent, N type, alpha 1B subunit	-4.87
ENSMUSG00000031284	Pak3	p21 (RAC1) activated kinase 3	-4.87
ENSMUSG00000094626	Tmem121b	transmembrane protein 121B	-4.88
ENSMUSG00000040118	Cacna2d1	calcium channel, voltage-dependent, alpha2/delta subunit 1	-4.89
ENSMUSG00000029121	Crmp1	collapsin response mediator protein 1	-4.89
ENSMUSG00000029223	Uchl1	ubiquitin carboxy-terminal hydrolase L1	-4.90
ENSMUSG00000027220	Syt13	synaptotagmin XIII	-4.91
ENSMUSG00000028364	Tnc	tenascin C	-4.91
ENSMUSG00000041681	Iapp	islet amyloid polypeptide	-4.91
ENSMUSG00000064080	Fbln2	fibulin 2	-4.93
ENSMUSG00000045657	Pcdhb10	protocadherin beta 10	-4.93
ENSMUSG00000021303	Gng4	guanine nucleotide binding protein (G protein), gamma 4	-4.94
ENSMUSG00000050556	Kcnb1	potassium voltage gated channel, Shab-related subfamily, member 1	-4.95
ENSMUSG00000021337	Scgn	secretagogin, EF-hand calcium binding protein	-4.97
ENSMUSG00000026764	Kif5c	kinesin family member 5C	-4.97
ENSMUSG00000037852	Cpe	carboxypeptidase E	-4.98
ENSMUSG00000043518	Rai2	retinoic acid induced 2	-4.98
ENSMUSG00000016346	Kcnq2	potassium voltage-gated channel, subfamily Q, member 2	-4.98
ENSMUSG00000027350	Chgb	chromogranin B	-4.99
ENSMUSG00000091345	Col6a5	collagen, type VI, alpha 5	-5.00
ENSMUSG00000026686	Lmx1a	LIM homeobox transcription factor 1 alpha	-5.01
ENSMUSG00000026432	Avpr1b	arginine vasopressin receptor 1B	-5.01
ENSMUSG00000028226	Mmp16	matrix metallopeptidase 16	-5.02
ENSMUSG00000021194	Chga	chromogranin A	-5.03
ENSMUSG00000061601	Pclo	piccolo (presynaptic cytomatrix protein)	-5.04
ENSMUSG00000035431	Sstr1	somatostatin receptor 1	-5.04
ENSMUSG00000017311	Pyy	peptide YY	-5.05
ENSMUSG00000050751	Pgbd5	piggyBac transposable element derived 5	-5.06
ENSMUSG00000041141	Pnmal1	PNMA-like 1	-5.07

ENSMUSG00000039323	Igfbp2	insulin-like growth factor binding protein 2	-5.09
ENSMUSG00000024553	Galr1	galanin receptor 1	-5.11
ENSMUSG00000038456	Dennd2a	DENN/MADD domain containing 2A	-5.12
ENSMUSG00000115190	Gm49542	predicted gene, 49542	-5.12
ENSMUSG00000057716	Tmem178b	transmembrane protein 178B	-5.13
ENSMUSG00000024112	Cacna1h	calcium channel, voltage-dependent, T type, alpha 1H subunit	-5.14
ENSMUSG00000037624	Kcnk2	potassium channel, subfamily K, member 2	-5.14
ENSMUSG00000048216	Gpr85	G protein-coupled receptor 85	-5.14
ENSMUSG00000048215	A630023P12Rik	RIKEN cDNA A630023P12 gene	-5.15
ENSMUSG00000032181	Scg3	secretogranin III	-5.16
ENSMUSG00000041798	Gck	glucokinase	-5.20
ENSMUSG00000102498	Gm19445	predicted gene, 19445	-5.20
ENSMUSG00000020734	Grin2c	glutamate receptor, ionotropic, NMDA2C (epsilon 3)	-5.21
ENSMUSG00000019876	Pkib	protein kinase inhibitor beta, cAMP dependent, testis specific	-5.22
ENSMUSG00000023387	Kcnk16	potassium channel, subfamily K, member 16	-5.25
ENSMUSG00000022449	Adamts20	a disintegrin-like and metallopeptidase (reprolysin type) with thrombospondin type 1 motif, 20	-5.26
ENSMUSG00000020704	Asic2	acid-sensing (proton-gated) ion channel 2	-5.26
ENSMUSG00000039601	Rcan2	regulator of calcineurin 2	-5.27
ENSMUSG00000027273	Snap25	synaptosomal-associated protein 25	-5.28
ENSMUSG00000004366	Sst	somatostatin	-5.28
ENSMUSG00000052572	Dlg2	discs large MAGUK scaffold protein 2	-5.29
ENSMUSG00000021559	Dapk1	death associated protein kinase 1	-5.31
ENSMUSG00000056492	Adgrf5	adhesion G protein-coupled receptor F5	-5.31
ENSMUSG00000039372	38047	membrane-associated ring finger (C3HC4) 4	-5.34
ENSMUSG00000054932	Afp	alpha fetoprotein	-5.37
ENSMUSG00000048483	Zdhhc22	zinc finger, DHHC-type containing 22	-5.37
ENSMUSG00000044453	Ffar1	free fatty acid receptor 1	-5.38
ENSMUSG00000054640	Slc8a1	solute carrier family 8 (sodium/calcium exchanger), member 1	-5.39
ENSMUSG00000002265	Peg3	paternally expressed 3	-5.39
ENSMUSG00000075012	Fjx1	four jointed box 1	-5.41
ENSMUSG00000061911	Myt1l	myelin transcription factor 1-like	-5.41
ENSMUSG00000028354	Fmn2	formin 2	-5.42
ENSMUSG00000020181	Nav3	neuron navigator 3	-5.43
ENSMUSG00000051985	Igfn1	immunoglobulin-like and fibronectin type III domain containing 1	-5.47
ENSMUSG00000061171	Slc38a11	solute carrier family 38, member 11	-5.48
ENSMUSG00000111977	Gm47163	predicted gene, 47163	-5.48
ENSMUSG00000033981	Gria2	glutamate receptor, ionotropic, AMPA2 (alpha 2)	-5.49
ENSMUSG00000052727	Map1b	microtubule-associated protein 1B	-5.50
ENSMUSG00000042631	Xkr7	X-linked Kx blood group related 7	-5.53
ENSMUSG00000035277	Arx	aristaless related homeobox	-5.55
ENSMUSG00000027947	Il6ra	interleukin 6 receptor, alpha	-5.55
ENSMUSG00000026163	Sphkap	SPHK1 interactor, AKAP domain containing	-5.55
ENSMUSG00000049148	Plcxd3	phosphatidylinositol-specific phospholipase C, X domain containing 3	-5.56
ENSMUSG00000020723	Cacng4	calcium channel, voltage-dependent, gamma subunit 4	-5.58
ENSMUSG00000027168	Pax6	paired box 6	-5.59
ENSMUSG00000028445	Enho	energy homeostasis associated	-5.62
ENSMUSG00000022619	Mapk8ip2	mitogen-activated protein kinase 8 interacting protein 2	-5.66
ENSMUSG00000071265	1700086L19Rik	RIKEN cDNA 1700086L19 gene	-5.66
ENSMUSG00000029878	Dbpht2	DNA binding protein with his-thr domain	-5.66
ENSMUSG00000028008	Asic5	acid-sensing (proton-gated) ion channel family member 5	-5.67
ENSMUSG00000025089	Gfra1	glial cell line derived neurotrophic factor family receptor alpha 1	-5.68
ENSMUSG00000047842	Diras2	DIRAS family, GTP-binding RAS-like 2	-5.70
ENSMUSG00000053166	Cdh22	cadherin 22	-5.74
ENSMUSG00000059921	Unc5c	unc-5 netrin receptor C	-5.74
ENSMUSG00000020651	Slc26a4	solute carrier family 26, member 4	-5.76
ENSMUSG00000021198	Unc79	unc-79 homolog	-5.79
ENSMUSG00000048038	Ccdc187	coiled-coil domain containing 187	-5.87
ENSMUSG00000019890	Nts	neurotensin	-5.87
ENSMUSG00000039278	Pcsk1n	proprotein convertase subtilisin/kexin type 1 inhibitor	-5.89
ENSMUSG00000039860	Srrm3	serine/arginine repetitive matrix 3	-5.90
ENSMUSG00000052726	Kcnt2	potassium channel, subfamily T, member 2	-5.92
ENSMUSG00000054423	Cadps	Ca2+-dependent secretion activator	-5.94
ENSMUSG00000042258	Isl1	ISL1 transcription factor, LIM/homeodomain	-5.97
ENSMUSG00000068263	Efcc1	EF hand and coiled-coil domain containing 1	-5.98
ENSMUSG00000031740	Mmp2	matrix metallopeptidase 2	-5.99
ENSMUSG00000004151	Etv1	ets variant 1	-6.01
ENSMUSG00000053852	Adgrg4	adhesion G protein-coupled receptor G4	-6.14
ENSMUSG00000032532	Cck	cholecystokinin	-6.27
ENSMUSG00000033061	Resp18	regulated endocrine-specific protein 18	-6.30
ENSMUSG00000026824	Kcnj3	potassium inwardly-rectifying channel, subfamily J, member 3	-6.45
ENSMUSG00000050711	Scg2	secretogranin II	-6.48
ENSMUSG00000025375	Aatk	apoptosis-associated tyrosine kinase	-6.51
ENSMUSG00000024304	Cdh2	cadherin 2	-6.52

ENSMUSG00000015829	Tnr	tenascin R	-6.56
ENSMUSG00000004110	Cacna1e	calcium channel, voltage-dependent, R type, alpha 1E subunit	-6.62
ENSMUSG00000043301	Kcnj6	potassium inwardly-rectifying channel, subfamily J, member 6	-6.73
ENSMUSG00000031099	Smarca1	SWI/SNF related, matrix associated, actin dependent regulator of chromatin, subfamily a, member 1	-7.19
ENSMUSG00000014351	Gip	gastric inhibitory polypeptide	-7.88
ENSMUSG00000055409	Nell1	NEL-like 1	-8.89
ENSMUSG00000103321	4933403L11Rik	RIKEN cDNA 4933403L11 gene	-22.00

Supplementary Table S6

Differentially expressed genes in two populations of tdTomato-positive tumor cells sorted for Trop2 expression and in a population of tdTomato-negative (differentiated) cells obtained from microroadenomas 6 weeks after Apc inactivation

Differentially expressed genes (DEGs) in two populations of tdTomato-positive tumor cells sorted for Trop2 expression and in a population of tdTomato-negative (differentiated) cells obtained from microroadenomas 6 weeks after Apc inactivation. DEGs between $Trop2^{+}$ $tdTomato^{+}$ and $Trop2^{-}tdTomato^{+}$ cells and between the two tdTomato-positive cell groups and tdTomato-negative cells are shown on the corresponding sheets. Genes with an adjusted p-value < 0.05 and $|\log_2 FC| \geq 1$ were considered significant. Wnt-responsive genes are highlighted in red.

Tom+Trop2+ vs. Tom+Trop2- cells

Ensemble gene code	Symbol	Gene name	logFC
ENSMUSG00000063632	Sox11	SRY (sex determining region Y)-box 11	6.73
ENSMUSG00000051397	Tacstd2	tumor-associated calcium signal transducer 2	6.34
ENSMUSG00000004951	Hspb1	heat shock protein 1	5.49
ENSMUSG00000000120	Ngfr	nerve growth factor receptor (TNFR superfamily, member 16)	5.47
ENSMUSG00000030873	Scnn1b	sodium channel, nonvoltage-gated 1 beta	5.45
ENSMUSG00000042179	Pnliprp1	pancreatic lipase related protein 1	5.39
ENSMUSG00000108218	Olfr1372-ps1	olfactory receptor 1372, pseudogene 1	5.08
ENSMUSG00000007097	Atp1a2	ATPase, Na+/K+ transporting, alpha 2 polypeptide	5.04
ENSMUSG00000029838	Ptn	pleiotrophin	4.85
ENSMUSG00000019577	Pdk4	pyruvate dehydrogenase kinase, isoenzyme 4	4.77
ENSMUSG00000031284	Pak3	p21 (RAC1) activated kinase 3	4.68
ENSMUSG00000036480	Prss56	protease, serine 56	4.61
ENSMUSG00000053007	Creb5	cAMP responsive element binding protein 5	4.60
ENSMUSG00000081392	Gm11668	predicted gene 11668	4.58
ENSMUSG00000063919	Srrm4	serine/arginine repetitive matrix 4	4.58
ENSMUSG00000069892	9930111J21Rik2	RIKEN cDNA 9930111J21 gene 2	4.58
ENSMUSG00000028031	Dkk2	dickkopf WNT signaling pathway inhibitor 2	4.56
ENSMUSG00000000901	Mmp11	matrix metallopeptidase 11	4.56
ENSMUSG00000029648	Flt1	FMS-like tyrosine kinase 1	4.52
ENSMUSG00000014599	Csf1	colony stimulating factor 1 (macrophage)	4.47
ENSMUSG00000020363	Gfpt2	glutamine fructose-6-phosphate transaminase 2	4.46
ENSMUSG00000026822	Lcn2	lipocalin 2	4.45
ENSMUSG00000062591	Tubb4a	tubulin, beta 4A class IVA	4.43
ENSMUSG00000034220	Gpc1	glypican 1	4.43
ENSMUSG00000020218	Wif1	Wnt inhibitory factor 1	4.40
ENSMUSG00000027674	Pex5l	peroxisomal biogenesis factor 5-like	4.40
ENSMUSG00000025473	Adam8	a disintegrin and metallopeptidase domain 8	4.39
ENSMUSG00000074766	Ism1	isthmin 1, angiogenesis inhibitor	4.37
ENSMUSG00000063531	Sema3e	sema domain, immunoglobulin domain (Ig), short basic domain, secreted, (semaphorin) 3E	4.35
ENSMUSG00000044313	Mab21l3	mab-21-like 3 (C. elegans)	4.35
ENSMUSG00000026167	Wnt10a	wingless-type MMTV integration site family, member 10A	4.34
ENSMUSG00000032936	Camkv	CaM kinase-like vesicle-associated	4.34
ENSMUSG00000022037	Clu	clusterin	4.32
ENSMUSG00000046623	Gjb4	gap junction protein, beta 4	4.31
ENSMUSG00000028328	Tmod1	tropomodulin 1	4.31
ENSMUSG00000021986	Amer2	APC membrane recruitment 2	4.29
ENSMUSG00000034450	Gulo	gulonolactone (L-) oxidase	4.25
ENSMUSG00000027803	Wwtr1	WW domain containing transcription regulator 1	4.24
ENSMUSG00000042804	Gpr153	G protein-coupled receptor 153	4.23
ENSMUSG00000029380	Cxcl1	chemokine (C-X-C motif) ligand 1	4.21
ENSMUSG00000072591	5930412G12Rik	RIKEN cDNA 5930412G12 gene	4.21
ENSMUSG00000034463	Scara3	scavenger receptor class A, member 3	4.20
ENSMUSG00000071847	Apcdd1	adenomatosis polyposis coli down-regulated 1	4.16
ENSMUSG00000068327	Tlx2	T cell leukemia, homeobox 2	4.15
ENSMUSG00000037188	Grhl3	grainyhead like transcription factor 3	4.12
ENSMUSG00000018340	Anxa6	annexin A6	4.11
ENSMUSG00000025324	Atp10a	ATPase, class V, type 10A	4.11
ENSMUSG00000085412	Halr1	Hoxa adjacent long noncoding RNA 1	4.10
ENSMUSG00000009097	Tbx1	T-box 1	4.09
ENSMUSG00000053613	Notumos	notum palmitoleoyl-protein carboxylesterase, opposite strand	4.06
ENSMUSG00000027210	Meis2	Meis homeobox 2	4.05
ENSMUSG00000006576	Slc4a3	solute carrier family 4 (anion exchanger), member 3	4.05
ENSMUSG00000030246	Ldhb	lactate dehydrogenase B	4.00
ENSMUSG00000024164	C3	complement component 3	3.98
ENSMUSG00000031538	Plat	plasminogen activator, tissue	3.97
ENSMUSG00000025140	Pycr1	pyrroline-5-carboxylate reductase 1	3.97
ENSMUSG00000043903	Zfp469	zinc finger protein 469	3.95
ENSMUSG00000034612	Chst11	carbohydrate sulfotransferase 11	3.88
ENSMUSG00000039232	Stx11	syntaxin 11	3.88
ENSMUSG00000087201	Gm15261	predicted gene 15261	3.88
ENSMUSG00000016763	Scube1	signal peptide, CUB domain, EGF-like 1	3.86
ENSMUSG00000029108	Pcdh7	protocadherin 7	3.85
ENSMUSG00000049807	Arhgap23	Rho GTPase activating protein 23	3.84
ENSMUSG00000054555	Adam12	a disintegrin and metallopeptidase domain 12 (meltrin alpha)	3.81
ENSMUSG00000039959	Hip1	huntingtin interacting protein 1	3.80
ENSMUSG00000026764	Kif5c	kinesin family member 5C	3.80
ENSMUSG00000043430	Psapl1	prosaposin-like 1	3.79
ENSMUSG00000039153	Runx2	runt related transcription factor 2	3.79
ENSMUSG00000004791	Pgf	placental growth factor	3.79

ENSMUSG00000014602	Kif1a	kinesin family member 1A	3.77
ENSMUSG00000017466	Timp2	tissue inhibitor of metalloproteinase 2	3.77
ENSMUSG00000032496	Ltf	lactotransferrin	3.77
ENSMUSG00000025330	Padi4	peptidyl arginine deiminase, type IV	3.75
ENSMUSG00000078161	Erich3	glutamate rich 3	3.74
ENSMUSG00000020092	Pald1	phosphatase domain containing, paladin 1	3.74
ENSMUSG00000104918	Gm42944	predicted gene 42944	3.74
ENSMUSG00000022665	Ccdc80	coiled-coil domain containing 80	3.73
ENSMUSG00000048960	Prex2	phosphatidylinositol-3,4,5-trisphosphate-dependent Rac exchange factor 2	3.73
ENSMUSG00000021765	Fst	follistatin	3.70
ENSMUSG00000038572	Bpifb5	BPI fold containing family B, member 5	3.69
ENSMUSG00000020900	Myh10	myosin, heavy polypeptide 10, non-muscle	3.64
ENSMUSG00000063727	Tnfrsf11b	tumor necrosis factor receptor superfamily, member 11b (osteoprotegerin)	3.61
ENSMUSG00000026728	Vim	vimentin	3.61
ENSMUSG00000058297	Spock2	sparc/osteonectin, cwcv and kazal-like domains proteoglycan 2	3.61
ENSMUSG00000011305	Plin5	perilipin 5	3.61
ENSMUSG00000040093	Bmf	BCL2 modifying factor	3.60
ENSMUSG00000098132	Rassf10	Ras association (RalGDS/AF-6) domain family (N-terminal) member 10	3.58
ENSMUSG00000029368	Alb	albumin	3.56
ENSMUSG00000041608	Entpd3	ectonucleoside triphosphate diphosphohydrolase 3	3.54
ENSMUSG00000033508	Asprv1	aspartic peptidase, retroviral-like 1	3.54
ENSMUSG00000009628	Tex15	testis expressed gene 15	3.53
ENSMUSG00000013419	Zfp651	zinc finger protein 651	3.52
ENSMUSG00000028360	Slc44a5	solute carrier family 44, member 5	3.51
ENSMUSG00000054932	Afp	alpha fetoprotein	3.51
ENSMUSG00000032965	Ift57	intraflagellar transport 57	3.50
ENSMUSG00000013584	Aldh1a2	aldehyde dehydrogenase family 1, subfamily A2	3.50
ENSMUSG00000084822	Myadml2os	myeloid-associated differentiation marker-like 2, opposite strand	3.50
ENSMUSG00000034764	1700006J14Rik	RIKEN cDNA 1700006J14 gene	3.49
ENSMUSG00000048191	Muc6	mucin 6, gastric	3.48
ENSMUSG00000041293	Adgrf1	adhesion G protein-coupled receptor F1	3.48
ENSMUSG00000020651	Slc26a4	solute carrier family 26, member 4	3.47
ENSMUSG00000006205	Htra1	HtrA serine peptidase 1	3.46
ENSMUSG00000029761	Cald1	caldesmon 1	3.46
ENSMUSG00000041189	Chrnb1	cholinergic receptor, nicotinic, beta polypeptide 1 (muscle)	3.46
ENSMUSG00000030772	Dkk3	dickkopf WNT signaling pathway inhibitor 3	3.45
ENSMUSG00000022790	Igsf11	immunoglobulin superfamily, member 11	3.45
ENSMUSG00000023828	Slc22a3	solute carrier family 22 (organic cation transporter), member 3	3.45
ENSMUSG00000001864	Aif1l	allograft inflammatory factor 1-like	3.44
ENSMUSG00000041046	Ramp3	receptor (calcitonin) activity modifying protein 3	3.43
ENSMUSG00000085811	Cep112it	centrosomal protein 112, intronic transcript	3.43
ENSMUSG00000034634	Ly6d	lymphocyte antigen 6 complex, locus D	3.43
ENSMUSG00000025666	Tmem47	transmembrane protein 47	3.43
ENSMUSG00000005503	Evx1	even-skipped homeobox 1	3.42
ENSMUSG00000025352	Gdf11	growth differentiation factor 11	3.41
ENSMUSG00000031595	Pdgfrl	platelet-derived growth factor receptor-like	3.41
ENSMUSG00000020086	H2afy2	H2A histone family, member Y2	3.41
ENSMUSG00000031925	Maml2	mastermind like transcriptional coactivator 2	3.41
ENSMUSG00000035783	Acta2	actin, alpha 2, smooth muscle, aorta	3.41
ENSMUSG00000017204	Gsdma	gasdermin A	3.40
ENSMUSG00000040998	Npnt	nephronectin	3.40
ENSMUSG00000049556	Lingo1	leucine rich repeat and Ig domain containing 1	3.40
ENSMUSG00000025938	Slco5a1	solute carrier organic anion transporter family, member 5A1	3.39
ENSMUSG00000033763	Mtss1l	metastasis suppressor 1-like	3.39
ENSMUSG00000037035	Inhbb	inhibin beta-B	3.39
ENSMUSG00000017491	Rarb	retinoic acid receptor, beta	3.39
ENSMUSG00000032186	Tmod2	tropomodulin 2	3.39
ENSMUSG00000054967	Zfp647	zinc finger protein 647	3.39
ENSMUSG00000070436	Serpinh1	serine (or cysteine) peptidase inhibitor, clade H, member 1	3.39
ENSMUSG00000022594	Lynx1	Ly6/neurotoxin 1	3.36
ENSMUSG00000078922	Tgtp1	T cell specific GTPase 1	3.34
ENSMUSG00000110187	Gm45496	predicted gene 45496	3.33
ENSMUSG00000053137	Mapk11	mitogen-activated protein kinase 11	3.32
ENSMUSG00000033209	Ttc28	tetratricopeptide repeat domain 28	3.31
ENSMUSG00000055980	Irs1	insulin receptor substrate 1	3.31
ENSMUSG00000066438	Plekhd1	pleckstrin homology domain containing, family D (with coiled-coil domains) member 1	3.30
ENSMUSG00000024912	Fosl1	fos-like antigen 1	3.27
ENSMUSG00000028214	Gem	GTP binding protein (gene overexpressed in skeletal muscle)	3.27
ENSMUSG00000059854	Hydin	HYDIN, axonemal central pair apparatus protein	3.27
ENSMUSG00000046191	Pcdhb20	protocadherin beta 20	3.27
ENSMUSG00000050640	Tmem150c	transmembrane protein 150C	3.26
ENSMUSG00000004552	Ctse	cathepsin E	3.26

ENSMUSG00000041324	Inhba	inhibin beta-A	3.26
ENSMUSG00000029769	Ccdc136	coiled-coil domain containing 136	3.25
ENSMUSG00000110290	Gm45336	predicted gene 45336	3.25
ENSMUSG00000055333	Fat2	FAT atypical cadherin 2	3.23
ENSMUSG00000013338	Fer1l4	fer-1-like 4 (C. elegans)	3.23
ENSMUSG00000018211	Wfdc15b	WAP four-disulfide core domain 15B	3.23
ENSMUSG00000037129	Tmprss13	transmembrane protease, serine 13	3.22
ENSMUSG00000051486	Pcdhb11	protocadherin beta 11	3.21
ENSMUSG00000026620	Mark1	MAP/microtubule affinity regulating kinase 1	3.20
ENSMUSG00000029757	Dync1i1	dynein cytoplasmic 1 intermediate chain 1	3.19
ENSMUSG00000025854	Fam20c	family with sequence similarity 20, member C	3.18
ENSMUSG00000080316	Spaca6	sperm acrosome associated 6	3.18
ENSMUSG00000023009	Nckap5l	NCK-associated protein 5-like	3.17
ENSMUSG00000086395	A630014C17Rik	RIKEN cDNA A630014C17 gene	3.17
ENSMUSG00000031841	Cdh13	cadherin 13	3.16
ENSMUSG00000038587	Akap12	A kinase (PRKA) anchor protein (gravin) 12	3.14
ENSMUSG00000004044	Cavin1	caveolae associated 1	3.13
ENSMUSG00000040152	Thbs1	thrombospondin 1	3.12
ENSMUSG00000000983	Wfdc18	WAP four-disulfide core domain 18	3.12
ENSMUSG00000020674	Pxdn	peroxidasin	3.11
ENSMUSG00000079363	Gbp4	guanylate binding protein 4	3.11
ENSMUSG00000027375	Mal	myelin and lymphocyte protein, T cell differentiation protein	3.11
ENSMUSG00000032363	Adamts7	a disintegrin-like and metallopeptidase (reprolysin type) with thrombospondin type 1 motif, 7	3.10
ENSMUSG00000019990	Pde7b	phosphodiesterase 7B	3.09
ENSMUSG00000027376	Prom2	prominin 2	3.09
ENSMUSG00000042988	Notum	notum palmitoleoyl-protein carboxylesterase	3.08
ENSMUSG00000068923	Syt11	synaptotagmin XI	3.08
ENSMUSG00000046618	Olfml2a	olfactomedin-like 2A	3.08
ENSMUSG00000029287	Tgfbr3	transforming growth factor, beta receptor III	3.08
ENSMUSG00000018012	Rac3	Rac family small GTPase 3	3.07
ENSMUSG00000022883	Robo1	roundabout guidance receptor 1	3.07
ENSMUSG00000093908	Gm5784	predicted gene 5784	3.07
ENSMUSG00000044033	Ccdc141	coiled-coil domain containing 141	3.06
ENSMUSG00000034295	Fhod3	formin homology 2 domain containing 3	3.06
ENSMUSG00000058656	Samd12	sterile alpha motif domain containing 12	3.05
ENSMUSG00000038456	Dennd2a	DENN/MADD domain containing 2A	3.05
ENSMUSG00000055799	Tcf7l1	transcription factor 7 like 1 (T cell specific, HMG box)	3.05
ENSMUSG00000033389	Arhgap44	Rho GTPase activating protein 44	3.05
ENSMUSG00000026494	Kif26b	kinesin family member 26B	3.04
ENSMUSG00000048078	Tenm4	teneurin transmembrane protein 4	3.04
ENSMUSG00000046449	Nexmif	neurite extension and migration factor	3.04
ENSMUSG00000049866	Arl4c	ADP-ribosylation factor-like 4C	3.03
ENSMUSG00000037016	Frem2	Fras1 related extracellular matrix protein 2	3.03
ENSMUSG00000022456	Sept3	septin 3	3.03
ENSMUSG00000031328	Flna	filamin, alpha	3.02
ENSMUSG00000040714	Klc3	kinesin light chain 3	3.02
ENSMUSG00000110666	Gm9172	predicted gene 9172	3.02
ENSMUSG00000038576	Susd4	sushi domain containing 4	3.02
ENSMUSG00000068699	Flnc	filamin C, gamma	3.01
ENSMUSG00000029298	Gbp9	guanylate-binding protein 9	2.99
ENSMUSG00000013076	Amotl1	angiomotin-like 1	2.98
ENSMUSG00000078921	Tgtp2	T cell specific GTPase 2	2.98
ENSMUSG00000050212	Eva1b	eva-1 homolog B (C. elegans)	2.98
ENSMUSG00000044071	Fam19a2	family with sequence similarity 19, member A2	2.98
ENSMUSG00000039693	Msantd3	Myb/SANT-like DNA-binding domain containing 3	2.97
ENSMUSG00000042357	Gjb5	gap junction protein, beta 5	2.96
ENSMUSG00000026193	Fn1	fibronectin 1	2.95
ENSMUSG00000046731	Kctd11	potassium channel tetramerisation domain containing 11	2.95
ENSMUSG00000034675	Dbn1	drebrin 1	2.95
ENSMUSG00000075304	Sp5	trans-acting transcription factor 5	2.94
ENSMUSG00000037712	Fermt2	fermitin family member 2	2.94
ENSMUSG00000054263	Lifr	leukemia inhibitory factor receptor	2.94
ENSMUSG00000026117	Zap70	zeta-chain (TCR) associated protein kinase	2.94
ENSMUSG00000085111	Ascl4	achaete-scute family bHLH transcription factor 4	2.94
ENSMUSG00000041889	Shisa4	shisa family member 4	2.94
ENSMUSG00000024659	Anxa1	annexin A1	2.93
ENSMUSG00000071531	Gprin2	G protein regulated inducer of neurite outgrowth 2	2.93
ENSMUSG00000024940	Ltbp3	latent transforming growth factor beta binding protein 3	2.91
ENSMUSG00000034460	Six4	sine oculis-related homeobox 4	2.91
ENSMUSG00000037833	Sh2d4b	SH2 domain containing 4B	2.91
ENSMUSG00000036599	Chst12	carbohydrate sulfotransferase 12	2.91

ENSMUSG00000037813	D630003M21Rik	RIKEN cDNA D630003M21 gene	2.90
ENSMUSG00000033227	Wnt6	wingless-type MMTV integration site family, member 6	2.90
ENSMUSG00000023008	Fmnl3	formin-like 3	2.89
ENSMUSG00000021835	Bmp4	bone morphogenetic protein 4	2.89
ENSMUSG00000104612	Gm42449	predicted gene 42449	2.89
ENSMUSG00000020181	Nav3	neuron navigator 3	2.89
ENSMUSG00000072437	Nanos1	nanos C2HC-type zinc finger 1	2.88
ENSMUSG00000029869	Ephb6	Eph receptor B6	2.88
ENSMUSG00000032717	Mdfi	MyoD family inhibitor	2.88
ENSMUSG00000037681	Esyt3	extended synaptotagmin-like protein 3	2.87
ENSMUSG00000024206	Rfx2	regulatory factor X, 2 (influences HLA class II expression)	2.87
ENSMUSG00000010175	Prox1	prospero homeobox 1	2.86
ENSMUSG00000046169	Adamts6	a disintegrin-like and metallopeptidase (reprolysin type) with thrombospondin type 1 motif, 6	2.86
ENSMUSG00000026765	Lypd6b	LY6/PLAUR domain containing 6B	2.86
ENSMUSG00000046971	Pla2g4f	phospholipase A2, group IVF	2.86
ENSMUSG00000034573	Ptpn13	protein tyrosine phosphatase, non-receptor type 13	2.86
ENSMUSG00000067889	Sptbn2	spectrin beta, non-erythrocytic 2	2.85
ENSMUSG00000031661	Nkd1	naked cuticle 1	2.84
ENSMUSG00000014813	Stc1	stanniocalcin 1	2.84
ENSMUSG00000039676	Capsl	calcyphosine-like	2.84
ENSMUSG00000103189	Gm37092	predicted gene, 37092	2.84
ENSMUSG00000035357	Pdzrn3	PDZ domain containing RING finger 3	2.83
ENSMUSG00000029359	Tesc	tescalcin	2.83
ENSMUSG00000031871	Cdh5	cadherin 5	2.83
ENSMUSG00000035246	Pcyt1b	phosphate cytidylyltransferase 1, choline, beta isoform	2.83
ENSMUSG00000002980	Bcam	basal cell adhesion molecule	2.83
ENSMUSG00000001288	Rarg	retinoic acid receptor, gamma	2.82
ENSMUSG00000022176	Rem2	rad and gem related GTP binding protein 2	2.82
ENSMUSG00000105096	Gbp10	guanylate-binding protein 10	2.82
ENSMUSG00000020723	Cacng4	calcium channel, voltage-dependent, gamma subunit 4	2.81
ENSMUSG00000057193	Slc44a2	solute carrier family 44, member 2	2.80
ENSMUSG00000041390	Mdfic	MyoD family inhibitor domain containing	2.80
ENSMUSG00000051243	Islr2	immunoglobulin superfamily containing leucine-rich repeat 2	2.80
ENSMUSG00000035678	Tnfsf9	tumor necrosis factor (ligand) superfamily, member 9	2.78
ENSMUSG00000040430	Pitpnc1	phosphatidylinositol transfer protein, cytoplasmic 1	2.77
ENSMUSG00000070337	Gpr179	G protein-coupled receptor 179	2.77
ENSMUSG00000031355	Arhgap6	Rho GTPase activating protein 6	2.76
ENSMUSG00000050052	Tdrp	testis development related protein	2.75
ENSMUSG00000027985	Lef1	lymphoid enhancer binding factor 1	2.74
ENSMUSG00000024909	Efemp2	epidermal growth factor-containing fibulin-like extracellular matrix protein 2	2.74
ENSMUSG00000044948	Cfap43	cilia and flagella associated protein 43	2.74
ENSMUSG00000071984	Fndc1	fibronectin type III domain containing 1	2.74
ENSMUSG00000069255	Dusp22	dual specificity phosphatase 22	2.73
ENSMUSG00000028883	Sema3a	sema domain, immunoglobulin domain (Ig), short basic domain, secreted, (semaphorin) 3A	2.73
ENSMUSG00000068748	Ptprz1	protein tyrosine phosphatase, receptor type Z, polypeptide 1	2.72
ENSMUSG00000039607	Rbms3	RNA binding motif, single stranded interacting protein	2.71
ENSMUSG00000070304	Scn2b	sodium channel, voltage-gated, type II, beta	2.71
ENSMUSG00000035954	Dock4	dedicator of cytokinesis 4	2.71
ENSMUSG00000028497	Hacd4	3-hydroxyacyl-CoA dehydratase 4	2.71
ENSMUSG00000045667	Smtnl2	smoothelin-like 2	2.71
ENSMUSG00000022435	Upk3a	uroplakin 3A	2.71
ENSMUSG00000063851	Rnf183	ring finger protein 183	2.71
ENSMUSG00000038415	Foxq1	forkhead box Q1	2.70
ENSMUSG00000070867	Trabd2b	TraB domain containing 2B	2.70
ENSMUSG00000035274	Tpbg	trophoblast glycoprotein	2.69
ENSMUSG00000015647	Lama5	laminin, alpha 5	2.68
ENSMUSG00000041134	Cyyr1	cysteine and tyrosine-rich protein 1	2.68
ENSMUSG00000020427	Igfbp3	insulin-like growth factor binding protein 3	2.68
ENSMUSG00000032327	Stra6	stimulated by retinoic acid gene 6	2.68
ENSMUSG00000032875	Arhgef17	Rho guanine nucleotide exchange factor (GEF) 17	2.67
ENSMUSG00000060429	Sntb1	syntrophin, basic 1	2.67
ENSMUSG00000020814	Mxra7	matrix-remodelling associated 7	2.67
ENSMUSG00000040852	Plekhh2	pleckstrin homology domain containing, family H (with MyTH4 domain) member 2	2.66
ENSMUSG00000111212	Gm47087	predicted gene, 47087	2.66
ENSMUSG00000026556	Vangl2	VANGL planar cell polarity 2	2.65
ENSMUSG00000040488	Ltbp4	latent transforming growth factor beta binding protein 4	2.65
ENSMUSG00000052911	Lamb2	laminin, beta 2	2.65
ENSMUSG00000032076	Cadm1	cell adhesion molecule 1	2.65
ENSMUSG00000058317	Ube2e2	ubiquitin-conjugating enzyme E2E 2	2.65
ENSMUSG00000033377	Palmd	palmdelphin	2.65
ENSMUSG00000034684	Sema3f	sema domain, immunoglobulin domain (Ig), short basic domain, secreted, (semaphorin) 3F	2.63

ENSMUSG00000020044	Timp3	tissue inhibitor of metalloproteinase 3	2.63
ENSMUSG00000036502	Tmem255a	transmembrane protein 255A	2.63
ENSMUSG00000015134	Aldh1a3	aldehyde dehydrogenase family 1, subfamily A3	2.62
ENSMUSG00000022587	Ly6e	lymphocyte antigen 6 complex, locus E	2.61
ENSMUSG00000040836	Gpr161	G protein-coupled receptor 161	2.61
ENSMUSG00000026475	Rgs16	regulator of G-protein signaling 16	2.61
ENSMUSG00000097344	Gm26732	predicted gene, 26732	2.61
ENSMUSG00000021493	Pdlim7	PDZ and LIM domain 7	2.60
ENSMUSG00000031849	Comp	cartilage oligomeric matrix protein	2.60
ENSMUSG00000058145	Adamts17	a disintegrin-like and metallopeptidase (reprolysin type) with thrombospondin type 1 motif, 17	2.59
ENSMUSG00000048644	Ctxn1	cortexin 1	2.59
ENSMUSG00000000552	Zfp385a	zinc finger protein 385A	2.59
ENSMUSG00000020599	Rgs9	regulator of G-protein signaling 9	2.58
ENSMUSG00000029771	Irf5	interferon regulatory factor 5	2.58
ENSMUSG00000061517	Sox21	SRY (sex determining region Y)-box 21	2.57
ENSMUSG00000036053	Fmnl2	formin-like 2	2.56
ENSMUSG00000047747	Rnf150	ring finger protein 150	2.56
ENSMUSG00000090394	4930523C07Rik	RIKEN cDNA 4930523C07 gene	2.56
ENSMUSG00000038496	Slc19a3	solute carrier family 19, member 3	2.56
ENSMUSG00000108833	Gm17909	predicted gene, 17909	2.56
ENSMUSG00000038552	Fndc4	fibronectin type III domain containing 4	2.56
ENSMUSG00000022871	Fetub	fetuin beta	2.56
ENSMUSG00000024235	Map3k8	mitogen-activated protein kinase kinase kinase 8	2.55
ENSMUSG00000032719	Sbspon	somatomedin B and thrombospondin, type 1 domain containing	2.55
ENSMUSG00000025492	Ifitm3	interferon induced transmembrane protein 3	2.54
ENSMUSG00000006411	Nectin4	nectin cell adhesion molecule 4	2.54
ENSMUSG00000022146	Osmr	oncostatin M receptor	2.54
ENSMUSG00000032135	Mcam	melanoma cell adhesion molecule	2.54
ENSMUSG00000050989	Selenon	selenoprotein N	2.53
ENSMUSG00000025855	Prkar1b	protein kinase, cAMP dependent regulatory, type I beta	2.53
ENSMUSG00000039601	Rcan2	regulator of calcineurin 2	2.52
ENSMUSG00000030600	Lrfn1	leucine rich repeat and fibronectin type III domain containing 1	2.52
ENSMUSG00000013089	Etv5	ets variant 5	2.51
ENSMUSG00000002633	Shh	sonic hedgehog	2.51
ENSMUSG00000030074	Gxylt2	glucoside xylosyltransferase 2	2.51
ENSMUSG00000030605	Mfge8	milk fat globule-EGF factor 8 protein	2.50
ENSMUSG00000041801	Phlda3	pleckstrin homology like domain, family A, member 3	2.50
ENSMUSG00000036158	Prickle1	prickle planar cell polarity protein 1	2.50
ENSMUSG00000026473	Glul	glutamate-ammonia ligase (glutamine synthetase)	2.49
ENSMUSG00000049281	Scn3b	sodium channel, voltage-gated, type III, beta	2.49
ENSMUSG00000051236	Msrb3	methionine sulfoxide reductase B3	2.48
ENSMUSG00000013236	Ptprs	protein tyrosine phosphatase, receptor type, S	2.47
ENSMUSG00000058952	Cfi	complement component factor i	2.47
ENSMUSG00000089715	Cbx6	chromobox 6	2.45
ENSMUSG00000029101	Rgs12	regulator of G-protein signaling 12	2.45
ENSMUSG00000037902	Sirpa	signal-regulatory protein alpha	2.45
ENSMUSG00000027257	Pacsin3	protein kinase C and casein kinase substrate in neurons 3	2.44
ENSMUSG00000022464	Slc38a4	solute carrier family 38, member 4	2.43
ENSMUSG00000034593	Myo5a	myosin VA	2.43
ENSMUSG00000027223	Mapk8ip1	mitogen-activated protein kinase 8 interacting protein 1	2.43
ENSMUSG00000028246	Faxc	failed axon connections homolog	2.43
ENSMUSG00000053477	Tcf4	transcription factor 4	2.42
ENSMUSG00000024349	Tmem173	transmembrane protein 173	2.42
ENSMUSG00000035376	Hacd2	3-hydroxyacyl-CoA dehydratase 2	2.42
ENSMUSG00000031398	Plxna3	plexin A3	2.42
ENSMUSG00000059668	Krt4	keratin 4	2.42
ENSMUSG00000009687	Fxyd5	FXYD domain-containing ion transport regulator 5	2.42
ENSMUSG00000112249	Gm30262	predicted gene, 30262	2.42
ENSMUSG00000030796	Tead2	TEA domain family member 2	2.41
ENSMUSG00000009739	Pou6f1	POU domain, class 6, transcription factor 1	2.41
ENSMUSG00000023972	Ptk7	PTK7 protein tyrosine kinase 7	2.40
ENSMUSG00000008398	Elk3	ELK3, member of ETS oncogene family	2.40
ENSMUSG00000035640	Cbarp	calcium channel, voltage-dependent, beta subunit associated regulatory protein	2.40
ENSMUSG00000024401	Tnf	tumor necrosis factor	2.40
ENSMUSG00000053646	Plxnb1	plexin B1	2.38
ENSMUSG00000002603	Tgfb1	transforming growth factor, beta 1	2.38
ENSMUSG00000001131	Timp1	tissue inhibitor of metalloproteinase 1	2.38
ENSMUSG00000020262	Adarb1	adenosine deaminase, RNA-specific, B1	2.38
ENSMUSG00000110802	Gm47141	predicted gene, 47141	2.38
ENSMUSG00000026579	F5	coagulation factor V	2.37
ENSMUSG00000045545	Krt14	keratin 14	2.37

ENSMUSG00000025202	Scd3	stearoyl-coenzyme A desaturase 3	2.37
ENSMUSG00000043391	2510009E07Rik	RIKEN cDNA 2510009E07 gene	2.36
ENSMUSG00000019853	Hebp2	heme binding protein 2	2.36
ENSMUSG00000053049	Gm15413	predicted gene 15413	2.36
ENSMUSG00000020728	Cep112	centrosomal protein 112	2.35
ENSMUSG00000014329	Bicc1	BicC family RNA binding protein 1	2.35
ENSMUSG00000085851	4921518K17Rik	RIKEN cDNA 4921518K17 gene	2.35
ENSMUSG00000074480	Mex3a	mex3 RNA binding family member A	2.34
ENSMUSG00000040415	Dtx3	deltex 3, E3 ubiquitin ligase	2.34
ENSMUSG00000026676	Ccdc3	coiled-coil domain containing 3	2.33
ENSMUSG00000022371	Col14a1	collagen, type XIV, alpha 1	2.33
ENSMUSG00000028100	Nudt17	nudix (nucleoside diphosphate linked moiety X)-type motif 17	2.33
ENSMUSG00000034685	Fam171a2	family with sequence similarity 171, member A2	2.33
ENSMUSG00000000782	Tcf7	transcription factor 7, T cell specific	2.32
ENSMUSG00000037095	Lrg1	leucine-rich alpha-2-glycoprotein 1	2.32
ENSMUSG00000024810	Il33	interleukin 33	2.32
ENSMUSG00000052105	Mtcl1	microtubule crosslinking factor 1	2.31
ENSMUSG00000022421	Nptxr	neuronal pentraxin receptor	2.31
ENSMUSG00000049848	Ceacam19	carcinoembryonic antigen-related cell adhesion molecule 19	2.30
ENSMUSG00000031760	Mt3	metallothionein 3	2.30
ENSMUSG00000060166	Zdhhc8	zinc finger, DHHC domain containing 8	2.29
ENSMUSG00000113211	4921525O09Rik	RIKEN cDNA 4921525O09 gene	2.29
ENSMUSG00000016526	Dyrk3	dual-specificity tyrosine-(Y)-phosphorylation regulated kinase 3	2.27
ENSMUSG00000041961	Znrf3	zinc and ring finger 3	2.26
ENSMUSG00000041570	Camsap2	calmodulin regulated spectrin-associated protein family, member 2	2.26
ENSMUSG00000029832	Nfe2l3	nuclear factor, erythroid derived 2, like 3	2.26
ENSMUSG00000021747	4930452B06Rik	RIKEN cDNA 4930452B06 gene	2.26
ENSMUSG00000037053	Azgp1	alpha-2-glycoprotein 1, zinc	2.25
ENSMUSG00000050071	Bex1	brain expressed X-linked 1	2.24
ENSMUSG00000028199	Cryz	crystallin, zeta	2.24
ENSMUSG00000021256	Vash1	vasohibin 1	2.24
ENSMUSG00000040690	Col16a1	collagen, type XVI, alpha 1	2.23
ENSMUSG00000020937	Plcd3	phospholipase C, delta 3	2.23
ENSMUSG00000039004	Bmp6	bone morphogenetic protein 6	2.22
ENSMUSG00000032625	Thsd7a	thrombospondin, type I, domain containing 7A	2.21
ENSMUSG00000033287	Kctd17	potassium channel tetramerisation domain containing 17	2.21
ENSMUSG00000082361	Btc	betacellulin, epidermal growth factor family member	2.20
ENSMUSG00000063455	D630045J12Rik	RIKEN cDNA D630045J12 gene	2.20
ENSMUSG00000025592	Dach2	dachshund family transcription factor 2	2.20
ENSMUSG00000045136	Tubb2b	tubulin, beta 2B class IIB	2.18
ENSMUSG00000020844	Nxn	nucleoredoxin	2.18
ENSMUSG00000024399	Ltb	lymphotoxin B	2.18
ENSMUSG00000001435	Col18a1	collagen, type XVIII, alpha 1	2.17
ENSMUSG00000043639	Rbm20	RNA binding motif protein 20	2.17
ENSMUSG00000038156	Spon1	spondin 1, (f-spondin) extracellular matrix protein	2.17
ENSMUSG00000042306	S100a14	S100 calcium binding protein A14	2.15
ENSMUSG00000056856	Jakmip3	janus kinase and microtubule interacting protein 3	2.15
ENSMUSG00000025038	Efhc2	EF-hand domain (C-terminal) containing 2	2.15
ENSMUSG00000031441	Atp11a	ATPase, class VI, type 11A	2.14
ENSMUSG00000022696	Sidt1	SID1 transmembrane family, member 1	2.14
ENSMUSG00000028698	Pik3r3	phosphoinositide-3-kinase regulatory subunit 3	2.14
ENSMUSG00000054793	Cadm4	cell adhesion molecule 4	2.14
ENSMUSG00000105681	Gm43428	predicted gene 43428	2.14
ENSMUSG00000044043	Pcdhb14	protocadherin beta 14	2.14
ENSMUSG00000039457	Ppl	periplakin	2.13
ENSMUSG00000041930	Fam222a	family with sequence similarity 222, member A	2.13
ENSMUSG00000107796	Gm44068	predicted gene, 44068	2.13
ENSMUSG00000001473	Tubb6	tubulin, beta 6 class V	2.12
ENSMUSG00000045319	Proser2	proline and serine rich 2	2.12
ENSMUSG00000048058	Ldlrad3	low density lipoprotein receptor class A domain containing 3	2.12
ENSMUSG00000060671	Atp8b2	ATPase, class I, type 8B, member 2	2.12
ENSMUSG00000033967	Rnf225	ring finger protein 225	2.12
ENSMUSG00000042524	Sun2	Sad1 and UNC84 domain containing 2	2.11
ENSMUSG00000020882	Cacnb1	calcium channel, voltage-dependent, beta 1 subunit	2.11
ENSMUSG00000032013	Trim29	tripartite motif-containing 29	2.11
ENSMUSG00000028780	Sema3c	sema domain, immunoglobulin domain (Ig), short basic domain, secreted, (semaphorin) 3C	2.10
ENSMUSG00000000320	Alox12	arachidonate 12-lipoxygenase	2.10
ENSMUSG00000029094	Afap1	actin filament associated protein 1	2.09
ENSMUSG00000032643	Fhl3	four and a half LIM domains 3	2.09
ENSMUSG00000023885	Thbs2	thrombospondin 2	2.08
ENSMUSG00000025582	Nptx1	neuronal pentraxin 1	2.08

ENSMUSG00000022754	Tmem45a	transmembrane protein 45a	2.08
ENSMUSG00000037239	Spred3	sprouty-related, EVH1 domain containing 3	2.08
ENSMUSG00000086537	Nespas	neuroendocrine secretory protein antisense	2.08
ENSMUSG00000039328	Rnf122	ring finger protein 122	2.07
ENSMUSG00000017723	Wfdc2	WAP four-disulfide core domain 2	2.07
ENSMUSG00000009378	Slc16a12	solute carrier family 16 (monocarboxylic acid transporters), member 12	2.06
ENSMUSG00000002058	Unc119	unc-119 lipid binding chaperone	2.06
ENSMUSG00000044034	Npb	neuropeptide B	2.06
ENSMUSG00000085514	Bcas3os2	breast carcinoma amplified sequence 3 opposite strand 2	2.06
ENSMUSG00000102758	Naaladl2	N-acetylated alpha-linked acidic dipeptidase-like 2	2.05
ENSMUSG00000023905	Tnfrsf12a	tumor necrosis factor receptor superfamily, member 12a	2.05
ENSMUSG00000017667	Zfp334	zinc finger protein 334	2.05
ENSMUSG00000026405	C4bp	complement component 4 binding protein	2.05
ENSMUSG00000042268	Slc26a9	solute carrier family 26, member 9	2.05
ENSMUSG00000059493	Nhs	NHS actin remodeling regulator	2.04
ENSMUSG00000036412	Arsi	arylsulfatase i	2.04
ENSMUSG00000021756	Il6st	interleukin 6 signal transducer	2.03
ENSMUSG00000025608	Podxl	podocalyxin-like	2.03
ENSMUSG00000110218	Gm20219	predicted gene, 20219	2.03
ENSMUSG00000004709	Cd244a	CD244 molecule A	2.03
ENSMUSG00000068270	Shroom4	shroom family member 4	2.03
ENSMUSG00000037946	Fgd3	FYVE, RhoGEF and PH domain containing 3	2.02
ENSMUSG00000031074	Fgf3	fibroblast growth factor 3	2.02
ENSMUSG00000068617	Efcab1	EF-hand calcium binding domain 1	2.02
ENSMUSG00000037736	Limch1	LIM and calponin homology domains 1	2.01
ENSMUSG00000028600	Podn	podocan	2.01
ENSMUSG00000003541	Ier3	immediate early response 3	2.01
ENSMUSG00000019817	Plagl1	pleiomorphic adenoma gene-like 1	2.01
ENSMUSG00000073409	H2-Q6	histocompatibility 2, Q region locus 6	2.01
ENSMUSG00000037892	Pcdh18	protocadherin 18	2.01
ENSMUSG00000046470	Sox18	SRY (sex determining region Y)-box 18	2.01
ENSMUSG00000071855	Ccdc112	coiled-coil domain containing 112	2.00
ENSMUSG00000022425	Enpp2	ectonucleotide pyrophosphatase/phosphodiesterase 2	2.00
ENSMUSG00000044022	Pcdhb21	protocadherin beta 21	2.00
ENSMUSG00000051790	Nlgn2	neuroligin 2	1.99
ENSMUSG00000024270	Slc39a6	solute carrier family 39 (metal ion transporter), member 6	1.99
ENSMUSG00000007207	Stx1a	syntaxin 1A (brain)	1.99
ENSMUSG00000089762	Ier5l	immediate early response 5-like	1.99
ENSMUSG00000039239	Tgfb2	transforming growth factor, beta 2	1.99
ENSMUSG00000028949	Smarcd3	SWI/SNF related, matrix associated, actin dependent regulator of chromatin, subfamily d, member 3	1.99
ENSMUSG00000021477	Ctsl	cathepsin L	1.98
ENSMUSG00000035901	Dennd5a	DENN/MADD domain containing 5A	1.98
ENSMUSG00000056758	Hmga2	high mobility group AT-hook 2	1.98
ENSMUSG00000029312	Klhl8	kelch-like 8	1.98
ENSMUSG00000044350	Lacc1	laccase domain containing 1	1.98
ENSMUSG00000059336	Slc14a1	solute carrier family 14 (urea transporter), member 1	1.98
ENSMUSG00000037060	Cavin3	caveolae associated 3	1.98
ENSMUSG00000027381	Bcl2l11	BCL2-like 11 (apoptosis facilitator)	1.97
ENSMUSG00000000555	Itga5	integrin alpha 5 (fibronectin receptor alpha)	1.97
ENSMUSG00000026411	Tmem9	transmembrane protein 9	1.97
ENSMUSG00000046546	Fam43a	family with sequence similarity 43, member A	1.97
ENSMUSG00000043895	S1pr2	sphingosine-1-phosphate receptor 2	1.97
ENSMUSG00000038146	Notch3	notch 3	1.97
ENSMUSG00000026121	Sema4c	sema domain, immunoglobulin domain (Ig), transmembrane domain (TM) and short cytoplasmic dom	1.96
ENSMUSG00000091183	Gm5141	predicted gene 5141	1.96
ENSMUSG00000037846	Rtkn2	rhotekin 2	1.95
ENSMUSG00000024558	Mapk4	mitogen-activated protein kinase 4	1.94
ENSMUSG00000046329	Slc25a23	solute carrier family 25 (mitochondrial carrier; phosphate carrier), member 23	1.94
ENSMUSG00000038775	Vill	villin-like	1.94
ENSMUSG00000002900	Lamb1	laminin B1	1.94
ENSMUSG00000031066	Usp11	ubiquitin specific peptidase 11	1.94
ENSMUSG00000030352	Tspan9	tetraspanin 9	1.94
ENSMUSG00000072964	Bhlhb9	basic helix-loop-helix domain containing, class B9	1.93
ENSMUSG00000037405	Icam1	intercellular adhesion molecule 1	1.93
ENSMUSG00000055745	Rtl6	retrotransposon Gag like 6	1.93
ENSMUSG00000021253	Tgfb3	transforming growth factor, beta 3	1.93
ENSMUSG00000057895	Zfp105	zinc finger protein 105	1.93
ENSMUSG00000044548	Dact1	dishevelled-binding antagonist of beta-catenin 1	1.93
ENSMUSG00000040990	Sh3kbp1	SH3-domain kinase binding protein 1	1.92
ENSMUSG00000071637	Cebpd	CCAAT/enhancer binding protein (C/EBP), delta	1.92
ENSMUSG00000037447	Arid5a	AT rich interactive domain 5A (MRF1-like)	1.92

ENSMUSG00000007039	Ddah2	dimethylarginine dimethylaminohydrolase 2	1.92
ENSMUSG00000062012	Zfp13	zinc finger protein 13	1.92
ENSMUSG00000060548	Tnfrsf19	tumor necrosis factor receptor superfamily, member 19	1.92
ENSMUSG00000043448	Gjc2	gap junction protein, gamma 2	1.92
ENSMUSG00000002059	Rab34	RAB34, member RAS oncogene family	1.92
ENSMUSG00000045287	Rtn4rl1	reticulon 4 receptor-like 1	1.91
ENSMUSG00000015243	Abca1	ATP-binding cassette, sub-family A (ABC1), member 1	1.90
ENSMUSG00000026223	Itm2c	integral membrane protein 2C	1.90
ENSMUSG00000031530	Dusp4	dual specificity phosphatase 4	1.90
ENSMUSG00000003363	Pld3	phospholipase D family, member 3	1.90
ENSMUSG00000019732	Calr3	calreticulin 3	1.90
ENSMUSG00000058908	Pla2g2a	phospholipase A2, group IIA (platelets, synovial fluid)	1.89
ENSMUSG00000039103	Nexn	nexilin	1.89
ENSMUSG00000031734	Irx3	Iroquois related homeobox 3	1.89
ENSMUSG00000028358	Zfp618	zinc finger protein 618	1.88
ENSMUSG00000041202	Pla2g2d	phospholipase A2, group IID	1.88
ENSMUSG00000113603	Gm47004	predicted gene, 47004	1.88
ENSMUSG00000026478	Lamc1	laminin, gamma 1	1.87
ENSMUSG00000022178	Ajuba	ajuba LIM protein	1.87
ENSMUSG00000090386	Mir99ahg	Mir99a and Mirlet7c-1 host gene (non-protein coding)	1.87
ENSMUSG00000044562	Rasip1	Ras interacting protein 1	1.87
ENSMUSG00000015709	Arnt2	aryl hydrocarbon receptor nuclear translocator 2	1.86
ENSMUSG00000020620	Abca8b	ATP-binding cassette, sub-family A (ABC1), member 8b	1.86
ENSMUSG00000053141	Ptprt	protein tyrosine phosphatase, receptor type, T	1.85
ENSMUSG00000031596	Slc7a2	solute carrier family 7 (cationic amino acid transporter, y+ system), member 2	1.85
ENSMUSG00000007989	Fzd3	frizzled class receptor 3	1.85
ENSMUSG00000019235	Rps6kl1	ribosomal protein S6 kinase-like 1	1.85
ENSMUSG00000028108	Ecm1	extracellular matrix protein 1	1.85
ENSMUSG00000030409	Dmpk	dystrophia myotonica-protein kinase	1.84
ENSMUSG00000026944	Abca2	ATP-binding cassette, sub-family A (ABC1), member 2	1.84
ENSMUSG00000073987	Ggh	gamma-glutamyl hydrolase	1.84
ENSMUSG00000039481	Nrtn	neurturin	1.84
ENSMUSG00000069170	Adgrv1	adhesion G protein-coupled receptor V1	1.84
ENSMUSG00000052212	Cd177	CD177 antigen	1.83
ENSMUSG00000025921	Rdh10	retinol dehydrogenase 10 (all-trans)	1.83
ENSMUSG00000028763	Hspg2	perlecan (heparan sulfate proteoglycan 2)	1.83
ENSMUSG00000001521	Tulp3	tubby-like protein 3	1.83
ENSMUSG00000022415	Syngr1	synaptogyrin 1	1.83
ENSMUSG00000019986	Ahi1	Abelson helper integration site 1	1.83
ENSMUSG00000104063	Pcdhgb7	protocadherin gamma subfamily B, 7	1.83
ENSMUSG00000038545	Cul7	cullin 7	1.82
ENSMUSG00000037071	Scd1	stearoyl-Coenzyme A desaturase 1	1.82
ENSMUSG00000062661	Ncs1	neuronal calcium sensor 1	1.82
ENSMUSG00000091941	Gm7399	predicted gene 7399	1.82
ENSMUSG00000028173	Wls	wntless WNT ligand secretion mediator	1.80
ENSMUSG00000053897	Slc39a8	solute carrier family 39 (metal ion transporter), member 8	1.80
ENSMUSG00000046572	Zfp518b	zinc finger protein 518B	1.80
ENSMUSG00000020646	Mboat2	membrane bound O-acyltransferase domain containing 2	1.80
ENSMUSG00000032698	Lmo2	LIM domain only 2	1.80
ENSMUSG00000026637	Traf5	TNF receptor-associated factor 5	1.80
ENSMUSG00000027712	Anxa5	annexin A5	1.79
ENSMUSG00000031875	Cmtm3	CKLF-like MARVEL transmembrane domain containing 3	1.79
ENSMUSG00000046589	Lrrc8e	leucine rich repeat containing 8 family, member E	1.79
ENSMUSG00000026065	Slc9a4	solute carrier family 9 (sodium/hydrogen exchanger), member 4	1.79
ENSMUSG00000040723	Rcsd1	RCSD domain containing 1	1.79
ENSMUSG00000076431	Sox4	SRY (sex determining region Y)-box 4	1.78
ENSMUSG00000028357	Kif12	kinesin family member 12	1.78
ENSMUSG00000021373	Cap2	CAP, adenylate cyclase-associated protein, 2 (yeast)	1.78
ENSMUSG00000066607	Insyn1	inhibitory synaptic factor 1	1.78
ENSMUSG00000039238	Zfp750	zinc finger protein 750	1.78
ENSMUSG00000063018	2010204K13Rik	RIKEN cDNA 2010204K13 gene	1.78
ENSMUSG00000027858	Tspan2	tetraspanin 2	1.78
ENSMUSG00000027860	Vangl1	VANGL planar cell polarity 1	1.77
ENSMUSG00000033730	Egr3	early growth response 3	1.77
ENSMUSG00000112014	Gm48435	predicted gene, 48435	1.77
ENSMUSG00000021340	Gpld1	glycosylphosphatidylinositol specific phospholipase D1	1.76
ENSMUSG00000025372	Baiap2	brain-specific angiogenesis inhibitor 1-associated protein 2	1.76
ENSMUSG00000053080	2700081O15Rik	RIKEN cDNA 2700081O15 gene	1.76
ENSMUSG00000012428	Steap4	STEAP family member 4	1.76
ENSMUSG00000050201	Otop2	otopetrin 2	1.76
ENSMUSG00000031996	Aplp2	amyloid beta (A4) precursor-like protein 2	1.75

ENSMUSG00000016382	Pls3	plastin 3 (T-isoform)	1.75
ENSMUSG00000038578	Susd1	sushi domain containing 1	1.75
ENSMUSG00000117123	AL805899.1	novel transcript	1.75
ENSMUSG00000073158	9030624G23Rik	RIKEN cDNA 9030624G23 gene	1.75
ENSMUSG00000015053	Gata2	GATA binding protein 2	1.75
ENSMUSG00000022996	Wnt10b	wingless-type MMTV integration site family, member 10B	1.75
ENSMUSG00000030223	Ptpro	protein tyrosine phosphatase, receptor type, O	1.74
ENSMUSG00000028347	Tmeff1	transmembrane protein with EGF-like and two follistatin-like domains 1	1.74
ENSMUSG00000024070	Prkd3	protein kinase D3	1.74
ENSMUSG00000024177	Nme4	NME/NM23 nucleoside diphosphate kinase 4	1.74
ENSMUSG00000020037	Rfx4	regulatory factor X, 4 (influences HLA class II expression)	1.74
ENSMUSG00000098068	Gm7909	predicted gene 7909	1.74
ENSMUSG00000051510	Mafg	v-maf musculoaponeurotic fibrosarcoma oncogene family, protein G (avian)	1.73
ENSMUSG00000057092	Fxyd3	FXYD domain-containing ion transport regulator 3	1.73
ENSMUSG00000038980	Rbbp8nl	RBBP8 N-terminal like	1.73
ENSMUSG00000032607	Amt	aminomethyltransferase	1.73
ENSMUSG00000024968	Rcor2	REST corepressor 2	1.73
ENSMUSG00000006931	P3h4	prolyl 3-hydroxylase family member 4 (non-enzymatic)	1.73
ENSMUSG00000031736	Crnde	colorectal neoplasia differentially expressed (non-protein coding)	1.73
ENSMUSG00000028541	B4galt2	UDP-Gal:betaGlcNAc beta 1,4- galactosyltransferase, polypeptide 2	1.73
ENSMUSG00000034177	Rnf43	ring finger protein 43	1.72
ENSMUSG00000047501	Cldn4	claudin 4	1.71
ENSMUSG00000028464	Tpm2	tropomyosin 2, beta	1.71
ENSMUSG00000033032	Afap1l1	actin filament associated protein 1-like 1	1.70
ENSMUSG00000042099	Kank3	KN motif and ankyrin repeat domains 3	1.70
ENSMUSG00000105990	Gm43307	predicted gene 43307	1.70
ENSMUSG00000031430	Vsig1	V-set and immunoglobulin domain containing 1	1.70
ENSMUSG00000022505	Emp2	epithelial membrane protein 2	1.69
ENSMUSG00000014592	Camta1	calmodulin binding transcription activator 1	1.69
ENSMUSG00000024535	Snx24	sorting nexing 24	1.69
ENSMUSG00000038594	Cep85l	centrosomal protein 85-like	1.69
ENSMUSG00000112084	Gm10773	predicted gene 10773	1.69
ENSMUSG00000004110	Cacna1e	calcium channel, voltage-dependent, R type, alpha 1E subunit	1.69
ENSMUSG00000060261	Gtf2i	general transcription factor II I	1.68
ENSMUSG00000000142	Axin2	axin 2	1.68
ENSMUSG00000025017	Pik3ap1	phosphoinositide-3-kinase adaptor protein 1	1.68
ENSMUSG00000048285	Frmd6	FERM domain containing 6	1.68
ENSMUSG00000037972	Snn	stannin	1.68
ENSMUSG00000027737	Slc7a11	solute carrier family 7 (cationic amino acid transporter, y+ system), member 11	1.68
ENSMUSG00000027346	Gpcpd1	glycerophosphocholine phosphodiesterase 1	1.67
ENSMUSG00000019843	Fyn	Fyn proto-oncogene	1.67
ENSMUSG00000034161	Scx	scleraxis	1.67
ENSMUSG00000050821	Fam131a	family with sequence similarity 131, member A	1.67
ENSMUSG00000029669	Tspan12	tetraspanin 12	1.66
ENSMUSG00000038295	Atg9b	autophagy related 9B	1.66
ENSMUSG00000054720	Lrrc8c	leucine rich repeat containing 8 family, member C	1.66
ENSMUSG00000059540	Tcea2	transcription elongation factor A (SII), 2	1.65
ENSMUSG00000030519	Apba2	amyloid beta (A4) precursor protein-binding, family A, member 2	1.65
ENSMUSG00000080198	Gm13573	predicted gene 13573	1.65
ENSMUSG00000087589	D430040D24Rik	RIKEN cDNA D430040D24 gene	1.65
ENSMUSG00000000957	Mmp14	matrix metallopeptidase 14 (membrane-inserted)	1.64
ENSMUSG00000020205	Phlda1	pleckstrin homology like domain, family A, member 1	1.64
ENSMUSG00000022475	Hdac7	histone deacetylase 7	1.64
ENSMUSG00000017390	Aldoc	aldolase C, fructose-bisphosphate	1.64
ENSMUSG00000076441	Ass1	argininosuccinate synthetase 1	1.64
ENSMUSG00000055407	Map6	microtubule-associated protein 6	1.64
ENSMUSG00000021539	Lect2	leukocyte cell-derived chemotaxin 2	1.64
ENSMUSG00000031633	Slc25a4	solute carrier family 25 (mitochondrial carrier, adenine nucleotide translocator), member 4	1.63
ENSMUSG00000031586	Rbpms	RNA binding protein gene with multiple splicing	1.63
ENSMUSG00000032606	Nicn1	nicolin 1	1.63
ENSMUSG00000049321	Zfp2	zinc finger protein 2	1.63
ENSMUSG00000026458	Ppfia4	protein tyrosine phosphatase, receptor type, f polypeptide (PTPRF), interacting protein (liprin), alpha	1.63
ENSMUSG00000006777	Krt23	keratin 23	1.62
ENSMUSG00000062937	Mtap	methylthioadenosine phosphorylase	1.62
ENSMUSG00000024524	Gnal	guanine nucleotide binding protein, alpha stimulating, olfactory type	1.62
ENSMUSG00000069874	Irgm2	immunity-related GTPase family M member 2	1.62
ENSMUSG00000090965	Gm17203	predicted gene 17203	1.62
ENSMUSG00000032232	Cgnl1	cingulin-like 1	1.61
ENSMUSG00000109727	Gm45464	predicted gene 45464	1.61
ENSMUSG00000001870	Ltbp1	latent transforming growth factor beta binding protein 1	1.61
ENSMUSG00000079330	Lemd1	LEM domain containing 1	1.60

ENSMUSG00000046743	Fat4	FAT atypical cadherin 4	1.60
ENSMUSG00000090399	Gm38399	predicted gene, 38399	1.60
ENSMUSG00000024109	Nrxn1	neurexin I	1.60
ENSMUSG00000091055	Siglec15	sialic acid binding Ig-like lectin 15	1.60
ENSMUSG00000032849	Abcc4	ATP-binding cassette, sub-family C (CFTR/MRP), member 4	1.59
ENSMUSG00000020453	Patz1	POZ (BTB) and AT hook containing zinc finger 1	1.59
ENSMUSG00000022433	Csnk1e	casein kinase 1, epsilon	1.58
ENSMUSG00000013033	Adgrl1	adhesion G protein-coupled receptor L1	1.58
ENSMUSG00000046718	Bst2	bone marrow stromal cell antigen 2	1.58
ENSMUSG00000000486	Sept1	septin 1	1.58
ENSMUSG00000038738	Shank1	SH3 and multiple ankyrin repeat domains 1	1.58
ENSMUSG00000058046	4933430I17Rik	RIKEN cDNA 4933430I17 gene	1.58
ENSMUSG00000044349	Snhg11	small nucleolar RNA host gene 11	1.58
ENSMUSG00000027570	Col9a3	collagen, type IX, alpha 3	1.57
ENSMUSG00000051735	Rinl	Ras and Rab interactor-like	1.57
ENSMUSG00000027485	Bpifb1	BPI fold containing family B, member 1	1.57
ENSMUSG00000036564	Ndrg4	N-myc downstream regulated gene 4	1.57
ENSMUSG00000019256	Ahr	aryl-hydrocarbon receptor	1.56
ENSMUSG00000082508	Rpl15-ps4	ribosomal protein L15, pseudogene 4	1.56
ENSMUSG00000049521	Cdc42ep1	CDC42 effector protein (Rho GTPase binding) 1	1.55
ENSMUSG00000006435	Neurl1a	neuralized E3 ubiquitin protein ligase 1A	1.55
ENSMUSG00000020032	Nuak1	NUAK family, SNF1-like kinase, 1	1.55
ENSMUSG00000103220	Gm37728	predicted gene, 37728	1.55
ENSMUSG00000019841	Rev3l	REV3 like, DNA directed polymerase zeta catalytic subunit	1.54
ENSMUSG00000035914	Cd276	CD276 antigen	1.54
ENSMUSG00000027784	Ppm1l	protein phosphatase 1 (formerly 2C)-like	1.54
ENSMUSG00000008450	Nutf2	nuclear transport factor 2	1.54
ENSMUSG00000018906	P4ha2	procollagen-proline, 2-oxoglutarate 4-dioxygenase (proline 4-hydroxylase), alpha II polypeptide	1.54
ENSMUSG00000110662	Gm45722	predicted gene 45722	1.54
ENSMUSG00000022995	Enah	ENAH actin regulator	1.54
ENSMUSG00000017493	Igfbp4	insulin-like growth factor binding protein 4	1.53
ENSMUSG00000032300	1700017B05Rik	RIKEN cDNA 1700017B05 gene	1.53
ENSMUSG00000054169	Ceacam10	carcinoembryonic antigen-related cell adhesion molecule 10	1.53
ENSMUSG00000072694	1500011B03Rik	RIKEN cDNA 1500011B03 gene	1.53
ENSMUSG00000050192	Eif5a2	eukaryotic translation initiation factor 5A2	1.53
ENSMUSG00000001025	S100a6	S100 calcium binding protein A6 (calcyclin)	1.52
ENSMUSG00000016534	Lamp2	lysosomal-associated membrane protein 2	1.52
ENSMUSG00000026383	Epb41l5	erythrocyte membrane protein band 4.1 like 5	1.52
ENSMUSG00000051359	Ncald	neurocalcin delta	1.52
ENSMUSG00000043068	Fam89a	family with sequence similarity 89, member A	1.52
ENSMUSG00000041592	Sdk2	sidekick cell adhesion molecule 2	1.52
ENSMUSG00000102278	Gm37145	predicted gene, 37145	1.52
ENSMUSG00000038872	Zfhx3	zinc finger homeobox 3	1.51
ENSMUSG00000016541	Atxn10	ataxin 10	1.51
ENSMUSG00000017376	Nlk	nemo like kinase	1.51
ENSMUSG00000090100	Ttbk2	tau tubulin kinase 2	1.51
ENSMUSG00000028601	Echdc2	enoyl Coenzyme A hydratase domain containing 2	1.51
ENSMUSG00000017897	Eya2	EYA transcriptional coactivator and phosphatase 2	1.51
ENSMUSG00000031292	Cdkl5	cyclin-dependent kinase-like 5	1.51
ENSMUSG00000047910	Pcdhb16	protocadherin beta 16	1.51
ENSMUSG00000047407	Tgif1	TGFB-induced factor homeobox 1	1.50
ENSMUSG00000039081	Zfp503	zinc finger protein 503	1.50
ENSMUSG00000001248	Gramd1a	GRAM domain containing 1A	1.50
ENSMUSG00000022780	Meltf	melanotransferrin	1.50
ENSMUSG00000027676	Ccdc39	coiled-coil domain containing 39	1.50
ENSMUSG00000013275	Slc41a1	solute carrier family 41, member 1	1.49
ENSMUSG00000048696	Mex3d	mex3 RNA binding family member D	1.49
ENSMUSG00000041025	Iffo2	intermediate filament family orphan 2	1.49
ENSMUSG00000069114	Zbtb10	zinc finger and BTB domain containing 10	1.49
ENSMUSG00000020546	Stxbp4	syntaxin binding protein 4	1.49
ENSMUSG00000020253	Ppm1m	protein phosphatase 1M	1.49
ENSMUSG00000096687	Mfsd4b4	major facilitator superfamily domain containing 4B4	1.49
ENSMUSG00000031660	Brd7	bromodomain containing 7	1.48
ENSMUSG00000045991	Onecut2	one cut domain, family member 2	1.48
ENSMUSG00000059895	Ptp4a3	protein tyrosine phosphatase 4a3	1.48
ENSMUSG00000022604	Cep97	centrosomal protein 97	1.48
ENSMUSG00000039683	Sdk1	sidekick cell adhesion molecule 1	1.48
ENSMUSG00000029093	Sorcs2	sortilin-related VPS10 domain containing receptor 2	1.47
ENSMUSG00000027907	S100a11	S100 calcium binding protein A11	1.47
ENSMUSG00000025577	Cbx2	chromobox 2	1.47
ENSMUSG00000028621	Cyb5rl	cytochrome b5 reductase-like	1.47

ENSMUSG00000020773	Trim47	tripartite motif-containing 47	1.47
ENSMUSG00000010751	Tnfrsf22	tumor necrosis factor receptor superfamily, member 22	1.47
ENSMUSG00000062151	Unc13c	unc-13 homolog C	1.47
ENSMUSG00000043065	Spice1	spindle and centriole associated protein 1	1.46
ENSMUSG00000020648	Dus4l	dihydrouridine synthase 4-like (S. cerevisiae)	1.46
ENSMUSG00000021108	Prkch	protein kinase C, eta	1.46
ENSMUSG00000071042	Rasgrp3	RAS, guanyl releasing protein 3	1.46
ENSMUSG00000007891	Ctsd	cathepsin D	1.45
ENSMUSG00000025268	Maged2	melanoma antigen, family D, 2	1.45
ENSMUSG00000058486	Wdr91	WD repeat domain 91	1.45
ENSMUSG00000002257	Def6	differentially expressed in FDCP 6	1.45
ENSMUSG00000030643	Rab30	RAB30, member RAS oncogene family	1.45
ENSMUSG00000038132	Rbm24	RNA binding motif protein 24	1.45
ENSMUSG00000052534	Pbx1	pre B cell leukemia homeobox 1	1.44
ENSMUSG00000028184	Adgrl2	adhesion G protein-coupled receptor L2	1.44
ENSMUSG00000030759	Far1	fatty acyl CoA reductase 1	1.44
ENSMUSG00000039384	Dusp10	dual specificity phosphatase 10	1.44
ENSMUSG00000032359	Ctsh	cathepsin H	1.44
ENSMUSG00000055639	Dach1	dachshund family transcription factor 1	1.43
ENSMUSG00000004032	Gstm5	glutathione S-transferase, mu 5	1.43
ENSMUSG00000095098	Ccdc85b	coiled-coil domain containing 85B	1.43
ENSMUSG00000024736	Tmem132a	transmembrane protein 132A	1.43
ENSMUSG00000103088	Pcdhgb6	protocadherin gamma subfamily B, 6	1.43
ENSMUSG00000028152	Tspan5	tetraspanin 5	1.42
ENSMUSG00000039801	Cplane1	ciliogenesis and planar polarity effector 1	1.42
ENSMUSG00000028542	Slc6a9	solute carrier family 6 (neurotransmitter transporter, glycine), member 9	1.42
ENSMUSG00000023348	Trip6	thyroid hormone receptor interactor 6	1.42
ENSMUSG00000030725	Lipt2	lipoyl(octanoyl) transferase 2 (putative)	1.42
ENSMUSG00000050295	Foxc1	forkhead box C1	1.41
ENSMUSG00000042694	Stn1	STN1, CST complex subunit	1.41
ENSMUSG00000024774	Ankrd22	ankyrin repeat domain 22	1.41
ENSMUSG00000031626	Sorbs2	sorbin and SH3 domain containing 2	1.40
ENSMUSG00000037936	Scarb1	scavenger receptor class B, member 1	1.40
ENSMUSG00000044927	H1fx	H1 histone family, member X	1.40
ENSMUSG00000047146	Tet1	tet methylcytosine dioxygenase 1	1.40
ENSMUSG00000034156	Tspoap1	TSPO associated protein 1	1.40
ENSMUSG00000029392	Rilpl1	Rab interacting lysosomal protein-like 1	1.40
ENSMUSG00000025145	Lrrc45	leucine rich repeat containing 45	1.39
ENSMUSG00000037664	Cdkn1c	cyclin-dependent kinase inhibitor 1C (P57)	1.39
ENSMUSG00000041886	Macc1	metastasis associated in colon cancer 1	1.39
ENSMUSG00000034854	Mfsd12	major facilitator superfamily domain containing 12	1.39
ENSMUSG00000024013	Fgd2	FYVE, RhoGEF and PH domain containing 2	1.39
ENSMUSG00000022055	Nefl	neurofilament, light polypeptide	1.39
ENSMUSG00000008090	Fgfrl1	fibroblast growth factor receptor-like 1	1.38
ENSMUSG00000006519	Cyba	cytochrome b-245, alpha polypeptide	1.38
ENSMUSG00000009418	Nav1	neuron navigator 1	1.38
ENSMUSG00000020650	Bcap29	B cell receptor associated protein 29	1.38
ENSMUSG00000033149	Phldb2	pleckstrin homology like domain, family B, member 2	1.38
ENSMUSG00000021792	Fam213a	family with sequence similarity 213, member A	1.38
ENSMUSG00000038797	Zscan2	zinc finger and SCAN domain containing 2	1.38
ENSMUSG00000027378	Nphp1	nephronophthisis 1 (juvenile) homolog (human)	1.38
ENSMUSG00000068758	Il3ra	interleukin 3 receptor, alpha chain	1.38
ENSMUSG00000000202	Btbd17	BTB (POZ) domain containing 17	1.38
ENSMUSG00000097787	2700046G09Rik	RIKEN cDNA 2700046G09 gene	1.38
ENSMUSG00000027997	Casp6	caspase 6	1.37
ENSMUSG00000037656	Slc20a2	solute carrier family 20, member 2	1.37
ENSMUSG00000040446	Rprd1a	regulation of nuclear pre-mRNA domain containing 1A	1.37
ENSMUSG00000032470	Mras	muscle and microspikes RAS	1.37
ENSMUSG00000038776	Ephx1	epoxide hydrolase 1, microsomal	1.37
ENSMUSG00000028573	Fggy	FGGY carbohydrate kinase domain containing	1.37
ENSMUSG00000028626	Col9a2	collagen, type IX, alpha 2	1.37
ENSMUSG00000040327	Cul9	cullin 9	1.36
ENSMUSG00000032609	Klhdc8b	kelch domain containing 8B	1.36
ENSMUSG00000025223	Ldb1	LIM domain binding 1	1.35
ENSMUSG00000021482	Aaed1	AhpC/TSA antioxidant enzyme domain containing 1	1.35
ENSMUSG00000033545	Znrf1	zinc and ring finger 1	1.35
ENSMUSG00000085972	1110028F11Rik	RIKEN cDNA 1110028F11 gene	1.35
ENSMUSG00000020388	Pdlim4	PDZ and LIM domain 4	1.35
ENSMUSG00000035615	Frmpd1	FERM and PDZ domain containing 1	1.35
ENSMUSG00000079414	Gm11110	predicted gene 11110	1.35
ENSMUSG00000029763	Exoc4	exocyst complex component 4	1.34

ENSMUSG00000023088	Abcc1	ATP-binding cassette, sub-family C (CFTR/MRP), member 1	1.34
ENSMUSG00000055485	Soga1	suppressor of glucose, autophagy associated 1	1.34
ENSMUSG00000036097	Slf2	SMC5-SMC6 complex localization factor 2	1.33
ENSMUSG00000025091	Pnliprp2	pancreatic lipase-related protein 2	1.33
ENSMUSG00000044468	Tent5c	terminal nucleotidyltransferase 5C	1.33
ENSMUSG00000048450	Msx1	msh homeobox 1	1.33
ENSMUSG00000059970	Hspa2	heat shock protein 2	1.33
ENSMUSG00000030284	Creld1	cysteine-rich with EGF-like domains 1	1.33
ENSMUSG00000019831	Wasf1	WAS protein family, member 1	1.33
ENSMUSG00000021013	Ttc8	tetratricopeptide repeat domain 8	1.33
ENSMUSG00000038663	Fsd2	fibronectin type III and SPRY domain containing 2	1.33
ENSMUSG00000058799	Nap1l1	nucleosome assembly protein 1-like 1	1.32
ENSMUSG00000009585	Apobec3	apolipoprotein B mRNA editing enzyme, catalytic polypeptide 3	1.32
ENSMUSG00000028838	Extl1	exostoses (multiple)-like 1	1.32
ENSMUSG00000028318	Polr1e	polymerase (RNA) I polypeptide E	1.32
ENSMUSG00000027189	Trim44	tripartite motif-containing 44	1.31
ENSMUSG00000028266	Lmo4	LIM domain only 4	1.31
ENSMUSG00000043439	Epop	elongin BC and polycomb repressive complex 2 associated protein	1.31
ENSMUSG00000014907	Naf1	nuclear assembly factor 1 ribonucleoprotein	1.31
ENSMUSG00000045288	Ush1g	USH1 protein network component sans	1.31
ENSMUSG00000047473	Zfp30	zinc finger protein 30	1.31
ENSMUSG00000027333	Smox	spermine oxidase	1.30
ENSMUSG00000026421	Csrp1	cysteine and glycine-rich protein 1	1.30
ENSMUSG00000032348	Gsta4	glutathione S-transferase, alpha 4	1.30
ENSMUSG00000035776	Cd99l2	CD99 antigen-like 2	1.30
ENSMUSG00000049871	Nlrc3	NLR family, CARD domain containing 3	1.30
ENSMUSG00000053931	Cnn3	calponin 3, acidic	1.29
ENSMUSG00000024896	Minpp1	multiple inositol polyphosphate histidine phosphatase 1	1.29
ENSMUSG00000032440	Tgfbr2	transforming growth factor, beta receptor II	1.29
ENSMUSG00000022096	Hr	hairless	1.29
ENSMUSG00000060591	Ifitm2	interferon induced transmembrane protein 2	1.29
ENSMUSG00000020376	Rnf130	ring finger protein 130	1.29
ENSMUSG00000025969	Nrp2	neuropilin 2	1.29
ENSMUSG00000034771	Tle2	transducin-like enhancer of split 2	1.29
ENSMUSG00000003190	Bcl2l12	BCL2-like 12 (proline rich)	1.29
ENSMUSG00000008734	Gprc5b	G protein-coupled receptor, family C, group 5, member B	1.29
ENSMUSG00000021720	Rnf180	ring finger protein 180	1.28
ENSMUSG00000004665	Cnn2	calponin 2	1.28
ENSMUSG00000068245	Phf11d	PHD finger protein 11D	1.28
ENSMUSG00000056486	Chn1	chimerin 1	1.28
ENSMUSG00000031342	Gpm6b	glycoprotein m6b	1.28
ENSMUSG00000059248	Sept9	septin 9	1.27
ENSMUSG00000018378	Cuedc1	CUE domain containing 1	1.27
ENSMUSG00000052852	Reep1	receptor accessory protein 1	1.27
ENSMUSG00000017314	Mpp2	membrane protein, palmitoylated 2 (MAGUK p55 subfamily member 2)	1.27
ENSMUSG00000097062	Gm17586	predicted gene, 17586	1.27
ENSMUSG00000084941	Gm11944	predicted gene 11944	1.27
ENSMUSG00000032238	Rora	RAR-related orphan receptor alpha	1.27
ENSMUSG00000034118	Tpst1	protein-tyrosine sulfotransferase 1	1.27
ENSMUSG00000035547	Capn5	calpain 5	1.26
ENSMUSG00000022390	Zc3h7b	zinc finger CCCH type containing 7B	1.26
ENSMUSG00000028832	Stmn1	stathmin 1	1.26
ENSMUSG00000034586	Hid1	HID1 domain containing	1.26
ENSMUSG00000026335	Pam	peptidylglycine alpha-amidating monooxygenase	1.26
ENSMUSG00000033319	Fem1c	fem-1 homolog c (C.elegans)	1.26
ENSMUSG00000068740	Celsr2	cadherin, EGF LAG seven-pass G-type receptor 2	1.26
ENSMUSG00000061981	Flot2	flotillin 2	1.26
ENSMUSG00000025212	Sfxn3	sideroflexin 3	1.26
ENSMUSG00000023495	Pcbp4	poly(rC) binding protein 4	1.26
ENSMUSG00000041836	Ptpre	protein tyrosine phosphatase, receptor type, E	1.25
ENSMUSG00000037287	Tbcel	tubulin folding cofactor E-like	1.25
ENSMUSG00000020776	Fbf1	Fas (TNFRSF6) binding factor 1	1.25
ENSMUSG00000037499	Nenf	neuron derived neurotrophic factor	1.25
ENSMUSG00000033039	Micall1	microtubule associated monooxygenase, calponin and LIM domain containing -like 1	1.24
ENSMUSG00000071662	Polr2g	polymerase (RNA) II (DNA directed) polypeptide G	1.24
ENSMUSG00000041135	Ripk2	receptor (TNFRSF)-interacting serine-threonine kinase 2	1.24
ENSMUSG00000021611	Tert	telomerase reverse transcriptase	1.24
ENSMUSG00000025887	Casp12	caspase 12	1.24
ENSMUSG00000044716	Dok7	docking protein 7	1.24
ENSMUSG00000013698	Pea15a	phosphoprotein enriched in astrocytes 15A	1.23
ENSMUSG00000020536	Llgl1	LLGL1 scribble cell polarity complex component	1.23

ENSMUSG00000025144	Cenpx	centromere protein X	1.23
ENSMUSG00000033900	Map9	microtubule-associated protein 9	1.23
ENSMUSG00000037890	Wdr19	WD repeat domain 19	1.23
ENSMUSG00000040187	Arntl2	aryl hydrocarbon receptor nuclear translocator-like 2	1.23
ENSMUSG00000009406	Elk1	ELK1, member of ETS oncogene family	1.23
ENSMUSG00000038463	Olfml2b	olfactomedin-like 2B	1.23
ENSMUSG00000030413	Pglyrp1	peptidoglycan recognition protein 1	1.22
ENSMUSG00000028378	Ptgr1	prostaglandin reductase 1	1.22
ENSMUSG00000020015	Cdk17	cyclin-dependent kinase 17	1.22
ENSMUSG00000032184	Lysmd2	LysM, putative peptidoglycan-binding, domain containing 2	1.22
ENSMUSG00000041540	Sox5	SRY (sex determining region Y)-box 5	1.22
ENSMUSG00000027878	Notch2	notch 2	1.21
ENSMUSG00000021506	Pitx1	paired-like homeodomain transcription factor 1	1.21
ENSMUSG00000038007	Acer2	alkaline ceramidase 2	1.21
ENSMUSG00000081683	Fzd10	frizzled class receptor 10	1.21
ENSMUSG00000083012	Fam220a	family with sequence similarity 220, member A	1.21
ENSMUSG00000020592	Sdc1	syndecan 1	1.20
ENSMUSG00000029229	Chic2	cysteine-rich hydrophobic domain 2	1.20
ENSMUSG00000046432	Bex3	brain expressed X-linked 3	1.20
ENSMUSG00000023031	Cela1	chymotrypsin-like elastase family, member 1	1.20
ENSMUSG00000034848	Ttc21b	tetratricopeptide repeat domain 21B	1.20
ENSMUSG00000046058	Eid2	EP300 interacting inhibitor of differentiation 2	1.20
ENSMUSG00000025153	Fasn	fatty acid synthase	1.19
ENSMUSG00000045211	Nudt18	nudix (nucleoside diphosphate linked moiety X)-type motif 18	1.19
ENSMUSG00000095325	Zfp870	zinc finger protein 870	1.19
ENSMUSG00000047749	Zc3hav1l	zinc finger CCCH-type, antiviral 1-like	1.19
ENSMUSG00000032657	Fam189b	family with sequence similarity 189, member B	1.19
ENSMUSG00000028836	Slc30a2	solute carrier family 30 (zinc transporter), member 2	1.18
ENSMUSG00000060510	Zfp266	zinc finger protein 266	1.18
ENSMUSG00000023055	Calcoco1	calcium binding and coiled coil domain 1	1.18
ENSMUSG00000049421	Zfp260	zinc finger protein 260	1.18
ENSMUSG00000022678	Nde1	nudE neurodevelopment protein 1	1.18
ENSMUSG00000020654	Adcy3	adenylate cyclase 3	1.18
ENSMUSG00000072621	Slfn10-ps	schlafen 10, pseudogene	1.18
ENSMUSG00000030315	Vgll4	vestigial like family member 4	1.17
ENSMUSG00000109324	Prmt1	protein arginine N-methyltransferase 1	1.17
ENSMUSG00000056648	Hoxb8	homeobox B8	1.17
ENSMUSG00000021466	Ptch1	patched 1	1.17
ENSMUSG00000000861	Bcl11a	B cell CLL/lymphoma 11A (zinc finger protein)	1.17
ENSMUSG00000040711	Sh3pxd2b	SH3 and PX domains 2B	1.17
ENSMUSG00000041096	Tspyl2	TSPY-like 2	1.17
ENSMUSG00000039831	Arhgap29	Rho GTPase activating protein 29	1.17
ENSMUSG00000050910	Cdr2l	cerebellar degeneration-related protein 2-like	1.17
ENSMUSG00000039236	Isg20	interferon-stimulated protein	1.17
ENSMUSG00000039262	Prrc2b	proline-rich coiled-coil 2B	1.16
ENSMUSG00000030729	Pgm2l1	phosphoglucomutase 2-like 1	1.16
ENSMUSG00000026425	Srgap2	SLIT-ROBO Rho GTPase activating protein 2	1.16
ENSMUSG00000025534	Gusb	glucuronidase, beta	1.16
ENSMUSG00000045045	Lrfn4	leucine rich repeat and fibronectin type III domain containing 4	1.16
ENSMUSG00000039976	Tbc1d16	TBC1 domain family, member 16	1.16
ENSMUSG00000010080	Epn3	epsin 3	1.16
ENSMUSG00000040543	Pitpnm3	PITPNM family member 3	1.16
ENSMUSG00000035164	Zc3h12c	zinc finger CCCH type containing 12C	1.16
ENSMUSG00000031207	Msn	moesin	1.16
ENSMUSG00000053110	Yap1	yes-associated protein 1	1.15
ENSMUSG00000049577	Zfpm1	zinc finger protein, multitype 1	1.15
ENSMUSG00000034981	Parm1	prostate androgen-regulated mucin-like protein 1	1.15
ENSMUSG00000025323	Sp4	trans-acting transcription factor 4	1.15
ENSMUSG00000031502	Col4a1	collagen, type IV, alpha 1	1.15
ENSMUSG00000033906	Zdhhc15	zinc finger, DHHC domain containing 15	1.14
ENSMUSG00000034723	Tmx4	thioredoxin-related transmembrane protein 4	1.14
ENSMUSG00000036006	Ripor2	RHO family interacting cell polarization regulator 2	1.14
ENSMUSG00000031919	Tmed6	transmembrane p24 trafficking protein 6	1.14
ENSMUSG00000022912	Pros1	protein S (alpha)	1.14
ENSMUSG00000064326	Siva1	SIVA1, apoptosis-inducing factor	1.14
ENSMUSG00000022895	Ets2	E26 avian leukemia oncogene 2, 3' domain	1.13
ENSMUSG00000019966	Kitl	kit ligand	1.13
ENSMUSG00000048371	Pdp2	pyruvate dehyrogenase phosphatase catalytic subunit 2	1.13
ENSMUSG00000040669	Phc1	polyhomeotic 1	1.13
ENSMUSG00000019461	Plscr3	phospholipid scramblase 3	1.13
ENSMUSG00000026727	Rsu1	Ras suppressor protein 1	1.13

ENSMUSG00000027684	Mecom	MDS1 and EVI1 complex locus	1.12
ENSMUSG00000084939	Gm830	predicted gene 830	1.12
ENSMUSG00000018199	Trove2	TROVE domain family, member 2	1.12
ENSMUSG00000057156	Homez	homeodomain leucine zipper-encoding gene	1.12
ENSMUSG00000069631	Strada	STE20-related kinase adaptor alpha	1.12
ENSMUSG00000038387	Rras	related RAS viral (r-ras) oncogene	1.12
ENSMUSG00000049791	Fzd4	frizzled class receptor 4	1.12
ENSMUSG00000002489	Tiam1	T cell lymphoma invasion and metastasis 1	1.11
ENSMUSG00000022708	Zbtb20	zinc finger and BTB domain containing 20	1.11
ENSMUSG00000059552	Trp53	transformation related protein 53	1.11
ENSMUSG00000021262	Evl	Ena-vasodilator stimulated phosphoprotein	1.11
ENSMUSG00000025986	Slc39a10	solute carrier family 39 (zinc transporter), member 10	1.11
ENSMUSG00000000325	Arvcf	armadillo repeat gene deleted in velocardiofacial syndrome	1.11
ENSMUSG00000033282	Rpgrip1l	Rpgrip1-like	1.11
ENSMUSG00000021589	Rhobtb3	Rho-related BTB domain containing 3	1.11
ENSMUSG00000021003	Galc	galactosylceramidase	1.11
ENSMUSG00000027995	Tlr2	toll-like receptor 2	1.11
ENSMUSG00000023947	Nfkbie	nuclear factor of kappa light polypeptide gene enhancer in B cells inhibitor, epsilon	1.11
ENSMUSG00000115902	AC113595.1	novel transcript, antisense to Apobec3	1.11
ENSMUSG00000020167	Tcf3	transcription factor 3	1.10
ENSMUSG00000029246	Ppat	phosphoribosyl pyrophosphate amidotransferase	1.10
ENSMUSG00000028245	Nsmaf	neutral sphingomyelinase (N-SMase) activation associated factor	1.09
ENSMUSG00000040466	Blvrb	biliverdin reductase B (flavin reductase (NADPH))	1.09
ENSMUSG00000058672	Tubb2a	tubulin, beta 2A class IIA	1.09
ENSMUSG00000044469	Tnfaip8l1	tumor necrosis factor, alpha-induced protein 8-like 1	1.09
ENSMUSG00000089809	Rasgef1b	RasGEF domain family, member 1B	1.09
ENSMUSG00000036052	Dnajb5	DnaJ heat shock protein family (Hsp40) member B5	1.09
ENSMUSG00000028576	Ift74	intraflagellar transport 74	1.09
ENSMUSG00000027490	E2f1	E2F transcription factor 1	1.09
ENSMUSG00000040681	Hmgn1	high mobility group nucleosomal binding domain 1	1.08
ENSMUSG00000041238	Rbbp8	retinoblastoma binding protein 8, endonuclease	1.08
ENSMUSG00000015217	Hmgb3	high mobility group box 3	1.08
ENSMUSG00000020393	Kremen1	kringle containing transmembrane protein 1	1.08
ENSMUSG00000046668	Cxxc5	CXXC finger 5	1.08
ENSMUSG00000049939	Lrrc4	leucine rich repeat containing 4	1.08
ENSMUSG00000032068	Plet1	placenta expressed transcript 1	1.08
ENSMUSG00000049300	Prmt6	protein arginine N-methyltransferase 6	1.08
ENSMUSG00000097431	Gm26782	predicted gene, 26782	1.08
ENSMUSG00000048232	Fbxo10	F-box protein 10	1.08
ENSMUSG00000035451	Foxa1	forkhead box A1	1.07
ENSMUSG00000021068	Nin	ninein	1.07
ENSMUSG00000041308	Sntb2	syntrophin, basic 2	1.07
ENSMUSG00000070305	Mpzl3	myelin protein zero-like 3	1.07
ENSMUSG00000048924	Ccdc125	coiled-coil domain containing 125	1.07
ENSMUSG00000022442	Ttll1	tubulin tyrosine ligase-like 1	1.07
ENSMUSG00000031799	Tpm4	tropomyosin 4	1.06
ENSMUSG00000026796	Fam129b	family with sequence similarity 129, member B	1.06
ENSMUSG00000022231	Sema5a	sema domain, seven thrombospondin repeats (type 1 and type 1-like), transmembrane domain (TM) a	1.06
ENSMUSG00000052117	D630039A03Rik	RIKEN cDNA D630039A03 gene	1.06
ENSMUSG00000006369	Fbln1	fibulin 1	1.06
ENSMUSG00000029999	Tgfa	transforming growth factor alpha	1.06
ENSMUSG00000040596	Pogk	pogo transposable element with KRAB domain	1.06
ENSMUSG00000040717	Il17rd	interleukin 17 receptor D	1.06
ENSMUSG00000018547	Pip4k2b	phosphatidylinositol-5-phosphate 4-kinase, type II, beta	1.06
ENSMUSG00000055945	Prr18	proline rich 18	1.06
ENSMUSG00000034064	Poglut1	protein O-glucosyltransferase 1	1.06
ENSMUSG00000038205	Prkab2	protein kinase, AMP-activated, beta 2 non-catalytic subunit	1.06
ENSMUSG00000032481	Smarcc1	SWI/SNF related, matrix associated, actin dependent regulator of chromatin, subfamily c, member 1	1.05
ENSMUSG00000042506	Usp22	ubiquitin specific peptidase 22	1.05
ENSMUSG00000026509	Capn2	calpain 2	1.05
ENSMUSG00000029055	Plch2	phospholipase C, eta 2	1.05
ENSMUSG00000019433	Gipc1	GIPC PDZ domain containing family, member 1	1.05
ENSMUSG00000058070	Eml1	echinoderm microtubule associated protein like 1	1.05
ENSMUSG00000002308	Cd320	CD320 antigen	1.05
ENSMUSG00000074876	Spata5l1	spermatogenesis associated 5-like 1	1.05
ENSMUSG00000009614	Sardh	sarcosine dehydrogenase	1.05
ENSMUSG00000033965	Slc16a2	solute carrier family 16 (monocarboxylic acid transporters), member 2	1.05
ENSMUSG00000057133	Chd6	chromodomain helicase DNA binding protein 6	1.04
ENSMUSG00000030342	Cd9	CD9 antigen	1.04
ENSMUSG00000053580	Tanc2	tetratricopeptide repeat, ankyrin repeat and coiled-coil containing 2	1.04
ENSMUSG00000025151	Maged1	melanoma antigen, family D, 1	1.04

ENSMUSG00000005034	Prkacb	protein kinase, cAMP dependent, catalytic, beta	1.04
ENSMUSG00000031360	Ctps2	cytidine 5'-triphosphate synthase 2	1.04
ENSMUSG00000024501	Dpysl3	dihydropyrimidinase-like 3	1.04
ENSMUSG00000046380	Jrk	jerky	1.04
ENSMUSG00000036368	Rmdn2	regulator of microtubule dynamics 2	1.04
ENSMUSG00000037706	Cd81	CD81 antigen	1.03
ENSMUSG00000022828	Gtf2e1	general transcription factor II E, polypeptide 1 (alpha subunit)	1.03
ENSMUSG00000040785	Ttc3	tetratricopeptide repeat domain 3	1.02
ENSMUSG00000040274	Cdk6	cyclin-dependent kinase 6	1.02
ENSMUSG00000028849	Map7d1	MAP7 domain containing 1	1.02
ENSMUSG00000028044	Cks1b	CDC28 protein kinase 1b	1.02
ENSMUSG00000032228	Tcf12	transcription factor 12	1.01
ENSMUSG00000051391	Ywhag	tyrosine 3-monooxygenase/tryptophan 5-monooxygenase activation protein, gamma polypeptide	1.01
ENSMUSG00000041688	Amot	angiomotin	1.01
ENSMUSG00000050912	Tmem123	transmembrane protein 123	1.01
ENSMUSG00000054520	Sh3bp2	SH3-domain binding protein 2	1.01
ENSMUSG00000049532	Sall2	spalt like transcription factor 2	1.01
ENSMUSG00000030980	Knop1	lysine rich nucleolar protein 1	1.00
ENSMUSG00000002107	Celf2	CUGBP, Elav-like family member 2	1.00
ENSMUSG00000018569	Cldn7	claudin 7	-1.00
ENSMUSG00000001089	Luzp1	leucine zipper protein 1	-1.00
ENSMUSG00000054277	Arfgap3	ADP-ribosylation factor GTPase activating protein 3	-1.00
ENSMUSG00000021948	Prkcd	protein kinase C, delta	-1.00
ENSMUSG00000019969	Psen1	presenilin 1	-1.00
ENSMUSG00000040188	Scamp2	secretory carrier membrane protein 2	-1.00
ENSMUSG00000041577	Prelp	proline arginine-rich end leucine-rich repeat	-1.00
ENSMUSG00000030499	Kctd15	potassium channel tetramerisation domain containing 15	-1.00
ENSMUSG00000037400	Atp11b	ATPase, class VI, type 11B	-1.01
ENSMUSG00000027957	Slc35a3	solute carrier family 35 (UDP-N-acetylglucosamine (UDP-GlcNAc) transporter), member 3	-1.01
ENSMUSG00000034435	Tmem30b	transmembrane protein 30B	-1.01
ENSMUSG00000024036	Slc37a1	solute carrier family 37 (glycerol-3-phosphate transporter), member 1	-1.01
ENSMUSG00000035064	Eef2k	eukaryotic elongation factor-2 kinase	-1.01
ENSMUSG00000053898	Ech1	enoyl coenzyme A hydratase 1, peroxisomal	-1.01
ENSMUSG00000053886	Sh2d4a	SH2 domain containing 4A	-1.01
ENSMUSG00000033318	Gstt2	glutathione S-transferase, theta 2	-1.01
ENSMUSG00000041845	Rhod	ras homolog family member D	-1.01
ENSMUSG00000057137	Tmem140	transmembrane protein 140	-1.01
ENSMUSG00000023328	Ache	acetylcholinesterase	-1.01
ENSMUSG00000070780	Rbm47	RNA binding motif protein 47	-1.02
ENSMUSG00000040659	Efhd2	EF hand domain containing 2	-1.02
ENSMUSG00000019302	Atp6v0a1	ATPase, H+ transporting, lysosomal V0 subunit A1	-1.02
ENSMUSG00000074305	Peak1	pseudopodium-enriched atypical kinase 1	-1.02
ENSMUSG00000061313	Ddhd2	DDHD domain containing 2	-1.02
ENSMUSG00000039349	C130074G19Rik	RIKEN cDNA C130074G19 gene	-1.02
ENSMUSG00000015536	Mocs2	molybdenum cofactor synthesis 2	-1.02
ENSMUSG00000037709	Fam13a	family with sequence similarity 13, member A	-1.02
ENSMUSG00000028885	Smpdl3b	sphingomyelin phosphodiesterase, acid-like 3B	-1.02
ENSMUSG00000043085	Tmem82	transmembrane protein 82	-1.02
ENSMUSG00000000386	Mx1	MX dynamin-like GTPase 1	-1.02
ENSMUSG00000000631	Myo18a	myosin XVIIIA	-1.03
ENSMUSG00000024052	Lpin2	lipin 2	-1.03
ENSMUSG00000020451	Limk2	LIM motif-containing protein kinase 2	-1.03
ENSMUSG00000022973	Synj1	synaptojanin 1	-1.03
ENSMUSG00000020803	Txndc17	thioredoxin domain containing 17	-1.03
ENSMUSG00000055116	Arntl	aryl hydrocarbon receptor nuclear translocator-like	-1.03
ENSMUSG00000074653	Lrrc31	leucine rich repeat containing 31	-1.03
ENSMUSG00000002346	Slc25a42	solute carrier family 25, member 42	-1.03
ENSMUSG00000027422	Rrbp1	ribosome binding protein 1	-1.04
ENSMUSG00000046447	Camk2n1	calcium/calmodulin-dependent protein kinase II inhibitor 1	-1.04
ENSMUSG00000053965	Pde5a	phosphodiesterase 5A, cGMP-specific	-1.04
ENSMUSG00000027215	Cd82	CD82 antigen	-1.04
ENSMUSG00000028413	B4galt1	UDP-Gal:betaGlcNAc beta 1,4- galactosyltransferase, polypeptide 1	-1.04
ENSMUSG00000032741	Tpcn1	two pore channel 1	-1.04
ENSMUSG00000074447	Defa21	defensin, alpha, 21	-1.04
ENSMUSG00000063206	Defa34	defensin, alpha, 34	-1.04
ENSMUSG00000026784	Pdss1	prenyl (solanesyl) diphosphate synthase, subunit 1	-1.04
ENSMUSG00000046794	Ppp1r3b	protein phosphatase 1, regulatory subunit 3B	-1.04
ENSMUSG00000039958	Etfbkmt	electron transfer flavoprotein beta subunit lysine methyltransferase	-1.04
ENSMUSG00000001300	Efnb2	ephrin B2	-1.05
ENSMUSG00000044786	Zfp36	zinc finger protein 36	-1.05
ENSMUSG00000046876	Atxn1	ataxin 1	-1.05

ENSMUSG00000034422	Parp14	poly (ADP-ribose) polymerase family, member 14	-1.05
ENSMUSG00000034330	Plcg2	phospholipase C, gamma 2	-1.05
ENSMUSG00000031853	Map3k21	mitogen-activated protein kinase kinase kinase 21	-1.05
ENSMUSG00000028480	Glipr2	GLI pathogenesis-related 2	-1.05
ENSMUSG00000032369	Plscr1	phospholipid scramblase 1	-1.05
ENSMUSG00000031639	Tlr3	toll-like receptor 3	-1.05
ENSMUSG00000056313	Tcim	transcriptional and immune response regulator	-1.05
ENSMUSG00000046352	Gjb2	gap junction protein, beta 2	-1.05
ENSMUSG00000038459	Abhd17c	abhydrolase domain containing 17C	-1.06
ENSMUSG00000035382	Pcsk7	proprotein convertase subtilisin/kexin type 7	-1.06
ENSMUSG00000041625	Ggact	gamma-glutamylamine cyclotransferase	-1.06
ENSMUSG00000027605	Acss2	acyl-CoA synthetase short-chain family member 2	-1.06
ENSMUSG00000022768	Ccdc116	coiled-coil domain containing 116	-1.06
ENSMUSG00000034271	Jdp2	Jun dimerization protein 2	-1.06
ENSMUSG00000042647	Acad12	acyl-Coenzyme A dehydrogenase family, member 12	-1.06
ENSMUSG00000038393	Txnip	thioredoxin interacting protein	-1.07
ENSMUSG00000021866	Anxa11	annexin A11	-1.07
ENSMUSG00000020134	Peli1	pellino 1	-1.07
ENSMUSG00000003545	Fosb	FBJ osteosarcoma oncogene B	-1.07
ENSMUSG00000034613	Ppm1h	protein phosphatase 1H (PP2C domain containing)	-1.07
ENSMUSG00000026360	Rgs2	regulator of G-protein signaling 2	-1.07
ENSMUSG00000034640	Tiparp	TCDD-inducible poly(ADP-ribose) polymerase	-1.07
ENSMUSG00000052776	Oas1a	2'-5' oligoadenylate synthetase 1A	-1.07
ENSMUSG00000067199	Frat1	frequently rearranged in advanced T cell lymphomas	-1.07
ENSMUSG00000046733	Gprc5a	G protein-coupled receptor, family C, group 5, member A	-1.07
ENSMUSG00000042750	Bex2	brain expressed X-linked 2	-1.07
ENSMUSG00000069833	Ahnak	AHNAK nucleoprotein (desmoyokin)	-1.08
ENSMUSG00000041870	Ankrd13a	ankyrin repeat domain 13a	-1.08
ENSMUSG00000038023	Atp6v0a2	ATPase, H+ transporting, lysosomal V0 subunit A2	-1.08
ENSMUSG00000026110	Mgat4a	mannoside acetylglucosaminyltransferase 4, isoenzyme A	-1.08
ENSMUSG00000022949	Clic6	chloride intracellular channel 6	-1.08
ENSMUSG00000090946	Ccdc71l	coiled-coil domain containing 71 like	-1.08
ENSMUSG00000066258	Trim12a	tripartite motif-containing 12A	-1.08
ENSMUSG00000031712	Il15	interleukin 15	-1.08
ENSMUSG00000057778	Cyb5d2	cytochrome b5 domain containing 2	-1.08
ENSMUSG00000103696	Gm37531	predicted gene, 37531	-1.08
ENSMUSG00000056185	Snx32	sorting nexin 32	-1.08
ENSMUSG00000032601	Prkar2a	protein kinase, cAMP dependent regulatory, type II alpha	-1.09
ENSMUSG00000030313	Dennd5b	DENN/MADD domain containing 5B	-1.09
ENSMUSG00000038967	Pdk2	pyruvate dehydrogenase kinase, isoenzyme 2	-1.09
ENSMUSG00000031156	Slc35a2	solute carrier family 35 (UDP-galactose transporter), member A2	-1.09
ENSMUSG00000021185	Dglucy	D-glutamate cyclase	-1.09
ENSMUSG00000024193	Phf1	PHD finger protein 1	-1.09
ENSMUSG00000026189	Pecr	peroxisomal trans-2-enoyl-CoA reductase	-1.09
ENSMUSG00000103711	Tstd1	thiosulfate sulfurtransferase (rhodanese)-like domain containing 1	-1.09
ENSMUSG00000109438	Gm45073	predicted gene 45073	-1.09
ENSMUSG00000056429	Tgoln1	trans-golgi network protein	-1.10
ENSMUSG00000038859	Baiap2l1	BAI1-associated protein 2-like 1	-1.10
ENSMUSG00000023087	Noct	nocturnin	-1.10
ENSMUSG00000032306	Mpi	mannose phosphate isomerase	-1.10
ENSMUSG00000049422	Chchd10	coiled-coil-helix-coiled-coil-helix domain containing 10	-1.10
ENSMUSG00000048970	C1galt1c1	C1GALT1-specific chaperone 1	-1.10
ENSMUSG00000028538	St3gal3	ST3 beta-galactoside alpha-2,3-sialyltransferase 3	-1.10
ENSMUSG00000034265	Zdhhc14	zinc finger, DHHC domain containing 14	-1.10
ENSMUSG00000038422	Hdhd3	haloacid dehalogenase-like hydrolase domain containing 3	-1.10
ENSMUSG00000019370	Calm3	calmodulin 3	-1.11
ENSMUSG00000028830	AU040320	expressed sequence AU040320	-1.11
ENSMUSG00000030494	Rhpn2	rhophilin, Rho GTPase binding protein 2	-1.11
ENSMUSG00000031090	Nadsyn1	NAD synthetase 1	-1.11
ENSMUSG00000028521	Slc35d1	solute carrier family 35 (UDP-glucuronic acid/UDP-N-acetylgalactosamine dual transporter), member	-1.11
ENSMUSG00000040613	Apobec1	apolipoprotein B mRNA editing enzyme, catalytic polypeptide 1	-1.11
ENSMUSG00000042251	Pm20d1	peptidase M20 domain containing 1	-1.11
ENSMUSG00000040957	Cables1	CDK5 and Abl enzyme substrate 1	-1.11
ENSMUSG00000026271	Gpr35	G protein-coupled receptor 35	-1.11
ENSMUSG00000068874	Selenbp1	selenium binding protein 1	-1.11
ENSMUSG00000050854	Tmem125	transmembrane protein 125	-1.11
ENSMUSG00000007817	Zmiz1	zinc finger, MIZ-type containing 1	-1.12
ENSMUSG00000020827	Mink1	misshapen-like kinase 1 (zebrafish)	-1.12
ENSMUSG00000070002	Ell	elongation factor RNA polymerase II	-1.12
ENSMUSG00000042363	Lgalsl	lectin, galactoside binding-like	-1.12
ENSMUSG00000054252	Fgfr3	fibroblast growth factor receptor 3	-1.12

ENSMUSG00000042073	Abhd14b	abhydrolase domain containing 14b	-1.12
ENSMUSG00000029735	Tpk1	thiamine pyrophosphokinase	-1.12
ENSMUSG00000021709	Erbin	Erbb2 interacting protein	-1.13
ENSMUSG00000024378	Stard4	StAR-related lipid transfer (START) domain containing 4	-1.13
ENSMUSG00000005580	Adcy9	adenylate cyclase 9	-1.13
ENSMUSG00000026956	Uap1l1	UDP-N-acteylglucosamine pyrophosphorylase 1-like 1	-1.13
ENSMUSG00000106375	Gm43361	predicted gene 43361	-1.13
ENSMUSG00000030545	Pex11a	peroxisomal biogenesis factor 11 alpha	-1.13
ENSMUSG00000029154	Cwh43	cell wall biogenesis 43 C-terminal homolog	-1.13
ENSMUSG00000020777	Acox1	acyl-Coenzyme A oxidase 1, palmitoyl	-1.14
ENSMUSG00000025068	Gsto1	glutathione S-transferase omega 1	-1.14
ENSMUSG00000092274	Neat1	nuclear paraspeckle assembly transcript 1 (non-protein coding)	-1.14
ENSMUSG00000024431	Nr3c1	nuclear receptor subfamily 3, group C, member 1	-1.14
ENSMUSG00000009828	Ick	intestinal cell kinase	-1.14
ENSMUSG00000048707	Tprn	taperin	-1.14
ENSMUSG00000036918	Ttc7	tetratricopeptide repeat domain 7	-1.14
ENSMUSG00000029102	Hgfac	hepatocyte growth factor activator	-1.14
ENSMUSG00000040631	Dok4	docking protein 4	-1.14
ENSMUSG00000028223	Decr1	2,4-dienoyl CoA reductase 1, mitochondrial	-1.14
ENSMUSG00000041143	Tmco4	transmembrane and coiled-coil domains 4	-1.14
ENSMUSG00000105071	Gm43336	predicted gene 43336	-1.14
ENSMUSG00000048442	Smim5	small integral membrane protein 5	-1.14
ENSMUSG00000034579	Pla2g3	phospholipase A2, group III	-1.14
ENSMUSG00000061718	Ppp1r1b	protein phosphatase 1, regulatory inhibitor subunit 1B	-1.15
ENSMUSG00000037434	Slc30a1	solute carrier family 30 (zinc transporter), member 1	-1.15
ENSMUSG00000022750	Klhl22	kelch-like 22	-1.15
ENSMUSG00000038291	Snx25	sorting nexin 25	-1.15
ENSMUSG00000015222	Map2	microtubule-associated protein 2	-1.15
ENSMUSG00000006641	Slc5a6	solute carrier family 5 (sodium-dependent vitamin transporter), member 6	-1.15
ENSMUSG00000050914	Ankrd37	ankyrin repeat domain 37	-1.15
ENSMUSG00000103948	4930594C11Rik	RIKEN cDNA 4930594C11 gene	-1.15
ENSMUSG00000041757	Plekha6	pleckstrin homology domain containing, family A member 6	-1.16
ENSMUSG00000020986	Sec23a	SEC23 homolog A, COPII coat complex component	-1.16
ENSMUSG00000021550	2210016F16Rik	RIKEN cDNA 2210016F16 gene	-1.16
ENSMUSG00000022221	Ripk3	receptor-interacting serine-threonine kinase 3	-1.16
ENSMUSG00000022090	Pdlim2	PDZ and LIM domain 2	-1.16
ENSMUSG00000029449	Rhof	ras homolog family member F (in filopodia)	-1.16
ENSMUSG00000022469	Rapgef3	Rap guanine nucleotide exchange factor (GEF) 3	-1.16
ENSMUSG00000017453	Pipox	pipecolic acid oxidase	-1.16
ENSMUSG00000021751	Acox2	acyl-Coenzyme A oxidase 2, branched chain	-1.16
ENSMUSG00000026131	Dst	dystonin	-1.17
ENSMUSG00000019866	Crybg1	crystallin beta-gamma domain containing 1	-1.17
ENSMUSG00000029032	Arhgef16	Rho guanine nucleotide exchange factor (GEF) 16	-1.17
ENSMUSG00000038781	Stap2	signal transducing adaptor family member 2	-1.17
ENSMUSG00000005802	Slc30a4	solute carrier family 30 (zinc transporter), member 4	-1.17
ENSMUSG00000025477	Inpp5a	inositol polyphosphate-5-phosphatase A	-1.17
ENSMUSG00000070730	Rmdn3	regulator of microtubule dynamics 3	-1.17
ENSMUSG00000016495	Plgrkt	plasminogen receptor, C-terminal lysine transmembrane protein	-1.17
ENSMUSG00000021587	Pcsk1	proprotein convertase subtilisin/kexin type 1	-1.17
ENSMUSG00000045679	Pqlc3	PQ loop repeat containing	-1.17
ENSMUSG00000043760	Pkhd1	polycystic kidney and hepatic disease 1	-1.17
ENSMUSG00000022565	Plec	plectin	-1.18
ENSMUSG00000024388	Myo7b	myosin VIIB	-1.18
ENSMUSG00000041959	S100a10	S100 calcium binding protein A10 (calpactin)	-1.18
ENSMUSG00000063952	Brpf3	bromodomain and PHD finger containing, 3	-1.18
ENSMUSG00000020173	Cobl	cordon-bleu WH2 repeat	-1.18
ENSMUSG00000039145	Camk1d	calcium/calmodulin-dependent protein kinase ID	-1.18
ENSMUSG00000031391	L1cam	L1 cell adhesion molecule	-1.18
ENSMUSG00000022683	Pla2g10	phospholipase A2, group X	-1.18
ENSMUSG00000027984	Hadh	hydroxyacyl-Coenzyme A dehydrogenase	-1.19
ENSMUSG00000038244	Mical2	microtubule associated monooxygenase, calponin and LIM domain containing 2	-1.19
ENSMUSG00000047123	Ticam1	toll-like receptor adaptor molecule 1	-1.19
ENSMUSG00000024112	Cacna1h	calcium channel, voltage-dependent, T type, alpha 1H subunit	-1.19
ENSMUSG00000033902	Mapkbp1	mitogen-activated protein kinase binding protein 1	-1.19
ENSMUSG00000069804	Gm10277	predicted gene 10277	-1.19
ENSMUSG00000069227	Gprin1	G protein-regulated inducer of neurite outgrowth 1	-1.19
ENSMUSG00000013418	B4galnt2	beta-1,4-N-acetyl-galactosaminyl transferase 2	-1.20
ENSMUSG00000064210	Ano6	anoctamin 6	-1.20
ENSMUSG00000032737	Inppl1	inositol polyphosphate phosphatase-like 1	-1.20
ENSMUSG00000017801	Mlx	MAX-like protein X	-1.20
ENSMUSG00000054733	Msra	methionine sulfoxide reductase A	-1.20

ENSMUSG00000042797	Aqp11	aquaporin 11	-1.20
ENSMUSG00000039747	Orai2	ORAI calcium release-activated calcium modulator 2	-1.20
ENSMUSG00000022680	Pdxdc1	pyridoxal-dependent decarboxylase domain containing 1	-1.21
ENSMUSG00000022946	Dopey2	dopey family member 2	-1.21
ENSMUSG00000039193	Nlrc4	NLR family, CARD domain containing 4	-1.21
ENSMUSG00000027938	Creb3l4	cAMP responsive element binding protein 3-like 4	-1.21
ENSMUSG00000074218	Cox7a1	cytochrome c oxidase subunit 7A1	-1.21
ENSMUSG00000020377	Ltc4s	leukotriene C4 synthase	-1.21
ENSMUSG00000033161	Atp1a1	ATPase, Na+/K+ transporting, alpha 1 polypeptide	-1.22
ENSMUSG00000021876	Rnase4	ribonuclease, RNase A family 4	-1.22
ENSMUSG00000039682	Lap3	leucine aminopeptidase 3	-1.22
ENSMUSG00000050931	Sgms2	sphingomyelin synthase 2	-1.22
ENSMUSG00000022265	Ank	progressive ankylosis	-1.22
ENSMUSG00000039166	Akap7	A kinase (PRKA) anchor protein 7	-1.22
ENSMUSG00000029167	Ppargc1a	peroxisome proliferative activated receptor, gamma, coactivator 1 alpha	-1.22
ENSMUSG00000059323	Tonsl	tonsoku-like, DNA repair protein	-1.22
ENSMUSG00000031378	Abcd1	ATP-binding cassette, sub-family D (ALD), member 1	-1.22
ENSMUSG00000029471	Camkk2	calcium/calmodulin-dependent protein kinase kinase 2, beta	-1.22
ENSMUSG00000054200	Ffar4	free fatty acid receptor 4	-1.22
ENSMUSG00000001227	Sema6b	sema domain, transmembrane domain (TM), and cytoplasmic domain, (semaphorin) 6B	-1.22
ENSMUSG00000035441	Myo1d	myosin ID	-1.23
ENSMUSG00000020841	Cpd	carboxypeptidase D	-1.23
ENSMUSG00000027007	Ssfa2	sperm specific antigen 2	-1.23
ENSMUSG00000038260	Trpm4	transient receptor potential cation channel, subfamily M, member 4	-1.23
ENSMUSG00000036499	Eea1	early endosome antigen 1	-1.23
ENSMUSG00000028894	Inpp5b	inositol polyphosphate-5-phosphatase B	-1.23
ENSMUSG00000015342	Xk	X-linked Kx blood group	-1.23
ENSMUSG00000021379	Id4	inhibitor of DNA binding 4	-1.23
ENSMUSG00000097006	9530082P21Rik	RIKEN cDNA 9530082P21 gene	-1.23
ENSMUSG00000102317	Gm37628	predicted gene, 37628	-1.23
ENSMUSG00000033577	Myo6	myosin VI	-1.24
ENSMUSG00000033885	Pxk	PX domain containing serine/threonine kinase	-1.24
ENSMUSG00000027634	Ndrg3	N-myc downstream regulated gene 3	-1.24
ENSMUSG00000039197	Adk	adenosine kinase	-1.24
ENSMUSG00000028862	Map3k6	mitogen-activated protein kinase kinase kinase 6	-1.24
ENSMUSG00000034731	Dgkh	diacylglycerol kinase, eta	-1.24
ENSMUSG00000030590	Fam98c	family with sequence similarity 98, member C	-1.24
ENSMUSG00000110289	4930412F12Rik	RIKEN cDNA 4930412F12 gene	-1.24
ENSMUSG00000020009	Ifngr1	interferon gamma receptor 1	-1.25
ENSMUSG00000033416	Gucd1	guanylyl cyclase domain containing 1	-1.25
ENSMUSG00000023019	Gpd1	glycerol-3-phosphate dehydrogenase 1 (soluble)	-1.25
ENSMUSG00000024063	Lbh	limb-bud and heart	-1.25
ENSMUSG00000021236	Entpd5	ectonucleoside triphosphate diphosphohydrolase 5	-1.25
ENSMUSG00000025190	Got1	glutamic-oxaloacetic transaminase 1, soluble	-1.25
ENSMUSG00000029413	Naaa	N-acylethanolamine acid amidase	-1.25
ENSMUSG00000058022	Adtrp	androgen dependent TFPI regulating protein	-1.25
ENSMUSG00000050777	Tmem37	transmembrane protein 37	-1.25
ENSMUSG00000049598	Vsig8	V-set and immunoglobulin domain containing 8	-1.25
ENSMUSG00000044042	Fmn1	formin 1	-1.26
ENSMUSG00000042808	Gpx2	glutathione peroxidase 2	-1.26
ENSMUSG00000084883	Ccdc85c	coiled-coil domain containing 85C	-1.26
ENSMUSG00000061845	Defa35	defensin, alpha, 35	-1.26
ENSMUSG00000029470	P2rx4	purinergic receptor P2X, ligand-gated ion channel 4	-1.26
ENSMUSG00000026259	Ngef	neuronal guanine nucleotide exchange factor	-1.26
ENSMUSG00000006567	Atp7b	ATPase, Cu++ transporting, beta polypeptide	-1.26
ENSMUSG00000093394	Gm20621	predicted gene 20621	-1.26
ENSMUSG00000022711	Pmm2	phosphomannomutase 2	-1.27
ENSMUSG00000038507	Parp12	poly (ADP-ribose) polymerase family, member 12	-1.27
ENSMUSG00000027457	Snph	syntaphilin	-1.27
ENSMUSG00000090115	Usp49	ubiquitin specific peptidase 49	-1.27
ENSMUSG00000042097	Zfp239	zinc finger protein 239	-1.27
ENSMUSG00000025584	Pde8a	phosphodiesterase 8A	-1.27
ENSMUSG00000010830	Kdelr3	KDEL (Lys-Asp-Glu-Leu) endoplasmic reticulum protein retention receptor 3	-1.27
ENSMUSG00000034509	Mad2l1bp	MAD2L1 binding protein	-1.27
ENSMUSG00000056492	Adgrf5	adhesion G protein-coupled receptor F5	-1.27
ENSMUSG00000099582	Gm29014	predicted gene 29014	-1.27
ENSMUSG00000032324	Tspan3	tetraspanin 3	-1.28
ENSMUSG00000005125	Ndrg1	N-myc downstream regulated gene 1	-1.28
ENSMUSG00000063931	Pepd	peptidase D	-1.28
ENSMUSG00000046519	Golph3l	golgi phosphoprotein 3-like	-1.28
ENSMUSG00000034066	Farp2	FERM, RhoGEF and pleckstrin domain protein 2	-1.28

ENSMUSG00000047409	Ctdspl	CTD (carboxy-terminal domain, RNA polymerase II, polypeptide A) small phosphatase-like	-1.28
ENSMUSG00000032842	Abcc10	ATP-binding cassette, sub-family C (CFTR/MRP), member 10	-1.28
ENSMUSG00000027314	Dll4	delta like canonical Notch ligand 4	-1.28
ENSMUSG00000072115	Ang	angiogenin, ribonuclease, RNase A family, 5	-1.28
ENSMUSG00000104576	F830115B05Rik	RIKEN cDNA F830115B05 gene	-1.28
ENSMUSG00000035852	Misp	mitotic spindle positioning	-1.29
ENSMUSG00000029859	Epha1	Eph receptor A1	-1.29
ENSMUSG00000039304	Tnfsf10	tumor necrosis factor (ligand) superfamily, member 10	-1.29
ENSMUSG00000060147	Serpinb6a	serine (or cysteine) peptidase inhibitor, clade B, member 6a	-1.30
ENSMUSG00000033684	Qsox1	quiescin Q6 sulfhydryl oxidase 1	-1.30
ENSMUSG00000035722	Abca7	ATP-binding cassette, sub-family A (ABC1), member 7	-1.30
ENSMUSG00000024955	Esrra	estrogen related receptor, alpha	-1.30
ENSMUSG00000038332	Sesn1	sestrin 1	-1.30
ENSMUSG00000029166	Mapre3	microtubule-associated protein, RP/EB family, member 3	-1.30
ENSMUSG00000054474	Thnsl2	threonine synthase-like 2 (bacterial)	-1.30
ENSMUSG00000050944	Efcab5	EF-hand calcium binding domain 5	-1.30
ENSMUSG00000063689	Hist2h2ab	histone cluster 2, H2ab	-1.30
ENSMUSG00000029452	Tmem116	transmembrane protein 116	-1.30
ENSMUSG00000102837	Gm37383	predicted gene, 37383	-1.30
ENSMUSG00000024597	Slc12a2	solute carrier family 12, member 2	-1.31
ENSMUSG00000020432	Tcn2	transcobalamin 2	-1.31
ENSMUSG00000042659	Arrdc4	arrestin domain containing 4	-1.31
ENSMUSG00000021665	Hexb	hexosaminidase B	-1.31
ENSMUSG00000031453	Rasa3	RAS p21 protein activator 3	-1.31
ENSMUSG00000031788	Kifc3	kinesin family member C3	-1.31
ENSMUSG00000032860	P2ry2	purinergic receptor P2Y, G-protein coupled 2	-1.31
ENSMUSG00000086674	Zfp286os	zinc finger protein 286, opposite strand	-1.31
ENSMUSG00000059412	Fxyd2	FXYD domain-containing ion transport regulator 2	-1.31
ENSMUSG00000027800	Tm4sf1	transmembrane 4 superfamily member 1	-1.31
ENSMUSG00000109852	Gm45360	predicted gene 45360	-1.31
ENSMUSG00000028399	Ptprd	protein tyrosine phosphatase, receptor type, D	-1.32
ENSMUSG00000036402	Gng12	guanine nucleotide binding protein (G protein), gamma 12	-1.32
ENSMUSG00000039131	Gipc2	GIPC PDZ domain containing family, member 2	-1.32
ENSMUSG00000029053	Prkcz	protein kinase C, zeta	-1.32
ENSMUSG00000034248	Slc25a37	solute carrier family 25, member 37	-1.32
ENSMUSG00000020573	Pik3cg	phosphatidylinositol-4,5-bisphosphate 3-kinase catalytic subunit gamma	-1.32
ENSMUSG00000038365	Fbxo25	F-box protein 25	-1.32
ENSMUSG00000034371	Tkfc	triokinase, FMN cyclase	-1.32
ENSMUSG00000022900	Ildr1	immunoglobulin-like domain containing receptor 1	-1.32
ENSMUSG00000058586	Serhl	serine hydrolase-like	-1.32
ENSMUSG00000023150	Ivns1abp	influenza virus NS1A binding protein	-1.33
ENSMUSG00000025504	Eps8l2	EPS8-like 2	-1.33
ENSMUSG00000020520	Galnt10	polypeptide N-acetylgalactosaminyltransferase 10	-1.33
ENSMUSG00000022330	Osr2	odd-skipped related 2	-1.33
ENSMUSG00000056602	Fry	FRY microtubule binding protein	-1.33
ENSMUSG00000055866	Per2	period circadian clock 2	-1.33
ENSMUSG00000032380	Dapk2	death-associated protein kinase 2	-1.33
ENSMUSG00000086549	Gm13648	predicted gene 13648	-1.33
ENSMUSG00000116594	AC133488.1	novel protein	-1.33
ENSMUSG00000045664	Cdc42ep2	CDC42 effector protein (Rho GTPase binding) 2	-1.33
ENSMUSG00000021223	Papln	papilin, proteoglycan-like sulfated glycoprotein	-1.33
ENSMUSG00000050270	Tmem220	transmembrane protein 220	-1.33
ENSMUSG00000026103	Gls	glutaminase	-1.34
ENSMUSG00000021411	Pxdc1	PX domain containing 1	-1.34
ENSMUSG00000115062	4930544F09Rik	RIKEN cDNA 4930544F09 gene	-1.34
ENSMUSG00000021803	Cdhr1	cadherin-related family member 1	-1.34
ENSMUSG00000114612	Gm47550	predicted gene, 47550	-1.34
ENSMUSG00000111176	Gm47233	predicted gene, 47233	-1.34
ENSMUSG00000028719	Cmpk1	cytidine monophosphate (UMP-CMP) kinase 1	-1.35
ENSMUSG00000060012	Kif13b	kinesin family member 13B	-1.35
ENSMUSG00000014418	Hps5	HPS5, biogenesis of lysosomal organelles complex 2 subunit 2	-1.35
ENSMUSG00000053702	Nebl	nebulette	-1.35
ENSMUSG00000035473	Galm	galactose mutarotase	-1.35
ENSMUSG00000029385	Ccng2	cyclin G2	-1.35
ENSMUSG00000045314	Sowahb	sosondowah ankyrin repeat domain family member B	-1.35
ENSMUSG00000031897	Psmb10	proteasome (prosome, macropain) subunit, beta type 10	-1.35
ENSMUSG00000063838	Cdc42ep5	CDC42 effector protein (Rho GTPase binding) 5	-1.35
ENSMUSG00000026480	Ncf2	neutrophil cytosolic factor 2	-1.35
ENSMUSG00000112944	Gm48885	predicted gene, 48885	-1.35
ENSMUSG00000067203	H2-K2	histocompatibility 2, K region locus 2	-1.35
ENSMUSG00000078954	Arhgap8	Rho GTPase activating protein 8	-1.35

ENSMUSG00000102953	Gm37019	predicted gene, 37019	-1.35
ENSMUSG00000106659	Gm42161	predicted gene, 42161	-1.35
ENSMUSG00000030739	Myh14	myosin, heavy polypeptide 14	-1.36
ENSMUSG00000020029	Nudt4	nudix (nucleoside diphosphate linked moiety X)-type motif 4	-1.36
ENSMUSG00000024981	Acsl5	acyl-CoA synthetase long-chain family member 5	-1.36
ENSMUSG00000061666	Gdpd1	glycerophosphodiester phosphodiesterase domain containing 1	-1.36
ENSMUSG00000020752	Recql5	RecQ protein-like 5	-1.36
ENSMUSG00000037579	Kcnh3	potassium voltage-gated channel, subfamily H (eag-related), member 3	-1.36
ENSMUSG00000032454	Rbp2	retinol binding protein 2, cellular	-1.36
ENSMUSG00000030921	Trim30a	tripartite motif-containing 30A	-1.36
ENSMUSG00000024187	Fam234a	family with sequence similarity 234, member A	-1.37
ENSMUSG00000022843	Clcn2	chloride channel, voltage-sensitive 2	-1.37
ENSMUSG00000036452	Arhgap26	Rho GTPase activating protein 26	-1.37
ENSMUSG00000043445	Pgp	phosphoglycolate phosphatase	-1.37
ENSMUSG00000026102	Inpp1	inositol polyphosphate-1-phosphatase	-1.37
ENSMUSG00000044681	Cnpy1	canopy FGF signaling regulator 1	-1.37
ENSMUSG00000070034	Sp110	Sp110 nuclear body protein	-1.37
ENSMUSG00000108526	Gm45828	predicted gene 45828	-1.37
ENSMUSG00000028217	Cdh17	cadherin 17	-1.38
ENSMUSG00000000915	Hip1r	huntingtin interacting protein 1 related	-1.38
ENSMUSG00000031482	Slc25a15	solute carrier family 25 (mitochondrial carrier ornithine transporter), member 15	-1.38
ENSMUSG00000031887	Tradd	TNFRSF1A-associated via death domain	-1.38
ENSMUSG00000075010	AW112010	expressed sequence AW112010	-1.38
ENSMUSG00000029275	Gfi1	growth factor independent 1	-1.38
ENSMUSG00000025466	Fuom	fucose mutarotase	-1.38
ENSMUSG00000029923	Rab19	RAB19, member RAS oncogene family	-1.38
ENSMUSG00000074882	Cyp2c68	cytochrome P450, family 2, subfamily c, polypeptide 68	-1.38
ENSMUSG00000033721	Vav3	vav 3 oncogene	-1.38
ENSMUSG00000104693	Gm42941	predicted gene 42941	-1.38
ENSMUSG00000038239	Hrc	histidine rich calcium binding protein	-1.38
ENSMUSG00000036813	Entpd8	ectonucleoside triphosphate diphosphohydrolase 8	-1.39
ENSMUSG00000041827	Oasl1	2'-5' oligoadenylate synthetase-like 1	-1.39
ENSMUSG00000056938	Acbd4	acyl-Coenzyme A binding domain containing 4	-1.39
ENSMUSG00000021553	Slc28a3	solute carrier family 28 (sodium-coupled nucleoside transporter), member 3	-1.39
ENSMUSG00000002289	Angptl4	angiopoietin-like 4	-1.39
ENSMUSG00000113516	Gm47368	predicted gene, 47368	-1.39
ENSMUSG00000109446	Gm9195	predicted gene 9195	-1.39
ENSMUSG00000040345	Arhgap9	Rho GTPase activating protein 9	-1.39
ENSMUSG00000106206	Gm43094	predicted gene 43094	-1.39
ENSMUSG00000104094	Gm37314	predicted gene, 37314	-1.39
ENSMUSG00000018166	Erbb3	erb-b2 receptor tyrosine kinase 3	-1.40
ENSMUSG00000031955	Bcar1	breast cancer anti-estrogen resistance 1	-1.40
ENSMUSG00000046861	Hectd3	HECT domain E3 ubiquitin protein ligase 3	-1.40
ENSMUSG00000039234	Sec24d	Sec24 related gene family, member D (S. cerevisiae)	-1.40
ENSMUSG00000031823	Zdhhc7	zinc finger, DHHC domain containing 7	-1.40
ENSMUSG00000030761	Myo7a	myosin VIIA	-1.40
ENSMUSG00000023915	Tnfrsf21	tumor necrosis factor receptor superfamily, member 21	-1.40
ENSMUSG00000018983	E2f2	E2F transcription factor 2	-1.40
ENSMUSG00000020182	Ddc	dopa decarboxylase	-1.40
ENSMUSG00000028795	Ccdc28b	coiled coil domain containing 28B	-1.40
ENSMUSG00000046456	Tmem150b	transmembrane protein 150B	-1.40
ENSMUSG00000022150	Dab2	disabled 2, mitogen-responsive phosphoprotein	-1.40
ENSMUSG00000030934	Oat	ornithine aminotransferase	-1.41
ENSMUSG00000026687	Aldh9a1	aldehyde dehydrogenase 9, subfamily A1	-1.41
ENSMUSG00000021266	Wars	tryptophanyl-tRNA synthetase	-1.41
ENSMUSG00000042228	Lyn	LYN proto-oncogene, Src family tyrosine kinase	-1.41
ENSMUSG00000070661	Rnf186	ring finger protein 186	-1.41
ENSMUSG00000024247	Pkdcc	protein kinase domain containing, cytoplasmic	-1.41
ENSMUSG00000006395	Hyi	hydroxypyruvate isomerase (putative)	-1.41
ENSMUSG00000020847	Rph3al	rabphilin 3A-like (without C2 domains)	-1.41
ENSMUSG00000023393	Slc17a9	solute carrier family 17, member 9	-1.41
ENSMUSG00000106969	Gm42882	predicted gene 42882	-1.41
ENSMUSG00000098678	Mroh6	maestro heat-like repeat family member 6	-1.41
ENSMUSG00000074440	Defa3	defensin, alpha, 3	-1.41
ENSMUSG00000022419	Deptor	DEP domain containing MTOR-interacting protein	-1.42
ENSMUSG00000007038	Neu1	neuraminidase 1	-1.42
ENSMUSG00000038331	Satb2	special AT-rich sequence binding protein 2	-1.42
ENSMUSG00000039813	Tbc1d2	TBC1 domain family, member 2	-1.42
ENSMUSG00000026976	Pax8	paired box 8	-1.42
ENSMUSG00000111468	5033425B01Rik	RIKEN cDNA 5033425B01 gene	-1.42
ENSMUSG00000086067	Gm16183	predicted gene 16183	-1.42

ENSMUSG00000022186	Oxct1	3-oxoacid CoA transferase 1	-1.43
ENSMUSG00000047153	Khnyn	KH and NYN domain containing	-1.43
ENSMUSG00000023262	Acy1	aminoacylase 1	-1.43
ENSMUSG00000035509	Fbxl21	F-box and leucine-rich repeat protein 21	-1.43
ENSMUSG00000029381	Shroom3	shroom family member 3	-1.44
ENSMUSG00000026879	Gsn	gelsolin	-1.44
ENSMUSG00000020111	Micu1	mitochondrial calcium uptake 1	-1.44
ENSMUSG00000032602	Slc25a20	solute carrier family 25 (mitochondrial carnitine/acylcarnitine translocase), member 20	-1.44
ENSMUSG00000018334	Ksr1	kinase suppressor of ras 1	-1.44
ENSMUSG00000034656	Cacna1a	calcium channel, voltage-dependent, P/Q type, alpha 1A subunit	-1.44
ENSMUSG00000013539	Tango2	transport and golgi organization 2	-1.44
ENSMUSG00000029401	Rilpl2	Rab interacting lysosomal protein-like 2	-1.44
ENSMUSG00000003585	Sec14l2	SEC14-like lipid binding 2	-1.44
ENSMUSG00000085088	4931413K12Rik	RIKEN cDNA 4931413K12 gene	-1.44
ENSMUSG00000019122	Ccl9	chemokine (C-C motif) ligand 9	-1.44
ENSMUSG00000071550	Cfap44	cilia and flagella associated protein 44	-1.44
ENSMUSG00000018776	Slc35g3	solute carrier family 35, member G3	-1.44
ENSMUSG00000111412	Gm47047	predicted gene, 47047	-1.44
ENSMUSG00000102540	Gm38134	predicted gene, 38134	-1.44
ENSMUSG00000009647	Mcu	mitochondrial calcium uniporter	-1.45
ENSMUSG00000041605	Inava	innate immunity activator	-1.45
ENSMUSG00000003746	Man1a	mannosidase 1, alpha	-1.45
ENSMUSG00000000538	Tom1l2	target of myb1-like 2 (chicken)	-1.45
ENSMUSG00000031732	Phlpp2	PH domain and leucine rich repeat protein phosphatase 2	-1.45
ENSMUSG00000026922	Agpat2	1-acylglycerol-3-phosphate O-acyltransferase 2 (lysophosphatidic acid acyltransferase, beta)	-1.45
ENSMUSG00000037921	Ddx60	DEAD (Asp-Glu-Ala-Asp) box polypeptide 60	-1.45
ENSMUSG00000047250	Ptgs1	prostaglandin-endoperoxide synthase 1	-1.45
ENSMUSG00000078612	Fyb2	FYN binding protein 2	-1.45
ENSMUSG00000091890	A830073O21Rik	RIKEN cDNA A830073O21 gene	-1.45
ENSMUSG00000092074	Dynlt1a	dynein light chain Tctex-type 1A	-1.45
ENSMUSG00000113119	Gm48883	predicted gene, 48883	-1.45
ENSMUSG00000037033	Clca3b	chloride channel accessory 3B	-1.46
ENSMUSG00000021556	Golm1	golgi membrane protein 1	-1.46
ENSMUSG00000033124	Atg9a	autophagy related 9A	-1.46
ENSMUSG00000068417	Pnp2	purine-nucleoside phosphorylase 2	-1.46
ENSMUSG00000038811	Gngt2	guanine nucleotide binding protein (G protein), gamma transducing activity polypeptide 2	-1.46
ENSMUSG00000116220	A430088P11Rik	RIKEN cDNA A430088P11 gene	-1.46
ENSMUSG00000107310	Gm7452	predicted pseudogene 7452	-1.46
ENSMUSG00000030681	Mvp	major vault protein	-1.47
ENSMUSG00000024556	Me2	malic enzyme 2, NAD(+)-dependent, mitochondrial	-1.47
ENSMUSG00000007036	Abhd16a	abhydrolase domain containing 16A	-1.47
ENSMUSG00000039637	Coro7	coronin 7	-1.47
ENSMUSG00000036106	Prr5	proline rich 5 (renal)	-1.47
ENSMUSG00000035878	Hykk	hydroxylysine kinase 1	-1.47
ENSMUSG00000079174	Gm3054	predicted gene 3054	-1.47
ENSMUSG00000090293	Gm17034	predicted gene 17034	-1.47
ENSMUSG00000113935	Gm35732	predicted gene, 35732	-1.47
ENSMUSG00000048992	Prss32	protease, serine 32	-1.48
ENSMUSG00000039774	Galnt12	polypeptide N-acetylgalactosaminyltransferase 12	-1.48
ENSMUSG00000020099	Unc5b	unc-5 netrin receptor B	-1.48
ENSMUSG00000038497	Tmco3	transmembrane and coiled-coil domains 3	-1.48
ENSMUSG00000026094	Stk17b	serine/threonine kinase 17b (apoptosis-inducing)	-1.48
ENSMUSG00000022560	Slc52a2	solute carrier protein 52, member 2	-1.48
ENSMUSG00000087535	Zmiz1os1	Zmiz1 opposite strand 1	-1.48
ENSMUSG00000084978	Gm11655	predicted gene 11655	-1.48
ENSMUSG00000013707	Tnfaip8l2	tumor necrosis factor, alpha-induced protein 8-like 2	-1.48
ENSMUSG00000043004	Gng2	guanine nucleotide binding protein (G protein), gamma 2	-1.48
ENSMUSG00000030616	Sytl2	synaptotagmin-like 2	-1.49
ENSMUSG00000028064	Sema4a	sema domain, immunoglobulin domain (Ig), transmembrane domain (TM) and short cytoplasmic dom	-1.49
ENSMUSG00000023909	Paqr4	progestin and adipoQ receptor family member IV	-1.49
ENSMUSG00000031021	Tmem9b	TMEM9 domain family, member B	-1.49
ENSMUSG00000036553	Sh3tc1	SH3 domain and tetratricopeptide repeats 1	-1.49
ENSMUSG00000087691	Gm15674	predicted gene 15674	-1.49
ENSMUSG00000113776	Gm19327	predicted gene, 19327	-1.49
ENSMUSG00000092212	Slc22a13b-ps	solute carrier family 22 (organic cation transporter), member 13b, pseudogene	-1.49
ENSMUSG00000081245	Gm6587	predicted gene 6587	-1.49
ENSMUSG00000038298	Pdzk1	PDZ domain containing 1	-1.49
ENSMUSG00000097862	Gm26859	predicted gene, 26859	-1.49
ENSMUSG00000085944	1700003D09Rik	RIKEN cDNA 1700003D09 gene	-1.49
ENSMUSG00000021591	Glrx	glutaredoxin	-1.50
ENSMUSG00000033917	Gde1	glycerophosphodiester phosphodiesterase 1	-1.50

ENSMUSG00000021913	Ogdhl	oxoglutarate dehydrogenase-like	-1.50
ENSMUSG00000085333	1700030A11Rik	RIKEN cDNA 1700030A11 gene	-1.50
ENSMUSG00000096145	Vkorc1	vitamin K epoxide reductase complex, subunit 1	-1.50
ENSMUSG00000083020	Gm15331	predicted gene 15331	-1.50
ENSMUSG00000026546	Cfap45	cilia and flagella associated protein 45	-1.50
ENSMUSG00000027695	Pld1	phospholipase D1	-1.51
ENSMUSG00000076435	Acsf2	acyl-CoA synthetase family member 2	-1.51
ENSMUSG00000032047	Acat1	acetyl-Coenzyme A acetyltransferase 1	-1.51
ENSMUSG00000027765	P2ry1	purinergic receptor P2Y, G-protein coupled 1	-1.51
ENSMUSG00000036136	Fam110c	family with sequence similarity 110, member C	-1.51
ENSMUSG00000001665	Gstt3	glutathione S-transferase, theta 3	-1.51
ENSMUSG00000104132	Gm36952	predicted gene, 36952	-1.51
ENSMUSG00000085152	Gm11496	predicted gene 11496	-1.51
ENSMUSG00000109715	Gm45606	predicted gene 45606	-1.51
ENSMUSG00000038209	Itln1	intelectin 1 (galactofuranose binding)	-1.52
ENSMUSG00000024074	Crim1	cysteine rich transmembrane BMP regulator 1 (chordin like)	-1.52
ENSMUSG00000031327	Chic1	cysteine-rich hydrophobic domain 1	-1.52
ENSMUSG00000032080	Apoa4	apolipoprotein A-IV	-1.52
ENSMUSG00000020334	Slc22a4	solute carrier family 22 (organic cation transporter), member 4	-1.52
ENSMUSG00000096954	Gdap10	ganglioside-induced differentiation-associated-protein 10	-1.52
ENSMUSG00000019762	Iyd	iodotyrosine deiodinase	-1.52
ENSMUSG00000024386	Proc	protein C	-1.52
ENSMUSG00000042942	Greb1l	growth regulation by estrogen in breast cancer-like	-1.52
ENSMUSG00000066178	6030445D17Rik	RIKEN cDNA 6030445D17 gene	-1.52
ENSMUSG00000020154	Ptprb	protein tyrosine phosphatase, receptor type, B	-1.52
ENSMUSG00000073608	Gal3st2c	galactose-3-O-sulfotransferase 2C	-1.52
ENSMUSG00000020226	Slc5a4b	solute carrier family 5 (neutral amino acid transporters, system A), member 4b	-1.52
ENSMUSG00000111028	Gm5922	predicted gene 5922	-1.52
ENSMUSG00000022048	Dpysl2	dihydropyrimidinase-like 2	-1.53
ENSMUSG00000045934	Mtmr11	myotubularin related protein 11	-1.53
ENSMUSG00000029847	Slc23a4	solute carrier family 23 member 4	-1.53
ENSMUSG00000039754	Alkbh4	alkB homolog 4, lysine demethylase	-1.53
ENSMUSG00000078664	Sprr2a1	small proline-rich protein 2A1	-1.53
ENSMUSG00000046005	D830044D21Rik	RIKEN cDNA D830044D21 gene	-1.53
ENSMUSG00000041237	Pklr	pyruvate kinase liver and red blood cell	-1.54
ENSMUSG00000022105	Rb1	RB transcriptional corepressor 1	-1.54
ENSMUSG00000041992	Rapgef5	Rap guanine nucleotide exchange factor (GEF) 5	-1.54
ENSMUSG00000026304	Rab17	RAB17, member RAS oncogene family	-1.54
ENSMUSG00000070563	Spaca4	sperm acrosome associated 4	-1.54
ENSMUSG00000029352	Crybb3	crystallin, beta B3	-1.54
ENSMUSG00000104776	Gm43691	predicted gene 43691	-1.54
ENSMUSG00000078307	AI593442	expressed sequence AI593442	-1.54
ENSMUSG00000074217	2210011C24Rik	RIKEN cDNA 2210011C24 gene	-1.54
ENSMUSG00000103845	Gm19026	predicted gene, 19026	-1.54
ENSMUSG00000091475	2810468N07Rik	RIKEN cDNA 2810468N07 gene	-1.54
ENSMUSG00000038541	Srd5a2	steroid 5 alpha-reductase 2	-1.54
ENSMUSG00000065581	Mir194-1	microRNA 194-1	-1.54
ENSMUSG00000029646	Cdx2	caudal type homeobox 2	-1.55
ENSMUSG00000028519	Dab1	disabled 1	-1.55
ENSMUSG00000027524	Edn3	endothelin 3	-1.55
ENSMUSG00000042333	Tnfrsf14	tumor necrosis factor receptor superfamily, member 14 (herpesvirus entry mediator)	-1.55
ENSMUSG00000071604	Fam189a2	family with sequence similarity 189, member A2	-1.55
ENSMUSG00000040147	Maob	monoamine oxidase B	-1.55
ENSMUSG00000104950	4833413G10Rik	RIKEN cDNA 4833413G10 gene	-1.55
ENSMUSG00000052767	Gm12703	predicted gene 12703	-1.55
ENSMUSG00000064036	Mro	maestro	-1.55
ENSMUSG00000040528	Milr1	mast cell immunoglobulin like receptor 1	-1.55
ENSMUSG00000067049	Unc93a	unc-93 homolog A	-1.55
ENSMUSG00000057914	Cacnb2	calcium channel, voltage-dependent, beta 2 subunit	-1.55
ENSMUSG00000024610	Cd74	CD74 antigen (invariant polypeptide of major histocompatibility complex, class II antigen-associated)	-1.56
ENSMUSG00000024511	Rab27b	RAB27B, member RAS oncogene family	-1.56
ENSMUSG00000024887	Asah2	N-acylsphingosine amidohydrolase 2	-1.56
ENSMUSG00000021779	Thrb	thyroid hormone receptor beta	-1.56
ENSMUSG00000109314	Gm44699	predicted gene 44699	-1.56
ENSMUSG00000026069	Il1rl1	interleukin 1 receptor-like 1	-1.56
ENSMUSG00000053819	Camk2d	calcium/calmodulin-dependent protein kinase II, delta	-1.57
ENSMUSG00000027901	Dennd2d	DENN/MADD domain containing 2D	-1.57
ENSMUSG00000052396	Mogat2	monoacylglycerol O-acyltransferase 2	-1.57
ENSMUSG00000025006	Sorbs1	sorbin and SH3 domain containing 1	-1.57
ENSMUSG00000034591	Slc41a2	solute carrier family 41, member 2	-1.57
ENSMUSG00000031337	Mtm1	X-linked myotubular myopathy gene 1	-1.57

ENSMUSG00000022799	Arhgap31	Rho GTPase activating protein 31	-1.57
ENSMUSG00000063652	Slc22a21	solute carrier family 22 (organic cation transporter), member 21	-1.57
ENSMUSG00000026969	Fam166a	family with sequence similarity 166, member A	-1.57
ENSMUSG00000051339	2900026A02Rik	RIKEN cDNA 2900026A02 gene	-1.58
ENSMUSG00000005373	Mlxipl	MLX interacting protein-like	-1.58
ENSMUSG00000024867	Pip5k1b	phosphatidylinositol-4-phosphate 5-kinase, type 1 beta	-1.58
ENSMUSG00000005686	Ampd3	adenosine monophosphate deaminase 3	-1.58
ENSMUSG00000022574	Naprt	nicotinate phosphoribosyltransferase	-1.58
ENSMUSG00000093695	Gm20717	predicted gene 20717	-1.58
ENSMUSG00000112129	Pbld1	phenazine biosynthesis-like protein domain containing 1	-1.58
ENSMUSG00000003546	Klc4	kinesin light chain 4	-1.59
ENSMUSG00000005615	Pcyt1a	phosphate cytidylyltransferase 1, choline, alpha isoform	-1.59
ENSMUSG00000025648	Pfkfb4	6-phosphofructo-2-kinase/fructose-2,6-biphosphatase 4	-1.59
ENSMUSG00000087230	Mroh3	maestro heat-like repeat family member 3	-1.59
ENSMUSG00000112513	4930422I22Rik	RIKEN cDNA 4930422I22 gene	-1.59
ENSMUSG00000107760	Gm44401	predicted gene, 44401	-1.59
ENSMUSG00000040134	Rdh7	retinol dehydrogenase 7	-1.59
ENSMUSG00000102283	Gm37000	predicted gene, 37000	-1.59
ENSMUSG00000063730	Hsd3b2	hydroxy-delta-5-steroid dehydrogenase, 3 beta- and steroid delta-isomerase 2	-1.59
ENSMUSG00000084325	Gm15550	predicted gene 15550	-1.59
ENSMUSG00000056973	Ces1d	carboxylesterase 1D	-1.59
ENSMUSG00000101814	Gm17807	predicted gene, 17807	-1.59
ENSMUSG00000032661	Oas3	2'-5' oligoadenylate synthetase 3	-1.60
ENSMUSG00000062410	Hsd3b3	hydroxy-delta-5-steroid dehydrogenase, 3 beta- and steroid delta-isomerase 3	-1.60
ENSMUSG00000004151	Etv1	ets variant 1	-1.60
ENSMUSG00000022575	Gsdmd	gasdermin D	-1.61
ENSMUSG00000026670	Uap1	UDP-N-acetylglucosamine pyrophosphorylase 1	-1.61
ENSMUSG00000028121	Bcar3	breast cancer anti-estrogen resistance 3	-1.61
ENSMUSG00000003271	Sult2b1	sulfotransferase family, cytosolic, 2B, member 1	-1.61
ENSMUSG00000074063	Osgin1	oxidative stress induced growth inhibitor 1	-1.61
ENSMUSG00000044080	S100a1	S100 calcium binding protein A1	-1.61
ENSMUSG00000041372	B4galnt3	beta-1,4-N-acetyl-galactosaminyl transferase 3	-1.61
ENSMUSG00000104571	Gm43010	predicted gene 43010	-1.61
ENSMUSG00000104114	Gm37297	predicted gene, 37297	-1.61
ENSMUSG00000036960	Clca2	chloride channel accessory 2	-1.61
ENSMUSG00000034528	Hsd17b13	hydroxysteroid (17-beta) dehydrogenase 13	-1.62
ENSMUSG00000092474	Gm20478	predicted gene 20478	-1.62
ENSMUSG00000090582	Gm17024	predicted gene 17024	-1.62
ENSMUSG00000032536	Trak1	trafficking protein, kinesin binding 1	-1.63
ENSMUSG00000026896	Ifih1	interferon induced with helicase C domain 1	-1.63
ENSMUSG00000052102	Gnpda1	glucosamine-6-phosphate deaminase 1	-1.63
ENSMUSG00000037649	H2-DMa	histocompatibility 2, class II, locus DMa	-1.63
ENSMUSG00000112346	Gm48768	predicted gene, 48768	-1.63
ENSMUSG00000048473	Sult6b2	sulfotransferase family 6B, member 2	-1.63
ENSMUSG00000024379	Tslp	thymic stromal lymphopoietin	-1.63
ENSMUSG00000040712	Camta2	calmodulin binding transcription activator 2	-1.64
ENSMUSG00000028088	Fmo5	flavin containing monooxygenase 5	-1.64
ENSMUSG00000025198	Erlin1	ER lipid raft associated 1	-1.64
ENSMUSG00000064254	Ethe1	ethylmalonic encephalopathy 1	-1.64
ENSMUSG00000033538	Casp4	caspase 4, apoptosis-related cysteine peptidase	-1.64
ENSMUSG00000015085	Entpd2	ectonucleoside triphosphate diphosphohydrolase 2	-1.64
ENSMUSG00000006711	D130043K22Rik	RIKEN cDNA D130043K22 gene	-1.64
ENSMUSG00000082677	Gm15371	predicted gene 15371	-1.64
ENSMUSG00000038188	Scarf1	scavenger receptor class F, member 1	-1.64
ENSMUSG00000071265	1700086L19Rik	RIKEN cDNA 1700086L19 gene	-1.64
ENSMUSG00000103391	Gm38302	predicted gene, 38302	-1.64
ENSMUSG00000043501	Lgals2	lectin, galactose-binding, soluble 2	-1.65
ENSMUSG00000029455	Aldh2	aldehyde dehydrogenase 2, mitochondrial	-1.65
ENSMUSG00000017057	Il13ra1	interleukin 13 receptor, alpha 1	-1.65
ENSMUSG00000020889	Nr1d1	nuclear receptor subfamily 1, group D, member 1	-1.65
ENSMUSG00000055675	Kbtbd11	kelch repeat and BTB (POZ) domain containing 11	-1.65
ENSMUSG00000051065	Mb21d2	Mab-21 domain containing 2	-1.65
ENSMUSG00000025759	Mfsd8	major facilitator superfamily domain containing 8	-1.65
ENSMUSG00000036473	Tbc1d24	TBC1 domain family, member 24	-1.65
ENSMUSG00000111379	Gm48346	predicted gene, 48346	-1.65
ENSMUSG00000102626	Gm18553	predicted gene, 18553	-1.65
ENSMUSG00000106583	4930447N08Rik	RIKEN cDNA 4930447N08 gene	-1.65
ENSMUSG00000034127	Tspan8	tetraspanin 8	-1.66
ENSMUSG00000108291	Gm44292	predicted gene, 44292	-1.66
ENSMUSG00000030790	Adm	adrenomedullin	-1.66
ENSMUSG00000106541	Gm43246	predicted gene 43246	-1.66

ENSMUSG00000028017	Egf	epidermal growth factor	-1.66
ENSMUSG00000027669	Gnb4	guanine nucleotide binding protein (G protein), beta 4	-1.66
ENSMUSG00000049037	Clec4a1	C-type lectin domain family 4, member a1	-1.66
ENSMUSG00000106466	Gm40348	predicted gene, 40348	-1.66
ENSMUSG00000108211	Gm44130	predicted gene, 44130	-1.66
ENSMUSG00000102752	Gm7694	predicted gene 7694	-1.67
ENSMUSG00000053310	Nrgn	neurogranin	-1.67
ENSMUSG00000001348	Acp5	acid phosphatase 5, tartrate resistant	-1.67
ENSMUSG00000037818	Abhd18	abhydrolase domain containing 18	-1.67
ENSMUSG00000090174	Gm10612	predicted gene 10612	-1.67
ENSMUSG00000084329	Gm6733	predicted gene 6733	-1.67
ENSMUSG00000106178	Gm42987	predicted gene 42987	-1.67
ENSMUSG00000049176	Frmpd4	FERM and PDZ domain containing 4	-1.67
ENSMUSG00000021097	Clmn	calmin	-1.68
ENSMUSG00000032802	Srxn1	sulfiredoxin 1 homolog (S. cerevisiae)	-1.68
ENSMUSG00000006651	Aplp1	amyloid beta (A4) precursor-like protein 1	-1.68
ENSMUSG00000045312	Lhfpl2	lipoma HMGIC fusion partner-like 2	-1.68
ENSMUSG00000072214	Sept5	septin 5	-1.68
ENSMUSG00000022853	Ehhadh	enoyl-Coenzyme A, hydratase/3-hydroxyacyl Coenzyme A dehydrogenase	-1.68
ENSMUSG00000105456	Gm43745	predicted gene 43745	-1.68
ENSMUSG00000071551	Akr1c19	aldo-keto reductase family 1, member C19	-1.68
ENSMUSG00000005360	Slc1a3	solute carrier family 1 (glial high affinity glutamate transporter), member 3	-1.68
ENSMUSG00000038473	Nos1ap	nitric oxide synthase 1 (neuronal) adaptor protein	-1.69
ENSMUSG00000000958	Slc7a7	solute carrier family 7 (cationic amino acid transporter, y+ system), member 7	-1.69
ENSMUSG00000106475	Gm43011	predicted gene 43011	-1.69
ENSMUSG00000020600	Slc7a15	solute carrier family 7 (cationic amino acid transporter, y+ system), member 15	-1.69
ENSMUSG00000027834	Serpini1	serine (or cysteine) peptidase inhibitor, clade I, member 1	-1.69
ENSMUSG00000041323	Ak7	adenylate kinase 7	-1.69
ENSMUSG00000085095	Gm15635	predicted gene 15635	-1.69
ENSMUSG00000068323	Slc4a5	solute carrier family 4, sodium bicarbonate cotransporter, member 5	-1.69
ENSMUSG00000021250	Fos	FBJ osteosarcoma oncogene	-1.70
ENSMUSG00000044626	Liph	lipase, member H	-1.70
ENSMUSG00000024222	Fkbp5	FK506 binding protein 5	-1.70
ENSMUSG00000101517	4732465J04Rik	RIKEN cDNA 4732465J04 gene	-1.70
ENSMUSG00000105460	Gm42671	predicted gene 42671	-1.70
ENSMUSG00000049493	Pls1	plastin 1 (I-isoform)	-1.71
ENSMUSG00000032120	C2cd2l	C2 calcium-dependent domain containing 2-like	-1.71
ENSMUSG00000024712	Rfk	riboflavin kinase	-1.71
ENSMUSG00000025876	Unc5a	unc-5 netrin receptor A	-1.71
ENSMUSG00000100183	Gm28512	predicted gene 28512	-1.71
ENSMUSG00000058740	Kcnt1	potassium channel, subfamily T, member 1	-1.71
ENSMUSG00000081236	Gm13574	predicted gene 13574	-1.71
ENSMUSG00000047728	BC025446	cDNA sequence BC025446	-1.72
ENSMUSG00000019699	Akt3	thymoma viral proto-oncogene 3	-1.72
ENSMUSG00000026204	Ptprn	protein tyrosine phosphatase, receptor type, N	-1.72
ENSMUSG00000030228	Pik3c2g	phosphatidylinositol-4-phosphate 3-kinase catalytic subunit type 2 gamma	-1.72
ENSMUSG00000006281	Tep1	telomerase associated protein 1	-1.73
ENSMUSG00000069515	Lyz1	lysozyme 1	-1.73
ENSMUSG00000020733	Slc9a3r1	solute carrier family 9 (sodium/hydrogen exchanger), member 3 regulator 1	-1.73
ENSMUSG00000054662	Ano9	anoctamin 9	-1.73
ENSMUSG00000035642	Aamdc	adipogenesis associated Mth938 domain containing	-1.73
ENSMUSG00000039208	Metrnl	meteorin, glial cell differentiation regulator-like	-1.73
ENSMUSG00000043333	Rhbdl2	rhomboid like 2	-1.73
ENSMUSG00000037455	Slc18b1	solute carrier family 18, subfamily B, member 1	-1.73
ENSMUSG00000001985	Grik3	glutamate receptor, ionotropic, kainate 3	-1.73
ENSMUSG00000024960	Plcb3	phospholipase C, beta 3	-1.74
ENSMUSG00000034781	Gna11	guanine nucleotide binding protein, alpha 11	-1.74
ENSMUSG00000041737	Tmem45b	transmembrane protein 45b	-1.74
ENSMUSG00000041515	Irf8	interferon regulatory factor 8	-1.74
ENSMUSG00000020407	Upp1	uridine phosphorylase 1	-1.74
ENSMUSG00000043432	Leng9	leukocyte receptor cluster (LRC) member 9	-1.74
ENSMUSG00000037661	Gpr160	G protein-coupled receptor 160	-1.74
ENSMUSG00000052392	Acot4	acyl-CoA thioesterase 4	-1.74
ENSMUSG00000056671	Prelid2	PRELI domain containing 2	-1.74
ENSMUSG00000105872	Gm9515	predicted gene 9515	-1.74
ENSMUSG00000026062	Slc9a2	solute carrier family 9 (sodium/hydrogen exchanger), member 2	-1.75
ENSMUSG00000028518	Prkaa2	protein kinase, AMP-activated, alpha 2 catalytic subunit	-1.75
ENSMUSG00000027412	Lpin3	lipin 3	-1.75
ENSMUSG00000074604	Mgst2	microsomal glutathione S-transferase 2	-1.75
ENSMUSG00000048440	Cyp4f16	cytochrome P450, family 4, subfamily f, polypeptide 16	-1.75
ENSMUSG00000004815	Dgkq	diacylglycerol kinase, theta	-1.75

ENSMUSG00000028179	Cth	cystathionase (cystathionine gamma-lyase)	-1.75
ENSMUSG00000002108	Nr1h3	nuclear receptor subfamily 1, group H, member 3	-1.75
ENSMUSG00000031255	Sytl4	synaptotagmin-like 4	-1.75
ENSMUSG00000024471	Myot	myotilin	-1.75
ENSMUSG00000037605	Adgrl3	adhesion G protein-coupled receptor L3	-1.75
ENSMUSG00000102425	Gm26616	predicted gene, 26616	-1.75
ENSMUSG00000029772	Ahcyl2	S-adenosylhomocysteine hydrolase-like 2	-1.76
ENSMUSG00000061751	Kalrn	kalirin, RhoGEF kinase	-1.76
ENSMUSG00000026688	Mgst3	microsomal glutathione S-transferase 3	-1.76
ENSMUSG00000046688	Tifa	TRAF-interacting protein with forkhead-associated domain	-1.76
ENSMUSG00000042428	Mgat3	mannoside acetylglucosaminyltransferase 3	-1.76
ENSMUSG00000027510	Rbm38	RNA binding motif protein 38	-1.76
ENSMUSG00000029919	Hpgds	hematopoietic prostaglandin D synthase	-1.76
ENSMUSG00000030278	Cidec	cell death-inducing DFFA-like effector c	-1.76
ENSMUSG00000067813	Xkr9	X-linked Kx blood group related 9	-1.76
ENSMUSG00000038917	3930402G23Rik	RIKEN cDNA 3930402G23 gene	-1.76
ENSMUSG00000086479	Gm16014	predicted gene 16014	-1.76
ENSMUSG00000020017	Hal	histidine ammonia lyase	-1.76
ENSMUSG00000027870	Hao2	hydroxyacid oxidase 2	-1.76
ENSMUSG00000038807	Rap1gap2	RAP1 GTPase activating protein 2	-1.77
ENSMUSG00000018909	Arrb1	arrestin, beta 1	-1.77
ENSMUSG00000059316	Slc27a4	solute carrier family 27 (fatty acid transporter), member 4	-1.77
ENSMUSG00000054469	Lclat1	lysocardiolipin acyltransferase 1	-1.77
ENSMUSG00000021830	Txndc16	thioredoxin domain containing 16	-1.77
ENSMUSG00000022504	Ciita	class II transactivator	-1.77
ENSMUSG00000067219	Nipal1	NIPA-like domain containing 1	-1.77
ENSMUSG00000034957	Cebpa	CCAAT/enhancer binding protein (C/EBP), alpha	-1.77
ENSMUSG00000005089	Slc1a2	solute carrier family 1 (glial high affinity glutamate transporter), member 2	-1.77
ENSMUSG00000081219	Bambi-ps1	BMP and activin membrane-bound inhibitor, pseudogene (Xenopus laevis)	-1.77
ENSMUSG00000021566	Slc6a19os	solute carrier family 6 (neurotransmitter transporter), member 19, opposite strand	-1.77
ENSMUSG00000055670	Zzef1	zinc finger, ZZ-type with EF hand domain 1	-1.78
ENSMUSG00000032246	Calml4	calmodulin-like 4	-1.78
ENSMUSG00000025888	Casp1	caspase 1	-1.78
ENSMUSG00000047496	Rnf152	ring finger protein 152	-1.78
ENSMUSG00000063142	Kcnma1	potassium large conductance calcium-activated channel, subfamily M, alpha member 1	-1.78
ENSMUSG00000025726	Slc28a1	solute carrier family 28 (sodium-coupled nucleoside transporter), member 1	-1.78
ENSMUSG00000031444	F10	coagulation factor X	-1.78
ENSMUSG00000104291	A130071D04Rik	RIKEN cDNA A130071D04 gene	-1.78
ENSMUSG00000109244	Gm44751	predicted gene 44751	-1.78
ENSMUSG00000037031	Tspan15	tetraspanin 15	-1.79
ENSMUSG00000106078	Gm43697	predicted gene 43697	-1.79
ENSMUSG00000060208	Defa17	defensin, alpha, 17	-1.79
ENSMUSG00000034115	Scn11a	sodium channel, voltage-gated, type XI, alpha	-1.79
ENSMUSG00000040600	Eps8l3	EPS8-like 3	-1.80
ENSMUSG00000067297	Ifit1bl2	interferon induced protein with tetratricopeptide repeats 1B like 2	-1.80
ENSMUSG00000064262	Gimap8	GTPase, IMAP family member 8	-1.80
ENSMUSG00000106933	Gm43621	predicted gene 43621	-1.80
ENSMUSG00000031364	Grpr	gastrin releasing peptide receptor	-1.80
ENSMUSG00000061171	Slc38a11	solute carrier family 38, member 11	-1.80
ENSMUSG00000107624	Gm44005	predicted gene, 44005	-1.80
ENSMUSG00000005640	Insrr	insulin receptor-related receptor	-1.80
ENSMUSG00000025076	Casp7	caspase 7	-1.81
ENSMUSG00000021062	Rab15	RAB15, member RAS oncogene family	-1.81
ENSMUSG00000017868	Sgk2	serum/glucocorticoid regulated kinase 2	-1.81
ENSMUSG00000089900	Tbc1d22bos	TBC1 domain family, member 22B, opposite strand	-1.81
ENSMUSG00000106186	Gm43627	predicted gene 43627	-1.81
ENSMUSG00000009772	Nuak2	NUAK family, SNF1-like kinase, 2	-1.82
ENSMUSG00000034714	Ttyh2	tweety family member 2	-1.82
ENSMUSG00000021280	Exoc3l4	exocyst complex component 3-like 4	-1.82
ENSMUSG00000052131	Akr1b7	aldo-keto reductase family 1, member B7	-1.82
ENSMUSG00000021221	Dpf3	D4, zinc and double PHD fingers, family 3	-1.82
ENSMUSG00000074628	Tldc2	TBC/LysM associated domain containing 2	-1.82
ENSMUSG00000099025	Gm27162	predicted gene 27162	-1.82
ENSMUSG00000035561	Aldh1b1	aldehyde dehydrogenase 1 family, member B1	-1.83
ENSMUSG00000024644	Cndp2	CNDP dipeptidase 2 (metallopeptidase M20 family)	-1.83
ENSMUSG00000061411	Nol4l	nucleolar protein 4-like	-1.83
ENSMUSG00000025993	Slc40a1	solute carrier family 40 (iron-regulated transporter), member 1	-1.83
ENSMUSG00000026880	Stom	stomatin	-1.83
ENSMUSG00000052085	Dock8	dedicator of cytokinesis 8	-1.83
ENSMUSG00000048572	Tmem252	transmembrane protein 252	-1.83
ENSMUSG00000051452	Gm11437	predicted gene 11437	-1.83

ENSMUSG00000011034	Slc5a1	solute carrier family 5 (sodium/glucose cotransporter), member 1	-1.83
ENSMUSG00000044017	Adgrd1	adhesion G protein-coupled receptor D1	-1.83
ENSMUSG00000101624	Gm28882	predicted gene 28882	-1.83
ENSMUSG00000050762	Prss27	protease, serine 27	-1.83
ENSMUSG00000108850	Gm44914	predicted gene 44914	-1.83
ENSMUSG00000021596	Mctp1	multiple C2 domains, transmembrane 1	-1.83
ENSMUSG00000072244	Trim6	tripartite motif-containing 6	-1.83
ENSMUSG00000001156	Mxd1	MAX dimerization protein 1	-1.84
ENSMUSG00000028943	Espn	espin	-1.84
ENSMUSG00000057069	Ero1lb	ERO1-like beta (S. cerevisiae)	-1.84
ENSMUSG00000045871	Slitrk6	SLIT and NTRK-like family, member 6	-1.84
ENSMUSG00000055963	Triqk	triple QxxK/R motif containing	-1.84
ENSMUSG00000028427	Aqp7	aquaporin 7	-1.84
ENSMUSG00000073405	H2-T-ps	histocompatibility 2, T region locus, pseudogene	-1.84
ENSMUSG00000038372	Gmds	GDP-mannose 4, 6-dehydratase	-1.85
ENSMUSG00000032899	Styk1	serine/threonine/tyrosine kinase 1	-1.85
ENSMUSG00000030281	Il17rc	interleukin 17 receptor C	-1.85
ENSMUSG00000060002	Chpt1	choline phosphotransferase 1	-1.85
ENSMUSG00000027358	Bmp2	bone morphogenetic protein 2	-1.85
ENSMUSG00000027219	Slc28a2	solute carrier family 28 (sodium-coupled nucleoside transporter), member 2	-1.85
ENSMUSG00000028262	Clca3a2	chloride channel accessory 3A2	-1.85
ENSMUSG00000097099	Gm9917	predicted gene 9917	-1.85
ENSMUSG00000028544	Slc5a9	solute carrier family 5 (sodium/glucose cotransporter), member 9	-1.85
ENSMUSG00000110266	Gm32742	predicted gene, 32742	-1.85
ENSMUSG00000030621	Me3	malic enzyme 3, NADP(+)-dependent, mitochondrial	-1.85
ENSMUSG00000041617	Ccdc74a	coiled-coil domain containing 74A	-1.85
ENSMUSG00000021337	Scgn	secretagogin, EF-hand calcium binding protein	-1.85
ENSMUSG00000017950	Hnf4a	hepatic nuclear factor 4, alpha	-1.86
ENSMUSG00000048905	4930539E08Rik	RIKEN cDNA 4930539E08 gene	-1.86
ENSMUSG00000063522	2010109I03Rik	RIKEN cDNA 2010109I03 gene	-1.86
ENSMUSG00000090214	Gm15657	predicted gene 15657	-1.86
ENSMUSG00000034765	Dusp5	dual specificity phosphatase 5	-1.86
ENSMUSG00000074892	B3galt5	UDP-Gal:betaGlcNAc beta 1,3-galactosyltransferase, polypeptide 5	-1.86
ENSMUSG00000054150	Syne3	spectrin repeat containing, nuclear envelope family member 3	-1.86
ENSMUSG00000110520	Gm45776	predicted gene 45776	-1.86
ENSMUSG00000110735	Gm47173	predicted gene, 47173	-1.86
ENSMUSG00000105986	Gm43065	predicted gene 43065	-1.86
ENSMUSG00000030838	Ush1c	USH1 protein network component harmonin	-1.87
ENSMUSG00000031442	Mcf2l	mcf.2 transforming sequence-like	-1.87
ENSMUSG00000024066	Xdh	xanthine dehydrogenase	-1.87
ENSMUSG00000030208	Emp1	epithelial membrane protein 1	-1.87
ENSMUSG00000028536	2610528J11Rik	RIKEN cDNA 2610528J11 gene	-1.87
ENSMUSG00000020709	Adap2	ArfGAP with dual PH domains 2	-1.87
ENSMUSG00000104316	Gm37909	predicted gene, 37909	-1.87
ENSMUSG00000031129	Slc9a9	solute carrier family 9 (sodium/hydrogen exchanger), member 9	-1.87
ENSMUSG00000035186	Ubd	ubiquitin D	-1.87
ENSMUSG00000114705	Gm18760	predicted gene, 18760	-1.87
ENSMUSG00000031608	Galnt7	polypeptide N-acetylgalactosaminyltransferase 7	-1.88
ENSMUSG00000026175	Vil1	villin 1	-1.88
ENSMUSG00000064213	Defa24	defensin, alpha, 24	-1.88
ENSMUSG00000039910	Cited2	Cbp/p300-interacting transactivator, with Glu/Asp-rich carboxy-terminal domain, 2	-1.88
ENSMUSG00000029727	Cyp3a13	cytochrome P450, family 3, subfamily a, polypeptide 13	-1.88
ENSMUSG00000020272	Stk10	serine/threonine kinase 10	-1.88
ENSMUSG00000032776	Mctp2	multiple C2 domains, transmembrane 2	-1.88
ENSMUSG00000022040	Ephx2	epoxide hydrolase 2, cytoplasmic	-1.88
ENSMUSG00000024225	Clps	colipase, pancreatic	-1.88
ENSMUSG00000089960	Ugt1a1	UDP glucuronosyltransferase 1 family, polypeptide A1	-1.88
ENSMUSG00000003032	Klf4	Kruppel-like factor 4 (gut)	-1.89
ENSMUSG00000029561	Oasl2	2'-5' oligoadenylate synthetase-like 2	-1.89
ENSMUSG00000048373	Fgfbp1	fibroblast growth factor binding protein 1	-1.89
ENSMUSG00000022018	Rgcc	regulator of cell cycle	-1.89
ENSMUSG00000029490	Mfsd7a	major facilitator superfamily domain containing 7A	-1.89
ENSMUSG00000029059	Fam213b	family with sequence similarity 213, member B	-1.90
ENSMUSG00000037003	Tns2	tensin 2	-1.90
ENSMUSG00000052135	Foxo6	forkhead box O6	-1.90
ENSMUSG00000047878	A4galt	alpha 1,4-galactosyltransferase	-1.90
ENSMUSG00000029802	Abcg2	ATP binding cassette subfamily G member 2 (Junior blood group)	-1.90
ENSMUSG00000037451	Slc22a20	solute carrier family 22 (organic anion transporter), member 20	-1.90
ENSMUSG00000045281	Gpr20	G protein-coupled receptor 20	-1.90
ENSMUSG00000029992	Gfpt1	glutamine fructose-6-phosphate transaminase 1	-1.91
ENSMUSG00000054161	Fam83e	family with sequence similarity 83, member E	-1.91

ENSMUSG00000024180	Tmem8	transmembrane protein 8 (five membrane-spanning domains)	-1.91
ENSMUSG00000022836	Mylk	myosin, light polypeptide kinase	-1.91
ENSMUSG00000043079	Synpo	synaptopodin	-1.91
ENSMUSG00000028194	Ddah1	dimethylarginine dimethylaminohydrolase 1	-1.91
ENSMUSG00000067206	Lrrc66	leucine rich repeat containing 66	-1.91
ENSMUSG00000057596	Trim30d	tripartite motif-containing 30D	-1.91
ENSMUSG00000022340	Sybu	syntabulin (syntaxin-interacting)	-1.91
ENSMUSG00000022445	Cyp2d26	cytochrome P450, family 2, subfamily d, polypeptide 26	-1.91
ENSMUSG00000028699	Tspan1	tetraspanin 1	-1.92
ENSMUSG00000021336	Slc17a4	solute carrier family 17 (sodium phosphate), member 4	-1.92
ENSMUSG00000041120	Nbl1	neuroblastoma, suppression of tumorigenicity 1	-1.92
ENSMUSG00000030214	Plbd1	phospholipase B domain containing 1	-1.92
ENSMUSG00000028364	Tnc	tenascin C	-1.92
ENSMUSG00000054453	Sytl5	synaptotagmin-like 5	-1.92
ENSMUSG00000022947	Cbr3	carbonyl reductase 3	-1.92
ENSMUSG00000015401	Cltrn	collectrin, amino acid transport regulator	-1.92
ENSMUSG00000034320	Slc26a2	solute carrier family 26 (sulfate transporter), member 2	-1.93
ENSMUSG00000031838	Ifi30	interferon gamma inducible protein 30	-1.93
ENSMUSG00000025429	Pstpip2	proline-serine-threonine phosphatase-interacting protein 2	-1.93
ENSMUSG00000035258	Abi3bp	ABI gene family, member 3 (NESH) binding protein	-1.93
ENSMUSG00000032690	Oas2	2'-5' oligoadenylate synthetase 2	-1.93
ENSMUSG00000040537	Adam22	a disintegrin and metallopeptidase domain 22	-1.93
ENSMUSG00000069456	Rdh16	retinol dehydrogenase 16	-1.93
ENSMUSG00000061762	Tac1	tachykinin 1	-1.93
ENSMUSG00000005045	Chd5	chromodomain helicase DNA binding protein 5	-1.93
ENSMUSG00000019359	Gdpd2	glycerophosphodiester phosphodiesterase domain containing 2	-1.93
ENSMUSG00000004864	Mapk13	mitogen-activated protein kinase 13	-1.94
ENSMUSG00000057963	Itpk1	inositol 1,3,4-triphosphate 5/6 kinase	-1.94
ENSMUSG00000024642	Tle4	transducin-like enhancer of split 4	-1.94
ENSMUSG00000005107	Slc2a9	solute carrier family 2 (facilitated glucose transporter), member 9	-1.94
ENSMUSG00000023827	Agpat4	1-acylglycerol-3-phosphate O-acyltransferase 4 (lysophosphatidic acid acyltransferase, delta)	-1.94
ENSMUSG00000040298	Btbd16	BTB (POZ) domain containing 16	-1.94
ENSMUSG00000084948	1700061H18Rik	RIKEN cDNA 1700061H18 gene	-1.94
ENSMUSG00000029322	Plac8	placenta-specific 8	-1.95
ENSMUSG00000025498	Irf7	interferon regulatory factor 7	-1.95
ENSMUSG00000020681	Ace	angiotensin I converting enzyme (peptidyl-dipeptidase A) 1	-1.95
ENSMUSG00000038534	Osbpl7	oxysterol binding protein-like 7	-1.95
ENSMUSG00000103527	Gm37261	predicted gene, 37261	-1.95
ENSMUSG00000019989	Enpp3	ectonucleotide pyrophosphatase/phosphodiesterase 3	-1.95
ENSMUSG00000086844	B230206H07Rik	RIKEN cDNA B230206H07 gene	-1.95
ENSMUSG00000106108	Gm43221	predicted gene 43221	-1.95
ENSMUSG00000115388	Eppk1	epiplakin 1	-1.96
ENSMUSG00000038178	Slc43a2	solute carrier family 43, member 2	-1.96
ENSMUSG00000053279	Aldh1a1	aldehyde dehydrogenase family 1, subfamily A1	-1.96
ENSMUSG00000000204	Slfn4	schlafen 4	-1.96
ENSMUSG00000052271	Bhlha15	basic helix-loop-helix family, member a15	-1.96
ENSMUSG00000023206	Il15ra	interleukin 15 receptor, alpha chain	-1.96
ENSMUSG00000090608	Gm17200	predicted gene 17200	-1.96
ENSMUSG00000110597	Gm8798	predicted gene 8798	-1.96
ENSMUSG00000017978	Cadps2	Ca2+-dependent activator protein for secretion 2	-1.97
ENSMUSG00000018217	Pmp22	peripheral myelin protein 22	-1.97
ENSMUSG00000057897	Camk2b	calcium/calmodulin-dependent protein kinase II, beta	-1.97
ENSMUSG00000051379	Flrt3	fibronectin leucine rich transmembrane protein 3	-1.97
ENSMUSG00000025002	Cyp2c55	cytochrome P450, family 2, subfamily c, polypeptide 55	-1.97
ENSMUSG00000010122	Slc47a1	solute carrier family 47, member 1	-1.97
ENSMUSG00000037157	Il22ra1	interleukin 22 receptor, alpha 1	-1.98
ENSMUSG00000034687	Fras1	Fraser extracellular matrix complex subunit 1	-1.98
ENSMUSG00000041261	Car8	carbonic anhydrase 8	-1.98
ENSMUSG00000085747	Slc13a2os	solute carrier family 13 (sodium-dependent dicarboxylate transporter), member 2, opposite strand	-1.98
ENSMUSG00000017718	Afmid	arylformamidase	-1.98
ENSMUSG00000056032	BC018473	cDNA sequence BC018473	-1.98
ENSMUSG00000032377	Plscr4	phospholipid scramblase 4	-1.98
ENSMUSG00000116010	Gm36026	predicted gene, 36026	-1.98
ENSMUSG00000103553	Gm38218	predicted gene, 38218	-1.98
ENSMUSG00000030909	Anks4b	ankyrin repeat and sterile alpha motif domain containing 4B	-1.99
ENSMUSG00000074445	Sprr2a3	small proline-rich protein 2A3	-1.99
ENSMUSG00000046410	Kcnk6	potassium inwardly-rectifying channel, subfamily K, member 6	-1.99
ENSMUSG00000038963	Slco4a1	solute carrier organic anion transporter family, member 4a1	-1.99
ENSMUSG00000039981	Zc3h12d	zinc finger CCCH type containing 12D	-1.99
ENSMUSG00000104966	Gm43273	predicted gene 43273	-1.99
ENSMUSG00000038037	Socs1	suppressor of cytokine signaling 1	-1.99

ENSMUSG00000090145	Ugt1a6b	UDP glucuronosyltransferase 1 family, polypeptide A6B	-1.99
ENSMUSG00000078964	Ces1b	carboxylesterase 1B	-1.99
ENSMUSG00000018126	Baiap2l2	BAI1-associated protein 2-like 2	-2.00
ENSMUSG00000060733	Ipmk	inositol polyphosphate multikinase	-2.00
ENSMUSG00000025357	Dgka	diacylglycerol kinase, alpha	-2.00
ENSMUSG00000045038	Prkce	protein kinase C, epsilon	-2.00
ENSMUSG00000022555	Dgat1	diacylglycerol O-acyltransferase 1	-2.00
ENSMUSG00000028635	Edn2	endothelin 2	-2.00
ENSMUSG00000085941	Gm11201	predicted gene 11201	-2.00
ENSMUSG00000006731	B4galnt1	beta-1,4-N-acetyl-galactosaminyl transferase 1	-2.01
ENSMUSG00000024937	Ehbp1l1	EH domain binding protein 1-like 1	-2.01
ENSMUSG00000023243	Kcnk5	potassium channel, subfamily K, member 5	-2.01
ENSMUSG00000079547	H2-DMb1	histocompatibility 2, class II, locus Mb1	-2.01
ENSMUSG00000090066	1110002E22Rik	RIKEN cDNA 1110002E22 gene	-2.01
ENSMUSG00000055027	Smyd1	SET and MYND domain containing 1	-2.01
ENSMUSG00000021624	Cd180	CD180 antigen	-2.01
ENSMUSG00000068587	Mgam	maltase-glucoamylase	-2.02
ENSMUSG00000007034	Slc44a4	solute carrier family 44, member 4	-2.02
ENSMUSG00000019970	Sgk1	serum/glucocorticoid regulated kinase 1	-2.02
ENSMUSG00000074622	Mafb	v-maf musculoaponeurotic fibrosarcoma oncogene family, protein B (avian)	-2.02
ENSMUSG00000043629	1700019D03Rik	RIKEN cDNA 1700019D03 gene	-2.02
ENSMUSG00000103854	Gm37250	predicted gene, 37250	-2.02
ENSMUSG00000079242	C730034F03Rik	RIKEN cDNA C730034F03 gene	-2.02
ENSMUSG00000021456	Fbp2	fructose bisphosphatase 2	-2.03
ENSMUSG00000034570	Inpp5j	inositol polyphosphate 5-phosphatase J	-2.03
ENSMUSG00000024743	Syt7	synaptotagmin VII	-2.03
ENSMUSG00000078945	Naip2	NLR family, apoptosis inhibitory protein 2	-2.03
ENSMUSG00000042041	2010003K11Rik	RIKEN cDNA 2010003K11 gene	-2.03
ENSMUSG00000024697	Gna14	guanine nucleotide binding protein, alpha 14	-2.03
ENSMUSG00000023057	Fabp2	fatty acid binding protein 2, intestinal	-2.03
ENSMUSG00000082148	Gm12266	predicted gene 12266	-2.03
ENSMUSG00000075517	Cyp2d37-ps	cytochrome P450, family 2, subfamily d, polypeptide 37, pseudogene	-2.03
ENSMUSG00000028337	Coro2a	coronin, actin binding protein 2A	-2.04
ENSMUSG00000034116	Vav1	vav 1 oncogene	-2.04
ENSMUSG00000027452	Acss1	acyl-CoA synthetase short-chain family member 1	-2.04
ENSMUSG00000020614	Fam20a	family with sequence similarity 20, member A	-2.04
ENSMUSG00000020774	Aspa	aspartoacylase	-2.04
ENSMUSG00000020105	Lrig3	leucine-rich repeats and immunoglobulin-like domains 3	-2.04
ENSMUSG00000038020	Rapgefl1	Rap guanine nucleotide exchange factor (GEF)-like 1	-2.04
ENSMUSG00000049799	Lrrc19	leucine rich repeat containing 19	-2.04
ENSMUSG00000072571	Tmem253	transmembrane protein 253	-2.04
ENSMUSG00000030683	Sez6l2	seizure related 6 homolog like 2	-2.04
ENSMUSG00000085196	Gm14963	predicted gene 14963	-2.04
ENSMUSG00000029811	Aoc1	amine oxidase, copper-containing 1	-2.05
ENSMUSG00000032033	Barx2	BarH-like homeobox 2	-2.05
ENSMUSG00000035041	Creb3l3	cAMP responsive element binding protein 3-like 3	-2.05
ENSMUSG00000038349	Plcl1	phospholipase C-like 1	-2.05
ENSMUSG00000101581	C430002N11Rik	RIKEN cDNA C430002N11 gene	-2.05
ENSMUSG00000029821	Gsdme	gasdermin E	-2.06
ENSMUSG00000027227	Sord	sorbitol dehydrogenase	-2.06
ENSMUSG00000020826	Nos2	nitric oxide synthase 2, inducible	-2.06
ENSMUSG00000020151	Ptprr	protein tyrosine phosphatase, receptor type, R	-2.06
ENSMUSG00000025467	Prap1	proline-rich acidic protein 1	-2.06
ENSMUSG00000035967	Ints6l	integrator complex subunit 6 like	-2.06
ENSMUSG00000015970	Chdh	choline dehydrogenase	-2.08
ENSMUSG00000066800	Rnasel	ribonuclease L (2', 5'-oligoisoadenylate synthetase-dependent)	-2.08
ENSMUSG00000026726	Cubn	cubilin (intrinsic factor-cobalamin receptor)	-2.08
ENSMUSG00000022938	Fam3b	family with sequence similarity 3, member B	-2.08
ENSMUSG00000079339	Ifit1bl1	interferon induced protein with tetratricpeptide repeats 1B like 1	-2.08
ENSMUSG00000026489	Coq8a	coenzyme Q8A	-2.08
ENSMUSG00000050106	Tmc8	transmembrane channel-like gene family 8	-2.08
ENSMUSG00000026692	Fmo4	flavin containing monooxygenase 4	-2.08
ENSMUSG00000092517	Art2a-ps	ADP-ribosyltransferase 2a, pseudogene	-2.08
ENSMUSG00000043673	Kcns3	potassium voltage-gated channel, delayed-rectifier, subfamily S, member 3	-2.08
ENSMUSG00000082485	Gm15693	predicted gene 15693	-2.08
ENSMUSG00000041268	Dmxl2	Dmx-like 2	-2.09
ENSMUSG00000036594	H2-Aa	histocompatibility 2, class II antigen A, alpha	-2.09
ENSMUSG00000032373	Car12	carbonic anhydrase 12	-2.09
ENSMUSG00000020447	Npc1l1	NPC1 like intracellular cholesterol transporter 1	-2.09
ENSMUSG00000032083	Apoa1	apolipoprotein A-I	-2.09
ENSMUSG00000075014	Gm10800	predicted gene 10800	-2.09

ENSMUSG00000040584	Abcb1a	ATP-binding cassette, sub-family B (MDR/TAP), member 1A	-2.10
ENSMUSG00000024619	Cdx1	caudal type homeobox 1	-2.10
ENSMUSG00000013523	Bcas1	breast carcinoma amplified sequence 1	-2.10
ENSMUSG00000034413	Neurl1b	neuralized E3 ubiquitin protein ligase 1B	-2.10
ENSMUSG00000026077	Npas2	neuronal PAS domain protein 2	-2.10
ENSMUSG00000041741	Pde3a	phosphodiesterase 3A, cGMP inhibited	-2.10
ENSMUSG00000040767	Snrnp25	small nuclear ribonucleoprotein 25 (U11/U12)	-2.10
ENSMUSG00000027508	Pag1	phosphoprotein associated with glycosphingolipid microdomains 1	-2.10
ENSMUSG00000042312	S100a13	S100 calcium binding protein A13	-2.10
ENSMUSG00000052415	Tchh	trichohyalin	-2.10
ENSMUSG00000039699	Batf2	basic leucine zipper transcription factor, ATF-like 2	-2.10
ENSMUSG00000000805	Car4	carbonic anhydrase 4	-2.10
ENSMUSG00000004113	Cacna1b	calcium channel, voltage-dependent, N type, alpha 1B subunit	-2.10
ENSMUSG00000020405	Fabp6	fatty acid binding protein 6, ileal (gastrotropin)	-2.10
ENSMUSG00000079588	Tmem182	transmembrane protein 182	-2.10
ENSMUSG00000030650	Tmc5	transmembrane channel-like gene family 5	-2.11
ENSMUSG00000021196	Pfkp	phosphofructokinase, platelet	-2.11
ENSMUSG00000017639	Rab11fip4	RAB11 family interacting protein 4 (class II)	-2.11
ENSMUSG00000022747	St3gal6	ST3 beta-galactoside alpha-2,3-sialyltransferase 6	-2.11
ENSMUSG00000039578	Ccser1	coiled-coil serine rich 1	-2.11
ENSMUSG00000104350	Gm38244	predicted gene, 38244	-2.11
ENSMUSG00000029082	Bst1	bone marrow stromal cell antigen 1	-2.11
ENSMUSG00000025037	Maoa	monoamine oxidase A	-2.12
ENSMUSG00000052595	A1cf	APOBEC1 complementation factor	-2.12
ENSMUSG00000068547	Clca4a	chloride channel accessory 4A	-2.12
ENSMUSG00000096001	2610528A11Rik	RIKEN cDNA 2610528A11 gene	-2.12
ENSMUSG00000028158	Mttp	microsomal triglyceride transfer protein	-2.12
ENSMUSG00000027230	Creb3l1	cAMP responsive element binding protein 3-like 1	-2.13
ENSMUSG00000041642	Kif21b	kinesin family member 21B	-2.13
ENSMUSG00000050747	Trim15	tripartite motif-containing 15	-2.13
ENSMUSG00000003420	Fcgrt	Fc receptor, IgG, alpha chain transporter	-2.13
ENSMUSG00000023045	Soat2	sterol O-acyltransferase 2	-2.13
ENSMUSG00000032105	Pdzd3	PDZ domain containing 3	-2.13
ENSMUSG00000009646	Pla2g12b	phospholipase A2, group XIIB	-2.13
ENSMUSG00000000125	Wnt3	wingless-type MMTV integration site family, member 3	-2.13
ENSMUSG00000097254	C430042M11Rik	RIKEN cDNA C430042M11 gene	-2.13
ENSMUSG00000041644	Slc5a12	solute carrier family 5 (sodium/glucose cotransporter), member 12	-2.13
ENSMUSG00000026417	Pigr	polymeric immunoglobulin receptor	-2.14
ENSMUSG00000024747	Aldh1a7	aldehyde dehydrogenase family 1, subfamily A7	-2.14
ENSMUSG00000019935	Slc17a8	solute carrier family 17 (sodium-dependent inorganic phosphate cotransporter), member 8	-2.14
ENSMUSG00000030587	2200002D01Rik	RIKEN cDNA 2200002D01 gene	-2.15
ENSMUSG00000022824	Muc13	mucin 13, epithelial transmembrane	-2.15
ENSMUSG00000024254	Abcg8	ATP binding cassette subfamily G member 8	-2.15
ENSMUSG00000056413	Adap1	ArfGAP with dual PH domains 1	-2.15
ENSMUSG00000108688	Gm44985	predicted gene 44985	-2.15
ENSMUSG00000015405	Ace2	angiotensin I converting enzyme (peptidyl-dipeptidase A) 2	-2.15
ENSMUSG00000060586	H2-Eb1	histocompatibility 2, class II antigen E beta	-2.16
ENSMUSG00000073802	Cdkn2b	cyclin dependent kinase inhibitor 2B	-2.16
ENSMUSG00000034919	Ttc22	tetratricopeptide repeat domain 22	-2.16
ENSMUSG00000039405	Prss23	protease, serine 23	-2.16
ENSMUSG00000031089	Slc6a14	solute carrier family 6 (neurotransmitter transporter), member 14	-2.16
ENSMUSG00000079445	B3gnt7	UDP-GlcNAc:betaGal beta-1,3-N-acetylglucosaminyltransferase 7	-2.16
ENSMUSG00000075611	Gm11545	predicted gene 11545	-2.16
ENSMUSG00000097779	4833407H14Rik	RIKEN cDNA 4833407H14 gene	-2.16
ENSMUSG00000030786	Itgam	integrin alpha M	-2.16
ENSMUSG00000096914	Galntl6	UDP-N-acetyl-alpha-D-galactosamine:polypeptide N-acetylgalactosaminyltransferase-like 6	-2.16
ENSMUSG00000004266	Ptpn6	protein tyrosine phosphatase, non-receptor type 6	-2.17
ENSMUSG00000037408	Cnnm4	cyclin M4	-2.17
ENSMUSG00000073421	H2-Ab1	histocompatibility 2, class II antigen A, beta 1	-2.17
ENSMUSG00000043592	Unc5cl	unc-5 family C-terminal like	-2.17
ENSMUSG00000037686	Aspg	asparaginase	-2.17
ENSMUSG00000100927	Gm28536	predicted gene 28536	-2.17
ENSMUSG00000106191	4930557B06Rik	RIKEN cDNA 4930557B06 gene	-2.17
ENSMUSG00000072875	Gpr27	G protein-coupled receptor 27	-2.17
ENSMUSG00000050965	Prkca	protein kinase C, alpha	-2.18
ENSMUSG00000030866	Ern2	endoplasmic reticulum (ER) to nucleus signalling 2	-2.18
ENSMUSG00000023073	Slc10a2	solute carrier family 10, member 2	-2.18
ENSMUSG00000055435	Maf	avian musculoaponeurotic fibrosarcoma oncogene homolog	-2.18
ENSMUSG00000012123	Crybg2	crystallin beta-gamma domain containing 2	-2.18
ENSMUSG00000031209	Heph	hephaestin	-2.18
ENSMUSG00000027463	Slc52a3	solute carrier protein family 52, member 3	-2.18

ENSMUSG00000028278	Rragd	Ras-related GTP binding D	-2.18
ENSMUSG00000040249	Lrp1	low density lipoprotein receptor-related protein 1	-2.19
ENSMUSG00000045316	Fahd1	fumarylacetoacetate hydrolase domain containing 1	-2.19
ENSMUSG00000025557	Slc15a1	solute carrier family 15 (oligopeptide transporter), member 1	-2.19
ENSMUSG00000091956	C2cd4b	C2 calcium-dependent domain containing 4B	-2.19
ENSMUSG00000029409	U90926	cDNA sequence U90926	-2.19
ENSMUSG00000107425	Gm44216	predicted gene, 44216	-2.19
ENSMUSG00000066687	Zbtb16	zinc finger and BTB domain containing 16	-2.19
ENSMUSG00000102802	Mgam2-ps	maltase-glucoamylase 2, pseudogene	-2.20
ENSMUSG00000030793	Pycard	PYD and CARD domain containing	-2.20
ENSMUSG00000024277	Mapre2	microtubule-associated protein, RP/EB family, member 2	-2.20
ENSMUSG00000024727	Trpm6	transient receptor potential cation channel, subfamily M, member 6	-2.20
ENSMUSG00000033871	Ppargc1b	peroxisome proliferative activated receptor, gamma, coactivator 1 beta	-2.20
ENSMUSG00000061825	Ces2c	carboxylesterase 2C	-2.20
ENSMUSG00000026222	Sp100	nuclear antigen Sp100	-2.20
ENSMUSG00000093867	Gsdmcl2	gasdermin C-like 2	-2.20
ENSMUSG00000047115	Fam221a	family with sequence similarity 221, member A	-2.20
ENSMUSG00000022219	Cideb	cell death-inducing DNA fragmentation factor, alpha subunit-like effector B	-2.20
ENSMUSG00000047414	Flrt2	fibronectin leucine rich transmembrane protein 2	-2.20
ENSMUSG00000109295	Gm5739	predicted gene 5739	-2.20
ENSMUSG00000044734	Serpinb1a	serine (or cysteine) peptidase inhibitor, clade B, member 1a	-2.21
ENSMUSG00000035000	Dpp4	dipeptidylpeptidase 4	-2.21
ENSMUSG00000021416	Eci3	enoyl-Coenzyme A delta isomerase 3	-2.21
ENSMUSG00000019558	Slc6a8	solute carrier family 6 (neurotransmitter transporter, creatine), member 8	-2.22
ENSMUSG00000031803	B3gnt3	UDP-GlcNAc:betaGal beta-1,3-N-acetylglucosaminyltransferase 3	-2.22
ENSMUSG00000031618	Nr3c2	nuclear receptor subfamily 3, group C, member 2	-2.22
ENSMUSG00000038305	Spats2l	spermatogenesis associated, serine-rich 2-like	-2.22
ENSMUSG00000044165	Bcl2l15	BCLl2-like 15	-2.22
ENSMUSG00000110573	Gm5485	predicted gene 5485	-2.22
ENSMUSG00000039062	Anpep	alanyl (membrane) aminopeptidase	-2.22
ENSMUSG00000074345	Tnfaip8l3	tumor necrosis factor, alpha-induced protein 8-like 3	-2.22
ENSMUSG00000060703	Cd302	CD302 antigen	-2.22
ENSMUSG00000110498	A630001O12Rik	RIKEN cDNA A630001O12 gene	-2.22
ENSMUSG00000050158	Olfr165	olfactory receptor 165	-2.22
ENSMUSG00000072941	Sod3	superoxide dismutase 3, extracellular	-2.22
ENSMUSG00000061958	Gm14851	predicted gene 14851	-2.23
ENSMUSG00000020609	Apob	apolipoprotein B	-2.23
ENSMUSG00000029377	Ereg	epiregulin	-2.23
ENSMUSG00000004655	Aqp1	aquaporin 1	-2.24
ENSMUSG00000022809	Nr1i2	nuclear receptor subfamily 1, group I, member 2	-2.24
ENSMUSG00000110060	Gm9860	predicted gene 9860	-2.24
ENSMUSG00000029188	Slc34a2	solute carrier family 34 (sodium phosphate), member 2	-2.24
ENSMUSG00000097316	Gm10516	predicted gene 10516	-2.24
ENSMUSG00000027474	Ccm2l	cerebral cavernous malformation 2-like	-2.24
ENSMUSG00000025738	Fbxl16	F-box and leucine-rich repeat protein 16	-2.24
ENSMUSG00000037280	Galnt6	polypeptide N-acetylgalactosaminyltransferase 6	-2.25
ENSMUSG00000017688	Hnf4g	hepatocyte nuclear factor 4, gamma	-2.25
ENSMUSG00000085683	Tmem238l	transmembrane protein 238 like	-2.25
ENSMUSG00000034449	Dhrs11	dehydrogenase/reductase (SDR family) member 11	-2.25
ENSMUSG00000049690	Nckap5	NCK-associated protein 5	-2.25
ENSMUSG00000024411	Aqp4	aquaporin 4	-2.25
ENSMUSG00000028976	Slc2a5	solute carrier family 2 (facilitated glucose transporter), member 5	-2.25
ENSMUSG00000042388	Dlgap3	DLG associated protein 3	-2.25
ENSMUSG00000071335	Mfsd4b3	major facilitator superfamily domain containing 4B3	-2.25
ENSMUSG00000032087	Dscaml1	DS cell adhesion molecule like 1	-2.25
ENSMUSG00000053695	Defb37	defensin beta 37	-2.25
ENSMUSG00000036912	Piwil4	piwi-like RNA-mediated gene silencing 4	-2.25
ENSMUSG00000038642	Ctss	cathepsin S	-2.26
ENSMUSG00000040505	Abcg5	ATP binding cassette subfamily G member 5	-2.26
ENSMUSG00000112592	Gm19972	predicted gene, 19972	-2.26
ENSMUSG00000061414	Cracr2a	calcium release activated channel regulator 2A	-2.27
ENSMUSG00000026399	Cd55	CD55 molecule, decay accelerating factor for complement	-2.27
ENSMUSG00000038903	Ccdc68	coiled-coil domain containing 68	-2.27
ENSMUSG00000014773	Dll1	delta like canonical Notch ligand 1	-2.27
ENSMUSG00000075702	Selenom	selenoprotein M	-2.27
ENSMUSG00000027068	Dhrs9	dehydrogenase/reductase (SDR family) member 9	-2.27
ENSMUSG00000048022	Tmem229a	transmembrane protein 229A	-2.27
ENSMUSG00000071506	Tmem139	transmembrane protein 139	-2.27
ENSMUSG00000102759	Gm10463	predicted gene 10463	-2.27
ENSMUSG00000025207	Sema4g	sema domain, immunoglobulin domain (Ig), transmembrane domain (TM) and short cytoplasmic dom	-2.28
ENSMUSG00000031402	Mpp1	membrane protein, palmitoylated	-2.28

ENSMUSG00000019872	Smpdl3a	sphingomyelin phosphodiesterase, acid-like 3A	-2.28
ENSMUSG00000006362	Cbfa2t3	core-binding factor, runt domain, alpha subunit 2, translocated to, 3 (human)	-2.28
ENSMUSG00000031844	Hsd17b2	hydroxysteroid (17-beta) dehydrogenase 2	-2.28
ENSMUSG00000057880	Abat	4-aminobutyrate aminotransferase	-2.28
ENSMUSG00000028150	Rorc	RAR-related orphan receptor gamma	-2.28
ENSMUSG00000040471	Ggt6	gamma-glutamyltransferase 6	-2.28
ENSMUSG00000031937	Vstm5	V-set and transmembrane domain containing 5	-2.28
ENSMUSG00000033453	Adamts15	a disintegrin-like and metallopeptidase (reprolysin type) with thrombospondin type 1 motif, 15	-2.28
ENSMUSG00000042096	Dao	D-amino acid oxidase	-2.28
ENSMUSG00000058624	Gda	guanine deaminase	-2.29
ENSMUSG00000024713	Pcsk5	proprotein convertase subtilisin/kexin type 5	-2.29
ENSMUSG00000025528	2010106E10Rik	RIKEN cDNA 2010106E10 gene	-2.29
ENSMUSG00000104535	Gm42686	predicted gene 42686	-2.29
ENSMUSG00000031389	Arhgap4	Rho GTPase activating protein 4	-2.29
ENSMUSG00000055833	1700034H15Rik	RIKEN cDNA 1700034H15 gene	-2.29
ENSMUSG00000032034	Kcnj5	potassium inwardly-rectifying channel, subfamily J, member 5	-2.29
ENSMUSG00000006313	Upk1a	uroplakin 1A	-2.29
ENSMUSG00000035493	Tgfbi	transforming growth factor, beta induced	-2.30
ENSMUSG00000030157	Clec2d	C-type lectin domain family 2, member d	-2.30
ENSMUSG00000019478	Rab4a	RAB4A, member RAS oncogene family	-2.30
ENSMUSG00000025790	Slco3a1	solute carrier organic anion transporter family, member 3a1	-2.30
ENSMUSG00000033213	AA467197	expressed sequence AA467197	-2.30
ENSMUSG00000019278	Dpep1	dipeptidase 1 (renal)	-2.30
ENSMUSG00000050982	Apol10a	apolipoprotein L 10A	-2.30
ENSMUSG00000090125	Pou3f1	POU domain, class 3, transcription factor 1	-2.30
ENSMUSG00000081303	Gm16011	predicted gene 16011	-2.31
ENSMUSG00000049307	Fut4	fucosyltransferase 4	-2.31
ENSMUSG00000104737	Gm42937	predicted gene 42937	-2.31
ENSMUSG00000109936	Gm45889	predicted gene 45889	-2.31
ENSMUSG00000094559	Cyp2d34	cytochrome P450, family 2, subfamily d, polypeptide 34	-2.31
ENSMUSG00000075184	F930017D23Rik	RIKEN cDNA F930017D23 gene	-2.31
ENSMUSG00000117084	AC154912.1	novel transcript, antisense to Fkbp5	-2.31
ENSMUSG00000026786	Apbb1ip	amyloid beta (A4) precursor protein-binding, family B, member 1 interacting protein	-2.31
ENSMUSG00000045005	Fzd5	frizzled class receptor 5	-2.32
ENSMUSG00000028307	Aldob	aldolase B, fructose-bisphosphate	-2.32
ENSMUSG00000025059	Gk	glycerol kinase	-2.32
ENSMUSG00000030155	Clec2e	C-type lectin domain family 2, member e	-2.32
ENSMUSG00000064373	Selenop	selenoprotein P	-2.32
ENSMUSG00000086825	Gm15675	predicted gene 15675	-2.32
ENSMUSG00000032098	Treh	trehalase (brush-border membrane glycoprotein)	-2.32
ENSMUSG00000054999	Naaladl1	N-acetylated alpha-linked acidic dipeptidase-like 1	-2.32
ENSMUSG00000079559	Colca2	COLCA2 homolog	-2.32
ENSMUSG00000082154	Gm16464	predicted gene 16464	-2.32
ENSMUSG00000038528	Mfsd4b5	major facilitator superfamily domain containing 4B5	-2.32
ENSMUSG00000053168	9030619P08Rik	RIKEN cDNA 9030619P08 gene	-2.32
ENSMUSG00000017943	Gdap1l1	ganglioside-induced differentiation-associated protein 1-like 1	-2.32
ENSMUSG00000030303	Far2	fatty acyl CoA reductase 2	-2.33
ENSMUSG00000024731	Ms4a10	membrane-spanning 4-domains, subfamily A, member 10	-2.33
ENSMUSG00000074195	Clca4b	chloride channel accessory 4B	-2.33
ENSMUSG00000073403	Gm10499	predicted gene 10499	-2.33
ENSMUSG00000113800	2210039B01Rik	RIKEN cDNA 2210039B01 gene	-2.33
ENSMUSG00000114150	Gm46367	predicted gene, 46367	-2.33
ENSMUSG00000050866	Clrn3	clarin 3	-2.34
ENSMUSG00000023914	Mep1a	meprin 1 alpha	-2.34
ENSMUSG00000078670	Fam174b	family with sequence similarity 174, member B	-2.34
ENSMUSG00000073399	Trim40	tripartite motif-containing 40	-2.34
ENSMUSG00000001670	Tat	tyrosine aminotransferase	-2.34
ENSMUSG00000027555	Car13	carbonic anhydrase 13	-2.34
ENSMUSG00000028525	Pde4b	phosphodiesterase 4B, cAMP specific	-2.34
ENSMUSG00000058260	Serpina9	serine (or cysteine) peptidase inhibitor, clade A (alpha-1 antiproteinase, antitrypsin), member 9	-2.34
ENSMUSG00000050505	Pcdh20	protocadherin 20	-2.34
ENSMUSG00000041301	Cftr	cystic fibrosis transmembrane conductance regulator	-2.35
ENSMUSG00000000308	Ckmt1	creatine kinase, mitochondrial 1, ubiquitous	-2.35
ENSMUSG00000040412	5330417C22Rik	RIKEN cDNA 5330417C22 gene	-2.35
ENSMUSG00000023259	Slc26a6	solute carrier family 26, member 6	-2.35
ENSMUSG00000094651	Gal3st2	galactose-3-O-sulfotransferase 2	-2.35
ENSMUSG00000030834	Abcc6	ATP-binding cassette, sub-family C (CFTR/MRP), member 6	-2.35
ENSMUSG00000024885	Aldh3b1	aldehyde dehydrogenase 3 family, member B1	-2.35
ENSMUSG00000044827	Tlr1	toll-like receptor 1	-2.35
ENSMUSG00000027297	Ltk	leukocyte tyrosine kinase	-2.35
ENSMUSG00000078439	Smim24	small integral membrane protein 24	-2.36

ENSMUSG00000029314	Gpat3	glycerol-3-phosphate acyltransferase 3	-2.36
ENSMUSG00000074300	BC030870	cDNA sequence BC030870	-2.36
ENSMUSG00000036242	Armh4	armadillo-like helical domain containing 4	-2.36
ENSMUSG00000074183	Gsta1	glutathione S-transferase, alpha 1 (Ya)	-2.36
ENSMUSG00000053964	Lgals4	lectin, galactose binding, soluble 4	-2.37
ENSMUSG00000032528	Vipr1	vasoactive intestinal peptide receptor 1	-2.37
ENSMUSG00000068452	Duox2	dual oxidase 2	-2.37
ENSMUSG00000035506	Slc12a8	solute carrier family 12 (potassium/chloride transporters), member 8	-2.37
ENSMUSG00000027225	Duoxa2	dual oxidase maturation factor 2	-2.37
ENSMUSG00000038515	Grtp1	GH regulated TBC protein 1	-2.37
ENSMUSG00000006345	Ggt1	gamma-glutamyltransferase 1	-2.37
ENSMUSG00000073402	Gm8909	predicted gene 8909	-2.37
ENSMUSG00000037390	Muc3	mucin 3, intestinal	-2.37
ENSMUSG00000024503	Spink1	serine peptidase inhibitor, Kazal type 1	-2.37
ENSMUSG00000038745	Nlrp6	NLR family, pyrin domain containing 6	-2.38
ENSMUSG00000031451	Gas6	growth arrest specific 6	-2.38
ENSMUSG00000006360	Crip1	cysteine-rich protein 1 (intestinal)	-2.38
ENSMUSG00000001739	Cldn15	claudin 15	-2.38
ENSMUSG00000030865	Chp2	calcineurin-like EF hand protein 2	-2.38
ENSMUSG00000034947	Tmem106a	transmembrane protein 106A	-2.38
ENSMUSG00000038843	Gcnt1	glucosaminyl (N-acetyl) transferase 1, core 2	-2.38
ENSMUSG00000074261	Erich4	glutamate rich 4	-2.38
ENSMUSG00000084174	Sycn	syncollin	-2.38
ENSMUSG00000039438	Ttc36	tetratricopeptide repeat domain 36	-2.38
ENSMUSG00000030492	Slc7a9	solute carrier family 7 (cationic amino acid transporter, y+ system), member 9	-2.39
ENSMUSG00000091705	H2-Q2	histocompatibility 2, Q region locus 2	-2.39
ENSMUSG00000001095	Slc13a2	solute carrier family 13 (sodium-dependent dicarboxylate transporter), member 2	-2.39
ENSMUSG00000043822	Adamtsl5	ADAMTS-like 5	-2.39
ENSMUSG00000019888	Mgat4c	MGAT4 family, member C	-2.40
ENSMUSG00000051111	Sv2c	synaptic vesicle glycoprotein 2c	-2.40
ENSMUSG00000072849	Serpina1e	serine (or cysteine) peptidase inhibitor, clade A, member 1E	-2.40
ENSMUSG00000104906	Gm43005	predicted gene 43005	-2.40
ENSMUSG00000031621	Isx	intestine specific homeobox	-2.41
ENSMUSG00000026126	Ptpn18	protein tyrosine phosphatase, non-receptor type 18	-2.41
ENSMUSG00000020062	Slc5a8	solute carrier family 5 (iodide transporter), member 8	-2.41
ENSMUSG00000020839	Tmigd1	transmembrane and immunoglobulin domain containing 1	-2.41
ENSMUSG00000032978	Guca2b	guanylate cyclase activator 2b (retina)	-2.42
ENSMUSG00000039899	Fgl2	fibrinogen-like protein 2	-2.42
ENSMUSG00000010660	Plcd1	phospholipase C, delta 1	-2.42
ENSMUSG00000020072	Pbld2	phenazine biosynthesis-like protein domain containing 2	-2.42
ENSMUSG00000032357	Tinag	tubulointerstitial nephritis antigen	-2.42
ENSMUSG00000035200	Chrnb4	cholinergic receptor, nicotinic, beta polypeptide 4	-2.42
ENSMUSG00000044453	Ffar1	free fatty acid receptor 1	-2.42
ENSMUSG00000059654	Reg1	regenerating islet-derived 1	-2.42
ENSMUSG00000005672	Kit	KIT proto-oncogene receptor tyrosine kinase	-2.43
ENSMUSG00000021676	Iqgap2	IQ motif containing GTPase activating protein 2	-2.43
ENSMUSG00000031886	Ces2e	carboxylesterase 2E	-2.43
ENSMUSG00000043705	Capn13	calpain 13	-2.43
ENSMUSG00000079440	Alpi	alkaline phosphatase, intestinal	-2.43
ENSMUSG00000055730	Ces2a	carboxylesterase 2A	-2.43
ENSMUSG00000057719	Sh3rf2	SH3 domain containing ring finger 2	-2.43
ENSMUSG00000027420	Bfsp1	beaded filament structural protein 1, in lens-CP94	-2.43
ENSMUSG00000040740	Slc25a34	solute carrier family 25, member 34	-2.43
ENSMUSG00000028940	Hes2	hes family bHLH transcription factor 2	-2.43
ENSMUSG00000049122	Frmd3	FERM domain containing 3	-2.43
ENSMUSG00000023959	Clic5	chloride intracellular channel 5	-2.44
ENSMUSG00000026398	Nr5a2	nuclear receptor subfamily 5, group A, member 2	-2.44
ENSMUSG00000072812	Ahnak2	AHNAK nucleoprotein 2	-2.44
ENSMUSG00000028024	Enpep	glutamyl aminopeptidase	-2.44
ENSMUSG00000010066	Cacna2d2	calcium channel, voltage-dependent, alpha 2/delta subunit 2	-2.44
ENSMUSG00000028186	Uox	urate oxidase	-2.44
ENSMUSG00000041119	Pde9a	phosphodiesterase 9A	-2.45
ENSMUSG00000074272	Ceacam1	carcinoembryonic antigen-related cell adhesion molecule 1	-2.45
ENSMUSG00000027317	Ppp1r14d	protein phosphatase 1, regulatory inhibitor subunit 14D	-2.45
ENSMUSG00000024131	Slc3a1	solute carrier family 3, member 1	-2.45
ENSMUSG00000087651	1500009L16Rik	RIKEN cDNA 1500009L16 gene	-2.45
ENSMUSG00000025189	Cnnm1	cyclin M1	-2.45
ENSMUSG00000053182	Gm609	predicted gene 609	-2.46
ENSMUSG00000025347	Mettl7b	methyltransferase like 7B	-2.46
ENSMUSG00000090700	Cyp4f40	cytochrome P450, family 4, subfamily f, polypeptide 40	-2.46
ENSMUSG00000034785	Dio1	deiodinase, iodothyronine, type I	-2.46

ENSMUSG00000079507	H2-Q1	histocompatibility 2, Q region locus 1	-2.46
ENSMUSG00000027843	Ptpn22	protein tyrosine phosphatase, non-receptor type 22 (lymphoid)	-2.46
ENSMUSG00000028003	Lrat	lecithin-retinol acyltransferase (phosphatidylcholine-retinol-O-acyltransferase)	-2.46
ENSMUSG00000028874	Fgr	FGR proto-oncogene, Src family tyrosine kinase	-2.46
ENSMUSG00000030340	Scnn1a	sodium channel, nonvoltage-gated 1 alpha	-2.47
ENSMUSG00000062638	Btnl1	butyrophilin-like 1	-2.47
ENSMUSG00000029700	Slc13a1	solute carrier family 13 (sodium/sulfate symporters), member 1	-2.47
ENSMUSG00000053862	Slc51b	solute carrier family 51, beta subunit	-2.47
ENSMUSG00000026614	Slc30a10	solute carrier family 30, member 10	-2.47
ENSMUSG00000025401	Myo1a	myosin IA	-2.48
ENSMUSG00000026825	Dnm1	dynamin 1	-2.48
ENSMUSG00000029630	Cyp3a25	cytochrome P450, family 3, subfamily a, polypeptide 25	-2.48
ENSMUSG00000034918	Cdhr2	cadherin-related family member 2	-2.48
ENSMUSG00000029095	Ablim2	actin-binding LIM protein 2	-2.48
ENSMUSG00000045930	Clec14a	C-type lectin domain family 14, member a	-2.48
ENSMUSG00000038910	Plcl2	phospholipase C-like 2	-2.49
ENSMUSG00000027200	Sema6d	sema domain, transmembrane domain (TM), and cytoplasmic domain, (semaphorin) 6D	-2.49
ENSMUSG00000049858	Suox	sulfite oxidase	-2.49
ENSMUSG00000021565	Slc6a19	solute carrier family 6 (neurotransmitter transporter), member 19	-2.49
ENSMUSG00000029162	Khk	ketohexokinase	-2.49
ENSMUSG00000042770	Hebp1	heme binding protein 1	-2.49
ENSMUSG00000060615	Ang4	angiogenin, ribonuclease A family, member 4	-2.50
ENSMUSG00000032548	Slco2a1	solute carrier organic anion transporter family, member 2a1	-2.50
ENSMUSG00000033308	Dpyd	dihydropyrimidine dehydrogenase	-2.50
ENSMUSG00000028327	Stra6l	STRA6-like	-2.50
ENSMUSG00000040703	Cyp2s1	cytochrome P450, family 2, subfamily s, polypeptide 1	-2.50
ENSMUSG00000021947	Cryl1	crystallin, lambda 1	-2.50
ENSMUSG00000021798	Ldb3	LIM domain binding 3	-2.50
ENSMUSG00000023032	Slc4a8	solute carrier family 4 (anion exchanger), member 8	-2.50
ENSMUSG00000021278	Amn	amnionless	-2.50
ENSMUSG00000089948	Far2os1	fatty acyl CoA reductase 2, opposite strand 1	-2.50
ENSMUSG00000026380	Tfcp2l1	transcription factor CP2-like 1	-2.51
ENSMUSG00000024105	Themis3	thymocyte selection associated family member 3	-2.51
ENSMUSG00000022235	Cmbl	carboxymethylenebutenolidase-like (Pseudomonas)	-2.51
ENSMUSG00000029420	Rimbp2	RIMS binding protein 2	-2.51
ENSMUSG00000020805	Slc13a5	solute carrier family 13 (sodium-dependent citrate transporter), member 5	-2.51
ENSMUSG00000029084	Cd38	CD38 antigen	-2.52
ENSMUSG00000090124	Ugt1a7c	UDP glucuronosyltransferase 1 family, polypeptide A7C	-2.52
ENSMUSG00000108476	Gm44974	predicted gene 44974	-2.52
ENSMUSG00000021125	Arg2	arginase type II	-2.52
ENSMUSG00000057614	Gnai1	guanine nucleotide binding protein (G protein), alpha inhibiting 1	-2.52
ENSMUSG00000093580	Gm20706	predicted gene 20706	-2.52
ENSMUSG00000026544	Dusp23	dual specificity phosphatase 23	-2.52
ENSMUSG00000024292	Cyp4f14	cytochrome P450, family 4, subfamily f, polypeptide 14	-2.53
ENSMUSG00000027377	Mall	mal, T cell differentiation protein-like	-2.53
ENSMUSG00000022546	Gpt	glutamic pyruvic transaminase, soluble	-2.53
ENSMUSG00000021509	Slc25a48	solute carrier family 25, member 48	-2.53
ENSMUSG00000107456	Gm10400	predicted gene 10400	-2.53
ENSMUSG00000032226	Gcnt3	glucosaminyl (N-acetyl) transferase 3, mucin type	-2.54
ENSMUSG00000033847	Pla2g4c	phospholipase A2, group IVC (cytosolic, calcium-independent)	-2.54
ENSMUSG00000044352	Sowaha	sosondowah ankyrin repeat domain family member A	-2.54
ENSMUSG00000037440	Vnn1	vanin 1	-2.54
ENSMUSG00000027359	Slc27a2	solute carrier family 27 (fatty acid transporter), member 2	-2.54
ENSMUSG00000031163	Glod5	glyoxalase domain containing 5	-2.54
ENSMUSG00000023122	Sult1c2	sulfotransferase family, cytosolic, 1C, member 2	-2.54
ENSMUSG00000025497	Cdhr5	cadherin-related family member 5	-2.55
ENSMUSG00000024313	Mep1b	meprin 1 beta	-2.55
ENSMUSG00000055114	Anxa13	annexin A13	-2.55
ENSMUSG00000024124	Prss30	protease, serine 30	-2.55
ENSMUSG00000036151	Tm6sf2	transmembrane 6 superfamily member 2	-2.55
ENSMUSG00000042284	Itga1	integrin alpha 1	-2.55
ENSMUSG00000026928	Card9	caspase recruitment domain family, member 9	-2.55
ENSMUSG00000032487	Ptgs2	prostaglandin-endoperoxide synthase 2	-2.55
ENSMUSG00000106417	Gm42628	predicted gene 42628	-2.55
ENSMUSG00000088609	Gm24187	predicted gene, 24187	-2.56
ENSMUSG00000010601	Apol7a	apolipoprotein L 7a	-2.56
ENSMUSG00000091387	Gcnt4	glucosaminyl (N-acetyl) transferase 4, core 2 (beta-1,6-N-acetylglucosaminyltransferase)	-2.56
ENSMUSG00000033174	Mgll	monoglyceride lipase	-2.56
ENSMUSG00000061808	Ttr	transthyretin	-2.56
ENSMUSG00000031980	Agt	angiotensinogen (serpin peptidase inhibitor, clade A, member 8)	-2.57
ENSMUSG00000056162	Cndp1	carnosine dipeptidase 1 (metallopeptidase M20 family)	-2.57

ENSMUSG00000036422	Pcdh8	protocadherin 8	-2.57
ENSMUSG00000074441	Gm15292	predicted gene 15292	-2.57
ENSMUSG00000075217	4833423E24Rik	RIKEN cDNA 4833423E24 gene	-2.57
ENSMUSG00000094840	Muc3a	mucin 3A, cell surface associated	-2.58
ENSMUSG00000038751	Ptk6	PTK6 protein tyrosine kinase 6	-2.58
ENSMUSG00000109644	0610005C13Rik	RIKEN cDNA 0610005C13 gene	-2.58
ENSMUSG00000061959	Ces1e	carboxylesterase 1E	-2.58
ENSMUSG00000027790	Sis	sucrase isomaltase (alpha-glucosidase)	-2.59
ENSMUSG00000036377	C530008M17Rik	RIKEN cDNA C530008M17 gene	-2.59
ENSMUSG00000038167	Plekhg6	pleckstrin homology domain containing, family G (with RhoGef domain) member 6	-2.59
ENSMUSG00000027983	Cyp2u1	cytochrome P450, family 2, subfamily u, polypeptide 1	-2.59
ENSMUSG00000020838	Slc6a4	solute carrier family 6 (neurotransmitter transporter, serotonin), member 4	-2.59
ENSMUSG00000035429	Ptprh	protein tyrosine phosphatase, receptor type, H	-2.60
ENSMUSG00000041794	Myrip	myosin VIIA and Rab interacting protein	-2.60
ENSMUSG00000049134	Nrap	nebulin-related anchoring protein	-2.60
ENSMUSG00000049154	Fam183b	family with sequence similarity 183, member B	-2.60
ENSMUSG00000097266	Gm26802	predicted gene, 26802	-2.60
ENSMUSG00000035775	Krt20	keratin 20	-2.61
ENSMUSG00000058435	Btnl4	butyrophilin-like 4	-2.61
ENSMUSG00000036814	Slc6a20a	solute carrier family 6 (neurotransmitter transporter), member 20A	-2.61
ENSMUSG00000085042	Abhd11os	abhydrolase domain containing 11, opposite strand	-2.61
ENSMUSG00000025092	Hspa12a	heat shock protein 12A	-2.61
ENSMUSG00000027350	Chgb	chromogranin B	-2.61
ENSMUSG00000000049	Apoh	apolipoprotein H	-2.61
ENSMUSG00000015468	Notch4	notch 4	-2.62
ENSMUSG00000096546	Smlr1	small leucine-rich protein 1	-2.62
ENSMUSG00000040770	Il25	interleukin 25	-2.62
ENSMUSG00000030364	Clec2h	C-type lectin domain family 2, member h	-2.63
ENSMUSG00000001225	Slc26a3	solute carrier family 26, member 3	-2.63
ENSMUSG00000022408	Fam83f	family with sequence similarity 83, member F	-2.63
ENSMUSG00000024548	Setbp1	SET binding protein 1	-2.63
ENSMUSG00000052026	Slc6a7	solute carrier family 6 (neurotransmitter transporter, L-proline), member 7	-2.63
ENSMUSG00000021640	Naip1	NLR family, apoptosis inhibitory protein 1	-2.64
ENSMUSG00000045775	Slc16a5	solute carrier family 16 (monocarboxylic acid transporters), member 5	-2.64
ENSMUSG00000046697	Enpp7	ectonucleotide pyrophosphatase/phosphodiesterase 7	-2.64
ENSMUSG00000021675	F2rl2	coagulation factor II (thrombin) receptor-like 2	-2.64
ENSMUSG00000066176	Gm12511	predicted gene 12511	-2.64
ENSMUSG00000034427	Myo15b	myosin XVB	-2.65
ENSMUSG00000092618	Btnl6	butyrophilin-like 6	-2.65
ENSMUSG00000021208	Ifi27l2b	interferon, alpha-inducible protein 27 like 2B	-2.65
ENSMUSG00000027792	Bche	butyrylcholinesterase	-2.65
ENSMUSG00000070315	4930581F22Rik	RIKEN cDNA 4930581F22 gene	-2.65
ENSMUSG00000049892	Rasd1	RAS, dexamethasone-induced 1	-2.65
ENSMUSG00000024371	C2	complement component 2 (within H-2S)	-2.65
ENSMUSG00000078650	G6pc	glucose-6-phosphatase, catalytic	-2.65
ENSMUSG00000071356	Reg3b	regenerating islet-derived 3 beta	-2.66
ENSMUSG00000037005	Xpnpep2	X-prolyl aminopeptidase (aminopeptidase P) 2, membrane-bound	-2.66
ENSMUSG00000050592	Fam78a	family with sequence similarity 78, member A	-2.66
ENSMUSG00000058163	Gm5431	predicted gene 5431	-2.66
ENSMUSG00000031445	Proz	protein Z, vitamin K-dependent plasma glycoprotein	-2.66
ENSMUSG00000096299	Gm21814	predicted gene, 21814	-2.66
ENSMUSG00000054128	H2-T3	histocompatibility 2, T region locus 3	-2.67
ENSMUSG00000027876	Reg4	regenerating islet-derived family, member 4	-2.67
ENSMUSG00000036298	Slc2a13	solute carrier family 2 (facilitated glucose transporter), member 13	-2.67
ENSMUSG00000033715	Akr1c14	aldo-keto reductase family 1, member C14	-2.67
ENSMUSG00000046613	Vwa5b2	von Willebrand factor A domain containing 5B2	-2.67
ENSMUSG00000021194	Chga	chromogranin A	-2.67
ENSMUSG00000044505	Lingo4	leucine rich repeat and Ig domain containing 4	-2.67
ENSMUSG00000023235	Ccl25	chemokine (C-C motif) ligand 25	-2.68
ENSMUSG00000074437	Defa29	defensin, alpha, 29	-2.68
ENSMUSG00000038195	Rilp	Rab interacting lysosomal protein	-2.68
ENSMUSG00000067231	Cyp2c65	cytochrome P450, family 2, subfamily c, polypeptide 65	-2.69
ENSMUSG00000002100	Mybpc3	myosin binding protein C, cardiac	-2.69
ENSMUSG00000027513	Pck1	phosphoenolpyruvate carboxykinase 1, cytosolic	-2.70
ENSMUSG00000091575	2010016I18Rik	RIKEN cDNA 2010016I18 gene	-2.70
ENSMUSG00000018919	Tm4sf5	transmembrane 4 superfamily member 5	-2.70
ENSMUSG00000039339	Mfsd4b2	major facilitator superfamily domain containing 4B2	-2.70
ENSMUSG00000075520	Malrd1	MAM and LDL receptor class A domain containing 1	-2.71
ENSMUSG00000035699	Slc51a	solute carrier family 51, alpha subunit	-2.71
ENSMUSG00000029161	Cgref1	cell growth regulator with EF hand domain 1	-2.71
ENSMUSG00000014361	Mertk	c-mer proto-oncogene tyrosine kinase	-2.71

ENSMUSG00000021850	ccdc198	coiled-coil domain containing 198	-2.71
ENSMUSG00000031073	Fgf15	fibroblast growth factor 15	-2.71
ENSMUSG00000031497	Tnfsf13b	tumor necrosis factor (ligand) superfamily, member 13b	-2.71
ENSMUSG00000028251	Tstd3	thiosulfate sulfurtransferase (rhodanese)-like domain containing 3	-2.71
ENSMUSG00000029134	Plb1	phospholipase B1	-2.71
ENSMUSG00000029330	Cds1	CDP-diacylglycerol synthase 1	-2.72
ENSMUSG00000045441	Gprin3	GPRIN family member 3	-2.72
ENSMUSG00000060070	Defa26	defensin, alpha, 26	-2.72
ENSMUSG00000073413	Ly6g6d	lymphocyte antigen 6 complex, locus G6D	-2.72
ENSMUSG00000030017	Reg3g	regenerating islet-derived 3 gamma	-2.73
ENSMUSG00000071203	Naip5	NLR family, apoptosis inhibitory protein 5	-2.73
ENSMUSG00000029269	Sult1b1	sulfotransferase family 1B, member 1	-2.73
ENSMUSG00000024935	Slc1a1	solute carrier family 1 (neuronal/epithelial high affinity glutamate transporter, system Xag), member	-2.73
ENSMUSG00000052229	Gpr17	G protein-coupled receptor 17	-2.73
ENSMUSG00000026610	Esrrg	estrogen-related receptor gamma	-2.73
ENSMUSG00000034112	Atp2c2	ATPase, Ca++ transporting, type 2C, member 2	-2.74
ENSMUSG00000021749	Oit1	oncoprotein induced transcript 1	-2.74
ENSMUSG00000021806	Nid2	nidogen 2	-2.74
ENSMUSG00000106542	Gm43410	predicted gene 43410	-2.74
ENSMUSG00000024140	Epas1	endothelial PAS domain protein 1	-2.75
ENSMUSG00000020080	Hkdc1	hexokinase domain containing 1	-2.75
ENSMUSG00000005268	Prlr	prolactin receptor	-2.75
ENSMUSG00000027173	Depdc7	DEP domain containing 7	-2.75
ENSMUSG00000094091	Gm21885	predicted gene, 21885	-2.75
ENSMUSG00000022479	Vdr	vitamin D (1,25-dihydroxyvitamin D3) receptor	-2.76
ENSMUSG00000032038	St3gal4	ST3 beta-galactoside alpha-2,3-sialyltransferase 4	-2.76
ENSMUSG00000020865	Abcc3	ATP-binding cassette, sub-family C (CFTR/MRP), member 3	-2.76
ENSMUSG00000073420	Btnl5-ps	butyrophilin-like 5, pseudogene	-2.76
ENSMUSG00000047638	Nr1h4	nuclear receptor subfamily 1, group H, member 4	-2.76
ENSMUSG00000058921	Slc10a5	solute carrier family 10 (sodium/bile acid cotransporter family), member 5	-2.77
ENSMUSG00000096146	Kcnj11	potassium inwardly rectifying channel, subfamily J, member 11	-2.77
ENSMUSG00000074115	Saa1	serum amyloid A 1	-2.78
ENSMUSG00000031725	Ces1f	carboxylesterase 1F	-2.78
ENSMUSG00000034762	Glis1	GLIS family zinc finger 1	-2.78
ENSMUSG00000056293	Gsdmc2	gasdermin C2	-2.79
ENSMUSG00000074715	Ccl28	chemokine (C-C motif) ligand 28	-2.79
ENSMUSG00000061728	Btnl7-ps	butyrophilin-like 7, pseudogene	-2.79
ENSMUSG00000026163	Sphkap	SPHK1 interactor, AKAP domain containing	-2.79
ENSMUSG00000063296	Tmem117	transmembrane protein 117	-2.80
ENSMUSG00000073406	H2-Bl	histocompatibility 2, blastocyst	-2.80
ENSMUSG00000020732	Rab37	RAB37, member RAS oncogene family	-2.81
ENSMUSG00000058063	Trim31	tripartite motif-containing 31	-2.82
ENSMUSG00000070777	Ceacam20	carcinoembryonic antigen-related cell adhesion molecule 20	-2.82
ENSMUSG00000024846	Cst6	cystatin E/M	-2.82
ENSMUSG00000031410	Nxf7	nuclear RNA export factor 7	-2.82
ENSMUSG00000024620	Pdgfrb	platelet derived growth factor receptor, beta polypeptide	-2.82
ENSMUSG00000103356	Gm37595	predicted gene, 37595	-2.82
ENSMUSG00000031551	Ido1	indoleamine 2,3-dioxygenase 1	-2.83
ENSMUSG00000030560	Ctsc	cathepsin C	-2.85
ENSMUSG00000036492	Rnf39	ring finger protein 39	-2.85
ENSMUSG00000059003	Grin2a	glutamate receptor, ionotropic, NMDA2A (epsilon 1)	-2.85
ENSMUSG00000035277	Arx	aristaless related homeobox	-2.85
ENSMUSG00000031613	Hpgd	hydroxyprostaglandin dehydrogenase 15 (NAD)	-2.86
ENSMUSG00000024730	Ms4a8a	membrane-spanning 4-domains, subfamily A, member 8A	-2.86
ENSMUSG00000022383	Ppara	peroxisome proliferator activated receptor alpha	-2.86
ENSMUSG00000034645	Zyg11a	zyg-11 family member A, cell cycle regulator	-2.86
ENSMUSG00000052955	Cpvl	carboxypeptidase, vitellogenic-like	-2.86
ENSMUSG00000011008	Mcoln2	mucolipin 2	-2.87
ENSMUSG00000108155	Gm44443	predicted gene, 44443	-2.87
ENSMUSG00000001604	Tcea3	transcription elongation factor A (SII), 3	-2.88
ENSMUSG00000022661	Cd200	CD200 antigen	-2.88
ENSMUSG00000079355	Ackr4	atypical chemokine receptor 4	-2.88
ENSMUSG00000064177	Ghrl	ghrelin	-2.88
ENSMUSG00000022489	Pde1b	phosphodiesterase 1B, Ca2+-calmodulin dependent	-2.88
ENSMUSG00000021070	Bdkrb2	bradykinin receptor, beta 2	-2.88
ENSMUSG00000089635	Gm16559	predicted gene 16559	-2.88
ENSMUSG00000044860	Gm1123	predicted gene 1123	-2.89
ENSMUSG00000098557	Kctd12	potassium channel tetramerisation domain containing 12	-2.89
ENSMUSG00000027661	Slc2a10	solute carrier family 2 (facilitated glucose transporter), member 10	-2.89
ENSMUSG00000116383	Apol10c-ps	apolipoprotein L 10C, pseudogene	-2.89
ENSMUSG00000002565	Scin	scinderin	-2.90

ENSMUSG00000090062	Galnt6os	polypeptide N-acetylgalactosaminyltransferase 6, opposite strand	-2.90
ENSMUSG00000022755	Adgrg7	adhesion G protein-coupled receptor G7	-2.91
ENSMUSG00000061531	Tmem236	transmembrane protein 236	-2.91
ENSMUSG00000074207	Adh1	alcohol dehydrogenase 1 (class I)	-2.91
ENSMUSG00000039878	Slc39a5	solute carrier family 39 (metal ion transporter), member 5	-2.91
ENSMUSG00000030472	Ceacam18	carcinoembryonic antigen-related cell adhesion molecule 18	-2.91
ENSMUSG00000006642	Tcf23	transcription factor 23	-2.92
ENSMUSG00000072623	Zfp9	zinc finger protein 9	-2.92
ENSMUSG00000033200	Tpsg1	tryptase gamma 1	-2.93
ENSMUSG00000030737	Slco2b1	solute carrier organic anion transporter family, member 2b1	-2.93
ENSMUSG00000064272	Gpbar1	G protein-coupled bile acid receptor 1	-2.93
ENSMUSG00000037953	A4gnt	alpha-1,4-N-acetylglucosaminyltransferase	-2.94
ENSMUSG00000027224	Duoxa1	dual oxidase maturation factor 1	-2.94
ENSMUSG00000067377	Tspan6	tetraspanin 6	-2.95
ENSMUSG00000041696	Rasl12	RAS-like, family 12	-2.95
ENSMUSG00000040653	Ppp1r14c	protein phosphatase 1, regulatory inhibitor subunit 14C	-2.95
ENSMUSG00000032181	Scg3	secretogranin III	-2.96
ENSMUSG00000087518	Gm13561	predicted gene 13561	-2.96
ENSMUSG00000050097	Ces2b	carboxyesterase 2B	-2.97
ENSMUSG00000025010	Ccnj	cyclin J	-2.98
ENSMUSG00000023247	Guca2a	guanylate cyclase activator 2a (guanylin)	-2.98
ENSMUSG00000078776	9530053A07Rik	RIKEN cDNA 9530053A07 gene	-2.98
ENSMUSG00000046804	Phgr1	proline/histidine/glycine-rich 1	-2.99
ENSMUSG00000021216	Tubal3	tubulin, alpha-like 3	-2.99
ENSMUSG00000055748	Gsdmc4	gasdermin C4	-3.00
ENSMUSG00000029273	Sult1d1	sulfotransferase family 1D, member 1	-3.00
ENSMUSG00000026870	Cutal	cutA divalent cation tolerance homolog-like	-3.00
ENSMUSG00000020120	Plek	pleckstrin	-3.00
ENSMUSG00000013643	Lypd8	LY6/PLAUR domain containing 8	-3.01
ENSMUSG00000094584	Ms4a18	membrane-spanning 4-domains, subfamily A, member 18	-3.02
ENSMUSG00000110405	Gm45534	predicted gene 45534	-3.02
ENSMUSG00000024340	Btnl2	butyrophilin-like 2	-3.03
ENSMUSG00000079180	Mptx2	mucosal pentraxin 2	-3.04
ENSMUSG00000072723	Gm10044	predicted gene 10044	-3.04
ENSMUSG00000047564	Krtap3-1	keratin associated protein 3-1	-3.04
ENSMUSG00000035849	Krt222	keratin 222	-3.05
ENSMUSG00000027220	Syt13	synaptotagmin XIII	-3.05
ENSMUSG00000034472	Rasd2	RASD family, member 2	-3.07
ENSMUSG00000041476	Smpx	small muscle protein, X-linked	-3.07
ENSMUSG00000024553	Galr1	galanin receptor 1	-3.07
ENSMUSG00000029335	Bmp3	bone morphogenetic protein 3	-3.08
ENSMUSG00000078137	Ankrd63	ankyrin repeat domain 63	-3.09
ENSMUSG00000034057	Myrfl	myelin regulatory factor-like	-3.10
ENSMUSG00000054640	Slc8a1	solute carrier family 8 (sodium/calcium exchanger), member 1	-3.10
ENSMUSG00000024215	Spdef	SAM pointed domain containing ets transcription factor	-3.11
ENSMUSG00000032202	Rab27a	RAB27A, member RAS oncogene family	-3.12
ENSMUSG00000060508	Nlrp9b	NLR family, pyrin domain containing 9B	-3.12
ENSMUSG00000050296	Abca12	ATP-binding cassette, sub-family A (ABC1), member 12	-3.12
ENSMUSG00000038599	Capn8	calpain 8	-3.13
ENSMUSG00000060224	Pyroxd2	pyridine nucleotide-disulphide oxidoreductase domain 2	-3.13
ENSMUSG00000027801	Tm4sf4	transmembrane 4 superfamily member 4	-3.14
ENSMUSG00000030207	Fam234b	family with sequence similarity 234, member B	-3.14
ENSMUSG00000055235	Wdr86	WD repeat domain 86	-3.14
ENSMUSG00000049036	Tmem121	transmembrane protein 121	-3.14
ENSMUSG00000055827	Gsdmc3	gasdermin C3	-3.15
ENSMUSG00000040121	Rep15	RAB15 effector protein	-3.15
ENSMUSG00000038580	Sct	secretin	-3.16
ENSMUSG00000027254	Map1a	microtubule-associated protein 1 A	-3.16
ENSMUSG00000040432	Ltb4r2	leukotriene B4 receptor 2	-3.16
ENSMUSG00000078942	Naip6	NLR family, apoptosis inhibitory protein 6	-3.17
ENSMUSG00000031891	Hsd11b2	hydroxysteroid 11-beta dehydrogenase 2	-3.17
ENSMUSG00000054385	Ceacam2	carcinoembryonic antigen-related cell adhesion molecule 2	-3.17
ENSMUSG00000023092	Fhl1	four and a half LIM domains 1	-3.17
ENSMUSG00000089669	Tnfsf13	tumor necrosis factor (ligand) superfamily, member 13	-3.17
ENSMUSG00000110195	Pde2a	phosphodiesterase 2A, cGMP-stimulated	-3.17
ENSMUSG00000026834	Acvr1c	activin A receptor, type IC	-3.18
ENSMUSG00000096852	Cyp2d12	cytochrome P450, family 2, subfamily d, polypeptide 12	-3.19
ENSMUSG00000029445	Hpd	4-hydroxyphenylpyruvic acid dioxygenase	-3.20
ENSMUSG00000017311	Pyy	peptide YY	-3.20
ENSMUSG00000053825	Ppfia2	protein tyrosine phosphatase, receptor type, f polypeptide (PTPRF), interacting protein (liprin), alpha	-3.21
ENSMUSG00000070704	Ugt2b36	UDP glucuronosyltransferase 2 family, polypeptide B36	-3.21

ENSMUSG00000020589	Fam49a	family with sequence similarity 49, member A	-3.22
ENSMUSG00000034107	Ano7	anoctamin 7	-3.23
ENSMUSG00000056220	Pla2g4a	phospholipase A2, group IVA (cytosolic, calcium-dependent)	-3.24
ENSMUSG00000068154	Insm1	insulinoma-associated 1	-3.24
ENSMUSG00000047528	Als2cr12	amyotrophic lateral sclerosis 2 chromosome region 12	-3.24
ENSMUSG00000108950	9130015G15Rik	RIKEN cDNA 9130015G15 gene	-3.24
ENSMUSG00000026832	Cytip	cytohesin 1 interacting protein	-3.26
ENSMUSG00000085651	Gm11695	predicted gene 11695	-3.27
ENSMUSG00000040896	Kcnd3	potassium voltage-gated channel, Shal-related family, member 3	-3.28
ENSMUSG00000052861	Dnah6	dynein, axonemal, heavy chain 6	-3.28
ENSMUSG00000107296	Gm43500	predicted gene 43500	-3.28
ENSMUSG00000095649	Gm8979	predicted gene 8979	-3.29
ENSMUSG00000047517	Dmbt1	deleted in malignant brain tumors 1	-3.30
ENSMUSG00000048217	Nags	N-acetylglutamate synthase	-3.30
ENSMUSG00000028415	Spink4	serine peptidase inhibitor, Kazal type 4	-3.30
ENSMUSG00000073043	Atoh1	atonal bHLH transcription factor 1	-3.31
ENSMUSG00000030911	Zp2	zona pellucida glycoprotein 2	-3.31
ENSMUSG00000037106	Fer1l6	fer-1-like 6 (C. elegans)	-3.32
ENSMUSG00000042115	Klhdc8a	kelch domain containing 8A	-3.32
ENSMUSG00000015966	Il17rb	interleukin 17 receptor B	-3.33
ENSMUSG00000058618	AY761184	cDNA sequence AY761184	-3.33
ENSMUSG00000074196	Clca4c-ps	chloride channel accessory 4C, pseudogene	-3.33
ENSMUSG00000111377	Gm40634	predicted gene, 40634	-3.33
ENSMUSG00000031173	Otc	ornithine transcarbamylase	-3.34
ENSMUSG00000033740	St18	suppression of tumorigenicity 18	-3.34
ENSMUSG00000053004	Hrh1	histamine receptor H1	-3.35
ENSMUSG00000026815	Gfi1b	growth factor independent 1B	-3.35
ENSMUSG00000036027	1810046K07Rik	RIKEN cDNA 1810046K07 gene	-3.36
ENSMUSG00000063903	Klk1	kallikrein 1	-3.37
ENSMUSG00000109685	Gm45912	predicted gene 45912	-3.37
ENSMUSG00000095026	Gm3336	predicted gene 3336	-3.37
ENSMUSG00000060176	Kif27	kinesin family member 27	-3.37
ENSMUSG00000101031	Ms4a12	membrane-spanning 4-domains, subfamily A, member 12	-3.39
ENSMUSG00000055567	Unc80	unc-80, NALCN activator	-3.39
ENSMUSG00000111340	Gm47171	predicted gene, 47171	-3.40
ENSMUSG00000042351	Grap2	GRB2-related adaptor protein 2	-3.40
ENSMUSG00000019890	Nts	neurotensin	-3.41
ENSMUSG00000050556	Kcnb1	potassium voltage gated channel, Shab-related subfamily, member 1	-3.41
ENSMUSG00000020581	Agr2	anterior gradient 2	-3.43
ENSMUSG00000020279	Il9r	interleukin 9 receptor	-3.43
ENSMUSG00000008193	Spib	Spi-B transcription factor (Spi-1/PU.1 related)	-3.44
ENSMUSG00000050824	Sstr5	somatostatin receptor 5	-3.44
ENSMUSG00000034353	Ramp1	receptor (calcitonin) activity modifying protein 1	-3.45
ENSMUSG00000032015	Pou2f3	POU domain, class 2, transcription factor 3	-3.45
ENSMUSG00000096751	Gm28373	predicted gene 28373	-3.46
ENSMUSG00000050100	Hmx2	H6 homeobox 2	-3.46
ENSMUSG00000040680	Kremen2	kringle containing transmembrane protein 2	-3.47
ENSMUSG00000025991	Cps1	carbamoyl-phosphate synthetase 1	-3.48
ENSMUSG00000102615	Gm37844	predicted gene, 37844	-3.48
ENSMUSG00000087801	Gm25980	predicted gene, 25980	-3.49
ENSMUSG00000038665	Dgki	diacylglycerol kinase, iota	-3.50
ENSMUSG00000040136	Abcc8	ATP-binding cassette, sub-family C (CFTR/MRP), member 8	-3.50
ENSMUSG00000114576	Naip3-ps1	NLR family, apoptosis inhibitory protein 3, pseudogene 1	-3.51
ENSMUSG00000034701	Neurod1	neurogenic differentiation 1	-3.51
ENSMUSG00000004952	Rasa4	RAS p21 protein activator 4	-3.52
ENSMUSG00000026828	Galnt5	polypeptide N-acetylgalactosaminyltransferase 5	-3.52
ENSMUSG00000056553	Ptprn2	protein tyrosine phosphatase, receptor type, N polypeptide 2	-3.52
ENSMUSG00000027296	Itpka	inositol 1,4,5-trisphosphate 3-kinase A	-3.52
ENSMUSG00000022840	Adcy5	adenylate cyclase 5	-3.55
ENSMUSG00000026811	St6galnac6	ST6 (alpha-N-acetyl-neuraminyl-2,3-beta-galactosyl-1,3)-N-acetylgalactosaminide alpha-2,6-sialyltran	-3.56
ENSMUSG00000046215	Rprml	reprimo-like	-3.56
ENSMUSG00000049350	Zg16	zymogen granule protein 16	-3.58
ENSMUSG00000047730	Fcgbp	Fc fragment of IgG binding protein	-3.59
ENSMUSG00000027762	Sucnr1	succinate receptor 1	-3.59
ENSMUSG00000110397	Gm45540	predicted gene 45540	-3.59
ENSMUSG00000012819	Cdh23	cadherin 23 (otocadherin)	-3.59
ENSMUSG00000027797	Dclk1	doublecortin-like kinase 1	-3.60
ENSMUSG00000044156	Hepacam2	HEPACAM family member 2	-3.61
ENSMUSG00000051606	2010001K21Rik	RIKEN cDNA 2010001K21 gene	-3.61
ENSMUSG00000086513	9130208D14Rik	RIKEN cDNA 9130208D14 gene	-3.63
ENSMUSG00000022262	Dnah5	dynein, axonemal, heavy chain 5	-3.63

ENSMUSG00000020901	Pik3r5	phosphoinositide-3-kinase regulatory subunit 5	-3.64
ENSMUSG00000032561	Acpp	acid phosphatase, prostate	-3.64
ENSMUSG00000106651	Gm42608	predicted gene 42608	-3.65
ENSMUSG00000039264	Gimap3	GTPase, IMAP family member 3	-3.66
ENSMUSG00000035930	Chst4	carbohydrate (chondroitin 6/keratan) sulfotransferase 4	-3.67
ENSMUSG00000024029	Tff3	trefoil factor 3, intestinal	-3.68
ENSMUSG00000046314	Stxbp6	syntaxin binding protein 6 (amisyn)	-3.68
ENSMUSG00000028255	Clca1	chloride channel accessory 1	-3.69
ENSMUSG00000094219	Calhm3	calcium homeostasis modulator 3	-3.69
ENSMUSG00000020788	Atp2a3	ATPase, Ca++ transporting, ubiquitous	-3.71
ENSMUSG00000040061	Plcb2	phospholipase C, beta 2	-3.71
ENSMUSG00000022148	Fyb	FYN binding protein	-3.72
ENSMUSG00000060063	Alox5ap	arachidonate 5-lipoxygenase activating protein	-3.72
ENSMUSG00000022686	B3gnt5	UDP-GlcNAc:betaGal beta-1,3-N-acetylglucosaminyltransferase 5	-3.76
ENSMUSG00000032036	Kirrel3	kirre like nephrin family adhesion molecule 3	-3.77
ENSMUSG00000044359	P2ry4	pyrimidinergic receptor P2Y, G-protein coupled, 4	-3.78
ENSMUSG00000050321	Neto1	neuropilin (NRP) and tolloid (TLL)-like 1	-3.78
ENSMUSG00000021730	Hcn1	hyperpolarization-activated, cyclic nucleotide-gated K+ 1	-3.79
ENSMUSG00000029865	Sval1	seminal vesicle antigen-like 1	-3.79
ENSMUSG00000009246	Trpm5	transient receptor potential cation channel, subfamily M, member 5	-3.81
ENSMUSG00000071178	Serpina1b	serine (or cysteine) preptidase inhibitor, clade A, member 1B	-3.81
ENSMUSG00000021700	Rab3c	RAB3C, member RAS oncogene family	-3.81
ENSMUSG00000054422	Fabp1	fatty acid binding protein 1, liver	-3.82
ENSMUSG00000025515	Muc2	mucin 2	-3.83
ENSMUSG00000046460	Sh2d7	SH2 domain containing 7	-3.86
ENSMUSG00000028996	Rbp7	retinol binding protein 7, cellular	-3.87
ENSMUSG00000024366	Gfra3	glial cell line derived neurotrophic factor family receptor alpha 3	-3.88
ENSMUSG00000004933	Matk	megakaryocyte-associated tyrosine kinase	-3.89
ENSMUSG00000026288	Inpp5d	inositol polyphosphate-5-phosphatase D	-3.90
ENSMUSG00000109237	9130214F15Rik	RIKEN cDNA 9130214F15 gene	-3.90
ENSMUSG00000082956	Naip3	NLR family, apoptosis inhibitory protein 3	-3.91
ENSMUSG00000116929	AC164088.2	TEC	-3.91
ENSMUSG00000114443	Gm19241	predicted gene, 19241	-3.93
ENSMUSG00000001827	Folr1	folate receptor 1 (adult)	-3.95
ENSMUSG00000084384	Gm12251	predicted gene 12251	-3.95
ENSMUSG00000026395	Ptprc	protein tyrosine phosphatase, receptor type, C	-3.96
ENSMUSG00000030302	Atp2b2	ATPase, Ca++ transporting, plasma membrane 2	-3.96
ENSMUSG00000031377	Bmx	BMX non-receptor tyrosine kinase	-3.97
ENSMUSG00000029797	Sspo	SCO-spondin	-3.97
ENSMUSG00000025701	Alox5	arachidonate 5-lipoxygenase	-3.98
ENSMUSG00000030263	Lrmp	lymphoid-restricted membrane protein	-4.00
ENSMUSG00000018927	Ccl6	chemokine (C-C motif) ligand 6	-4.01
ENSMUSG00000031981	Capn9	calpain 9	-4.02
ENSMUSG00000027168	Pax6	paired box 6	-4.04
ENSMUSG00000051079	Rgs13	regulator of G-protein signaling 13	-4.05
ENSMUSG00000050711	Scg2	secretogranin II	-4.06
ENSMUSG00000090254	Gm1965	predicted gene 1965	-4.08
ENSMUSG00000058216	Gstp3	glutathione S-transferase pi 3	-4.09
ENSMUSG00000057400	Ces1c	carboxylesterase 1C	-4.09
ENSMUSG00000022026	Olfm4	olfactomedin 4	-4.12
ENSMUSG00000021069	Pygl	liver glycogen phosphorylase	-4.12
ENSMUSG00000051497	Kcnj16	potassium inwardly-rectifying channel, subfamily J, member 16	-4.14
ENSMUSG00000105302	Gm19817	predicted gene, 19817	-4.14
ENSMUSG00000039629	Strip2	striatin interacting protein 2	-4.16
ENSMUSG00000033220	Rac2	Rac family small GTPase 2	-4.17
ENSMUSG00000042010	Acacb	acetyl-Coenzyme A carboxylase beta	-4.26
ENSMUSG00000026531	Mptx1	mucosal pentraxin 1	-4.26
ENSMUSG00000040035	Disp2	dispatched RND tramsporter family member 2	-4.28
ENSMUSG00000040148	Hmx3	H6 homeobox 3	-4.31
ENSMUSG00000105975	Gm42609	predicted gene 42609	-4.31
ENSMUSG00000003283	Hck	hemopoietic cell kinase	-4.32
ENSMUSG00000029878	Dbpht2	DNA binding protein with his-thr domain	-4.32
ENSMUSG00000052631	Sh2d6	SH2 domain containing 6	-4.34
ENSMUSG00000064140	Trim38	tripartite motif-containing 38	-4.35
ENSMUSG00000019429	Ffar3	free fatty acid receptor 3	-4.38
ENSMUSG00000028339	Col15a1	collagen, type XV, alpha 1	-4.42
ENSMUSG00000040380	Cbln3	cerebellin 3 precursor protein	-4.43
ENSMUSG00000032826	Ank2	ankyrin 2, brain	-4.53
ENSMUSG00000037973	Ccdc129	coiled-coil domain containing 129	-4.54
ENSMUSG00000047798	Cd300lf	CD300 molecule like family member F	-4.57
ENSMUSG00000020787	P2rx1	purinergic receptor P2X, ligand-gated ion channel, 1	-4.64

ENSMUSG00000039013	Siglecf	sialic acid binding Ig-like lectin F	-4.65
ENSMUSG00000096630	Vmn2r26	vomeronasal 2, receptor 26	-4.68
ENSMUSG00000033569	Adgrb3	adhesion G protein-coupled receptor B3	-4.68
ENSMUSG00000047497	Adamts12	a disintegrin-like and metallopeptidase (reprolysin type) with thrombospondin type 1 motif, 12	-4.81
ENSMUSG00000034923	Ly6g6f	lymphocyte antigen 6 complex, locus G6F	-4.82
ENSMUSG00000106354	Gm42607	predicted gene 42607	-4.83
ENSMUSG00000115816	Gm34589	predicted gene, 34589	-4.95
ENSMUSG00000037627	Rgs22	regulator of G-protein signalling 22	-5.06
ENSMUSG00000028777	Gnat3	guanine nucleotide binding protein, alpha transducing 3	-5.06
ENSMUSG00000022650	Retnlb	resistin like beta	-5.10
ENSMUSG00000021919	Chat	choline acetyltransferase	-5.28
ENSMUSG00000046318	Ccbe1	collagen and calcium binding EGF domains 1	-5.28
ENSMUSG00000032532	Cck	cholecystokinin	-5.41
ENSMUSG00000021536	Adcy2	adenylate cyclase 2	-5.55
ENSMUSG00000026938	Fcna	ficolin A	-6.75
ENSMUSG00000014351	Gip	gastric inhibitory polypeptide	-6.98
ENSMUSG00000000394	Gcg	glucagon	-7.26
ENSMUSG00000053852	Adgrg4	adhesion G protein-coupled receptor G4	-7.27
ENSMUSG00000026532	Spta1	spectrin alpha, erythrocytic 1	-7.38

Tom+Trop2+ vs. differentiated cells

Ensemble gene code	Symbol	Gene name	logFC
ENSMUSG00000031841	Cdh13	cadherin 13	11.80
ENSMUSG00000029838	Ptn	pleiotrophin	10.90
ENSMUSG00000028031	Dkk2	dickkopf WNT signaling pathway inhibitor 2	10.70
ENSMUSG00000036480	Prss56	protease, serine 56	10.30
ENSMUSG00000053613	Notumos	notum palmitoleoyl-protein carboxylesterase, opposite strand	10.20
ENSMUSG00000037035	Inhbb	inhibin beta-B	10.20
ENSMUSG00000025330	Padi4	peptidyl arginine deiminase, type IV	10.10
ENSMUSG00000029368	Alb	albumin	10.00
ENSMUSG00000026167	Wnt10a	wingless-type MMTV integration site family, member 10A	9.81
ENSMUSG00000017491	Rarb	retinoic acid receptor, beta	9.68
ENSMUSG00000063632	Sox11	SRY (sex determining region Y)-box 11	9.52
ENSMUSG00000040852	Plekhh2	pleckstrin homology domain containing, family H (with MyTH4 domain) member 2	9.38
ENSMUSG00000029648	Flt1	FMS-like tyrosine kinase 1	9.29
ENSMUSG00000028360	Slc44a5	solute carrier family 44, member 5	9.24
ENSMUSG00000031871	Cdh5	cadherin 5	9.22
ENSMUSG00000022883	Robo1	roundabout guidance receptor 1	9.05
ENSMUSG00000035615	Frmpd1	FERM and PDZ domain containing 1	8.94
ENSMUSG00000042268	Slc26a9	solute carrier family 26, member 9	8.87
ENSMUSG00000110290	Gm45336	predicted gene 45336	8.87
ENSMUSG00000032965	Ift57	intraflagellar transport 57	8.85
ENSMUSG00000048960	Prex2	phosphatidylinositol-3,4,5-trisphosphate-dependent Rac exchange factor 2	8.82
ENSMUSG00000051397	Tacstd2	tumor-associated calcium signal transducer 2	8.81
ENSMUSG00000020181	Nav3	neuron navigator 3	8.81
ENSMUSG00000068699	Flnc	filamin C, gamma	8.79
ENSMUSG00000020388	Pdlim4	PDZ and LIM domain 4	8.70
ENSMUSG00000005503	Evx1	even-skipped homeobox 1	8.70
ENSMUSG00000054263	Lifr	leukemia inhibitory factor receptor	8.69
ENSMUSG00000039232	Stx11	syntaxin 11	8.66
ENSMUSG00000055322	Tns1	tensin 1	8.50
ENSMUSG00000041046	Ramp3	receptor (calcitonin) activity modifying protein 3	8.46
ENSMUSG00000013584	Aldh1a2	aldehyde dehydrogenase family 1, subfamily A2	8.46
ENSMUSG00000079330	Lemd1	LEM domain containing 1	8.43
ENSMUSG00000000120	Ngfr	nerve growth factor receptor (TNFR superfamily, member 16)	8.42
ENSMUSG00000032936	Camkv	CaM kinase-like vesicle-associated	8.37
ENSMUSG00000026117	Zap70	zeta-chain (TCR) associated protein kinase	8.30
ENSMUSG00000016763	Scube1	signal peptide, CUB domain, EGF-like 1	8.28
ENSMUSG00000023885	Thbs2	thrombospondin 2	8.21
ENSMUSG00000059854	Hydin	HYDIN, axonemal central pair apparatus protein	8.21
ENSMUSG00000071531	Gprin2	G protein regulated inducer of neurite outgrowth 2	8.19
ENSMUSG00000110187	Gm45496	predicted gene 45496	8.17
ENSMUSG00000022754	Tmem45a	transmembrane protein 45a	8.17
ENSMUSG00000054555	Adam12	a disintegrin and metallopeptidase domain 12 (meltrin alpha)	8.15
ENSMUSG00000034460	Six4	sine oculis-related homeobox 4	8.11
ENSMUSG00000004552	Ctse	cathepsin E	8.05
ENSMUSG00000084822	Myadml2os	myeloid-associated differentiation marker-like 2, opposite strand	8.03
ENSMUSG00000034634	Ly6d	lymphocyte antigen 6 complex, locus D	7.98
ENSMUSG00000029869	Ephb6	Eph receptor B6	7.95
ENSMUSG00000063531	Sema3e	sema domain, immunoglobulin domain (Ig), short basic domain, secreted, (semaphorin) 3E	7.87
ENSMUSG00000012428	Steap4	STEAP family member 4	7.86
ENSMUSG00000022037	Clu	clusterin	7.85
ENSMUSG00000044313	Mab21l3	mab-21-like 3 (C. elegans)	7.74
ENSMUSG00000020218	Wif1	Wnt inhibitory factor 1	7.67
ENSMUSG00000070867	Trabd2b	TraB domain containing 2B	7.67
ENSMUSG00000033377	Palmd	palmdelphin	7.65
ENSMUSG00000013415	Igf2bp1	insulin-like growth factor 2 mRNA binding protein 1	7.61
ENSMUSG00000062591	Tubb4a	tubulin, beta 4A class IVA	7.60
ENSMUSG00000032719	Sbspon	somatomedin B and thrombospondin, type 1 domain containing	7.48
ENSMUSG00000043903	Zfp469	zinc finger protein 469	7.47
ENSMUSG00000030772	Dkk3	dickkopf WNT signaling pathway inhibitor 3	7.45
ENSMUSG00000049848	Ceacam19	carcinoembryonic antigen-related cell adhesion molecule 19	7.40
ENSMUSG00000041134	Cyyr1	cysteine and tyrosine-rich protein 1	7.37
ENSMUSG00000025491	Ifitm1	interferon induced transmembrane protein 1	7.37
ENSMUSG00000018211	Wfdc15b	WAP four-disulfide core domain 15B	7.37
ENSMUSG00000038415	Foxq1	forkhead box Q1	7.34
ENSMUSG00000029223	Uchl1	ubiquitin carboxy-terminal hydrolase L1	7.29
ENSMUSG00000022146	Osmr	oncostatin M receptor	7.29
ENSMUSG00000042988	Notum	notum palmitoleoyl-protein carboxylesterase	7.28
ENSMUSG00000031074	Fgf3	fibroblast growth factor 3	7.24
ENSMUSG00000032502	Stac	src homology three (SH3) and cysteine rich domain	7.23
ENSMUSG00000038572	Bpifb5	BPI fold containing family B, member 5	7.22
ENSMUSG00000031284	Pak3	p21 (RAC1) activated kinase 3	7.17
ENSMUSG00000031503	Col4a2	collagen, type IV, alpha 2	7.17
ENSMUSG00000022464	Slc38a4	solute carrier family 38, member 4	7.16

ENSMUSG00000034463	Scara3	scavenger receptor class A, member 3	7.13
ENSMUSG00000036599	Chst12	carbohydrate sulfotransferase 12	7.08
ENSMUSG00000026822	Lcn2	lipocalin 2	7.05
ENSMUSG00000021091	Serpina3n	serine (or cysteine) peptidase inhibitor, clade A, member 3N	7.01
ENSMUSG00000037712	Fermt2	fermitin family member 2	6.97
ENSMUSG00000059898	Dsc3	desmocollin 3	6.97
ENSMUSG00000014813	Stc1	stanniocalcin 1	6.97
ENSMUSG00000020363	Gfpt2	glutamine fructose-6-phosphate transaminase 2	6.96
ENSMUSG00000056856	Jakmip3	janus kinase and microtubule interacting protein 3	6.96
ENSMUSG00000048078	Tenm4	teneurin transmembrane protein 4	6.94
ENSMUSG00000029371	Cxcl5	chemokine (C-X-C motif) ligand 5	6.94
ENSMUSG00000020037	Rfx4	regulatory factor X, 4 (influences HLA class II expression)	6.94
ENSMUSG00000108218	Olfr1372-ps1	olfactory receptor 1372, pseudogene 1	6.90
ENSMUSG00000031661	Nkd1	naked cuticle 1	6.89
ENSMUSG00000053007	Creb5	cAMP responsive element binding protein 5	6.86
ENSMUSG00000032717	Mdfi	MyoD family inhibitor	6.85
ENSMUSG00000054932	Afp	alpha fetoprotein	6.85
ENSMUSG00000085811	Cep112it	centrosomal protein 112, intronic transcript	6.77
ENSMUSG00000015134	Aldh1a3	aldehyde dehydrogenase family 1, subfamily A3	6.76
ENSMUSG00000032327	Stra6	stimulated by retinoic acid gene 6	6.74
ENSMUSG00000044071	Fam19a2	family with sequence similarity 19, member A2	6.73
ENSMUSG00000061048	Cdh3	cadherin 3	6.71
ENSMUSG00000014599	Csf1	colony stimulating factor 1 (macrophage)	6.70
ENSMUSG00000110666	Gm9172	predicted gene 9172	6.69
ENSMUSG00000004951	Hspb1	heat shock protein 1	6.68
ENSMUSG00000032186	Tmod2	tropomodulin 2	6.67
ENSMUSG00000031736	Crnde	colorectal neoplasia differentially expressed (non-protein coding)	6.66
ENSMUSG00000006205	Htra1	HtrA serine peptidase 1	6.64
ENSMUSG00000024810	Il33	interleukin 33	6.59
ENSMUSG00000001864	Aif1l	allograft inflammatory factor 1-like	6.59
ENSMUSG00000049866	Arl4c	ADP-ribosylation factor-like 4C	6.58
ENSMUSG00000042734	Ttc9	tetratricopeptide repeat domain 9	6.58
ENSMUSG00000046623	Gjb4	gap junction protein, beta 4	6.57
ENSMUSG00000027376	Prom2	prominin 2	6.56
ENSMUSG00000000901	Mmp11	matrix metallopeptidase 11	6.55
ENSMUSG00000040488	Ltbp4	latent transforming growth factor beta binding protein 4	6.54
ENSMUSG00000031595	Pdgfrl	platelet-derived growth factor receptor-like	6.54
ENSMUSG00000022176	Rem2	rad and gem related GTP binding protein 2	6.54
ENSMUSG00000000983	Wfdc18	WAP four-disulfide core domain 18	6.53
ENSMUSG00000019577	Pdk4	pyruvate dehydrogenase kinase, isoenzyme 4	6.53
ENSMUSG00000037016	Frem2	Fras1 related extracellular matrix protein 2	6.51
ENSMUSG00000029769	Ccdc136	coiled-coil domain containing 136	6.48
ENSMUSG00000029108	Pcdh7	protocadherin 7	6.47
ENSMUSG00000046169	Adamts6	a disintegrin-like and metallopeptidase (reprolysin type) with thrombospondin type 1 motif, 6	6.47
ENSMUSG00000001131	Timp1	tissue inhibitor of metalloproteinase 1	6.45
ENSMUSG00000042357	Gjb5	gap junction protein, beta 5	6.44
ENSMUSG00000025352	Gdf11	growth differentiation factor 11	6.42
ENSMUSG00000032643	Fhl3	four and a half LIM domains 3	6.39
ENSMUSG00000021253	Tgfb3	transforming growth factor, beta 3	6.37
ENSMUSG00000051159	Cited1	Cbp/p300-interacting transactivator with Glu/Asp-rich carboxy-terminal domain 1	6.36
ENSMUSG00000022995	Enah	ENAH actin regulator	6.36
ENSMUSG00000025202	Scd3	stearoyl-coenzyme A desaturase 3	6.36
ENSMUSG00000051236	Msrb3	methionine sulfoxide reductase B3	6.35
ENSMUSG00000097651	4930461G14Rik	RIKEN cDNA 4930461G14 gene	6.35
ENSMUSG00000058297	Spock2	sparc/osteonectin, cwcv and kazal-like domains proteoglycan 2	6.33
ENSMUSG00000024268	Celf4	CUGBP, Elav-like family member 4	6.32
ENSMUSG00000042179	Pnliprp1	pancreatic lipase related protein 1	6.31
ENSMUSG00000021822	Plau	plasminogen activator, urokinase	6.31
ENSMUSG00000040836	Gpr161	G protein-coupled receptor 161	6.29
ENSMUSG00000025473	Adam8	a disintegrin and metallopeptidase domain 8	6.28
ENSMUSG00000037129	Tmprss13	transmembrane protease, serine 13	6.22
ENSMUSG00000022425	Enpp2	ectonucleotide pyrophosphatase/phosphodiesterase 2	6.22
ENSMUSG00000052957	Gas1	growth arrest specific 1	6.20
ENSMUSG00000062327	T	brachyury, T-box transcription factor T	6.19
ENSMUSG00000038860	Garnl3	GTPase activating RANGAP domain-like 3	6.18
ENSMUSG00000034220	Gpc1	glypican 1	6.16
ENSMUSG00000049807	Arhgap23	Rho GTPase activating protein 23	6.15
ENSMUSG00000034450	Gulo	gulonolactone (L-) oxidase	6.15
ENSMUSG00000097365	C030034L19Rik	RIKEN cDNA C030034L19 gene	6.15
ENSMUSG00000001870	Ltbp1	latent transforming growth factor beta binding protein 1	6.12
ENSMUSG00000063727	Tnfrsf11b	tumor necrosis factor receptor superfamily, member 11b (osteoprotegerin)	6.08
ENSMUSG00000026764	Kif5c	kinesin family member 5C	6.08
ENSMUSG00000015053	Gata2	GATA binding protein 2	6.08
ENSMUSG00000103738	Gm37652	predicted gene, 37652	6.06
ENSMUSG00000066607	Insyn1	inhibitory synaptic factor 1	6.01
ENSMUSG00000050052	Tdrp	testis development related protein	6.00

ENSMUSG00000050201	Otop2	otopetrin 2	6.00
ENSMUSG00000071847	Apcdd1	adenomatosis polyposis coli down-regulated 1	5.99
ENSMUSG00000025938	Slco5a1	solute carrier organic anion transporter family, member 5A1	5.96
ENSMUSG00000078161	Erich3	glutamate rich 3	5.95
ENSMUSG00000025324	Atp10a	ATPase, class V, type 10A	5.94
ENSMUSG00000034684	Sema3f	sema domain, immunoglobulin domain (Ig), short basic domain, secreted, (semaphorin) 3F	5.93
ENSMUSG00000042804	Gpr153	G protein-coupled receptor 153	5.89
ENSMUSG00000027985	Lef1	lymphoid enhancer binding factor 1	5.88
ENSMUSG00000014329	Bicc1	BicC family RNA binding protein 1	5.88
ENSMUSG00000010751	Tnfrsf22	tumor necrosis factor receptor superfamily, member 22	5.86
ENSMUSG00000040998	Npnt	nephronectin	5.85
ENSMUSG00000038146	Notch3	notch 3	5.84
ENSMUSG00000028497	Hacd4	3-hydroxyacyl-CoA dehydratase 4	5.83
ENSMUSG00000110802	Gm47141	predicted gene, 47141	5.83
ENSMUSG00000020086	H2afy2	H2A histone family, member Y2	5.82
ENSMUSG00000085412	Halr1	Hoxa adjacent long noncoding RNA 1	5.81
ENSMUSG00000042514	Klhl14	kelch-like 14	5.79
ENSMUSG00000007097	Atp1a2	ATPase, Na+/K+ transporting, alpha 2 polypeptide	5.77
ENSMUSG00000013338	Fer1l4	fer-1-like 4 (C. elegans)	5.77
ENSMUSG00000022665	Ccdc80	coiled-coil domain containing 80	5.76
ENSMUSG00000023008	Fmnl3	formin-like 3	5.75
ENSMUSG00000005883	Spo11	SPO11 meiotic protein covalently bound to DSB	5.75
ENSMUSG00000078235	Fam43b	family with sequence similarity 43, member B	5.74
ENSMUSG00000027547	Sall4	spalt like transcription factor 4	5.70
ENSMUSG00000052516	Robo2	roundabout guidance receptor 2	5.69
ENSMUSG00000108145	Gm38811	predicted gene, 38811	5.69
ENSMUSG00000051596	Otop1	otopetrin 1	5.69
ENSMUSG00000026193	Fn1	fibronectin 1	5.68
ENSMUSG00000027210	Meis2	Meis homeobox 2	5.64
ENSMUSG00000017466	Timp2	tissue inhibitor of metalloproteinase 2	5.58
ENSMUSG00000026579	F5	coagulation factor V	5.58
ENSMUSG00000040289	Hey1	hairy/enhancer-of-split related with YRPW motif 1	5.58
ENSMUSG00000033508	Asprv1	aspartic peptidase, retroviral-like 1	5.58
ENSMUSG00000048191	Muc6	mucin 6, gastric	5.56
ENSMUSG00000041202	Pla2g2d	phospholipase A2, group IID	5.56
ENSMUSG00000113642	Gm49329	predicted gene, 49329	5.55
ENSMUSG00000097767	Miat	myocardial infarction associated transcript (non-protein coding)	5.52
ENSMUSG00000045667	Smtnl2	smoothelin-like 2	5.50
ENSMUSG00000026728	Vim	vimentin	5.45
ENSMUSG00000029757	Dync1i1	dynein cytoplasmic 1 intermediate chain 1	5.45
ENSMUSG00000025089	Gfra1	glial cell line derived neurotrophic factor family receptor alpha 1	5.45
ENSMUSG00000039239	Tgfb2	transforming growth factor, beta 2	5.44
ENSMUSG00000022548	Apod	apolipoprotein D	5.44
ENSMUSG00000053049	Gm15413	predicted gene 15413	5.42
ENSMUSG00000029868	Trpv6	transient receptor potential cation channel, subfamily V, member 6	5.41
ENSMUSG00000022456	Sept3	septin 3	5.38
ENSMUSG00000075033	Nxpe3	neurexophilin and PC-esterase domain family, member 3	5.37
ENSMUSG00000085903	Gm15340	predicted gene 15340	5.36
ENSMUSG00000028246	Faxc	failed axon connections homolog	5.35
ENSMUSG00000033227	Wnt6	wingless-type MMTV integration site family, member 6	5.35
ENSMUSG00000091055	Siglec15	sialic acid binding Ig-like lectin 15	5.34
ENSMUSG00000106073	Gm42892	predicted gene 42892	5.34
ENSMUSG00000027858	Tspan2	tetraspanin 2	5.33
ENSMUSG00000035678	Tnfsf9	tumor necrosis factor (ligand) superfamily, member 9	5.32
ENSMUSG00000027931	Npr1	natriuretic peptide receptor 1	5.32
ENSMUSG00000044562	Rasip1	Ras interacting protein 1	5.31
ENSMUSG00000041801	Phlda3	pleckstrin homology like domain, family A, member 3	5.30
ENSMUSG00000026620	Mark1	MAP/microtubule affinity regulating kinase 1	5.29
ENSMUSG00000052221	Ppp1r36	protein phosphatase 1, regulatory subunit 36	5.29
ENSMUSG00000040663	Clcf1	cardiotrophin-like cytokine factor 1	5.28
ENSMUSG00000045991	Onecut2	one cut domain, family member 2	5.26
ENSMUSG00000060548	Tnfrsf19	tumor necrosis factor receptor superfamily, member 19	5.25
ENSMUSG00000036412	Arsi	arylsulfatase i	5.25
ENSMUSG00000035164	Zc3h12c	zinc finger CCCH type containing 12C	5.23
ENSMUSG00000067818	Myl9	myosin, light polypeptide 9, regulatory	5.21
ENSMUSG00000032496	Ltf	lactotransferrin	5.21
ENSMUSG00000068923	Syt11	synaptotagmin XI	5.20
ENSMUSG00000063011	Msln	mesothelin	5.20
ENSMUSG00000014602	Kif1a	kinesin family member 1A	5.18
ENSMUSG00000024659	Anxa1	annexin A1	5.18
ENSMUSG00000022371	Col14a1	collagen, type XIV, alpha 1	5.18
ENSMUSG00000029417	Cxcl9	chemokine (C-X-C motif) ligand 9	5.18
ENSMUSG00000040415	Dtx3	deltex 3, E3 ubiquitin ligase	5.15
ENSMUSG00000046470	Sox18	SRY (sex determining region Y)-box 18	5.15
ENSMUSG00000031538	Plat	plasminogen activator, tissue	5.13
ENSMUSG00000050382	Kif7	kinesin family member 7	5.12

ENSMUSG00000074813	Gm14005	predicted gene 14005	5.12
ENSMUSG00000055333	Fat2	FAT atypical cadherin 2	5.11
ENSMUSG00000020431	Adcy1	adenylate cyclase 1	5.10
ENSMUSG00000018340	Anxa6	annexin A6	5.09
ENSMUSG00000081016	Olfr1397-ps1	olfactory receptor 1397, pseudogene 1	5.07
ENSMUSG00000000031	H19	H19, imprinted maternally expressed transcript	5.06
ENSMUSG00000031737	Irx5	Iroquois homeobox 5	5.06
ENSMUSG00000009097	Tbx1	T-box 1	5.05
ENSMUSG00000025666	Tmem47	transmembrane protein 47	5.05
ENSMUSG00000070337	Gpr179	G protein-coupled receptor 179	5.04
ENSMUSG00000036502	Tmem255a	transmembrane protein 255A	5.03
ENSMUSG00000022594	Lynx1	Ly6/neurotoxin 1	5.00
ENSMUSG00000006576	Slc4a3	solute carrier family 4 (anion exchanger), member 3	5.00
ENSMUSG00000020427	Igfbp3	insulin-like growth factor binding protein 3	4.99
ENSMUSG00000039323	Igfbp2	insulin-like growth factor binding protein 2	4.98
ENSMUSG00000100733	4932411K12Rik	RIKEN cDNA 4932411K12 gene	4.98
ENSMUSG00000081392	Gm11668	predicted gene 11668	4.97
ENSMUSG00000030844	Rgs10	regulator of G-protein signalling 10	4.97
ENSMUSG00000035062	Zc4h2	zinc finger, C4H2 domain containing	4.97
ENSMUSG00000031734	Irx3	Iroquois related homeobox 3	4.95
ENSMUSG00000050640	Tmem150c	transmembrane protein 150C	4.94
ENSMUSG00000040430	Pitpnc1	phosphatidylinositol transfer protein, cytoplasmic 1	4.91
ENSMUSG00000000552	Zfp385a	zinc finger protein 385A	4.91
ENSMUSG00000034675	Dbn1	drebrin 1	4.91
ENSMUSG00000038496	Slc19a3	solute carrier family 19, member 3	4.91
ENSMUSG00000034685	Fam171a2	family with sequence similarity 171, member A2	4.91
ENSMUSG00000030873	Scnn1b	sodium channel, nonvoltage-gated 1 beta	4.86
ENSMUSG00000029380	Cxcl1	chemokine (C-X-C motif) ligand 1	4.84
ENSMUSG00000068748	Ptprz1	protein tyrosine phosphatase, receptor type Z, polypeptide 1	4.84
ENSMUSG00000032875	Arhgef17	Rho guanine nucleotide exchange factor (GEF) 17	4.83
ENSMUSG00000043430	Psapl1	prosaposin-like 1	4.83
ENSMUSG00000032625	Thsd7a	thrombospondin, type I, domain containing 7A	4.82
ENSMUSG00000021950	Anxa8	annexin A8	4.82
ENSMUSG00000022435	Upk3a	uroplakin 3A	4.82
ENSMUSG00000035783	Acta2	actin, alpha 2, smooth muscle, aorta	4.82
ENSMUSG00000029359	Tesc	tescalcin	4.81
ENSMUSG00000058952	Cfi	complement component factor i	4.79
ENSMUSG00000017204	Gsdma	gasdermin A	4.79
ENSMUSG00000020937	Plcd3	phospholipase C, delta 3	4.79
ENSMUSG00000010175	Prox1	prospero homeobox 1	4.78
ENSMUSG00000000093	Tbx2	T-box 2	4.78
ENSMUSG00000030351	Tspan11	tetraspanin 11	4.78
ENSMUSG00000027485	Bpifb1	BPI fold containing family B, member 1	4.78
ENSMUSG00000038775	Vill	villin-like	4.77
ENSMUSG00000032076	Cadm1	cell adhesion molecule 1	4.76
ENSMUSG00000045062	Pcdhb7	protocadherin beta 7	4.76
ENSMUSG00000027570	Col9a3	collagen, type IX, alpha 3	4.75
ENSMUSG00000052105	Mtcl1	microtubule crosslinking factor 1	4.73
ENSMUSG00000006931	P3h4	prolyl 3-hydroxylase family member 4 (non-enzymatic)	4.73
ENSMUSG00000069170	Adgrv1	adhesion G protein-coupled receptor V1	4.73
ENSMUSG00000015647	Lama5	laminin, alpha 5	4.72
ENSMUSG00000020844	Nxn	nucleoredoxin	4.72
ENSMUSG00000046618	Olfml2a	olfactomedin-like 2A	4.72
ENSMUSG00000072437	Nanos1	nanos C2HC-type zinc finger 1	4.71
ENSMUSG00000038456	Dennd2a	DENN/MADD domain containing 2A	4.70
ENSMUSG00000062151	Unc13c	unc-13 homolog C	4.69
ENSMUSG00000020814	Mxra7	matrix-remodelling associated 7	4.68
ENSMUSG00000034295	Fhod3	formin homology 2 domain containing 3	4.68
ENSMUSG00000025492	Ifitm3	interferon induced transmembrane protein 3	4.67
ENSMUSG00000039607	Rbms3	RNA binding motif, single stranded interacting protein	4.67
ENSMUSG00000028626	Col9a2	collagen, type IX, alpha 2	4.67
ENSMUSG00000037188	Grhl3	grainyhead like transcription factor 3	4.66
ENSMUSG00000004044	Cavin1	caveolae associated 1	4.66
ENSMUSG00000106709	Gm30270	predicted gene, 30270	4.66
ENSMUSG00000032492	Pth1r	parathyroid hormone 1 receptor	4.64
ENSMUSG00000039959	Hip1	huntingtin interacting protein 1	4.63
ENSMUSG00000026065	Slc9a4	solute carrier family 9 (sodium/hydrogen exchanger), member 4	4.62
ENSMUSG00000038295	Atg9b	autophagy related 9B	4.61
ENSMUSG00000059401	Mamld1	mastermind-like domain containing 1	4.61
ENSMUSG00000048450	Msx1	msh homeobox 1	4.60
ENSMUSG00000050989	Selenon	selenoprotein N	4.60
ENSMUSG00000024164	C3	complement component 3	4.56
ENSMUSG00000044034	Npb	neuropeptide B	4.56
ENSMUSG00000030796	Tead2	TEA domain family member 2	4.55
ENSMUSG00000037813	D630003M21Rik	RIKEN cDNA D630003M21 gene	4.55
ENSMUSG00000021539	Lect2	leukocyte cell-derived chemotaxin 2	4.55

ENSMUSG00000021765	Fst	follistatin	4.55
ENSMUSG00000112871	Gm35404	predicted gene, 35404	4.55
ENSMUSG00000021763	BC067074	cDNA sequence BC067074	4.54
ENSMUSG00000050192	Eif5a2	eukaryotic translation initiation factor 5A2	4.53
ENSMUSG00000025328	Padi3	peptidyl arginine deiminase, type III	4.53
ENSMUSG00000031328	Flna	filamin, alpha	4.52
ENSMUSG00000027803	Wwtr1	WW domain containing transcription regulator 1	4.52
ENSMUSG00000073599	Ecscr	endothelial cell surface expressed chemotaxis and apoptosis regulator	4.52
ENSMUSG00000059336	Slc14a1	solute carrier family 14 (urea transporter), member 1	4.50
ENSMUSG00000038132	Rbm24	RNA binding motif protein 24	4.50
ENSMUSG00000070436	Serpinh1	serine (or cysteine) peptidase inhibitor, clade H, member 1	4.50
ENSMUSG00000060429	Sntb1	syntrophin, basic 1	4.49
ENSMUSG00000030246	Ldhb	lactate dehydrogenase B	4.49
ENSMUSG00000044033	Ccdc141	coiled-coil domain containing 141	4.48
ENSMUSG00000030048	Gkn3	gastrokine 3	4.48
ENSMUSG00000021986	Amer2	APC membrane recruitment 2	4.47
ENSMUSG00000028883	Sema3a	sema domain, immunoglobulin domain (Ig), short basic domain, secreted, (semaphorin) 3A	4.43
ENSMUSG00000004668	Abca13	ATP-binding cassette, sub-family A (ABC1), member 13	4.43
ENSMUSG00000067889	Sptbn2	spectrin beta, non-erythrocytic 2	4.42
ENSMUSG00000040152	Thbs1	thrombospondin 1	4.42
ENSMUSG00000026888	Grb14	growth factor receptor bound protein 14	4.42
ENSMUSG00000086484	Nron	non-protein coding RNA, repressor of NFAT	4.42
ENSMUSG00000103636	Gm38331	predicted gene, 38331	4.39
ENSMUSG00000038180	Spag4	sperm associated antigen 4	4.36
ENSMUSG00000008398	Elk3	ELK3, member of ETS oncogene family	4.34
ENSMUSG00000031355	Arhgap6	Rho GTPase activating protein 6	4.34
ENSMUSG00000072591	5930412G12Rik	RIKEN cDNA 5930412G12 gene	4.34
ENSMUSG00000026676	Ccdc3	coiled-coil domain containing 3	4.33
ENSMUSG00000024502	Jakmip2	janus kinase and microtubule interacting protein 2	4.33
ENSMUSG00000017897	Eya2	EYA transcriptional coactivator and phosphatase 2	4.32
ENSMUSG00000068327	Tlx2	T cell leukemia, homeobox 2	4.32
ENSMUSG00000013089	Etv5	ets variant 5	4.29
ENSMUSG00000033763	Mtss1l	metastasis suppressor 1-like	4.29
ENSMUSG00000028328	Tmod1	tropomodulin 1	4.29
ENSMUSG00000042759	Apobr	apolipoprotein B receptor	4.29
ENSMUSG00000019997	Ctgf	connective tissue growth factor	4.29
ENSMUSG00000020092	Pald1	phosphatase domain containing, paladin 1	4.27
ENSMUSG00000063605	Ccdc102a	coiled-coil domain containing 102A	4.26
ENSMUSG00000060962	Dmkn	dermokine	4.26
ENSMUSG00000098132	Rassf10	Ras association (RalGDS/AF-6) domain family (N-terminal) member 10	4.25
ENSMUSG00000009628	Tex15	testis expressed gene 15	4.25
ENSMUSG00000080316	Spaca6	sperm acrosome associated 6	4.25
ENSMUSG00000058070	Eml1	echinoderm microtubule associated protein like 1	4.22
ENSMUSG00000024940	Ltbp3	latent transforming growth factor beta binding protein 3	4.20
ENSMUSG00000020926	Adam11	a disintegrin and metallopeptidase domain 11	4.19
ENSMUSG00000025656	Arhgef9	CDC42 guanine nucleotide exchange factor (GEF) 9	4.18
ENSMUSG00000074796	Slc4a11	solute carrier family 4, sodium bicarbonate transporter-like, member 11	4.17
ENSMUSG00000039601	Rcan2	regulator of calcineurin 2	4.16
ENSMUSG00000053297	AI854703	expressed sequence AI854703	4.16
ENSMUSG00000027257	Pacsin3	protein kinase C and casein kinase substrate in neurons 3	4.14
ENSMUSG00000020900	Myh10	myosin, heavy polypeptide 10, non-muscle	4.13
ENSMUSG00000036944	Tmem71	transmembrane protein 71	4.13
ENSMUSG00000075304	Sp5	trans-acting transcription factor 5	4.12
ENSMUSG00000050212	Eva1b	eva-1 homolog B (C. elegans)	4.12
ENSMUSG00000022871	Fetub	fetuin beta	4.12
ENSMUSG00000104612	Gm42449	predicted gene 42449	4.07
ENSMUSG00000004791	Pgf	placental growth factor	4.07
ENSMUSG00000025140	Pycr1	pyrroline-5-carboxylate reductase 1	4.06
ENSMUSG00000078922	Tgtp1	T cell specific GTPase 1	4.06
ENSMUSG00000013076	Amotl1	angiomotin-like 1	4.05
ENSMUSG00000026950	Neb	nebulin	4.05
ENSMUSG00000031502	Col4a1	collagen, type IV, alpha 1	4.05
ENSMUSG00000025855	Prkar1b	protein kinase, cAMP dependent regulatory, type I beta	4.05
ENSMUSG00000044548	Dact1	dishevelled-binding antagonist of beta-catenin 1	4.04
ENSMUSG00000022996	Wnt10b	wingless-type MMTV integration site family, member 10B	4.03
ENSMUSG00000002980	Bcam	basal cell adhesion molecule	4.02
ENSMUSG00000039153	Runx2	runt related transcription factor 2	4.01
ENSMUSG00000032698	Lmo2	LIM domain only 2	4.01
ENSMUSG00000041608	Entpd3	ectonucleoside triphosphate diphosphohydrolase 3	4.00
ENSMUSG00000009687	Fxyd5	FXYD domain-containing ion transport regulator 5	3.99
ENSMUSG00000031760	Mt3	metallothionein 3	3.99
ENSMUSG00000025902	Sox17	SRY (sex determining region Y)-box 17	3.98
ENSMUSG00000021256	Vash1	vasohibin 1	3.98
ENSMUSG00000032452	Clstn2	calsyntenin 2	3.97
ENSMUSG00000055980	Irs1	insulin receptor substrate 1	3.97
ENSMUSG00000044022	Pcdhb21	protocadherin beta 21	3.97

ENSMUSG00000062661	Ncs1	neuronal calcium sensor 1	3.96
ENSMUSG00000000320	Alox12	arachidonate 12-lipoxygenase	3.96
ENSMUSG00000087179	Gm14230	predicted gene 14230	3.96
ENSMUSG00000079363	Gbp4	guanylate binding protein 4	3.95
ENSMUSG00000055413	H2-Q5	histocompatibility 2, Q region locus 5	3.95
ENSMUSG00000043068	Fam89a	family with sequence similarity 89, member A	3.93
ENSMUSG00000103749	Pcdhgb5	protocadherin gamma subfamily B, 5	3.92
ENSMUSG00000037095	Lrg1	leucine-rich alpha-2-glycoprotein 1	3.90
ENSMUSG00000029287	Tgfbr3	transforming growth factor, beta receptor III	3.90
ENSMUSG00000049556	Lingo1	leucine rich repeat and Ig domain containing 1	3.90
ENSMUSG00000019817	Plagl1	pleiomorphic adenoma gene-like 1	3.89
ENSMUSG00000113211	4921525O09Rik	RIKEN cDNA 4921525O09 gene	3.89
ENSMUSG00000040187	Arntl2	aryl hydrocarbon receptor nuclear translocator-like 2	3.87
ENSMUSG00000105096	Gbp10	guanylate-binding protein 10	3.87
ENSMUSG00000092586	Ly6g6c	lymphocyte antigen 6 complex, locus G6C	3.86
ENSMUSG00000035640	Cbarp	calcium channel, voltage-dependent, beta subunit associated regulatory protein	3.85
ENSMUSG00000090399	Gm38399	predicted gene, 38399	3.85
ENSMUSG00000029298	Gbp9	guanylate-binding protein 9	3.85
ENSMUSG00000021493	Pdlim7	PDZ and LIM domain 7	3.84
ENSMUSG00000000782	Tcf7	transcription factor 7, T cell specific	3.83
ENSMUSG00000074766	Ism1	isthmin 1, angiogenesis inhibitor	3.83
ENSMUSG00000041189	Chrnb1	cholinergic receptor, nicotinic, beta polypeptide 1 (muscle)	3.81
ENSMUSG00000038578	Susd1	sushi domain containing 1	3.81
ENSMUSG00000046058	Eid2	EP300 interacting inhibitor of differentiation 2	3.81
ENSMUSG00000074916	Chst14	carbohydrate (N-acetylgalactosamine 4-0) sulfotransferase 14	3.81
ENSMUSG00000023828	Slc22a3	solute carrier family 22 (organic cation transporter), member 3	3.80
ENSMUSG00000023009	Nckap5l	NCK-associated protein 5-like	3.80
ENSMUSG00000107796	Gm44068	predicted gene, 44068	3.80
ENSMUSG00000030223	Ptpro	protein tyrosine phosphatase, receptor type, O	3.79
ENSMUSG00000047363	Cstad	CSA-conditional, T cell activation-dependent protein	3.79
ENSMUSG00000061517	Sox21	SRY (sex determining region Y)-box 21	3.78
ENSMUSG00000045288	Ush1g	USH1 protein network component sans	3.77
ENSMUSG00000115602	Gm7908	predicted gene 7908	3.77
ENSMUSG00000020646	Mboat2	membrane bound O-acyltransferase domain containing 2	3.76
ENSMUSG00000047910	Pcdhb16	protocadherin beta 16	3.76
ENSMUSG00000034156	Tspoap1	TSPO associated protein 1	3.76
ENSMUSG00000041324	Inhba	inhibin beta-A	3.76
ENSMUSG00000112084	Gm10773	predicted gene 10773	3.76
ENSMUSG00000055945	Prr18	proline rich 18	3.75
ENSMUSG00000036062	Phf24	PHD finger protein 24	3.75
ENSMUSG00000015957	Wnt11	wingless-type MMTV integration site family, member 11	3.75
ENSMUSG00000048058	Ldlrad3	low density lipoprotein receptor class A domain containing 3	3.74
ENSMUSG00000055805	Fmnl1	formin-like 1	3.74
ENSMUSG00000021306	Gpr137b	G protein-coupled receptor 137B	3.74
ENSMUSG00000029832	Nfe2l3	nuclear factor, erythroid derived 2, like 3	3.72
ENSMUSG00000001521	Tulp3	tubby-like protein 3	3.71
ENSMUSG00000054793	Cadm4	cell adhesion molecule 4	3.70
ENSMUSG00000103189	Gm37092	predicted gene, 37092	3.70
ENSMUSG00000030352	Tspan9	tetraspanin 9	3.69
ENSMUSG00000059668	Krt4	keratin 4	3.68
ENSMUSG00000029361	Nos1	nitric oxide synthase 1, neuronal	3.68
ENSMUSG00000046818	Ddit4l	DNA-damage-inducible transcript 4-like	3.68
ENSMUSG00000049907	Rasl11b	RAS-like, family 11, member B	3.67
ENSMUSG00000003541	Ier3	immediate early response 3	3.65
ENSMUSG00000028199	Cryz	crystallin, zeta	3.65
ENSMUSG00000036500	Akp3	alkaline phosphatase 3, intestine, not Mn requiring	3.64
ENSMUSG00000046768	Rhoj	ras homolog family member J	3.64
ENSMUSG00000034612	Chst11	carbohydrate sulfotransferase 11	3.63
ENSMUSG00000048644	Ctxn1	cortexin 1	3.63
ENSMUSG00000078921	Tgtp2	T cell specific GTPase 2	3.62
ENSMUSG00000085111	Ascl4	achaete-scute family bHLH transcription factor 4	3.62
ENSMUSG00000043924	Ncmap	noncompact myelin associated protein	3.62
ENSMUSG00000013419	Zfp651	zinc finger protein 651	3.62
ENSMUSG00000111293	Gm34006	predicted gene, 34006	3.62
ENSMUSG00000008932	Slc1a7	solute carrier family 1 (glutamate transporter), member 7	3.62
ENSMUSG00000045136	Tubb2b	tubulin, beta 2B class IIB	3.61
ENSMUSG00000090394	4930523C07Rik	RIKEN cDNA 4930523C07 gene	3.61
ENSMUSG00000054720	Lrrc8c	leucine rich repeat containing 8 family, member C	3.61
ENSMUSG00000020651	Slc26a4	solute carrier family 26, member 4	3.60
ENSMUSG00000026981	Il1rn	interleukin 1 receptor antagonist	3.58
ENSMUSG00000022816	Fstl1	follistatin-like 1	3.56
ENSMUSG00000019990	Pde7b	phosphodiesterase 7B	3.56
ENSMUSG00000108897	Gm44861	predicted gene 44861	3.56
ENSMUSG00000027238	Frmd5	FERM domain containing 5	3.55
ENSMUSG00000037071	Scd1	stearoyl-Coenzyme A desaturase 1	3.54
ENSMUSG00000037379	Spon2	spondin 2, extracellular matrix protein	3.54

ENSMUSG00000001473	Tubb6	tubulin, beta 6 class V	3.53
ENSMUSG00000001288	Rarg	retinoic acid receptor, gamma	3.53
ENSMUSG00000029674	Limk1	LIM-domain containing, protein kinase	3.53
ENSMUSG00000008734	Gprc5b	G protein-coupled receptor, family C, group 5, member B	3.53
ENSMUSG00000035246	Pcyt1b	phosphate cytidylyltransferase 1, choline, beta isoform	3.52
ENSMUSG00000048776	Pthlh	parathyroid hormone-like peptide	3.52
ENSMUSG00000024401	Tnf	tumor necrosis factor	3.51
ENSMUSG00000055407	Map6	microtubule-associated protein 6	3.50
ENSMUSG00000022893	Adamts1	a disintegrin-like and metallopeptidase (reprolysin type) with thrombospondin type 1 motif, 1	3.50
ENSMUSG00000006411	Nectin4	nectin cell adhesion molecule 4	3.48
ENSMUSG00000057193	Slc44a2	solute carrier family 44, member 2	3.47
ENSMUSG00000063455	D630045J12Rik	RIKEN cDNA D630045J12 gene	3.47
ENSMUSG00000025582	Nptx1	neuronal pentraxin 1	3.47
ENSMUSG00000028600	Podn	podocan	3.46
ENSMUSG00000016526	Dyrk3	dual-specificity tyrosine-(Y)-phosphorylation regulated kinase 3	3.46
ENSMUSG00000029761	Cald1	caldesmon 1	3.45
ENSMUSG00000020325	Fstl3	follistatin-like 3	3.45
ENSMUSG00000014592	Camta1	calmodulin binding transcription activator 1	3.43
ENSMUSG00000019235	Rps6kl1	ribosomal protein S6 kinase-like 1	3.43
ENSMUSG00000081683	Fzd10	frizzled class receptor 10	3.43
ENSMUSG00000055653	Gpc3	glypican 3	3.43
ENSMUSG00000028108	Ecm1	extracellular matrix protein 1	3.42
ENSMUSG00000039004	Bmp6	bone morphogenetic protein 6	3.41
ENSMUSG00000074384	AI429214	expressed sequence AI429214	3.41
ENSMUSG00000030510	Cers3	ceramide synthase 3	3.41
ENSMUSG00000032387	Rbpms2	RNA binding protein with multiple splicing 2	3.41
ENSMUSG00000023905	Tnfrsf12a	tumor necrosis factor receptor superfamily, member 12a	3.40
ENSMUSG00000059991	Nptx2	neuronal pentraxin 2	3.40
ENSMUSG00000025608	Podxl	podocalyxin-like	3.39
ENSMUSG00000058145	Adamts17	a disintegrin-like and metallopeptidase (reprolysin type) with thrombospondin type 1 motif, 17	3.38
ENSMUSG00000030409	Dmpk	dystrophia myotonica-protein kinase	3.38
ENSMUSG00000058908	Pla2g2a	phospholipase A2, group IIA (platelets, synovial fluid)	3.37
ENSMUSG00000036158	Prickle1	prickle planar cell polarity protein 1	3.37
ENSMUSG00000024349	Tmem173	transmembrane protein 173	3.36
ENSMUSG00000043439	Epop	elongin BC and polycomb repressive complex 2 associated protein	3.36
ENSMUSG00000037681	Esyt3	extended synaptotagmin-like protein 3	3.35
ENSMUSG00000053646	Plxnb1	plexin B1	3.34
ENSMUSG00000052911	Lamb2	laminin, beta 2	3.34
ENSMUSG00000035954	Dock4	dedicator of cytokinesis 4	3.34
ENSMUSG00000071723	Gspt2	G1 to S phase transition 2	3.34
ENSMUSG00000034595	Ppp1r18	protein phosphatase 1, regulatory subunit 18	3.34
ENSMUSG00000057895	Zfp105	zinc finger protein 105	3.34
ENSMUSG00000006403	Adamts4	a disintegrin-like and metallopeptidase (reprolysin type) with thrombospondin type 1 motif, 4	3.34
ENSMUSG00000032068	Plet1	placenta expressed transcript 1	3.32
ENSMUSG00000026556	Vangl2	VANGL planar cell polarity 2	3.31
ENSMUSG00000070304	Scn2b	sodium channel, voltage-gated, type II, beta	3.31
ENSMUSG00000030600	Lrfn1	leucine rich repeat and fibronectin type III domain containing 1	3.31
ENSMUSG00000019853	Hebp2	heme binding protein 2	3.30
ENSMUSG00000044350	Lacc1	laccase domain containing 1	3.30
ENSMUSG00000037405	Icam1	intercellular adhesion molecule 1	3.30
ENSMUSG00000014303	Glis2	GLIS family zinc finger 2	3.30
ENSMUSG00000032940	Rbm11	RNA binding motif protein 11	3.28
ENSMUSG00000045545	Krt14	keratin 14	3.28
ENSMUSG00000001435	Col18a1	collagen, type XVIII, alpha 1	3.27
ENSMUSG00000043391	2510009E07Rik	RIKEN cDNA 2510009E07 gene	3.26
ENSMUSG00000030259	Rassf8	Ras association (RalGDS/AF-6) domain family (N-terminal) member 8	3.25
ENSMUSG00000046550	Spin2c	spindlin family, member 2C	3.25
ENSMUSG00000017493	Igfbp4	insulin-like growth factor binding protein 4	3.24
ENSMUSG00000035357	Pdzrn3	PDZ domain containing RING finger 3	3.24
ENSMUSG00000073164	2410018L13Rik	RIKEN cDNA 2410018L13 gene	3.24
ENSMUSG00000020044	Timp3	tissue inhibitor of metalloproteinase 3	3.23
ENSMUSG00000062488	Ifit3b	interferon-induced protein with tetratricopeptide repeats 3B	3.23
ENSMUSG00000022780	Meltf	melanotransferrin	3.22
ENSMUSG00000063919	Srrm4	serine/arginine repetitive matrix 4	3.22
ENSMUSG00000090386	Mir99ahg	Mir99a and Mirlet7c-1 host gene (non-protein coding)	3.22
ENSMUSG00000087589	D430040D24Rik	RIKEN cDNA D430040D24 gene	3.22
ENSMUSG00000062175	Tgif2	TGFB-induced factor homeobox 2	3.20
ENSMUSG00000002900	Lamb1	laminin B1	3.18
ENSMUSG00000040093	Bmf	BCL2 modifying factor	3.18
ENSMUSG00000055745	Rtl6	retrotransposon Gag like 6	3.18
ENSMUSG00000022178	Ajuba	ajuba LIM protein	3.17
ENSMUSG00000024109	Nrxn1	neurexin I	3.16
ENSMUSG00000019831	Wasf1	WAS protein family, member 1	3.15
ENSMUSG00000056947	Mab21l1	mab-21-like 1 (C. elegans)	3.15
ENSMUSG00000041592	Sdk2	sidekick cell adhesion molecule 2	3.15
ENSMUSG00000030605	Mfge8	milk fat globule-EGF factor 8 protein	3.14

ENSMUSG00000051343	Rab11fip5	RAB11 family interacting protein 5 (class I)	3.14
ENSMUSG00000027978	Prss12	protease, serine 12 neurotrypsin (motopsin)	3.14
ENSMUSG00000015709	Arnt2	aryl hydrocarbon receptor nuclear translocator 2	3.13
ENSMUSG00000000202	Btbd17	BTB (POZ) domain containing 17	3.13
ENSMUSG00000033436	Armcx2	armadillo repeat containing, X-linked 2	3.13
ENSMUSG00000046402	Rbp1	retinol binding protein 1, cellular	3.12
ENSMUSG00000073409	H2-Q6	histocompatibility 2, Q region locus 6	3.12
ENSMUSG00000031441	Atp11a	ATPase, class VI, type 11A	3.11
ENSMUSG00000037736	Limch1	LIM and calponin homology domains 1	3.11
ENSMUSG00000046546	Fam43a	family with sequence similarity 43, member A	3.11
ENSMUSG00000004709	Cd244a	CD244 molecule A	3.11
ENSMUSG00000060181	Slc35e3	solute carrier family 35, member E3	3.11
ENSMUSG00000023972	Ptk7	PTK7 protein tyrosine kinase 7	3.10
ENSMUSG00000027375	Mal	myelin and lymphocyte protein, T cell differentiation protein	3.10
ENSMUSG00000044043	Pcdhb14	protocadherin beta 14	3.10
ENSMUSG00000037053	Azgp1	alpha-2-glycoprotein 1, zinc	3.10
ENSMUSG00000037664	Cdkn1c	cyclin-dependent kinase inhibitor 1C (P57)	3.09
ENSMUSG00000042523	Dnal1	dynein, axonemal, light chain 1	3.09
ENSMUSG00000038651	Sycp2l	synaptonemal complex protein 2-like	3.09
ENSMUSG00000048347	Pcdhb18	protocadherin beta 18	3.09
ENSMUSG00000089762	Ier5l	immediate early response 5-like	3.08
ENSMUSG00000050295	Foxc1	forkhead box C1	3.08
ENSMUSG00000026072	Il1r1	interleukin 1 receptor, type I	3.08
ENSMUSG00000036036	Zfp57	zinc finger protein 57	3.08
ENSMUSG00000041390	Mdfic	MyoD family inhibitor domain containing	3.07
ENSMUSG00000038738	Shank1	SH3 and multiple ankyrin repeat domains 1	3.07
ENSMUSG00000062078	Qk	quaking	3.07
ENSMUSG00000027015	Cybrd1	cytochrome b reductase 1	3.05
ENSMUSG00000029101	Rgs12	regulator of G-protein signaling 12	3.03
ENSMUSG00000072964	Bhlhb9	basic helix-loop-helix domain containing, class B9	3.03
ENSMUSG00000020728	Cep112	centrosomal protein 112	3.02
ENSMUSG00000046387	Pcdhb17	protocadherin beta 17	3.02
ENSMUSG00000104379	Gm37509	predicted gene, 37509	3.02
ENSMUSG00000013236	Ptprs	protein tyrosine phosphatase, receptor type, S	3.01
ENSMUSG00000050447	Lypd6	LY6/PLAUR domain containing 6	3.01
ENSMUSG00000053477	Tcf4	transcription factor 4	3.00
ENSMUSG00000020674	Pxdn	peroxidasin	3.00
ENSMUSG00000038980	Rbbp8nl	RBBP8 N-terminal like	3.00
ENSMUSG00000048285	Frmd6	FERM domain containing 6	3.00
ENSMUSG00000022790	Igsf11	immunoglobulin superfamily, member 11	3.00
ENSMUSG00000038463	Olfml2b	olfactomedin-like 2B	3.00
ENSMUSG00000022587	Ly6e	lymphocyte antigen 6 complex, locus E	2.99
ENSMUSG00000063623	C230062I16Rik	RIKEN cDNA C230062I16 gene	2.99
ENSMUSG00000064263	Platr26	pluripotency associated transcript 26	2.98
ENSMUSG00000032554	Trf	transferrin	2.98
ENSMUSG00000032232	Cgnl1	cingulin-like 1	2.96
ENSMUSG00000074480	Mex3a	mex3 RNA binding family member A	2.96
ENSMUSG00000033149	Phldb2	pleckstrin homology like domain, family B, member 2	2.96
ENSMUSG00000063851	Rnf183	ring finger protein 183	2.96
ENSMUSG00000021477	Ctsl	cathepsin L	2.95
ENSMUSG00000091243	Vgll3	vestigial like family member 3	2.95
ENSMUSG00000080937	Gm13510	predicted gene 13510	2.95
ENSMUSG00000007039	Ddah2	dimethylarginine dimethylaminohydrolase 2	2.94
ENSMUSG00000053080	2700081O15Rik	RIKEN cDNA 2700081O15 gene	2.93
ENSMUSG00000041570	Camsap2	calmodulin regulated spectrin-associated protein family, member 2	2.92
ENSMUSG00000107076	Gm43480	predicted gene 43480	2.92
ENSMUSG00000028698	Pik3r3	phosphoinositide-3-kinase regulatory subunit 3	2.91
ENSMUSG00000108833	Gm17909	predicted gene, 17909	2.91
ENSMUSG00000031849	Comp	cartilage oligomeric matrix protein	2.91
ENSMUSG00000066705	Fxyd6	FXYD domain-containing ion transport regulator 6	2.91
ENSMUSG00000016382	Pls3	plastin 3 (T-isoform)	2.90
ENSMUSG00000040690	Col16a1	collagen, type XVI, alpha 1	2.90
ENSMUSG00000019846	Lama4	laminin, alpha 4	2.89
ENSMUSG00000026475	Rgs16	regulator of G-protein signaling 16	2.88
ENSMUSG00000017314	Mpp2	membrane protein, palmitoylated 2 (MAGUK p55 subfamily member 2)	2.88
ENSMUSG00000033276	Stk36	serine/threonine kinase 36	2.88
ENSMUSG00000007207	Stx1a	syntaxin 1A (brain)	2.86
ENSMUSG00000026970	Rbms1	RNA binding motif, single stranded interacting protein 1	2.86
ENSMUSG00000079669	Gm17396	predicted gene, 17396	2.86
ENSMUSG00000026121	Sema4c	sema domain, immunoglobulin domain (Ig), transmembrane domain (TM) and short cytoplasmic do	2.84
ENSMUSG00000039693	Msantd3	Myb/SANT-like DNA-binding domain containing 3	2.84
ENSMUSG00000089715	Cbx6	chromobox 6	2.83
ENSMUSG00000056214	Pard6g	par-6 family cell polarity regulator gamma	2.83
ENSMUSG00000055633	Zfp580	zinc finger protein 580	2.83
ENSMUSG00000085440	Sorbs2os	sorbin and SH3 domain containing 2, opposite strand	2.83
ENSMUSG00000036123	Slc9a3	solute carrier family 9 (sodium/hydrogen exchanger), member 3	2.82

ENSMUSG00000010021	Kif19a	kinesin family member 19A	2.82
ENSMUSG00000026509	Capn2	calpain 2	2.81
ENSMUSG00000044250	Pced1b	PC-esterase domain containing 1B	2.81
ENSMUSG00000022864	D16Ertd472e	DNA segment, Chr 16, ERATO Doi 472, expressed	2.81
ENSMUSG00000026185	Igfbp5	insulin-like growth factor binding protein 5	2.81
ENSMUSG00000028780	Sema3c	sema domain, immunoglobulin domain (Ig), short basic domain, secreted, (semaphorin) 3C	2.80
ENSMUSG00000036053	Fmnl2	formin-like 2	2.80
ENSMUSG00000028347	Tmeff1	transmembrane protein with EGF-like and two follistatin-like domains 1	2.80
ENSMUSG00000017667	Zfp334	zinc finger protein 334	2.80
ENSMUSG00000097857	Gm26603	predicted gene, 26603	2.80
ENSMUSG00000052921	Arhgef15	Rho guanine nucleotide exchange factor (GEF) 15	2.80
ENSMUSG00000041961	Znrf3	zinc and ring finger 3	2.79
ENSMUSG00000031586	Rbpms	RNA binding protein gene with multiple splicing	2.79
ENSMUSG00000023912	Slc25a27	solute carrier family 25, member 27	2.79
ENSMUSG00000030630	Fah	fumarylacetoacetate hydrolase	2.79
ENSMUSG00000040714	Klc3	kinesin light chain 3	2.78
ENSMUSG00000084087	Gm13650	predicted gene 13650	2.78
ENSMUSG00000013275	Slc41a1	solute carrier family 41, member 1	2.77
ENSMUSG00000043639	Rbm20	RNA binding motif protein 20	2.77
ENSMUSG00000009614	Sardh	sarcosine dehydrogenase	2.77
ENSMUSG00000034774	Dsg1c	desmoglein 1 gamma	2.77
ENSMUSG00000026365	Cfh	complement component factor h	2.76
ENSMUSG00000026494	Kif26b	kinesin family member 26B	2.75
ENSMUSG00000035901	Dennd5a	DENN/MADD domain containing 5A	2.75
ENSMUSG00000051790	Nlgn2	neuroligin 2	2.75
ENSMUSG00000030513	Pcsk6	proprotein convertase subtilisin/kexin type 6	2.75
ENSMUSG00000024901	Peli3	pellino 3	2.75
ENSMUSG00000003665	Has1	hyaluronan synthase 1	2.75
ENSMUSG00000046449	Nexmif	neurite extension and migration factor	2.74
ENSMUSG00000028357	Kif12	kinesin family member 12	2.73
ENSMUSG00000049871	Nlrc3	NLR family, CARD domain containing 3	2.73
ENSMUSG00000027381	Bcl2l11	BCL2-like 11 (apoptosis facilitator)	2.72
ENSMUSG00000050910	Cdr2l	cerebellar degeneration-related protein 2-like	2.72
ENSMUSG00000073433	Arhgdig	Rho GDP dissociation inhibitor (GDI) gamma	2.72
ENSMUSG00000043313	Pcdhb19	protocadherin beta 19	2.72
ENSMUSG00000020205	Phlda1	pleckstrin homology like domain, family A, member 1	2.71
ENSMUSG00000037972	Snn	stannin	2.71
ENSMUSG00000028358	Zfp618	zinc finger protein 618	2.70
ENSMUSG00000019843	Fyn	Fyn proto-oncogene	2.70
ENSMUSG00000060166	Zdhhc8	zinc finger, DHHC domain containing 8	2.70
ENSMUSG00000003573	Homer3	homer scaffolding protein 3	2.70
ENSMUSG00000060314	Zfp941	zinc finger protein 941	2.69
ENSMUSG00000105681	Gm43428	predicted gene 43428	2.69
ENSMUSG00000112124	Gm19056	predicted gene, 19056	2.69
ENSMUSG00000060550	H2-Q7	histocompatibility 2, Q region locus 7	2.68
ENSMUSG00000056665	Them6	thioesterase superfamily member 6	2.68
ENSMUSG00000092569	Gm20544	predicted gene 20544	2.68
ENSMUSG00000020804	Aanat	arylalkylamine N-acetyltransferase	2.68
ENSMUSG00000000957	Mmp14	matrix metallopeptidase 14 (membrane-inserted)	2.67
ENSMUSG00000042306	S100a14	S100 calcium binding protein A14	2.67
ENSMUSG00000097494	4933406C10Rik	RIKEN cDNA 4933406C10 gene	2.67
ENSMUSG00000015243	Abca1	ATP-binding cassette, sub-family A (ABC1), member 1	2.66
ENSMUSG00000008090	Fgfrl1	fibroblast growth factor receptor-like 1	2.66
ENSMUSG00000020773	Trim47	tripartite motif-containing 47	2.66
ENSMUSG00000041889	Shisa4	shisa family member 4	2.66
ENSMUSG00000026211	Obsl1	obscurin-like 1	2.65
ENSMUSG00000040990	Sh3kbp1	SH3-domain kinase binding protein 1	2.64
ENSMUSG00000018008	Cyth4	cytohesin 4	2.64
ENSMUSG00000035274	Tpbg	trophoblast glycoprotein	2.63
ENSMUSG00000024736	Tmem132a	transmembrane protein 132A	2.63
ENSMUSG00000038079	Tmem237	transmembrane protein 237	2.63
ENSMUSG00000030222	Rerg	RAS-like, estrogen-regulated, growth-inhibitor	2.63
ENSMUSG00000037936	Scarb1	scavenger receptor class B, member 1	2.62
ENSMUSG00000049823	Zbtb12	zinc finger and BTB domain containing 12	2.61
ENSMUSG00000086395	A630014C17Rik	RIKEN cDNA A630014C17 gene	2.61
ENSMUSG00000026473	Glul	glutamate-ammonia ligase (glutamine synthetase)	2.60
ENSMUSG00000046731	Kctd11	potassium channel tetramerisation domain containing 11	2.60
ENSMUSG00000033287	Kctd17	potassium channel tetramerisation domain containing 17	2.60
ENSMUSG00000022421	Nptxr	neuronal pentraxin receptor	2.60
ENSMUSG00000002633	Shh	sonic hedgehog	2.60
ENSMUSG00000031351	Zfp185	zinc finger protein 185	2.60
ENSMUSG00000002847	Pla1a	phospholipase A1 member A	2.60
ENSMUSG00000037902	Sirpa	signal-regulatory protein alpha	2.59
ENSMUSG00000024427	Spry4	sprouty RTK signaling antagonist 4	2.59
ENSMUSG00000073791	Efcab7	EF-hand calcium binding domain 7	2.59
ENSMUSG00000031530	Dusp4	dual specificity phosphatase 4	2.58

ENSMUSG00000036764	Dnajc12	DnaJ heat shock protein family (Hsp40) member C12	2.58
ENSMUSG00000106219	5830416I19Rik	RIKEN cDNA 5830416I19 gene	2.58
ENSMUSG00000097993	Ptprv	protein tyrosine phosphatase, receptor type, V	2.58
ENSMUSG00000021340	Gpld1	glycosylphosphatidylinositol specific phospholipase D1	2.57
ENSMUSG00000009378	Slc16a12	solute carrier family 16 (monocarboxylic acid transporters), member 12	2.57
ENSMUSG00000071042	Rasgrp3	RAS, guanyl releasing protein 3	2.57
ENSMUSG00000104415	Gm37069	predicted gene, 37069	2.57
ENSMUSG00000028766	Alpl	alkaline phosphatase, liver/bone/kidney	2.57
ENSMUSG00000042156	Dzip1	DAZ interacting protein 1	2.57
ENSMUSG00000032849	Abcc4	ATP-binding cassette, sub-family C (CFTR/MRP), member 4	2.56
ENSMUSG00000041930	Fam222a	family with sequence similarity 222, member A	2.55
ENSMUSG00000026411	Tmem9	transmembrane protein 9	2.55
ENSMUSG00000024912	Fosl1	fos-like antigen 1	2.55
ENSMUSG00000031925	Maml2	mastermind like transcriptional coactivator 2	2.55
ENSMUSG00000029771	Irf5	interferon regulatory factor 5	2.55
ENSMUSG00000049281	Scn3b	sodium channel, voltage-gated, type III, beta	2.55
ENSMUSG00000029094	Afap1	actin filament associated protein 1	2.54
ENSMUSG00000035914	Cd276	CD276 antigen	2.54
ENSMUSG00000032013	Trim29	tripartite motif-containing 29	2.54
ENSMUSG00000033730	Egr3	early growth response 3	2.53
ENSMUSG00000037341	Slc9a7	solute carrier family 9 (sodium/hydrogen exchanger), member 7	2.53
ENSMUSG00000016534	Lamp2	lysosomal-associated membrane protein 2	2.52
ENSMUSG00000048232	Fbxo10	F-box protein 10	2.52
ENSMUSG00000102460	Gm38197	predicted gene, 38197	2.52
ENSMUSG00000028152	Tspan5	tetraspanin 5	2.51
ENSMUSG00000053137	Mapk11	mitogen-activated protein kinase 11	2.51
ENSMUSG00000026405	C4bp	complement component 4 binding protein	2.50
ENSMUSG00000040118	Cacna2d1	calcium channel, voltage-dependent, alpha2/delta subunit 1	2.50
ENSMUSG00000055485	Soga1	suppressor of glucose, autophagy associated 1	2.49
ENSMUSG00000031292	Cdkl5	cyclin-dependent kinase-like 5	2.49
ENSMUSG00000060671	Atp8b2	ATPase, class I, type 8B, member 2	2.49
ENSMUSG00000002603	Tgfb1	transforming growth factor, beta 1	2.49
ENSMUSG00000022199	Slc22a17	solute carrier family 22 (organic cation transporter), member 17	2.49
ENSMUSG00000031239	Itm2a	integral membrane protein 2A	2.49
ENSMUSG00000025921	Rdh10	retinol dehydrogenase 10 (all-trans)	2.48
ENSMUSG00000013033	Adgrl1	adhesion G protein-coupled receptor L1	2.48
ENSMUSG00000052852	Reep1	receptor accessory protein 1	2.48
ENSMUSG00000025592	Dach2	dachshund family transcription factor 2	2.48
ENSMUSG00000102278	Gm37145	predicted gene, 37145	2.48
ENSMUSG00000034872	Gipc3	GIPC PDZ domain containing family, member 3	2.48
ENSMUSG00000041245	Wnk3	WNK lysine deficient protein kinase 3	2.48
ENSMUSG00000056758	Hmga2	high mobility group AT-hook 2	2.47
ENSMUSG00000036902	Neto2	neuropilin (NRP) and tolloid (TLL)-like 2	2.47
ENSMUSG00000031398	Plxna3	plexin A3	2.47
ENSMUSG00000028464	Tpm2	tropomyosin 2, beta	2.47
ENSMUSG00000029093	Sorcs2	sortilin-related VPS10 domain containing receptor 2	2.46
ENSMUSG00000039478	Micu3	mitochondrial calcium uptake family, member 3	2.46
ENSMUSG00000025479	Cyp2e1	cytochrome P450, family 2, subfamily e, polypeptide 1	2.46
ENSMUSG00000071724	Smpd5	sphingomyelin phosphodiesterase 5	2.46
ENSMUSG00000006720	Zfp184	zinc finger protein 184 (Kruppel-like)	2.45
ENSMUSG00000031872	Bean1	brain expressed, associated with Nedd4, 1	2.45
ENSMUSG00000037716	Ccdc33	coiled-coil domain containing 33	2.44
ENSMUSG00000044716	Dok7	docking protein 7	2.43
ENSMUSG00000017723	Wfdc2	WAP four-disulfide core domain 2	2.43
ENSMUSG00000013921	Clip3	CAP-GLY domain containing linker protein 3	2.43
ENSMUSG00000027999	Pla2g12a	phospholipase A2, group XIIA	2.42
ENSMUSG00000044927	H1fx	H1 histone family, member X	2.42
ENSMUSG00000040808	S100g	S100 calcium binding protein G	2.42
ENSMUSG00000115275	9630050E16Rik	RIKEN cDNA 9630050E16 gene	2.42
ENSMUSG00000060591	Ifitm2	interferon induced transmembrane protein 2	2.41
ENSMUSG00000022505	Emp2	epithelial membrane protein 2	2.41
ENSMUSG00000041540	Sox5	SRY (sex determining region Y)-box 5	2.41
ENSMUSG00000068245	Phf11d	PHD finger protein 11D	2.41
ENSMUSG00000000486	Sept1	septin 1	2.41
ENSMUSG00000032470	Mras	muscle and microspikes RAS	2.40
ENSMUSG00000056486	Chn1	chimerin 1	2.40
ENSMUSG00000028100	Nudt17	nudix (nucleoside diphosphate linked moiety X)-type motif 17	2.39
ENSMUSG00000028836	Slc30a2	solute carrier family 30 (zinc transporter), member 2	2.38
ENSMUSG00000045932	Ifit2	interferon-induced protein with tetratricopeptide repeats 2	2.38
ENSMUSG00000031919	Tmed6	transmembrane p24 trafficking protein 6	2.38
ENSMUSG00000020599	Rgs9	regulator of G-protein signaling 9	2.37
ENSMUSG00000018012	Rac3	Rac family small GTPase 3	2.37
ENSMUSG00000020660	Pomc	pro-opiomelanocortin-alpha	2.37
ENSMUSG00000036667	Tcaf1	TRPM8 channel-associated factor 1	2.36
ENSMUSG00000018906	P4ha2	procollagen-proline, 2-oxoglutarate 4-dioxygenase (proline 4-hydroxylase), alpha II polypeptide	2.36
ENSMUSG00000029762	Akr1b8	aldo-keto reductase family 1, member B8	2.36

ENSMUSG00000034771	Tle2	transducin-like enhancer of split 2	2.36
ENSMUSG00000049191	Rtl5	retrotransposon Gag like 5	2.36
ENSMUSG00000037060	Cavin3	caveolae associated 3	2.36
ENSMUSG00000031207	Msn	moesin	2.35
ENSMUSG00000028545	Bend5	BEN domain containing 5	2.35
ENSMUSG00000022696	Sidt1	SID1 transmembrane family, member 1	2.34
ENSMUSG00000069255	Dusp22	dual specificity phosphatase 22	2.34
ENSMUSG00000113326	Gm47586	predicted gene, 47586	2.34
ENSMUSG00000030022	Adamts9	a disintegrin-like and metallopeptidase (reprolysin type) with thrombospondin type 1 motif, 9	2.34
ENSMUSG00000023913	Pla2g7	phospholipase A2, group VII (platelet-activating factor acetylhydrolase, plasma)	2.34
ENSMUSG00000045629	Sh3tc2	SH3 domain and tetratricopeptide repeats 2	2.34
ENSMUSG00000038296	Galnt18	polypeptide N-acetylgalactosaminyltransferase 18	2.34
ENSMUSG00000031633	Slc25a4	solute carrier family 25 (mitochondrial carrier, adenine nucleotide translocator), member 4	2.33
ENSMUSG00000048696	Mex3d	mex3 RNA binding family member D	2.33
ENSMUSG00000031112	Stk26	serine/threonine kinase 26	2.33
ENSMUSG00000021756	Il6st	interleukin 6 signal transducer	2.32
ENSMUSG00000053141	Ptprt	protein tyrosine phosphatase, receptor type, T	2.32
ENSMUSG00000084910	C630043F03Rik	RIKEN cDNA C630043F03 gene	2.32
ENSMUSG00000030789	Itgax	integrin alpha X	2.32
ENSMUSG00000024558	Mapk4	mitogen-activated protein kinase 4	2.31
ENSMUSG00000031596	Slc7a2	solute carrier family 7 (cationic amino acid transporter, y+ system), member 2	2.31
ENSMUSG00000110218	Gm20219	predicted gene, 20219	2.31
ENSMUSG00000039328	Rnf122	ring finger protein 122	2.30
ENSMUSG00000032607	Amt	aminomethyltransferase	2.30
ENSMUSG00000085421	4732490B19Rik	RIKEN cDNA 4732490B19 gene	2.30
ENSMUSG00000020620	Abca8b	ATP-binding cassette, sub-family A (ABC1), member 8b	2.30
ENSMUSG00000097709	2810429I04Rik	RIKEN cDNA 2810429I04 gene	2.30
ENSMUSG00000022433	Csnk1e	casein kinase 1, epsilon	2.29
ENSMUSG00000038545	Cul7	cullin 7	2.29
ENSMUSG00000040711	Sh3pxd2b	SH3 and PX domains 2B	2.29
ENSMUSG00000028838	Extl1	exostoses (multiple)-like 1	2.29
ENSMUSG00000104292	Gm38042	predicted gene, 38042	2.29
ENSMUSG00000069892	9930111J21Rik2	RIKEN cDNA 9930111J21 gene 2	2.29
ENSMUSG00000032289	Thsd4	thrombospondin, type I, domain containing 4	2.29
ENSMUSG00000039457	Ppl	periplakin	2.28
ENSMUSG00000056515	Rab31	RAB31, member RAS oncogene family	2.28
ENSMUSG00000034593	Myo5a	myosin VA	2.28
ENSMUSG00000039103	Nexn	nexilin	2.28
ENSMUSG00000002059	Rab34	RAB34, member RAS oncogene family	2.28
ENSMUSG00000044906	4930503L19Rik	RIKEN cDNA 4930503L19 gene	2.27
ENSMUSG00000046971	Pla2g4f	phospholipase A2, group IVF	2.27
ENSMUSG00000082361	Btc	betacellulin, epidermal growth factor family member	2.26
ENSMUSG00000045287	Rtn4rl1	reticulon 4 receptor-like 1	2.26
ENSMUSG00000038884	A230050P20Rik	RIKEN cDNA A230050P20 gene	2.26
ENSMUSG00000076431	Sox4	SRY (sex determining region Y)-box 4	2.25
ENSMUSG00000006777	Krt23	keratin 23	2.25
ENSMUSG00000047747	Rnf150	ring finger protein 150	2.25
ENSMUSG00000058317	Ube2e2	ubiquitin-conjugating enzyme E2E 2	2.25
ENSMUSG00000085329	2810404F17Rik	RIKEN cDNA 2810404F17 gene	2.25
ENSMUSG00000078762	Haus5	HAUS augmin-like complex, subunit 5	2.25
ENSMUSG00000113811	Gm47882	predicted gene, 47882	2.25
ENSMUSG00000026223	Itm2c	integral membrane protein 2C	2.24
ENSMUSG00000004032	Gstm5	glutathione S-transferase, mu 5	2.24
ENSMUSG00000037568	Vash2	vasohibin 2	2.24
ENSMUSG00000021835	Bmp4	bone morphogenetic protein 4	2.24
ENSMUSG00000056476	Med12l	mediator complex subunit 12-like	2.24
ENSMUSG00000020668	Kif3c	kinesin family member 3C	2.24
ENSMUSG00000104150	Gm44573	predicted gene 44573	2.24
ENSMUSG00000044468	Tent5c	terminal nucleotidyltransferase 5C	2.23
ENSMUSG00000038740	Mvb12b	multivesicular body subunit 12B	2.23
ENSMUSG00000058672	Tubb2a	tubulin, beta 2A class IIA	2.23
ENSMUSG00000025854	Fam20c	family with sequence similarity 20, member C	2.23
ENSMUSG00000111313	Gm18942	predicted gene, 18942	2.23
ENSMUSG00000009418	Nav1	neuron navigator 1	2.22
ENSMUSG00000033032	Afap1l1	actin filament associated protein 1-like 1	2.22
ENSMUSG00000001248	Gramd1a	GRAM domain containing 1A	2.22
ENSMUSG00000032640	Chsy1	chondroitin sulfate synthase 1	2.22
ENSMUSG00000028076	Cd1d1	CD1d1 antigen	2.22
ENSMUSG00000020396	Nefh	neurofilament, heavy polypeptide	2.22
ENSMUSG00000023031	Cela1	chymotrypsin-like elastase family, member 1	2.21
ENSMUSG00000055409	Nell1	NEL-like 1	2.21
ENSMUSG00000021506	Pitx1	paired-like homeodomain transcription factor 1	2.20
ENSMUSG00000023007	Prpf40b	pre-mRNA processing factor 40B	2.20
ENSMUSG00000028654	Mycl	v-myc avian myelocytomatosis viral oncogene lung carcinoma derived	2.19
ENSMUSG00000021464	Ror2	receptor tyrosine kinase-like orphan receptor 2	2.19
ENSMUSG00000038594	Cep85l	centrosomal protein 85-like	2.19

ENSMUSG00000029669	Tspan12	tetraspanin 12	2.18
ENSMUSG00000097715	Gpr137b-ps	G protein-coupled receptor 137B, pseudogene	2.18
ENSMUSG00000046191	Pcdhb20	protocadherin beta 20	2.18
ENSMUSG00000032514	Ttc21a	tetratricopeptide repeat domain 21A	2.18
ENSMUSG00000027346	Gpcpd1	glycerophosphocholine phosphodiesterase 1	2.17
ENSMUSG00000025017	Pik3ap1	phosphoinositide-3-kinase adaptor protein 1	2.17
ENSMUSG00000027712	Anxa5	annexin A5	2.17
ENSMUSG00000026944	Abca2	ATP-binding cassette, sub-family A (ABC1), member 2	2.16
ENSMUSG00000033233	Trim45	tripartite motif-containing 45	2.16
ENSMUSG00000050071	Bex1	brain expressed X-linked 1	2.15
ENSMUSG00000034848	Ttc21b	tetratricopeptide repeat domain 21B	2.15
ENSMUSG00000039934	Gsap	gamma-secretase activating protein	2.15
ENSMUSG00000037833	Sh2d4b	SH2 domain containing 4B	2.15
ENSMUSG00000060985	Tdrd5	tudor domain containing 5	2.15
ENSMUSG00000105140	Gm43127	predicted gene 43127	2.15
ENSMUSG00000049577	Zfpm1	zinc finger protein, multitype 1	2.14
ENSMUSG00000047501	Cldn4	claudin 4	2.14
ENSMUSG00000041308	Sntb2	syntrophin, basic 2	2.14
ENSMUSG00000031066	Usp11	ubiquitin specific peptidase 11	2.14
ENSMUSG00000109079	Gm44755	predicted gene 44755	2.14
ENSMUSG00000027186	Elf5	E74-like factor 5	2.14
ENSMUSG00000000142	Axin2	axin 2	2.13
ENSMUSG00000028613	Lrp8	low density lipoprotein receptor-related protein 8, apolipoprotein e receptor	2.13
ENSMUSG00000055926	Gm14137	predicted gene 14137	2.13
ENSMUSG00000111905	Gm48045	predicted gene, 48045	2.13
ENSMUSG00000034177	Rnf43	ring finger protein 43	2.12
ENSMUSG00000022415	Syngr1	synaptogyrin 1	2.12
ENSMUSG00000047146	Tet1	tet methylcytosine dioxygenase 1	2.12
ENSMUSG00000110411	Gm45457	predicted gene 45457	2.12
ENSMUSG00000062937	Mtap	methylthioadenosine phosphorylase	2.11
ENSMUSG00000017390	Aldoc	aldolase C, fructose-bisphosphate	2.11
ENSMUSG00000032363	Adamts7	a disintegrin-like and metallopeptidase (reprolysin type) with thrombospondin type 1 motif, 7	2.11
ENSMUSG00000090100	Ttbk2	tau tubulin kinase 2	2.10
ENSMUSG00000049670	Morn4	MORN repeat containing 4	2.10
ENSMUSG00000031907	Zfp90	zinc finger protein 90	2.10
ENSMUSG00000052212	Cd177	CD177 antigen	2.09
ENSMUSG00000024270	Slc39a6	solute carrier family 39 (metal ion transporter), member 6	2.09
ENSMUSG00000029312	Klhl8	kelch-like 8	2.08
ENSMUSG00000027674	Pex5l	peroxisomal biogenesis factor 5-like	2.08
ENSMUSG00000046589	Lrrc8e	leucine rich repeat containing 8 family, member E	2.08
ENSMUSG00000031996	Aplp2	amyloid beta (A4) precursor-like protein 2	2.07
ENSMUSG00000073987	Ggh	gamma-glutamyl hydrolase	2.07
ENSMUSG00000032184	Lysmd2	LysM, putative peptidoglycan-binding, domain containing 2	2.07
ENSMUSG00000037946	Fgd3	FYVE, RhoGEF and PH domain containing 3	2.07
ENSMUSG00000001025	S100a6	S100 calcium binding protein A6 (calcyclin)	2.06
ENSMUSG00000026478	Lamc1	laminin, gamma 1	2.06
ENSMUSG00000046329	Slc25a23	solute carrier family 25 (mitochondrial carrier; phosphate carrier), member 23	2.06
ENSMUSG00000076441	Ass1	argininosuccinate synthetase 1	2.06
ENSMUSG00000041293	Adgrf1	adhesion G protein-coupled receptor F1	2.06
ENSMUSG00000080775	Gm6368	predicted gene 6368	2.06
ENSMUSG00000025038	Efhc2	EF-hand domain (C-terminal) containing 2	2.06
ENSMUSG00000087792	Gm25968	predicted gene, 25968	2.06
ENSMUSG00000028184	Adgrl2	adhesion G protein-coupled receptor L2	2.05
ENSMUSG00000024896	Minpp1	multiple inositol polyphosphate histidine phosphatase 1	2.05
ENSMUSG00000089809	Rasgef1b	RasGEF domain family, member 1B	2.05
ENSMUSG00000071855	Ccdc112	coiled-coil domain containing 112	2.05
ENSMUSG00000109341	Gm30873	predicted gene, 30873	2.05
ENSMUSG00000042524	Sun2	Sad1 and UNC84 domain containing 2	2.04
ENSMUSG00000058656	Samd12	sterile alpha motif domain containing 12	2.04
ENSMUSG00000056832	Ttc26	tetratricopeptide repeat domain 26	2.04
ENSMUSG00000014158	Trpv4	transient receptor potential cation channel, subfamily V, member 4	2.04
ENSMUSG00000044229	Nxpe4	neurexophilin and PC-esterase domain family, member 4	2.04
ENSMUSG00000062184	Hs6st2	heparan sulfate 6-O-sulfotransferase 2	2.04
ENSMUSG00000060261	Gtf2i	general transcription factor II I	2.03
ENSMUSG00000057092	Fxyd3	FXYD domain-containing ion transport regulator 3	2.03
ENSMUSG00000059895	Ptp4a3	protein tyrosine phosphatase 4a3	2.03
ENSMUSG00000027698	Nceh1	neutral cholesterol ester hydrolase 1	2.02
ENSMUSG00000021589	Rhobtb3	Rho-related BTB domain containing 3	2.02
ENSMUSG00000001249	Hpn	hepsin	2.02
ENSMUSG00000028348	Cavin4	caveolae associated 4	2.02
ENSMUSG00000059741	Myl3	myosin, light polypeptide 3	2.02
ENSMUSG00000053398	Phgdh	3-phosphoglycerate dehydrogenase	2.01
ENSMUSG00000037287	Tbcel	tubulin folding cofactor E-like	2.00
ENSMUSG00000041025	Iffo2	intermediate filament family orphan 2	2.00
ENSMUSG00000026604	Ptpn14	protein tyrosine phosphatase, non-receptor type 14	2.00
ENSMUSG00000050359	Sprr1a	small proline-rich protein 1A	2.00

ENSMUSG00000110256	Gm45412	predicted gene 45412	2.00
ENSMUSG00000027907	S100a11	S100 calcium binding protein A11	1.99
ENSMUSG00000009739	Pou6f1	POU domain, class 6, transcription factor 1	1.99
ENSMUSG00000020546	Stxbp4	syntaxin binding protein 4	1.99
ENSMUSG00000030862	Cpxm2	carboxypeptidase X 2 (M14 family)	1.99
ENSMUSG00000007989	Fzd3	frizzled class receptor 3	1.98
ENSMUSG00000038156	Spon1	spondin 1, (f-spondin) extracellular matrix protein	1.98
ENSMUSG00000024990	Rbp4	retinol binding protein 4, plasma	1.98
ENSMUSG00000042684	Npl	N-acetylneuraminate pyruvate lyase	1.98
ENSMUSG00000051510	Mafg	v-maf musculoaponeurotic fibrosarcoma oncogene family, protein G (avian)	1.97
ENSMUSG00000084939	Gm830	predicted gene 830	1.97
ENSMUSG00000062044	Lmtk3	lemur tyrosine kinase 3	1.97
ENSMUSG00000113876	Gm48042	predicted gene, 48042	1.97
ENSMUSG00000116656	AC131339.2	novel transcript	1.97
ENSMUSG00000107388	Gm42788	predicted gene 42788	1.97
ENSMUSG00000027333	Smox	spermine oxidase	1.96
ENSMUSG00000056648	Hoxb8	homeobox B8	1.96
ENSMUSG00000021611	Tert	telomerase reverse transcriptase	1.96
ENSMUSG00000024968	Rcor2	REST corepressor 2	1.96
ENSMUSG00000038065	Mturn	maturin, neural progenitor differentiation regulator homolog (Xenopus)	1.96
ENSMUSG00000051652	Lrrc3	leucine rich repeat containing 3	1.95
ENSMUSG00000046805	Mpeg1	macrophage expressed gene 1	1.95
ENSMUSG00000034573	Ptpn13	protein tyrosine phosphatase, non-receptor type 13	1.95
ENSMUSG00000013846	St3gal1	ST3 beta-galactoside alpha-2,3-sialyltransferase 1	1.94
ENSMUSG00000069874	Irgm2	immunity-related GTPase family M member 2	1.94
ENSMUSG00000030284	Creld1	cysteine-rich with EGF-like domains 1	1.93
ENSMUSG00000032194	Kank2	KN motif and ankyrin repeat domains 2	1.93
ENSMUSG00000066643	Wdr35	WD repeat domain 35	1.93
ENSMUSG00000071984	Fndc1	fibronectin type III domain containing 1	1.93
ENSMUSG00000051359	Ncald	neurocalcin delta	1.92
ENSMUSG00000039621	Prex1	phosphatidylinositol-3,4,5-trisphosphate-dependent Rac exchange factor 1	1.92
ENSMUSG00000032578	Cish	cytokine inducible SH2-containing protein	1.92
ENSMUSG00000073591	Pcdhb22	protocadherin beta 22	1.92
ENSMUSG00000026727	Rsu1	Ras suppressor protein 1	1.91
ENSMUSG00000024524	Gnal	guanine nucleotide binding protein, alpha stimulating, olfactory type	1.91
ENSMUSG00000031617	Tmem184c	transmembrane protein 184C	1.91
ENSMUSG00000020389	Cdkl3	cyclin-dependent kinase-like 3	1.91
ENSMUSG00000099404	Gm28172	predicted gene 28172	1.91
ENSMUSG00000028581	Laptm5	lysosomal-associated protein transmembrane 5	1.91
ENSMUSG00000048728	Zfp454	zinc finger protein 454	1.91
ENSMUSG00000047945	Marcksl1	MARCKS-like 1	1.90
ENSMUSG00000006369	Fbln1	fibulin 1	1.90
ENSMUSG00000003363	Pld3	phospholipase D family, member 3	1.90
ENSMUSG00000025986	Slc39a10	solute carrier family 39 (zinc transporter), member 10	1.90
ENSMUSG00000041096	Tspyl2	TSPY-like 2	1.90
ENSMUSG00000008206	Cers4	ceramide synthase 4	1.90
ENSMUSG00000003812	Dnase2a	deoxyribonuclease II alpha	1.90
ENSMUSG00000049804	Armcx4	armadillo repeat containing, X-linked 4	1.90
ENSMUSG00000111212	Gm47087	predicted gene, 47087	1.90
ENSMUSG00000044062	Plekhd1os	pleckstrin homology domain containing, family D (with coiled-coil domains) member 1, opposite str	1.90
ENSMUSG00000023088	Abcc1	ATP-binding cassette, sub-family C (CFTR/MRP), member 1	1.89
ENSMUSG00000035376	Hacd2	3-hydroxyacyl-CoA dehydratase 2	1.88
ENSMUSG00000020453	Patz1	POZ (BTB) and AT hook containing zinc finger 1	1.88
ENSMUSG00000037447	Arid5a	AT rich interactive domain 5A (MRF1-like)	1.88
ENSMUSG00000040717	Il17rd	interleukin 17 receptor D	1.88
ENSMUSG00000033906	Zdhhc15	zinc finger, DHHC domain containing 15	1.88
ENSMUSG00000022297	Fzd6	frizzled class receptor 6	1.88
ENSMUSG00000035638	Muc20	mucin 20	1.88
ENSMUSG00000109957	Gm45353	predicted gene 45353	1.88
ENSMUSG00000037254	Itih2	inter-alpha trypsin inhibitor, heavy chain 2	1.88
ENSMUSG00000027997	Casp6	caspase 6	1.87
ENSMUSG00000106847	Peg13	paternally expressed 13	1.87
ENSMUSG00000073565	Prr16	proline rich 16	1.87
ENSMUSG00000022512	Cldn1	claudin 1	1.87
ENSMUSG00000086544	Chn1os3	chimerin 1, opposite strand 3	1.87
ENSMUSG00000048924	Ccdc125	coiled-coil domain containing 125	1.86
ENSMUSG00000059975	Zfp74	zinc finger protein 74	1.86
ENSMUSG00000029156	Sgcb	sarcoglycan, beta (dystrophin-associated glycoprotein)	1.86
ENSMUSG00000016942	Tmprss6	transmembrane serine protease 6	1.86
ENSMUSG00000109245	Gm44860	predicted gene 44860	1.86
ENSMUSG00000056155	Nanos3	nanos C2HC-type zinc finger 3	1.86
ENSMUSG00000019841	Rev3l	REV3 like, DNA directed polymerase zeta catalytic subunit	1.85
ENSMUSG00000071637	Cebpd	CCAAT/enhancer binding protein (C/EBP), delta	1.85
ENSMUSG00000016541	Atxn10	ataxin 10	1.85
ENSMUSG00000042155	Klhl23	kelch-like 23	1.85
ENSMUSG00000051243	Islr2	immunoglobulin superfamily containing leucine-rich repeat 2	1.85

ENSMUSG00000049521	Cdc42ep1	CDC42 effector protein (Rho GTPase binding) 1	1.84
ENSMUSG00000114524	Gm48357	predicted gene, 48357	1.84
ENSMUSG00000026383	Epb41l5	erythrocyte membrane protein band 4.1 like 5	1.83
ENSMUSG00000019256	Ahr	aryl-hydrocarbon receptor	1.83
ENSMUSG00000025075	Habp2	hyaluronic acid binding protein 2	1.83
ENSMUSG00000102758	Naaladl2	N-acetylated alpha-linked acidic dipeptidase-like 2	1.83
ENSMUSG00000049939	Lrrc4	leucine rich repeat containing 4	1.83
ENSMUSG00000069208	Zfp825	zinc finger protein 825	1.83
ENSMUSG00000026458	Ppfia4	protein tyrosine phosphatase, receptor type, f polypeptide (PTPRF), interacting protein (liprin), alph	1.83
ENSMUSG00000070822	Zscan18	zinc finger and SCAN domain containing 18	1.83
ENSMUSG00000023348	Trip6	thyroid hormone receptor interactor 6	1.82
ENSMUSG00000054412	Gm4793	predicted gene 4793	1.82
ENSMUSG00000103818	Gm38009	predicted gene, 38009	1.82
ENSMUSG00000058799	Nap1l1	nucleosome assembly protein 1-like 1	1.81
ENSMUSG00000064061	Dzip3	DAZ interacting protein 3, zinc finger	1.81
ENSMUSG00000035829	Ppp1r26	protein phosphatase 1, regulatory subunit 26	1.81
ENSMUSG00000035390	Brsk1	BR serine/threonine kinase 1	1.81
ENSMUSG00000031480	Thsd1	thrombospondin, type I, domain 1	1.81
ENSMUSG00000078716	Tmem8b	transmembrane protein 8B	1.81
ENSMUSG00000050821	Fam131a	family with sequence similarity 131, member A	1.81
ENSMUSG00000031660	Brd7	bromodomain containing 7	1.80
ENSMUSG00000070000	Fcho1	FCH domain only 1	1.80
ENSMUSG00000050737	Ptges	prostaglandin E synthase	1.80
ENSMUSG00000026479	Lamc2	laminin, gamma 2	1.79
ENSMUSG00000030486	Zfp108	zinc finger protein 108	1.79
ENSMUSG00000030161	Gabarapl1	gamma-aminobutyric acid (GABA) A receptor-associated protein-like 1	1.78
ENSMUSG00000038205	Prkab2	protein kinase, AMP-activated, beta 2 non-catalytic subunit	1.78
ENSMUSG00000054580	Pla2r1	phospholipase A2 receptor 1	1.78
ENSMUSG00000083816	Gm13033	predicted gene 13033	1.78
ENSMUSG00000036867	Smad6	SMAD family member 6	1.77
ENSMUSG00000032300	1700017B05Rik	RIKEN cDNA 1700017B05 gene	1.76
ENSMUSG00000032440	Tgfbr2	transforming growth factor, beta receptor II	1.76
ENSMUSG00000021262	Evl	Ena-vasodilator stimulated phosphoprotein	1.76
ENSMUSG00000039384	Dusp10	dual specificity phosphatase 10	1.76
ENSMUSG00000020650	Bcap29	B cell receptor associated protein 29	1.76
ENSMUSG00000040591	1110051M20Rik	RIKEN cDNA 1110051M20 gene	1.76
ENSMUSG00000020253	Ppm1m	protein phosphatase 1M	1.76
ENSMUSG00000053931	Cnn3	calponin 3, acidic	1.75
ENSMUSG00000028211	Trp53inp1	transformation related protein 53 inducible nuclear protein 1	1.75
ENSMUSG00000008999	Bmp7	bone morphogenetic protein 7	1.75
ENSMUSG00000070305	Mpzl3	myelin protein zero-like 3	1.75
ENSMUSG00000034854	Mfsd12	major facilitator superfamily domain containing 12	1.75
ENSMUSG00000020032	Nuak1	NUAK family, SNF1-like kinase, 1	1.75
ENSMUSG00000038797	Zscan2	zinc finger and SCAN domain containing 2	1.75
ENSMUSG00000103088	Pcdhgb6	protocadherin gamma subfamily B, 6	1.74
ENSMUSG00000016024	Lbp	lipopolysaccharide binding protein	1.74
ENSMUSG00000025185	Loxl4	lysyl oxidase-like 4	1.74
ENSMUSG00000026278	Bok	BCL2-related ovarian killer	1.73
ENSMUSG00000053716	Dusp7	dual specificity phosphatase 7	1.73
ENSMUSG00000004665	Cnn2	calponin 2	1.73
ENSMUSG00000073000	Gm10451	predicted gene 10451	1.73
ENSMUSG00000020570	Sypl	synaptophysin-like protein	1.72
ENSMUSG00000028849	Map7d1	MAP7 domain containing 1	1.72
ENSMUSG00000030315	Vgll4	vestigial like family member 4	1.72
ENSMUSG00000045319	Proser2	proline and serine rich 2	1.72
ENSMUSG00000025265	Fgd1	FYVE, RhoGEF and PH domain containing 1	1.72
ENSMUSG00000103037	Pcdhgb1	protocadherin gamma subfamily B, 1	1.72
ENSMUSG00000047344	Lancl3	LanC lantibiotic synthetase component C-like 3 (bacterial)	1.72
ENSMUSG00000027796	Smad9	SMAD family member 9	1.71
ENSMUSG00000039081	Zfp503	zinc finger protein 503	1.70
ENSMUSG00000032348	Gsta4	glutathione S-transferase, alpha 4	1.70
ENSMUSG00000041886	Macc1	metastasis associated in colon cancer 1	1.70
ENSMUSG00000024013	Fgd2	FYVE, RhoGEF and PH domain containing 2	1.70
ENSMUSG00000002107	Celf2	CUGBP, Elav-like family member 2	1.70
ENSMUSG00000096544	Gm4617	predicted pseudogene 4617	1.70
ENSMUSG00000000058	Cav2	caveolin 2	1.70
ENSMUSG00000038872	Zfhx3	zinc finger homeobox 3	1.69
ENSMUSG00000047407	Tgif1	TGFB-induced factor homeobox 1	1.69
ENSMUSG00000020776	Fbf1	Fas (TNFRSF6) binding factor 1	1.69
ENSMUSG00000017376	Nlk	nemo like kinase	1.69
ENSMUSG00000020848	Doc2b	double C2, beta	1.69
ENSMUSG00000035778	Ggta1	glycoprotein galactosyltransferase alpha 1, 3	1.69
ENSMUSG00000031098	Syt8	synaptotagmin VIII	1.69
ENSMUSG00000020592	Sdc1	syndecan 1	1.68
ENSMUSG00000009585	Apobec3	apolipoprotein B mRNA editing enzyme, catalytic polypeptide 3	1.68
ENSMUSG00000038400	Pmepa1	prostate transmembrane protein, androgen induced 1	1.68

ENSMUSG00000030759	Far1	fatty acyl CoA reductase 1	1.68
ENSMUSG00000045045	Lrfn4	leucine rich repeat and fibronectin type III domain containing 4	1.68
ENSMUSG00000028737	Aldh4a1	aldehyde dehydrogenase 4 family, member A1	1.68
ENSMUSG00000028378	Ptgr1	prostaglandin reductase 1	1.67
ENSMUSG00000025161	Slc16a3	solute carrier family 16 (monocarboxylic acid transporters), member 3	1.67
ENSMUSG00000029246	Ppat	phosphoribosyl pyrophosphate amidotransferase	1.67
ENSMUSG00000000555	Itga5	integrin alpha 5 (fibronectin receptor alpha)	1.67
ENSMUSG00000046572	Zfp518b	zinc finger protein 518B	1.67
ENSMUSG00000028573	Fggy	FGGY carbohydrate kinase domain containing	1.67
ENSMUSG00000038068	Rnf144b	ring finger protein 144B	1.67
ENSMUSG00000037892	Pcdh18	protocadherin 18	1.67
ENSMUSG00000040473	Cfap69	cilia and flagella associated protein 69	1.67
ENSMUSG00000025534	Gusb	glucuronidase, beta	1.66
ENSMUSG00000024070	Prkd3	protein kinase D3	1.66
ENSMUSG00000004105	Angptl2	angiopoietin-like 2	1.66
ENSMUSG00000027995	Tlr2	toll-like receptor 2	1.66
ENSMUSG00000021373	Cap2	CAP, adenylate cyclase-associated protein, 2 (yeast)	1.66
ENSMUSG00000041836	Ptpre	protein tyrosine phosphatase, receptor type, E	1.65
ENSMUSG00000053897	Slc39a8	solute carrier family 39 (metal ion transporter), member 8	1.65
ENSMUSG00000037656	Slc20a2	solute carrier family 20, member 2	1.65
ENSMUSG00000040466	Blvrb	biliverdin reductase B (flavin reductase (NADPH))	1.65
ENSMUSG00000021792	Fam213a	family with sequence similarity 213, member A	1.65
ENSMUSG00000044636	Csrnp2	cysteine-serine-rich nuclear protein 2	1.65
ENSMUSG00000037499	Nenf	neuron derived neurotrophic factor	1.65
ENSMUSG00000020656	Grhl1	grainyhead like transcription factor 1	1.65
ENSMUSG00000018822	Sfrp5	secreted frizzled-related sequence protein 5	1.65
ENSMUSG00000020176	Grb10	growth factor receptor bound protein 10	1.65
ENSMUSG00000091438	Gm17088	predicted gene 17088	1.65
ENSMUSG00000078853	Igtp	interferon gamma induced GTPase	1.64
ENSMUSG00000031365	Zfp275	zinc finger protein 275	1.64
ENSMUSG00000081480	Gm13608	predicted gene 13608	1.64
ENSMUSG00000025083	Afap1l2	actin filament associated protein 1-like 2	1.64
ENSMUSG00000007891	Ctsd	cathepsin D	1.63
ENSMUSG00000033039	Micall1	microtubule associated monooxygenase, calponin and LIM domain containing -like 1	1.63
ENSMUSG00000025268	Maged2	melanoma antigen, family D, 2	1.63
ENSMUSG00000047264	Zfp358	zinc finger protein 358	1.63
ENSMUSG00000017724	Etv4	ets variant 4	1.63
ENSMUSG00000059493	Nhs	NHS actin remodeling regulator	1.63
ENSMUSG00000085972	1110028F11Rik	RIKEN cDNA 1110028F11 gene	1.63
ENSMUSG00000037185	Krt80	keratin 80	1.63
ENSMUSG00000023224	Serping1	serine (or cysteine) peptidase inhibitor, clade G, member 1	1.63
ENSMUSG00000027860	Vangl1	VANGL planar cell polarity 1	1.62
ENSMUSG00000053110	Yap1	yes-associated protein 1	1.61
ENSMUSG00000037679	Inf2	inverted formin, FH2 and WH2 domain containing	1.61
ENSMUSG00000063760	Rnf217	ring finger protein 217	1.61
ENSMUSG00000032531	Amotl2	angiomotin-like 2	1.61
ENSMUSG00000025577	Cbx2	chromobox 2	1.61
ENSMUSG00000032657	Fam189b	family with sequence similarity 189, member B	1.61
ENSMUSG00000038022	Mindy4	MINDY lysine 48 deubiquitinase 4	1.61
ENSMUSG00000025145	Lrrc45	leucine rich repeat containing 45	1.60
ENSMUSG00000022043	Trim35	tripartite motif-containing 35	1.60
ENSMUSG00000047749	Zc3hav1l	zinc finger CCCH-type, antiviral 1-like	1.60
ENSMUSG00000028214	Gem	GTP binding protein (gene overexpressed in skeletal muscle)	1.60
ENSMUSG00000117231	AC079441.1	novel transcript, antisense to Dlgap1	1.60
ENSMUSG00000045349	Sh2d5	SH2 domain containing 5	1.60
ENSMUSG00000018378	Cuedc1	CUE domain containing 1	1.59
ENSMUSG00000010080	Epn3	epsin 3	1.59
ENSMUSG00000023495	Pcbp4	poly(rC) binding protein 4	1.59
ENSMUSG00000043895	S1pr2	sphingosine-1-phosphate receptor 2	1.59
ENSMUSG00000068617	Efcab1	EF-hand calcium binding domain 1	1.59
ENSMUSG00000026335	Pam	peptidylglycine alpha-amidating monooxygenase	1.58
ENSMUSG00000025372	Baiap2	brain-specific angiogenesis inhibitor 1-associated protein 2	1.58
ENSMUSG00000024579	Pcyox1l	prenylcysteine oxidase 1 like	1.58
ENSMUSG00000113665	Gm47603	predicted gene, 47603	1.58
ENSMUSG00000032228	Tcf12	transcription factor 12	1.57
ENSMUSG00000053580	Tanc2	tetratricopeptide repeat, ankyrin repeat and coiled-coil containing 2	1.57
ENSMUSG00000042439	Zfp532	zinc finger protein 532	1.57
ENSMUSG00000020882	Cacnb1	calcium channel, voltage-dependent, beta 1 subunit	1.57
ENSMUSG00000054967	Zfp647	zinc finger protein 647	1.57
ENSMUSG00000030729	Pgm2l1	phosphoglucomutase 2-like 1	1.56
ENSMUSG00000032320	Rcn2	reticulocalbin 2	1.56
ENSMUSG00000002458	Rgs19	regulator of G-protein signaling 19	1.56
ENSMUSG00000027737	Slc7a11	solute carrier family 7 (cationic amino acid transporter, y+ system), member 11	1.56
ENSMUSG00000085116	Gm2735	predicted gene 2735	1.56
ENSMUSG00000087201	Gm15261	predicted gene 15261	1.56
ENSMUSG00000052563	D930048N14Rik	RIKEN cDNA D930048N14 gene	1.55

ENSMUSG00000064125	Prr36	proline rich 36	1.55
ENSMUSG00000008153	Clstn3	calsyntenin 3	1.55
ENSMUSG00000042426	Dhx29	DEAH (Asp-Glu-Ala-His) box polypeptide 29	1.54
ENSMUSG00000001053	N4bp3	NEDD4 binding protein 3	1.54
ENSMUSG00000068115	Ninl	ninein-like	1.54
ENSMUSG00000102440	Pcdhga9	protocadherin gamma subfamily A, 9	1.54
ENSMUSG00000034118	Tpst1	protein-tyrosine sulfotransferase 1	1.54
ENSMUSG00000020723	Cacng4	calcium channel, voltage-dependent, gamma subunit 4	1.54
ENSMUSG00000049580	Tsku	tsukushi, small leucine rich proteoglycan	1.54
ENSMUSG00000006519	Cyba	cytochrome b-245, alpha polypeptide	1.53
ENSMUSG00000025151	Maged1	melanoma antigen, family D, 1	1.53
ENSMUSG00000033319	Fem1c	fem-1 homolog c (C.elegans)	1.53
ENSMUSG00000026425	Srgap2	SLIT-ROBO Rho GTPase activating protein 2	1.53
ENSMUSG00000021720	Rnf180	ring finger protein 180	1.53
ENSMUSG00000026627	Tmem206	transmembrane protein 206	1.53
ENSMUSG00000024909	Efemp2	epidermal growth factor-containing fibulin-like extracellular matrix protein 2	1.53
ENSMUSG00000037890	Wdr19	WD repeat domain 19	1.52
ENSMUSG00000005087	Cd44	CD44 antigen	1.50
ENSMUSG00000032359	Ctsh	cathepsin H	1.50
ENSMUSG00000039831	Arhgap29	Rho GTPase activating protein 29	1.50
ENSMUSG00000025035	Arl3	ADP-ribosylation factor-like 3	1.50
ENSMUSG00000102748	Pcdhgb2	protocadherin gamma subfamily B, 2	1.50
ENSMUSG00000022895	Ets2	E26 avian leukemia oncogene 2, 3' domain	1.49
ENSMUSG00000025153	Fasn	fatty acid synthase	1.49
ENSMUSG00000021482	Aaed1	AhpC/TSA antioxidant enzyme domain containing 1	1.49
ENSMUSG00000028268	Gbp3	guanylate binding protein 3	1.49
ENSMUSG00000074211	Sdhaf1	succinate dehydrogenase complex assembly factor 1	1.49
ENSMUSG00000055639	Dach1	dachshund family transcription factor 1	1.48
ENSMUSG00000024177	Nme4	NME/NM23 nucleoside diphosphate kinase 4	1.48
ENSMUSG00000029999	Tgfa	transforming growth factor alpha	1.47
ENSMUSG00000038007	Acer2	alkaline ceramidase 2	1.47
ENSMUSG00000038886	Man2a2	mannosidase 2, alpha 2	1.47
ENSMUSG00000068270	Shroom4	shroom family member 4	1.47
ENSMUSG00000038587	Akap12	A kinase (PRKA) anchor protein (gravin) 12	1.47
ENSMUSG00000021109	Hif1a	hypoxia inducible factor 1, alpha subunit	1.46
ENSMUSG00000028173	Wls	wntless WNT ligand secretion mediator	1.46
ENSMUSG00000036368	Rmdn2	regulator of microtubule dynamics 2	1.46
ENSMUSG00000027223	Mapk8ip1	mitogen-activated protein kinase 8 interacting protein 1	1.46
ENSMUSG00000034586	Hid1	HID1 domain containing	1.45
ENSMUSG00000020536	Llgl1	LLGL1 scribble cell polarity complex component	1.45
ENSMUSG00000040511	Pvr	poliovirus receptor	1.45
ENSMUSG00000031303	Map3k15	mitogen-activated protein kinase kinase kinase 15	1.45
ENSMUSG00000027784	Ppm1l	protein phosphatase 1 (formerly 2C)-like	1.45
ENSMUSG00000072235	Tuba1a	tubulin, alpha 1A	1.45
ENSMUSG00000048755	Mcat	malonyl CoA:ACP acyltransferase (mitochondrial)	1.45
ENSMUSG00000041729	Coro2b	coronin, actin binding protein, 2B	1.45
ENSMUSG00000059248	Sept9	septin 9	1.44
ENSMUSG00000024098	Twsg1	twisted gastrulation BMP signaling modulator 1	1.44
ENSMUSG00000046668	Cxxc5	CXXC finger 5	1.44
ENSMUSG00000047090	Tmem198b	transmembrane protein 198b	1.44
ENSMUSG00000022637	Cblb	Casitas B-lineage lymphoma b	1.43
ENSMUSG00000040669	Phc1	polyhomeotic 1	1.43
ENSMUSG00000049532	Sall2	spalt like transcription factor 2	1.43
ENSMUSG00000083012	Fam220a	family with sequence similarity 220, member A	1.43
ENSMUSG00000004837	Grap	GRB2-related adaptor protein	1.43
ENSMUSG00000022096	Hr	hairless	1.42
ENSMUSG00000038648	Creb3l2	cAMP responsive element binding protein 3-like 2	1.42
ENSMUSG00000054256	Msi1	musashi RNA-binding protein 1	1.42
ENSMUSG00000031360	Ctps2	cytidine 5'-triphosphate synthase 2	1.42
ENSMUSG00000000861	Bcl11a	B cell CLL/lymphoma 11A (zinc finger protein)	1.42
ENSMUSG00000036968	Cnpy4	canopy FGF signaling regulator 4	1.42
ENSMUSG00000024818	Slc25a45	solute carrier family 25, member 45	1.42
ENSMUSG00000025787	Tgm4	transglutaminase 4 (prostate)	1.42
ENSMUSG00000013698	Pea15a	phosphoprotein enriched in astrocytes 15A	1.41
ENSMUSG00000033900	Map9	microtubule-associated protein 9	1.41
ENSMUSG00000019986	Ahi1	Abelson helper integration site 1	1.41
ENSMUSG00000051391	Ywhag	tyrosine 3-monooxygenase/tryptophan 5-monooxygenase activation protein, gamma polypeptide	1.40
ENSMUSG00000022390	Zc3h7b	zinc finger CCCH type containing 7B	1.40
ENSMUSG00000025223	Ldb1	LIM domain binding 1	1.40
ENSMUSG00000000085	Scmh1	sex comb on midleg homolog 1	1.40
ENSMUSG00000038151	Prdm1	PR domain containing 1, with ZNF domain	1.40
ENSMUSG00000022604	Cep97	centrosomal protein 97	1.40
ENSMUSG00000041135	Ripk2	receptor (TNFRSF)-interacting serine-threonine kinase 2	1.40
ENSMUSG00000052534	Pbx1	pre B cell leukemia homeobox 1	1.39
ENSMUSG00000067071	Hes6	hairy and enhancer of split 6	1.39
ENSMUSG00000028749	Pla2g2f	phospholipase A2, group IIF	1.39

ENSMUSG00000069631	Strada	STE20-related kinase adaptor alpha	1.39
ENSMUSG00000042506	Usp22	ubiquitin specific peptidase 22	1.38
ENSMUSG00000029763	Exoc4	exocyst complex component 4	1.38
ENSMUSG00000060510	Zfp266	zinc finger protein 266	1.38
ENSMUSG00000040681	Hmgn1	high mobility group nucleosomal binding domain 1	1.38
ENSMUSG00000053414	Hunk	hormonally upregulated Neu-associated kinase	1.38
ENSMUSG00000041440	Gk5	glycerol kinase 5 (putative)	1.38
ENSMUSG00000032285	Dnaja4	DnaJ heat shock protein family (Hsp40) member A4	1.38
ENSMUSG00000030725	Lipt2	lipoyl(octanoyl) transferase 2 (putative)	1.38
ENSMUSG00000070883	Ccdc173	coiled-coil domain containing 173	1.38
ENSMUSG00000031626	Sorbs2	sorbin and SH3 domain containing 2	1.37
ENSMUSG00000039801	Cplane1	ciliogenesis and planar polarity effector 1	1.37
ENSMUSG00000024855	Pacs1	phosphofurin acidic cluster sorting protein 1	1.37
ENSMUSG00000004768	Rab23	RAB23, member RAS oncogene family	1.37
ENSMUSG00000031342	Gpm6b	glycoprotein m6b	1.37
ENSMUSG00000107205	Gm42576	predicted gene 42576	1.37
ENSMUSG00000036097	Slf2	SMC5-SMC6 complex localization factor 2	1.36
ENSMUSG00000002489	Tiam1	T cell lymphoma invasion and metastasis 1	1.36
ENSMUSG00000027189	Trim44	tripartite motif-containing 44	1.36
ENSMUSG00000066357	Wdr6	WD repeat domain 6	1.36
ENSMUSG00000057147	Dph6	diphthamine biosynthesis 6	1.36
ENSMUSG00000095098	Ccdc85b	coiled-coil domain containing 85B	1.36
ENSMUSG00000074825	Itpripl1	inositol 1,4,5-triphosphate receptor interacting protein-like 1	1.36
ENSMUSG00000035104	Eva1a	eva-1 homolog A (C. elegans)	1.36
ENSMUSG00000039262	Prrc2b	proline-rich coiled-coil 2B	1.35
ENSMUSG00000026305	Lrrfip1	leucine rich repeat (in FLII) interacting protein 1	1.35
ENSMUSG00000037145	2210407C18Rik	RIKEN cDNA 2210407C18 gene	1.35
ENSMUSG00000022475	Hdac7	histone deacetylase 7	1.35
ENSMUSG00000018199	Trove2	TROVE domain family, member 2	1.35
ENSMUSG00000000325	Arvcf	armadillo repeat gene deleted in velocardiofacial syndrome	1.35
ENSMUSG00000037640	Zfp60	zinc finger protein 60	1.35
ENSMUSG00000030980	Knop1	lysine rich nucleolar protein 1	1.34
ENSMUSG00000039976	Tbc1d16	TBC1 domain family, member 16	1.34
ENSMUSG00000020654	Adcy3	adenylate cyclase 3	1.34
ENSMUSG00000031861	Lpar2	lysophosphatidic acid receptor 2	1.34
ENSMUSG00000039481	Nrtn	neurturin	1.34
ENSMUSG00000054693	Adam10	a disintegrin and metallopeptidase domain 10	1.33
ENSMUSG00000029822	Osbpl3	oxysterol binding protein-like 3	1.33
ENSMUSG00000019461	Plscr3	phospholipid scramblase 3	1.33
ENSMUSG00000031434	Morc4	microrchidia 4	1.33
ENSMUSG00000024350	Dnajc18	DnaJ heat shock protein family (Hsp40) member C18	1.33
ENSMUSG00000023947	Nfkbie	nuclear factor of kappa light polypeptide gene enhancer in B cells inhibitor, epsilon	1.33
ENSMUSG00000038264	Sema7a	sema domain, immunoglobulin domain (Ig), and GPI membrane anchor, (semaphorin) 7A	1.32
ENSMUSG00000020015	Cdk17	cyclin-dependent kinase 17	1.32
ENSMUSG00000035776	Cd99l2	CD99 antigen-like 2	1.32
ENSMUSG00000033773	Rpap2	RNA polymerase II associated protein 2	1.32
ENSMUSG00000036052	Dnajb5	DnaJ heat shock protein family (Hsp40) member B5	1.32
ENSMUSG00000025321	Itgb8	integrin beta 8	1.32
ENSMUSG00000027878	Notch2	notch 2	1.31
ENSMUSG00000033669	Zfp7	zinc finger protein 7	1.31
ENSMUSG00000033545	Znrf1	zinc and ring finger 1	1.31
ENSMUSG00000061981	Flot2	flotillin 2	1.31
ENSMUSG00000040811	Eml2	echinoderm microtubule associated protein like 2	1.31
ENSMUSG00000038718	Pbx3	pre B cell leukemia homeobox 3	1.31
ENSMUSG00000014907	Naf1	nuclear assembly factor 1 ribonucleoprotein	1.31
ENSMUSG00000046223	Plaur	plasminogen activator, urokinase receptor	1.31
ENSMUSG00000107000	Gm43481	predicted gene 43481	1.31
ENSMUSG00000085433	Gm16001	predicted gene 16001	1.31
ENSMUSG00000072694	1500011B03Rik	RIKEN cDNA 1500011B03 gene	1.31
ENSMUSG00000027684	Mecom	MDS1 and EVI1 complex locus	1.30
ENSMUSG00000033767	Tmem131l	transmembrane 131 like	1.30
ENSMUSG00000086040	Wipf3	WAS/WASL interacting protein family, member 3	1.30
ENSMUSG00000022098	Bmp1	bone morphogenetic protein 1	1.30
ENSMUSG00000025203	Scd2	stearoyl-Coenzyme A desaturase 2	1.29
ENSMUSG00000036206	Sh3bp4	SH3-domain binding protein 4	1.29
ENSMUSG00000025969	Nrp2	neuropilin 2	1.29
ENSMUSG00000036006	Ripor2	RHO family interacting cell polarization regulator 2	1.29
ENSMUSG00000021368	Tbc1d7	TBC1 domain family, member 7	1.29
ENSMUSG00000007659	Bcl2l1	BCL2-like 1	1.28
ENSMUSG00000041238	Rbbp8	retinoblastoma binding protein 8, endonuclease	1.28
ENSMUSG00000046574	Prr12	proline rich 12	1.28
ENSMUSG00000020142	Slc1a4	solute carrier family 1 (glutamate/neutral amino acid transporter), member 4	1.28
ENSMUSG00000040296	Ddx58	DEAD (Asp-Glu-Ala-Asp) box polypeptide 58	1.28
ENSMUSG00000024501	Dpysl3	dihydropyrimidinase-like 3	1.28
ENSMUSG00000028599	Tnfrsf1b	tumor necrosis factor receptor superfamily, member 1b	1.28
ENSMUSG00000025931	Paqr8	progestin and adipoQ receptor family member VIII	1.28

ENSMUSG00000032883	Acsl3	acyl-CoA synthetase long-chain family member 3	1.27
ENSMUSG00000021277	Traf3	TNF receptor-associated factor 3	1.27
ENSMUSG00000006800	Sulf2	sulfatase 2	1.27
ENSMUSG00000027122	Arl14ep	ADP-ribosylation factor-like 14 effector protein	1.27
ENSMUSG00000030170	Wnt5b	wingless-type MMTV integration site family, member 5B	1.27
ENSMUSG00000020607	Fam84a	family with sequence similarity 84, member A	1.26
ENSMUSG00000029229	Chic2	cysteine-rich hydrophobic domain 2	1.26
ENSMUSG00000022971	Ifnar2	interferon (alpha and beta) receptor 2	1.26
ENSMUSG00000025082	Vwa2	von Willebrand factor A domain containing 2	1.26
ENSMUSG00000031799	Tpm4	tropomyosin 4	1.25
ENSMUSG00000035547	Capn5	calpain 5	1.25
ENSMUSG00000048371	Pdp2	pyruvate dehyrogenase phosphatase catalytic subunit 2	1.25
ENSMUSG00000043467	Zbtb37	zinc finger and BTB domain containing 37	1.25
ENSMUSG00000019261	Map1s	microtubule-associated protein 1S	1.25
ENSMUSG00000030323	Ift122	intraflagellar transport 122	1.25
ENSMUSG00000117123	AL805899.1	novel transcript	1.25
ENSMUSG00000043461	Sptssb	serine palmitoyltransferase, small subunit B	1.25
ENSMUSG00000021782	Dlg5	discs large MAGUK scaffold protein 5	1.24
ENSMUSG00000036885	Arhgef26	Rho guanine nucleotide exchange factor (GEF) 26	1.24
ENSMUSG00000031278	Acsl4	acyl-CoA synthetase long-chain family member 4	1.24
ENSMUSG00000070803	Cited4	Cbp/p300-interacting transactivator, with Glu/Asp-rich carboxy-terminal domain, 4	1.24
ENSMUSG00000079038	D130040H23Rik	RIKEN cDNA D130040H23 gene	1.24
ENSMUSG00000031903	Pla2g15	phospholipase A2, group XV	1.24
ENSMUSG00000023169	Slc38a1	solute carrier family 38, member 1	1.23
ENSMUSG00000022723	Crybg3	beta-gamma crystallin domain containing 3	1.23
ENSMUSG00000058135	Gstm1	glutathione S-transferase, mu 1	1.23
ENSMUSG00000021608	Lpcat1	lysophosphatidylcholine acyltransferase 1	1.23
ENSMUSG00000068758	Il3ra	interleukin 3 receptor, alpha chain	1.23
ENSMUSG00000019966	Kitl	kit ligand	1.22
ENSMUSG00000050912	Tmem123	transmembrane protein 123	1.22
ENSMUSG00000019433	Gipc1	GIPC PDZ domain containing family, member 1	1.22
ENSMUSG00000021774	Ube2e1	ubiquitin-conjugating enzyme E2E 1	1.22
ENSMUSG00000028344	Invs	inversin	1.22
ENSMUSG00000038721	Hoxb7	homeobox B7	1.21
ENSMUSG00000028601	Echdc2	enoyl Coenzyme A hydratase domain containing 2	1.21
ENSMUSG00000045211	Nudt18	nudix (nucleoside diphosphate linked moiety X)-type motif 18	1.21
ENSMUSG00000006356	Crip2	cysteine rich protein 2	1.21
ENSMUSG00000083282	Ctsf	cathepsin F	1.21
ENSMUSG00000035021	Baz1a	bromodomain adjacent to zinc finger domain 1A	1.20
ENSMUSG00000039985	Sinhcaf	SIN3-HDAC complex associated factor	1.20
ENSMUSG00000040446	Rprd1a	regulation of nuclear pre-mRNA domain containing 1A	1.20
ENSMUSG00000025262	Fam120c	family with sequence similarity 120, member C	1.20
ENSMUSG00000025196	Cpn1	carboxypeptidase N, polypeptide 1	1.20
ENSMUSG00000051705	Senp8	SUMO/sentrin specific peptidase 8	1.20
ENSMUSG00000035713	Usp35	ubiquitin specific peptidase 35	1.20
ENSMUSG00000015745	Plekho1	pleckstrin homology domain containing, family O member 1	1.20
ENSMUSG00000063382	Bcl9l	B cell CLL/lymphoma 9-like	1.19
ENSMUSG00000020376	Rnf130	ring finger protein 130	1.19
ENSMUSG00000048264	Dip2c	disco interacting protein 2 homolog C	1.19
ENSMUSG00000058704	Memo1	mediator of cell motility 1	1.19
ENSMUSG00000020393	Kremen1	kringle containing transmembrane protein 1	1.19
ENSMUSG00000033970	Rfc3	replication factor C (activator 1) 3	1.19
ENSMUSG00000038301	Snx10	sorting nexin 10	1.19
ENSMUSG00000067928	Zfp760	zinc finger protein 760	1.19
ENSMUSG00000043542	Zc2hc1a	zinc finger, C2HC-type containing 1A	1.19
ENSMUSG00000074785	Plxnc1	plexin C1	1.19
ENSMUSG00000070565	Rasal2	RAS protein activator like 2	1.18
ENSMUSG00000042105	Inpp5f	inositol polyphosphate-5-phosphatase F	1.18
ENSMUSG00000040502	39873	membrane-associated ring finger (C3HC4) 9	1.18
ENSMUSG00000044254	Pcsk9	proprotein convertase subtilisin/kexin type 9	1.17
ENSMUSG00000040928	S100pbp	S100P binding protein	1.17
ENSMUSG00000049439	Cyp20a1	cytochrome P450, family 20, subfamily a, polypeptide 1	1.17
ENSMUSG00000038387	Rras	related RAS viral (r-ras) oncogene	1.17
ENSMUSG00000087366	Junos	jun proto-oncogene, opposite strand	1.17
ENSMUSG00000109324	Prmt1	protein arginine N-methyltransferase 1	1.16
ENSMUSG00000041895	Wipi1	WD repeat domain, phosphoinositide interacting 1	1.16
ENSMUSG00000021451	Sema4d	sema domain, immunoglobulin domain (Ig), transmembrane domain (TM) and short cytoplasmic do	1.16
ENSMUSG00000027533	Fabp5	fatty acid binding protein 5, epidermal	1.16
ENSMUSG00000025887	Casp12	caspase 12	1.16
ENSMUSG00000004961	Syt5	synaptotagmin V	1.16
ENSMUSG00000041298	Katnal1	katanin p60 subunit A-like 1	1.16
ENSMUSG00000020875	Hoxb9	homeobox B9	1.15
ENSMUSG00000026421	Csrp1	cysteine and glycine-rich protein 1	1.15
ENSMUSG00000033454	Zbtb1	zinc finger and BTB domain containing 1	1.15
ENSMUSG00000030946	Lhpp	phospholysine phosphohistidine inorganic pyrophosphate phosphatase	1.15
ENSMUSG00000031641	Cbr4	carbonyl reductase 4	1.15

ENSMUSG00000069114	Zbtb10	zinc finger and BTB domain containing 10	1.15
ENSMUSG00000087177	E130307A14Rik	RIKEN cDNA E130307A14 gene	1.15
ENSMUSG00000057133	Chd6	chromodomain helicase DNA binding protein 6	1.14
ENSMUSG00000026566	Mpzl1	myelin protein zero-like 1	1.14
ENSMUSG00000031150	Ccdc120	coiled-coil domain containing 120	1.14
ENSMUSG00000028542	Slc6a9	solute carrier family 6 (neurotransmitter transporter, glycine), member 9	1.14
ENSMUSG00000025323	Sp4	trans-acting transcription factor 4	1.14
ENSMUSG00000062590	Armc9	armadillo repeat containing 9	1.14
ENSMUSG00000115902	AC113595.1	novel transcript, antisense to Apobec3	1.14
ENSMUSG00000022837	Iqcb1	IQ calmodulin-binding motif containing 1	1.14
ENSMUSG00000115783	Bc1	brain cytoplasmic RNA 1	1.14
ENSMUSG00000032216	Nedd4	neural precursor cell expressed, developmentally down-regulated 4	1.13
ENSMUSG00000014601	Strip1	striatin interacting protein 1	1.13
ENSMUSG00000025142	Aspscr1	alveolar soft part sarcoma chromosome region, candidate 1 (human)	1.13
ENSMUSG00000034981	Parm1	prostate androgen-regulated mucin-like protein 1	1.13
ENSMUSG00000022822	Abcc5	ATP-binding cassette, sub-family C (CFTR/MRP), member 5	1.13
ENSMUSG00000028245	Nsmaf	neutral sphingomyelinase (N-SMase) activation associated factor	1.13
ENSMUSG00000046432	Bex3	brain expressed X-linked 3	1.13
ENSMUSG00000026484	Rnf2	ring finger protein 2	1.13
ENSMUSG00000032481	Smarcc1	SWI/SNF related, matrix associated, actin dependent regulator of chromatin, subfamily c, member 1	1.12
ENSMUSG00000029478	Ncor2	nuclear receptor co-repressor 2	1.12
ENSMUSG00000030413	Pglyrp1	peptidoglycan recognition protein 1	1.12
ENSMUSG00000004846	Plod3	procollagen-lysine, 2-oxoglutarate 5-dioxygenase 3	1.12
ENSMUSG00000022197	Pdzd2	PDZ domain containing 2	1.12
ENSMUSG00000056148	Rdh9	retinol dehydrogenase 9	1.12
ENSMUSG00000042050	Wdr60	WD repeat domain 60	1.12
ENSMUSG00000074283	Zfp109	zinc finger protein 109	1.12
ENSMUSG00000112964	E430024I08Rik	RIKEN cDNA E430024I08 gene	1.12
ENSMUSG00000019838	Slc16a10	solute carrier family 16 (monocarboxylic acid transporters), member 10	1.11
ENSMUSG00000028919	Arhgef19	Rho guanine nucleotide exchange factor (GEF) 19	1.11
ENSMUSG00000037933	Bicd2	BICD cargo adaptor 2	1.11
ENSMUSG00000028126	Pip5k1a	phosphatidylinositol-4-phosphate 5-kinase, type 1 alpha	1.11
ENSMUSG00000067430	Zfp763	zinc finger protein 763	1.11
ENSMUSG00000025171	Ubtd1	ubiquitin domain containing 1	1.11
ENSMUSG00000011254	Thg1l	tRNA-histidine guanylyltransferase 1-like (S. cerevisiae)	1.11
ENSMUSG00000016487	Ppfibp1	PTPRF interacting protein, binding protein 1 (liprin beta 1)	1.10
ENSMUSG00000052949	Rnf157	ring finger protein 157	1.10
ENSMUSG00000024640	Psat1	phosphoserine aminotransferase 1	1.10
ENSMUSG00000020167	Tcf3	transcription factor 3	1.09
ENSMUSG00000036275	9530068E07Rik	RIKEN cDNA 9530068E07 gene	1.09
ENSMUSG00000051934	Spats2	spermatogenesis associated, serine-rich 2	1.09
ENSMUSG00000026782	Abi2	abl-interactor 2	1.09
ENSMUSG00000050714	Zbtb26	zinc finger and BTB domain containing 26	1.09
ENSMUSG00000022003	Slc25a30	solute carrier family 25, member 30	1.09
ENSMUSG00000029657	Hsph1	heat shock 105kDa/110kDa protein 1	1.08
ENSMUSG00000037706	Cd81	CD81 antigen	1.08
ENSMUSG00000020140	Lgr5	leucine rich repeat containing G protein coupled receptor 5	1.08
ENSMUSG00000048047	Zbtb33	zinc finger and BTB domain containing 33	1.08
ENSMUSG00000040327	Cul9	cullin 9	1.08
ENSMUSG00000049300	Prmt6	protein arginine N-methyltransferase 6	1.08
ENSMUSG00000024269	Tpgs2	tubulin polyglutamylase complex subunit 2	1.08
ENSMUSG00000021108	Prkch	protein kinase C, eta	1.08
ENSMUSG00000033565	Rbfox2	RNA binding protein, fox-1 homolog (C. elegans) 2	1.07
ENSMUSG00000046312	AI464131	expressed sequence AI464131	1.07
ENSMUSG00000027495	Fam210b	family with sequence similarity 210, member B	1.07
ENSMUSG00000022434	Fam118a	family with sequence similarity 118, member A	1.07
ENSMUSG00000045071	E130308A19Rik	RIKEN cDNA E130308A19 gene	1.07
ENSMUSG00000037907	Ankrd13b	ankyrin repeat domain 13b	1.07
ENSMUSG00000034723	Tmx4	thioredoxin-related transmembrane protein 4	1.07
ENSMUSG00000027778	Ift80	intraflagellar transport 80	1.07
ENSMUSG00000108238	Gm43984	predicted gene, 43984	1.07
ENSMUSG00000028953	Abcf2	ATP-binding cassette, sub-family F (GCN20), member 2	1.06
ENSMUSG00000006728	Cdk4	cyclin-dependent kinase 4	1.06
ENSMUSG00000059552	Trp53	transformation related protein 53	1.06
ENSMUSG00000061689	Dlgap4	DLG associated protein 4	1.06
ENSMUSG00000001761	Smo	smoothened, frizzled class receptor	1.06
ENSMUSG00000045598	Zfp553	zinc finger protein 553	1.06
ENSMUSG00000027551	Zfp64	zinc finger protein 64	1.06
ENSMUSG00000079478	Sssca1	Sjogren syndrome/scleroderma autoantigen 1	1.06
ENSMUSG00000001525	Tubb5	tubulin, beta 5 class I	1.05
ENSMUSG00000020458	Rtn4	reticulon 4	1.05
ENSMUSG00000023039	Krt7	keratin 7	1.05
ENSMUSG00000034300	Fam53c	family with sequence similarity 53, member C	1.05
ENSMUSG00000034708	Grn	granulin	1.04
ENSMUSG00000028484	Psip1	PC4 and SFRS1 interacting protein 1	1.04
ENSMUSG00000022673	Mcm4	minichromosome maintenance complex component 4	1.04

ENSMUSG00000036099	Vezt	vezatin, adherens junctions transmembrane protein	1.04
ENSMUSG00000044968	Napepld	N-acyl phosphatidylethanolamine phospholipase D	1.04
ENSMUSG00000024906	Mus81	MUS81 structure-specific endonuclease subunit	1.04
ENSMUSG00000035451	Foxa1	forkhead box A1	1.03
ENSMUSG00000028832	Stmn1	stathmin 1	1.03
ENSMUSG00000038034	Igsf8	immunoglobulin superfamily, member 8	1.03
ENSMUSG00000060301	2610008E11Rik	RIKEN cDNA 2610008E11 gene	1.03
ENSMUSG00000049882	Vcpkmt	valosin containing protein lysine (K) methyltransferase	1.03
ENSMUSG00000033060	Lmo7	LIM domain only 7	1.02
ENSMUSG00000024811	Tnks2	tankyrase, TRF1-interacting ankyrin-related ADP-ribose polymerase 2	1.02
ENSMUSG00000025085	Ablim1	actin-binding LIM protein 1	1.02
ENSMUSG00000027087	Itgav	integrin alpha V	1.02
ENSMUSG00000022952	Runx1	runt related transcription factor 1	1.02
ENSMUSG00000001630	Stk38l	serine/threonine kinase 38 like	1.02
ENSMUSG00000002870	Mcm2	minichromosome maintenance complex component 2	1.02
ENSMUSG00000105703	Gm43305	predicted gene 43305	1.02
ENSMUSG00000058318	Phf21a	PHD finger protein 21A	1.02
ENSMUSG00000019971	Cep290	centrosomal protein 290	1.02
ENSMUSG00000021068	Nin	ninein	1.02
ENSMUSG00000028517	Plpp3	phospholipid phosphatase 3	1.02
ENSMUSG00000051022	Hs3st1	heparan sulfate (glucosamine) 3-O-sulfotransferase 1	1.02
ENSMUSG00000033987	Dnah17	dynein, axonemal, heavy chain 17	1.02
ENSMUSG00000022773	Ypel1	yippee like 1	1.02
ENSMUSG00000039671	Zmynd8	zinc finger, MYND-type containing 8	1.01
ENSMUSG00000026639	Lamb3	laminin, beta 3	1.01
ENSMUSG00000035671	Zswim4	zinc finger SWIM-type containing 4	1.01
ENSMUSG00000022964	Tmem50b	transmembrane protein 50B	1.01
ENSMUSG00000056612	Ppp1r14b	protein phosphatase 1, regulatory inhibitor subunit 14B	1.01
ENSMUSG00000055897	Ppp4r1l-ps	protein phosphatase 4, regulatory subunit 1-like, pseudogene	1.01
ENSMUSG00000018001	Cyth3	cytohesin 3	1.01
ENSMUSG00000034064	Poglut1	protein O-glucosyltransferase 1	1.01
ENSMUSG00000036093	Arl5a	ADP-ribosylation factor-like 5A	1.00
ENSMUSG00000029461	Fam168a	family with sequence similarity 168, member A	1.00
ENSMUSG00000041343	Ankrd42	ankyrin repeat domain 42	1.00
ENSMUSG00000042638	Gucy2c	guanylate cyclase 2c	-1.00
ENSMUSG00000022900	Ildr1	immunoglobulin-like domain containing receptor 1	-1.00
ENSMUSG00000032633	Flcn	folliculin	-1.00
ENSMUSG00000062110	Scfd2	Sec1 family domain containing 2	-1.00
ENSMUSG00000032593	Amigo3	adhesion molecule with Ig like domain 3	-1.00
ENSMUSG00000049608	Gpr55	G protein-coupled receptor 55	-1.00
ENSMUSG00000027422	Rrbp1	ribosome binding protein 1	-1.01
ENSMUSG00000044042	Fmn1	formin 1	-1.01
ENSMUSG00000090035	Galnt4	polypeptide N-acetylgalactosaminyltransferase 4	-1.01
ENSMUSG00000040048	Ndufb10	NADH:ubiquinone oxidoreductase subunit B10	-1.01
ENSMUSG00000005802	Slc30a4	solute carrier family 30 (zinc transporter), member 4	-1.01
ENSMUSG00000063206	Defa34	defensin, alpha, 34	-1.01
ENSMUSG00000017707	Serinc3	serine incorporator 3	-1.02
ENSMUSG00000020456	Ogdh	oxoglutarate (alpha-ketoglutarate) dehydrogenase (lipoamide)	-1.02
ENSMUSG00000028792	Ak2	adenylate kinase 2	-1.02
ENSMUSG00000039031	Arhgap18	Rho GTPase activating protein 18	-1.02
ENSMUSG00000018427	Ypel2	yippee like 2	-1.02
ENSMUSG00000070284	Gmppb	GDP-mannose pyrophosphorylase B	-1.02
ENSMUSG00000021684	Pde8b	phosphodiesterase 8B	-1.02
ENSMUSG00000051335	Gfod1	glucose-fructose oxidoreductase domain containing 1	-1.02
ENSMUSG00000051517	Arhgef39	Rho guanine nucleotide exchange factor (GEF) 39	-1.02
ENSMUSG00000029136	Rbks	ribokinase	-1.02
ENSMUSG00000104443	4932442E05Rik	RIKEN cDNA 4932442E05 gene	-1.02
ENSMUSG00000038859	Baiap2l1	BAI1-associated protein 2-like 1	-1.03
ENSMUSG00000018899	Irf1	interferon regulatory factor 1	-1.03
ENSMUSG00000009863	Sdhb	succinate dehydrogenase complex, subunit B, iron sulfur (Ip)	-1.03
ENSMUSG00000024190	Dusp1	dual specificity phosphatase 1	-1.03
ENSMUSG00000008540	Mgst1	microsomal glutathione S-transferase 1	-1.03
ENSMUSG00000032602	Slc25a20	solute carrier family 25 (mitochondrial carnitine/acylcarnitine translocase), member 20	-1.03
ENSMUSG00000015536	Mocs2	molybdenum cofactor synthesis 2	-1.03
ENSMUSG00000025006	Sorbs1	sorbin and SH3 domain containing 1	-1.03
ENSMUSG00000031089	Slc6a14	solute carrier family 6 (neurotransmitter transporter), member 14	-1.03
ENSMUSG00000022332	Khdrbs3	KH domain containing, RNA binding, signal transduction associated 3	-1.03
ENSMUSG00000034731	Dgkh	diacylglycerol kinase, eta	-1.03
ENSMUSG00000020334	Slc22a4	solute carrier family 22 (organic cation transporter), member 4	-1.03
ENSMUSG00000025545	Clybl	citrate lyase beta like	-1.03
ENSMUSG00000038702	Dsel	dermatan sulfate epimerase-like	-1.03
ENSMUSG00000028137	Celf3	CUGBP, Elav-like family member 3	-1.03
ENSMUSG00000050777	Tmem37	transmembrane protein 37	-1.03
ENSMUSG00000020220	Vps13d	vacuolar protein sorting 13D	-1.04
ENSMUSG00000041870	Ankrd13a	ankyrin repeat domain 13a	-1.04
ENSMUSG00000020741	Cluh	clustered mitochondria (cluA/CLU1) homolog	-1.04

ENSMUSG00000048707	Tprn	taperin	-1.04
ENSMUSG00000007036	Abhd16a	abhydrolase domain containing 16A	-1.04
ENSMUSG00000070730	Rmdn3	regulator of microtubule dynamics 3	-1.04
ENSMUSG00000033902	Mapkbp1	mitogen-activated protein kinase binding protein 1	-1.04
ENSMUSG00000025466	Fuom	fucose mutarotase	-1.04
ENSMUSG00000050721	Plekho2	pleckstrin homology domain containing, family O member 2	-1.04
ENSMUSG00000032278	Paqr5	progestin and adipoQ receptor family member V	-1.04
ENSMUSG00000030739	Myh14	myosin, heavy polypeptide 14	-1.05
ENSMUSG00000039953	Clstn1	calsyntenin 1	-1.05
ENSMUSG00000038393	Txnip	thioredoxin interacting protein	-1.05
ENSMUSG00000034109	Golim4	golgi integral membrane protein 4	-1.05
ENSMUSG00000021876	Rnase4	ribonuclease, RNase A family 4	-1.05
ENSMUSG00000024338	Psmb8	proteasome (prosome, macropain) subunit, beta type 8 (large multifunctional peptidase 7)	-1.05
ENSMUSG00000042677	Zc3h12a	zinc finger CCCH type containing 12A	-1.05
ENSMUSG00000052396	Mogat2	monoacylglycerol O-acyltransferase 2	-1.05
ENSMUSG00000033187	BC016579	cDNA sequence, BC016579	-1.05
ENSMUSG00000038332	Sesn1	sestrin 1	-1.05
ENSMUSG00000092274	Neat1	nuclear paraspeckle assembly transcript 1 (non-protein coding)	-1.06
ENSMUSG00000019302	Atp6v0a1	ATPase, H+ transporting, lysosomal V0 subunit A1	-1.06
ENSMUSG00000021591	Glrx	glutaredoxin	-1.06
ENSMUSG00000021236	Entpd5	ectonucleoside triphosphate diphosphohydrolase 5	-1.06
ENSMUSG00000033124	Atg9a	autophagy related 9A	-1.06
ENSMUSG00000064120	Mocs1	molybdenum cofactor synthesis 1	-1.06
ENSMUSG00000025002	Cyp2c55	cytochrome P450, family 2, subfamily c, polypeptide 55	-1.06
ENSMUSG00000038987	Cfap157	cilia and flagella associated protein 157	-1.06
ENSMUSG00000020029	Nudt4	nudix (nucleoside diphosphate linked moiety X)-type motif 4	-1.07
ENSMUSG00000040479	Dgkz	diacylglycerol kinase zeta	-1.07
ENSMUSG00000036402	Gng12	guanine nucleotide binding protein (G protein), gamma 12	-1.07
ENSMUSG00000022575	Gsdmd	gasdermin D	-1.07
ENSMUSG00000024887	Asah2	N-acylsphingosine amidohydrolase 2	-1.07
ENSMUSG00000027524	Edn3	endothelin 3	-1.07
ENSMUSG00000026856	Dolpp1	dolichyl pyrophosphate phosphatase 1	-1.07
ENSMUSG00000024875	Yif1a	Yip1 interacting factor homolog A (S. cerevisiae)	-1.07
ENSMUSG00000033161	Atp1a1	ATPase, Na+/K+ transporting, alpha 1 polypeptide	-1.08
ENSMUSG00000007038	Neu1	neuraminidase 1	-1.08
ENSMUSG00000033526	Ppip5k1	diphosphoinositol pentakisphosphate kinase 1	-1.08
ENSMUSG00000034613	Ppm1h	protein phosphatase 1H (PP2C domain containing)	-1.08
ENSMUSG00000035509	Fbxl21	F-box and leucine-rich repeat protein 21	-1.08
ENSMUSG00000024900	Cpt1a	carnitine palmitoyltransferase 1a, liver	-1.09
ENSMUSG00000027406	Idh3b	isocitrate dehydrogenase 3 (NAD+) beta	-1.09
ENSMUSG00000034656	Cacna1a	calcium channel, voltage-dependent, P/Q type, alpha 1A subunit	-1.09
ENSMUSG00000040618	Pck2	phosphoenolpyruvate carboxykinase 2 (mitochondrial)	-1.09
ENSMUSG00000031327	Chic1	cysteine-rich hydrophobic domain 1	-1.09
ENSMUSG00000041654	Slc39a11	solute carrier family 39 (metal ion transporter), member 11	-1.09
ENSMUSG00000052776	Oas1a	2'-5' oligoadenylate synthetase 1A	-1.09
ENSMUSG00000103711	Tstd1	thiosulfate sulfurtransferase (rhodanese)-like domain containing 1	-1.09
ENSMUSG00000035041	Creb3l3	cAMP responsive element binding protein 3-like 3	-1.09
ENSMUSG00000089960	Ugt1a1	UDP glucuronosyltransferase 1 family, polypeptide A1	-1.09
ENSMUSG00000103693	Gm37529	predicted gene, 37529	-1.09
ENSMUSG00000029189	Sel1l3	sel-1 suppressor of lin-12-like 3 (C. elegans)	-1.10
ENSMUSG00000044786	Zfp36	zinc finger protein 36	-1.10
ENSMUSG00000020736	Nt5c	5',3'-nucleotidase, cytosolic	-1.10
ENSMUSG00000000204	Slfn4	schlafen 4	-1.10
ENSMUSG00000016239	Lonrf3	LON peptidase N-terminal domain and ring finger 3	-1.10
ENSMUSG00000000184	Ccnd2	cyclin D2	-1.11
ENSMUSG00000046447	Camk2n1	calcium/calmodulin-dependent protein kinase II inhibitor 1	-1.11
ENSMUSG00000020577	Tspan13	tetraspanin 13	-1.11
ENSMUSG00000018334	Ksr1	kinase suppressor of ras 1	-1.11
ENSMUSG00000018509	Cenpv	centromere protein V	-1.11
ENSMUSG00000031073	Fgf15	fibroblast growth factor 15	-1.11
ENSMUSG00000025227	Mfsd13a	major facilitator superfamily domain containing 13a	-1.11
ENSMUSG00000023473	Celsr3	cadherin, EGF LAG seven-pass G-type receptor 3	-1.11
ENSMUSG00000025195	Dnmbp	dynamin binding protein	-1.12
ENSMUSG00000034485	Uaca	uveal autoantigen with coiled-coil domains and ankyrin repeats	-1.12
ENSMUSG00000020432	Tcn2	transcobalamin 2	-1.12
ENSMUSG00000061455	Stx17	syntaxin 17	-1.12
ENSMUSG00000013160	Atp6v0d1	ATPase, H+ transporting, lysosomal V0 subunit D1	-1.12
ENSMUSG00000049422	Chchd10	coiled-coil-helix-coiled-coil-helix domain containing 10	-1.12
ENSMUSG00000031168	Ebp	phenylalkylamine Ca2+ antagonist (emopamil) binding protein	-1.12
ENSMUSG00000036390	Gadd45a	growth arrest and DNA-damage-inducible 45 alpha	-1.12
ENSMUSG00000031788	Kifc3	kinesin family member C3	-1.12
ENSMUSG00000060147	Serpinb6a	serine (or cysteine) peptidase inhibitor, clade B, member 6a	-1.13
ENSMUSG00000019866	Crybg1	crystallin beta-gamma domain containing 1	-1.13
ENSMUSG00000034435	Tmem30b	transmembrane protein 30B	-1.13
ENSMUSG00000043003	Rasef	RAS and EF hand domain containing	-1.13
ENSMUSG00000042377	Fam83g	family with sequence similarity 83, member G	-1.13

ENSMUSG00000045312	Lhfpl2	lipoma HMGIC fusion partner-like 2	-1.13
ENSMUSG00000041625	Ggact	gamma-glutamylamine cyclotransferase	-1.13
ENSMUSG00000002329	Mdp1	magnesium-dependent phosphatase 1	-1.13
ENSMUSG00000045934	Mtmr11	myotubularin related protein 11	-1.13
ENSMUSG00000035835	Plppr3	phospholipid phosphatase related 3	-1.13
ENSMUSG00000069456	Rdh16	retinol dehydrogenase 16	-1.13
ENSMUSG00000056429	Tgoln1	trans-golgi network protein	-1.14
ENSMUSG00000063952	Brpf3	bromodomain and PHD finger containing, 3	-1.14
ENSMUSG00000002250	Ppard	peroxisome proliferator activator receptor delta	-1.14
ENSMUSG00000039193	Nlrc4	NLR family, CARD domain containing 4	-1.14
ENSMUSG00000036138	Acaa1a	acetyl-Coenzyme A acyltransferase 1A	-1.14
ENSMUSG00000035877	Zhx3	zinc fingers and homeoboxes 3	-1.14
ENSMUSG00000020272	Stk10	serine/threonine kinase 10	-1.14
ENSMUSG00000058022	Adtrp	androgen dependent TFPI regulating protein	-1.14
ENSMUSG00000037279	Ovol2	ovo like zinc finger 2	-1.14
ENSMUSG00000038777	Sema6c	sema domain, transmembrane domain (TM), and cytoplasmic domain, (semaphorin) 6C	-1.14
ENSMUSG00000104114	Gm37297	predicted gene, 37297	-1.14
ENSMUSG00000031971	Ccsap	centriole, cilia and spindle associated protein	-1.14
ENSMUSG00000019370	Calm3	calmodulin 3	-1.15
ENSMUSG00000024378	Stard4	StAR-related lipid transfer (START) domain containing 4	-1.15
ENSMUSG00000029032	Arhgef16	Rho guanine nucleotide exchange factor (GEF) 16	-1.15
ENSMUSG00000028217	Cdh17	cadherin 17	-1.15
ENSMUSG00000021457	Syk	spleen tyrosine kinase	-1.15
ENSMUSG00000022265	Ank	progressive ankylosis	-1.15
ENSMUSG00000025648	Pfkfb4	6-phosphofructo-2-kinase/fructose-2,6-biphosphatase 4	-1.15
ENSMUSG00000039395	Mreg	melanoregulin	-1.15
ENSMUSG00000028709	Mob3c	MOB kinase activator 3C	-1.15
ENSMUSG00000085333	1700030A11Rik	RIKEN cDNA 1700030A11 gene	-1.15
ENSMUSG00000032083	Apoa1	apolipoprotein A-I	-1.15
ENSMUSG00000056025	Clca3a1	chloride channel accessory 3A1	-1.15
ENSMUSG00000030769	Slc5a11	solute carrier family 5 (sodium/glucose cotransporter), member 11	-1.15
ENSMUSG00000025575	Cant1	calcium activated nucleotidase 1	-1.16
ENSMUSG00000019982	Myb	myeloblastosis oncogene	-1.16
ENSMUSG00000028088	Fmo5	flavin containing monooxygenase 5	-1.16
ENSMUSG00000040631	Dok4	docking protein 4	-1.16
ENSMUSG00000026614	Slc30a10	solute carrier family 30, member 10	-1.16
ENSMUSG00000037868	Egr2	early growth response 2	-1.16
ENSMUSG00000087691	Gm15674	predicted gene 15674	-1.16
ENSMUSG00000020009	Ifngr1	interferon gamma receptor 1	-1.17
ENSMUSG00000006651	Aplp1	amyloid beta (A4) precursor-like protein 1	-1.17
ENSMUSG00000063931	Pepd	peptidase D	-1.17
ENSMUSG00000042659	Arrdc4	arrestin domain containing 4	-1.17
ENSMUSG00000045314	Sowahb	sosondowah ankyrin repeat domain family member B	-1.17
ENSMUSG00000047409	Ctdspl	CTD (carboxy-terminal domain, RNA polymerase II, polypeptide A) small phosphatase-like	-1.17
ENSMUSG00000075420	Smim6	small integral membrane protein 6	-1.17
ENSMUSG00000047557	Lxn	latexin	-1.17
ENSMUSG00000057315	Arhgap24	Rho GTPase activating protein 24	-1.17
ENSMUSG00000040740	Slc25a34	solute carrier family 25, member 34	-1.17
ENSMUSG00000035852	Misp	mitotic spindle positioning	-1.18
ENSMUSG00000026090	2010300C02Rik	RIKEN cDNA 2010300C02 gene	-1.18
ENSMUSG00000032306	Mpi	mannose phosphate isomerase	-1.18
ENSMUSG00000025190	Got1	glutamic-oxaloacetic transaminase 1, soluble	-1.18
ENSMUSG00000033083	Tbc1d4	TBC1 domain family, member 4	-1.18
ENSMUSG00000017868	Sgk2	serum/glucocorticoid regulated kinase 2	-1.18
ENSMUSG00000001901	Kcnh6	potassium voltage-gated channel, subfamily H (eag-related), member 6	-1.18
ENSMUSG00000020841	Cpd	carboxypeptidase D	-1.19
ENSMUSG00000015846	Rxra	retinoid X receptor alpha	-1.19
ENSMUSG00000034248	Slc25a37	solute carrier family 25, member 37	-1.19
ENSMUSG00000076435	Acsf2	acyl-CoA synthetase family member 2	-1.19
ENSMUSG00000007833	Aldh16a1	aldehyde dehydrogenase 16 family, member A1	-1.19
ENSMUSG00000033917	Gde1	glycerophosphodiester phosphodiesterase 1	-1.19
ENSMUSG00000031378	Abcd1	ATP-binding cassette, sub-family D (ALD), member 1	-1.19
ENSMUSG00000043445	Pgp	phosphoglycolate phosphatase	-1.19
ENSMUSG00000067297	Ifit1bl2	interferon induced protein with tetratricopeptide repeats 1B like 2	-1.19
ENSMUSG00000029618	Ocm	oncomodulin	-1.19
ENSMUSG00000079174	Gm3054	predicted gene 3054	-1.19
ENSMUSG00000028399	Ptprd	protein tyrosine phosphatase, receptor type, D	-1.20
ENSMUSG00000027957	Slc35a3	solute carrier family 35 (UDP-N-acetylglucosamine (UDP-GlcNAc) transporter), member 3	-1.20
ENSMUSG00000084883	Ccdc85c	coiled-coil domain containing 85C	-1.20
ENSMUSG00000005615	Pcyt1a	phosphate cytidylyltransferase 1, choline, alpha isoform	-1.20
ENSMUSG00000025477	Inpp5a	inositol polyphosphate-5-phosphatase A	-1.20
ENSMUSG00000031156	Slc35a2	solute carrier family 35 (UDP-galactose transporter), member A2	-1.20
ENSMUSG00000016495	Plgrkt	plasminogen receptor, C-terminal lysine transmembrane protein	-1.20
ENSMUSG00000028488	Sh3gl2	SH3-domain GRB2-like 2	-1.20
ENSMUSG00000029471	Camkk2	calcium/calmodulin-dependent protein kinase kinase 2, beta	-1.20
ENSMUSG00000056185	Snx32	sorting nexin 32	-1.20

ENSMUSG00000010122	Slc47a1	solute carrier family 47, member 1	-1.20
ENSMUSG00000025762	Larp1b	La ribonucleoprotein domain family, member 1B	-1.21
ENSMUSG00000024036	Slc37a1	solute carrier family 37 (glycerol-3-phosphate transporter), member 1	-1.21
ENSMUSG00000032802	Srxn1	sulfiredoxin 1 homolog (S. cerevisiae)	-1.21
ENSMUSG00000026062	Slc9a2	solute carrier family 9 (sodium/hydrogen exchanger), member 2	-1.21
ENSMUSG00000033885	Pxk	PX domain containing serine/threonine kinase	-1.21
ENSMUSG00000054499	Dedd2	death effector domain-containing DNA binding protein 2	-1.21
ENSMUSG00000053898	Ech1	enoyl coenzyme A hydratase 1, peroxisomal	-1.21
ENSMUSG00000026922	Agpat2	1-acylglycerol-3-phosphate O-acyltransferase 2 (lysophosphatidic acid acyltransferase, beta)	-1.21
ENSMUSG00000026102	Inpp1	inositol polyphosphate-1-phosphatase	-1.21
ENSMUSG00000102953	Gm37019	predicted gene, 37019	-1.21
ENSMUSG00000040276	Pacsin1	protein kinase C and casein kinase substrate in neurons 1	-1.21
ENSMUSG00000003948	Mmd	monocyte to macrophage differentiation-associated	-1.21
ENSMUSG00000025359	Pmel	premelanosome protein	-1.21
ENSMUSG00000026879	Gsn	gelsolin	-1.22
ENSMUSG00000009828	Ick	intestinal cell kinase	-1.22
ENSMUSG00000031827	Cotl1	coactosin-like 1 (Dictyostelium)	-1.22
ENSMUSG00000028555	Ttc39a	tetratricopeptide repeat domain 39A	-1.22
ENSMUSG00000032661	Oas3	2'-5' oligoadenylate synthetase 3	-1.22
ENSMUSG00000040147	Maob	monoamine oxidase B	-1.22
ENSMUSG00000087535	Zmiz1os1	Zmiz1 opposite strand 1	-1.22
ENSMUSG00000023861	Mpc1	mitochondrial pyruvate carrier 1	-1.22
ENSMUSG00000067813	Xkr9	X-linked Kx blood group related 9	-1.22
ENSMUSG00000034271	Jdp2	Jun dimerization protein 2	-1.22
ENSMUSG00000034330	Plcg2	phospholipase C, gamma 2	-1.23
ENSMUSG00000000538	Tom1l2	target of myb1-like 2 (chicken)	-1.23
ENSMUSG00000038894	Irs2	insulin receptor substrate 2	-1.23
ENSMUSG00000039197	Adk	adenosine kinase	-1.23
ENSMUSG00000034522	Zfp395	zinc finger protein 395	-1.23
ENSMUSG00000042198	Chchd7	coiled-coil-helix-coiled-coil-helix domain containing 7	-1.23
ENSMUSG00000042097	Zfp239	zinc finger protein 239	-1.23
ENSMUSG00000024610	Cd74	CD74 antigen (invariant polypeptide of major histocompatibility complex, class II antigen-associated	-1.24
ENSMUSG00000026303	Mlph	melanophilin	-1.24
ENSMUSG00000020111	Micu1	mitochondrial calcium uptake 1	-1.24
ENSMUSG00000036452	Arhgap26	Rho GTPase activating protein 26	-1.24
ENSMUSG00000029561	Oasl2	2'-5' oligoadenylate synthetase-like 2	-1.24
ENSMUSG00000059323	Tonsl	tonsoku-like, DNA repair protein	-1.24
ENSMUSG00000031337	Mtm1	X-linked myotubular myopathy gene 1	-1.24
ENSMUSG00000041143	Tmco4	transmembrane and coiled-coil domains 4	-1.24
ENSMUSG00000031021	Tmem9b	TMEM9 domain family, member B	-1.24
ENSMUSG00000035967	Ints6l	integrator complex subunit 6 like	-1.24
ENSMUSG00000064210	Ano6	anoctamin 6	-1.25
ENSMUSG00000038260	Trpm4	transient receptor potential cation channel, subfamily M, member 4	-1.25
ENSMUSG00000020099	Unc5b	unc-5 netrin receptor B	-1.25
ENSMUSG00000014418	Hps5	HPS5, biogenesis of lysosomal organelles complex 2 subunit 2	-1.25
ENSMUSG00000035722	Abca7	ATP-binding cassette, sub-family A (ABC1), member 7	-1.25
ENSMUSG00000042228	Lyn	LYN proto-oncogene, Src family tyrosine kinase	-1.25
ENSMUSG00000028293	Slc35a1	solute carrier family 35 (CMP-sialic acid transporter), member 1	-1.25
ENSMUSG00000063838	Cdc42ep5	CDC42 effector protein (Rho GTPase binding) 5	-1.25
ENSMUSG00000026259	Ngef	neuronal guanine nucleotide exchange factor	-1.25
ENSMUSG00000031570	Plpp5	phospholipid phosphatase 5	-1.25
ENSMUSG00000110277	Gm45871	predicted gene 45871	-1.25
ENSMUSG00000020377	Ltc4s	leukotriene C4 synthase	-1.25
ENSMUSG00000061601	Pclo	piccolo (presynaptic cytomatrix protein)	-1.25
ENSMUSG00000018166	Erbb3	erb-b2 receptor tyrosine kinase 3	-1.26
ENSMUSG00000036918	Ttc7	tetratricopeptide repeat domain 7	-1.26
ENSMUSG00000037852	Cpe	carboxypeptidase E	-1.26
ENSMUSG00000028229	Rmdn1	regulator of microtubule dynamics 1	-1.26
ENSMUSG00000103983	Gm20045	predicted gene, 20045	-1.26
ENSMUSG00000020733	Slc9a3r1	solute carrier family 9 (sodium/hydrogen exchanger), member 3 regulator 1	-1.27
ENSMUSG00000022186	Oxct1	3-oxoacid CoA transferase 1	-1.27
ENSMUSG00000028064	Sema4a	sema domain, immunoglobulin domain (Ig), transmembrane domain (TM) and short cytoplasmic do	-1.27
ENSMUSG00000025764	Jade1	jade family PHD finger 1	-1.27
ENSMUSG00000017801	Mlx	MAX-like protein X	-1.27
ENSMUSG00000026304	Rab17	RAB17, member RAS oncogene family	-1.27
ENSMUSG00000035186	Ubd	ubiquitin D	-1.27
ENSMUSG00000026976	Pax8	paired box 8	-1.27
ENSMUSG00000024975	Pdcd4	programmed cell death 4	-1.28
ENSMUSG00000038342	Mlxip	MLX interacting protein	-1.28
ENSMUSG00000031955	Bcar1	breast cancer anti-estrogen resistance 1	-1.28
ENSMUSG00000038365	Fbxo25	F-box protein 25	-1.28
ENSMUSG00000056313	Tcim	transcriptional and immune response regulator	-1.28
ENSMUSG00000006395	Hyi	hydroxypyruvate isomerase (putative)	-1.28
ENSMUSG00000044072	Eml6	echinoderm microtubule associated protein like 6	-1.28
ENSMUSG00000003585	Sec14l2	SEC14-like lipid binding 2	-1.28
ENSMUSG00000104940	Gm42908	predicted gene 42908	-1.28

ENSMUSG00000018554	Ybx2	Y box protein 2	-1.28
ENSMUSG00000031845	Bco1	beta-carotene oxygenase 1	-1.28
ENSMUSG00000100183	Gm28512	predicted gene 28512	-1.28
ENSMUSG00000028479	Gne	glucosamine (UDP-N-acetyl)-2-epimerase/N-acetylmannosamine kinase	-1.29
ENSMUSG00000022844	Pdia5	protein disulfide isomerase associated 5	-1.29
ENSMUSG00000055116	Arntl	aryl hydrocarbon receptor nuclear translocator-like	-1.29
ENSMUSG00000038473	Nos1ap	nitric oxide synthase 1 (neuronal) adaptor protein	-1.29
ENSMUSG00000048970	C1galt1c1	C1GALT1-specific chaperone 1	-1.29
ENSMUSG00000021665	Hexb	hexosaminidase B	-1.29
ENSMUSG00000042747	Krtcap2	keratinocyte associated protein 2	-1.29
ENSMUSG00000086784	Isoc2a	isochorismatase domain containing 2a	-1.29
ENSMUSG00000026204	Ptprn	protein tyrosine phosphatase, receptor type, N	-1.29
ENSMUSG00000005089	Slc1a2	solute carrier family 1 (glial high affinity glutamate transporter), member 2	-1.29
ENSMUSG00000032601	Prkar2a	protein kinase, cAMP dependent regulatory, type II alpha	-1.30
ENSMUSG00000017057	Il13ra1	interleukin 13 receptor, alpha 1	-1.30
ENSMUSG00000030987	Stim1	stromal interaction molecule 1	-1.30
ENSMUSG00000028862	Map3k6	mitogen-activated protein kinase kinase kinase 6	-1.30
ENSMUSG00000054342	Kcnn4	potassium intermediate/small conductance calcium-activated channel, subfamily N, member 4	-1.30
ENSMUSG00000054752	Fsd1l	fibronectin type III and SPRY domain containing 1-like	-1.30
ENSMUSG00000046949	Nqo2	N-ribosyldihydronicotinamide quinone reductase 2	-1.30
ENSMUSG00000106205	C230096K16Rik	RIKEN cDNA C230096K16 gene	-1.30
ENSMUSG00000001227	Sema6b	sema domain, transmembrane domain (TM), and cytoplasmic domain, (semaphorin) 6B	-1.30
ENSMUSG00000013418	B4galnt2	beta-1,4-N-acetyl-galactosaminyl transferase 2	-1.31
ENSMUSG00000027984	Hadh	hydroxyacyl-Coenzyme A dehydrogenase	-1.31
ENSMUSG00000049493	Pls1	plastin 1 (I-isoform)	-1.31
ENSMUSG00000005125	Ndrg1	N-myc downstream regulated gene 1	-1.31
ENSMUSG00000024937	Ehbp1l1	EH domain binding protein 1-like 1	-1.31
ENSMUSG00000041827	Oasl1	2'-5' oligoadenylate synthetase-like 1	-1.31
ENSMUSG00000034591	Slc41a2	solute carrier family 41, member 2	-1.31
ENSMUSG00000026880	Stom	stomatin	-1.31
ENSMUSG00000041120	Nbl1	neuroblastoma, suppression of tumorigenicity 1	-1.31
ENSMUSG00000039304	Tnfsf10	tumor necrosis factor (ligand) superfamily, member 10	-1.31
ENSMUSG00000020447	Npc1l1	NPC1 like intracellular cholesterol transporter 1	-1.31
ENSMUSG00000064177	Ghrl	ghrelin	-1.31
ENSMUSG00000019122	Ccl9	chemokine (C-C motif) ligand 9	-1.31
ENSMUSG00000033577	Myo6	myosin VI	-1.32
ENSMUSG00000060012	Kif13b	kinesin family member 13B	-1.32
ENSMUSG00000033684	Qsox1	quiescin Q6 sulfhydryl oxidase 1	-1.32
ENSMUSG00000024431	Nr3c1	nuclear receptor subfamily 3, group C, member 1	-1.32
ENSMUSG00000061845	Defa35	defensin, alpha, 35	-1.32
ENSMUSG00000026749	Nek6	NIMA (never in mitosis gene a)-related expressed kinase 6	-1.32
ENSMUSG00000047604	Frat2	frequently rearranged in advanced T cell lymphomas 2	-1.32
ENSMUSG00000022683	Pla2g10	phospholipase A2, group X	-1.32
ENSMUSG00000024395	Lims2	LIM and senescent cell antigen like domains 2	-1.32
ENSMUSG00000070574	2310016G11Rik	RIKEN cDNA 2310016G11 gene	-1.32
ENSMUSG00000052738	Suclg1	succinate-CoA ligase, GDP-forming, alpha subunit	-1.33
ENSMUSG00000005580	Adcy9	adenylate cyclase 9	-1.33
ENSMUSG00000031887	Tradd	TNFRSF1A-associated via death domain	-1.33
ENSMUSG00000030761	Myo7a	myosin VIIA	-1.33
ENSMUSG00000029859	Epha1	Eph receptor A1	-1.33
ENSMUSG00000020609	Apob	apolipoprotein B	-1.33
ENSMUSG00000093880	Tmem181c-ps	transmembrane protein 181C, pseudogene	-1.33
ENSMUSG00000035441	Myo1d	myosin ID	-1.34
ENSMUSG00000039611	Tmem246	transmembrane protein 246	-1.34
ENSMUSG00000042797	Aqp11	aquaporin 11	-1.34
ENSMUSG00000079429	Mroh2a	maestro heat-like repeat family member 2A	-1.34
ENSMUSG00000042737	Dpm3	dolichyl-phosphate mannosyltransferase polypeptide 3	-1.34
ENSMUSG00000097006	9530082P21Rik	RIKEN cDNA 9530082P21 gene	-1.34
ENSMUSG00000022946	Dopey2	dopey family member 2	-1.35
ENSMUSG00000042808	Gpx2	glutathione peroxidase 2	-1.35
ENSMUSG00000039234	Sec24d	Sec24 related gene family, member D (S. cerevisiae)	-1.35
ENSMUSG00000038244	Mical2	microtubule associated monooxygenase, calponin and LIM domain containing 2	-1.35
ENSMUSG00000020826	Nos2	nitric oxide synthase 2, inducible	-1.35
ENSMUSG00000025429	Pstpip2	proline-serine-threonine phosphatase-interacting protein 2	-1.35
ENSMUSG00000038967	Pdk2	pyruvate dehydrogenase kinase, isoenzyme 2	-1.35
ENSMUSG00000022750	Klhl22	kelch-like 22	-1.35
ENSMUSG00000037709	Fam13a	family with sequence similarity 13, member A	-1.35
ENSMUSG00000074882	Cyp2c68	cytochrome P450, family 2, subfamily c, polypeptide 68	-1.35
ENSMUSG00000032766	Gng11	guanine nucleotide binding protein (G protein), gamma 11	-1.35
ENSMUSG00000028719	Cmpk1	cytidine monophosphate (UMP-CMP) kinase 1	-1.36
ENSMUSG00000033318	Gstt2	glutathione S-transferase, theta 2	-1.36
ENSMUSG00000030935	Acsm3	acyl-CoA synthetase medium-chain family member 3	-1.36
ENSMUSG00000074218	Cox7a1	cytochrome c oxidase subunit 7A1	-1.36
ENSMUSG00000020827	Mink1	misshapen-like kinase 1 (zebrafish)	-1.37
ENSMUSG00000009647	Mcu	mitochondrial calcium uniporter	-1.37
ENSMUSG00000028334	Nans	N-acetylneuraminic acid synthase (sialic acid synthase)	-1.37

ENSMUSG00000020752	Recql5	RecQ protein-like 5	-1.37
ENSMUSG00000059316	Slc27a4	solute carrier family 27 (fatty acid transporter), member 4	-1.37
ENSMUSG00000026896	Ifih1	interferon induced with helicase C domain 1	-1.37
ENSMUSG00000047496	Rnf152	ring finger protein 152	-1.37
ENSMUSG00000027412	Lpin3	lipin 3	-1.37
ENSMUSG00000004815	Dgkq	diacylglycerol kinase, theta	-1.37
ENSMUSG00000072115	Ang	angiogenin, ribonuclease, RNase A family, 5	-1.37
ENSMUSG00000029188	Slc34a2	solute carrier family 34 (sodium phosphate), member 2	-1.37
ENSMUSG00000029802	Abcg2	ATP binding cassette subfamily G member 2 (Junior blood group)	-1.37
ENSMUSG00000051339	2900026A02Rik	RIKEN cDNA 2900026A02 gene	-1.38
ENSMUSG00000039131	Gipc2	GIPC PDZ domain containing family, member 2	-1.38
ENSMUSG00000033416	Gucd1	guanylyl cyclase domain containing 1	-1.38
ENSMUSG00000036499	Eea1	early endosome antigen 1	-1.38
ENSMUSG00000024867	Pip5k1b	phosphatidylinositol-4-phosphate 5-kinase, type 1 beta	-1.38
ENSMUSG00000031823	Zdhhc7	zinc finger, DHHC domain containing 7	-1.38
ENSMUSG00000034371	Tkfc	triokinase, FMN cyclase	-1.38
ENSMUSG00000026961	Lrrc26	leucine rich repeat containing 26	-1.38
ENSMUSG00000001670	Tat	tyrosine aminotransferase	-1.38
ENSMUSG00000067199	Frat1	frequently rearranged in advanced T cell lymphomas	-1.38
ENSMUSG00000035769	Xylb	xylulokinase homolog (H. influenzae)	-1.38
ENSMUSG00000102336	Gm37233	predicted gene, 37233	-1.38
ENSMUSG00000106659	Gm42161	predicted gene, 42161	-1.38
ENSMUSG00000044231	Nhlrc1	NHL repeat containing 1	-1.38
ENSMUSG00000045019	Acer1	alkaline ceramidase 1	-1.38
ENSMUSG00000061751	Kalrn	kalirin, RhoGEF kinase	-1.39
ENSMUSG00000043079	Synpo	synaptopodin	-1.39
ENSMUSG00000050931	Sgms2	sphingomyelin synthase 2	-1.39
ENSMUSG00000022105	Rb1	RB transcriptional corepressor 1	-1.39
ENSMUSG00000029727	Cyp3a13	cytochrome P450, family 3, subfamily a, polypeptide 13	-1.39
ENSMUSG00000021185	Dglucy	D-glutamate cyclase	-1.39
ENSMUSG00000036863	Syde2	synapse defective 1, Rho GTPase, homolog 2 (C. elegans)	-1.39
ENSMUSG00000029780	Nt5c3	5'-nucleotidase, cytosolic III	-1.39
ENSMUSG00000108291	Gm44292	predicted gene, 44292	-1.39
ENSMUSG00000027457	Snph	syntaphilin	-1.39
ENSMUSG00000029452	Tmem116	transmembrane protein 116	-1.39
ENSMUSG00000025068	Gsto1	glutathione S-transferase omega 1	-1.40
ENSMUSG00000003746	Man1a	mannosidase 1, alpha	-1.40
ENSMUSG00000074443	Defa22	defensin, alpha, 22	-1.40
ENSMUSG00000046519	Golph3l	golgi phosphoprotein 3-like	-1.40
ENSMUSG00000067219	Nipal1	NIPA-like domain containing 1	-1.40
ENSMUSG00000024776	Stambpl1	STAM binding protein like 1	-1.40
ENSMUSG00000036106	Prr5	proline rich 5 (renal)	-1.40
ENSMUSG00000039699	Batf2	basic leucine zipper transcription factor, ATF-like 2	-1.40
ENSMUSG00000028518	Prkaa2	protein kinase, AMP-activated, alpha 2 catalytic subunit	-1.41
ENSMUSG00000024074	Crim1	cysteine rich transmembrane BMP regulator 1 (chordin like)	-1.41
ENSMUSG00000025993	Slc40a1	solute carrier family 40 (iron-regulated transporter), member 1	-1.41
ENSMUSG00000038240	Pdss2	prenyl (solanesyl) diphosphate synthase, subunit 2	-1.41
ENSMUSG00000022040	Ephx2	epoxide hydrolase 2, cytoplasmic	-1.41
ENSMUSG00000030428	Ttyh1	tweety family member 1	-1.41
ENSMUSG00000032737	Inppl1	inositol polyphosphate phosphatase-like 1	-1.42
ENSMUSG00000024955	Esrra	estrogen related receptor, alpha	-1.42
ENSMUSG00000003134	Tbc1d8	TBC1 domain family, member 8	-1.42
ENSMUSG00000036136	Fam110c	family with sequence similarity 110, member C	-1.42
ENSMUSG00000058586	Serhl	serine hydrolase-like	-1.42
ENSMUSG00000026018	Ica1l	islet cell autoantigen 1-like	-1.42
ENSMUSG00000024644	Cndp2	CNDP dipeptidase 2 (metallopeptidase M20 family)	-1.43
ENSMUSG00000047153	Khnyn	KH and NYN domain containing	-1.43
ENSMUSG00000022330	Osr2	odd-skipped related 2	-1.43
ENSMUSG00000028121	Bcar3	breast cancer anti-estrogen resistance 3	-1.43
ENSMUSG00000037921	Ddx60	DEAD (Asp-Glu-Ala-Asp) box polypeptide 60	-1.43
ENSMUSG00000022687	Boc	biregional cell adhesion molecule-related/down-regulated by oncogenes (Cdon) binding protein	-1.43
ENSMUSG00000040350	Trim7	tripartite motif-containing 7	-1.43
ENSMUSG00000104737	Gm42937	predicted gene 42937	-1.43
ENSMUSG00000043155	Hpdl	4-hydroxyphenylpyruvate dioxygenase-like	-1.43
ENSMUSG00000106191	4930557B06Rik	RIKEN cDNA 4930557B06 gene	-1.43
ENSMUSG00000068341	Reg3d	regenerating islet-derived 3 delta	-1.43
ENSMUSG00000001156	Mxd1	MAX dimerization protein 1	-1.44
ENSMUSG00000015533	Itga2	integrin alpha 2	-1.44
ENSMUSG00000034066	Farp2	FERM, RhoGEF and pleckstrin domain protein 2	-1.44
ENSMUSG00000026360	Rgs2	regulator of G-protein signaling 2	-1.44
ENSMUSG00000013539	Tango2	transport and golgi organization 2	-1.44
ENSMUSG00000086894	Gm15708	predicted gene 15708	-1.44
ENSMUSG00000046861	Hectd3	HECT domain E3 ubiquitin protein ligase 3	-1.45
ENSMUSG00000031090	Nadsyn1	NAD synthetase 1	-1.45
ENSMUSG00000021708	Rasgrf2	RAS protein-specific guanine nucleotide-releasing factor 2	-1.45
ENSMUSG00000067931	Zfp948	zinc finger protein 948	-1.45

ENSMUSG00000004610	Etfb	electron transferring flavoprotein, beta polypeptide	-1.45
ENSMUSG00000043629	1700019D03Rik	RIKEN cDNA 1700019D03 gene	-1.45
ENSMUSG00000056035	Cyp3a11	cytochrome P450, family 3, subfamily a, polypeptide 11	-1.45
ENSMUSG00000085088	4931413K12Rik	RIKEN cDNA 4931413K12 gene	-1.45
ENSMUSG00000053819	Camk2d	calcium/calmodulin-dependent protein kinase II, delta	-1.46
ENSMUSG00000021556	Golm1	golgi membrane protein 1	-1.46
ENSMUSG00000022949	Clic6	chloride intracellular channel 6	-1.46
ENSMUSG00000039637	Coro7	coronin 7	-1.46
ENSMUSG00000028538	St3gal3	ST3 beta-galactoside alpha-2,3-sialyltransferase 3	-1.46
ENSMUSG00000097059	Fam120aos	family with sequence similarity 120A, opposite strand	-1.46
ENSMUSG00000023206	Il15ra	interleukin 15 receptor, alpha chain	-1.46
ENSMUSG00000054150	Syne3	spectrin repeat containing, nuclear envelope family member 3	-1.46
ENSMUSG00000023057	Fabp2	fatty acid binding protein 2, intestinal	-1.46
ENSMUSG00000030681	Mvp	major vault protein	-1.47
ENSMUSG00000003546	Klc4	kinesin light chain 4	-1.47
ENSMUSG00000025498	Irf7	interferon regulatory factor 7	-1.47
ENSMUSG00000035473	Galm	galactose mutarotase	-1.47
ENSMUSG00000028480	Glipr2	GLI pathogenesis-related 2	-1.47
ENSMUSG00000025198	Erlin1	ER lipid raft associated 1	-1.47
ENSMUSG00000014778	Fhod1	formin homology 2 domain containing 1	-1.47
ENSMUSG00000042333	Tnfrsf14	tumor necrosis factor receptor superfamily, member 14 (herpesvirus entry mediator)	-1.47
ENSMUSG00000028158	Mttp	microsomal triglyceride transfer protein	-1.47
ENSMUSG00000108526	Gm45828	predicted gene 45828	-1.47
ENSMUSG00000025978	Rftn2	raftlin family member 2	-1.47
ENSMUSG00000029163	Emilin1	elastin microfibril interfacer 1	-1.47
ENSMUSG00000045817	Zfp36l2	zinc finger protein 36, C3H type-like 2	-1.48
ENSMUSG00000028943	Espn	espin	-1.48
ENSMUSG00000039166	Akap7	A kinase (PRKA) anchor protein 7	-1.48
ENSMUSG00000032842	Abcc10	ATP-binding cassette, sub-family C (CFTR/MRP), member 10	-1.48
ENSMUSG00000068874	Selenbp1	selenium binding protein 1	-1.48
ENSMUSG00000015405	Ace2	angiotensin I converting enzyme (peptidyl-dipeptidase A) 2	-1.48
ENSMUSG00000042367	Gjb3	gap junction protein, beta 3	-1.48
ENSMUSG00000052135	Foxo6	forkhead box O6	-1.48
ENSMUSG00000112346	Gm48768	predicted gene, 48768	-1.48
ENSMUSG00000039278	Pcsk1n	proprotein convertase subtilisin/kexin type 1 inhibitor	-1.48
ENSMUSG00000050395	Tnfsf15	tumor necrosis factor (ligand) superfamily, member 15	-1.48
ENSMUSG00000054733	Msra	methionine sulfoxide reductase A	-1.49
ENSMUSG00000037455	Slc18b1	solute carrier family 18, subfamily B, member 1	-1.49
ENSMUSG00000068417	Pnp2	purine-nucleoside phosphorylase 2	-1.49
ENSMUSG00000021280	Exoc3l4	exocyst complex component 3-like 4	-1.49
ENSMUSG00000063652	Slc22a21	solute carrier family 22 (organic cation transporter), member 21	-1.49
ENSMUSG00000072571	Tmem253	transmembrane protein 253	-1.49
ENSMUSG00000054200	Ffar4	free fatty acid receptor 4	-1.49
ENSMUSG00000037169	Mycn	v-myc avian myelocytomatosis viral related oncogene, neuroblastoma derived	-1.49
ENSMUSG00000087651	1500009L16Rik	RIKEN cDNA 1500009L16 gene	-1.49
ENSMUSG00000000915	Hip1r	huntingtin interacting protein 1 related	-1.50
ENSMUSG00000029646	Cdx2	caudal type homeobox 2	-1.50
ENSMUSG00000055675	Kbtbd11	kelch repeat and BTB (POZ) domain containing 11	-1.50
ENSMUSG00000021587	Pcsk1	proprotein convertase subtilisin/kexin type 1	-1.50
ENSMUSG00000030545	Pex11a	peroxisomal biogenesis factor 11 alpha	-1.50
ENSMUSG00000104693	Gm42941	predicted gene 42941	-1.50
ENSMUSG00000017718	Afmid	arylformamidase	-1.50
ENSMUSG00000041617	Ccdc74a	coiled-coil domain containing 74A	-1.50
ENSMUSG00000086189	Gm15462	predicted gene 15462	-1.50
ENSMUSG00000024066	Xdh	xanthine dehydrogenase	-1.51
ENSMUSG00000024712	Rfk	riboflavin kinase	-1.51
ENSMUSG00000031482	Slc25a15	solute carrier family 25 (mitochondrial carrier ornithine transporter), member 15	-1.51
ENSMUSG00000041237	Pklr	pyruvate kinase liver and red blood cell	-1.51
ENSMUSG00000024727	Trpm6	transient receptor potential cation channel, subfamily M, member 6	-1.51
ENSMUSG00000042073	Abhd14b	abhydrolase domain containing 14b	-1.51
ENSMUSG00000098678	Mroh6	maestro heat-like repeat family member 6	-1.51
ENSMUSG00000039653	Baat	bile acid-Coenzyme A: amino acid N-acyltransferase	-1.51
ENSMUSG00000022836	Mylk	myosin, light polypeptide kinase	-1.52
ENSMUSG00000061666	Gdpd1	glycerophosphodiester phosphodiesterase domain containing 1	-1.52
ENSMUSG00000020681	Ace	angiotensin I converting enzyme (peptidyl-dipeptidase A) 1	-1.52
ENSMUSG00000033826	Dnah8	dynein, axonemal, heavy chain 8	-1.52
ENSMUSG00000021286	Zfyve21	zinc finger, FYVE domain containing 21	-1.52
ENSMUSG00000028795	Ccdc28b	coiled coil domain containing 28B	-1.52
ENSMUSG00000032380	Dapk2	death-associated protein kinase 2	-1.52
ENSMUSG00000023393	Slc17a9	solute carrier family 17, member 9	-1.52
ENSMUSG00000038058	Nod1	nucleotide-binding oligomerization domain containing 1	-1.52
ENSMUSG00000082148	Gm12266	predicted gene 12266	-1.52
ENSMUSG00000087611	4930458D05Rik	RIKEN cDNA 4930458D05 gene	-1.52
ENSMUSG00000029102	Hgfac	hepatocyte growth factor activator	-1.53
ENSMUSG00000020573	Pik3cg	phosphatidylinositol-4,5-bisphosphate 3-kinase catalytic subunit gamma	-1.53
ENSMUSG00000075010	AW112010	expressed sequence AW112010	-1.53

ENSMUSG00000073402	Gm8909	predicted gene 8909	-1.53
ENSMUSG00000017453	Pipox	pipecolic acid oxidase	-1.53
ENSMUSG00000029923	Rab19	RAB19, member RAS oncogene family	-1.53
ENSMUSG00000069227	Gprin1	G protein-regulated inducer of neurite outgrowth 1	-1.53
ENSMUSG00000097924	A730020E08Rik	RIKEN cDNA A730020E08 gene	-1.53
ENSMUSG00000074628	Tldc2	TBC/LysM associated domain containing 2	-1.53
ENSMUSG00000041757	Plekha6	pleckstrin homology domain containing, family A member 6	-1.54
ENSMUSG00000023073	Slc10a2	solute carrier family 10, member 2	-1.54
ENSMUSG00000030155	Clec2e	C-type lectin domain family 2, member e	-1.54
ENSMUSG00000052102	Gnpda1	glucosamine-6-phosphate deaminase 1	-1.54
ENSMUSG00000029413	Naaa	N-acylethanolamine acid amidase	-1.54
ENSMUSG00000027765	P2ry1	purinergic receptor P2Y, G-protein coupled 1	-1.54
ENSMUSG00000052271	Bhlha15	basic helix-loop-helix family, member a15	-1.54
ENSMUSG00000051065	Mb21d2	Mab-21 domain containing 2	-1.54
ENSMUSG00000097354	2310001H17Rik	RIKEN cDNA 2310001H17 gene	-1.54
ENSMUSG00000080715	Gm5406	predicted gene 5406	-1.54
ENSMUSG00000105586	Gm43009	predicted gene 43009	-1.54
ENSMUSG00000016206	H2-M3	histocompatibility 2, M region locus 3	-1.54
ENSMUSG00000032243	Itga11	integrin alpha 11	-1.54
ENSMUSG00000055197	Fev	FEV (ETS oncogene family)	-1.54
ENSMUSG00000034781	Gna11	guanine nucleotide binding protein, alpha 11	-1.55
ENSMUSG00000036813	Entpd8	ectonucleoside triphosphate diphosphohydrolase 8	-1.55
ENSMUSG00000104535	Gm42686	predicted gene 42686	-1.55
ENSMUSG00000022711	Pmm2	phosphomannomutase 2	-1.56
ENSMUSG00000024187	Fam234a	family with sequence similarity 234, member A	-1.56
ENSMUSG00000026687	Aldh9a1	aldehyde dehydrogenase 9, subfamily A1	-1.56
ENSMUSG00000028223	Decr1	2,4-dienoyl CoA reductase 1, mitochondrial	-1.56
ENSMUSG00000027314	Dll4	delta like canonical Notch ligand 4	-1.56
ENSMUSG00000039208	Metrnl	meteorin, glial cell differentiation regulator-like	-1.56
ENSMUSG00000044080	S100a1	S100 calcium binding protein A1	-1.56
ENSMUSG00000026189	Pecr	peroxisomal trans-2-enoyl-CoA reductase	-1.56
ENSMUSG00000074653	Lrrc31	leucine rich repeat containing 31	-1.56
ENSMUSG00000011034	Slc5a1	solute carrier family 5 (sodium/glucose cotransporter), member 1	-1.56
ENSMUSG00000025584	Pde8a	phosphodiesterase 8A	-1.56
ENSMUSG00000042258	Isl1	ISL1 transcription factor, LIM/homeodomain	-1.56
ENSMUSG00000055670	Zzef1	zinc finger, ZZ-type with EF hand domain 1	-1.57
ENSMUSG00000022680	Pdxdc1	pyridoxal-dependent decarboxylase domain containing 1	-1.57
ENSMUSG00000116594	AC133488.1	novel protein	-1.57
ENSMUSG00000026322	Htr4	5 hydroxytryptamine (serotonin) receptor 4	-1.57
ENSMUSG00000070331	Qrich2	glutamine rich 2	-1.57
ENSMUSG00000108850	Gm44914	predicted gene 44914	-1.57
ENSMUSG00000048992	Prss32	protease, serine 32	-1.58
ENSMUSG00000020451	Limk2	LIM motif-containing protein kinase 2	-1.58
ENSMUSG00000024063	Lbh	limb-bud and heart	-1.58
ENSMUSG00000001348	Acp5	acid phosphatase 5, tartrate resistant	-1.58
ENSMUSG00000070563	Spaca4	sperm acrosome associated 4	-1.58
ENSMUSG00000039145	Camk1d	calcium/calmodulin-dependent protein kinase ID	-1.59
ENSMUSG00000029053	Prkcz	protein kinase C, zeta	-1.59
ENSMUSG00000020019	Ntn4	netrin 4	-1.59
ENSMUSG00000041372	B4galnt3	beta-1,4-N-acetyl-galactosaminyl transferase 3	-1.59
ENSMUSG00000034320	Slc26a2	solute carrier family 26 (sulfate transporter), member 2	-1.60
ENSMUSG00000054662	Ano9	anoctamin 9	-1.60
ENSMUSG00000007034	Slc44a4	solute carrier family 44, member 4	-1.60
ENSMUSG00000041737	Tmem45b	transmembrane protein 45b	-1.60
ENSMUSG00000027938	Creb3l4	cAMP responsive element binding protein 3-like 4	-1.60
ENSMUSG00000055866	Per2	period circadian clock 2	-1.60
ENSMUSG00000043333	Rhbdl2	rhomboid like 2	-1.60
ENSMUSG00000071604	Fam189a2	family with sequence similarity 189, member A2	-1.60
ENSMUSG00000036553	Sh3tc1	SH3 domain and tetratricopeptide repeats 1	-1.60
ENSMUSG00000036473	Tbc1d24	TBC1 domain family, member 24	-1.60
ENSMUSG00000027834	Serpini1	serine (or cysteine) peptidase inhibitor, clade I, member 1	-1.60
ENSMUSG00000029082	Bst1	bone marrow stromal cell antigen 1	-1.60
ENSMUSG00000111468	5033425B01Rik	RIKEN cDNA 5033425B01 gene	-1.60
ENSMUSG00000013653	1810065E05Rik	RIKEN cDNA 1810065E05 gene	-1.60
ENSMUSG00000049037	Clec4a1	C-type lectin domain family 4, member a1	-1.60
ENSMUSG00000063730	Hsd3b2	hydroxy-delta-5-steroid dehydrogenase, 3 beta- and steroid delta-isomerase 2	-1.60
ENSMUSG00000029381	Shroom3	shroom family member 3	-1.61
ENSMUSG00000040584	Abcb1a	ATP-binding cassette, sub-family B (MDR/TAP), member 1A	-1.61
ENSMUSG00000074445	Sprr2a3	small proline-rich protein 2A3	-1.61
ENSMUSG00000058624	Gda	guanine deaminase	-1.61
ENSMUSG00000022555	Dgat1	diacylglycerol O-acyltransferase 1	-1.61
ENSMUSG00000064373	Selenop	selenoprotein P	-1.61
ENSMUSG00000037649	H2-DMa	histocompatibility 2, class II, locus DMa	-1.61
ENSMUSG00000019762	Iyd	iodotyrosine deiodinase	-1.61
ENSMUSG00000093580	Gm20706	predicted gene 20706	-1.61
ENSMUSG00000071506	Tmem139	transmembrane protein 139	-1.61

ENSMUSG00000036533	Cdc42ep3	CDC42 effector protein (Rho GTPase binding) 3	-1.61
ENSMUSG00000009772	Nuak2	NUAK family, SNF1-like kinase, 2	-1.62
ENSMUSG00000072214	Sept5	septin 5	-1.62
ENSMUSG00000027510	Rbm38	RNA binding motif protein 38	-1.62
ENSMUSG00000020774	Aspa	aspartoacylase	-1.62
ENSMUSG00000022574	Naprt	nicotinate phosphoribosyltransferase	-1.62
ENSMUSG00000054252	Fgfr3	fibroblast growth factor receptor 3	-1.62
ENSMUSG00000005107	Slc2a9	solute carrier family 2 (facilitated glucose transporter), member 9	-1.62
ENSMUSG00000039747	Orai2	ORAI calcium release-activated calcium modulator 2	-1.62
ENSMUSG00000029314	Gpat3	glycerol-3-phosphate acyltransferase 3	-1.62
ENSMUSG00000045679	Pqlc3	PQ loop repeat containing	-1.62
ENSMUSG00000021125	Arg2	arginase type II	-1.62
ENSMUSG00000003849	Nqo1	NAD(P)H dehydrogenase, quinone 1	-1.62
ENSMUSG00000078954	Arhgap8	Rho GTPase activating protein 8	-1.62
ENSMUSG00000050762	Prss27	protease, serine 27	-1.62
ENSMUSG00000040600	Eps8l3	EPS8-like 3	-1.63
ENSMUSG00000027695	Pld1	phospholipase D1	-1.63
ENSMUSG00000000958	Slc7a7	solute carrier family 7 (cationic amino acid transporter, y+ system), member 7	-1.63
ENSMUSG00000021509	Slc25a48	solute carrier family 25, member 48	-1.63
ENSMUSG00000029409	U90926	cDNA sequence U90926	-1.63
ENSMUSG00000021337	Scgn	secretagogin, EF-hand calcium binding protein	-1.63
ENSMUSG00000085944	1700003D09Rik	RIKEN cDNA 1700003D09 gene	-1.63
ENSMUSG00000023909	Paqr4	progestin and adipoQ receptor family member IV	-1.64
ENSMUSG00000064254	Ethe1	ethylmalonic encephalopathy 1	-1.64
ENSMUSG00000033538	Casp4	caspase 4, apoptosis-related cysteine peptidase	-1.64
ENSMUSG00000020847	Rph3al	rabphilin 3A-like (without C2 domains)	-1.64
ENSMUSG00000038811	Gngt2	guanine nucleotide binding protein (G protein), gamma transducing activity polypeptide 2	-1.64
ENSMUSG00000070802	Pnmal2	PNMA-like 2	-1.64
ENSMUSG00000030909	Anks4b	ankyrin repeat and sterile alpha motif domain containing 4B	-1.65
ENSMUSG00000102752	Gm7694	predicted gene 7694	-1.65
ENSMUSG00000051379	Flrt3	fibronectin leucine rich transmembrane protein 3	-1.65
ENSMUSG00000029847	Slc23a4	solute carrier family 23 member 4	-1.65
ENSMUSG00000057069	Ero1lb	ERO1-like beta (S. cerevisiae)	-1.65
ENSMUSG00000025557	Slc15a1	solute carrier family 15 (oligopeptide transporter), member 1	-1.65
ENSMUSG00000104316	Gm37909	predicted gene, 37909	-1.65
ENSMUSG00000085196	Gm14963	predicted gene 14963	-1.65
ENSMUSG00000032500	Dclk3	doublecortin-like kinase 3	-1.65
ENSMUSG00000025357	Dgka	diacylglycerol kinase, alpha	-1.66
ENSMUSG00000021266	Wars	tryptophanyl-tRNA synthetase	-1.66
ENSMUSG00000037579	Kcnh3	potassium voltage-gated channel, subfamily H (eag-related), member 3	-1.66
ENSMUSG00000022219	Cideb	cell death-inducing DNA fragmentation factor, alpha subunit-like effector B	-1.66
ENSMUSG00000115062	4930544F09Rik	RIKEN cDNA 4930544F09 gene	-1.66
ENSMUSG00000086187	Gm12860	predicted gene 12860	-1.66
ENSMUSG00000026175	Vil1	villin 1	-1.67
ENSMUSG00000060733	Ipmk	inositol polyphosphate multikinase	-1.67
ENSMUSG00000040712	Camta2	calmodulin binding transcription activator 2	-1.67
ENSMUSG00000020520	Galnt10	polypeptide N-acetylgalactosaminyltransferase 10	-1.67
ENSMUSG00000027225	Duoxa2	dual oxidase maturation factor 2	-1.67
ENSMUSG00000028519	Dab1	disabled 1	-1.67
ENSMUSG00000057596	Trim30d	tripartite motif-containing 30D	-1.67
ENSMUSG00000039813	Tbc1d2	TBC1 domain family, member 2	-1.67
ENSMUSG00000064262	Gimap8	GTPase, IMAP family member 8	-1.67
ENSMUSG00000085747	Slc13a2os	solute carrier family 13 (sodium-dependent dicarboxylate transporter), member 2, opposite strand	-1.67
ENSMUSG00000022861	Dgkg	diacylglycerol kinase, gamma	-1.67
ENSMUSG00000030499	Kctd15	potassium channel tetramerisation domain containing 15	-1.67
ENSMUSG00000004748	Mtfp1	mitochondrial fission process 1	-1.67
ENSMUSG00000078202	Nrarp	Notch-regulated ankyrin repeat protein	-1.67
ENSMUSG00000108210	Gm35808	predicted gene, 35808	-1.67
ENSMUSG00000048905	4930539E08Rik	RIKEN cDNA 4930539E08 gene	-1.68
ENSMUSG00000024556	Me2	malic enzyme 2, NAD(+)-dependent, mitochondrial	-1.68
ENSMUSG00000031402	Mpp1	membrane protein, palmitoylated	-1.68
ENSMUSG00000050747	Trim15	tripartite motif-containing 15	-1.68
ENSMUSG00000030790	Adm	adrenomedullin	-1.68
ENSMUSG00000029455	Aldh2	aldehyde dehydrogenase 2, mitochondrial	-1.69
ENSMUSG00000038497	Tmco3	transmembrane and coiled-coil domains 3	-1.69
ENSMUSG00000116542	Gm17783	predicted, 17783	-1.69
ENSMUSG00000025467	Prap1	proline-rich acidic protein 1	-1.69
ENSMUSG00000006567	Atp7b	ATPase, Cu++ transporting, beta polypeptide	-1.69
ENSMUSG00000042041	2010003K11Rik	RIKEN cDNA 2010003K11 gene	-1.69
ENSMUSG00000062410	Hsd3b3	hydroxy-delta-5-steroid dehydrogenase, 3 beta- and steroid delta-isomerase 3	-1.69
ENSMUSG00000106206	Gm43094	predicted gene 43094	-1.69
ENSMUSG00000050158	Olfr165	olfactory receptor 165	-1.69
ENSMUSG00000023150	Ivns1abp	influenza virus NS1A binding protein	-1.70
ENSMUSG00000029772	Ahcyl2	S-adenosylhomocysteine hydrolase-like 2	-1.70
ENSMUSG00000034275	Igsf9b	immunoglobulin superfamily, member 9B	-1.70
ENSMUSG00000029401	Rilpl2	Rab interacting lysosomal protein-like 2	-1.70

ENSMUSG00000074063	Osgin1	oxidative stress induced growth inhibitor 1	-1.70
ENSMUSG00000039062	Anpep	alanyl (membrane) aminopeptidase	-1.70
ENSMUSG00000112592	Gm19972	predicted gene, 19972	-1.70
ENSMUSG00000110520	Gm45776	predicted gene 45776	-1.70
ENSMUSG00000062962	Gm6378	predicted pseudogene 6378	-1.70
ENSMUSG00000029470	P2rx4	purinergic receptor P2X, ligand-gated ion channel 4	-1.71
ENSMUSG00000038642	Ctss	cathepsin S	-1.71
ENSMUSG00000045038	Prkce	protein kinase C, epsilon	-1.71
ENSMUSG00000034413	Neurl1b	neuralized E3 ubiquitin protein ligase 1B	-1.71
ENSMUSG00000036594	H2-Aa	histocompatibility 2, class II antigen A, alpha	-1.71
ENSMUSG00000042312	S100a13	S100 calcium binding protein A13	-1.71
ENSMUSG00000037139	Myom3	myomesin family, member 3	-1.71
ENSMUSG00000104350	Gm38244	predicted gene, 38244	-1.71
ENSMUSG00000020405	Fabp6	fatty acid binding protein 6, ileal (gastrotropin)	-1.71
ENSMUSG00000100927	Gm28536	predicted gene 28536	-1.71
ENSMUSG00000022445	Cyp2d26	cytochrome P450, family 2, subfamily d, polypeptide 26	-1.71
ENSMUSG00000057963	Itpk1	inositol 1,3,4-triphosphate 5/6 kinase	-1.72
ENSMUSG00000041605	Inava	innate immunity activator	-1.72
ENSMUSG00000034714	Ttyh2	tweety family member 2	-1.72
ENSMUSG00000023915	Tnfrsf21	tumor necrosis factor receptor superfamily, member 21	-1.72
ENSMUSG00000019970	Sgk1	serum/glucocorticoid regulated kinase 1	-1.72
ENSMUSG00000022180	Slc7a8	solute carrier family 7 (cationic amino acid transporter, y+ system), member 8	-1.72
ENSMUSG00000037157	Il22ra1	interleukin 22 receptor, alpha 1	-1.73
ENSMUSG00000038331	Satb2	special AT-rich sequence binding protein 2	-1.73
ENSMUSG00000097099	Gm9917	predicted gene 9917	-1.73
ENSMUSG00000037390	Muc3	mucin 3, intestinal	-1.73
ENSMUSG00000024386	Proc	protein C	-1.73
ENSMUSG00000106417	Gm42628	predicted gene 42628	-1.73
ENSMUSG00000037605	Adgrl3	adhesion G protein-coupled receptor L3	-1.73
ENSMUSG00000106542	Gm43410	predicted gene 43410	-1.73
ENSMUSG00000019558	Slc6a8	solute carrier family 6 (neurotransmitter transporter, creatine), member 8	-1.74
ENSMUSG00000025076	Casp7	caspase 7	-1.74
ENSMUSG00000039910	Cited2	Cbp/p300-interacting transactivator, with Glu/Asp-rich carboxy-terminal domain, 2	-1.74
ENSMUSG00000037661	Gpr160	G protein-coupled receptor 160	-1.74
ENSMUSG00000038843	Gcnt1	glucosaminyl (N-acetyl) transferase 1, core 2	-1.74
ENSMUSG00000079507	H2-Q1	histocompatibility 2, Q region locus 1	-1.74
ENSMUSG00000022048	Dpysl2	dihydropyrimidinase-like 2	-1.75
ENSMUSG00000039774	Galnt12	polypeptide N-acetylgalactosaminyltransferase 12	-1.75
ENSMUSG00000102802	Mgam2-ps	maltase-glucoamylase 2, pseudogene	-1.75
ENSMUSG00000061411	Nol4l	nucleolar protein 4-like	-1.75
ENSMUSG00000031897	Psmb10	proteasome (prosome, macropain) subunit, beta type 10	-1.75
ENSMUSG00000028194	Ddah1	dimethylarginine dimethylaminohydrolase 1	-1.75
ENSMUSG00000022504	Ciita	class II transactivator	-1.75
ENSMUSG00000028536	2610528J11Rik	RIKEN cDNA 2610528J11 gene	-1.75
ENSMUSG00000009646	Pla2g12b	phospholipase A2, group XIIB	-1.75
ENSMUSG00000054474	Thnsl2	threonine synthase-like 2 (bacterial)	-1.75
ENSMUSG00000015452	Ager	advanced glycosylation end product-specific receptor	-1.75
ENSMUSG00000032536	Trak1	trafficking protein, kinesin binding 1	-1.76
ENSMUSG00000032120	C2cd2l	C2 calcium-dependent domain containing 2-like	-1.76
ENSMUSG00000035642	Aamdc	adipogenesis associated Mth938 domain containing	-1.76
ENSMUSG00000025092	Hspa12a	heat shock protein 12A	-1.76
ENSMUSG00000032773	Chrm1	cholinergic receptor, muscarinic 1, CNS	-1.76
ENSMUSG00000015468	Notch4	notch 4	-1.76
ENSMUSG00000104094	Gm37314	predicted gene, 37314	-1.76
ENSMUSG00000056133	Unc93a2	unc-93 homolog A2	-1.76
ENSMUSG00000068452	Duox2	dual oxidase 2	-1.77
ENSMUSG00000020889	Nr1d1	nuclear receptor subfamily 1, group D, member 1	-1.77
ENSMUSG00000020182	Ddc	dopa decarboxylase	-1.77
ENSMUSG00000046352	Gjb2	gap junction protein, beta 2	-1.77
ENSMUSG00000040432	Ltb4r2	leukotriene B4 receptor 2	-1.77
ENSMUSG00000066516	Klk1b21	kallikrein 1-related peptidase b21	-1.77
ENSMUSG00000071036	Gm10309	predicted gene 10309	-1.77
ENSMUSG00000061718	Ppp1r1b	protein phosphatase 1, regulatory inhibitor subunit 1B	-1.78
ENSMUSG00000032246	Calml4	calmodulin-like 4	-1.78
ENSMUSG00000026726	Cubn	cubilin (intrinsic factor-cobalamin receptor)	-1.78
ENSMUSG00000046688	Tifa	TRAF-interacting protein with forkhead-associated domain	-1.78
ENSMUSG00000034687	Fras1	Fraser extracellular matrix complex subunit 1	-1.78
ENSMUSG00000022799	Arhgap31	Rho GTPase activating protein 31	-1.78
ENSMUSG00000023262	Acy1	aminoacylase 1	-1.78
ENSMUSG00000034765	Dusp5	dual specificity phosphatase 5	-1.78
ENSMUSG00000002108	Nr1h3	nuclear receptor subfamily 1, group H, member 3	-1.78
ENSMUSG00000038128	Camk4	calcium/calmodulin-dependent protein kinase IV	-1.78
ENSMUSG00000106541	Gm43246	predicted gene 43246	-1.78
ENSMUSG00000110062	B430319F04Rik	RIKEN cDNA B430319F04 gene	-1.78
ENSMUSG00000020017	Hal	histidine ammonia lyase	-1.78
ENSMUSG00000074444	Defa30	defensin, alpha, 30	-1.79

ENSMUSG00000038178	Slc43a2	solute carrier family 43, member 2	-1.79
ENSMUSG00000026271	Gpr35	G protein-coupled receptor 35	-1.79
ENSMUSG00000026692	Fmo4	flavin containing monooxygenase 4	-1.79
ENSMUSG00000078451	Ppil6	peptidylprolyl isomerase (cyclophilin)-like 6	-1.79
ENSMUSG00000024511	Rab27b	RAB27B, member RAS oncogene family	-1.80
ENSMUSG00000030281	Il17rc	interleukin 17 receptor C	-1.80
ENSMUSG00000041992	Rapgef5	Rap guanine nucleotide exchange factor (GEF) 5	-1.80
ENSMUSG00000032373	Car12	carbonic anhydrase 12	-1.80
ENSMUSG00000032105	Pdzd3	PDZ domain containing 3	-1.80
ENSMUSG00000026389	Steap3	STEAP family member 3	-1.80
ENSMUSG00000040562	Gstm2	glutathione S-transferase, mu 2	-1.80
ENSMUSG00000106078	Gm43697	predicted gene 43697	-1.80
ENSMUSG00000114705	Gm18760	predicted gene, 18760	-1.80
ENSMUSG00000031144	Syp	synaptophysin	-1.80
ENSMUSG00000006281	Tep1	telomerase associated protein 1	-1.81
ENSMUSG00000029322	Plac8	placenta-specific 8	-1.81
ENSMUSG00000021336	Slc17a4	solute carrier family 17 (sodium phosphate), member 4	-1.81
ENSMUSG00000021779	Thrb	thyroid hormone receptor beta	-1.81
ENSMUSG00000070661	Rnf186	ring finger protein 186	-1.81
ENSMUSG00000074261	Erich4	glutamate rich 4	-1.81
ENSMUSG00000055963	Triqk	triple QxxK/R motif containing	-1.81
ENSMUSG00000041323	Ak7	adenylate kinase 7	-1.81
ENSMUSG00000110289	4930412F12Rik	RIKEN cDNA 4930412F12 gene	-1.81
ENSMUSG00000090608	Gm17200	predicted gene 17200	-1.81
ENSMUSG00000017950	Hnf4a	hepatic nuclear factor 4, alpha	-1.82
ENSMUSG00000021097	Clmn	calmin	-1.82
ENSMUSG00000031209	Heph	hephaestin	-1.82
ENSMUSG00000026480	Ncf2	neutrophil cytosolic factor 2	-1.82
ENSMUSG00000056602	Fry	FRY microtubule binding protein	-1.82
ENSMUSG00000027605	Acss2	acyl-CoA synthetase short-chain family member 2	-1.82
ENSMUSG00000049799	Lrrc19	leucine rich repeat containing 19	-1.82
ENSMUSG00000109438	Gm45073	predicted gene 45073	-1.82
ENSMUSG00000019699	Akt3	thymoma viral proto-oncogene 3	-1.82
ENSMUSG00000035878	Hykk	hydroxylysine kinase 1	-1.82
ENSMUSG00000030616	Sytl2	synaptotagmin-like 2	-1.83
ENSMUSG00000030934	Oat	ornithine aminotransferase	-1.83
ENSMUSG00000037031	Tspan15	tetraspanin 15	-1.83
ENSMUSG00000055435	Maf	avian musculoaponeurotic fibrosarcoma oncogene homolog	-1.83
ENSMUSG00000031853	Map3k21	mitogen-activated protein kinase kinase kinase 21	-1.83
ENSMUSG00000021830	Txndc16	thioredoxin domain containing 16	-1.83
ENSMUSG00000029377	Ereg	epiregulin	-1.83
ENSMUSG00000054598	9130230L23Rik	RIKEN cDNA 9130230L23 gene	-1.83
ENSMUSG00000038239	Hrc	histidine rich calcium binding protein	-1.83
ENSMUSG00000000530	Acvrl1	activin A receptor, type II-like 1	-1.83
ENSMUSG00000109293	Dcst2	DC-STAMP domain containing 2	-1.83
ENSMUSG00000022824	Muc13	mucin 13, epithelial transmembrane	-1.84
ENSMUSG00000022938	Fam3b	family with sequence similarity 3, member B	-1.84
ENSMUSG00000022469	Rapgef3	Rap guanine nucleotide exchange factor (GEF) 3	-1.84
ENSMUSG00000034265	Zdhhc14	zinc finger, DHHC domain containing 14	-1.84
ENSMUSG00000051452	Gm11437	predicted gene 11437	-1.84
ENSMUSG00000112944	Gm48885	predicted gene, 48885	-1.84
ENSMUSG00000096546	Smlr1	small leucine-rich protein 1	-1.84
ENSMUSG00000033872	Best4-ps	bestrophin 4, pseudogene	-1.84
ENSMUSG00000059146	Ntrk3	neurotrophic tyrosine kinase, receptor, type 3	-1.84
ENSMUSG00000108622	Gm36864	predicted gene, 36864	-1.84
ENSMUSG00000003545	Fosb	FBJ osteosarcoma oncogene B	-1.85
ENSMUSG00000024254	Abcg8	ATP binding cassette subfamily G member 8	-1.85
ENSMUSG00000049690	Nckap5	NCK-associated protein 5	-1.85
ENSMUSG00000090214	Gm15657	predicted gene 15657	-1.85
ENSMUSG00000096954	Gdap10	ganglioside-induced differentiation-associated-protein 10	-1.85
ENSMUSG00000020600	Slc7a15	solute carrier family 7 (cationic amino acid transporter, y+ system), member 15	-1.85
ENSMUSG00000038188	Scarf1	scavenger receptor class F, member 1	-1.85
ENSMUSG00000097575	Gm26796	predicted gene, 26796	-1.85
ENSMUSG00000030587	2200002D01Rik	RIKEN cDNA 2200002D01 gene	-1.86
ENSMUSG00000042428	Mgat3	mannoside acetylglucosaminyltransferase 3	-1.86
ENSMUSG00000038903	Ccdc68	coiled-coil domain containing 68	-1.86
ENSMUSG00000039405	Prss23	protease, serine 23	-1.86
ENSMUSG00000022853	Ehhadh	enoyl-Coenzyme A, hydratase/3-hydroxyacyl Coenzyme A dehydrogenase	-1.86
ENSMUSG00000034957	Cebpa	CCAAT/enhancer binding protein (C/EBP), alpha	-1.86
ENSMUSG00000029630	Cyp3a25	cytochrome P450, family 3, subfamily a, polypeptide 25	-1.86
ENSMUSG00000050106	Tmc8	transmembrane channel-like gene family 8	-1.86
ENSMUSG00000021223	Papln	papilin, proteoglycan-like sulfated glycoprotein	-1.86
ENSMUSG00000053675	Tgm5	transglutaminase 5	-1.86
ENSMUSG00000008461	Fut1	fucosyltransferase 1	-1.86
ENSMUSG00000054146	Krt15	keratin 15	-1.86
ENSMUSG00000091475	2810468N07Rik	RIKEN cDNA 2810468N07 gene	-1.86

ENSMUSG00000100182	1810006J02Rik	RIKEN cDNA 1810006J02 gene	-1.86
ENSMUSG00000043020	Wdr63	WD repeat domain 63	-1.86
ENSMUSG00000024180	Tmem8	transmembrane protein 8 (five membrane-spanning domains)	-1.87
ENSMUSG00000056413	Adap1	ArfGAP with dual PH domains 1	-1.87
ENSMUSG00000053310	Nrgn	neurogranin	-1.87
ENSMUSG00000105872	Gm9515	predicted gene 9515	-1.87
ENSMUSG00000042846	Lrrtm3	leucine rich repeat transmembrane neuronal 3	-1.87
ENSMUSG00000027463	Slc52a3	solute carrier protein family 52, member 3	-1.88
ENSMUSG00000027358	Bmp2	bone morphogenetic protein 2	-1.88
ENSMUSG00000024131	Slc3a1	solute carrier family 3, member 1	-1.88
ENSMUSG00000045316	Fahd1	fumarylacetoacetate hydrolase domain containing 1	-1.88
ENSMUSG00000052085	Dock8	dedicator of cytokinesis 8	-1.88
ENSMUSG00000074622	Mafb	v-maf musculoaponeurotic fibrosarcoma oncogene family, protein B (avian)	-1.88
ENSMUSG00000032487	Ptgs2	prostaglandin-endoperoxide synthase 2	-1.88
ENSMUSG00000004151	Etv1	ets variant 1	-1.88
ENSMUSG00000071550	Cfap44	cilia and flagella associated protein 44	-1.88
ENSMUSG00000030838	Ush1c	USH1 protein network component harmonin	-1.89
ENSMUSG00000018909	Arrb1	arrestin, beta 1	-1.89
ENSMUSG00000030650	Tmc5	transmembrane channel-like gene family 5	-1.89
ENSMUSG00000025888	Casp1	caspase 1	-1.89
ENSMUSG00000053862	Slc51b	solute carrier family 51, beta subunit	-1.89
ENSMUSG00000026784	Pdss1	prenyl (solanesyl) diphosphate synthase, subunit 1	-1.89
ENSMUSG00000021798	Ldb3	LIM domain binding 3	-1.89
ENSMUSG00000101693	Gm19461	predicted gene, 19461	-1.89
ENSMUSG00000078650	G6pc	glucose-6-phosphatase, catalytic	-1.89
ENSMUSG00000108353	Gm45205	predicted gene 45205	-1.89
ENSMUSG00000029992	Gfpt1	glutamine fructose-6-phosphate transaminase 1	-1.90
ENSMUSG00000024597	Slc12a2	solute carrier family 12, member 2	-1.90
ENSMUSG00000027901	Dennd2d	DENN/MADD domain containing 2D	-1.90
ENSMUSG00000073421	H2-Ab1	histocompatibility 2, class II antigen A, beta 1	-1.90
ENSMUSG00000044626	Liph	lipase, member H	-1.90
ENSMUSG00000020151	Ptprr	protein tyrosine phosphatase, receptor type, R	-1.90
ENSMUSG00000056671	Prelid2	PRELI domain containing 2	-1.90
ENSMUSG00000043432	Leng9	leukocyte receptor cluster (LRC) member 9	-1.91
ENSMUSG00000029919	Hpgds	hematopoietic prostaglandin D synthase	-1.91
ENSMUSG00000029059	Fam213b	family with sequence similarity 213, member B	-1.91
ENSMUSG00000037440	Vnn1	vanin 1	-1.91
ENSMUSG00000096001	2610528A11Rik	RIKEN cDNA 2610528A11 gene	-1.91
ENSMUSG00000104966	Gm43273	predicted gene 43273	-1.91
ENSMUSG00000098609	Anxa11os	annexin A11, opposite strand	-1.91
ENSMUSG00000085890	Tnfsf13os	tumor necrosis factor (ligand) superfamily, member 13, opposite strand	-1.91
ENSMUSG00000044576	Garem2	GRB2 associated regulator of MAPK1 subtype 2	-1.91
ENSMUSG00000109093	Gm19950	predicted gene, 19950	-1.91
ENSMUSG00000034570	Inpp5j	inositol polyphosphate 5-phosphatase J	-1.92
ENSMUSG00000035000	Dpp4	dipeptidylpeptidase 4	-1.92
ENSMUSG00000012123	Crybg2	crystallin beta-gamma domain containing 2	-1.92
ENSMUSG00000079440	Alpi	alkaline phosphatase, intestinal	-1.92
ENSMUSG00000054999	Naaladl1	N-acetylated alpha-linked acidic dipeptidase-like 1	-1.92
ENSMUSG00000029811	Aoc1	amine oxidase, copper-containing 1	-1.92
ENSMUSG00000069814	Ccdc92b	coiled-coil domain containing 92B	-1.92
ENSMUSG00000033491	Prss35	protease, serine 35	-1.92
ENSMUSG00000112129	Pbld1	phenazine biosynthesis-like protein domain containing 1	-1.92
ENSMUSG00000116738	CT009627.2	TEC	-1.92
ENSMUSG00000086012	Gm15902	predicted gene 15902	-1.92
ENSMUSG00000113935	Gm35732	predicted gene, 35732	-1.92
ENSMUSG00000034127	Tspan8	tetraspanin 8	-1.93
ENSMUSG00000026670	Uap1	UDP-N-acetylglucosamine pyrophosphorylase 1	-1.93
ENSMUSG00000026399	Cd55	CD55 molecule, decay accelerating factor for complement	-1.93
ENSMUSG00000051111	Sv2c	synaptic vesicle glycoprotein 2c	-1.93
ENSMUSG00000109585	Gm45358	predicted gene 45358	-1.93
ENSMUSG00000047414	Flrt2	fibronectin leucine rich transmembrane protein 2	-1.93
ENSMUSG00000109711	Gm45445	predicted gene 45445	-1.93
ENSMUSG00000071185	Olfr1357	olfactory receptor 1357	-1.93
ENSMUSG00000029054	Gabrd	gamma-aminobutyric acid (GABA) A receptor, subunit delta	-1.93
ENSMUSG00000029821	Gsdme	gasdermin E	-1.94
ENSMUSG00000041268	Dmxl2	Dmx-like 2	-1.94
ENSMUSG00000001095	Slc13a2	solute carrier family 13 (sodium-dependent dicarboxylate transporter), member 2	-1.94
ENSMUSG00000053279	Aldh1a1	aldehyde dehydrogenase family 1, subfamily A1	-1.94
ENSMUSG00000021913	Ogdhl	oxoglutarate dehydrogenase-like	-1.94
ENSMUSG00000110060	Gm9860	predicted gene 9860	-1.94
ENSMUSG00000020839	Tmigd1	transmembrane and immunoglobulin domain containing 1	-1.94
ENSMUSG00000112513	4930422I22Rik	RIKEN cDNA 4930422I22 gene	-1.94
ENSMUSG00000087047	1700110K17Rik	RIKEN cDNA 1700110K17 gene	-1.94
ENSMUSG00000040528	Milr1	mast cell immunoglobulin like receptor 1	-1.94
ENSMUSG00000044734	Serpinb1a	serine (or cysteine) peptidase inhibitor, clade B, member 1a	-1.95
ENSMUSG00000074272	Ceacam1	carcinoembryonic antigen-related cell adhesion molecule 1	-1.95

ENSMUSG00000052595	A1cf	APOBEC1 complementation factor	-1.95
ENSMUSG00000025059	Gk	glycerol kinase	-1.95
ENSMUSG00000067206	Lrrc66	leucine rich repeat containing 66	-1.95
ENSMUSG00000027377	Mall	mal, T cell differentiation protein-like	-1.95
ENSMUSG00000033213	AA467197	expressed sequence AA467197	-1.95
ENSMUSG00000106475	Gm43011	predicted gene 43011	-1.95
ENSMUSG00000010830	Kdelr3	KDEL (Lys-Asp-Glu-Leu) endoplasmic reticulum protein retention receptor 3	-1.95
ENSMUSG00000050944	Efcab5	EF-hand calcium binding domain 5	-1.95
ENSMUSG00000004038	Gstm3	glutathione S-transferase, mu 3	-1.95
ENSMUSG00000086479	Gm16014	predicted gene 16014	-1.95
ENSMUSG00000111379	Gm48346	predicted gene, 48346	-1.95
ENSMUSG00000024960	Plcb3	phospholipase C, beta 3	-1.96
ENSMUSG00000031391	L1cam	L1 cell adhesion molecule	-1.96
ENSMUSG00000024105	Themis3	thymocyte selection associated family member 3	-1.96
ENSMUSG00000006345	Ggt1	gamma-glutamyltransferase 1	-1.96
ENSMUSG00000110622	Iqcn	IQ motif containing N	-1.96
ENSMUSG00000069804	Gm10277	predicted gene 10277	-1.96
ENSMUSG00000047728	BC025446	cDNA sequence BC025446	-1.96
ENSMUSG00000038195	Rilp	Rab interacting lysosomal protein	-1.96
ENSMUSG00000087230	Mroh3	maestro heat-like repeat family member 3	-1.96
ENSMUSG00000003032	Klf4	Kruppel-like factor 4 (gut)	-1.97
ENSMUSG00000030865	Chp2	calcineurin-like EF hand protein 2	-1.97
ENSMUSG00000104571	Gm43010	predicted gene 43010	-1.97
ENSMUSG00000019278	Dpep1	dipeptidase 1 (renal)	-1.97
ENSMUSG00000020709	Adap2	ArfGAP with dual PH domains 2	-1.97
ENSMUSG00000029352	Crybb3	crystallin, beta B3	-1.97
ENSMUSG00000099025	Gm27162	predicted gene 27162	-1.97
ENSMUSG00000097257	Gm26768	predicted gene, 26768	-1.97
ENSMUSG00000040249	Lrp1	low density lipoprotein receptor-related protein 1	-1.98
ENSMUSG00000074447	Defa21	defensin, alpha, 21	-1.98
ENSMUSG00000041515	Irf8	interferon regulatory factor 8	-1.98
ENSMUSG00000005686	Ampd3	adenosine monophosphate deaminase 3	-1.98
ENSMUSG00000024222	Fkbp5	FK506 binding protein 5	-1.98
ENSMUSG00000047250	Ptgs1	prostaglandin-endoperoxide synthase 1	-1.98
ENSMUSG00000041565	L3mbtl4	L3MBTL4 histone methyl-lysine binding protein	-1.98
ENSMUSG00000038372	Gmds	GDP-mannose 4, 6-dehydratase	-1.99
ENSMUSG00000053886	Sh2d4a	SH2 domain containing 4A	-1.99
ENSMUSG00000032047	Acat1	acetyl-Coenzyme A acetyltransferase 1	-1.99
ENSMUSG00000037818	Abhd18	abhydrolase domain containing 18	-1.99
ENSMUSG00000046159	Chrm3	cholinergic receptor, muscarinic 3, cardiac	-1.99
ENSMUSG00000019232	Etnppl	ethanolamine phosphate phospholyase	-1.99
ENSMUSG00000038209	Itln1	intelectin 1 (galactofuranose binding)	-2.00
ENSMUSG00000021196	Pfkp	phosphofructokinase, platelet	-2.00
ENSMUSG00000003420	Fcgrt	Fc receptor, IgG, alpha chain transporter	-2.00
ENSMUSG00000029700	Slc13a1	solute carrier family 13 (sodium/sulfate symporters), member 1	-2.00
ENSMUSG00000086825	Gm15675	predicted gene 15675	-2.00
ENSMUSG00000024503	Spink1	serine peptidase inhibitor, Kazal type 1	-2.00
ENSMUSG00000024885	Aldh3b1	aldehyde dehydrogenase 3 family, member B1	-2.00
ENSMUSG00000021379	Id4	inhibitor of DNA binding 4	-2.00
ENSMUSG00000079559	Colca2	COLCA2 homolog	-2.00
ENSMUSG00000106802	Gm42885	predicted gene 42885	-2.00
ENSMUSG00000015970	Chdh	choline dehydrogenase	-2.01
ENSMUSG00000022419	Deptor	DEP domain containing MTOR-interacting protein	-2.01
ENSMUSG00000091705	H2-Q2	histocompatibility 2, Q region locus 2	-2.01
ENSMUSG00000038020	Rapgefl1	Rap guanine nucleotide exchange factor (GEF)-like 1	-2.01
ENSMUSG00000061825	Ces2c	carboxylesterase 2C	-2.01
ENSMUSG00000057182	Scn3a	sodium channel, voltage-gated, type III, alpha	-2.01
ENSMUSG00000028337	Coro2a	coronin, actin binding protein 2A	-2.02
ENSMUSG00000038807	Rap1gap2	RAP1 GTPase activating protein 2	-2.02
ENSMUSG00000028699	Tspan1	tetraspanin 1	-2.02
ENSMUSG00000006360	Crip1	cysteine-rich protein 1 (intestinal)	-2.02
ENSMUSG00000024642	Tle4	transducin-like enhancer of split 4	-2.02
ENSMUSG00000034919	Ttc22	tetratricopeptide repeat domain 22	-2.02
ENSMUSG00000074115	Saa1	serum amyloid A 1	-2.02
ENSMUSG00000034845	Plvap	plasmalemma vesicle associated protein	-2.02
ENSMUSG00000032899	Styk1	serine/threonine/tyrosine kinase 1	-2.03
ENSMUSG00000026688	Mgst3	microsomal glutathione S-transferase 3	-2.03
ENSMUSG00000039899	Fgl2	fibrinogen-like protein 2	-2.03
ENSMUSG00000023045	Soat2	sterol O-acyltransferase 2	-2.03
ENSMUSG00000034947	Tmem106a	transmembrane protein 106A	-2.03
ENSMUSG00000028544	Slc5a9	solute carrier family 5 (sodium/glucose cotransporter), member 9	-2.03
ENSMUSG00000001665	Gstt3	glutathione S-transferase, theta 3	-2.03
ENSMUSG00000115970	8430426J06Rik	RIKEN cDNA 8430426J06 gene	-2.03
ENSMUSG00000004864	Mapk13	mitogen-activated protein kinase 13	-2.04
ENSMUSG00000066800	Rnasel	ribonuclease L (2', 5'-oligoisoadenylate synthetase-dependent)	-2.04
ENSMUSG00000024313	Mep1b	meprin 1 beta	-2.04

ENSMUSG00000074604	Mgst2	microsomal glutathione S-transferase 2	-2.04
ENSMUSG00000024124	Prss30	protease, serine 30	-2.04
ENSMUSG00000022546	Gpt	glutamic pyruvic transaminase, soluble	-2.04
ENSMUSG00000028976	Slc2a5	solute carrier family 2 (facilitated glucose transporter), member 5	-2.04
ENSMUSG00000074196	Clca4c-ps	chloride channel accessory 4C, pseudogene	-2.04
ENSMUSG00000108206	Gm44427	predicted gene, 44427	-2.04
ENSMUSG00000085152	Gm11496	predicted gene 11496	-2.04
ENSMUSG00000021250	Fos	FBJ osteosarcoma oncogene	-2.05
ENSMUSG00000028307	Aldob	aldolase B, fructose-bisphosphate	-2.05
ENSMUSG00000023914	Mep1a	meprin 1 alpha	-2.05
ENSMUSG00000023243	Kcnk5	potassium channel, subfamily K, member 5	-2.05
ENSMUSG00000032978	Guca2b	guanylate cyclase activator 2b (retina)	-2.05
ENSMUSG00000030214	Plbd1	phospholipase B domain containing 1	-2.05
ENSMUSG00000084948	1700061H18Rik	RIKEN cDNA 1700061H18 gene	-2.05
ENSMUSG00000040505	Abcg5	ATP binding cassette subfamily G member 5	-2.06
ENSMUSG00000010601	Apol7a	apolipoprotein L 7a	-2.06
ENSMUSG00000038534	Osbpl7	oxysterol binding protein-like 7	-2.06
ENSMUSG00000030492	Slc7a9	solute carrier family 7 (cationic amino acid transporter, y+ system), member 9	-2.06
ENSMUSG00000044165	Bcl2l15	BCLl2-like 15	-2.06
ENSMUSG00000026928	Card9	caspase recruitment domain family, member 9	-2.06
ENSMUSG00000057074	Ces1g	carboxylesterase 1G	-2.06
ENSMUSG00000054161	Fam83e	family with sequence similarity 83, member E	-2.07
ENSMUSG00000041642	Kif21b	kinesin family member 21B	-2.07
ENSMUSG00000035429	Ptprh	protein tyrosine phosphatase, receptor type, H	-2.07
ENSMUSG00000103696	Gm37531	predicted gene, 37531	-2.07
ENSMUSG00000087326	Gm12503	predicted gene 12503	-2.07
ENSMUSG00000087530	Gm15533	predicted gene 15533	-2.07
ENSMUSG00000050866	Clrn3	clarin 3	-2.08
ENSMUSG00000054469	Lclat1	lysocardiolipin acyltransferase 1	-2.08
ENSMUSG00000032548	Slco2a1	solute carrier organic anion transporter family, member 2a1	-2.08
ENSMUSG00000020062	Slc5a8	solute carrier family 5 (iodide transporter), member 8	-2.08
ENSMUSG00000028150	Rorc	RAR-related orphan receptor gamma	-2.08
ENSMUSG00000074217	2210011C24Rik	RIKEN cDNA 2210011C24 gene	-2.08
ENSMUSG00000115388	Eppk1	epiplakin 1	-2.09
ENSMUSG00000035493	Tgfbi	transforming growth factor, beta induced	-2.09
ENSMUSG00000017639	Rab11fip4	RAB11 family interacting protein 4 (class II)	-2.09
ENSMUSG00000032776	Mctp2	multiple C2 domains, transmembrane 2	-2.09
ENSMUSG00000103527	Gm37261	predicted gene, 37261	-2.09
ENSMUSG00000025189	Cnnm1	cyclin M1	-2.09
ENSMUSG00000072244	Trim6	tripartite motif-containing 6	-2.09
ENSMUSG00000024112	Cacna1h	calcium channel, voltage-dependent, T type, alpha 1H subunit	-2.10
ENSMUSG00000019478	Rab4a	RAB4A, member RAS oncogene family	-2.10
ENSMUSG00000031453	Rasa3	RAS p21 protein activator 3	-2.10
ENSMUSG00000094651	Gal3st2	galactose-3-O-sulfotransferase 2	-2.10
ENSMUSG00000028024	Enpep	glutamyl aminopeptidase	-2.10
ENSMUSG00000034918	Cdhr2	cadherin-related family member 2	-2.10
ENSMUSG00000074345	Tnfaip8l3	tumor necrosis factor, alpha-induced protein 8-like 3	-2.10
ENSMUSG00000030834	Abcc6	ATP-binding cassette, sub-family C (CFTR/MRP), member 6	-2.10
ENSMUSG00000035775	Krt20	keratin 20	-2.11
ENSMUSG00000070777	Ceacam20	carcinoembryonic antigen-related cell adhesion molecule 20	-2.11
ENSMUSG00000108476	Gm44974	predicted gene 44974	-2.11
ENSMUSG00000024292	Cyp4f14	cytochrome P450, family 4, subfamily f, polypeptide 14	-2.12
ENSMUSG00000019888	Mgat4c	MGAT4 family, member C	-2.12
ENSMUSG00000085683	Tmem238l	transmembrane protein 238 like	-2.12
ENSMUSG00000027508	Pag1	phosphoprotein associated with glycosphingolipid microdomains 1	-2.12
ENSMUSG00000058163	Gm5431	predicted gene 5431	-2.12
ENSMUSG00000040441	Slc26a10	solute carrier family 26, member 10	-2.12
ENSMUSG00000072618	Gm10384	predicted gene 10384	-2.12
ENSMUSG00000108961	Gm32540	predicted gene, 32540	-2.12
ENSMUSG00000114945	Gm48265	predicted gene, 48265	-2.12
ENSMUSG00000004266	Ptpn6	protein tyrosine phosphatase, non-receptor type 6	-2.13
ENSMUSG00000073802	Cdkn2b	cyclin dependent kinase inhibitor 2B	-2.13
ENSMUSG00000019872	Smpdl3a	sphingomyelin phosphodiesterase, acid-like 3A	-2.13
ENSMUSG00000068587	Mgam	maltase-glucoamylase	-2.14
ENSMUSG00000060586	H2-Eb1	histocompatibility 2, class II antigen E beta	-2.14
ENSMUSG00000018983	E2f2	E2F transcription factor 2	-2.14
ENSMUSG00000026222	Sp100	nuclear antigen Sp100	-2.14
ENSMUSG00000031980	Agt	angiotensinogen (serpin peptidase inhibitor, clade A, member 8)	-2.14
ENSMUSG00000024846	Cst6	cystatin E/M	-2.14
ENSMUSG00000044361	BC024139	cDNA sequence BC024139	-2.14
ENSMUSG00000026546	Cfap45	cilia and flagella associated protein 45	-2.14
ENSMUSG00000043953	Ccrl2	chemokine (C-C motif) receptor-like 2	-2.14
ENSMUSG00000024734	Zp1	zona pellucida glycoprotein 1	-2.14
ENSMUSG00000078945	Naip2	NLR family, apoptosis inhibitory protein 2	-2.15
ENSMUSG00000024713	Pcsk5	proprotein convertase subtilisin/kexin type 5	-2.15
ENSMUSG00000072812	Ahnak2	AHNAK nucleoprotein 2	-2.15

ENSMUSG00000090115	Usp49	ubiquitin specific peptidase 49	-2.15
ENSMUSG00000000805	Car4	carbonic anhydrase 4	-2.15
ENSMUSG00000033615	Cplx1	complexin 1	-2.15
ENSMUSG00000091443	Gm17023	predicted gene 17023	-2.15
ENSMUSG00000038167	Plekhg6	pleckstrin homology domain containing, family G (with RhoGef domain) member 6	-2.16
ENSMUSG00000031844	Hsd17b2	hydroxysteroid (17-beta) dehydrogenase 2	-2.16
ENSMUSG00000048572	Tmem252	transmembrane protein 252	-2.16
ENSMUSG00000056162	Cndp1	carnosine dipeptidase 1 (metallopeptidase M20 family)	-2.16
ENSMUSG00000030621	Me3	malic enzyme 3, NADP(+)-dependent, mitochondrial	-2.16
ENSMUSG00000110597	Gm8798	predicted gene 8798	-2.16
ENSMUSG00000021062	Rab15	RAB15, member RAS oncogene family	-2.17
ENSMUSG00000024747	Aldh1a7	aldehyde dehydrogenase family 1, subfamily A7	-2.17
ENSMUSG00000097789	Gm2115	predicted gene 2115	-2.17
ENSMUSG00000034116	Vav1	vav 1 oncogene	-2.18
ENSMUSG00000020614	Fam20a	family with sequence similarity 20, member A	-2.18
ENSMUSG00000024247	Pkdcc	protein kinase domain containing, cytoplasmic	-2.18
ENSMUSG00000061728	Btnl7-ps	butyrophilin-like 7, pseudogene	-2.18
ENSMUSG00000091956	C2cd4b	C2 calcium-dependent domain containing 4B	-2.18
ENSMUSG00000026077	Npas2	neuronal PAS domain protein 2	-2.19
ENSMUSG00000028635	Edn2	endothelin 2	-2.19
ENSMUSG00000024697	Gna14	guanine nucleotide binding protein, alpha 14	-2.19
ENSMUSG00000114662	Gm31683	predicted gene, 31683	-2.19
ENSMUSG00000104917	Gm43289	predicted gene 43289	-2.19
ENSMUSG00000031442	Mcf2l	mcf.2 transforming sequence-like	-2.20
ENSMUSG00000027452	Acss1	acyl-CoA synthetase short-chain family member 1	-2.20
ENSMUSG00000046697	Enpp7	ectonucleotide pyrophosphatase/phosphodiesterase 7	-2.20
ENSMUSG00000052131	Akr1b7	aldo-keto reductase family 1, member B7	-2.20
ENSMUSG00000106969	Gm42882	predicted gene 42882	-2.20
ENSMUSG00000027230	Creb3l1	cAMP responsive element binding protein 3-like 1	-2.21
ENSMUSG00000045441	Gprin3	GPRIN family member 3	-2.21
ENSMUSG00000038305	Spats2l	spermatogenesis associated, serine-rich 2-like	-2.21
ENSMUSG00000023259	Slc26a6	solute carrier family 26, member 6	-2.21
ENSMUSG00000103761	Gm37859	predicted gene, 37859	-2.21
ENSMUSG00000033268	Duox1	dual oxidase 1	-2.21
ENSMUSG00000019359	Gdpd2	glycerophosphodiester phosphodiesterase domain containing 2	-2.21
ENSMUSG00000100241	Slc18a3	solute carrier family 18 (vesicular monoamine), member 3	-2.21
ENSMUSG00000041119	Pde9a	phosphodiesterase 9A	-2.22
ENSMUSG00000027200	Sema6d	sema domain, transmembrane domain (TM), and cytoplasmic domain, (semaphorin) 6D	-2.22
ENSMUSG00000031255	Sytl4	synaptotagmin-like 4	-2.22
ENSMUSG00000067231	Cyp2c65	cytochrome P450, family 2, subfamily c, polypeptide 65	-2.22
ENSMUSG00000035969	Rusc2	RUN and SH3 domain containing 2	-2.22
ENSMUSG00000046840	Hnf4aos	hepatic nuclear factor 4 alpha, opposite strand	-2.22
ENSMUSG00000030364	Clec2h	C-type lectin domain family 2, member h	-2.23
ENSMUSG00000031838	Ifi30	interferon gamma inducible protein 30	-2.23
ENSMUSG00000062638	Btnl1	butyrophilin-like 1	-2.23
ENSMUSG00000022809	Nr1i2	nuclear receptor subfamily 1, group I, member 2	-2.23
ENSMUSG00000010660	Plcd1	phospholipase C, delta 1	-2.23
ENSMUSG00000038963	Slco4a1	solute carrier organic anion transporter family, member 4a1	-2.23
ENSMUSG00000052229	Gpr17	G protein-coupled receptor 17	-2.23
ENSMUSG00000021456	Fbp2	fructose bisphosphatase 2	-2.24
ENSMUSG00000031803	B3gnt3	UDP-GlcNAc:betaGal beta-1,3-N-acetylglucosaminyltransferase 3	-2.24
ENSMUSG00000030157	Clec2d	C-type lectin domain family 2, member d	-2.24
ENSMUSG00000036151	Tm6sf2	transmembrane 6 superfamily member 2	-2.24
ENSMUSG00000025790	Slco3a1	solute carrier organic anion transporter family, member 3a1	-2.24
ENSMUSG00000025528	2010106E10Rik	RIKEN cDNA 2010106E10 gene	-2.24
ENSMUSG00000079547	H2-DMb1	histocompatibility 2, class II, locus Mb1	-2.24
ENSMUSG00000031608	Galnt7	polypeptide N-acetylgalactosaminyltransferase 7	-2.25
ENSMUSG00000023959	Clic5	chloride intracellular channel 5	-2.25
ENSMUSG00000021565	Slc6a19	solute carrier family 6 (neurotransmitter transporter), member 19	-2.25
ENSMUSG00000044017	Adgrd1	adhesion G protein-coupled receptor D1	-2.25
ENSMUSG00000033721	Vav3	vav 3 oncogene	-2.25
ENSMUSG00000090293	Gm17034	predicted gene 17034	-2.25
ENSMUSG00000045912	C2cd4c	C2 calcium-dependent domain containing 4C	-2.25
ENSMUSG00000104519	Gm37161	predicted gene, 37161	-2.25
ENSMUSG00000090145	Ugt1a6b	UDP glucuronosyltransferase 1 family, polypeptide A6B	-2.25
ENSMUSG00000021676	Iqgap2	IQ motif containing GTPase activating protein 2	-2.26
ENSMUSG00000027227	Sord	sorbitol dehydrogenase	-2.26
ENSMUSG00000034785	Dio1	deiodinase, iodothyronine, type I	-2.26
ENSMUSG00000031497	Tnfsf13b	tumor necrosis factor (ligand) superfamily, member 13b	-2.26
ENSMUSG00000025497	Cdhr5	cadherin-related family member 5	-2.27
ENSMUSG00000031886	Ces2e	carboxylesterase 2E	-2.27
ENSMUSG00000024140	Epas1	endothelial PAS domain protein 1	-2.27
ENSMUSG00000021553	Slc28a3	solute carrier family 28 (sodium-coupled nucleoside transporter), member 3	-2.27
ENSMUSG00000023827	Agpat4	1-acylglycerol-3-phosphate O-acyltransferase 4 (lysophosphatidic acid acyltransferase, delta)	-2.27
ENSMUSG00000029275	Gfi1	growth factor independent 1	-2.27
ENSMUSG00000105456	Gm43745	predicted gene 43745	-2.27

ENSMUSG00000097316	Gm10516	predicted gene 10516	-2.27
ENSMUSG00000109372	Gm19410	predicted gene, 19410	-2.27
ENSMUSG00000017688	Hnf4g	hepatocyte nuclear factor 4, gamma	-2.28
ENSMUSG00000024277	Mapre2	microtubule-associated protein, RP/EB family, member 2	-2.28
ENSMUSG00000040537	Adam22	a disintegrin and metallopeptidase domain 22	-2.28
ENSMUSG00000105986	Gm43065	predicted gene 43065	-2.28
ENSMUSG00000037408	Cnnm4	cyclin M4	-2.29
ENSMUSG00000024340	Btnl2	butyrophilin-like 2	-2.29
ENSMUSG00000025401	Myo1a	myosin IA	-2.30
ENSMUSG00000034449	Dhrs11	dehydrogenase/reductase (SDR family) member 11	-2.30
ENSMUSG00000085289	Gm15337	predicted gene 15337	-2.30
ENSMUSG00000037033	Clca3b	chloride channel accessory 3B	-2.31
ENSMUSG00000035699	Slc51a	solute carrier family 51, alpha subunit	-2.31
ENSMUSG00000028940	Hes2	hes family bHLH transcription factor 2	-2.31
ENSMUSG00000089635	Gm16559	predicted gene 16559	-2.31
ENSMUSG00000031618	Nr3c2	nuclear receptor subfamily 3, group C, member 2	-2.32
ENSMUSG00000096299	Gm21814	predicted gene, 21814	-2.32
ENSMUSG00000110557	Gm5159	predicted gene 5159	-2.32
ENSMUSG00000082677	Gm15371	predicted gene 15371	-2.32
ENSMUSG00000043501	Lgals2	lectin, galactose-binding, soluble 2	-2.33
ENSMUSG00000027790	Sis	sucrase isomaltase (alpha-glucosidase)	-2.33
ENSMUSG00000049858	Suox	sulfite oxidase	-2.33
ENSMUSG00000027317	Ppp1r14d	protein phosphatase 1, regulatory inhibitor subunit 14D	-2.33
ENSMUSG00000055730	Ces2a	carboxylesterase 2A	-2.33
ENSMUSG00000110266	Gm32742	predicted gene, 32742	-2.33
ENSMUSG00000000308	Ckmt1	creatine kinase, mitochondrial 1, ubiquitous	-2.34
ENSMUSG00000021640	Naip1	NLR family, apoptosis inhibitory protein 1	-2.34
ENSMUSG00000038751	Ptk6	PTK6 protein tyrosine kinase 6	-2.34
ENSMUSG00000073403	Gm10499	predicted gene 10499	-2.34
ENSMUSG00000023328	Ache	acetylcholinesterase	-2.34
ENSMUSG00000059213	Ddn	dendrin	-2.34
ENSMUSG00000043822	Adamtsl5	ADAMTS-like 5	-2.35
ENSMUSG00000027173	Depdc7	DEP domain containing 7	-2.35
ENSMUSG00000113680	Gm47405	predicted gene, 47405	-2.35
ENSMUSG00000032726	Bmp8a	bone morphogenetic protein 8a	-2.35
ENSMUSG00000018126	Baiap2l2	BAI1-associated protein 2-like 2	-2.36
ENSMUSG00000092618	Btnl6	butyrophilin-like 6	-2.36
ENSMUSG00000005373	Mlxipl	MLX interacting protein-like	-2.36
ENSMUSG00000033308	Dpyd	dihydropyrimidine dehydrogenase	-2.36
ENSMUSG00000040767	Snrnp25	small nuclear ribonucleoprotein 25 (U11/U12)	-2.36
ENSMUSG00000049307	Fut4	fucosyltransferase 4	-2.36
ENSMUSG00000050097	Ces2b	carboxyesterase 2B	-2.36
ENSMUSG00000087615	Pnpla1os	patatin-like phospholipase domain containing 1, opposite strand	-2.36
ENSMUSG00000052415	Tchh	trichohyalin	-2.37
ENSMUSG00000034427	Myo15b	myosin XVB	-2.38
ENSMUSG00000078439	Smim24	small integral membrane protein 24	-2.38
ENSMUSG00000037003	Tns2	tensin 2	-2.38
ENSMUSG00000032690	Oas2	2'-5' oligoadenylate synthetase 2	-2.38
ENSMUSG00000050592	Fam78a	family with sequence similarity 78, member A	-2.38
ENSMUSG00000025726	Slc28a1	solute carrier family 28 (sodium-coupled nucleoside transporter), member 1	-2.38
ENSMUSG00000100738	2010106C02Rik	RIKEN cDNA 2010106C02 gene	-2.38
ENSMUSG00000030366	Ceacam12	carcinoembryonic antigen-related cell adhesion molecule 12	-2.38
ENSMUSG00000029269	Sult1b1	sulfotransferase family 1B, member 1	-2.39
ENSMUSG00000036377	C530008M17Rik	RIKEN cDNA C530008M17 gene	-2.39
ENSMUSG00000043592	Unc5cl	unc-5 family C-terminal like	-2.39
ENSMUSG00000033871	Ppargc1b	peroxisome proliferative activated receptor, gamma, coactivator 1 beta	-2.39
ENSMUSG00000030683	Sez6l2	seizure related 6 homolog like 2	-2.39
ENSMUSG00000024619	Cdx1	caudal type homeobox 1	-2.40
ENSMUSG00000037280	Galnt6	polypeptide N-acetylgalactosaminyltransferase 6	-2.40
ENSMUSG00000021416	Eci3	enoyl-Coenzyme A delta isomerase 3	-2.40
ENSMUSG00000092517	Art2a-ps	ADP-ribosyltransferase 2a, pseudogene	-2.40
ENSMUSG00000047298	Kcnv2	potassium channel, subfamily V, member 2	-2.40
ENSMUSG00000030017	Reg3g	regenerating islet-derived 3 gamma	-2.41
ENSMUSG00000034528	Hsd17b13	hydroxysteroid (17-beta) dehydrogenase 13	-2.41
ENSMUSG00000039339	Mfsd4b2	major facilitator superfamily domain containing 4B2	-2.41
ENSMUSG00000014786	Slc9a5	solute carrier family 9 (sodium/hydrogen exchanger), member 5	-2.41
ENSMUSG00000049999	Ppp1r3d	protein phosphatase 1, regulatory subunit 3D	-2.41
ENSMUSG00000071265	1700086L19Rik	RIKEN cDNA 1700086L19 gene	-2.41
ENSMUSG00000035561	Aldh1b1	aldehyde dehydrogenase 1 family, member B1	-2.42
ENSMUSG00000064213	Defa24	defensin, alpha, 24	-2.42
ENSMUSG00000005045	Chd5	chromodomain helicase DNA binding protein 5	-2.42
ENSMUSG00000028874	Fgr	FGR proto-oncogene, Src family tyrosine kinase	-2.42
ENSMUSG00000095328	Defa-ps6	defensin, alpha, pseudogene 6	-2.43
ENSMUSG00000042828	Trim72	tripartite motif-containing 72	-2.43
ENSMUSG00000081245	Gm6587	predicted gene 6587	-2.43
ENSMUSG00000105822	Gm42969	predicted gene 42969	-2.43

ENSMUSG00000050965	Prkca	protein kinase C, alpha	-2.44
ENSMUSG00000050982	Apol10a	apolipoprotein L 10A	-2.44
ENSMUSG00000006642	Tcf23	transcription factor 23	-2.44
ENSMUSG00000071356	Reg3b	regenerating islet-derived 3 beta	-2.45
ENSMUSG00000017978	Cadps2	Ca2+-dependent activator protein for secretion 2	-2.45
ENSMUSG00000054453	Sytl5	synaptotagmin-like 5	-2.45
ENSMUSG00000093458	Gm20688	predicted gene 20688	-2.45
ENSMUSG00000094091	Gm21885	predicted gene, 21885	-2.46
ENSMUSG00000057614	Gnai1	guanine nucleotide binding protein (G protein), alpha inhibiting 1	-2.46
ENSMUSG00000024957	Kcnk4	potassium channel, subfamily K, member 4	-2.46
ENSMUSG00000024548	Setbp1	SET binding protein 1	-2.47
ENSMUSG00000021278	Amn	amnionless	-2.47
ENSMUSG00000057880	Abat	4-aminobutyrate aminotransferase	-2.48
ENSMUSG00000039981	Zc3h12d	zinc finger CCCH type containing 12D	-2.48
ENSMUSG00000021221	Dpf3	D4, zinc and double PHD fingers, family 3	-2.48
ENSMUSG00000107310	Gm7452	predicted pseudogene 7452	-2.48
ENSMUSG00000049598	Vsig8	V-set and immunoglobulin domain containing 8	-2.48
ENSMUSG00000026825	Dnm1	dynamin 1	-2.49
ENSMUSG00000039578	Ccser1	coiled-coil serine rich 1	-2.49
ENSMUSG00000079445	B3gnt7	UDP-GlcNAc:betaGal beta-1,3-N-acetylglucosaminyltransferase 7	-2.49
ENSMUSG00000031937	Vstm5	V-set and transmembrane domain containing 5	-2.49
ENSMUSG00000025876	Unc5a	unc-5 netrin receptor A	-2.49
ENSMUSG00000060208	Defa17	defensin, alpha, 17	-2.49
ENSMUSG00000040770	Il25	interleukin 25	-2.49
ENSMUSG00000024743	Syt7	synaptotagmin VII	-2.50
ENSMUSG00000073399	Trim40	tripartite motif-containing 40	-2.50
ENSMUSG00000024731	Ms4a10	membrane-spanning 4-domains, subfamily A, member 10	-2.50
ENSMUSG00000071551	Akr1c19	aldo-keto reductase family 1, member C19	-2.50
ENSMUSG00000047528	Als2cr12	amyotrophic lateral sclerosis 2 chromosome region 12	-2.50
ENSMUSG00000112489	9230116L04Rik	RIKEN cDNA 9230116L04 gene	-2.50
ENSMUSG00000012777	Acp4	acid phosphatase 4	-2.50
ENSMUSG00000066178	6030445D17Rik	RIKEN cDNA 6030445D17 gene	-2.50
ENSMUSG00000063142	Kcnma1	potassium large conductance calcium-activated channel, subfamily M, alpha member 1	-2.51
ENSMUSG00000054640	Slc8a1	solute carrier family 8 (sodium/calcium exchanger), member 1	-2.51
ENSMUSG00000031883	Car7	carbonic anhydrase 7	-2.51
ENSMUSG00000026417	Pigr	polymeric immunoglobulin receptor	-2.52
ENSMUSG00000038515	Grtp1	GH regulated TBC protein 1	-2.52
ENSMUSG00000030472	Ceacam18	carcinoembryonic antigen-related cell adhesion molecule 18	-2.52
ENSMUSG00000108688	Gm44985	predicted gene 44985	-2.52
ENSMUSG00000047878	A4galt	alpha 1,4-galactosyltransferase	-2.52
ENSMUSG00000060703	Cd302	CD302 antigen	-2.52
ENSMUSG00000019906	Lin7a	lin-7 homolog A (C. elegans)	-2.52
ENSMUSG00000031621	Isx	intestine specific homeobox	-2.53
ENSMUSG00000025347	Mettl7b	methyltransferase like 7B	-2.53
ENSMUSG00000053004	Hrh1	histamine receptor H1	-2.53
ENSMUSG00000005360	Slc1a3	solute carrier family 1 (glial high affinity glutamate transporter), member 3	-2.53
ENSMUSG00000032034	Kcnj5	potassium inwardly-rectifying channel, subfamily J, member 5	-2.53
ENSMUSG00000101814	Gm17807	predicted gene, 17807	-2.54
ENSMUSG00000025037	Maoa	monoamine oxidase A	-2.55
ENSMUSG00000036814	Slc6a20a	solute carrier family 6 (neurotransmitter transporter), member 20A	-2.55
ENSMUSG00000040471	Ggt6	gamma-glutamyltransferase 6	-2.55
ENSMUSG00000110573	Gm5485	predicted gene 5485	-2.55
ENSMUSG00000102140	Gm38058	predicted gene, 38058	-2.55
ENSMUSG00000025207	Sema4g	sema domain, immunoglobulin domain (Ig), transmembrane domain (TM) and short cytoplasmic do	-2.56
ENSMUSG00000005672	Kit	KIT proto-oncogene receptor tyrosine kinase	-2.56
ENSMUSG00000060002	Chpt1	choline phosphotransferase 1	-2.56
ENSMUSG00000057897	Camk2b	calcium/calmodulin-dependent protein kinase II, beta	-2.56
ENSMUSG00000028262	Clca3a2	chloride channel accessory 3A2	-2.56
ENSMUSG00000032098	Treh	trehalase (brush-border membrane glycoprotein)	-2.56
ENSMUSG00000109644	0610005C13Rik	RIKEN cDNA 0610005C13 gene	-2.56
ENSMUSG00000061762	Tac1	tachykinin 1	-2.56
ENSMUSG00000110238	Gm19269	predicted gene, 19269	-2.56
ENSMUSG00000020072	Pbld2	phenazine biosynthesis-like protein domain containing 2	-2.57
ENSMUSG00000031445	Proz	protein Z, vitamin K-dependent plasma glycoprotein	-2.57
ENSMUSG00000075611	Gm11545	predicted gene 11545	-2.57
ENSMUSG00000022747	St3gal6	ST3 beta-galactoside alpha-2,3-sialyltransferase 6	-2.58
ENSMUSG00000032181	Scg3	secretogranin III	-2.58
ENSMUSG00000004113	Cacna1b	calcium channel, voltage-dependent, N type, alpha 1B subunit	-2.58
ENSMUSG00000053964	Lgals4	lectin, galactose binding, soluble 4	-2.59
ENSMUSG00000037005	Xpnpep2	X-prolyl aminopeptidase (aminopeptidase P) 2, membrane-bound	-2.59
ENSMUSG00000029162	Khk	ketohexokinase	-2.59
ENSMUSG00000084978	Gm11655	predicted gene 11655	-2.59
ENSMUSG00000075517	Cyp2d37-ps	cytochrome P450, family 2, subfamily d, polypeptide 37, pseudogene	-2.59
ENSMUSG00000032377	Plscr4	phospholipid scramblase 4	-2.59
ENSMUSG00000055567	Unc80	unc-80, NALCN activator	-2.59
ENSMUSG00000108155	Gm44443	predicted gene, 44443	-2.59

ENSMUSG00000054128	H2-T3	histocompatibility 2, T region locus 3	-2.60
ENSMUSG00000058435	Btnl4	butyrophilin-like 4	-2.60
ENSMUSG00000071203	Naip5	NLR family, apoptosis inhibitory protein 5	-2.61
ENSMUSG00000029084	Cd38	CD38 antigen	-2.61
ENSMUSG00000028179	Cth	cystathionase (cystathionine gamma-lyase)	-2.61
ENSMUSG00000040653	Ppp1r14c	protein phosphatase 1, regulatory inhibitor subunit 14C	-2.61
ENSMUSG00000086067	Gm16183	predicted gene 16183	-2.61
ENSMUSG00000050296	Abca12	ATP-binding cassette, sub-family A (ABC1), member 12	-2.62
ENSMUSG00000072941	Sod3	superoxide dismutase 3, extracellular	-2.62
ENSMUSG00000022947	Cbr3	carbonyl reductase 3	-2.62
ENSMUSG00000013643	Lypd8	LY6/PLAUR domain containing 8	-2.63
ENSMUSG00000055114	Anxa13	annexin A13	-2.63
ENSMUSG00000030866	Ern2	endoplasmic reticulum (ER) to nucleus signalling 2	-2.63
ENSMUSG00000038910	Plcl2	phospholipase C-like 2	-2.63
ENSMUSG00000027876	Reg4	regenerating islet-derived family, member 4	-2.63
ENSMUSG00000014773	Dll1	delta like canonical Notch ligand 1	-2.63
ENSMUSG00000107524	Gm44136	predicted gene, 44136	-2.63
ENSMUSG00000033847	Pla2g4c	phospholipase A2, group IVC (cytosolic, calcium-independent)	-2.64
ENSMUSG00000013523	Bcas1	breast carcinoma amplified sequence 1	-2.64
ENSMUSG00000020080	Hkdc1	hexokinase domain containing 1	-2.64
ENSMUSG00000058921	Slc10a5	solute carrier family 10 (sodium/bile acid cotransporter family), member 5	-2.64
ENSMUSG00000027555	Car13	carbonic anhydrase 13	-2.64
ENSMUSG00000027297	Ltk	leukocyte tyrosine kinase	-2.64
ENSMUSG00000032038	St3gal4	ST3 beta-galactoside alpha-2,3-sialyltransferase 4	-2.65
ENSMUSG00000026126	Ptpn18	protein tyrosine phosphatase, non-receptor type 18	-2.65
ENSMUSG00000021208	Ifi27l2b	interferon, alpha-inducible protein 27 like 2B	-2.65
ENSMUSG00000073420	Btnl5-ps	butyrophilin-like 5, pseudogene	-2.65
ENSMUSG00000047638	Nr1h4	nuclear receptor subfamily 1, group H, member 4	-2.65
ENSMUSG00000023235	Ccl25	chemokine (C-C motif) ligand 25	-2.66
ENSMUSG00000060508	Nlrp9b	NLR family, pyrin domain containing 9B	-2.66
ENSMUSG00000074300	BC030870	cDNA sequence BC030870	-2.66
ENSMUSG00000116929	AC164088.2	TEC	-2.66
ENSMUSG00000070315	4930581F22Rik	RIKEN cDNA 4930581F22 gene	-2.67
ENSMUSG00000022340	Sybu	syntabulin (syntaxin-interacting)	-2.67
ENSMUSG00000037451	Slc22a20	solute carrier family 22 (organic anion transporter), member 20	-2.67
ENSMUSG00000091387	Gcnt4	glucosaminyl (N-acetyl) transferase 4, core 2 (beta-1,6-N-acetylglucosaminyltransferase)	-2.68
ENSMUSG00000014361	Mertk	c-mer proto-oncogene tyrosine kinase	-2.68
ENSMUSG00000085042	Abhd11os	abhydrolase domain containing 11, opposite strand	-2.68
ENSMUSG00000026489	Coq8a	coenzyme Q8A	-2.68
ENSMUSG00000070034	Sp110	Sp110 nuclear body protein	-2.68
ENSMUSG00000096914	Galntl6	UDP-N-acetyl-alpha-D-galactosamine:polypeptide N-acetylgalactosaminyltransferase-like 6	-2.68
ENSMUSG00000032297	Celf6	CUGBP, Elav-like family member 6	-2.68
ENSMUSG00000075702	Selenom	selenoprotein M	-2.69
ENSMUSG00000024225	Clps	colipase, pancreatic	-2.69
ENSMUSG00000074067	Gm10619	predicted gene 10619	-2.69
ENSMUSG00000046410	Kcnk6	potassium inwardly-rectifying channel, subfamily K, member 6	-2.70
ENSMUSG00000059654	Reg1	regenerating islet-derived 1	-2.70
ENSMUSG00000021947	Cryl1	crystallin, lambda 1	-2.71
ENSMUSG00000031163	Glod5	glyoxalase domain containing 5	-2.71
ENSMUSG00000071335	Mfsd4b3	major facilitator superfamily domain containing 4B3	-2.71
ENSMUSG00000027107	Chrna1	cholinergic receptor, nicotinic, alpha polypeptide 1 (muscle)	-2.71
ENSMUSG00000046908	Ltb4r1	leukotriene B4 receptor 1	-2.71
ENSMUSG00000040703	Cyp2s1	cytochrome P450, family 2, subfamily s, polypeptide 1	-2.72
ENSMUSG00000091575	2010016I18Rik	RIKEN cDNA 2010016I18 gene	-2.72
ENSMUSG00000031613	Hpgd	hydroxyprostaglandin dehydrogenase 15 (NAD)	-2.73
ENSMUSG00000020838	Slc6a4	solute carrier family 6 (neurotransmitter transporter, serotonin), member 4	-2.73
ENSMUSG00000032357	Tinag	tubulointerstitial nephritis antigen	-2.73
ENSMUSG00000027792	Bche	butyrylcholinesterase	-2.74
ENSMUSG00000057719	Sh3rf2	SH3 domain containing ring finger 2	-2.74
ENSMUSG00000028427	Aqp7	aquaporin 7	-2.74
ENSMUSG00000116010	Gm36026	predicted gene, 36026	-2.74
ENSMUSG00000063296	Tmem117	transmembrane protein 117	-2.75
ENSMUSG00000044352	Sowaha	sosondowah ankyrin repeat domain family member A	-2.75
ENSMUSG00000067377	Tspan6	tetraspanin 6	-2.75
ENSMUSG00000038745	Nlrp6	NLR family, pyrin domain containing 6	-2.76
ENSMUSG00000006731	B4galnt1	beta-1,4-N-acetyl-galactosaminyl transferase 1	-2.76
ENSMUSG00000061531	Tmem236	transmembrane protein 236	-2.76
ENSMUSG00000022235	Cmbl	carboxymethylenebutenolidase-like (Pseudomonas)	-2.76
ENSMUSG00000043705	Capn13	calpain 13	-2.77
ENSMUSG00000041741	Pde3a	phosphodiesterase 3A, cGMP inhibited	-2.77
ENSMUSG00000031364	Grpr	gastrin releasing peptide receptor	-2.77
ENSMUSG00000032528	Vipr1	vasoactive intestinal peptide receptor 1	-2.78
ENSMUSG00000020105	Lrig3	leucine-rich repeats and immunoglobulin-like domains 3	-2.78
ENSMUSG00000045775	Slc16a5	solute carrier family 16 (monocarboxylic acid transporters), member 5	-2.78
ENSMUSG00000060176	Kif27	kinesin family member 27	-2.78
ENSMUSG00000024730	Ms4a8a	membrane-spanning 4-domains, subfamily A, member 8A	-2.79

ENSMUSG00000032226	Gcnt3	glucosaminyl (N-acetyl) transferase 3, mucin type	-2.80
ENSMUSG00000029330	Cds1	CDP-diacylglycerol synthase 1	-2.80
ENSMUSG00000024411	Aqp4	aquaporin 4	-2.80
ENSMUSG00000036298	Slc2a13	solute carrier family 2 (facilitated glucose transporter), member 13	-2.80
ENSMUSG00000033174	Mgll	monoglyceride lipase	-2.80
ENSMUSG00000021396	Nxnl2	nucleoredoxin-like 2	-2.80
ENSMUSG00000004655	Aqp1	aquaporin 1	-2.81
ENSMUSG00000022755	Adgrg7	adhesion G protein-coupled receptor G7	-2.81
ENSMUSG00000081303	Gm16011	predicted gene 16011	-2.81
ENSMUSG00000026870	Cutal	cutA divalent cation tolerance homolog-like	-2.81
ENSMUSG00000029420	Rimbp2	RIMS binding protein 2	-2.81
ENSMUSG00000057914	Cacnb2	calcium channel, voltage-dependent, beta 2 subunit	-2.81
ENSMUSG00000090700	Cyp4f40	cytochrome P450, family 4, subfamily f, polypeptide 40	-2.83
ENSMUSG00000073413	Ly6g6d	lymphocyte antigen 6 complex, locus G6D	-2.83
ENSMUSG00000041644	Slc5a12	solute carrier family 5 (sodium/glucose cotransporter), member 12	-2.83
ENSMUSG00000027420	Bfsp1	beaded filament structural protein 1, in lens-CP94	-2.84
ENSMUSG00000028278	Rragd	Ras-related GTP binding D	-2.84
ENSMUSG00000027224	Duoxa1	dual oxidase maturation factor 1	-2.84
ENSMUSG00000097445	Gm26631	predicted gene, 26631	-2.84
ENSMUSG00000021216	Tubal3	tubulin, alpha-like 3	-2.85
ENSMUSG00000047115	Fam221a	family with sequence similarity 221, member A	-2.85
ENSMUSG00000039419	Cntnap2	contactin associated protein-like 2	-2.85
ENSMUSG00000045005	Fzd5	frizzled class receptor 5	-2.86
ENSMUSG00000053182	Gm609	predicted gene 609	-2.86
ENSMUSG00000006362	Cbfa2t3	core-binding factor, runt domain, alpha subunit 2, translocated to, 3 (human)	-2.86
ENSMUSG00000051726	Kcnf1	potassium voltage-gated channel, subfamily F, member 1	-2.86
ENSMUSG00000071633	Gm4952	predicted gene 4952	-2.86
ENSMUSG00000041301	Cftr	cystic fibrosis transmembrane conductance regulator	-2.87
ENSMUSG00000075520	Malrd1	MAM and LDL receptor class A domain containing 1	-2.87
ENSMUSG00000019989	Enpp3	ectonucleotide pyrophosphatase/phosphodiesterase 3	-2.87
ENSMUSG00000087430	Gm13405	predicted gene 13405	-2.87
ENSMUSG00000026380	Tfcp2l1	transcription factor CP2-like 1	-2.88
ENSMUSG00000040412	5330417C22Rik	RIKEN cDNA 5330417C22 gene	-2.88
ENSMUSG00000032033	Barx2	BarH-like homeobox 2	-2.88
ENSMUSG00000036912	Piwil4	piwi-like RNA-mediated gene silencing 4	-2.88
ENSMUSG00000046804	Phgr1	proline/histidine/glycine-rich 1	-2.89
ENSMUSG00000037686	Aspg	asparaginase	-2.89
ENSMUSG00000108388	Gm44673	predicted gene 44673	-2.89
ENSMUSG00000109446	Gm9195	predicted gene 9195	-2.89
ENSMUSG00000029161	Cgref1	cell growth regulator with EF hand domain 1	-2.90
ENSMUSG00000054385	Ceacam2	carcinoembryonic antigen-related cell adhesion molecule 2	-2.90
ENSMUSG00000027843	Ptpn22	protein tyrosine phosphatase, non-receptor type 22 (lymphoid)	-2.90
ENSMUSG00000035849	Krt222	keratin 222	-2.90
ENSMUSG00000027669	Gnb4	guanine nucleotide binding protein (G protein), beta 4	-2.90
ENSMUSG00000058063	Trim31	tripartite motif-containing 31	-2.91
ENSMUSG00000116220	A430088P11Rik	RIKEN cDNA A430088P11 gene	-2.91
ENSMUSG00000036422	Pcdh8	protocadherin 8	-2.91
ENSMUSG00000021194	Chga	chromogranin A	-2.91
ENSMUSG00000041261	Car8	carbonic anhydrase 8	-2.92
ENSMUSG00000024620	Pdgfrb	platelet derived growth factor receptor, beta polypeptide	-2.92
ENSMUSG00000087389	Gm15592	predicted gene 15592	-2.92
ENSMUSG00000094840	Muc3a	mucin 3A, cell surface associated	-2.93
ENSMUSG00000005268	Prlr	prolactin receptor	-2.93
ENSMUSG00000049134	Nrap	nebulin-related anchoring protein	-2.93
ENSMUSG00000103579	Gm37113	predicted gene, 37113	-2.93
ENSMUSG00000090291	Lrrc10b	leucine rich repeat containing 10B	-2.93
ENSMUSG00000078670	Fam174b	family with sequence similarity 174, member B	-2.94
ENSMUSG00000029134	Plb1	phospholipase B1	-2.94
ENSMUSG00000046613	Vwa5b2	von Willebrand factor A domain containing 5B2	-2.94
ENSMUSG00000073608	Gal3st2c	galactose-3-O-sulfotransferase 2C	-2.94
ENSMUSG00000018919	Tm4sf5	transmembrane 4 superfamily member 5	-2.95
ENSMUSG00000041696	Rasl12	RAS-like, family 12	-2.95
ENSMUSG00000015224	Cyp2j9	cytochrome P450, family 2, subfamily j, polypeptide 9	-2.95
ENSMUSG00000001739	Cldn15	claudin 15	-2.96
ENSMUSG00000010066	Cacna2d2	calcium channel, voltage-dependent, alpha 2/delta subunit 2	-2.96
ENSMUSG00000028327	Stra6l	STRA6-like	-2.97
ENSMUSG00000031451	Gas6	growth arrest specific 6	-2.98
ENSMUSG00000026398	Nr5a2	nuclear receptor subfamily 5, group A, member 2	-2.98
ENSMUSG00000030340	Scnn1a	sodium channel, nonvoltage-gated 1 alpha	-2.98
ENSMUSG00000021850	ccdc198	coiled-coil domain containing 198	-2.98
ENSMUSG00000091189	Ear-ps3	eosinophil-associated, ribonuclease A family, pseudogene 3	-2.98
ENSMUSG00000042284	Itga1	integrin alpha 1	-2.99
ENSMUSG00000001166	Oas1c	2'-5' oligoadenylate synthetase 1C	-2.99
ENSMUSG00000015401	Cltrn	collectrin, amino acid transport regulator	-2.99
ENSMUSG00000030793	Pycard	PYD and CARD domain containing	-3.00
ENSMUSG00000094219	Calhm3	calcium homeostasis modulator 3	-3.00

ENSMUSG00000027870	Hao2	hydroxyacid oxidase 2	-3.00
ENSMUSG00000028251	Tstd3	thiosulfate sulfurtransferase (rhodanese)-like domain containing 3	-3.01
ENSMUSG00000036960	Clca2	chloride channel accessory 2	-3.01
ENSMUSG00000024379	Tslp	thymic stromal lymphopoietin	-3.01
ENSMUSG00000093867	Gsdmcl2	gasdermin C-like 2	-3.02
ENSMUSG00000022661	Cd200	CD200 antigen	-3.02
ENSMUSG00000085723	Gm15915	predicted gene 15915	-3.02
ENSMUSG00000022479	Vdr	vitamin D (1,25-dihydroxyvitamin D3) receptor	-3.03
ENSMUSG00000022408	Fam83f	family with sequence similarity 83, member F	-3.03
ENSMUSG00000023032	Slc4a8	solute carrier family 4 (anion exchanger), member 8	-3.03
ENSMUSG00000022262	Dnah5	dynein, axonemal, heavy chain 5	-3.03
ENSMUSG00000056752	Dnah9	dynein, axonemal, heavy chain 9	-3.04
ENSMUSG00000027513	Pck1	phosphoenolpyruvate carboxykinase 1, cytosolic	-3.05
ENSMUSG00000038917	3930402G23Rik	RIKEN cDNA 3930402G23 gene	-3.05
ENSMUSG00000001225	Slc26a3	solute carrier family 26, member 3	-3.06
ENSMUSG00000090124	Ugt1a7c	UDP glucuronosyltransferase 1 family, polypeptide A7C	-3.06
ENSMUSG00000048022	Tmem229a	transmembrane protein 229A	-3.06
ENSMUSG00000059003	Grin2a	glutamate receptor, ionotropic, NMDA2A (epsilon 1)	-3.06
ENSMUSG00000028003	Lrat	lecithin-retinol acyltransferase (phosphatidylcholine-retinol-O-acyltransferase)	-3.07
ENSMUSG00000020865	Abcc3	ATP-binding cassette, sub-family C (CFTR/MRP), member 3	-3.08
ENSMUSG00000024935	Slc1a1	solute carrier family 1 (neuronal/epithelial high affinity glutamate transporter, system Xag), membe	-3.08
ENSMUSG00000029490	Mfsd7a	major facilitator superfamily domain containing 7A	-3.08
ENSMUSG00000079355	Ackr4	atypical chemokine receptor 4	-3.09
ENSMUSG00000028525	Pde4b	phosphodiesterase 4B, cAMP specific	-3.09
ENSMUSG00000027350	Chgb	chromogranin B	-3.09
ENSMUSG00000025010	Ccnj	cyclin J	-3.10
ENSMUSG00000035506	Slc12a8	solute carrier family 12 (potassium/chloride transporters), member 8	-3.10
ENSMUSG00000034112	Atp2c2	ATPase, Ca++ transporting, type 2C, member 2	-3.10
ENSMUSG00000096751	Gm28373	predicted gene 28373	-3.10
ENSMUSG00000044748	Defb1	defensin beta 1	-3.10
ENSMUSG00000021749	Oit1	oncoprotein induced transcript 1	-3.11
ENSMUSG00000074207	Adh1	alcohol dehydrogenase 1 (class I)	-3.12
ENSMUSG00000028457	Atp8b5	ATPase, class I, type 8B, member 5	-3.12
ENSMUSG00000055027	Smyd1	SET and MYND domain containing 1	-3.13
ENSMUSG00000024371	C2	complement component 2 (within H-2S)	-3.14
ENSMUSG00000085941	Gm11201	predicted gene 11201	-3.14
ENSMUSG00000116288	Gm41349	predicted gene, 41349	-3.14
ENSMUSG00000060615	Ang4	angiogenin, ribonuclease A family, member 4	-3.15
ENSMUSG00000098557	Kctd12	potassium channel tetramerisation domain containing 12	-3.15
ENSMUSG00000073406	H2-Bl	histocompatibility 2, blastocyst	-3.15
ENSMUSG00000033740	St18	suppression of tumorigenicity 18	-3.16
ENSMUSG00000040896	Kcnd3	potassium voltage-gated channel, Shal-related family, member 3	-3.16
ENSMUSG00000070424	Art5	ADP-ribosyltransferase 5	-3.16
ENSMUSG00000103356	Gm37595	predicted gene, 37595	-3.17
ENSMUSG00000108260	Gm3793	predicted gene 3793	-3.17
ENSMUSG00000027254	Map1a	microtubule-associated protein 1 A	-3.18
ENSMUSG00000039878	Slc39a5	solute carrier family 39 (metal ion transporter), member 5	-3.18
ENSMUSG00000094584	Ms4a18	membrane-spanning 4-domains, subfamily A, member 18	-3.18
ENSMUSG00000061414	Cracr2a	calcium release activated channel regulator 2A	-3.19
ENSMUSG00000034057	Myrfl	myelin regulatory factor-like	-3.20
ENSMUSG00000090125	Pou3f1	POU domain, class 3, transcription factor 1	-3.20
ENSMUSG00000042770	Hebp1	heme binding protein 1	-3.21
ENSMUSG00000038580	Sct	secretin	-3.22
ENSMUSG00000072623	Zfp9	zinc finger protein 9	-3.22
ENSMUSG00000027359	Slc27a2	solute carrier family 27 (fatty acid transporter), member 2	-3.23
ENSMUSG00000078942	Naip6	NLR family, apoptosis inhibitory protein 6	-3.24
ENSMUSG00000033715	Akr1c14	aldo-keto reductase family 1, member C14	-3.25
ENSMUSG00000049154	Fam183b	family with sequence similarity 183, member B	-3.25
ENSMUSG00000001604	Tcea3	transcription elongation factor A (SII), 3	-3.26
ENSMUSG00000029273	Sult1d1	sulfotransferase family 1D, member 1	-3.26
ENSMUSG00000022383	Ppara	peroxisome proliferator activated receptor alpha	-3.27
ENSMUSG00000030786	Itgam	integrin alpha M	-3.27
ENSMUSG00000072723	Gm10044	predicted gene 10044	-3.28
ENSMUSG00000030303	Far2	fatty acyl CoA reductase 2	-3.29
ENSMUSG00000027474	Ccm2l	cerebral cavernous malformation 2-like	-3.29
ENSMUSG00000023247	Guca2a	guanylate cyclase activator 2a (guanylin)	-3.30
ENSMUSG00000030560	Ctsc	cathepsin C	-3.30
ENSMUSG00000029095	Ablim2	actin-binding LIM protein 2	-3.30
ENSMUSG00000049892	Rasd1	RAS, dexamethasone-induced 1	-3.30
ENSMUSG00000021806	Nid2	nidogen 2	-3.30
ENSMUSG00000005640	Insrr	insulin receptor-related receptor	-3.30
ENSMUSG00000021675	F2rl2	coagulation factor II (thrombin) receptor-like 2	-3.31
ENSMUSG00000108818	Gm44869	predicted gene 44869	-3.32
ENSMUSG00000052026	Slc6a7	solute carrier family 6 (neurotransmitter transporter, L-proline), member 7	-3.33
ENSMUSG00000033453	Adamts15	a disintegrin-like and metallopeptidase (reprolysin type) with thrombospondin type 1 motif, 15	-3.33
ENSMUSG00000114576	Naip3-ps1	NLR family, apoptosis inhibitory protein 3, pseudogene 1	-3.33

ENSMUSG00000042388	Dlgap3	DLG associated protein 3	-3.33
ENSMUSG00000050556	Kcnb1	potassium voltage gated channel, Shab-related subfamily, member 1	-3.33
ENSMUSG00000096852	Cyp2d12	cytochrome P450, family 2, subfamily d, polypeptide 12	-3.33
ENSMUSG00000071424	Grid2	glutamate receptor, ionotropic, delta 2	-3.33
ENSMUSG00000031551	Ido1	indoleamine 2,3-dioxygenase 1	-3.34
ENSMUSG00000048373	Fgfbp1	fibroblast growth factor binding protein 1	-3.34
ENSMUSG00000061808	Ttr	transthyretin	-3.34
ENSMUSG00000048217	Nags	N-acetylglutamate synthase	-3.35
ENSMUSG00000020120	Plek	pleckstrin	-3.35
ENSMUSG00000044827	Tlr1	toll-like receptor 1	-3.35
ENSMUSG00000056973	Ces1d	carboxylesterase 1D	-3.35
ENSMUSG00000031444	F10	coagulation factor X	-3.35
ENSMUSG00000011008	Mcoln2	mucolipin 2	-3.36
ENSMUSG00000031725	Ces1f	carboxylesterase 1F	-3.36
ENSMUSG00000035200	Chrnb4	cholinergic receptor, nicotinic, beta polypeptide 4	-3.37
ENSMUSG00000074441	Gm15292	predicted gene 15292	-3.39
ENSMUSG00000068323	Slc4a5	solute carrier family 4, sodium bicarbonate cotransporter, member 5	-3.39
ENSMUSG00000020805	Slc13a5	solute carrier family 13 (sodium-dependent citrate transporter), member 5	-3.40
ENSMUSG00000027801	Tm4sf4	transmembrane 4 superfamily member 4	-3.41
ENSMUSG00000027661	Slc2a10	solute carrier family 2 (facilitated glucose transporter), member 10	-3.41
ENSMUSG00000108169	Gm43958	predicted gene, 43958	-3.41
ENSMUSG00000110397	Gm45540	predicted gene 45540	-3.42
ENSMUSG00000027983	Cyp2u1	cytochrome P450, family 2, subfamily u, polypeptide 1	-3.43
ENSMUSG00000020589	Fam49a	family with sequence similarity 49, member A	-3.43
ENSMUSG00000074437	Defa29	defensin, alpha, 29	-3.44
ENSMUSG00000114443	Gm19241	predicted gene, 19241	-3.44
ENSMUSG00000032015	Pou2f3	POU domain, class 2, transcription factor 3	-3.45
ENSMUSG00000041794	Myrip	myosin VIIA and Rab interacting protein	-3.46
ENSMUSG00000023092	Fhl1	four and a half LIM domains 1	-3.46
ENSMUSG00000104841	Gm17743	predicted gene, 17743	-3.46
ENSMUSG00000042351	Grap2	GRB2-related adaptor protein 2	-3.46
ENSMUSG00000026610	Esrrg	estrogen-related receptor gamma	-3.47
ENSMUSG00000097254	C430042M11Rik	RIKEN cDNA C430042M11 gene	-3.47
ENSMUSG00000015966	Il17rb	interleukin 17 receptor B	-3.48
ENSMUSG00000020732	Rab37	RAB37, member RAS oncogene family	-3.48
ENSMUSG00000026811	St6galnac6	ST6 (alpha-N-acetyl-neuraminyl-2,3-beta-galactosyl-1,3)-N-acetylgalactosaminide alpha-2,6-sialyltra	-3.49
ENSMUSG00000052861	Dnah6	dynein, axonemal, heavy chain 6	-3.49
ENSMUSG00000107425	Gm44216	predicted gene, 44216	-3.49
ENSMUSG00000038665	Dgki	diacylglycerol kinase, iota	-3.50
ENSMUSG00000024215	Spdef	SAM pointed domain containing ets transcription factor	-3.50
ENSMUSG00000048473	Sult6b2	sulfotransferase family 6B, member 2	-3.51
ENSMUSG00000044860	Gm1123	predicted gene 1123	-3.52
ENSMUSG00000002565	Scin	scinderin	-3.52
ENSMUSG00000022840	Adcy5	adenylate cyclase 5	-3.52
ENSMUSG00000026834	Acvr1c	activin A receptor, type IC	-3.52
ENSMUSG00000064036	Mro	maestro	-3.52
ENSMUSG00000020434	4921536K21Rik	RIKEN cDNA 4921536K21 gene	-3.52
ENSMUSG00000074715	Ccl28	chemokine (C-C motif) ligand 28	-3.53
ENSMUSG00000031410	Nxf7	nuclear RNA export factor 7	-3.53
ENSMUSG00000021730	Hcn1	hyperpolarization-activated, cyclic nucleotide-gated K+ 1	-3.53
ENSMUSG00000082485	Gm15693	predicted gene 15693	-3.53
ENSMUSG00000105975	Gm42609	predicted gene 42609	-3.54
ENSMUSG00000032202	Rab27a	RAB27A, member RAS oncogene family	-3.55
ENSMUSG00000116383	Apol10c-ps	apolipoprotein L 10C, pseudogene	-3.55
ENSMUSG00000095457	Gm8989	predicted gene 8989	-3.55
ENSMUSG00000008193	Spib	Spi-B transcription factor (Spi-1/PU.1 related)	-3.56
ENSMUSG00000036492	Rnf39	ring finger protein 39	-3.56
ENSMUSG00000017311	Pyy	peptide YY	-3.56
ENSMUSG00000045930	Clec14a	C-type lectin domain family 14, member a	-3.56
ENSMUSG00000021624	Cd180	CD180 antigen	-3.57
ENSMUSG00000105105	Gm31363	predicted gene, 31363	-3.57
ENSMUSG00000030302	Atp2b2	ATPase, Ca++ transporting, plasma membrane 2	-3.59
ENSMUSG00000042115	Klhdc8a	kelch domain containing 8A	-3.59
ENSMUSG00000103320	Gm37403	predicted gene, 37403	-3.59
ENSMUSG00000056293	Gsdmc2	gasdermin C2	-3.60
ENSMUSG00000078776	9530053A07Rik	RIKEN cDNA 9530053A07 gene	-3.60
ENSMUSG00000072849	Serpina1e	serine (or cysteine) peptidase inhibitor, clade A, member 1E	-3.60
ENSMUSG00000066687	Zbtb16	zinc finger and BTB domain containing 16	-3.61
ENSMUSG00000038599	Capn8	calpain 8	-3.62
ENSMUSG00000060063	Alox5ap	arachidonate 5-lipoxygenase activating protein	-3.62
ENSMUSG00000030228	Pik3c2g	phosphatidylinositol-4-phosphate 3-kinase catalytic subunit type 2 gamma	-3.62
ENSMUSG00000021700	Rab3c	RAB3C, member RAS oncogene family	-3.63
ENSMUSG00000050100	Hmx2	H6 homeobox 2	-3.64
ENSMUSG00000068154	Insm1	insulinoma-associated 1	-3.64
ENSMUSG00000025738	Fbxl16	F-box and leucine-rich repeat protein 16	-3.64
ENSMUSG00000061958	Gm14851	predicted gene 14851	-3.65

ENSMUSG00000106651	Gm42608	predicted gene 42608	-3.65
ENSMUSG00000107296	Gm43500	predicted gene 43500	-3.65
ENSMUSG00000027797	Dclk1	doublecortin-like kinase 1	-3.66
ENSMUSG00000090062	Galnt6os	polypeptide N-acetylgalactosaminyltransferase 6, opposite strand	-3.66
ENSMUSG00000085184	4933439K11Rik	RIKEN cDNA 4933439K11 gene	-3.66
ENSMUSG00000020901	Pik3r5	phosphoinositide-3-kinase regulatory subunit 5	-3.67
ENSMUSG00000060224	Pyroxd2	pyridine nucleotide-disulphide oxidoreductase domain 2	-3.67
ENSMUSG00000020279	Il9r	interleukin 9 receptor	-3.67
ENSMUSG00000001985	Grik3	glutamate receptor, ionotropic, kainate 3	-3.67
ENSMUSG00000029445	Hpd	4-hydroxyphenylpyruvic acid dioxygenase	-3.68
ENSMUSG00000033200	Tpsg1	tryptase gamma 1	-3.69
ENSMUSG00000056220	Pla2g4a	phospholipase A2, group IVA (cytosolic, calcium-dependent)	-3.69
ENSMUSG00000101581	C430002N11Rik	RIKEN cDNA C430002N11 gene	-3.69
ENSMUSG00000085451	Gm40614	predicted gene, 40614	-3.69
ENSMUSG00000070332	Trim80	tripartite motif-containing 80	-3.69
ENSMUSG00000030737	Slco2b1	solute carrier organic anion transporter family, member 2b1	-3.70
ENSMUSG00000113800	2210039B01Rik	RIKEN cDNA 2210039B01 gene	-3.70
ENSMUSG00000019890	Nts	neurotensin	-3.71
ENSMUSG00000107456	Gm10400	predicted gene 10400	-3.71
ENSMUSG00000060070	Defa26	defensin, alpha, 26	-3.72
ENSMUSG00000058216	Gstp3	glutathione S-transferase pi 3	-3.73
ENSMUSG00000026815	Gfi1b	growth factor independent 1B	-3.74
ENSMUSG00000058740	Kcnt1	potassium channel, subfamily T, member 1	-3.75
ENSMUSG00000095026	Gm3336	predicted gene 3336	-3.76
ENSMUSG00000089948	Far2os1	fatty acyl CoA reductase 2, opposite strand 1	-3.76
ENSMUSG00000055748	Gsdmc4	gasdermin C4	-3.77
ENSMUSG00000079180	Mptx2	mucosal pentraxin 2	-3.77
ENSMUSG00000098426	Gm27149	predicted gene 27149	-3.77
ENSMUSG00000022148	Fyb	FYN binding protein	-3.78
ENSMUSG00000028415	Spink4	serine peptidase inhibitor, Kazal type 4	-3.78
ENSMUSG00000026544	Dusp23	dual specificity phosphatase 23	-3.79
ENSMUSG00000034107	Ano7	anoctamin 7	-3.80
ENSMUSG00000036242	Armh4	armadillo-like helical domain containing 4	-3.80
ENSMUSG00000055827	Gsdmc3	gasdermin C3	-3.81
ENSMUSG00000082956	Naip3	NLR family, apoptosis inhibitory protein 3	-3.82
ENSMUSG00000047517	Dmbt1	deleted in malignant brain tumors 1	-3.84
ENSMUSG00000085651	Gm11695	predicted gene 11695	-3.84
ENSMUSG00000031891	Hsd11b2	hydroxysteroid 11-beta dehydrogenase 2	-3.85
ENSMUSG00000037953	A4gnt	alpha-1,4-N-acetylglucosaminyltransferase	-3.85
ENSMUSG00000110101	Gm6249	predicted gene 6249	-3.85
ENSMUSG00000073043	Atoh1	atonal bHLH transcription factor 1	-3.86
ENSMUSG00000026288	Inpp5d	inositol polyphosphate-5-phosphatase D	-3.87
ENSMUSG00000040121	Rep15	RAB15 effector protein	-3.88
ENSMUSG00000104291	A130071D04Rik	RIKEN cDNA A130071D04 gene	-3.88
ENSMUSG00000084174	Sycn	syncollin	-3.88
ENSMUSG00000102615	Gm37844	predicted gene, 37844	-3.89
ENSMUSG00000039264	Gimap3	GTPase, IMAP family member 3	-3.90
ENSMUSG00000111377	Gm40634	predicted gene, 40634	-3.90
ENSMUSG00000046460	Sh2d7	SH2 domain containing 7	-3.91
ENSMUSG00000050321	Neto1	neuropilin (NRP) and tolloid (TLL)-like 1	-3.91
ENSMUSG00000041476	Smpx	small muscle protein, X-linked	-3.91
ENSMUSG00000009246	Trpm5	transient receptor potential cation channel, subfamily M, member 5	-3.92
ENSMUSG00000004933	Matk	megakaryocyte-associated tyrosine kinase	-3.92
ENSMUSG00000111340	Gm47171	predicted gene, 47171	-3.92
ENSMUSG00000026452	Syt2	synaptotagmin II	-3.92
ENSMUSG00000027762	Sucnr1	succinate receptor 1	-3.93
ENSMUSG00000056553	Ptprn2	protein tyrosine phosphatase, receptor type, N polypeptide 2	-3.94
ENSMUSG00000063903	Klk1	kallikrein 1	-3.94
ENSMUSG00000110405	Gm45534	predicted gene 45534	-3.95
ENSMUSG00000020581	Agr2	anterior gradient 2	-3.96
ENSMUSG00000036027	1810046K07Rik	RIKEN cDNA 1810046K07 gene	-3.96
ENSMUSG00000020788	Atp2a3	ATPase, Ca++ transporting, ubiquitous	-3.97
ENSMUSG00000026832	Cytip	cytohesin 1 interacting protein	-4.00
ENSMUSG00000040478	Prdm13	PR domain containing 13	-4.00
ENSMUSG00000034472	Rasd2	RASD family, member 2	-4.02
ENSMUSG00000022686	B3gnt5	UDP-GlcNAc:betaGal beta-1,3-N-acetylglucosaminyltransferase 5	-4.02
ENSMUSG00000058260	Serpina9	serine (or cysteine) peptidase inhibitor, clade A (alpha-1 antiproteinase, antitrypsin), member 9	-4.04
ENSMUSG00000034701	Neurod1	neurogenic differentiation 1	-4.05
ENSMUSG00000024553	Galr1	galanin receptor 1	-4.05
ENSMUSG00000037106	Fer1l6	fer-1-like 6 (C. elegans)	-4.06
ENSMUSG00000040061	Plcb2	phospholipase C, beta 2	-4.06
ENSMUSG00000054422	Fabp1	fatty acid binding protein 1, liver	-4.07
ENSMUSG00000096146	Kcnj11	potassium inwardly rectifying channel, subfamily J, member 11	-4.07
ENSMUSG00000031377	Bmx	BMX non-receptor tyrosine kinase	-4.08
ENSMUSG00000030263	Lrmp	lymphoid-restricted membrane protein	-4.09
ENSMUSG00000039372	38047	membrane-associated ring finger (C3HC4) 4	-4.09

ENSMUSG00000034353	Ramp1	receptor (calcitonin) activity modifying protein 1	-4.10
ENSMUSG00000044156	Hepacam2	HEPACAM family member 2	-4.15
ENSMUSG00000084325	Gm15550	predicted gene 15550	-4.16
ENSMUSG00000047730	Fcgbp	Fc fragment of IgG binding protein	-4.17
ENSMUSG00000001827	Folr1	folate receptor 1 (adult)	-4.17
ENSMUSG00000006586	Runx1t1	runt-related transcription factor 1; translocated to, 1 (cyclin D-related)	-4.17
ENSMUSG00000087801	Gm25980	predicted gene, 25980	-4.19
ENSMUSG00000023122	Sult1c2	sulfotransferase family, cytosolic, 1C, member 2	-4.19
ENSMUSG00000039629	Strip2	striatin interacting protein 2	-4.20
ENSMUSG00000032561	Acpp	acid phosphatase, prostate	-4.20
ENSMUSG00000061171	Slc38a11	solute carrier family 38, member 11	-4.20
ENSMUSG00000004952	Rasa4	RAS p21 protein activator 4	-4.21
ENSMUSG00000029335	Bmp3	bone morphogenetic protein 3	-4.21
ENSMUSG00000030207	Fam234b	family with sequence similarity 234, member B	-4.21
ENSMUSG00000042096	Dao	D-amino acid oxidase	-4.21
ENSMUSG00000028186	Uox	urate oxidase	-4.21
ENSMUSG00000078964	Ces1b	carboxylesterase 1B	-4.21
ENSMUSG00000026828	Galnt5	polypeptide N-acetylgalactosaminyltransferase 5	-4.22
ENSMUSG00000087518	Gm13561	predicted gene 13561	-4.22
ENSMUSG00000047564	Krtap3-1	keratin associated protein 3-1	-4.23
ENSMUSG00000043673	Kcns3	potassium voltage-gated channel, delayed-rectifier, subfamily S, member 3	-4.23
ENSMUSG00000051079	Rgs13	regulator of G-protein signaling 13	-4.24
ENSMUSG00000021069	Pygl	liver glycogen phosphorylase	-4.24
ENSMUSG00000058618	AY761184	cDNA sequence AY761184	-4.24
ENSMUSG00000017007	Rbpjl	recombination signal binding protein for immunoglobulin kappa J region-like	-4.24
ENSMUSG00000038528	Mfsd4b5	major facilitator superfamily domain containing 4B5	-4.24
ENSMUSG00000064272	Gpbar1	G protein-coupled bile acid receptor 1	-4.25
ENSMUSG00000049350	Zg16	zymogen granule protein 16	-4.27
ENSMUSG00000024029	Tff3	trefoil factor 3, intestinal	-4.28
ENSMUSG00000061959	Ces1e	carboxylesterase 1E	-4.28
ENSMUSG00000026395	Ptprc	protein tyrosine phosphatase, receptor type, C	-4.29
ENSMUSG00000056032	BC018473	cDNA sequence BC018473	-4.29
ENSMUSG00000018776	Slc35g3	solute carrier family 35, member G3	-4.29
ENSMUSG00000032036	Kirrel3	kirre like nephrin family adhesion molecule 3	-4.31
ENSMUSG00000051606	2010001K21Rik	RIKEN cDNA 2010001K21 gene	-4.32
ENSMUSG00000044453	Ffar1	free fatty acid receptor 1	-4.32
ENSMUSG00000079242	C730034F03Rik	RIKEN cDNA C730034F03 gene	-4.32
ENSMUSG00000071489	Ptgdr	prostaglandin D receptor	-4.32
ENSMUSG00000035930	Chst4	carbohydrate (chondroitin 6/keratan) sulfotransferase 4	-4.33
ENSMUSG00000006313	Upk1a	uroplakin 1A	-4.33
ENSMUSG00000082211	Defa27	defensin, alpha, 27	-4.34
ENSMUSG00000045826	Ptprcap	protein tyrosine phosphatase, receptor type, C polypeptide-associated protein	-4.34
ENSMUSG00000090174	Gm10612	predicted gene 10612	-4.34
ENSMUSG00000031173	Otc	ornithine transcarbamylase	-4.35
ENSMUSG00000046314	Stxbp6	syntaxin binding protein 6 (amisyn)	-4.35
ENSMUSG00000105302	Gm19817	predicted gene, 19817	-4.37
ENSMUSG00000078137	Ankrd63	ankyrin repeat domain 63	-4.37
ENSMUSG00000033220	Rac2	Rac family small GTPase 2	-4.38
ENSMUSG00000025515	Muc2	mucin 2	-4.40
ENSMUSG00000052631	Sh2d6	SH2 domain containing 6	-4.41
ENSMUSG00000084384	Gm12251	predicted gene 12251	-4.41
ENSMUSG00000049036	Tmem121	transmembrane protein 121	-4.46
ENSMUSG00000056468	5730596B20Rik	RIKEN cDNA 5730596B20 gene	-4.47
ENSMUSG00000095649	Gm8979	predicted gene 8979	-4.48
ENSMUSG00000109244	Gm44751	predicted gene 44751	-4.48
ENSMUSG00000000049	Apoh	apolipoprotein H	-4.48
ENSMUSG00000101031	Ms4a12	membrane-spanning 4-domains, subfamily A, member 12	-4.49
ENSMUSG00000019429	Ffar3	free fatty acid receptor 3	-4.51
ENSMUSG00000025991	Cps1	carbamoyl-phosphate synthetase 1	-4.53
ENSMUSG00000037973	Ccdc129	coiled-coil domain containing 129	-4.54
ENSMUSG00000029865	Sval1	seminal vesicle antigen-like 1	-4.54
ENSMUSG00000032087	Dscaml1	DS cell adhesion molecule like 1	-4.54
ENSMUSG00000003283	Hck	hemopoietic cell kinase	-4.55
ENSMUSG00000064140	Trim38	tripartite motif-containing 38	-4.55
ENSMUSG00000028255	Clca1	chloride channel accessory 1	-4.56
ENSMUSG00000047798	Cd300lf	CD300 molecule like family member F	-4.57
ENSMUSG00000100022	Gm29590	predicted gene 29590	-4.58
ENSMUSG00000044359	P2ry4	pyrimidinergic receptor P2Y, G-protein coupled, 4	-4.60
ENSMUSG00000079588	Tmem182	transmembrane protein 182	-4.61
ENSMUSG00000049122	Frmd3	FERM domain containing 3	-4.62
ENSMUSG00000068231	Vmn1r43	vomeronasal 1 receptor 43	-4.62
ENSMUSG00000025701	Alox5	arachidonate 5-lipoxygenase	-4.63
ENSMUSG00000040148	Hmx3	H6 homeobox 3	-4.64
ENSMUSG00000040136	Abcc8	ATP-binding cassette, sub-family C (CFTR/MRP), member 8	-4.64
ENSMUSG00000044505	Lingo4	leucine rich repeat and Ig domain containing 4	-4.64
ENSMUSG00000018927	Ccl6	chemokine (C-C motif) ligand 6	-4.65

ENSMUSG00000034645	Zyg11a	zyg-11 family member A, cell cycle regulator	-4.67
ENSMUSG00000026163	Sphkap	SPHK1 interactor, AKAP domain containing	-4.68
ENSMUSG00000040298	Btbd16	BTB (POZ) domain containing 16	-4.68
ENSMUSG00000027220	Syt13	synaptotagmin XIII	-4.71
ENSMUSG00000089669	Tnfsf13	tumor necrosis factor (ligand) superfamily, member 13	-4.71
ENSMUSG00000027296	Itpka	inositol 1,4,5-trisphosphate 3-kinase A	-4.72
ENSMUSG00000052955	Cpvl	carboxypeptidase, vitellogenic-like	-4.75
ENSMUSG00000071178	Serpina1b	serine (or cysteine) preptidase inhibitor, clade A, member 1B	-4.77
ENSMUSG00000028339	Col15a1	collagen, type XV, alpha 1	-4.78
ENSMUSG00000031981	Capn9	calpain 9	-4.79
ENSMUSG00000066176	Gm12511	predicted gene 12511	-4.79
ENSMUSG00000027168	Pax6	paired box 6	-4.83
ENSMUSG00000039013	Siglecf	sialic acid binding Ig-like lectin F	-4.84
ENSMUSG00000022489	Pde1b	phosphodiesterase 1B, Ca2+-calmodulin dependent	-4.85
ENSMUSG00000097266	Gm26802	predicted gene, 26802	-4.86
ENSMUSG00000090066	1110002E22Rik	RIKEN cDNA 1110002E22 gene	-4.92
ENSMUSG00000086513	9130208D14Rik	RIKEN cDNA 9130208D14 gene	-4.94
ENSMUSG00000070704	Ugt2b36	UDP glucuronosyltransferase 2 family, polypeptide B36	-4.95
ENSMUSG00000085095	Gm15635	predicted gene 15635	-4.95
ENSMUSG00000108950	9130015G15Rik	RIKEN cDNA 9130015G15 gene	-4.96
ENSMUSG00000029797	Sspo	SCO-spondin	-4.97
ENSMUSG00000028777	Gnat3	guanine nucleotide binding protein, alpha transducing 3	-5.00
ENSMUSG00000040345	Arhgap9	Rho GTPase activating protein 9	-5.02
ENSMUSG00000042010	Acacb	acetyl-Coenzyme A carboxylase beta	-5.04
ENSMUSG00000022026	Olfm4	olfactomedin 4	-5.08
ENSMUSG00000037627	Rgs22	regulator of G-protein signalling 22	-5.12
ENSMUSG00000110195	Pde2a	phosphodiesterase 2A, cGMP-stimulated	-5.14
ENSMUSG00000040035	Disp2	dispatched RND tramsporter family member 2	-5.19
ENSMUSG00000109685	Gm45912	predicted gene 45912	-5.20
ENSMUSG00000050711	Scg2	secretogranin II	-5.24
ENSMUSG00000034762	Glis1	GLIS family zinc finger 1	-5.34
ENSMUSG00000034923	Ly6g6f	lymphocyte antigen 6 complex, locus G6F	-5.37
ENSMUSG00000034115	Scn11a	sodium channel, voltage-gated, type XI, alpha	-5.39
ENSMUSG00000032826	Ank2	ankyrin 2, brain	-5.43
ENSMUSG00000057400	Ces1c	carboxylesterase 1C	-5.49
ENSMUSG00000040680	Kremen2	kringle containing transmembrane protein 2	-5.61
ENSMUSG00000055235	Wdr86	WD repeat domain 86	-5.61
ENSMUSG00000051497	Kcnj16	potassium inwardly-rectifying channel, subfamily J, member 16	-5.62
ENSMUSG00000096630	Vmn2r26	vomeronasal 2, receptor 26	-5.63
ENSMUSG00000021919	Chat	choline acetyltransferase	-5.68
ENSMUSG00000107624	Gm44005	predicted gene, 44005	-5.68
ENSMUSG00000090254	Gm1965	predicted gene 1965	-5.70
ENSMUSG00000021070	Bdkrb2	bradykinin receptor, beta 2	-5.70
ENSMUSG00000035277	Arx	aristaless related homeobox	-5.75
ENSMUSG00000028996	Rbp7	retinol binding protein 7, cellular	-5.83
ENSMUSG00000012819	Cdh23	cadherin 23 (otocadherin)	-5.88
ENSMUSG00000106354	Gm42607	predicted gene 42607	-5.89
ENSMUSG00000020787	P2rx1	purinergic receptor P2X, ligand-gated ion channel, 1	-5.98
ENSMUSG00000002100	Mybpc3	myosin binding protein C, cardiac	-5.99
ENSMUSG00000022650	Retnlb	resistin like beta	-6.04
ENSMUSG00000029878	Dbpht2	DNA binding protein with his-thr domain	-6.04
ENSMUSG00000033569	Adgrb3	adhesion G protein-coupled receptor B3	-6.12
ENSMUSG00000046318	Ccbe1	collagen and calcium binding EGF domains 1	-6.22
ENSMUSG00000046215	Rprml	reprimo-like	-6.27
ENSMUSG00000040380	Cbln3	cerebellin 3 precursor protein	-6.31
ENSMUSG00000050824	Sstr5	somatostatin receptor 5	-6.44
ENSMUSG00000115816	Gm34589	predicted gene, 34589	-6.45
ENSMUSG00000102759	Gm10463	predicted gene 10463	-6.62
ENSMUSG00000109237	9130214F15Rik	RIKEN cDNA 9130214F15 gene	-6.99
ENSMUSG00000026531	Mptx1	mucosal pentraxin 1	-7.01
ENSMUSG00000024366	Gfra3	glial cell line derived neurotrophic factor family receptor alpha 3	-7.18
ENSMUSG00000032532	Cck	cholecystokinin	-7.26
ENSMUSG00000021536	Adcy2	adenylate cyclase 2	-7.91
ENSMUSG00000053852	Adgrg4	adhesion G protein-coupled receptor G4	-8.21
ENSMUSG00000026938	Fcna	ficolin A	-8.30
ENSMUSG00000047497	Adamts12	a disintegrin-like and metallopeptidase (reprolysin type) with thrombospondin type 1 motif, 12	-8.61
ENSMUSG00000000394	Gcg	glucagon	-8.91
ENSMUSG00000014351	Gip	gastric inhibitory polypeptide	-9.22
ENSMUSG00000026532	Spta1	spectrin alpha, erythrocytic 1	-9.57

Tom+Trop2- vs. differentiated cells

Ensemble gene code	Symbol	Gene name	logFC
ENSMUSG00000031841	Cdh13	cadherin 13	8.43
ENSMUSG00000035615	Frmpd1	FERM and PDZ domain containing 1	7.13
ENSMUSG00000020388	Pdlim4	PDZ and LIM domain 4	6.99
ENSMUSG00000013415	Igf2bp1	insulin-like growth factor 2 mRNA binding protein 1	6.96
ENSMUSG00000037035	Inhbb	inhibin beta-B	6.67
ENSMUSG00000042268	Slc26a9	solute carrier family 26, member 9	6.58
ENSMUSG00000040852	Plekhh2	pleckstrin homology domain containing, family H (with MyTH4 domain) member 2	6.55
ENSMUSG00000079330	Lemd1	LEM domain containing 1	6.42
ENSMUSG00000029368	Alb	albumin	6.38
ENSMUSG00000031871	Cdh5	cadherin 5	6.23
ENSMUSG00000025330	Padi4	peptidyl arginine deiminase, type IV	6.19
ENSMUSG00000028031	Dkk2	dickkopf WNT signaling pathway inhibitor 2	6.14
ENSMUSG00000055322	Tns1	tensin 1	6.13
ENSMUSG00000017491	Rarb	retinoic acid receptor, beta	6.12
ENSMUSG00000053613	Notumos	notum palmitoleoyl-protein carboxylesterase, opposite strand	6.11
ENSMUSG00000029371	Cxcl5	chemokine (C-X-C motif) ligand 5	6.04
ENSMUSG00000023885	Thbs2	thrombospondin 2	6.02
ENSMUSG00000029838	Ptn	pleiotrophin	6.02
ENSMUSG00000031503	Col4a2	collagen, type IV, alpha 2	6.02
ENSMUSG00000032502	Stac	src homology three (SH3) and cysteine rich domain	5.95
ENSMUSG00000097651	4930461G14Rik	RIKEN cDNA 4930461G14 gene	5.82
ENSMUSG00000012428	Steap4	STEAP family member 4	5.74
ENSMUSG00000022883	Robo1	roundabout guidance receptor 1	5.70
ENSMUSG00000029223	Uchl1	ubiquitin carboxy-terminal hydrolase L1	5.68
ENSMUSG00000022754	Tmem45a	transmembrane protein 45a	5.55
ENSMUSG00000097767	Miat	myocardial infarction associated transcript (non-protein coding)	5.48
ENSMUSG00000068699	Flnc	filamin C, gamma	5.35
ENSMUSG00000054263	Lifr	leukemia inhibitory factor receptor	5.34
ENSMUSG00000024268	Celf4	CUGBP, Elav-like family member 4	5.27
ENSMUSG00000036480	Prss56	protease, serine 56	5.25
ENSMUSG00000028360	Slc44a5	solute carrier family 44, member 5	5.22
ENSMUSG00000059898	Dsc3	desmocollin 3	5.16
ENSMUSG00000026167	Wnt10a	wingless-type MMTV integration site family, member 10A	5.14
ENSMUSG00000110290	Gm45336	predicted gene 45336	5.06
ENSMUSG00000021091	Serpina3n	serine (or cysteine) peptidase inhibitor, clade A, member 3N	4.94
ENSMUSG00000029869	Ephb6	Eph receptor B6	4.92
ENSMUSG00000033377	Palmd	palmdelphin	4.84
ENSMUSG00000110187	Gm45496	predicted gene 45496	4.74
ENSMUSG00000051159	Cited1	Cbp/p300-interacting transactivator with Glu/Asp-rich carboxy-terminal domain 1	4.72
ENSMUSG00000022464	Slc38a4	solute carrier family 38, member 4	4.69
ENSMUSG00000052516	Robo2	roundabout guidance receptor 2	4.64
ENSMUSG00000038415	Foxq1	forkhead box Q1	4.59
ENSMUSG00000032965	Ift57	intraflagellar transport 57	4.44
ENSMUSG00000026117	Zap70	zeta-chain (TCR) associated protein kinase	4.41
ENSMUSG00000060962	Dmkn	dermokine	4.32
ENSMUSG00000016763	Scube1	signal peptide, CUB domain, EGF-like 1	4.28
ENSMUSG00000005503	Evx1	even-skipped homeobox 1	4.22
ENSMUSG00000042988	Notum	notum palmitoleoyl-protein carboxylesterase	4.16
ENSMUSG00000032643	Fhl3	four and a half LIM domains 3	4.16
ENSMUSG00000024810	Il33	interleukin 33	4.15
ENSMUSG00000021950	Anxa8	annexin A8	4.12
ENSMUSG00000108897	Gm44861	predicted gene 44861	4.11
ENSMUSG00000039232	Stx11	syntaxin 11	4.04
ENSMUSG00000031661	Nkd1	naked cuticle 1	4.03
ENSMUSG00000054555	Adam12	a disintegrin and metallopeptidase domain 12 (meltrin alpha)	4.03
ENSMUSG00000004552	Ctse	cathepsin E	3.97
ENSMUSG00000030772	Dkk3	dickkopf WNT signaling pathway inhibitor 3	3.96
ENSMUSG00000071531	Gprin2	G protein regulated inducer of neurite outgrowth 2	3.96
ENSMUSG00000067818	Myl9	myosin, light polypeptide 9, regulatory	3.93
ENSMUSG00000037712	Fermt2	fermitin family member 2	3.89
ENSMUSG00000026950	Neb	nebulin	3.87
ENSMUSG00000040488	Ltbp4	latent transforming growth factor beta binding protein 4	3.83
ENSMUSG00000066607	Insyn1	inhibitory synaptic factor 1	3.79
ENSMUSG00000051236	Msrb3	methionine sulfoxide reductase B3	3.78
ENSMUSG00000032492	Pth1r	parathyroid hormone 1 receptor	3.73
ENSMUSG00000045991	Onecut2	one cut domain, family member 2	3.72
ENSMUSG00000000031	H19	H19, imprinted maternally expressed transcript	3.71
ENSMUSG00000035164	Zc3h12c	zinc finger CCCH type containing 12C	3.69

ENSMUSG00000030911	Zp2	zona pellucida glycoprotein 2	3.69
ENSMUSG00000048078	Tenm4	teneurin transmembrane protein 4	3.67
ENSMUSG00000041046	Ramp3	receptor (calcitonin) activity modifying protein 3	3.61
ENSMUSG00000022176	Rem2	rad and gem related GTP binding protein 2	3.57
ENSMUSG00000038860	Garnl3	GTPase activating RANGAP domain-like 3	3.57
ENSMUSG00000004668	Abca13	ATP-binding cassette, sub-family A (ABC1), member 13	3.54
ENSMUSG00000034460	Six4	sine oculis-related homeobox 4	3.53
ENSMUSG00000022037	Clu	clusterin	3.48
ENSMUSG00000049866	Arl4c	ADP-ribosylation factor-like 4C	3.48
ENSMUSG00000040836	Gpr161	G protein-coupled receptor 161	3.44
ENSMUSG00000063531	Sema3e	sema domain, immunoglobulin domain (Ig), short basic domain, secreted, (semaphorin) 3E	3.41
ENSMUSG00000013584	Aldh1a2	aldehyde dehydrogenase family 1, subfamily A2	3.38
ENSMUSG00000062327	T	brachyury, T-box transcription factor T	3.36
ENSMUSG00000037016	Frem2	Fras1 related extracellular matrix protein 2	3.33
ENSMUSG00000034684	Sema3f	sema domain, immunoglobulin domain (Ig), short basic domain, secreted, (semaphorin) 3F	3.26
ENSMUSG00000030048	Gkn3	gastrokine 3	3.26
ENSMUSG00000038146	Notch3	notch 3	3.25
ENSMUSG00000078235	Fam43b	family with sequence similarity 43, member B	3.25
ENSMUSG00000048450	Msx1	msh homeobox 1	3.16
ENSMUSG00000026579	F5	coagulation factor V	3.13
ENSMUSG00000029769	Ccdc136	coiled-coil domain containing 136	3.12
ENSMUSG00000006205	Htra1	HtrA serine peptidase 1	3.11
ENSMUSG00000062591	Tubb4a	tubulin, beta 4A class IVA	3.09
ENSMUSG00000027570	Col9a3	collagen, type IX, alpha 3	3.09
ENSMUSG00000039239	Tgfb2	transforming growth factor, beta 2	3.08
ENSMUSG00000027985	Lef1	lymphoid enhancer binding factor 1	3.06
ENSMUSG00000043903	Zfp469	zinc finger protein 469	3.02
ENSMUSG00000052957	Gas1	growth arrest specific 1	3.02
ENSMUSG00000058070	Eml1	echinoderm microtubule associated protein like 1	2.98
ENSMUSG00000031595	Pdgfrl	platelet-derived growth factor receptor-like	2.96
ENSMUSG00000036036	Zfp57	zinc finger protein 57	2.95
ENSMUSG00000028497	Hacd4	3-hydroxyacyl-CoA dehydratase 4	2.94
ENSMUSG00000061048	Cdh3	cadherin 3	2.94
ENSMUSG00000086484	Nron	non-protein coding RNA, repressor of NFAT	2.91
ENSMUSG00000020218	Wif1	Wnt inhibitory factor 1	2.89
ENSMUSG00000025352	Gdf11	growth differentiation factor 11	2.87
ENSMUSG00000000983	Wfdc18	WAP four-disulfide core domain 18	2.85
ENSMUSG00000048960	Prex2	phosphatidylinositol-3,4,5-trisphosphate-dependent Rac exchange factor 2	2.81
ENSMUSG00000031074	Fgf3	fibroblast growth factor 3	2.80
ENSMUSG00000038775	Vill	villin-like	2.78
ENSMUSG00000032717	Mdfi	MyoD family inhibitor	2.78
ENSMUSG00000019997	Ctgf	connective tissue growth factor	2.77
ENSMUSG00000059854	Hydin	HYDIN, axonemal central pair apparatus protein	2.75
ENSMUSG00000059991	Nptx2	neuronal pentraxin 2	2.74
ENSMUSG00000043924	Ncmap	noncompact myelin associated protein	2.72
ENSMUSG00000026193	Fn1	fibronectin 1	2.70
ENSMUSG00000055653	Gpc3	glypican 3	2.70
ENSMUSG00000032186	Tmod2	tropomodulin 2	2.68
ENSMUSG00000031502	Col4a1	collagen, type IV, alpha 1	2.67
ENSMUSG00000023008	Fmnl3	formin-like 3	2.66
ENSMUSG00000022371	Col14a1	collagen, type XIV, alpha 1	2.64
ENSMUSG00000063605	Ccdc102a	coiled-coil domain containing 102A	2.64
ENSMUSG00000017897	Eya2	EYA transcriptional coactivator and phosphatase 2	2.63
ENSMUSG00000074813	Gm14005	predicted gene 14005	2.63
ENSMUSG00000074796	Slc4a11	solute carrier family 4, sodium bicarbonate transporter-like, member 11	2.62
ENSMUSG00000021539	Lect2	leukocyte cell-derived chemotaxin 2	2.61
ENSMUSG00000062184	Hs6st2	heparan sulfate 6-O-sulfotransferase 2	2.56
ENSMUSG00000045932	Ifit2	interferon-induced protein with tetratricopeptide repeats 2	2.51
ENSMUSG00000026822	Lcn2	lipocalin 2	2.50
ENSMUSG00000029674	Limk1	LIM-domain containing, protein kinase	2.50
ENSMUSG00000049848	Ceacam19	carcinoembryonic antigen-related cell adhesion molecule 19	2.50
ENSMUSG00000032625	Thsd7a	thrombospondin, type I, domain containing 7A	2.49
ENSMUSG00000088609	Gm24187	predicted gene, 24187	2.47
ENSMUSG00000060548	Tnfrsf19	tumor necrosis factor receptor superfamily, member 19	2.46
ENSMUSG00000070867	Trabd2b	TraB domain containing 2B	2.46
ENSMUSG00000020844	Nxn	nucleoredoxin	2.44
ENSMUSG00000038295	Atg9b	autophagy related 9B	2.43
ENSMUSG00000063727	Tnfrsf11b	tumor necrosis factor receptor superfamily, member 11b (osteoprotegerin)	2.42
ENSMUSG00000025938	Slco5a1	solute carrier organic anion transporter family, member 5A1	2.42
ENSMUSG00000026981	Il1rn	interleukin 1 receptor antagonist	2.39
ENSMUSG00000068859	Sp9	trans-acting transcription factor 9	2.39

ENSMUSG00000052105	Mtcl1	microtubule crosslinking factor 1	2.38
ENSMUSG00000055945	Prr18	proline rich 18	2.38
ENSMUSG00000058297	Spock2	sparc/osteonectin, cwcv and kazal-like domains proteoglycan 2	2.37
ENSMUSG00000040998	Npnt	nephronectin	2.33
ENSMUSG00000109245	Gm44860	predicted gene 44860	2.33
ENSMUSG00000059336	Slc14a1	solute carrier family 14 (urea transporter), member 1	2.31
ENSMUSG00000058952	Cfi	complement component factor i	2.28
ENSMUSG00000040663	Clcf1	cardiotrophin-like cytokine factor 1	2.23
ENSMUSG00000020937	Plcd3	phospholipase C, delta 3	2.19
ENSMUSG00000040415	Dtx3	deltex 3, E3 ubiquitin ligase	2.19
ENSMUSG00000024659	Anxa1	annexin A1	2.15
ENSMUSG00000027068	Dhrs9	dehydrogenase/reductase (SDR family) member 9	2.13
ENSMUSG00000046402	Rbp1	retinol binding protein 1, cellular	2.12
ENSMUSG00000028364	Tnc	tenascin C	2.11
ENSMUSG00000025492	Ifitm3	interferon induced transmembrane protein 3	2.10
ENSMUSG00000085903	Gm15340	predicted gene 15340	2.10
ENSMUSG00000032875	Arhgef17	Rho guanine nucleotide exchange factor (GEF) 17	2.09
ENSMUSG00000030796	Tead2	TEA domain family member 2	2.08
ENSMUSG00000040808	S100g	S100 calcium binding protein G	2.08
ENSMUSG00000016942	Tmprss6	transmembrane serine protease 6	2.07
ENSMUSG00000078161	Erich3	glutamate rich 3	2.06
ENSMUSG00000015647	Lama5	laminin, alpha 5	2.02
ENSMUSG00000048191	Muc6	mucin 6, gastric	2.01
ENSMUSG00000030223	Ptpro	protein tyrosine phosphatase, receptor type, O	2.01
ENSMUSG00000040430	Pitpnc1	phosphatidylinositol transfer protein, cytoplasmic 1	2.01
ENSMUSG00000020086	H2afy2	H2A histone family, member Y2	1.98
ENSMUSG00000025491	Ifitm1	interferon induced transmembrane protein 1	1.97
ENSMUSG00000032076	Cadm1	cell adhesion molecule 1	1.95
ENSMUSG00000050989	Selenon	selenoprotein N	1.94
ENSMUSG00000036123	Slc9a3	solute carrier family 9 (sodium/hydrogen exchanger), member 3	1.93
ENSMUSG00000038578	Susd1	sushi domain containing 1	1.92
ENSMUSG00000010175	Prox1	prospero homeobox 1	1.90
ENSMUSG00000032068	Plet1	placenta expressed transcript 1	1.90
ENSMUSG00000039607	Rbms3	RNA binding motif, single stranded interacting protein	1.89
ENSMUSG00000026888	Grb14	growth factor receptor bound protein 14	1.89
ENSMUSG00000036902	Neto2	neuropilin (NRP) and tolloid (TLL)-like 2	1.87
ENSMUSG00000051343	Rab11fip5	RAB11 family interacting protein 5 (class I)	1.87
ENSMUSG00000014599	Csf1	colony stimulating factor 1 (macrophage)	1.86
ENSMUSG00000060181	Slc35e3	solute carrier family 35, member E3	1.85
ENSMUSG00000040118	Cacna2d1	calcium channel, voltage-dependent, alpha2/delta subunit 1	1.81
ENSMUSG00000020646	Mboat2	membrane bound O-acyltransferase domain containing 2	1.79
ENSMUSG00000025089	Gfra1	glial cell line derived neurotrophic factor family receptor alpha 1	1.79
ENSMUSG00000026728	Vim	vimentin	1.78
ENSMUSG00000068923	Syt11	synaptotagmin XI	1.78
ENSMUSG00000008398	Elk3	ELK3, member of ETS oncogene family	1.76
ENSMUSG00000026676	Ccdc3	coiled-coil domain containing 3	1.76
ENSMUSG00000071847	Apcdd1	adenomatosis polyposis coli down-regulated 1	1.75
ENSMUSG00000041801	Phlda3	pleckstrin homology like domain, family A, member 3	1.75
ENSMUSG00000040187	Arntl2	aryl hydrocarbon receptor nuclear translocator-like 2	1.74
ENSMUSG00000060429	Sntb1	syntrophin, basic 1	1.73
ENSMUSG00000013089	Etv5	ets variant 5	1.71
ENSMUSG00000017466	Timp2	tissue inhibitor of metalloproteinase 2	1.70
ENSMUSG00000017493	Igfbp4	insulin-like growth factor binding protein 4	1.69
ENSMUSG00000026509	Capn2	calpain 2	1.69
ENSMUSG00000049823	Zbtb12	zinc finger and BTB domain containing 12	1.69
ENSMUSG00000043439	Epop	elongin BC and polycomb repressive complex 2 associated protein	1.67
ENSMUSG00000062661	Ncs1	neuronal calcium sensor 1	1.67
ENSMUSG00000021822	Plau	plasminogen activator, urokinase	1.67
ENSMUSG00000037071	Scd1	stearoyl-Coenzyme A desaturase 1	1.65
ENSMUSG00000072437	Nanos1	nanos C2HC-type zinc finger 1	1.65
ENSMUSG00000049807	Arhgap23	Rho GTPase activating protein 23	1.64
ENSMUSG00000081016	Olfr1397-ps1	olfactory receptor 1397, pseudogene 1	1.63
ENSMUSG00000014592	Camta1	calmodulin binding transcription activator 1	1.62
ENSMUSG00000034220	Gpc1	glypican 1	1.60
ENSMUSG00000022146	Osmr	oncostatin M receptor	1.60
ENSMUSG00000037379	Spon2	spondin 2, extracellular matrix protein	1.58
ENSMUSG00000036667	Tcaf1	TRPM8 channel-associated factor 1	1.57
ENSMUSG00000037664	Cdkn1c	cyclin-dependent kinase inhibitor 1C (P57)	1.57
ENSMUSG00000001521	Tulp3	tubby-like protein 3	1.57
ENSMUSG00000025479	Cyp2e1	cytochrome P450, family 2, subfamily e, polypeptide 1	1.57
ENSMUSG00000081683	Fzd10	frizzled class receptor 10	1.56

ENSMUSG00000063522	2010109I03Rik	RIKEN cDNA 2010109I03 gene	1.54
ENSMUSG00000067889	Sptbn2	spectrin beta, non-erythrocytic 2	1.52
ENSMUSG00000028654	Mycl	v-myc avian myelocytomatosis viral oncogene lung carcinoma derived	1.52
ENSMUSG00000055333	Fat2	FAT atypical cadherin 2	1.52
ENSMUSG00000039109	F13a1	coagulation factor XIII, A1 subunit	1.51
ENSMUSG00000027999	Pla2g12a	phospholipase A2, group XIIA	1.50
ENSMUSG00000030409	Dmpk	dystrophia myotonica-protein kinase	1.49
ENSMUSG00000078949	R3hdml	R3H domain containing-like	1.47
ENSMUSG00000031328	Flna	filamin, alpha	1.46
ENSMUSG00000085329	2810404F17Rik	RIKEN cDNA 2810404F17 gene	1.46
ENSMUSG00000058908	Pla2g2a	phospholipase A2, group IIA (platelets, synovial fluid)	1.45
ENSMUSG00000037095	Lrg1	leucine-rich alpha-2-glycoprotein 1	1.45
ENSMUSG00000029359	Tesc	tescalcin	1.43
ENSMUSG00000000782	Tcf7	transcription factor 7, T cell specific	1.41
ENSMUSG00000013846	St3gal1	ST3 beta-galactoside alpha-2,3-sialyltransferase 1	1.41
ENSMUSG00000033149	Phldb2	pleckstrin homology like domain, family B, member 2	1.40
ENSMUSG00000032698	Lmo2	LIM domain only 2	1.40
ENSMUSG00000027698	Nceh1	neutral cholesterol ester hydrolase 1	1.39
ENSMUSG00000025902	Sox17	SRY (sex determining region Y)-box 17	1.39
ENSMUSG00000048776	Pthlh	parathyroid hormone-like peptide	1.37
ENSMUSG00000048058	Ldlrad3	low density lipoprotein receptor class A domain containing 3	1.34
ENSMUSG00000026620	Mark1	MAP/microtubule affinity regulating kinase 1	1.34
ENSMUSG00000028613	Lrp8	low density lipoprotein receptor-related protein 8, apolipoprotein e receptor	1.33
ENSMUSG00000039621	Prex1	phosphatidylinositol-3,4,5-trisphosphate-dependent Rac exchange factor 1	1.33
ENSMUSG00000038151	Prdm1	PR domain containing 1, with ZNF domain	1.32
ENSMUSG00000032232	Cgnl1	cingulin-like 1	1.28
ENSMUSG00000029832	Nfe2l3	nuclear factor, erythroid derived 2, like 3	1.28
ENSMUSG00000038400	Pmepa1	prostate transmembrane protein, androgen induced 1	1.24
ENSMUSG00000044033	Ccdc141	coiled-coil domain containing 141	1.24
ENSMUSG00000017204	Gsdma	gasdermin A	1.23
ENSMUSG00000008090	Fgfrl1	fibroblast growth factor receptor-like 1	1.20
ENSMUSG00000060487	Samd5	sterile alpha motif domain containing 5	1.20
ENSMUSG00000013275	Slc41a1	solute carrier family 41, member 1	1.19
ENSMUSG00000014602	Kif1a	kinesin family member 1A	1.19
ENSMUSG00000027257	Pacsin3	protein kinase C and casein kinase substrate in neurons 3	1.18
ENSMUSG00000068547	Clca4a	chloride channel accessory 4A	1.17
ENSMUSG00000025608	Podxl	podocalyxin-like	1.17
ENSMUSG00000025075	Habp2	hyaluronic acid binding protein 2	1.16
ENSMUSG00000038740	Mvb12b	multivesicular body subunit 12B	1.16
ENSMUSG00000001473	Tubb6	tubulin, beta 6 class V	1.16
ENSMUSG00000035678	Tnfsf9	tumor necrosis factor (ligand) superfamily, member 9	1.15
ENSMUSG00000021493	Pdlim7	PDZ and LIM domain 7	1.14
ENSMUSG00000028268	Gbp3	guanylate binding protein 3	1.13
ENSMUSG00000028836	Slc30a2	solute carrier family 30 (zinc transporter), member 2	1.10
ENSMUSG00000045136	Tubb2b	tubulin, beta 2B class IIB	1.10
ENSMUSG00000097494	4933406C10Rik	RIKEN cDNA 4933406C10 gene	1.10
ENSMUSG00000030352	Tspan9	tetraspanin 9	1.07
ENSMUSG00000054793	Cadm4	cell adhesion molecule 4	1.03
ENSMUSG00000050295	Foxc1	forkhead box C1	1.03
ENSMUSG00000047945	Marcksl1	MARCKS-like 1	1.01
ENSMUSG00000020186	Csrp2	cysteine and glycine-rich protein 2	-1.06
ENSMUSG00000095325	Zfp870	zinc finger protein 870	-1.09
ENSMUSG00000095649	Gm8979	predicted gene 8979	-1.11
ENSMUSG00000086513	9130208D14Rik	RIKEN cDNA 9130208D14 gene	-1.23
ENSMUSG00000061958	Gm14851	predicted gene 14851	-1.24
ENSMUSG00000053846	Lipg	lipase, endothelial	-1.48
ENSMUSG00000066438	Plekhd1	pleckstrin homology domain containing, family D (with coiled-coil domains) member 1	-1.51
ENSMUSG00000109685	Gm45912	predicted gene 45912	-1.68
ENSMUSG00000026531	Mptx1	mucosal pentraxin 1	-2.13
ENSMUSG00000104918	Gm42944	predicted gene 42944	-2.19
ENSMUSG00000033209	Ttc28	tetratricopeptide repeat domain 28	-2.83

Supplementary Table S7

Overlap of differentially expressed genes in Trop2+RFP/tdTomato+ vs.

Trop2-RFP/tdTomato+ neoplastic cells obtained in hyperplastic epithelium and adenomas 7 days and 6 weeks after Apc inactivation, respectively.

Overlap of differentially expressed genes in $Trop2^+$ $RFP/tdTomato^+$ vs $Trop2^-$ $RFP/tdTomato^+$ neoplastic cells obtained in hyperplastic epithelium and adenomas 7 days and 6 weeks after Apc inactivation, respectively. Genes with adjusted p-value < 0.05 and $|\log_2 FC| \geq 1$ experiments were used for comparison.

Upregulated genes

Ensemble gene code	Symbol	Gene name	logFC hyperplasia	logFC microadenoma
ENSMUSG00000051397	Tacstd2	tumor-associated calcium signal transducer 2	6.26	6.34
ENSMUSG00000004951	Hspb1	heat shock protein 1	2.09	5.49
ENSMUSG00000108218	Olfr1372-ps1	olfactory receptor 1372, pseudogene 1	2.38	5.08
ENSMUSG00000053007	Creb5	cAMP responsive element binding protein 5	2.11	4.6
ENSMUSG00000028031	Dkk2	dickkopf WNT signaling pathway inhibitor 2	2.02	4.56
ENSMUSG00000029648	Flt1	FMS-like tyrosine kinase 1	3.69	4.52
ENSMUSG00000062591	Tubb4a	tubulin, beta 4A class IVA	2.43	4.43
ENSMUSG00000034220	Gpc1	glypican 1	1.95	4.43
ENSMUSG00000025473	Adam8	a disintegrin and metallopeptidase domain 8	2.45	4.39
ENSMUSG00000026167	Wnt10a	wingless-type MMTV integration site family, member 10A	1.85	4.34
ENSMUSG00000022037	Clu	clusterin	4.57	4.32
ENSMUSG00000046623	Gjb4	gap junction protein, beta 4	2.49	4.31
ENSMUSG00000034463	Scara3	scavenger receptor class A, member 3	3.71	4.2
ENSMUSG00000037188	Grhl3	grainyhead like transcription factor 3	3.54	4.12
ENSMUSG00000018340	Anxa6	annexin A6	2.19	4.11
ENSMUSG00000085412	Halr1	Hoxa adjacent long noncoding RNA 1	2.06	4.1
ENSMUSG00000009097	Tbx1	T-box 1	1.73	4.09
ENSMUSG00000027210	Meis2	Meis homeobox 2	1.71	4.05
ENSMUSG00000031538	Plat	plasminogen activator, tissue	1.94	3.97
ENSMUSG00000039232	Stx11	syntaxin 11	1.83	3.88
ENSMUSG00000049807	Arhgap23	Rho GTPase activating protein 23	1.12	3.84
ENSMUSG00000039153	Runx2	runt related transcription factor 2	2.93	3.79
ENSMUSG00000043430	Psapl1	prosaposin-like 1	2.01	3.79
ENSMUSG00000032496	Ltf	lactotransferrin	1.5	3.77
ENSMUSG00000025330	Padi4	peptidyl arginine deiminase, type IV	1.79	3.75
ENSMUSG00000048960	Prex2	phosphatidylinositol-3,4,5-trisphosphate-dependent Rac exchange factor 2	1.58	3.73
ENSMUSG00000022665	Ccdc80	coiled-coil domain containing 80	1.19	3.73
ENSMUSG00000021765	Fst	follistatin	3.64	3.7
ENSMUSG00000063727	Tnfrsf11b	tumor necrosis factor receptor superfamily, member 11b (osteoprotegerin)	2.48	3.61
ENSMUSG00000041608	Entpd3	ectonucleoside triphosphate diphosphohydrolase 3	1.43	3.54
ENSMUSG00000009628	Tex15	testis expressed gene 15	1.21	3.53
ENSMUSG00000020651	Slc26a4	solute carrier family 26, member 4	2.35	3.47
ENSMUSG00000029761	Cald1	caldesmon 1	1.72	3.46
ENSMUSG00000041189	Chrnb1	cholinergic receptor, nicotinic, beta polypeptide 1 (muscle)	1.17	3.46
ENSMUSG00000034634	Ly6d	lymphocyte antigen 6 complex, locus D	2.18	3.43
ENSMUSG00000031925	Maml2	mastermind like transcriptional coactivator 2	1.29	3.41
ENSMUSG00000040998	Npnt	nephronectin	3.5	3.4
ENSMUSG00000025938	Slco5a1	solute carrier organic anion transporter family, member 5A1	2.46	3.39
ENSMUSG00000032186	Tmod2	tropomodulin 2	1.68	3.39
ENSMUSG00000053137	Mapk11	mitogen-activated protein kinase 11	2.06	3.32
ENSMUSG00000024912	Fosl1	fos-like antigen 1	1.21	3.27
ENSMUSG00000004552	Ctse	cathepsin E	2.95	3.26
ENSMUSG00000050640	Tmem150c	transmembrane protein 150C	2.7	3.26
ENSMUSG00000013338	Fer1l4	fer-1-like 4 (C. elegans)	2.77	3.23
ENSMUSG00000055333	Fat2	FAT atypical cadherin 2	2.75	3.23
ENSMUSG00000026620	Mark1	MAP/microtubule affinity regulating kinase 1	2.73	3.2
ENSMUSG00000080316	Spaca6	sperm acrosome associated 6	2.15	3.18
ENSMUSG00000031841	Cdh13	cadherin 13	4.27	3.16
ENSMUSG00000038587	Akap12	A kinase (PRKA) anchor protein (gravin) 12	2.21	3.14
ENSMUSG00000004044	Cavin1	caveolae associated 1	1.82	3.13
ENSMUSG00000040152	Thbs1	thrombospondin 1	2.1	3.12
ENSMUSG00000000983	Wfdc18	WAP four-disulfide core domain 18	1.95	3.12
ENSMUSG00000020674	Pxdn	peroxidasin	1.08	3.11
ENSMUSG00000027376	Prom2	prominin 2	3.33	3.09
ENSMUSG00000034295	Fhod3	formin homology 2 domain containing 3	1.1	3.06
ENSMUSG00000046449	Nexmif	neurite extension and migration factor	2.5	3.04
ENSMUSG00000048078	Tenm4	teneurin transmembrane protein 4	1.51	3.04
ENSMUSG00000049866	Arl4c	ADP-ribosylation factor-like 4C	1.01	3.03
ENSMUSG00000031328	Flna	filamin, alpha	1.65	3.02
ENSMUSG00000013076	Amotl1	angiomotin-like 1	1.26	2.98
ENSMUSG00000026193	Fn1	fibronectin 1	2.46	2.95
ENSMUSG00000046731	Kctd11	potassium channel tetramerisation domain containing 11	1.19	2.95
ENSMUSG00000024659	Anxa1	annexin A1	4.59	2.93
ENSMUSG00000037833	Sh2d4b	SH2 domain containing 4B	3.16	2.91
ENSMUSG00000024940	Ltbp3	latent transforming growth factor beta binding protein 3	1.12	2.91
ENSMUSG00000033227	Wnt6	wingless-type MMTV integration site family, member 6	2.59	2.9
ENSMUSG00000023008	Fmnl3	formin-like 3	1.63	2.89
ENSMUSG00000020181	Nav3	neuron navigator 3	1.27	2.89
ENSMUSG00000029869	Ephb6	Eph receptor B6	2.67	2.88
ENSMUSG00000032717	Mdfi	MyoD family inhibitor	1.35	2.88
ENSMUSG00000034573	Ptpn13	protein tyrosine phosphatase, non-receptor type 13	3.29	2.86
ENSMUSG00000067889	Sptbn2	spectrin beta, non-erythrocytic 2	1.98	2.85
ENSMUSG00000031871	Cdh5	cadherin 5	1.94	2.83

ENSMUSG00000002980	Bcam	basal cell adhesion molecule	1.59	2.83
ENSMUSG00000001288	Rarg	retinoic acid receptor, gamma	2.72	2.82
ENSMUSG00000022176	Rem2	rad and gem related GTP binding protein 2	1.23	2.82
ENSMUSG00000041390	Mdfic	MyoD family inhibitor domain containing	1.25	2.8
ENSMUSG00000057193	Slc44a2	solute carrier family 44, member 2	1.01	2.8
ENSMUSG00000035678	Tnfsf9	tumor necrosis factor (ligand) superfamily, member 9	1.47	2.78
ENSMUSG00000040430	Pitpnc1	phosphatidylinositol transfer protein, cytoplasmic 1	1.91	2.77
ENSMUSG00000069255	Dusp22	dual specificity phosphatase 22	1.16	2.73
ENSMUSG00000038415	Foxq1	forkhead box Q1	1.54	2.7
ENSMUSG00000032327	Stra6	stimulated by retinoic acid gene 6	3.35	2.68
ENSMUSG00000020427	Igfbp3	insulin-like growth factor binding protein 3	1.57	2.68
ENSMUSG00000032875	Arhgef17	Rho guanine nucleotide exchange factor (GEF) 17	1.36	2.67
ENSMUSG00000033377	Palmd	palmdelphin	4.44	2.65
ENSMUSG00000040488	Ltbp4	latent transforming growth factor beta binding protein 4	2.58	2.65
ENSMUSG00000015134	Aldh1a3	aldehyde dehydrogenase family 1, subfamily A3	3.3	2.62
ENSMUSG00000040836	Gpr161	G protein-coupled receptor 161	1.91	2.61
ENSMUSG00000021493	Pdlim7	PDZ and LIM domain 7	1.72	2.6
ENSMUSG00000061517	Sox21	SRY (sex determining region Y)-box 21	3.2	2.57
ENSMUSG00000090394	4930523C07Rik	RIKEN cDNA 4930523C07 gene	1.43	2.56
ENSMUSG00000006411	Nectin4	nectin cell adhesion molecule 4	1.87	2.54
ENSMUSG00000027257	Pacsin3	protein kinase C and casein kinase substrate in neurons 3	1.2	2.44
ENSMUSG00000059668	Krt4	keratin 4	4.76	2.42
ENSMUSG00000009687	Fxyd5	FXYD domain-containing ion transport regulator 5	2.59	2.42
ENSMUSG00000008398	Elk3	ELK3, member of ETS oncogene family	2.27	2.4
ENSMUSG00000024401	Tnf	tumor necrosis factor	1.72	2.4
ENSMUSG00000019853	Hebp2	heme binding protein 2	1.16	2.36
ENSMUSG00000022371	Col14a1	collagen, type XIV, alpha 1	1.46	2.33
ENSMUSG00000024810	Il33	interleukin 33	1.28	2.32
ENSMUSG00000052105	Mtcl1	microtubule crosslinking factor 1	1.65	2.31
ENSMUSG00000041570	Camsap2	calmodulin regulated spectrin-associated protein family, member 2	1.4	2.26
ENSMUSG00000029832	Nfe2l3	nuclear factor, erythroid derived 2, like 3	1.08	2.26
ENSMUSG00000082361	Btc	betacellulin, epidermal growth factor family member	1.28	2.2
ENSMUSG00000001435	Col18a1	collagen, type XVIII, alpha 1	2.34	2.17
ENSMUSG00000042306	S100a14	S100 calcium binding protein A14	1.89	2.15
ENSMUSG00000031441	Atp11a	ATPase, class VI, type 11A	1.05	2.14
ENSMUSG00000039457	Ppl	periplakin	1.7	2.13
ENSMUSG00000048058	Ldlrad3	low density lipoprotein receptor class A domain containing 3	1.27	2.12
ENSMUSG00000001473	Tubb6	tubulin, beta 6 class V	1.1	2.12
ENSMUSG00000032013	Trim29	tripartite motif-containing 29	1.96	2.11
ENSMUSG00000032643	Fhl3	four and a half LIM domains 3	1.16	2.09
ENSMUSG00000022754	Tmem45a	transmembrane protein 45a	2.56	2.08
ENSMUSG00000023885	Thbs2	thrombospondin 2	1.59	2.08
ENSMUSG00000017723	Wfdc2	WAP four-disulfide core domain 2	1.7	2.07
ENSMUSG00000026405	C4bp	complement component 4 binding protein	2.24	2.05
ENSMUSG00000110218	Gm20219	predicted gene, 20219	1.23	2.03
ENSMUSG00000037946	Fgd3	FYVE, RhoGEF and PH domain containing 3	3.28	2.02
ENSMUSG00000003541	Ier3	immediate early response 3	1.41	2.01
ENSMUSG00000037060	Cavin3	caveolae associated 3	2.29	1.98
ENSMUSG00000021477	Ctsl	cathepsin L	1.34	1.98
ENSMUSG00000027381	Bcl2l11	BCL2-like 11 (apoptosis facilitator)	1.2	1.97
ENSMUSG00000038775	Vill	villin-like	1.89	1.94
ENSMUSG00000030352	Tspan9	tetraspanin 9	1.23	1.94
ENSMUSG00000040990	Sh3kbp1	SH3-domain kinase binding protein 1	2.67	1.92
ENSMUSG00000007039	Ddah2	dimethylarginine dimethylaminohydrolase 2	2.05	1.92
ENSMUSG00000022178	Ajuba	ajuba LIM protein	1.7	1.87
ENSMUSG00000026478	Lamc1	laminin, gamma 1	1.01	1.87
ENSMUSG00000025921	Rdh10	retinol dehydrogenase 10 (all-trans)	1.83	1.83
ENSMUSG00000062661	Ncs1	neuronal calcium sensor 1	1.63	1.82
ENSMUSG00000026065	Slc9a4	solute carrier family 9 (sodium/hydrogen exchanger), member 4	3.37	1.79
ENSMUSG00000027712	Anxa5	annexin A5	2.12	1.79
ENSMUSG00000046589	Lrrc8e	leucine rich repeat containing 8 family, member E	1.54	1.79
ENSMUSG00000027858	Tspan2	tetraspanin 2	1.76	1.78
ENSMUSG00000027860	Vangl1	VANGL planar cell polarity 1	1.07	1.77
ENSMUSG00000025372	Baiap2	brain-specific angiogenesis inhibitor 1-associated protein 2	1.34	1.76
ENSMUSG00000047501	Cldn4	claudin 4	2.13	1.71
ENSMUSG00000028464	Tpm2	tropomyosin 2, beta	1.82	1.71
ENSMUSG00000031430	Vsig1	V-set and immunoglobulin domain containing 1	2.76	1.7
ENSMUSG00000022505	Emp2	epithelial membrane protein 2	1.97	1.69
ENSMUSG00000027737	Slc7a11	solute carrier family 7 (cationic amino acid transporter, y+ system), member 11	1.61	1.68
ENSMUSG00000038295	Atg9b	autophagy related 9B	1.06	1.66
ENSMUSG00000055407	Map6	microtubule-associated protein 6	2.93	1.64
ENSMUSG00000031586	Rbpms	RNA binding protein gene with multiple splicing	2.02	1.63
ENSMUSG00000006777	Krt23	keratin 23	1.63	1.62
ENSMUSG00000079330	Lemd1	LEM domain containing 1	2.11	1.6
ENSMUSG00000027485	Bpifb1	BPI fold containing family B, member 1	6.53	1.57
ENSMUSG00000006435	Neurl1a	neuralized E3 ubiquitin protein ligase 1A	1.09	1.55

ENSMUSG00000001025	S100a6	S100 calcium binding protein A6 (calcyclin)	2.11	1.52
ENSMUSG00000017897	Eya2	EYA transcriptional coactivator and phosphatase 2	1.52	1.51
ENSMUSG00000069114	Zbtb10	zinc finger and BTB domain containing 10	1.02	1.49
ENSMUSG00000027907	S100a11	S100 calcium binding protein A11	1.44	1.47
ENSMUSG00000020773	Trim47	tripartite motif-containing 47	1.38	1.47
ENSMUSG00000021108	Prkch	protein kinase C, eta	1.23	1.46
ENSMUSG00000039384	Dusp10	dual specificity phosphatase 10	2.15	1.44
ENSMUSG00000030759	Far1	fatty acyl CoA reductase 1	1.38	1.44
ENSMUSG00000022055	Nefl	neurofilament, light polypeptide	2.01	1.39
ENSMUSG00000041886	Macc1	metastasis associated in colon cancer 1	1.83	1.39
ENSMUSG00000021792	Fam213a	family with sequence similarity 213, member A	2.72	1.38
ENSMUSG00000032348	Gsta4	glutathione S-transferase, alpha 4	2.55	1.3
ENSMUSG00000026335	Pam	peptidylglycine alpha-amidating monooxygenase	1.31	1.26
ENSMUSG00000035547	Capn5	calpain 5	1.07	1.26
ENSMUSG00000038463	Olfml2b	olfactomedin-like 2B	1.98	1.23
ENSMUSG00000028378	Ptgr1	prostaglandin reductase 1	1.25	1.22
ENSMUSG00000039831	Arhgap29	Rho GTPase activating protein 29	1.33	1.17
ENSMUSG00000040711	Sh3pxd2b	SH3 and PX domains 2B	1.26	1.17
ENSMUSG00000040543	Pitpnm3	PITPNM family member 3	1.45	1.16
ENSMUSG00000038387	Rras	related RAS viral (r-ras) oncogene	1.16	1.12
ENSMUSG00000089809	Rasgef1b	RasGEF domain family, member 1B	1.31	1.09
ENSMUSG00000032068	Plet1	placenta expressed transcript 1	2.88	1.08
ENSMUSG00000026796	Fam129b	family with sequence similarity 129, member B	1.01	1.06
ENSMUSG00000026509	Capn2	calpain 2	2.17	1.05
ENSMUSG00000024501	Dpysl3	dihydropyrimidinase-like 3	1.84	1.04
ENSMUSG00000030342	Cd9	CD9 antigen	1.03	1.04

Downregulated genes

Ensemble gene code	Symbol	Gene name	logFC hyperplasia	logFC microadenoma
ENSMUSG00000022650	Retnlb	resistin like beta	-1.33	-5.1
ENSMUSG00000022026	Olfm4	olfactomedin 4	-2.76	-4.12
ENSMUSG00000051079	Rgs13	regulator of G-protein signaling 13	-1.04	-4.05
ENSMUSG00000032561	Acpp	acid phosphatase, prostate	-1.44	-3.64
ENSMUSG00000086513	9130208D14Rik	RIKEN cDNA 9130208D14 gene	-1.65	-3.63
ENSMUSG00000056553	Ptprn2	protein tyrosine phosphatase, receptor type, N polypeptide 2	-1.25	-3.52
ENSMUSG00000025991	Cps1	carbamoyl-phosphate synthetase 1	-1.6	-3.48
ENSMUSG00000109685	Gm45912	predicted gene 45912	-1.85	-3.37
ENSMUSG00000031173	Otc	ornithine transcarbamylase	-1.29	-3.34
ENSMUSG00000033740	St18	suppression of tumorigenicity 18	-1.25	-3.34
ENSMUSG00000058618	AY761184	cDNA sequence AY761184	-1.33	-3.33
ENSMUSG00000028415	Spink4	serine peptidase inhibitor, Kazal type 4	-1.01	-3.3
ENSMUSG00000095649	Gm8979	predicted gene 8979	-1.75	-3.29
ENSMUSG00000029445	Hpd	4-hydroxyphenylpyruvic acid dioxygenase	-1.76	-3.2
ENSMUSG00000031891	Hsd11b2	hydroxysteroid 11-beta dehydrogenase 2	-1.04	-3.17
ENSMUSG00000030207	Fam234b	family with sequence similarity 234, member B	-1.77	-3.14
ENSMUSG00000034472	Rasd2	RASD family, member 2	-2.09	-3.07
ENSMUSG00000079180	Mptx2	mucosal pentraxin 2	-1.27	-3.04
ENSMUSG00000037953	A4gnt	alpha-1,4-N-acetylglucosaminyltransferase	-1.81	-2.94
ENSMUSG00000074715	Ccl28	chemokine (C-C motif) ligand 28	-1.19	-2.79
ENSMUSG00000005268	Prlr	prolactin receptor	-1.39	-2.75
ENSMUSG00000026610	Esrrg	estrogen-related receptor gamma	-1.13	-2.73
ENSMUSG00000074437	Defa29	defensin, alpha, 29	-1.39	-2.68
ENSMUSG00000033715	Akr1c14	aldo-keto reductase family 1, member C14	-1.15	-2.67
ENSMUSG00000046613	Vwa5b2	von Willebrand factor A domain containing 5B2	-1.06	-2.67
ENSMUSG00000052026	Slc6a7	solute carrier family 6 (neurotransmitter transporter, L-proline), member 7	-1.16	-2.63
ENSMUSG00000036422	Pcdh8	protocadherin 8	-2.6	-2.57
ENSMUSG00000027359	Slc27a2	solute carrier family 27 (fatty acid transporter), member 2	-1.66	-2.54
ENSMUSG00000028327	Stra6l	STRA6-like	-1.3	-2.5
ENSMUSG00000026398	Nr5a2	nuclear receptor subfamily 5, group A, member 2	-1.21	-2.44
ENSMUSG00000038745	Nlrp6	NLR family, pyrin domain containing 6	-1.16	-2.38
ENSMUSG00000031451	Gas6	growth arrest specific 6	-1.16	-2.38
ENSMUSG00000040412	5330417C22Rik	RIKEN cDNA 5330417C22 gene	-1.25	-2.35
ENSMUSG00000079559	Colca2	COLCA2 homolog	-1.38	-2.32
ENSMUSG00000026786	Apbb1ip	amyloid beta (A4) precursor protein-binding, family B, member 1 interacting protein	-1.56	-2.31
ENSMUSG00000109936	Gm45889	predicted gene 45889	-1.25	-2.31
ENSMUSG00000025790	Slco3a1	solute carrier organic anion transporter family, member 3a1	-1.32	-2.3
ENSMUSG00000024411	Aqp4	aquaporin 4	-2	-2.25
ENSMUSG00000004655	Aqp1	aquaporin 1	-1.36	-2.24
ENSMUSG00000025738	Fbxl16	F-box and leucine-rich repeat protein 16	-1.02	-2.24
ENSMUSG00000066687	Zbtb16	zinc finger and BTB domain containing 16	-1.89	-2.19
ENSMUSG00000060586	H2-Eb1	histocompatibility 2, class II antigen E beta	-1.04	-2.16
ENSMUSG00000026489	Coq8a	coenzyme Q8A	-1.61	-2.08
ENSMUSG00000027452	Acss1	acyl-CoA synthetase short-chain family member 1	-1.01	-2.04
ENSMUSG00000079547	H2-DMb1	histocompatibility 2, class II, locus Mb1	-1	-2.01
ENSMUSG00000052271	Bhlha15	basic helix-loop-helix family, member a15	-1.38	-1.96
ENSMUSG00000022018	Rgcc	regulator of cell cycle	-1.32	-1.89
ENSMUSG00000024225	Clps	colipase, pancreatic	-1.18	-1.88
ENSMUSG00000110266	Gm32742	predicted gene, 32742	-2.31	-1.85
ENSMUSG00000035561	Aldh1b1	aldehyde dehydrogenase 1 family, member B1	-1.61	-1.83
ENSMUSG00000060208	Defa17	defensin, alpha, 17	-1.19	-1.79
ENSMUSG00000063142	Kcnma1	potassium large conductance calcium-activated channel, subfamily M, alpha member 1	-1.08	-1.78
ENSMUSG00000022504	Ciita	class II transactivator	-1.43	-1.77
ENSMUSG00000005089	Slc1a2	solute carrier family 1 (glial high affinity glutamate transporter), member 2	-1.26	-1.77
ENSMUSG00000021830	Txndc16	thioredoxin domain containing 16	-1.09	-1.77
ENSMUSG00000058740	Kcnt1	potassium channel, subfamily T, member 1	-1.16	-1.71
ENSMUSG00000005360	Slc1a3	solute carrier family 1 (glial high affinity glutamate transporter), member 3	-1.48	-1.68
ENSMUSG00000040528	Milr1	mast cell immunoglobulin like receptor 1	-1.6	-1.55
ENSMUSG00000030934	Oat	ornithine aminotransferase	-1.1	-1.41
ENSMUSG00000033721	Vav3	vav 3 oncogene	-1.34	-1.38
ENSMUSG00000031453	Rasa3	RAS p21 protein activator 3	-1.03	-1.31
ENSMUSG00000003545	Fosb	FBJ osteosarcoma oncogene B	-1.07	-1.07
ENSMUSG00000002346	Slc25a42	solute carrier family 25, member 42	-1.21	-1.03
ENSMUSG00000074653	Lrrc31	leucine rich repeat containing 31	-1.03	-1.03
ENSMUSG00000041577	Prelp	proline arginine-rich end leucine-rich repeat	-2.09	-1
